# A fan-attached jacket worn in an environment exceeding body temperature suppresses an increase in core temperature

**DOI:** 10.1038/s41598-021-00655-2

**Published:** 2021-10-28

**Authors:** Kahori Hashimoto, Seichi Horie, Chikage Nagano, Hiroyuki Hibino, Kimiyo Mori, Kimie Fukuzawa, Masashi Nakayama, Hiroyuki Tanaka, Jinro Inoue

**Affiliations:** Department of Health Policy and Management, Institute of Industrial Ecological Sciences, University of Occupational and Environmental Health, 1-1 Iseigaoka, Yahatanishi-ku, Kitakyushu, 807-8555 Japan

**Keywords:** Health care, Occupational health, Environmental social sciences, Climate-change mitigation

## Abstract

We examined whether blowing hot air above body temperature under work clothing may suppress core temperature. Nine Japanese men engaged in two 30-min bicycle ergometer sessions at a workload of 40% VO_2_max at 40 °C and 50% relative humidity. The experiment was conducted without wearing any cooling apparatus (CON), wearing a cooling vest that circulated 10.0 °C water (VEST), and wearing a fan-attached jacket that transferred ambient air underneath the jacket at a rate of 30 L/s (FAN). The VEST and FAN conditions suppressed the increases of rectal temperature (CON, VEST, FAN; 38.01 ± 0.19 °C, 37.72 ± 0.12 °C (*p* = 0.0076), 37.54 ± 0.19 °C (*p* = 0.0023), respectively), esophageal temperature (38.22 ± 0.30 °C, 37.55 ± 0.18 °C (*p* = 0.0039), 37.54 ± 0.21 °C (*p* = 0.0039), respectively), and heart rate (157.3 ± 9.8 bpm, 136.9 ± 8.9 bpm, (*p* = 0.0042), 137.5 ± 6.5 bpm (*p* = 0.0023), respectively). Two conditions also reduced the estimated amount of sweating and improved various subjective evaluations. Even in the 40 °C and 50% relative humidity environment, we may recommend wearing a fan-attached jacket because the heat dissipation through evaporation exceeded the heat convection from the hot ambient air.

## Introduction

An increase in core body temperature can lead to heat-related illness and be detrimental to a person’s work performance^1^. Dehydration and an elevated core body temperature can also impair a person’s cognitive and physical functioning^2^. The prevention of heat-related illness is important in terms of enabling workers to continue their work without experiencing harmful health effects. Undesirably, even the drastic effort will be made to limit the global temperature increase to 1.5 °C above to pre-industrial level, the temperature in Tokyo is estimated to reach 40 °C in Tokyo in 2100^3^. Physically demanding work in extreme heat exceeding body core temperature is often inevitable in real work settings. Therefore, vest and garments equipped with cooling materials such as ice, phase change materials, and cooled water-perfusion system have been developed and reported to lower the elevation of heart rates and body temperatures and to improve the work performance. However, their cooling capacities will not last for long and expire mostly within an hour^4–6^. One of the most promising and stable devices is a cooling garment that is continuously cooled by a circulating-water cooling vest connected to a chiller; however, the garment has a hose that restricts body movements and requires electricity to operate. In Japan, the fan-attached jacket, a thin long-sleeve jacket that is similar to work clothing but has two small fans attached to the back of the waist, has become popular among workers. This jacket was designed to improve comfort by transferring ambient air under the clothing and promoting the evaporation of sweat^7,8^. It is estimated that the large volume of ventilated air may be potent enough to reduce the increase of core temperatures^9^. However, the company that developed the jacket does not recommend its use in hot, humid environments because the fan transfers hot air that can exceed the body temperature to the skin surface. The company’s homepage states that the cooling effect is insufficient at an ambient temperature (T_a_) of 35 °C and relative humidity (RH) of 50%^7^, despite similar conditions being experienced daily in Japan.

A recent study observed suppressed elevations of the average skin temperature, tympanic temperature measured with an infrared thermometer, heart rate (HR), subjective thermal sensation, and thermal discomfort when a fan-attached jacket available in Japan was worn for 30 min of repeated exercise in an environment controlled at 33 °C and 65% RH^10^. Another study observed suppressed elevations of the average skin temperature, HR, and estimated sweat volume at T_a_ of 34 °C (RH not stated) for a fan-attached jacket^11^. However, neither study examined the efficacy of the fan-attached jacket in an environment having a temperature exceeding the core body temperature. Other studies have investigated a more rigorous type of cooling jacket that provides cooled air under the clothes and demonstrated the suppression of the increased rectal temperature (T_re_), average skin temperature, HR, and estimated sweat volume as well as a longer maximum time of physical exertion^12–16^. Several studies have confirmed the cooling effect of work clothes equipped with cooled airflow and phase-change materials using human participants or thermal mannequins^17–20^. However, these studies did not use the fan-attached jacket that simply transfers ambient air to evaluate the cooling effect of the jacket. To address this knowledge gap, we investigated the efficacy of wearing a fan-attached jacket that transfers ambient air directly into the space under the work clothing while performing physically demanding labor in a 40 °C-T_a_ and 50%-RH environment.

## Methods

### Participants

The study’s participants were nine unacclimatized Japanese male adults who did not have currently or previously treated medical conditions, did not currently take medications, and had no smoking history (age: 24.4 ± 4.3 years; height: 169.6 ± 6.0 cm; body weight: 64.3 ± 7.5 kg; VO_2_max: 39.2 ± 4.68 mL/kg/min). Each participant received an explanation of the study and written informed consent has been obtained from all participants before participating.

### Experimental procedures

We compared the increases in core temperature and HR and changes in subjective assessments during two sessions of physical workload under the conditions with a cooling vest that circulated 10.0 °C water (VEST), with a fan-attached jacket that transferred ambient air underneath the jacket at a rate of 30 L/s (FAN), or without wearing any cooling apparatus (CON). In CON condition, no cooling garment was worn. In VEST condition, a cool circulating-water garment (CW-WVNCLFN, Kamakura Seisakusho, Tokyo, Japan) was worn and a circulating-water chiller was connected to maintain the water temperature inside the cooling vest. In FAN condition, a fan-attached long-sleeve jacket made from 100% woven polyester was worn, where the jacket had had two fans (10 cm in diameter, attached at the left and right sides of the back of the waist), which were set at airflow rate of 30 L/s, the strongest of four-level air volume switch equipped in this garment, and were each powered by four AA batteries (KU90720, Kuchofuku, Tokyo, Japan). This fan-attached jacket is available throughout Japan and is typically worn over a short-sleeve shirt. Under the VEST condition, the external chiller and the cooling vest were connected by a thermally insulated hose. The vest was sleeveless, made from 100% woven polyester, and had thin polyvinylchloride ducts distributed throughout the upper body. Altogether it weighed 810 g, including 90 g of water inside^21^. The chiller temperature was set at 9.3 °C to maintain the temperature of the cooling vest inlet at 10.0 ± 0.3 °C.

The thermal environment, physical workload, and underwear worn by the participants were the same for all conditions. All participants performed the exercise experiment for each of the three conditions in such a way as to cancel out any ordering effect, and each participant participated in experimental sessions that were at least 3 days apart.

All experiments were conducted between September 2018 and December 2019 in the artificial climate chamber of the Shared-Use Research Center, University of Occupational and Environmental Health (UOEH), Japan (TBR-8E20W0P2T, Espec, Osaka, Japan). The chamber was divided into two environments; one room maintained at 28 °C-T_a_ and 50%-RH and the other maintained at 40 °C-T_a_ and 50%-RH, and both rooms were kept almost windless. On the day before an experiment, each participant underwent a workload test to estimate the maximal oxygen consumption (VO_2_max) using an ergometer (T.K.K. 3070, Takei Scientific Instruments, Niigata, Japan).

We calculated the 80% heart rate reserve (HRR) as

$$ 80\% {\text{HRR}} = \left( {{\text{maximum}}\;{\text{HR}} - {\text{resting}}\;{\text{HR}}} \right) \times 0.8 + {\text{resting}}\;{\text{HR}}. $$

The ergometer workload started at 20 W and then increased by 10 W every 1 min until the 80%HRR was reached. We calculated the workload corresponding to 40%VO_2_max through linear regression analysis of the individual examination data.

Participants were instructed to sleep well and abstain from alcohol on the day before and to refrain from consuming caffeine-rich products and avoid doing exercise on the day of the experiment before participating in the experiment. The participants consumed a 400-kcal meal and 500 mL of bottled plain water 1 h before the start of the experiment.

The study design and procedures were approved by the Ethics Committee of Medical Research of UOEH, Japan (approval number H30-120). All methods were performed in accordance with the relevant guidelines and regulations. The body weight of each participant was measured without clothing, and the participant then entered a room maintained at 28 °C-T_a_ and 50%-RH while wearing underwear, a short-sleeve shirt made of 100% polyester, and long, summertime work trousers. After the participant entered the room, monitors were attached to the participant, and measurements began. After resting for 15 min in the room and putting on a long-sleeve summertime work jacket, the participant moved to a 40 °C-T_a_ and 50%-RH room and exercised on a bicycle ergometer at a workload of 40%VO_2_max for 30 min. After the first 30 min of exercise, the participant returned to the 28 °C room and rested on a chair for 15 min. During this rest period, the participant drank 10 mL/kg body weight of carbohydrate mineral solution kept at room temperature within 10 min of the start of rest, and the participant then returned to the 40 °C room for a second session of the same exercise. After the second session, the participant rested for 10 min in the 28 °C room, and the measurements were continued until a decreasing trend in T_re_ was confirmed. After the removal of the measurement devices, the body weight of the participant was measured. A physician attended all times, and the exercise was discontinued if T_re_ exceeded 38.5 °C or if the participant experienced any symptoms of heatstroke. Under the FAN condition, the participants were asked to wear the fan-attached jacket instead of the long-sleeve jacket and continued to wear it during the rest period. Under the VEST condition, the participants wore the cool circulating-water garment between the short-sleeve shirt and the long-sleeve jacket and were asked to remove the long-sleeve jacket during the rest period.

### Measurements

The T_re_, esophageal temperature (T_es_), and skin temperature of the frontal head (T_sk-head_) were continuously monitored in real time every 10 s by IEC 60584-1 Class 1 copper–constantan thermocouples, 0.32 mm, 0.10 mm, and 0.20 mm in diameter for T_re_, T_es_, and T_sk-head_, respectively, calibrated using a standard temperature device (ZC-114A, Coper Electronics Co., Ltd., Atsugi, Japan). The T_re_ probe covered with elastic sheath (P249A, NIKKISO-THERM, Tokyo, Japan) was inserted approximately 15 cm into the anus, where the monitored temperature stabilized at 37 °C. The T_es_ probe covered with smooth polyethylene tube was swallowed by mouth to a depth of 45 cm and then expelled and reinserted as needed, with the depth altered by up to 2 cm to find the correct location at which to obtain the maximum temperature. The probe was finally fixed near the mouth. The T_sk-head_ probe was placed on the front of the participant’s head. The HR was measured by an electrocardiograph, and the mean value for eight beats was recorded every 10 s. The mean breath-by-breath oxygen uptake was recorded every 10 s.

Subjective evaluations were provided ten times: during rest, immediately before starting exercise, and 10, 20, and 30 min after starting exercise in each of the two rounds (t_1–10_). The evaluation scales used were the Borg scale for the rating of perceived exertion (RPE; 7 very, very light, 9 very light, 11 fairly light, 13 somewhat hard, 15 hard, 17 very hard, 19 very, very hard); the ISO10551 scale for thermal sensation (+ 4 very hot, + 3 hot, + 2 warm, + 1 slightly warm, 0 neutral, − 1 slightly cool, − 2 cool, − 3 cold, − 4 very cold); and a simple scale of thermal comfort (4 extremely uncomfortable, 3 very uncomfortable, 2 uncomfortable, 1 slightly uncomfortable, 0 comfortable)^22^.

The estimated amount of sweating during exercise was calculated from the change in body weight before and after exercise; the weight of the liquid consumed during rest was deducted.

### Statistical analysis

For T_re_, T_es_, T_sk-head_, and HR, the average of measured values during 5 min were determined for five times: checkpoint 1 (CP1), right after the start of the first exercise session (23 min 00 s (23:00–27:50); CP2, immediately before the end of the first session (48:00–52:50); CP3, right after the start of the second exercise session (77:00–81:50); CP4, immediately before the end of the second session (102:00–106:50); and CP5, when T_re_ peaked (110:00–114:50). The T_es_ values were missing for one participant under the CON condition, and the results of this participant were excluded from the final analysis. We examined the normality of the data distributions between CP1 and CP4 using the Anderson–Darling test. We then compared the results obtained under the three conditions using the Steel–Dwass test for T_re_, T_es_, and HR; the Tukey–Krammer test for T_sk-head_; and an analysis of variance for the estimated sweat volume. P values of less than 0.05 were considered statistically significant. Temperature changes of at least 0.1 °C and HR changes of at least 5 bpm were considered to be significant differences. The statistical analysis was performed using JMP Pro v.15.

## Results

### Temperatures

**Figure 1:**
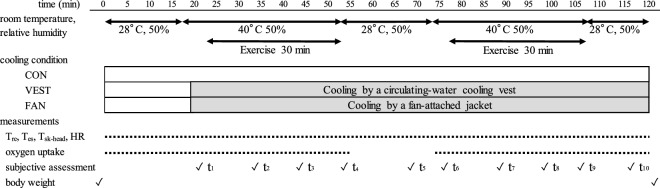
Schematic of the testing protocol. CON: participants did not wear any cooling garment, VEST: participants wore a circulating-water cooling vest, FAN: participants wore a fan-attached jacket, T_re_: rectal temperatures, T_es_: esophageal temperatures, T_sk-head_: skin temperatures of the frontal head, HR: heart rate. t_1_ and t_6_: immediately before starting exercise; t_2_–t_4_ and t_7_–t_9_: 10, 20, and 30 min, respectively, after starting exercise; t_5_ and t_10_: during rest.

**Figure 2:**
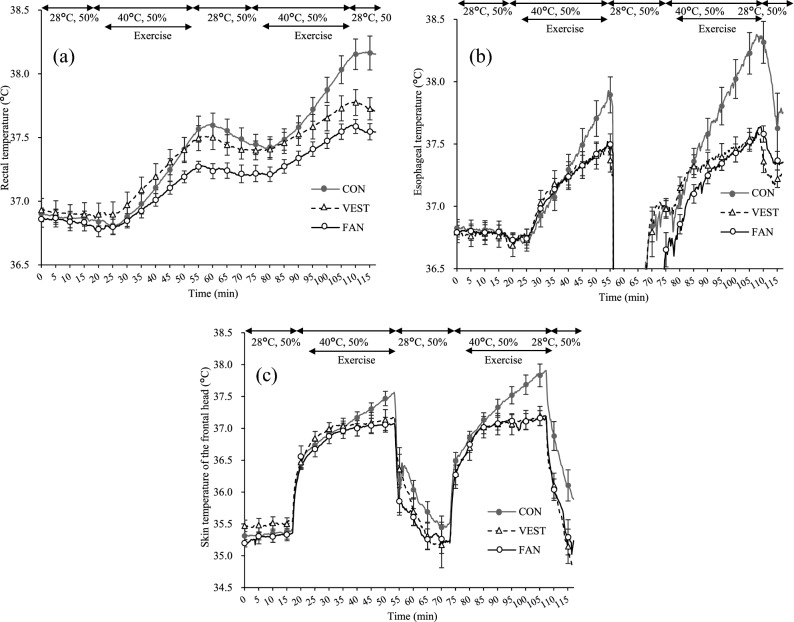
(**a**) Rectal temperature in the CON, VEST, and FAN conditions. (**b**) Esophageal temperature in the CON, VEST, and FAN conditions. (**c**) Skin temperature of the frontal head in the CON, VEST, and FAN conditions. Data at every 5 min are presented as the mean ± standard error. *CON* participants did not wear any cooling garment, *VEST* participants wore a circulating-water cooling vest, *FAN* participants wore a fan-attached jacket. Number of participants: nine, except for one missing T_es_ datapoint in the CON condition.

The T_re_, T_es_, and T_sk-head_ during two sessions of 30 min exercise at 40% of VO_2_max intensity (Fig. 1) performed in the 40 °C-T_a_ and 50%-RH environment increased slowly and the peak values were markedly suppressed under VEST or FAN conditions compared to CON condition. The mean values of T_re_, T_es_, T_sk-head_ during the period from the start of rest to 10 min after the end of exercise are shown in Fig. 2a–c, respectively (“Supplementary Information”). At the end of exercises, T_re_ was 0.3 °C lower under the VEST condition and 0.5 °C lower under the FAN condition. Immediately after entering the 40 °C-T_a_ and 50%-RH room, T_re_ slightly and temporarily decreased under the FAN and CON conditions and then gradually increased, but the T_re_ values were continuously lower than those under the VEST condition. Under the VEST condition, T_re_ increased upon entry into the 40 °C-T_a_ and 50%-RH room and was higher than that under the CON condition until nearly the end of the first exercise session. During the rest period, T_re_ under the VEST condition decreased to the same level as that under the CON condition and then increased more slowly than that under the CON condition during the second session. T_es_ under the VEST and FAN conditions had a trend similar to that seen under the CON condition, but the value was 0.7 °C lower than that under the CON condition at the end of exercise. During the second session, T_es_ under the CON condition increased more rapidly and reached a higher temperature than it did in the first session. In contrast, under the VEST and FAN conditions, the values were nearly the same as those in the first session. The T_sk-head_ value reflects the effect of the ambient temperature, and it increased when the participants moved to the 40 °C-T_a_ and 50%-RH room. However, the T_es_ values under both the VEST and FAN conditions plateaued at 37.2 °C, which was lower by as much as 0.7 °C than the corresponding value under the CON condition. Table 1 demonstrates the mean and standard deviations of T_re_, T_es_, T_sk-head_, and HR under the three conditions during 5 min at five checkpoints from CP1 to CP5. T_re_, T_es_, and T_sk-head_ were significantly different among the three conditions at CP4.

**Table 1:** Rectal temperature (T_re_), esophageal temperature (T_es_), and skin temperature of the frontal head (T_sk-head_) and heart rate (HR) at each checkpoint.

Trials	N	CP1			CP2			CP3			CP4		
		Mean	SD	*p*	Mean	SD	*p*	Mean	SD	*P*	Mean	SD	*p*
**T**_**re**_													
CON	9	36.81	0.15	Reference	37.43	0.18	Reference	37.42	0.18	Reference	38.01	0.19	Reference
VEST	9	36.90	0.19	0.485	37.42	0.17	0.996	37.41	0.12	0.934	37.72	0.12	0.0076**
FAN	9	36.80	0.21	0.996	37.20	0.19	0.070	37.21	0.19	0.056	37.54	0.19	0.0023**
**T**_**es**_													
CON	8	36.74	0.14	Reference	37.72	0.27	Reference	37.01	0.18	Reference	38.22	0.30	Reference
VEST	8	36.84	0.15	0.449	37.48	0.20	0.193	37.16	0.14	0.332	37.55	0.18	0.0039**
FAN	8	36.80	0.19	0.774	37.46	0.23	0.157	36.79	0.23	0.0055**	37.54	0.21	0.0039**
**T**_**sk-head**_													
CON	9	36.76	0.16	Reference	37.47	0.23	Reference	36.80	0.20	Reference	37.83	0.30	Reference
VEST	9	36.86	0.08	0.430	37.14	0.28	0.129	36.66	0.32	0.649	37.17	0.28	0.0072**
FAN	9	36.69	0.15	0.677	37.06	0.33	0.054	36.69	0.26	0.739	37.14	0.37	0.005**
**HR**													
CON	9	109.5	4.8	Reference	144.8	9.2	Reference	124.2	7.8	Reference	157.3	9.8	Reference
VEST	9	109.3	4.4	0.983	131.8	6.5	0.0076**	113.3	6.1	0.0057**	136.9	8.9	0.0042**
FAN	9	107.9	3.1	0.934	133.9	6.0	0.0131*	112.8	5.0	0.0031**	137.5	6.5	0.0023**

*CON* participants did not wear any cooling garment, *VEST* participants wore a circulating-water cooling vest, *FAN* participants wore a fan-attached jacket.

CP1: 23 min 00 s to 27 min 50 s, CP2: 48:00–52:50, CP3: 77:00–81:50, CP4: 102:00–106:50.

The *p* values for T_re_, T_es_, and HR were determined by the Steel–Dwass test; the *p* value for T_sk-head_ was determined by the Tukey–Kramer test.

*p < 0.05.

**p < 0.01.

### Heart rate

**Figure 3:**
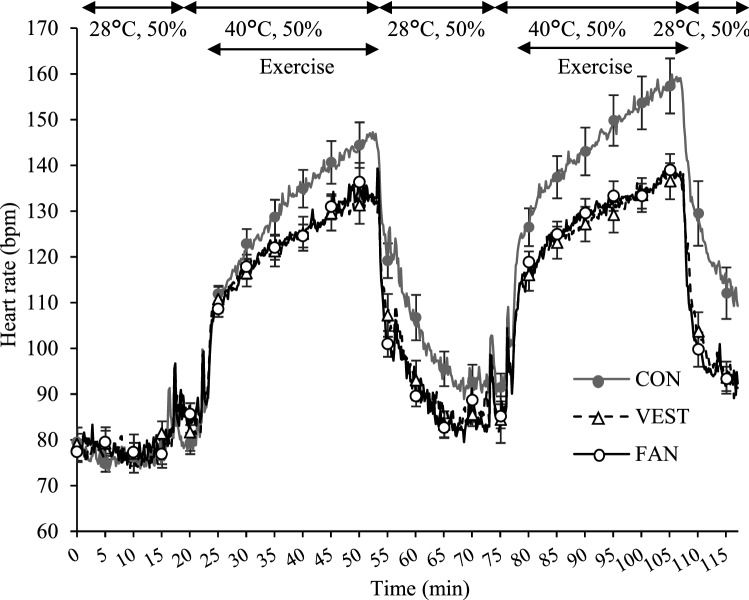
Heart rate in the CON, VEST, and FAN conditions. Data at every 5 min are presented as the mean ± standard error. *CON* participants did not wear any cooling garment, *VEST* participants wore a circulating-water cooling vest, *FAN* participants wore a fan-attached jacket. Number of participants: nine.

The HRs of participants during the period from the start of rest to 10 min after the end of exercise are shown in Fig. 3 (“Supplementary Information”). During exercise, the average HR gradually increased to nearly 160 bpm under the CON condition. Under the VEST and FAN conditions, the HR increased slowly during exercise and was 20 bpm lower than that under the CON condition at the end of exercise. The HR was significantly different among the three conditions at CP2–CP4 (Table 1).

### Estimated amount of sweating

The mean (± SD) estimated amount of sweating was 0.69 ± 0.45, 0.80 ± 0.28, and 1.09 ± 0.37 kg under the FAN, VEST, and CON conditions, respectively. The VEST and FAN conditions resulted in a tendency to reduce the estimated amount of sweating (*p* = 0.0968, Kruskal–Wallis test).

### Oxygen uptake

Oxygen uptake was similar among the experimental conditions.

### Subjective evaluations

**Figure 4:**
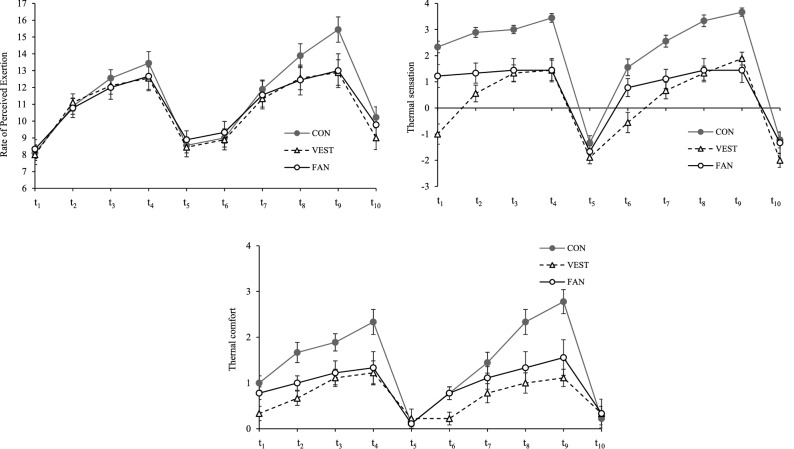
(**a**) Rate of perceived exertion in the CON, VEST, and FAN conditions. (**b**) Thermal sensation in the CON, VEST, and FAN conditions. (**c**) Thermal comfort in the CON, VEST, and FAN conditions. Data at every time points are presented as the mean ± standard error. *CON* participants did not wear any cooling garment, *VEST* participants wore a circulating-water cooling vest, *FAN* participants wore a fan-attached jacket. t_1_ and t_6_: immediately before starting exercise; t_2_–t_4_ and t_7_–t_9_: 10, 20, and 30 min, respectively, after starting exercise; t_5_ and t_10_: during rest. Number of participants: nine.

The RPE, thermal sensation, and thermal comfort values at the ten time points (t_1_–t_10_) are respectively presented in Fig. 4a–c. The RPE scores for the VEST and FAN conditions had similar trends and were lower than those for the CON condition throughout the exercise session. Under the CON condition, the RPE score was higher in the second exercise session than in the first session; however, under the VEST and FAN conditions, the RPE scores were nearly the same in the two sessions. The values of thermal sensation and thermal comfort were also lower under the VEST and FAN conditions than under the CON condition. Compared with their perceived comfort at the beginning of the experiment, the participants stated that they became even more comfortable when the ambient temperature changed from 40 to 28 °C.

## Discussion

To the best of our knowledge, this is the first study to confirm the suppression of the increase in the human body core temperature when ambient air is blown directly underneath work clothing at 40 °C. We also revealed the efficacy of a garment that circulates cool water. The body cooling effect observed in this study was equivalent to or even better than the effects achieved in previous studies that examined such cooling vests^23–25^. We believe that these body-cooling effects explain the favorable subjective evaluations provided by the participants in this study. This study has four main strengths. First, the participants underwent the exercise in an environment that was above body temperature. Second, we monitored the core temperature in multiple ways, which would be difficult to do in an actual workplace. Third, we investigated cooling workwear that is readily available in the market and popular in workplaces. Finally, we compared the efficacy of a fan-attached jacket with that of a circulating-water cooling vest. Compared with the control condition, we observed a 0.5 °C decrease in T_re_ and a 0.7 °C decrease in T_es_ under the FAN condition, and the T_re_ decrease was larger than that under the VEST condition. The amount of heat dissipation by evaporation exceeded the amount of heat convection from the hot ambient air, which was above body temperature. Additionally, these garments might ameliorate the effect of heat radiation from the environment. The observed suppression of the core temperature increase might help maintain body temperature homeostasis and relieve stress on the body while sweating.

Against our expectation, T_re_ was consistently higher under the VEST condition than under the CON condition during the first exercise session. This is probably because the evaporation of sweat from upper and middle trunk was interfered directly by the vest, while core body heat dissipated gradually to the skin surface. In addition, the cold water circulating throughout the vest might constrict the arteriovenous anastomosis in the subcutaneous region and thus inhibit heat dissipation from the skin surface through heat conduction and convection^26,27^. In contrast, T_re_ and T_es_ temporarily dropped immediately after the participants began exercise under the FAN and CON conditions—this might have been the “after-drop” phenomenon caused by vasodilation in the skin and increased blood return to the body from the cooled body surface^28–30^. The decreases in temperature were small and persisted for only a short period compared with the corresponding observations in our previous study. This is because T_sk-head_ was further lowered using a large fan in the previous study.

A temporary drop in T_es_ immediately after the beginning of the second exercise session was observed in four participants, probably because the esophageal sensor probe went into the stomach when they drank the beverage during the rest period. Indeed, we observed an increase in T_es_ when the probe was pulled up slightly.

It is reasonable to observe differences between the values of T_re_ and T_es_. This is because T_es_ changes rapidly, reflecting the temperature of circulating blood, whereas T_re_ changes more slowly, reflecting the temperature of visceral organs. We believe that the suppression of the peak temperatures of both T_re_ and T_es_ would contribute to preventing heat-related illness.

In a previous study, the fans of the fan-attached jacket decreased the skin temperatures of the back and abdomen by 1.0 °C compared with the control condition^11^. In this study, we did not measure the skin temperatures of body trunk because they must be directly influenced by the temperature of circulating water in VEST condition. The observed decrease in T_sk-head_ might be the reflection from a suppressed core temperature. Another previous study reported the faster recovery of the skin temperature, tympanic temperature, and HR after exercise when wearing a fan-attached jacket compared with not wearing the jacket^10^; however, we observed a much faster recovery of T_sk-head_, the core body temperature, and the HR not only after exercise but even during exercise. This is probably because we examined the cooling effect in a hotter environment and the airflow of the fan was set at a stronger level than in the previous study.

In a hot environment, we generally observe increases in the HR and sweat volume and a decrease in the stroke volume due to the increased proportion of blood circulating to the body surface to enhance heat dissipation. To maintain cardiac output when the stroke volume decreases, the HR will increase. The increase in the HR was suppressed under the VEST and FAN conditions because of the suppression of the core temperature increase. There was no difference in the HR between the VEST and FAN conditions because the elevations in core temperature were nearly the same.

There was no significant difference in the estimated sweat volume among the three conditions. However, the mean estimated sweat volume was highest under the CON condition, followed by the VEST and FAN conditions, likely owing to the suppression of the core temperature increase by the cooling garments. Moreover, the proportion of evaporated sweat was highest under the FAN condition, and sweat evaporation effectively lowered the body temperature.

We did not observe differences in oxygen uptake among the three conditions, which indicated that the environmental exposure and physical workload were uniform throughout the study.

The subjective evaluations generally reflected reduced heat stress under the VEST and FAN conditions. Nevertheless, under the FAN condition, the participants reported that they were slightly uncomfortable before the start of the first exercise session because the fan conveyed hot air under the clothing before the start of sweat secretion and evaporation. A few participants reported that they were uncomfortable under the VEST condition before the start of the first exercise session, likely because they felt cold at rest even in the hot environment.

Our study has limitations. All participants were Japanese male adults in their 20s and 30s. Therefore, the study may have limitations in terms of its interpretability to women, to the elderly generation, and to people from different nations. However, the uniformity of the sample was beneficial because physiological differences between individuals are less apparent among young people. Additionally, because the physical workload primarily targeted the lower extremities, and the artificial environment was almost windless, the conditions did not reflect an actual work situation. However, these conditions allowed for the standardization of the exposure protocol. We believe that the advantage of the FAN condition would hold even with additional wind flow to promote heat dissipation under the CON and VEST conditions because the airflow provided by the fan-attached jacket is directed under the clothing.

Our observation may not be applicable in the situation disadvantageous to evaporation particularly in more humid environment; however, the 50% of relative humidity is already higher than the usual meteorological data recorded at around 40 °C.

## Summary and conclusions

The fan-attached jacket that blows ambient air at T_a_ = 40 °C and RH of 50% directly under work clothing suppressed the core temperature increase better than the cooling vest with circulating water maintained at 10.0 °C. After two 30-min exercise sessions, T_re_ was respectively 0.5 °C and 0.3 °C lower under the FAN and VEST conditions than under the CON condition. When the garments were worn in T_a_ = 40 °C and RH of 50% environment, the heat dissipation through evaporation exceeded the heat convection from the hot ambient air. The result of this study broadens the choice of cooling garments that can effectively prevent heat-related illnesses during physically demanding work in extreme heat.

## Supplementary Information

Supplementary Information.
