# Supplementary material for: A fan-attached jacket worn in an environment exceeding body temperature suppresses an increase in core temperature
Source: Sci Rep. 2021 Oct 28;11:21269. doi: 10.1038/s41598-021-00655-2 (PMC8553827; doi:10.1038/s41598-021-00655-2)
Supplement: Supplementary file 1 — Supplementary Information. [file 41598_2021_655_MOESM1_ESM.pdf]

min	number	Tre	Tes	Tsk-head	HR	ID	condition	period	Ta	RH
0	1	36.72	36.58	35.52	82	1	CON	REST1	28	50
	2	36.72	36.58	35.51	81	1	CON	REST1	28	50
	3	36.72	36.59	35.50	81	1	CON	REST1	28	50
	4	36.73	36.59	35.49	89	1	CON	REST1	28	50
	5	36.73	36.58	35.50		1	CON	REST1	28	50
	6	36.73	36.59	35.53	80	1	CON	REST1	28	50
	7	36.73	36.60	35.55	90	1	CON	REST1	28	50
	8	36.72	36.59	35.54	77	1	CON	REST1	28	50
	9	36.73	36.59	35.54	78	1	CON	REST1	28	50
	10	36.73	36.60	35.58	87	1	CON	REST1	28	50
	11	36.72	36.59	35.63	76	1	CON	REST1	28	50
	12	36.72	36.60	35.67	76	1	CON	REST1	28	50
	13	36.74	36.63	35.68	76	1	CON	REST1	28	50
	14	36.74	36.61	35.67	82	1	CON	REST1	28	50
	15	36.72	36.61	35.66	75	1	CON	REST1	28	50
	16	36.72	36.61	35.66	81	1	CON	REST1	28	50
	17	36.73	36.59	35.66	77	1	CON	REST1	28	50
	18	36.72	36.60	35.63	78	1	CON	REST1	28	50
	19	36.71	36.59	35.58	86	1	CON	REST1	28	50
	20	36.70	36.58	35.55	77	1	CON	REST1	28	50
	21	36.71	36.60	35.52	75	1	CON	REST1	28	50
	22	36.72	36.59	35.50	74	1	CON	REST1	28	50
	23	36.73	36.59	35.49	72	1	CON	REST1	28	50
	24	36.73	36.61	35.47	72	1	CON	REST1	28	50
	25	36.71	36.59	35.45	72	1	CON	REST1	28	50
	26	36.71	36.59	35.45	73	1	CON	REST1	28	50
	27	36.71	36.60	35.45	73	1	CON	REST1	28	50
	28	36.70	36.60	35.45	71	1	CON	REST1	28	50
	29	36.71	36.60	35.45	72	1	CON	REST1	28	50
	30	36.72	36.59	35.45	73	1	CON	REST1	28	50
5	31	36.71	36.59	35.46	74	1	CON	REST1	28	50
	32	36.71	36.60	35.46	74	1	CON	REST1	28	50
	33	36.70	36.59	35.47	79	1	CON	REST1	28	50
	34	36.70	36.59	35.50	69	1	CON	REST1	28	50
	35	36.71	36.60	35.50	74	1	CON	REST1	28	50
	36	36.71	36.60	35.51	69	1	CON	REST1	28	50
	37	36.70	36.60	35.53	73	1	CON	REST1	28	50
	38	36.70	36.58	35.53	71	1	CON	REST1	28	50
	39	36.71	36.58	35.53	69	1	CON	REST1	28	50
	40	36.70	36.58	35.55	71	1	CON	REST1	28	50
	41	36.70	36.57	35.57	70	1	CON	REST1	28	50
	42	36.70	36.56	35.58	71	1	CON	REST1	28	50
	43	36.71	36.56	35.58	69	1	CON	REST1	28	50
	44	36.71	36.58	35.56	72	1	CON	REST1	28	50
	45	36.71	36.60	35.54	73	1	CON	REST1	28	50
	46	36.71	36.60	35.53	75	1	CON	REST1	28	50

min	number	Tre	Tes	Tsk-head	HR	ID	condition	period	Ta	RH
	47	36.72	36.59	35.53	74	1	CON	REST1	28	50
	48	36.71	36.57	35.52	73	1	CON	REST1	28	50
	49	36.70	36.56	35.49	77	1	CON	REST1	28	50
	50	36.70	36.58	35.48	76	1	CON	REST1	28	50
	51	36.70	36.59	35.48	74	1	CON	REST1	28	50
	52	36.71	36.60	35.49	76	1	CON	REST1	28	50
	53	36.72	36.60	35.50	83	1	CON	REST1	28	50
	54	36.71	36.59	35.50	92	1	CON	REST1	28	50
	55	36.71	36.57	35.51	77	1	CON	REST1	28	50
	56	36.71	36.56	35.52	76	1	CON	REST1	28	50
	57	36.71	36.56	35.52	77	1	CON	REST1	28	50
	58	36.72	36.57	35.51	76	1	CON	REST1	28	50
	59	36.72	36.58	35.50	74	1	CON	REST1	28	50
	60	36.72	36.56	35.50	75	1	CON	REST1	28	50
10	61	36.73	36.58	35.51	72	1	CON	REST1	28	50
	62	36.72	36.59	35.52	76	1	CON	REST1	28	50
	63	36.71	36.60	35.52	73	1	CON	REST1	28	50
	64	36.71	36.62	35.53	67	1	CON	REST1	28	50
	65	36.71	36.59	35.54	75	1	CON	REST1	28	50
	66	36.71	36.56	35.54	71	1	CON	REST1	28	50
	67	36.70	36.58	35.54	70	1	CON	REST1	28	50
	68	36.70	36.59	35.54	70	1	CON	REST1	28	50
	69	36.70	36.59	35.53	70	1	CON	REST1	28	50
	70	36.70	36.60	35.54	71	1	CON	REST1	28	50
	71	36.70	36.61	35.55	78	1	CON	REST1	28	50
	72	36.71	36.61	35.57	70	1	CON	REST1	28	50
	73	36.70	36.61	35.57	70	1	CON	REST1	28	50
	74	36.70	36.60	35.58	68	1	CON	REST1	28	50
	75	36.70	36.59	35.60	68	1	CON	REST1	28	50
	76	36.70	36.59	35.59	71	1	CON	REST1	28	50
	77	36.70	36.59	35.59	76	1	CON	REST1	28	50
	78	36.71	36.58	35.59	84	1	CON	REST1	28	50
	79	36.71	36.59	35.60	77	1	CON	REST1	28	50
	80	36.72	36.60	35.60	72	1	CON	REST1	28	50
	81	36.72	36.62	35.60	73	1	CON	REST1	28	50
	82	36.72	36.65	35.61	74	1	CON	REST1	28	50
	83	36.72	36.66	35.64	74	1	CON	REST1	28	50
	84	36.72	36.66	35.65	75	1	CON	REST1	28	50
	85	36.72	36.66	35.64	73	1	CON	REST1	28	50
	86	36.72	36.64	35.65	71	1	CON	REST1	28	50
	87	36.72	36.64	35.64	71	1	CON	REST1	28	50
	88	36.71	36.65	35.63	83	1	CON	REST1	28	50
	89	36.71	36.67	35.64	84	1	CON	REST1	28	50
	90	36.71	36.66	35.64	76	1	CON	REST1	28	50
15	91	36.71	36.65	35.62	73	1	CON	REST1	28	50
	92	36.71	36.65	35.62	78	1	CON	REST1	28	50

min	number	Tre	Tes	Tsk-head	HR	ID	condition	period	Ta	RH
	93	36.71	36.65	35.62	67	1	CON	REST1	28	50
	94	36.72	36.67	35.61	66	1	CON	REST1	28	50
	95	36.73	36.68	35.60	64	1	CON	REST1	28	50
	96	36.73	36.68	35.61	67	1	CON	REST1	28	50
	97	36.73	36.69	35.60	65	1	CON	REST1	28	50
	98	36.73	36.65	35.60	73	1	CON	REST1	28	50
	99	36.71	36.63	35.60	90	1	CON	REST1	28	50
	100	36.70	36.63	35.61	89	1	CON	REST1	28	50
	101	36.69	36.66	35.61	86	1	CON	REST1	28	50
	102	36.69	36.67	35.61	88	1	CON	REST1	28	50
	103	36.70	36.67	35.61	85	1	CON	REST1	40	50
	104	36.70	36.67	35.72	98	1	CON	REST1	40	50
	105	36.71	36.65	35.90	78	1	CON	REST1	40	50
	106	36.72	36.66	36.02	77	1	CON	REST1	40	50
	107	36.73	36.67	36.10	77	1	CON	REST1	40	50
	108	36.74	36.67	36.17	78	1	CON	REST1	40	50
	109	36.74	36.68	36.23	75	1	CON	REST1	40	50
	110	36.74	36.68	36.29	74	1	CON	REST1	40	50
	111	36.74	36.67	36.33	73	1	CON	REST1	40	50
	112	36.74	36.68	36.36	68	1	CON	REST1	40	50
	113	36.74	36.67	36.39	74	1	CON	REST1	40	50
	114	36.73	36.67	36.40	72	1	CON	REST1	40	50
	115	36.73	36.69	36.40	74	1	CON	REST1	40	50
	116	36.73	36.69	36.41	79	1	CON	REST1	40	50
	117	36.74	36.69	36.42	80	1	CON	REST1	40	50
	118	36.74	36.69	36.44	73	1	CON	REST1	40	50
	119	36.74	36.69	36.45	76	1	CON	REST1	40	50
	120	36.75	36.70	36.47	80	1	CON	REST1	40	50
20	121	36.74	36.70	36.49	81	1	CON	REST1	40	50
	122	36.74	36.70	36.49	82	1	CON	REST1	40	50
	123	36.73	36.69	36.49	82	1	CON	REST1	40	50
	124	36.73	36.69	36.51	76	1	CON	REST1	40	50
	125	36.74	36.69	36.52	80	1	CON	REST1	40	50
	126	36.74	36.68	36.52	78	1	CON	REST1	40	50
	127	36.74	36.68	36.53	73	1	CON	REST1	40	50
	128	36.74	36.67	36.52	77	1	CON	REST1	40	50
	129	36.74	36.66	36.53	83	1	CON	REST1	40	50
	130	36.73	36.66	36.53	77	1	CON	REST1	40	50
	131	36.73	36.67	36.53	73	1	CON	REST1	40	50
	132	36.72	36.67	36.54	78	1	CON	REST1	40	50
	133	36.71	36.65	36.53	80	1	CON	REST1	40	50
	134	36.71	36.64	36.52	96	1	CON	REST1	40	50
	135	36.71	36.64	36.51	96	1	CON	REST1	40	50
	136	36.70	36.64	36.51	75	1	CON	REST1	40	50
	137	36.69	36.63	36.52	76	1	CON	REST1	40	50
	138	36.69	36.62	36.52	84	1	CON	REST1	40	50

min	number	Tre	Tes	Tsk-head	HR	ID	condition	period	Ta	RH
	139	36.69	36.62	36.54	82	1	CON	EXERCISE1	40	50
	140	36.69	36.61	36.55	84	1	CON	EXERCISE1	40	50
	141	36.68	36.60	36.53	90	1	CON	EXERCISE1	40	50
	142	36.67	36.59	36.51	98	1	CON	EXERCISE1	40	50
	143	36.66	36.59	36.50	99	1	CON	EXERCISE1	40	50
	144	36.65	36.59	36.50	98	1	CON	EXERCISE1	40	50
	145	36.64	36.58	36.49	103	1	CON	EXERCISE1	40	50
	146	36.64	36.58	36.47	105	1	CON	EXERCISE1	40	50
	147	36.63	36.57	36.47	105	1	CON	EXERCISE1	40	50
	148	36.63	36.57	36.48	108	1	CON	EXERCISE1	40	50
	149	36.64	36.58	36.48	108	1	CON	EXERCISE1	40	50
	150	36.64	36.58	36.48	111	1	CON	EXERCISE1	40	50
25	151	36.64	36.56	36.47	111	1	CON	EXERCISE1	40	50
	152	36.64	36.55	36.47	110	1	CON	EXERCISE1	40	50
	153	36.64	36.56	36.47	110	1	CON	EXERCISE1	40	50
	154	36.64	36.57	36.48	111	1	CON	EXERCISE1	40	50
	155	36.65	36.57	36.49	110	1	CON	EXERCISE1	40	50
	156	36.65	36.58	36.49	112	1	CON	EXERCISE1	40	50
	157	36.65	36.59	36.51	112	1	CON	EXERCISE1	40	50
	158	36.65	36.60	36.51	113	1	CON	EXERCISE1	40	50
	159	36.65	36.61	36.53	110	1	CON	EXERCISE1	40	50
	160	36.66	36.62	36.54	110	1	CON	EXERCISE1	40	50
	161	36.66	36.63	36.54	111	1	CON	EXERCISE1	40	50
	162	36.65	36.64	36.54	112	1	CON	EXERCISE1	40	50
	163	36.66	36.64	36.54	110	1	CON	EXERCISE1	40	50
	164	36.66	36.66	36.55	110	1	CON	EXERCISE1	40	50
	165	36.66	36.67	36.56	115	1	CON	EXERCISE1	40	50
	166	36.67	36.68	36.58	116	1	CON	EXERCISE1	40	50
	167	36.68	36.70	36.61	115	1	CON	EXERCISE1	40	50
	168	36.69	36.71	36.63	118	1	CON	EXERCISE1	40	50
	169	36.69	36.72	36.65	118	1	CON	EXERCISE1	40	50
	170	36.69	36.74	36.67	116	1	CON	EXERCISE1	40	50
	171	36.69	36.76	36.68	116	1	CON	EXERCISE1	40	50
	172	36.69	36.76	36.66	116	1	CON	EXERCISE1	40	50
	173	36.69	36.78	36.66	116	1	CON	EXERCISE1	40	50
	174	36.70	36.78	36.67	114	1	CON	EXERCISE1	40	50
	175	36.71	36.79	36.69	115	1	CON	EXERCISE1	40	50
	176	36.72	36.81	36.70	115	1	CON	EXERCISE1	40	50
	177	36.72	36.81	36.69	113	1	CON	EXERCISE1	40	50
	178	36.71	36.82	36.70	113	1	CON	EXERCISE1	40	50
	179	36.71	36.83	36.70	116	1	CON	EXERCISE1	40	50
	180	36.72	36.84	36.71	118	1	CON	EXERCISE1	40	50
30	181	36.73	36.87	36.74	118	1	CON	EXERCISE1	40	50
	182	36.74	36.87	36.74	119	1	CON	EXERCISE1	40	50
	183	36.74	36.88	36.74	119	1	CON	EXERCISE1	40	50
	184	36.74	36.89	36.74	120	1	CON	EXERCISE1	40	50

min	number	Tre	Tes	Tsk-head	HR	ID	condition	period	Ta	RH
	185	36.73	36.89	36.73	121	1	CON	EXERCISE1	40	50
	186	36.75	36.90	36.74	119	1	CON	EXERCISE1	40	50
	187	36.76	36.90	36.76	118	1	CON	EXERCISE1	40	50
	188	36.76	36.92	36.79	118	1	CON	EXERCISE1	40	50
	189	36.75	36.92	36.79	121	1	CON	EXERCISE1	40	50
	190	36.76	36.92	36.79	118	1	CON	EXERCISE1	40	50
	191	36.76	36.93	36.80	120	1	CON	EXERCISE1	40	50
	192	36.77	36.94	36.83	121	1	CON	EXERCISE1	40	50
	193	36.78	36.95	36.83	125	1	CON	EXERCISE1	40	50
	194	36.78	36.96	36.84	124	1	CON	EXERCISE1	40	50
	195	36.78	36.97	36.86	119	1	CON	EXERCISE1	40	50
	196	36.78	36.97	36.86	121	1	CON	EXERCISE1	40	50
	197	36.78	36.96	36.86	121	1	CON	EXERCISE1	40	50
	198	36.78	36.97	36.87	115	1	CON	EXERCISE1	40	50
	199	36.79	36.98	36.89	114	1	CON	EXERCISE1	40	50
	200	36.79	36.98	36.88	121	1	CON	EXERCISE1	40	50
	201	36.79	36.99	36.87	121	1	CON	EXERCISE1	40	50
	202	36.80	37.01	36.88	119	1	CON	EXERCISE1	40	50
	203	36.79	37.02	36.88	117	1	CON	EXERCISE1	40	50
	204	36.80	37.02	36.89	117	1	CON	EXERCISE1	40	50
	205	36.80	37.03	36.90	114	1	CON	EXERCISE1	40	50
	206	36.80	37.04	36.89	119	1	CON	EXERCISE1	40	50
	207	36.81	37.04	36.88	119	1	CON	EXERCISE1	40	50
	208	36.82	37.04	36.89	122	1	CON	EXERCISE1	40	50
	209	36.83	37.06	36.91	123	1	CON	EXERCISE1	40	50
	210	36.83	36.92	36.92	124	1	CON	EXERCISE1	40	50
35	211	36.83	36.89	36.93	118	1	CON	EXERCISE1	40	50
	212	36.83	37.02	36.95	118	1	CON	EXERCISE1	40	50
	213	36.83	37.04	36.97	118	1	CON	EXERCISE1	40	50
	214	36.84	37.05	36.98	117	1	CON	EXERCISE1	40	50
	215	36.84	37.07	36.97	117	1	CON	EXERCISE1	40	50
	216	36.84	37.08	36.98	119	1	CON	EXERCISE1	40	50
	217	36.85	37.08	36.98	118	1	CON	EXERCISE1	40	50
	218	36.86	37.08	36.97	118	1	CON	EXERCISE1	40	50
	219	36.87	37.09	36.97	122	1	CON	EXERCISE1	40	50
	220	36.87	37.10	36.97	122	1	CON	EXERCISE1	40	50
	221	36.88	37.11	36.97	119	1	CON	EXERCISE1	40	50
	222	36.88	37.12	36.96	121	1	CON	EXERCISE1	40	50
	223	36.88	37.12	36.97	120	1	CON	EXERCISE1	40	50
	224	36.88	37.13	36.97	119	1	CON	EXERCISE1	40	50
	225	36.89	37.14	36.98	122	1	CON	EXERCISE1	40	50
	226	36.90	37.14	36.98	122	1	CON	EXERCISE1	40	50
	227	36.89	37.14	36.98	122	1	CON	EXERCISE1	40	50
	228	36.89	37.15	36.99	121	1	CON	EXERCISE1	40	50
	229	36.90	37.16	37.00	122	1	CON	EXERCISE1	40	50
	230	36.91	37.17	36.99	124	1	CON	EXERCISE1	40	50

min	number	Tre	Tes	Tsk-head	HR	ID	condition	period	Ta	RH
	231	36.91	37.17	37.00	124	1	CON	EXERCISE1	40	50
	232	36.92	37.19	37.01	125	1	CON	EXERCISE1	40	50
	233	36.92	37.20	37.02	124	1	CON	EXERCISE1	40	50
	234	36.92	37.19	37.02	124	1	CON	EXERCISE1	40	50
	235	36.92	37.20	37.02	125	1	CON	EXERCISE1	40	50
	236	36.93	37.21	37.02	126	1	CON	EXERCISE1	40	50
	237	36.93	37.21	37.02	127	1	CON	EXERCISE1	40	50
	238	36.94	37.21	37.02	128	1	CON	EXERCISE1	40	50
	239	36.95	37.23	37.03	128	1	CON	EXERCISE1	40	50
	240	36.95	37.24	37.04	128	1	CON	EXERCISE1	40	50
40	241	36.95	37.23	37.03	128	1	CON	EXERCISE1	40	50
	242	36.96	37.24	37.02	128	1	CON	EXERCISE1	40	50
	243	36.97	37.25	37.04	126	1	CON	EXERCISE1	40	50
	244	36.97	37.26	37.05	127	1	CON	EXERCISE1	40	50
	245	36.98	37.27	37.05	127	1	CON	EXERCISE1	40	50
	246	36.99	37.28	37.06	128	1	CON	EXERCISE1	40	50
	247	36.99	37.28	37.05	128	1	CON	EXERCISE1	40	50
	248	36.98	37.27	37.04	128	1	CON	EXERCISE1	40	50
	249	36.98	37.28	37.03	127	1	CON	EXERCISE1	40	50
	250	36.99	37.29	37.02	128	1	CON	EXERCISE1	40	50
	251	37.00	37.30	37.03	128	1	CON	EXERCISE1	40	50
	252	37.00	37.30	37.03	128	1	CON	EXERCISE1	40	50
	253	37.00	37.30	37.03	129	1	CON	EXERCISE1	40	50
	254	37.01	37.31	37.04	130	1	CON	EXERCISE1	40	50
	255	37.01	37.32	37.05	129	1	CON	EXERCISE1	40	50
	256	37.02	37.32	37.06	126	1	CON	EXERCISE1	40	50
	257	37.02	37.33	37.06	127	1	CON	EXERCISE1	40	50
	258	37.02	37.33	37.05	129	1	CON	EXERCISE1	40	50
	259	37.03	37.31	37.05	129	1	CON	EXERCISE1	40	50
	260	37.02	37.30	37.05	128	1	CON	EXERCISE1	40	50
	261	37.02	37.32	37.05	130	1	CON	EXERCISE1	40	50
	262	37.04	37.34	37.07	132	1	CON	EXERCISE1	40	50
	263	37.05	37.35	37.09	130	1	CON	EXERCISE1	40	50
	264	37.05	37.35	37.09	129	1	CON	EXERCISE1	40	50
	265	37.06	37.37	37.11	128	1	CON	EXERCISE1	40	50
	266	37.06	37.39	37.11	128	1	CON	EXERCISE1	40	50
	267	37.05	37.39	37.10	130	1	CON	EXERCISE1	40	50
	268	37.05	37.38	37.09	129	1	CON	EXERCISE1	40	50
	269	37.06	37.39	37.09	130	1	CON	EXERCISE1	40	50
	270	37.07	37.40	37.10	133	1	CON	EXERCISE1	40	50
45	271	37.06	37.40	37.10	133	1	CON	EXERCISE1	40	50
	272	37.07	37.41	37.10	131	1	CON	EXERCISE1	40	50
	273	37.07	37.41	37.11	130	1	CON	EXERCISE1	40	50
	274	37.07	37.41	37.12	133	1	CON	EXERCISE1	40	50
	275	37.08	37.42	37.11	133	1	CON	EXERCISE1	40	50
	276	37.09	37.42	37.10	133	1	CON	EXERCISE1	40	50

min	number	Tre	Tes	Tsk-head	HR	ID	condition	period	Ta	RH
	277	37.09	37.43	37.10	132	1	CON	EXERCISE1	40	50
	278	37.10	37.43	37.11	131	1	CON	EXERCISE1	40	50
	279	37.11	37.44	37.12	131	1	CON	EXERCISE1	40	50
	280	37.11	37.45	37.12	133	1	CON	EXERCISE1	40	50
	281	37.12	37.45	37.13	134	1	CON	EXERCISE1	40	50
	282	37.12	37.46	37.14	133	1	CON	EXERCISE1	40	50
	283	37.13	37.46	37.14	133	1	CON	EXERCISE1	40	50
	284	37.13	37.47	37.14	134	1	CON	EXERCISE1	40	50
	285	37.14	37.48	37.13	134	1	CON	EXERCISE1	40	50
	286	37.14	37.48	37.15	135	1	CON	EXERCISE1	40	50
	287	37.14	37.49	37.15	135	1	CON	EXERCISE1	40	50
	288	37.14	37.49	37.15	135	1	CON	EXERCISE1	40	50
	289	37.14	37.49	37.16	133	1	CON	EXERCISE1	40	50
	290	37.14	37.50	37.17	134	1	CON	EXERCISE1	40	50
	291	37.14	37.50	37.18	134	1	CON	EXERCISE1	40	50
	292	37.16	37.51	37.18	135	1	CON	EXERCISE1	40	50
	293	37.17	37.54	37.17	136	1	CON	EXERCISE1	40	50
	294	37.18	37.55	37.16	134	1	CON	EXERCISE1	40	50
	295	37.18	37.55	37.10	133	1	CON	EXERCISE1	40	50
	296	37.18	37.55	37.04	132	1	CON	EXERCISE1	40	50
	297	37.19	37.55	37.05	130	1	CON	EXERCISE1	40	50
	298	37.19	37.55	37.06	131	1	CON	EXERCISE1	40	50
	299	37.19	37.55	37.06	132	1	CON	EXERCISE1	40	50
	300	37.19	37.50	37.05	133	1	CON	EXERCISE1	40	50
50	301	37.19	37.48	37.05	134	1	CON	EXERCISE1	40	50
	302	37.19	37.52	37.06	133	1	CON	EXERCISE1	40	50
	303	37.20	37.53	37.06	133	1	CON	EXERCISE1	40	50
	304	37.20	37.54	37.08	136	1	CON	EXERCISE1	40	50
	305	37.21	37.55	37.09	136	1	CON	EXERCISE1	40	50
	306	37.22	37.55	37.10	136	1	CON	EXERCISE1	40	50
	307	37.22	37.56	37.10	137	1	CON	EXERCISE1	40	50
	308	37.23	37.57	37.11	136	1	CON	EXERCISE1	40	50
	309	37.23	37.58	37.12	135	1	CON	EXERCISE1	40	50
	310	37.24	37.59	37.13	136	1	CON	EXERCISE1	40	50
	311	37.25	37.60	37.14	139	1	CON	EXERCISE1	40	50
	312	37.25	37.60	37.15	139	1	CON	EXERCISE1	40	50
	313	37.26	37.60	37.18	138	1	CON	EXERCISE1	40	50
	314	37.27	37.61	37.20	138	1	CON	EXERCISE1	40	50
	315	37.27	37.62	37.20	138	1	CON	EXERCISE1	40	50
	316	37.27	37.62	37.20	138	1	CON	EXERCISE1	40	50
	317	37.27	37.62	37.19	137	1	CON	EXERCISE1	40	50
	318	37.27	37.64	37.20	140	1	CON	EXERCISE1	40	50
	319	37.26	37.65	37.18	140	1	CON	EXERCISE1	40	50
	320	37.27	37.67	37.18	136	1	CON	REST2	28	50
	321	37.28	37.69	36.98	136	1	CON	REST2	28	50
	322	37.27	37.65	36.73	123	1	CON	REST2	28	50

min	number	Tre	Tes	Tsk-head	HR	ID	condition	period	Ta	RH
	323	37.26	37.66	36.62	123	1	CON	REST2	28	50
	324	37.26	37.62	36.57	125	1	CON	REST2	28	50
	325	37.27	37.59	36.55	115	1	CON	REST2	28	50
	326	37.27	37.63	36.52	116	1	CON	REST2	28	50
	327	37.27	37.63	36.44	119	1	CON	REST2	28	50
	328	37.28	37.63	36.32	121	1	CON	REST2	28	50
	329	37.29	37.63	36.19	120	1	CON	REST2	28	50
	330	37.29	37.63	36.10	118	1	CON	REST2	28	50
55	331	37.30	37.64	36.08	120	1	CON	REST2	28	50
	332	37.30	37.68	36.04	114	1	CON	REST2	28	50
	333	37.30	37.67	36.02	120	1	CON	REST2	28	50
	334	37.30	37.62	36.02	96	1	CON	REST2	28	50
	335	37.30	37.61	36.06	102	1	CON	REST2	28	50
	336	37.30	37.62	36.10	113	1	CON	REST2	28	50
	337	37.29	37.58	36.04	105	1	CON	REST2	28	50
	338	37.29	37.53	36.02	108	1	CON	REST2	28	50
	339	37.29	33.16	36.01	116	1	CON	REST2	28	50
	340	37.29	30.69	35.96	110	1	CON	REST2	28	50
	341	37.30	31.66	35.95	116	1	CON	REST2	28	50
	342	37.30	31.33	35.89	111	1	CON	REST2	28	50
	343	37.30	32.30	35.76	105	1	CON	REST2	28	50
	344	37.30	32.90	35.74	110	1	CON	REST2	28	50
	345	37.29	30.22	35.84	118	1	CON	REST2	28	50
	346	37.29	29.53	35.86	100	1	CON	REST2	28	50
	347	37.29	32.12	35.85	105	1	CON	REST2	28	50
	348	37.29	32.67	35.85	103	1	CON	REST2	28	50
	349	37.28	33.05	35.83	106	1	CON	REST2	28	50
	350	37.28	33.43	35.83	103	1	CON	REST2	28	50
	351	37.28	33.85	35.78	107	1	CON	REST2	28	50
	352	37.28	34.26	35.67	105	1	CON	REST2	28	50
	353	37.28	30.80	35.65	112	1	CON	REST2	28	50
	354	37.28	28.21	35.69	110	1	CON	REST2	28	50
	355	37.29	30.27	35.67	111	1	CON	REST2	28	50
	356	37.28	31.53	35.61	107	1	CON	REST2	28	50
	357	37.28	32.18	35.57	100	1	CON	REST2	28	50
	358	37.27	33.07	35.52	103	1	CON	REST2	28	50
	359	37.27	33.65	35.44	93	1	CON	REST2	28	50
	360	37.27	33.78	35.40	99	1	CON	REST2	28	50
60	361	37.27	33.98	35.35	99	1	CON	REST2	28	50
	362	37.27	34.25	35.31	100	1	CON	REST2	28	50
	363	37.27	31.59	35.33	108	1	CON	REST2	28	50
	364	37.27	29.69	35.38	114	1	CON	REST2	28	50
	365	37.27	31.20	35.39	94	1	CON	REST2	28	50
	366	37.26	32.47	35.36	103	1	CON	REST2	28	50
	367	37.25	33.26	35.36	98	1	CON	REST2	28	50
	368	37.26	33.53	35.33	100	1	CON	REST2	28	50

min	number	Tre	Tes	Tsk-head	HR	ID	condition	period	Ta	RH
	369	37.25	33.83	35.29	100	1	CON	REST2	28	50
	370	37.25	34.10	35.27	101	1	CON	REST2	28	50
	371	37.26	34.35	35.21	95	1	CON	REST2	28	50
	372	37.27	34.56	35.15	96	1	CON	REST2	28	50
	373	37.27	34.76	35.13	98	1	CON	REST2	28	50
	374	37.27	33.78	35.15	104	1	CON	REST2	28	50
	375	37.27	32.52	35.21	105	1	CON	REST2	28	50
	376	37.29	32.79	35.25	96	1	CON	REST2	28	50
	377	37.30	31.30	35.26	108	1	CON	REST2	28	50
	378	37.30	28.48	35.28	104	1	CON	REST2	28	50
	379	37.30	29.68	35.29	106	1	CON	REST2	28	50
	380	37.30	29.23	35.27	107	1	CON	REST2	28	50
	381	37.29	26.97	35.27	107	1	CON	REST2	28	50
	382	37.29	27.20	35.28	103	1	CON	REST2	28	50
	383	37.29	27.98	35.27	107	1	CON	REST2	28	50
	384	37.29	27.55	35.26	106	1	CON	REST2	28	50
	385	37.28	27.87	35.23	97	1	CON	REST2	28	50
	386	37.28	30.00	35.19	93	1	CON	REST2	28	50
	387	37.28	28.59	35.19	101	1	CON	REST2	28	50
	388	37.28	28.37	35.21	105	1	CON	REST2	28	50
	389	37.28	30.35	35.23	92	1	CON	REST2	28	50
	390	37.28	30.82	35.23	94	1	CON	REST2	28	50
65	391	37.28	31.59	35.21	102	1	CON	REST2	28	50
	392	37.27	32.33	35.16	92	1	CON	REST2	28	50
	393	37.28	32.79	35.14	96	1	CON	REST2	28	50
	394	37.28	33.20	35.15	97	1	CON	REST2	28	50
	395	37.28	33.52	35.14	94	1	CON	REST2	28	50
	396	37.27	33.81	35.13	94	1	CON	REST2	28	50
	397	37.26	34.05	35.14	94	1	CON	REST2	28	50
	398	37.25	34.28	35.11	94	1	CON	REST2	28	50
	399	37.26	34.52	35.08	100	1	CON	REST2	28	50
	400	37.26	34.88	35.05	101	1	CON	REST2	28	50
	401	37.25	35.09	35.05	92	1	CON	REST2	28	50
	402	37.25	35.13	35.07	94	1	CON	REST2	28	50
	403	37.24	35.26	35.06	96	1	CON	REST2	28	50
	404	37.22	35.38	35.05	95	1	CON	REST2	28	50
	405	37.22	35.49	35.00	91	1	CON	REST2	28	50
	406	37.23	35.60	34.97	92	1	CON	REST2	28	50
	407	37.22	35.71	34.99	94	1	CON	REST2	28	50
	408	37.22	35.81	35.00	93	1	CON	REST2	28	50
	409	37.22	35.89	35.00	92	1	CON	REST2	28	50
	410	37.22	35.97	35.00	93	1	CON	REST2	28	50
	411	37.22	36.06	34.98	93	1	CON	REST2	28	50
	412	37.22	36.12	34.94	94	1	CON	REST2	28	50
	413	37.22	36.17	34.92	95	1	CON	REST2	28	50
	414	37.23	36.23	34.90	94	1	CON	REST2	28	50

min	number	Tre	Tes	Tsk-head	HR	ID	condition	period	Ta	RH
	415	37.22	36.29	34.88	94	1	CON	REST2	28	50
	416	37.22	36.33	34.85	96	1	CON	REST2	28	50
	417	37.22	36.37	34.80	97	1	CON	REST2	28	50
	418	37.21	36.41	34.80	95	1	CON	REST2	28	50
	419	37.21	36.44	34.81	94	1	CON	REST2	28	50
	420	37.22	36.50	34.84	93	1	CON	REST2	28	50
70	421	37.21	36.54	34.89	103	1	CON	REST2	28	50
	422	37.20	36.53	34.94	105	1	CON	REST2	28	50
	423	37.20	36.54	34.97	86	1	CON	REST2	28	50
	424	37.21	36.55	35.04	92	1	CON	REST2	28	50
	425	37.21	36.55	35.09	77	1	CON	REST2	28	50
	426	37.21	36.57	35.12	86	1	CON	REST2	28	50
	427	37.21	36.62	35.21	88	1	CON	REST2	28	50
	428	37.20	36.65	35.29	88	1	CON	REST2	28	50
	429	37.20	36.66	35.39	85	1	CON	REST2	28	50
	430	37.20	36.65	35.49	83	1	CON	REST2	28	50
	431	37.20	36.66	35.52	84	1	CON	REST2	28	50
	432	37.20	36.67	35.55	85	1	CON	REST2	28	50
	433	37.19	36.66	35.55	89	1	CON	REST2	28	50
	434	37.19	36.67	35.53	88	1	CON	REST2	28	50
	435	37.19	36.68	35.53	91	1	CON	REST2	28	50
	436	37.18	36.72	35.46	101	1	CON	REST2	28	50
	437	37.17	36.75	35.42	91	1	CON	REST2	28	50
	438	37.17	36.01	35.45	91	1	CON	REST2	28	50
	439	37.18	35.79	35.46	92	1	CON	REST2	40	50
	440	37.18	36.42	35.44	98	1	CON	REST2	40	50
	441	37.18	36.57	35.50	93	1	CON	REST2	40	50
	442	37.18	36.64	35.63	99	1	CON	REST2	40	50
	443	37.18	36.65	35.76	91	1	CON	REST2	40	50
	444	37.18	36.66	35.88	90	1	CON	REST2	40	50
	445	37.19	36.70	36.04	83	1	CON	REST2	40	50
	446	37.20	36.72	36.18	82	1	CON	REST2	40	50
	447	37.20	36.72	36.23	84	1	CON	REST2	40	50
	448	37.20	36.72	36.28	84	1	CON	REST2	40	50
	449	37.20	36.80	36.37	84	1	CON	REST2	40	50
	450	37.19	36.85	36.43	88	1	CON	REST2	40	50
75	451	37.19	36.82	36.43	87	1	CON	REST2	40	50
	452	37.19	36.82	36.47	91	1	CON	REST2	40	50
	453	37.19	36.82	36.48	84	1	CON	REST2	40	50
	454	37.19	36.82	36.50	87	1	CON	REST2	40	50
	455	37.20	36.82	36.52	88	1	CON	REST2	40	50
	456	37.20	36.83	36.53	88	1	CON	REST2	40	50
	457	37.19	36.82	36.53	89	1	CON	REST2	40	50
	458	37.18	36.80	36.45	105	1	CON	REST2	40	50
	459	37.17	36.80	36.40	104	1	CON	REST2	40	50
	460	37.16	36.80	36.43	98	1	CON	REST2	40	50

min	number	Tre	Tes	Tsk-head	HR	ID	condition	period	Ta	RH
	461	37.15	36.81	36.47	101	1	CON	REST2	40	50
	462	37.16	36.82	36.50	101	1	CON	REST2	40	50
	463	37.15	36.83	36.53	98	1	CON	EXERCISE2	40	50
	464	37.15	36.83	36.56	95	1	CON	EXERCISE2	40	50
	465	37.15	36.19	36.55	108	1	CON	EXERCISE2	40	50
	466	37.15	36.08	36.52	115	1	CON	EXERCISE2	40	50
	467	37.15	36.66	36.50	115	1	CON	EXERCISE2	40	50
	468	37.14	36.75	36.49	115	1	CON	EXERCISE2	40	50
	469	37.13	36.78	36.51	118	1	CON	EXERCISE2	40	50
	470	37.13	36.79	36.53	120	1	CON	EXERCISE2	40	50
	471	37.13	36.80	36.53	121	1	CON	EXERCISE2	40	50
	472	37.14	36.80	36.55	120	1	CON	EXERCISE2	40	50
	473	37.15	36.82	36.56	121	1	CON	EXERCISE2	40	50
	474	37.14	36.85	36.58	120	1	CON	EXERCISE2	40	50
	475	37.13	36.87	36.57	121	1	CON	EXERCISE2	40	50
	476	37.13	36.86	36.56	120	1	CON	EXERCISE2	40	50
	477	37.14	36.86	36.56	122	1	CON	EXERCISE2	40	50
	478	37.14	36.87	36.55	121	1	CON	EXERCISE2	40	50
	479	37.14	36.89	36.57	122	1	CON	EXERCISE2	40	50
	480	37.14	36.92	36.59	122	1	CON	EXERCISE2	40	50
80	481	37.13	36.93	36.57	121	1	CON	EXERCISE2	40	50
	482	37.13	36.93	36.55	121	1	CON	EXERCISE2	40	50
	483	37.13	36.94	36.56	123	1	CON	EXERCISE2	40	50
	484	37.14	36.95	36.58	125	1	CON	EXERCISE2	40	50
	485	37.14	36.97	36.61	126	1	CON	EXERCISE2	40	50
	486	37.14	36.98	36.63	128	1	CON	EXERCISE2	40	50
	487	37.14	37.00	36.60	126	1	CON	EXERCISE2	40	50
	488	37.15	37.01	36.55	125	1	CON	EXERCISE2	40	50
	489	37.15	36.71	36.51	127	1	CON	EXERCISE2	40	50
	490	37.15	36.47	36.50	124	1	CON	EXERCISE2	40	50
	491	37.15	36.72	36.48	125	1	CON	EXERCISE2	40	50
	492	37.15	36.91	36.47	125	1	CON	EXERCISE2	40	50
	493	37.16	36.95	36.46	127	1	CON	EXERCISE2	40	50
	494	37.16	37.00	36.46	128	1	CON	EXERCISE2	40	50
	495	37.16	37.02	36.46	129	1	CON	EXERCISE2	40	50
	496	37.17	37.03	36.48	128	1	CON	EXERCISE2	40	50
	497	37.17	37.03	36.49	131	1	CON	EXERCISE2	40	50
	498	37.17	37.02	36.48	132	1	CON	EXERCISE2	40	50
	499	37.18	37.05	36.50	128	1	CON	EXERCISE2	40	50
	500	37.19	37.06	36.51	129	1	CON	EXERCISE2	40	50
	501	37.19	37.06	36.52	129	1	CON	EXERCISE2	40	50
	502	37.19	37.08	36.55	130	1	CON	EXERCISE2	40	50
	503	37.19	37.11	36.58	130	1	CON	EXERCISE2	40	50
	504	37.19	37.13	36.59	130	1	CON	EXERCISE2	40	50
	505	37.19	37.14	36.61	130	1	CON	EXERCISE2	40	50
	506	37.19	37.14	36.63	132	1	CON	EXERCISE2	40	50

min	number	Tre	Tes	Tsk-head	HR	ID	condition	period	Ta	RH
	507	37.20	37.14	36.63	133	1	CON	EXERCISE2	40	50
	508	37.21	37.17	36.63	132	1	CON	EXERCISE2	40	50
	509	37.22	37.19	36.64	132	1	CON	EXERCISE2	40	50
	510	37.22	37.20	36.65	134	1	CON	EXERCISE2	40	50
85	511	37.21	37.20	36.64	134	1	CON	EXERCISE2	40	50
	512	37.20	37.19	36.62	131	1	CON	EXERCISE2	40	50
	513	37.21	37.20	36.60	132	1	CON	EXERCISE2	40	50
	514	37.22	37.20	36.57	133	1	CON	EXERCISE2	40	50
	515	37.22	37.19	36.54	133	1	CON	EXERCISE2	40	50
	516	37.22	37.18	36.51	133	1	CON	EXERCISE2	40	50
	517	37.23	37.21	36.50	133	1	CON	EXERCISE2	40	50
	518	37.24	37.24	36.53	133	1	CON	EXERCISE2	40	50
	519	37.24	37.24	36.55	134	1	CON	EXERCISE2	40	50
	520	37.25	37.25	36.57	134	1	CON	EXERCISE2	40	50
	521	37.25	37.26	36.59	134	1	CON	EXERCISE2	40	50
	522	37.24	37.27	36.61	134	1	CON	EXERCISE2	40	50
	523	37.24	37.28	36.63	135	1	CON	EXERCISE2	40	50
	524	37.25	37.29	36.63	135	1	CON	EXERCISE2	40	50
	525	37.26	37.30	36.63	134	1	CON	EXERCISE2	40	50
	526	37.26	37.29	36.62	134	1	CON	EXERCISE2	40	50
	527	37.26	37.29	36.63	135	1	CON	EXERCISE2	40	50
	528	37.26	37.31	36.63	136	1	CON	EXERCISE2	40	50
	529	37.26	37.33	36.64	135	1	CON	EXERCISE2	40	50
	530	37.26	37.33	36.65	135	1	CON	EXERCISE2	40	50
	531	37.27	37.33	36.65	136	1	CON	EXERCISE2	40	50
	532	37.28	37.35	36.66	136	1	CON	EXERCISE2	40	50
	533	37.27	37.37	36.68	135	1	CON	EXERCISE2	40	50
	534	37.27	37.37	36.70	135	1	CON	EXERCISE2	40	50
	535	37.28	37.37	36.72	135	1	CON	EXERCISE2	40	50
	536	37.29	37.38	36.72	135	1	CON	EXERCISE2	40	50
	537	37.29	37.37	36.70	134	1	CON	EXERCISE2	40	50
	538	37.30	37.38	36.71	137	1	CON	EXERCISE2	40	50
	539	37.29	37.30	36.73	140	1	CON	EXERCISE2	40	50
	540	37.29	37.26	36.74	135	1	CON	EXERCISE2	40	50
90	541	37.30	37.33	36.76	135	1	CON	EXERCISE2	40	50
	542	37.31	37.35	36.77	134	1	CON	EXERCISE2	40	50
	543	37.31	37.37	36.75	136	1	CON	EXERCISE2	40	50
	544	37.31	37.17	36.74	135	1	CON	EXERCISE2	40	50
	545	37.31	37.13	36.73	138	1	CON	EXERCISE2	40	50
	546	37.31	37.32	36.72	136	1	CON	EXERCISE2	40	50
	547	37.31	37.36	36.71	136	1	CON	EXERCISE2	40	50
	548	37.32	37.38	36.71	136	1	CON	EXERCISE2	40	50
	549	37.33	37.41	36.73	139	1	CON	EXERCISE2	40	50
	550	37.33	37.42	36.76	139	1	CON	EXERCISE2	40	50
	551	37.33	37.44	36.82	137	1	CON	EXERCISE2	40	50
	552	37.34	37.45	36.85	138	1	CON	EXERCISE2	40	50

min	number	Tre	Tes	Tsk-head	HR	ID	condition	period	Ta	RH
	553	37.35	37.46	36.83	139	1	CON	EXERCISE2	40	50
	554	37.35	37.47	36.83	140	1	CON	EXERCISE2	40	50
	555	37.35	37.48	36.81	141	1	CON	EXERCISE2	40	50
	556	37.35	37.49	36.79	141	1	CON	EXERCISE2	40	50
	557	37.35	37.50	36.80	140	1	CON	EXERCISE2	40	50
	558	37.36	37.51	36.83	140	1	CON	EXERCISE2	40	50
	559	37.37	37.52	36.85	140	1	CON	EXERCISE2	40	50
	560	37.37	37.52	36.85	139	1	CON	EXERCISE2	40	50
	561	37.36	37.52	36.84	137	1	CON	EXERCISE2	40	50
	562	37.36	37.54	36.85	139	1	CON	EXERCISE2	40	50
	563	37.37	37.54	36.85	137	1	CON	EXERCISE2	40	50
	564	37.38	37.55	36.87	137	1	CON	EXERCISE2	40	50
	565	37.38	37.56	36.87	138	1	CON	EXERCISE2	40	50
	566	37.38	37.57	36.86	138	1	CON	EXERCISE2	40	50
	567	37.37	37.57	36.86	140	1	CON	EXERCISE2	40	50
	568	37.38	37.57	36.85	141	1	CON	EXERCISE2	40	50
	569	37.39	37.58	36.85	141	1	CON	EXERCISE2	40	50
	570	37.40	37.60	36.86	142	1	CON	EXERCISE2	40	50
95	571	37.40	37.61	36.87	142	1	CON	EXERCISE2	40	50
	572	37.41	37.62	36.88	141	1	CON	EXERCISE2	40	50
	573	37.41	37.62	36.91	139	1	CON	EXERCISE2	40	50
	574	37.42	37.62	36.94	140	1	CON	EXERCISE2	40	50
	575	37.41	37.62	36.93	141	1	CON	EXERCISE2	40	50
	576	37.41	37.63	36.91	141	1	CON	EXERCISE2	40	50
	577	37.42	37.63	36.89	140	1	CON	EXERCISE2	40	50
	578	37.42	37.64	36.89	142	1	CON	EXERCISE2	40	50
	579	37.42	37.65	36.89	140	1	CON	EXERCISE2	40	50
	580	37.43	37.65	36.90	140	1	CON	EXERCISE2	40	50
	581	37.43	37.66	36.93	140	1	CON	EXERCISE2	40	50
	582	37.43	37.67	36.95	140	1	CON	EXERCISE2	40	50
	583	37.44	37.67	36.98	141	1	CON	EXERCISE2	40	50
	584	37.45	37.68	36.96	142	1	CON	EXERCISE2	40	50
	585	37.45	37.69	36.93	142	1	CON	EXERCISE2	40	50
	586	37.45	37.56	36.93	144	1	CON	EXERCISE2	40	50
	587	37.46	37.52	36.91	142	1	CON	EXERCISE2	40	50
	588	37.47	37.63	36.91	142	1	CON	EXERCISE2	40	50
	589	37.47	37.64	36.94	144	1	CON	EXERCISE2	40	50
	590	37.47	37.65	36.95	144	1	CON	EXERCISE2	40	50
	591	37.47	37.68	36.96	144	1	CON	EXERCISE2	40	50
	592	37.47	37.69	36.94	145	1	CON	EXERCISE2	40	50
	593	37.48	37.69	36.92	147	1	CON	EXERCISE2	40	50
	594	37.49	37.70	36.90	143	1	CON	EXERCISE2	40	50
	595	37.50	37.71	36.88	142	1	CON	EXERCISE2	40	50
	596	37.50	37.72	36.88	142	1	CON	EXERCISE2	40	50
	597	37.49	37.72	36.89	143	1	CON	EXERCISE2	40	50
	598	37.49	37.73	36.89	145	1	CON	EXERCISE2	40	50

min	number	Tre	Tes	Tsk-head	HR	ID	condition	period	Ta	RH
	599	37.50	37.74	36.90	146	1	CON	EXERCISE2	40	50
	600	37.50	37.75	36.90	146	1	CON	EXERCISE2	40	50
100	601	37.51	37.74	36.91	146	1	CON	EXERCISE2	40	50
	602	37.52	37.75	36.94	145	1	CON	EXERCISE2	40	50
	603	37.52	37.75	36.95	143	1	CON	EXERCISE2	40	50
	604	37.52	37.76	36.93	144	1	CON	EXERCISE2	40	50
	605	37.52	37.77	36.91	146	1	CON	EXERCISE2	40	50
	606	37.52	37.77	36.90	146	1	CON	EXERCISE2	40	50
	607	37.53	37.77	36.89	147	1	CON	EXERCISE2	40	50
	608	37.53	37.78	36.90	146	1	CON	EXERCISE2	40	50
	609	37.54	37.79	36.90	146	1	CON	EXERCISE2	40	50
	610	37.54	37.80	36.89	146	1	CON	EXERCISE2	40	50
	611	37.55	37.80	36.92	146	1	CON	EXERCISE2	40	50
	612	37.55	37.80	36.93	144	1	CON	EXERCISE2	40	50
	613	37.55	37.80	36.93	146	1	CON	EXERCISE2	40	50
	614	37.55	37.84	36.97	144	1	CON	EXERCISE2	40	50
	615	37.56	37.87	36.97	144	1	CON	EXERCISE2	40	50
	616	37.56	37.85	36.99	145	1	CON	EXERCISE2	40	50
	617	37.57	37.83	37.01	146	1	CON	EXERCISE2	40	50
	618	37.57	37.83	37.00	146	1	CON	EXERCISE2	40	50
	619	37.57	37.84	37.02	145	1	CON	EXERCISE2	40	50
	620	37.58	37.84	37.03	145	1	CON	EXERCISE2	40	50
	621	37.58	37.84	37.06	143	1	CON	EXERCISE2	40	50
	622	37.58	37.86	37.09	144	1	CON	EXERCISE2	40	50
	623	37.58	37.86	37.07	144	1	CON	EXERCISE2	40	50
	624	37.59	37.85	37.06	146	1	CON	EXERCISE2	40	50
	625	37.60	37.87	37.07	144	1	CON	EXERCISE2	40	50
	626	37.60	37.88	37.08	146	1	CON	EXERCISE2	40	50
	627	37.60	37.82	37.06	150	1	CON	EXERCISE2	40	50
	628	37.60	37.78	37.02	149	1	CON	EXERCISE2	40	50
	629	37.60	37.82	37.01	149	1	CON	EXERCISE2	40	50
	630	37.60	37.84	37.00	150	1	CON	EXERCISE2	40	50
105	631	37.61	37.81	36.99	146	1	CON	EXERCISE2	40	50
	632	37.61	37.82	36.98	150	1	CON	EXERCISE2	40	50
	633	37.62	37.88	37.00	147	1	CON	EXERCISE2	40	50
	634	37.64	37.89	36.99	150	1	CON	EXERCISE2	40	50
	635	37.63	37.89	36.97	149	1	CON	EXERCISE2	40	50
	636	37.63	37.90	36.97	149	1	CON	EXERCISE2	40	50
	637	37.65	37.91	36.99	151	1	CON	EXERCISE2	40	50
	638	37.65	37.92	37.00	150	1	CON	EXERCISE2	40	50
	639	37.65	37.93	37.01	147	1	CON	EXERCISE2	40	50
	640	37.65	37.96	37.06	147	1	CON	EXERCISE2	40	50
	641	37.65	37.97	37.10	150	1	CON	EXERCISE2	40	50
	642	37.66	37.95	37.11	150	1	CON	EXERCISE2	40	50
	643	37.67	37.98	37.12	150	1	CON	EXERCISE2	40	50
	644	37.67	38.02	37.06	147	1	CON	REST3	28	50

min	number	Tre	Tes	Tsk-head	HR	ID	condition	period	Ta	RH
	645	37.67	38.03	36.78	141	1	CON	REST3	28	50
	646	37.67	38.02	36.49	134	1	CON	REST3	28	50
	647	37.68	38.02	36.34	133	1	CON	REST3	28	50
	648	37.69	38.02	36.27	133	1	CON	REST3	28	50
	649	37.68	38.02	36.25	130	1	CON	REST3	28	50
	650	37.68	38.03	36.31	129	1	CON	REST3	28	50
	651	37.69	38.04	36.46	127	1	CON	REST3	28	50
	652	37.69	38.02	36.39	125	1	CON	REST3	28	50
	653	37.69	37.98	36.27	124	1	CON	REST3	28	50
	654	37.69	37.98	36.36	123	1	CON	REST3	28	50
	655	37.70	37.97	36.33	123	1	CON	REST3	28	50
	656	37.70	37.94	36.26	128	1	CON	REST3	28	50
	657	37.71	37.93	36.25	127	1	CON	REST3	28	50
	658	37.70	37.92	36.18	127	1	CON	REST3	28	50
	659	37.69	37.91	36.19	126	1	CON	REST3	28	50
	660	37.68	37.89	36.13	123	1	CON	REST3	28	50
110	661	37.68	37.85	36.07	121	1	CON	REST3	28	50
	662	37.68	37.82	36.10	122	1	CON	REST3	28	50
	663	37.68	37.79	36.09	122	1	CON	REST3	28	50
	664	37.67	37.77	36.02	118	1	CON	REST3	28	50
	665	37.67	37.81	35.94	117	1	CON	REST3	28	50
	666	37.67	37.79	35.76	117	1	CON	REST3	28	50
	667	37.67	37.75	35.63	118	1	CON	REST3	28	50
	668	37.68	37.75	35.78	117	1	CON	REST3	28	50
	669	37.68	37.73	35.86	116	1	CON	REST3	28	50
	670	37.68	37.69	35.80	116	1	CON	REST3	28	50
	671	37.67	37.52	35.75	120	1	CON	REST3	28	50
	672	37.66	37.41	35.72	116	1	CON	REST3	28	50
	673	37.66	37.44	35.71	115	1	CON	REST3	28	50
	674	37.66	37.44	35.73	114	1	CON	REST3	28	50
	675	37.66	37.46	35.74	113	1	CON	REST3	28	50
	676	37.65	37.46	35.63	112	1	CON	REST3	28	50
	677	37.65	37.43	35.52	111	1	CON	REST3	28	50
	678	37.65	37.44	35.54	110	1	CON	REST3	28	50
	679	37.65	37.47	35.56	109	1	CON	REST3	28	50
	680	37.65	37.43	35.62	108	1	CON	REST3	28	50
	681	37.65	37.36	35.54	113	1	CON	REST3	28	50
	682	37.65	37.33	35.53	108	1	CON	REST3	28	50
	683	37.65	37.34	35.68	107	1	CON	REST3	28	50
	684	37.64	37.34	35.69	107	1	CON	REST3	28	50
	685	37.63	37.34	35.65	104	1	CON	REST3	28	50
	686	37.63	37.35	35.59	101	1	CON	REST3	28	50
	687	37.63	37.36	35.49	107	1	CON	REST3	28	50
	688	37.63	37.35	35.34	104	1	CON	REST3	28	50
	689	37.63	37.33	35.25	107	1	CON	REST3	28	50
	690	37.64	37.34	35.25	106	1	CON	REST3	28	50

min	number	Tre	Tes	Tsk-head	HR	ID	condition	period	Ta	RH
115	691	37.63	37.33	35.20	107	1	CON	REST3	28	50
	692	37.62	37.30	35.07	106	1	CON	REST3	28	50
	693	37.61	37.31	34.99	105	1	CON	REST3	28	50
	694	37.61	37.34	34.99	104	1	CON	REST3	28	50
	695	37.61	37.32	35.02	105	1	CON	REST3	28	50
	696	37.61	37.28	35.03	103	1	CON	REST3	28	50
	697	37.62	37.27	35.01	105	1	CON	REST3	28	50
	698	37.61	37.28	34.91	100	1	CON	REST3	28	50
	699	37.61	37.26	34.74	100	1	CON	REST3	28	50
	700	37.61	37.26	34.64	102	1	CON	REST3	28	50
	701	37.62	37.26	34.65	103	1	CON	REST3	28	50
	702	37.64	37.23	34.69	103	1	CON	REST3	28	50
	703	37.62	37.22	34.66	99	1	CON	REST3	28	50
	704	37.60	37.22	34.65	100	1	CON	REST3	28	50
	705	37.61	37.22	34.66	96	1	CON	REST3	28	50
	706	37.62	37.22	34.61	100	1	CON	REST3	28	50
	707	37.61	37.19	34.58	99	1	CON	REST3	28	50
	708	37.61	37.18	34.60	100	1	CON	REST3	28	50
	709	37.60	37.18	34.61	101	1	CON	REST3	28	50
0	1	36.89	36.82	35.25	70	2	CON	REST1	28	50
	2	36.89	36.83	35.25	69	2	CON	REST1	28	50
	3	36.89	36.81	35.24	69	2	CON	REST1	28	50
	4	36.88	36.81	35.22	70	2	CON	REST1	28	50
	5	36.88	36.84	35.23	71	2	CON	REST1	28	50
	6	36.89	36.86	35.25	70	2	CON	REST1	28	50
	7	36.89	36.85	35.23	71	2	CON	REST1	28	50
	8	36.88	36.83	35.21	72	2	CON	REST1	28	50
	9	36.87	36.84	35.18	72	2	CON	REST1	28	50
	10	36.87	36.85	35.16	71	2	CON	REST1	28	50
	11	36.88	36.85	35.17	76	2	CON	REST1	28	50
	12	36.89	36.86	35.14	72	2	CON	REST1	28	50
	13	36.89	36.86	35.14	71	2	CON	REST1	28	50
	14	36.88	36.88	35.16	72	2	CON	REST1	28	50
	15	36.88	36.89	35.18	69	2	CON	REST1	28	50
	16	36.89	36.89	35.17	69	2	CON	REST1	28	50
	17	36.89	36.89	35.15	68	2	CON	REST1	28	50
	18	36.88	36.88	35.17	68	2	CON	REST1	28	50
	19	36.88	36.86	35.18	70	2	CON	REST1	28	50
	20	36.89	36.88	35.18	79	2	CON	REST1	28	50
	21	36.89	36.89	35.17	77	2	CON	REST1	28	50
	22	36.89	36.85	35.19	74	2	CON	REST1	28	50
	23	36.89	36.84	35.20	70	2	CON	REST1	28	50
	24	36.89	36.88	35.20	72	2	CON	REST1	28	50
	25	36.89	36.90	35.20	68	2	CON	REST1	28	50
	26	36.88	36.89	35.19	66	2	CON	REST1	28	50
	27	36.88	36.87	35.19	70	2	CON	REST1	28	50

min	number	Tre	Tes	Tsk-head	HR	ID	condition	period	Ta	RH
	28	36.89	36.86	35.20	72	2	CON	REST1	28	50
	29	36.89	36.88	35.19	71	2	CON	REST1	28	50
	30	36.88	36.90	35.18	67	2	CON	REST1	28	50
5	31	36.88	36.90	35.18	64	2	CON	REST1	28	50
	32	36.88	36.90	35.17	66	2	CON	REST1	28	50
	33	36.88	36.90	35.16	67	2	CON	REST1	28	50
	34	36.89	36.88	35.16	67	2	CON	REST1	28	50
	35	36.89	36.87	35.17	66	2	CON	REST1	28	50
	36	36.88	36.88	35.15	69	2	CON	REST1	28	50
	37	36.88	36.89	35.14	72	2	CON	REST1	28	50
	38	36.88	36.91	35.14	70	2	CON	REST1	28	50
	39	36.88	36.92	35.15	67	2	CON	REST1	28	50
	40	36.88	36.91	35.14	66	2	CON	REST1	28	50
	41	36.88	36.90	35.14	68	2	CON	REST1	28	50
	42	36.88	36.91	35.14	66	2	CON	REST1	28	50
	43	36.89	36.90	35.14	69	2	CON	REST1	28	50
	44	36.89	36.89	35.15	68	2	CON	REST1	28	50
	45	36.87	36.88	35.15	65	2	CON	REST1	28	50
	46	36.87	36.88	35.16	68	2	CON	REST1	28	50
	47	36.88	36.89	35.17	71	2	CON	REST1	28	50
	48	36.88	36.90	35.17	71	2	CON	REST1	28	50
	49	36.88	36.90	35.17	74	2	CON	REST1	28	50
	50	36.87	36.90	35.18	73	2	CON	REST1	28	50
	51	36.88	36.86	35.18	78	2	CON	REST1	28	50
	52	36.88	36.84	35.19	73	2	CON	REST1	28	50
	53	36.88	36.90	35.20	67	2	CON	REST1	28	50
	54	36.88	36.90	35.18	64	2	CON	REST1	28	50
	55	36.88	36.88	35.18	65	2	CON	REST1	28	50
	56	36.88	36.89	35.18	67	2	CON	REST1	28	50
	57	36.88	36.90	35.16	70	2	CON	REST1	28	50
	58	36.88	36.89	35.14	72	2	CON	REST1	28	50
	59	36.87	36.89	35.17	72	2	CON	REST1	28	50
	60	36.87	36.89	35.19	70	2	CON	REST1	28	50
10	61	36.87	36.89	35.19	74	2	CON	REST1	28	50
	62	36.88	36.90	35.20	73	2	CON	REST1	28	50
	63	36.88	36.91	35.20	72	2	CON	REST1	28	50
	64	36.89	36.91	35.21	70	2	CON	REST1	28	50
	65	36.88	36.90	35.22	69	2	CON	REST1	28	50
	66	36.88	36.91	35.21	71	2	CON	REST1	28	50
	67	36.89	36.92	35.21	69	2	CON	REST1	28	50
	68	36.88	36.92	35.22	71	2	CON	REST1	28	50
	69	36.88	36.94	35.22	71	2	CON	REST1	28	50
	70	36.89	36.95	35.22	71	2	CON	REST1	28	50
	71	36.88	36.95	35.22	75	2	CON	REST1	28	50
	72	36.88	36.94	35.23	72	2	CON	REST1	28	50
	73	36.87	36.91	35.24	73	2	CON	REST1	28	50

min	number	Tre	Tes	Tsk-head	HR	ID	condition	period	Ta	RH
	74	36.87	36.91	35.24	72	2	CON	REST1	28	50
	75	36.88	36.94	35.22	71	2	CON	REST1	28	50
	76	36.88	36.95	35.20	74	2	CON	REST1	28	50
	77	36.89	36.93	35.20	73	2	CON	REST1	28	50
	78	36.90	36.95	35.20	75	2	CON	REST1	28	50
	79	36.90	36.95	35.22	76	2	CON	REST1	28	50
	80	36.90	36.93	35.23	78	2	CON	REST1	28	50
	81	36.90	36.93	35.21	73	2	CON	REST1	28	50
	82	36.90	36.92	35.18	71	2	CON	REST1	28	50
	83	36.90	36.92	35.19	74	2	CON	REST1	28	50
	84	36.89	36.87	35.22	75	2	CON	REST1	28	50
	85	36.89	36.85	35.21	71	2	CON	REST1	28	50
	86	36.90	36.89	35.23	80	2	CON	REST1	28	50
	87	36.90	36.91	35.26	88	2	CON	REST1	28	50
	88	36.90	36.89	35.29	73	2	CON	REST1	28	50
	89	36.90	36.87	35.27	72	2	CON	REST1	28	50
	90	36.90	36.84	35.26	67	2	CON	REST1	28	50
15	91	36.90	36.84	35.29	68	2	CON	REST1	28	50
	92	36.90	36.85	35.31	69	2	CON	REST1	28	50
	93	36.91	36.85	35.32	72	2	CON	REST1	28	50
	94	36.91	36.87	35.34	75	2	CON	REST1	28	50
	95	36.92	36.87	35.35	73	2	CON	REST1	28	50
	96	36.93	36.86	35.35	70	2	CON	REST1	28	50
	97	36.91	36.84	35.28	84	2	CON	REST1	28	50
	98	36.90	36.82	35.23	95	2	CON	REST1	28	50
	99	36.89	36.80	35.25	90	2	CON	REST1	28	50
	100	36.87	36.78	35.24	82	2	CON	REST1	28	50
	101	36.87	36.78	35.25	81	2	CON	REST1	28	50
	102	36.88	36.82	35.27	77	2	CON	REST1	28	50
	103	36.89	36.83	35.24	78	2	CON	REST1	40	50
	104	36.89	36.81	35.41	95	2	CON	REST1	40	50
	105	36.90	36.84	35.70	83	2	CON	REST1	40	50
	106	36.90	36.86	35.83	76	2	CON	REST1	40	50
	107	36.89	36.84	35.94	82	2	CON	REST1	40	50
	108	36.89	36.86	36.04	81	2	CON	REST1	40	50
	109	36.90	36.87	36.10	74	2	CON	REST1	40	50
	110	36.89	36.83	36.15	73	2	CON	REST1	40	50
	111	36.91	36.81	36.20	79	2	CON	REST1	40	50
	112	36.93	36.82	36.26	77	2	CON	REST1	40	50
	113	36.93	36.80	36.31	77	2	CON	REST1	40	50
	114	36.93	36.79	36.35	73	2	CON	REST1	40	50
	115	36.93	36.81	36.38	78	2	CON	REST1	40	50
	116	36.92	36.80	36.40	74	2	CON	REST1	40	50
	117	36.91	36.77	36.42	77	2	CON	REST1	40	50
	118	36.90	36.76	36.45	74	2	CON	REST1	40	50
	119	36.90	36.81	36.49	83	2	CON	REST1	40	50

min	number	Tre	Tes	Tsk-head	HR	ID	condition	period	Ta	RH
	120	36.90	36.80	36.51	81	2	CON	REST1	40	50
20	121	36.90	36.77	36.53	70	2	CON	REST1	40	50
	122	36.90	36.80	36.55	76	2	CON	REST1	40	50
	123	36.89	36.82	36.57	75	2	CON	REST1	40	50
	124	36.89	36.81	36.60	77	2	CON	REST1	40	50
	125	36.89	36.79	36.61	87	2	CON	REST1	40	50
	126	36.89	36.79	36.63	85	2	CON	REST1	40	50
	127	36.89	36.77	36.65	83	2	CON	REST1	40	50
	128	36.90	36.76	36.66	82	2	CON	REST1	40	50
	129	36.90	36.76	36.68	83	2	CON	REST1	40	50
	130	36.89	36.76	36.69	83	2	CON	REST1	40	50
	131	36.89	36.79	36.71	78	2	CON	REST1	40	50
	132	36.89	36.80	36.72	73	2	CON	REST1	40	50
	133	36.89	36.81	36.73	78	2	CON	REST1	40	50
	134	36.87	36.80	36.75	95	2	CON	REST1	40	50
	135	36.86	36.83	36.76	102	2	CON	REST1	40	50
	136	36.87	36.86	36.75	86	2	CON	REST1	40	50
	137	36.86	36.80	36.75	86	2	CON	REST1	40	50
	138	36.85	36.74	36.74	80	2	CON	REST1	40	50
	139	36.85	36.73	36.74	82	2	CON	EXERCISE1	40	50
	140	36.84	36.71	36.75	93	2	CON	EXERCISE1	40	50
	141	36.83	36.71	36.77	91	2	CON	EXERCISE1	40	50
	142	36.83	36.72	36.79	94	2	CON	EXERCISE1	40	50
	143	36.83	36.71	36.80	99	2	CON	EXERCISE1	40	50
	144	36.82	36.70	36.80	104	2	CON	EXERCISE1	40	50
	145	36.81	36.70	36.80	103	2	CON	EXERCISE1	40	50
	146	36.82	36.71	36.82	100	2	CON	EXERCISE1	40	50
	147	36.82	36.72	36.83	104	2	CON	EXERCISE1	40	50
	148	36.82	36.72	36.83	98	2	CON	EXERCISE1	40	50
	149	36.81	36.73	36.84	92	2	CON	EXERCISE1	40	50
	150	36.80	36.75	36.84	102	2	CON	EXERCISE1	40	50
25	151	36.81	36.74	36.84	108	2	CON	EXERCISE1	40	50
	152	36.81	36.68	36.84	101	2	CON	EXERCISE1	40	50
	153	36.80	36.67	36.84	107	2	CON	EXERCISE1	40	50
	154	36.81	36.70	36.84	104	2	CON	EXERCISE1	40	50
	155	36.81	36.71	36.85	100	2	CON	EXERCISE1	40	50
	156	36.81	36.71	36.85	95	2	CON	EXERCISE1	40	50
	157	36.80	36.73	36.86	101	2	CON	EXERCISE1	40	50
	158	36.80	36.75	36.85	102	2	CON	EXERCISE1	40	50
	159	36.80	36.75	36.85	107	2	CON	EXERCISE1	40	50
	160	36.80	36.74	36.86	106	2	CON	EXERCISE1	40	50
	161	36.79	36.74	36.85	105	2	CON	EXERCISE1	40	50
	162	36.78	36.77	36.85	106	2	CON	EXERCISE1	40	50
	163	36.79	36.79	36.86	104	2	CON	EXERCISE1	40	50
	164	36.79	36.78	36.87	106	2	CON	EXERCISE1	40	50
	165	36.79	36.77	36.88	103	2	CON	EXERCISE1	40	50

min	number	Tre	Tes	Tsk-head	HR	ID	condition	period	Ta	RH
	166	36.79	36.78	36.88	107	2	CON	EXERCISE1	40	50
	167	36.79	36.81	36.88	107	2	CON	EXERCISE1	40	50
	168	36.79	36.82	36.88	106	2	CON	EXERCISE1	40	50
	169	36.79	36.81	36.89	108	2	CON	EXERCISE1	40	50
	170	36.79	36.80	36.89	104	2	CON	EXERCISE1	40	50
	171	36.80	36.82	36.88	100	2	CON	EXERCISE1	40	50
	172	36.80	36.83	36.88	104	2	CON	EXERCISE1	40	50
	173	36.81	36.82	36.88	102	2	CON	EXERCISE1	40	50
	174	36.81	36.84	36.87	104	2	CON	EXERCISE1	40	50
	175	36.81	36.84	36.87	107	2	CON	EXERCISE1	40	50
	176	36.81	36.86	36.88	105	2	CON	EXERCISE1	40	50
	177	36.81	36.88	36.88	107	2	CON	EXERCISE1	40	50
	178	36.83	36.87	36.88	105	2	CON	EXERCISE1	40	50
	179	36.84	36.85	36.88	108	2	CON	EXERCISE1	40	50
	180	36.84	36.87	36.89	111	2	CON	EXERCISE1	40	50
30	181	36.84	36.91	36.89	114	2	CON	EXERCISE1	40	50
	182	36.84	36.89	36.89	109	2	CON	EXERCISE1	40	50
	183	36.83	36.90	36.89	108	2	CON	EXERCISE1	40	50
	184	36.84	36.90	36.89	108	2	CON	EXERCISE1	40	50
	185	36.85	36.89	36.90	112	2	CON	EXERCISE1	40	50
	186	36.85	36.90	36.91	103	2	CON	EXERCISE1	40	50
	187	36.85	36.91	36.92	107	2	CON	EXERCISE1	40	50
	188	36.86	36.89	36.92	109	2	CON	EXERCISE1	40	50
	189	36.85	36.89	36.92	110	2	CON	EXERCISE1	40	50
	190	36.85	36.90	36.93	110	2	CON	EXERCISE1	40	50
	191	36.86	36.90	36.92	110	2	CON	EXERCISE1	40	50
	192	36.86	36.89	36.92	110	2	CON	EXERCISE1	40	50
	193	36.86	36.93	36.93	112	2	CON	EXERCISE1	40	50
	194	36.85	36.94	36.92	109	2	CON	EXERCISE1	40	50
	195	36.84	36.94	36.91	109	2	CON	EXERCISE1	40	50
	196	36.84	36.94	36.91	111	2	CON	EXERCISE1	40	50
	197	36.84	36.95	36.92	110	2	CON	EXERCISE1	40	50
	198	36.84	36.97	36.92	113	2	CON	EXERCISE1	40	50
	199	36.85	36.97	36.92	111	2	CON	EXERCISE1	40	50
	200	36.85	36.95	36.92	111	2	CON	EXERCISE1	40	50
	201	36.86	36.95	36.92	111	2	CON	EXERCISE1	40	50
	202	36.86	36.97	36.91	111	2	CON	EXERCISE1	40	50
	203	36.86	36.98	36.91	111	2	CON	EXERCISE1	40	50
	204	36.86	36.98	36.92	99	2	CON	EXERCISE1	40	50
	205	36.87	37.00	36.93	112	2	CON	EXERCISE1	40	50
	206	36.88	36.99	36.93	112	2	CON	EXERCISE1	40	50
	207	36.89	36.98	36.93	114	2	CON	EXERCISE1	40	50
	208	36.89	36.99	36.92	114	2	CON	EXERCISE1	40	50
	209	36.89	36.99	36.90	116	2	CON	EXERCISE1	40	50
	210	36.89	37.01	36.90	115	2	CON	EXERCISE1	40	50
35	211	36.88	36.89	36.90	120	2	CON	EXERCISE1	40	50

min	number	Tre	Tes	Tsk-head	HR	ID	condition	period	Ta	RH
	212	36.88	36.86	36.91	115	2	CON	EXERCISE1	40	50
	213	36.89	36.96	36.91	111	2	CON	EXERCISE1	40	50
	214	36.88	36.97	36.91	113	2	CON	EXERCISE1	40	50
	215	36.88	37.01	36.91	114	2	CON	EXERCISE1	40	50
	216	36.88	37.02	36.91	117	2	CON	EXERCISE1	40	50
	217	36.88	37.03	36.91	117	2	CON	EXERCISE1	40	50
	218	36.89	37.05	36.92	116	2	CON	EXERCISE1	40	50
	219	36.89	37.05	36.92	115	2	CON	EXERCISE1	40	50
	220	36.89	37.04	36.93	115	2	CON	EXERCISE1	40	50
	221	36.90	37.04	36.94	115	2	CON	EXERCISE1	40	50
	222	36.90	37.06	36.95	115	2	CON	EXERCISE1	40	50
	223	36.90	37.08	36.96	115	2	CON	EXERCISE1	40	50
	224	36.90	37.08	36.96	113	2	CON	EXERCISE1	40	50
	225	36.90	37.07	36.95	114	2	CON	EXERCISE1	40	50
	226	36.89	37.08	36.95	117	2	CON	EXERCISE1	40	50
	227	36.90	37.07	36.97	116	2	CON	EXERCISE1	40	50
	228	36.91	37.07	36.98	113	2	CON	EXERCISE1	40	50
	229	36.92	37.09	36.98	111	2	CON	EXERCISE1	40	50
	230	36.92	37.11	36.98	114	2	CON	EXERCISE1	40	50
	231	36.92	37.13	36.99	113	2	CON	EXERCISE1	40	50
	232	36.94	37.12	37.00	116	2	CON	EXERCISE1	40	50
	233	36.95	37.13	36.99	116	2	CON	EXERCISE1	40	50
	234	36.94	37.14	36.99	117	2	CON	EXERCISE1	40	50
	235	36.95	37.12	37.00	116	2	CON	EXERCISE1	40	50
	236	36.96	37.12	36.99	114	2	CON	EXERCISE1	40	50
	237	36.96	37.14	36.98	118	2	CON	EXERCISE1	40	50
	238	36.96	37.16	36.98	116	2	CON	EXERCISE1	40	50
	239	36.96	37.18	36.98	114	2	CON	EXERCISE1	40	50
	240	36.97	37.17	36.98	115	2	CON	EXERCISE1	40	50
40	241	36.97	37.15	36.98	115	2	CON	EXERCISE1	40	50
	242	36.98	37.16	37.00	113	2	CON	EXERCISE1	40	50
	243	36.98	37.18	37.00	117	2	CON	EXERCISE1	40	50
	244	36.98	37.16	36.99	117	2	CON	EXERCISE1	40	50
	245	36.99	37.17	36.99	117	2	CON	EXERCISE1	40	50
	246	36.99	37.18	37.00	117	2	CON	EXERCISE1	40	50
	247	36.99	37.18	37.00	117	2	CON	EXERCISE1	40	50
	248	37.00	37.18	37.00	117	2	CON	EXERCISE1	40	50
	249	37.01	37.19	37.00	119	2	CON	EXERCISE1	40	50
	250	37.02	37.20	37.00	120	2	CON	EXERCISE1	40	50
	251	37.03	37.20	37.01	122	2	CON	EXERCISE1	40	50
	252	37.02	37.21	37.02	125	2	CON	EXERCISE1	40	50
	253	37.02	37.21	37.02	121	2	CON	EXERCISE1	40	50
	254	37.03	37.22	37.03	118	2	CON	EXERCISE1	40	50
	255	37.03	37.23	37.03	117	2	CON	EXERCISE1	40	50
	256	37.03	37.25	37.03	118	2	CON	EXERCISE1	40	50
	257	37.04	37.25	37.04	122	2	CON	EXERCISE1	40	50

min	number	Tre	Tes	Tsk-head	HR	ID	condition	period	Ta	RH
	258	37.03	37.22	37.04	120	2	CON	EXERCISE1	40	50
	259	37.03	37.10	37.03	124	2	CON	EXERCISE1	40	50
	260	37.04	37.13	37.04	119	2	CON	EXERCISE1	40	50
	261	37.04	37.25	37.05	120	2	CON	EXERCISE1	40	50
	262	37.03	37.24	37.05	119	2	CON	EXERCISE1	40	50
	263	37.02	37.24	37.05	118	2	CON	EXERCISE1	40	50
	264	37.03	37.26	37.05	118	2	CON	EXERCISE1	40	50
	265	37.04	37.30	37.04	118	2	CON	EXERCISE1	40	50
	266	37.03	37.31	37.04	122	2	CON	EXERCISE1	40	50
	267	37.03	37.30	37.05	122	2	CON	EXERCISE1	40	50
	268	37.03	37.29	37.05	120	2	CON	EXERCISE1	40	50
	269	37.03	37.32	37.05	122	2	CON	EXERCISE1	40	50
	270	37.04	37.34	37.06	124	2	CON	EXERCISE1	40	50
45	271	37.04	37.32	37.05	124	2	CON	EXERCISE1	40	50
	272	37.05	37.29	37.06	120	2	CON	EXERCISE1	40	50
	273	37.06	37.29	37.08	118	2	CON	EXERCISE1	40	50
	274	37.06	37.30	37.09	119	2	CON	EXERCISE1	40	50
	275	37.06	37.30	37.08	121	2	CON	EXERCISE1	40	50
	276	37.06	37.31	37.06	121	2	CON	EXERCISE1	40	50
	277	37.07	37.32	37.06	120	2	CON	EXERCISE1	40	50
	278	37.07	37.33	37.06	120	2	CON	EXERCISE1	40	50
	279	37.07	37.35	37.07	122	2	CON	EXERCISE1	40	50
	280	37.06	37.36	37.08	120	2	CON	EXERCISE1	40	50
	281	37.06	37.37	37.08	120	2	CON	EXERCISE1	40	50
	282	37.07	37.37	37.09	123	2	CON	EXERCISE1	40	50
	283	37.08	37.37	37.09	122	2	CON	EXERCISE1	40	50
	284	37.09	37.39	37.09	120	2	CON	EXERCISE1	40	50
	285	37.09	37.43	37.10	121	2	CON	EXERCISE1	40	50
	286	37.08	37.38	37.09	123	2	CON	EXERCISE1	40	50
	287	37.09	37.33	37.10	123	2	CON	EXERCISE1	40	50
	288	37.10	37.39	37.11	123	2	CON	EXERCISE1	40	50
	289	37.10	37.42	37.10	124	2	CON	EXERCISE1	40	50
	290	37.10	37.42	37.10	122	2	CON	EXERCISE1	40	50
	291	37.11	37.43	37.10	124	2	CON	EXERCISE1	40	50
	292	37.11	37.43	37.11	124	2	CON	EXERCISE1	40	50
	293	37.11	37.44	37.12	127	2	CON	EXERCISE1	40	50
	294	37.11	37.47	37.13	123	2	CON	EXERCISE1	40	50
	295	37.11	37.47	37.10	122	2	CON	EXERCISE1	40	50
	296	37.12	37.48	37.09	126	2	CON	EXERCISE1	40	50
	297	37.13	37.50	37.12	127	2	CON	EXERCISE1	40	50
	298	37.13	37.50	37.13	126	2	CON	EXERCISE1	40	50
	299	37.13	37.49	37.14	127	2	CON	EXERCISE1	40	50
	300	37.15	37.49	37.16	125	2	CON	EXERCISE1	40	50
50	301	37.15	37.49	37.16	123	2	CON	EXERCISE1	40	50
	302	37.14	37.50	37.14	125	2	CON	EXERCISE1	40	50
	303	37.15	37.53	37.13	124	2	CON	EXERCISE1	40	50

min	number	Tre	Tes	Tsk-head	HR	ID	condition	period	Ta	RH
	304	37.15	37.52	37.13	124	2	CON	EXERCISE1	40	50
	305	37.15	37.52	37.11	125	2	CON	EXERCISE1	40	50
	306	37.16	37.52	37.11	125	2	CON	EXERCISE1	40	50
	307	37.16	37.53	37.12	126	2	CON	EXERCISE1	40	50
	308	37.17	37.53	37.12	125	2	CON	EXERCISE1	40	50
	309	37.17	37.53	37.13	125	2	CON	EXERCISE1	40	50
	310	37.17	37.53	37.13	125	2	CON	EXERCISE1	40	50
	311	37.17	37.54	37.14	122	2	CON	EXERCISE1	40	50
	312	37.18	37.55	37.15	124	2	CON	EXERCISE1	40	50
	313	37.18	37.57	37.17	123	2	CON	EXERCISE1	40	50
	314	37.18	37.60	37.18	123	2	CON	EXERCISE1	40	50
	315	37.19	37.58	37.19	128	2	CON	EXERCISE1	40	50
	316	37.18	37.56	37.18	126	2	CON	EXERCISE1	40	50
	317	37.19	37.59	37.17	125	2	CON	EXERCISE1	40	50
	318	37.20	37.61	37.17	123	2	CON	EXERCISE1	40	50
	319	37.20	37.63	37.18	123	2	CON	EXERCISE1	40	50
	320	37.21	37.63	37.22	122	2	CON	REST2	28	50
	321	37.21	37.60	37.23	120	2	CON	REST2	28	50
	322	37.20	37.56	37.00	127	2	CON	REST2	28	50
	323	37.19	37.60	36.77	115	2	CON	REST2	28	50
	324	37.20	37.64	36.71	107	2	CON	REST2	28	50
	325	37.22	37.62	36.63	107	2	CON	REST2	28	50
	326	37.23	37.49	36.44	115	2	CON	REST2	28	50
	327	37.24	37.45	36.24	112	2	CON	REST2	28	50
	328	37.24	37.55	36.17	110	2	CON	REST2	28	50
	329	37.24	37.44	36.13	111	2	CON	REST2	28	50
	330	37.25	37.45	36.15	105	2	CON	REST2	28	50
55	331	37.24	37.59	36.17	108	2	CON	REST2	28	50
	332	37.24	37.65	36.11	124	2	CON	REST2	28	50
	333	37.23	37.67	36.09	112	2	CON	REST2	28	50
	334	37.22	37.66	36.05	105	2	CON	REST2	28	50
	335	37.23	37.62	36.04	109	2	CON	REST2	28	50
	336	37.24	37.59	36.09	103	2	CON	REST2	28	50
	337	37.25	37.62	36.01	104	2	CON	REST2	28	50
	338	37.26	37.67	35.93	102	2	CON	REST2	28	50
	339	37.27	31.68	35.95	109	2	CON	REST2	28	50
	340	37.27	27.10	35.98	120	2	CON	REST2	28	50
	341	37.27	30.02	36.02	109	2	CON	REST2	28	50
	342	37.26	28.31	36.07	117	2	CON	REST2	28	50
	343	37.26	25.75	36.10	118	2	CON	REST2	28	50
	344	37.27	28.25	36.08	101	2	CON	REST2	28	50
	345	37.28	30.65	36.03	102	2	CON	REST2	28	50
	346	37.29	31.98	36.00	94	2	CON	REST2	28	50
	347	37.29	32.95	36.01	94	2	CON	REST2	28	50
	348	37.29	33.35	36.00	97	2	CON	REST2	28	50
	349	37.29	33.84	36.01	93	2	CON	REST2	28	50

min	number	Tre	Tes	Tsk-head	HR	ID	condition	period	Ta	RH
	350	37.30	34.20	36.00	93	2	CON	REST2	28	50
	351	37.30	33.11	35.98	100	2	CON	REST2	28	50
	352	37.31	33.11	35.96	95	2	CON	REST2	28	50
	353	37.30	33.08	35.92	98	2	CON	REST2	28	50
	354	37.31	33.05	35.93	89	2	CON	REST2	28	50
	355	37.32	34.47	35.92	89	2	CON	REST2	28	50
	356	37.32	34.88	35.84	90	2	CON	REST2	28	50
	357	37.32	35.13	35.80	93	2	CON	REST2	28	50
	358	37.32	35.34	35.81	94	2	CON	REST2	28	50
	359	37.32	35.53	35.80	90	2	CON	REST2	28	50
	360	37.32	35.66	35.77	89	2	CON	REST2	28	50
60	361	37.33	35.79	35.76	88	2	CON	REST2	28	50
	362	37.32	35.91	35.73	90	2	CON	REST2	28	50
	363	37.32	36.01	35.65	89	2	CON	REST2	28	50
	364	37.32	36.08	35.61	86	2	CON	REST2	28	50
	365	37.32	33.49	35.63	96	2	CON	REST2	28	50
	366	37.32	29.62	35.62	104	2	CON	REST2	28	50
	367	37.32	30.44	35.56	88	2	CON	REST2	28	50
	368	37.31	32.68	35.55	96	2	CON	REST2	28	50
	369	37.31	33.45	35.57	85	2	CON	REST2	28	50
	370	37.32	34.26	35.54	83	2	CON	REST2	28	50
	371	37.32	34.62	35.49	82	2	CON	REST2	28	50
	372	37.32	34.90	35.48	85	2	CON	REST2	28	50
	373	37.33	35.12	35.47	86	2	CON	REST2	28	50
	374	37.33	35.29	35.46	86	2	CON	REST2	28	50
	375	37.33	35.46	35.43	84	2	CON	REST2	28	50
	376	37.33	35.61	35.43	83	2	CON	REST2	28	50
	377	37.33	35.71	35.45	83	2	CON	REST2	28	50
	378	37.33	35.80	35.43	79	2	CON	REST2	28	50
	379	37.32	35.86	35.46	81	2	CON	REST2	28	50
	380	37.32	35.91	35.50	81	2	CON	REST2	28	50
	381	37.32	35.99	35.50	80	2	CON	REST2	28	50
	382	37.31	36.04	35.49	82	2	CON	REST2	28	50
	383	37.31	36.06	35.47	84	2	CON	REST2	28	50
	384	37.31	36.11	35.43	87	2	CON	REST2	28	50
	385	37.31	30.83	35.47	98	2	CON	REST2	28	50
	386	37.31	27.97	35.47	74	2	CON	REST2	28	50
	387	37.30	31.60	35.41	86	2	CON	REST2	28	50
	388	37.30	33.12	35.39	82	2	CON	REST2	28	50
	389	37.30	33.53	35.37	81	2	CON	REST2	28	50
	390	37.30	33.96	35.33	74	2	CON	REST2	28	50
65	391	37.30	34.43	35.33	78	2	CON	REST2	28	50
	392	37.31	34.59	35.37	85	2	CON	REST2	28	50
	393	37.30	31.97	35.37	87	2	CON	REST2	28	50
	394	37.30	30.99	35.31	90	2	CON	REST2	28	50
	395	37.29	33.14	35.27	80	2	CON	REST2	28	50

min	number	Tre	Tes	Tsk-head	HR	ID	condition	period	Ta	RH
	396	37.29	31.05	35.31	88	2	CON	REST2	28	50
	397	37.29	30.08	35.30	93	2	CON	REST2	28	50
	398	37.29	32.22	35.24	82	2	CON	REST2	28	50
	399	37.29	33.16	35.26	78	2	CON	REST2	28	50
	400	37.29	33.73	35.24	77	2	CON	REST2	28	50
	401	37.28	34.08	35.20	75	2	CON	REST2	28	50
	402	37.27	34.43	35.20	79	2	CON	REST2	28	50
	403	37.27	34.65	35.18	87	2	CON	REST2	28	50
	404	37.27	34.82	35.17	81	2	CON	REST2	28	50
	405	37.27	35.04	35.16	73	2	CON	REST2	28	50
	406	37.28	35.18	35.16	79	2	CON	REST2	28	50
	407	37.28	35.32	35.18	79	2	CON	REST2	28	50
	408	37.28	34.73	35.17	75	2	CON	REST2	28	50
	409	37.27	34.05	35.12	88	2	CON	REST2	28	50
	410	37.26	34.65	35.16	96	2	CON	REST2	28	50
	411	37.26	35.32	35.14	110	2	CON	REST2	28	50
	412	37.26	35.47	35.10	64	2	CON	REST2	28	50
	413	37.26	35.62	35.11	71	2	CON	REST2	28	50
	414	37.26	35.89	35.09	94	2	CON	REST2	28	50
	415	37.26	36.08	35.04	83	2	CON	REST2	28	50
	416	37.26	36.07	34.97	76	2	CON	REST2	28	50
	417	37.25	36.08	34.89	82	2	CON	REST2	28	50
	418	37.26	36.18	34.80	87	2	CON	REST2	28	50
	419	37.25	36.27	34.67	86	2	CON	REST2	28	50
	420	37.24	36.28	34.71	90	2	CON	REST2	28	50
70	421	37.25	36.34	34.87	85	2	CON	REST2	28	50
	422	37.24	36.39	34.90	84	2	CON	REST2	28	50
	423	37.24	36.42	34.91	81	2	CON	REST2	28	50
	424	37.23	36.45	34.86	79	2	CON	REST2	28	50
	425	37.22	36.48	34.74	80	2	CON	REST2	28	50
	426	37.21	36.51	34.69	79	2	CON	REST2	28	50
	427	37.22	36.54	34.67	80	2	CON	REST2	28	50
	428	37.22	36.56	34.67	95	2	CON	REST2	28	50
	429	37.22	36.60	34.64	84	2	CON	REST2	28	50
	430	37.22	35.53	34.58	85	2	CON	REST2	28	50
	431	37.21	35.01	34.57	83	2	CON	REST2	28	50
	432	37.19	35.79	34.53	98	2	CON	REST2	28	50
	433	37.19	36.18	34.49	82	2	CON	REST2	28	50
	434	37.20	36.39	34.53	90	2	CON	REST2	28	50
	435	37.20	36.40	34.57	86	2	CON	REST2	28	50
	436	37.20	36.43	34.56	84	2	CON	REST2	28	50
	437	37.20	36.48	34.56	80	2	CON	REST2	28	50
	438	37.19	36.50	34.59	78	2	CON	REST2	28	50
	439	37.19	36.52	34.62	77	2	CON	REST2	40	50
	440	37.18	36.57	34.78	104	2	CON	REST2	40	50
	441	37.17	36.63	35.06	89	2	CON	REST2	40	50

min	number	Tre	Tes	Tsk-head	HR	ID	condition	period	Ta	RH
	442	37.16	36.53	35.25	100	2	CON	REST2	40	50
	443	37.16	36.56	35.34	99	2	CON	REST2	40	50
	444	37.17	36.76	35.41	90	2	CON	REST2	40	50
	445	37.18	36.79	35.51	83	2	CON	REST2	40	50
	446	37.18	36.79	35.63	71	2	CON	REST2	40	50
	447	37.19	36.80	35.70	77	2	CON	REST2	40	50
	448	37.18	36.80	35.73	83	2	CON	REST2	40	50
	449	37.18	36.83	35.77	79	2	CON	REST2	40	50
	450	37.19	36.86	35.84	79	2	CON	REST2	40	50
75	451	37.19	36.86	35.91	81	2	CON	REST2	40	50
	452	37.20	36.87	35.94	85	2	CON	REST2	40	50
	453	37.20	36.88	35.99	83	2	CON	REST2	40	50
	454	37.21	36.88	36.05	81	2	CON	REST2	40	50
	455	37.21	36.91	36.09	82	2	CON	REST2	40	50
	456	37.22	36.92	36.13	80	2	CON	REST2	40	50
	457	37.21	36.90	36.18	76	2	CON	REST2	40	50
	458	37.21	36.89	36.21	102	2	CON	REST2	40	50
	459	37.21	36.76	36.21	109	2	CON	REST2	40	50
	460	37.22	36.70	36.20	92	2	CON	REST2	40	50
	461	37.23	36.82	36.22	86	2	CON	REST2	40	50
	462	37.23	36.86	36.26	88	2	CON	REST2	40	50
	463	37.22	36.82	36.29	84	2	CON	EXERCISE2	40	50
	464	37.22	36.78	36.32	87	2	CON	EXERCISE2	40	50
	465	37.23	36.85	36.37	98	2	CON	EXERCISE2	40	50
	466	37.23	36.20	36.39	108	2	CON	EXERCISE2	40	50
	467	37.23	35.73	36.35	110	2	CON	EXERCISE2	40	50
	468	37.23	36.18	36.35	105	2	CON	EXERCISE2	40	50
	469	37.23	36.42	36.36	107	2	CON	EXERCISE2	40	50
	470	37.24	36.52	36.36	111	2	CON	EXERCISE2	40	50
	471	37.25	36.60	36.34	110	2	CON	EXERCISE2	40	50
	472	37.24	36.62	36.37	111	2	CON	EXERCISE2	40	50
	473	37.24	36.63	36.41	106	2	CON	EXERCISE2	40	50
	474	37.24	36.65	36.42	109	2	CON	EXERCISE2	40	50
	475	37.24	36.69	36.45	110	2	CON	EXERCISE2	40	50
	476	37.24	36.71	36.48	107	2	CON	EXERCISE2	40	50
	477	37.25	36.71	36.49	113	2	CON	EXERCISE2	40	50
	478	37.24	36.77	36.52	112	2	CON	EXERCISE2	40	50
	479	37.24	36.76	36.54	111	2	CON	EXERCISE2	40	50
	480	37.24	36.73	36.55	108	2	CON	EXERCISE2	40	50
80	481	37.25	36.66	36.57	109	2	CON	EXERCISE2	40	50
	482	37.26	36.20	36.59	115	2	CON	EXERCISE2	40	50
	483	37.26	36.04	36.60	113	2	CON	EXERCISE2	40	50
	484	37.25	36.34	36.61	113	2	CON	EXERCISE2	40	50
	485	37.24	36.49	36.61	111	2	CON	EXERCISE2	40	50
	486	37.24	36.58	36.61	111	2	CON	EXERCISE2	40	50
	487	37.25	36.65	36.63	114	2	CON	EXERCISE2	40	50

min	number	Tre	Tes	Tsk-head	HR	ID	condition	period	Ta	RH
	488	37.26	36.71	36.65	114	2	CON	EXERCISE2	40	50
	489	37.26	36.76	36.66	114	2	CON	EXERCISE2	40	50
	490	37.25	36.81	36.68	112	2	CON	EXERCISE2	40	50
	491	37.24	36.85	36.69	116	2	CON	EXERCISE2	40	50
	492	37.24	36.86	36.70	119	2	CON	EXERCISE2	40	50
	493	37.25	36.90	36.71	115	2	CON	EXERCISE2	40	50
	494	37.25	36.96	36.71	112	2	CON	EXERCISE2	40	50
	495	37.24	36.95	36.71	116	2	CON	EXERCISE2	40	50
	496	37.25	36.97	36.72	116	2	CON	EXERCISE2	40	50
	497	37.26	37.01	36.72	115	2	CON	EXERCISE2	40	50
	498	37.26	37.04	36.73	114	2	CON	EXERCISE2	40	50
	499	37.28	37.07	36.75	117	2	CON	EXERCISE2	40	50
	500	37.28	37.09	36.76	115	2	CON	EXERCISE2	40	50
	501	37.28	37.09	36.77	112	2	CON	EXERCISE2	40	50
	502	37.28	37.09	36.77	111	2	CON	EXERCISE2	40	50
	503	37.28	37.10	36.78	111	2	CON	EXERCISE2	40	50
	504	37.28	37.12	36.77	114	2	CON	EXERCISE2	40	50
	505	37.28	37.14	36.76	117	2	CON	EXERCISE2	40	50
	506	37.29	37.18	36.78	117	2	CON	EXERCISE2	40	50
	507	37.29	37.19	36.82	117	2	CON	EXERCISE2	40	50
	508	37.29	37.19	36.82	117	2	CON	EXERCISE2	40	50
	509	37.29	37.15	36.82	119	2	CON	EXERCISE2	40	50
	510	37.29	37.14	36.82	118	2	CON	EXERCISE2	40	50
85	511	37.29	37.19	36.83	119	2	CON	EXERCISE2	40	50
	512	37.29	37.20	36.84	117	2	CON	EXERCISE2	40	50
	513	37.30	37.20	36.85	119	2	CON	EXERCISE2	40	50
	514	37.31	37.20	36.88	119	2	CON	EXERCISE2	40	50
	515	37.31	37.21	36.90	116	2	CON	EXERCISE2	40	50
	516	37.31	37.22	36.91	116	2	CON	EXERCISE2	40	50
	517	37.31	37.24	36.91	116	2	CON	EXERCISE2	40	50
	518	37.32	37.25	36.91	114	2	CON	EXERCISE2	40	50
	519	37.32	37.24	36.91	118	2	CON	EXERCISE2	40	50
	520	37.31	37.22	36.91	119	2	CON	EXERCISE2	40	50
	521	37.31	37.22	36.93	120	2	CON	EXERCISE2	40	50
	522	37.32	37.27	36.95	122	2	CON	EXERCISE2	40	50
	523	37.32	37.28	36.94	121	2	CON	EXERCISE2	40	50
	524	37.32	36.92	36.92	120	2	CON	EXERCISE2	40	50
	525	37.32	36.74	36.92	124	2	CON	EXERCISE2	40	50
	526	37.32	36.98	36.92	119	2	CON	EXERCISE2	40	50
	527	37.32	37.07	36.93	119	2	CON	EXERCISE2	40	50
	528	37.32	37.11	36.93	118	2	CON	EXERCISE2	40	50
	529	37.33	37.15	36.95	121	2	CON	EXERCISE2	40	50
	530	37.33	37.20	36.96	122	2	CON	EXERCISE2	40	50
	531	37.33	37.22	36.95	124	2	CON	EXERCISE2	40	50
	532	37.34	37.24	36.95	124	2	CON	EXERCISE2	40	50
	533	37.34	37.27	36.95	122	2	CON	EXERCISE2	40	50

min	number	Tre	Tes	Tsk-head	HR	ID	condition	period	Ta	RH
	534	37.35	37.29	36.95	121	2	CON	EXERCISE2	40	50
	535	37.35	37.29	36.95	125	2	CON	EXERCISE2	40	50
	536	37.35	37.29	36.94	125	2	CON	EXERCISE2	40	50
	537	37.37	37.30	36.96	124	2	CON	EXERCISE2	40	50
	538	37.37	37.35	36.98	123	2	CON	EXERCISE2	40	50
	539	37.37	37.27	36.97	128	2	CON	EXERCISE2	40	50
	540	37.37	37.24	36.98	124	2	CON	EXERCISE2	40	50
90	541	37.37	37.34	36.99	123	2	CON	EXERCISE2	40	50
	542	37.36	37.37	36.99	121	2	CON	EXERCISE2	40	50
	543	37.36	37.37	37.00	118	2	CON	EXERCISE2	40	50
	544	37.37	37.41	37.00	120	2	CON	EXERCISE2	40	50
	545	37.37	37.43	37.00	122	2	CON	EXERCISE2	40	50
	546	37.38	37.44	37.02	120	2	CON	EXERCISE2	40	50
	547	37.38	37.47	37.04	123	2	CON	EXERCISE2	40	50
	548	37.38	37.47	37.05	123	2	CON	EXERCISE2	40	50
	549	37.39	37.48	37.05	124	2	CON	EXERCISE2	40	50
	550	37.40	37.48	37.05	123	2	CON	EXERCISE2	40	50
	551	37.40	37.46	37.07	123	2	CON	EXERCISE2	40	50
	552	37.40	37.42	37.07	125	2	CON	EXERCISE2	40	50
	553	37.40	37.44	37.07	119	2	CON	EXERCISE2	40	50
	554	37.42	37.48	37.08	119	2	CON	EXERCISE2	40	50
	555	37.42	37.48	37.06	125	2	CON	EXERCISE2	40	50
	556	37.41	37.50	37.04	124	2	CON	EXERCISE2	40	50
	557	37.41	37.28	37.06	123	2	CON	EXERCISE2	40	50
	558	37.41	37.26	37.08	127	2	CON	EXERCISE2	40	50
	559	37.41	37.46	37.08	126	2	CON	EXERCISE2	40	50
	560	37.42	37.46	37.08	122	2	CON	EXERCISE2	40	50
	561	37.43	37.48	37.08	121	2	CON	EXERCISE2	40	50
	562	37.43	37.48	37.08	123	2	CON	EXERCISE2	40	50
	563	37.43	37.50	37.09	122	2	CON	EXERCISE2	40	50
	564	37.43	37.53	37.10	120	2	CON	EXERCISE2	40	50
	565	37.44	37.55	37.10	122	2	CON	EXERCISE2	40	50
	566	37.44	37.55	37.09	125	2	CON	EXERCISE2	40	50
	567	37.44	37.56	37.08	124	2	CON	EXERCISE2	40	50
	568	37.45	37.57	37.09	123	2	CON	EXERCISE2	40	50
	569	37.45	37.57	37.11	126	2	CON	EXERCISE2	40	50
	570	37.45	37.59	37.11	127	2	CON	EXERCISE2	40	50
95	571	37.46	37.60	37.11	126	2	CON	EXERCISE2	40	50
	572	37.46	37.61	37.12	128	2	CON	EXERCISE2	40	50
	573	37.47	37.63	37.14	126	2	CON	EXERCISE2	40	50
	574	37.48	37.63	37.16	127	2	CON	EXERCISE2	40	50
	575	37.48	37.65	37.17	131	2	CON	EXERCISE2	40	50
	576	37.48	37.65	37.15	129	2	CON	EXERCISE2	40	50
	577	37.48	37.64	37.14	128	2	CON	EXERCISE2	40	50
	578	37.48	37.66	37.14	122	2	CON	EXERCISE2	40	50
	579	37.49	37.67	37.14	120	2	CON	EXERCISE2	40	50

min	number	Tre	Tes	Tsk-head	HR	ID	condition	period	Ta	RH
	580	37.51	37.67	37.15	125	2	CON	EXERCISE2	40	50
	581	37.51	37.69	37.16	126	2	CON	EXERCISE2	40	50
	582	37.52	37.72	37.17	127	2	CON	EXERCISE2	40	50
	583	37.53	37.71	37.16	129	2	CON	EXERCISE2	40	50
	584	37.53	37.70	37.15	128	2	CON	EXERCISE2	40	50
	585	37.53	37.72	37.15	128	2	CON	EXERCISE2	40	50
	586	37.55	37.74	37.17	132	2	CON	EXERCISE2	40	50
	587	37.55	37.69	37.21	128	2	CON	EXERCISE2	40	50
	588	37.55	37.68	37.22	126	2	CON	EXERCISE2	40	50
	589	37.56	37.73	37.23	128	2	CON	EXERCISE2	40	50
	590	37.56	37.76	37.24	129	2	CON	EXERCISE2	40	50
	591	37.57	37.79	37.23	128	2	CON	EXERCISE2	40	50
	592	37.57	37.77	37.22	128	2	CON	EXERCISE2	40	50
	593	37.57	37.78	37.21	128	2	CON	EXERCISE2	40	50
	594	37.58	37.79	37.20	128	2	CON	EXERCISE2	40	50
	595	37.58	37.67	37.21	128	2	CON	EXERCISE2	40	50
	596	37.58	37.53	37.21	131	2	CON	EXERCISE2	40	50
	597	37.59	37.58	37.22	126	2	CON	EXERCISE2	40	50
	598	37.59	37.67	37.22	127	2	CON	EXERCISE2	40	50
	599	37.60	37.69	37.23	123	2	CON	EXERCISE2	40	50
	600	37.60	37.72	37.24	126	2	CON	EXERCISE2	40	50
100	601	37.60	37.75	37.24	127	2	CON	EXERCISE2	40	50
	602	37.61	37.75	37.26	127	2	CON	EXERCISE2	40	50
	603	37.61	37.76	37.27	128	2	CON	EXERCISE2	40	50
	604	37.61	37.80	37.25	129	2	CON	EXERCISE2	40	50
	605	37.61	37.81	37.23	127	2	CON	EXERCISE2	40	50
	606	37.62	37.79	37.23	129	2	CON	EXERCISE2	40	50
	607	37.61	37.72	37.24	132	2	CON	EXERCISE2	40	50
	608	37.61	37.72	37.25	128	2	CON	EXERCISE2	40	50
	609	37.61	37.77	37.26	129	2	CON	EXERCISE2	40	50
	610	37.61	37.79	37.21	129	2	CON	EXERCISE2	40	50
	611	37.61	37.80	37.21	131	2	CON	EXERCISE2	40	50
	612	37.63	37.81	37.27	134	2	CON	EXERCISE2	40	50
	613	37.64	37.83	37.28	135	2	CON	EXERCISE2	40	50
	614	37.64	37.84	37.29	134	2	CON	EXERCISE2	40	50
	615	37.64	37.86	37.27	131	2	CON	EXERCISE2	40	50
	616	37.65	37.87	37.27	133	2	CON	EXERCISE2	40	50
	617	37.65	37.84	37.30	135	2	CON	EXERCISE2	40	50
	618	37.65	37.84	37.31	133	2	CON	EXERCISE2	40	50
	619	37.66	37.88	37.33	134	2	CON	EXERCISE2	40	50
	620	37.66	37.89	37.35	132	2	CON	EXERCISE2	40	50
	621	37.67	37.89	37.36	130	2	CON	EXERCISE2	40	50
	622	37.68	37.91	37.33	131	2	CON	EXERCISE2	40	50
	623	37.68	37.92	37.31	131	2	CON	EXERCISE2	40	50
	624	37.68	37.87	37.33	134	2	CON	EXERCISE2	40	50
	625	37.69	37.85	37.33	135	2	CON	EXERCISE2	40	50

min	number	Tre	Tes	Tsk-head	HR	ID	condition	period	Ta	RH
	626	37.68	37.89	37.25	131	2	CON	EXERCISE2	40	50
	627	37.68	37.91	37.20	131	2	CON	EXERCISE2	40	50
	628	37.69	37.94	37.23	130	2	CON	EXERCISE2	40	50
	629	37.70	37.96	37.26	131	2	CON	EXERCISE2	40	50
	630	37.71	37.96	37.24	129	2	CON	EXERCISE2	40	50
105	631	37.71	37.98	37.22	129	2	CON	EXERCISE2	40	50
	632	37.72	38.01	37.22	130	2	CON	EXERCISE2	40	50
	633	37.72	38.01	37.21	129	2	CON	EXERCISE2	40	50
	634	37.73	38.00	37.22	132	2	CON	EXERCISE2	40	50
	635	37.73	37.99	37.23	129	2	CON	EXERCISE2	40	50
	636	37.73	37.99	37.25	132	2	CON	EXERCISE2	40	50
	637	37.73	38.01	37.25	135	2	CON	EXERCISE2	40	50
	638	37.74	38.05	37.25	136	2	CON	EXERCISE2	40	50
	639	37.74	38.04	37.25	134	2	CON	EXERCISE2	40	50
	640	37.74	38.05	37.23	133	2	CON	EXERCISE2	40	50
	641	37.75	38.06	37.22	134	2	CON	EXERCISE2	40	50
	642	37.76	38.00	37.21	137	2	CON	EXERCISE2	40	50
	643	37.76	37.97	37.21	134	2	CON	EXERCISE2	40	50
	644	37.75	38.02	37.25	129	2	CON	REST3	28	50
	645	37.75	38.05	37.23	137	2	CON	REST3	28	50
	646	37.74	38.05	36.71	131	2	CON	REST3	28	50
	647	37.74	38.04	36.20	130	2	CON	REST3	28	50
	648	37.74	38.06	36.13	120	2	CON	REST3	28	50
	649	37.76	38.10	36.15	116	2	CON	REST3	28	50
	650	37.76	38.11	36.13	114	2	CON	REST3	28	50
	651	37.76	38.08	36.01	111	2	CON	REST3	28	50
	652	37.76	38.06	35.96	133	2	CON	REST3	28	50
	653	37.77	38.09	36.04	113	2	CON	REST3	28	50
	654	37.78	38.13	36.17	108	2	CON	REST3	28	50
	655	37.79	38.13	36.18	109	2	CON	REST3	28	50
	656	37.79	38.12	36.10	112	2	CON	REST3	28	50
	657	37.80	38.14	36.06	109	2	CON	REST3	28	50
	658	37.79	38.15	36.01	111	2	CON	REST3	28	50
	659	37.80	38.15	35.93	110	2	CON	REST3	28	50
	660	37.81	38.13	35.85	108	2	CON	REST3	28	50
110	661	37.81	38.10	35.78	106	2	CON	REST3	28	50
	662	37.80	38.10	35.71	109	2	CON	REST3	28	50
	663	37.80	38.08	35.66	107	2	CON	REST3	28	50
	664	37.80	38.02	35.66	107	2	CON	REST3	28	50
	665	37.80	38.05	35.64	107	2	CON	REST3	28	50
	666	37.81	38.09	35.54	107	2	CON	REST3	28	50
	667	37.80	38.10	35.40	109	2	CON	REST3	28	50
	668	37.80	38.10	35.34	106	2	CON	REST3	28	50
	669	37.81	38.09	35.41	106	2	CON	REST3	28	50
	670	37.82	38.07	35.30	105	2	CON	REST3	28	50
	671	37.81	38.02	35.25	107	2	CON	REST3	28	50

min	number	Tre	Tes	Tsk-head	HR	ID	condition	period	Ta	RH
	672	37.80	37.99	35.22	106	2	CON	REST3	28	50
	673	37.80	37.98	34.99	107	2	CON	REST3	28	50
	674	37.81	37.98	34.93	107	2	CON	REST3	28	50
	675	37.82	37.99	34.96	105	2	CON	REST3	28	50
	676	37.82	37.97	34.90	105	2	CON	REST3	28	50
	677	37.81	37.88	34.88	111	2	CON	REST3	28	50
	678	37.82	37.81	34.99	104	2	CON	REST3	28	50
	679	37.81	37.79	34.98	102	2	CON	REST3	28	50
	680	37.80	37.79	34.86	103	2	CON	REST3	28	50
	681	37.80	37.81	34.79	100	2	CON	REST3	28	50
	682	37.81	37.81	34.78	101	2	CON	REST3	28	50
	683	37.80	37.79	34.77	100	2	CON	REST3	28	50
	684	37.80	37.78	34.72	101	2	CON	REST3	28	50
	685	37.80	37.81	34.68	100	2	CON	REST3	28	50
	686	37.80	37.80	34.78	102	2	CON	REST3	28	50
	687	37.80	37.79	34.86	97	2	CON	REST3	28	50
	688	37.78	37.80	34.85	94	2	CON	REST3	28	50
	689	37.77	37.82	34.86	99	2	CON	REST3	28	50
	690	37.78	37.71	35.04	115	2	CON	REST3	28	50
115	691	37.77	37.66	35.25	81	2	CON	REST3	28	50
	692	37.77	37.71	35.23	103	2	CON	REST3	28	50
	693	37.77	37.66	35.04	101	2	CON	REST3	28	50
	694	37.77	37.63	34.90	98	2	CON	REST3	28	50
	695	37.77	37.65	34.92	99	2	CON	REST3	28	50
	696	37.77	37.55	35.04	107	2	CON	REST3	28	50
	697	37.78	37.53	35.19	101	2	CON	REST3	28	50
	698	37.78	37.63	35.19	101	2	CON	REST3	28	50
	699	37.77	37.64	35.06	100	2	CON	REST3	28	50
	700	37.76	37.64	34.95	105	2	CON	REST3	28	50
	701	37.76	37.59	34.96	101	2	CON	REST3	28	50
	702	37.76	37.57	34.99	97	2	CON	REST3	28	50
	703	37.75	37.59	34.92	95	2	CON	REST3	28	50
	704	37.75	37.60	34.88	97	2	CON	REST3	28	50
	705	37.74	37.63	34.82	98	2	CON	REST3	28	50
	706	37.74	37.62	34.85	100	2	CON	REST3	28	50
	707	37.75	37.59	34.90	98	2	CON	REST3	28	50
	708	37.75	37.61	34.98	102	2	CON	REST3	28	50
	709	37.75	37.64	35.03	101	2	CON	REST3	28	50
0	1	36.93	36.96	35.59	83	3	CON	REST1	28	50
	2	36.92	36.96	35.56	88	3	CON	REST1	28	50
	3	36.92	36.98	35.54	91	3	CON	REST1	28	50
	4	36.92	36.97	35.58	78	3	CON	REST1	28	50
	5	36.91	36.97	35.60	76	3	CON	REST1	28	50
	6	36.91	36.96	35.61	92	3	CON	REST1	28	50
	7	36.91	36.97	35.61	82	3	CON	REST1	28	50
	8	36.91	36.97	35.60	79	3	CON	REST1	28	50

min	number	Tre	Tes	Tsk-head	HR	ID	condition	period	Ta	RH
	9	36.91	36.96	35.60	80	3	CON	REST1	28	50
	10	36.91	36.97	35.61	90	3	CON	REST1	28	50
	11	36.92	36.97	35.62	95	3	CON	REST1	28	50
	12	36.92	36.97	35.62	91	3	CON	REST1	28	50
	13	36.91	36.96	35.62	83	3	CON	REST1	28	50
	14	36.91	36.95	35.65	80	3	CON	REST1	28	50
	15	36.91	36.96	35.66	85	3	CON	REST1	28	50
	16	36.90	36.97	35.64	82	3	CON	REST1	28	50
	17	36.89	36.96	35.63	85	3	CON	REST1	28	50
	18	36.90	36.97	35.62	80	3	CON	REST1	28	50
	19	36.91	36.98	35.62	77	3	CON	REST1	28	50
	20	36.91	36.98	35.63	77	3	CON	REST1	28	50
	21	36.91	36.98	35.64	81	3	CON	REST1	28	50
	22	36.91	36.98	35.67	85	3	CON	REST1	28	50
	23	36.91	36.97	35.68	80	3	CON	REST1	28	50
	24	36.91	36.97	35.67	86	3	CON	REST1	28	50
	25	36.90	36.97	35.68	87	3	CON	REST1	28	50
	26	36.90	36.97	35.68	80	3	CON	REST1	28	50
	27	36.90	36.97	35.69	76	3	CON	REST1	28	50
	28	36.90	36.98	35.69	79	3	CON	REST1	28	50
	29	36.90	36.96	35.69	80	3	CON	REST1	28	50
	30	36.90	36.96	35.71	80	3	CON	REST1	28	50
5	31	36.91	36.98	35.69	82	3	CON	REST1	28	50
	32	36.90	36.98	35.66	84	3	CON	REST1	28	50
	33	36.91	37.00	35.67	84	3	CON	REST1	28	50
	34	36.91	37.01	35.70	79	3	CON	REST1	28	50
	35	36.91	36.99	35.71	78	3	CON	REST1	28	50
	36	36.91	36.98	35.72	82	3	CON	REST1	28	50
	37	36.90	36.97	35.73	82	3	CON	REST1	28	50
	38	36.90	36.98	35.74	80	3	CON	REST1	28	50
	39	36.90	36.99	35.75	82	3	CON	REST1	28	50
	40	36.89	36.99	35.75	91	3	CON	REST1	28	50
	41	36.89	36.99	35.73	92	3	CON	REST1	28	50
	42	36.89	36.99	35.71	87	3	CON	REST1	28	50
	43	36.89	37.01	35.70	86	3	CON	REST1	28	50
	44	36.88	36.98	35.73	89	3	CON	REST1	28	50
	45	36.89	36.96	35.77	84	3	CON	REST1	28	50
	46	36.89	36.95	35.76	86	3	CON	REST1	28	50
	47	36.88	36.93	35.77	86	3	CON	REST1	28	50
	48	36.89	36.95	35.77	94	3	CON	REST1	28	50
	49	36.88	36.96	35.75	81	3	CON	REST1	28	50
	50	36.88	36.94	35.76	86	3	CON	REST1	28	50
	51	36.88	36.93	35.75	85	3	CON	REST1	28	50
	52	36.88	36.92	35.73	78	3	CON	REST1	28	50
	53	36.88	36.93	35.73	79	3	CON	REST1	28	50
	54	36.89	36.93	35.75	80	3	CON	REST1	28	50

min	number	Tre	Tes	Tsk-head	HR	ID	condition	period	Ta	RH
	55	36.88	36.91	35.76	84	3	CON	REST1	28	50
	56	36.88	36.93	35.75	83	3	CON	REST1	28	50
	57	36.88	36.93	35.74	83	3	CON	REST1	28	50
	58	36.87	36.94	35.74	81	3	CON	REST1	28	50
	59	36.88	36.95	35.77	80	3	CON	REST1	28	50
	60	36.88	36.94	35.80	81	3	CON	REST1	28	50
10	61	36.88	36.94	35.81	89	3	CON	REST1	28	50
	62	36.88	36.95	35.82	89	3	CON	REST1	28	50
	63	36.89	36.94	35.81	86	3	CON	REST1	28	50
	64	36.89	36.94	35.81	85	3	CON	REST1	28	50
	65	36.88	36.93	35.83	88	3	CON	REST1	28	50
	66	36.86	36.92	35.83	83	3	CON	REST1	28	50
	67	36.85	36.92	35.80	79	3	CON	REST1	28	50
	68	36.85	36.92	35.79	77	3	CON	REST1	28	50
	69	36.86	36.93	35.79	86	3	CON	REST1	28	50
	70	36.85	36.93	35.78	80	3	CON	REST1	28	50
	71	36.85	36.92	35.78	82	3	CON	REST1	28	50
	72	36.85	36.93	35.78	77	3	CON	REST1	28	50
	73	36.85	36.95	35.77	77	3	CON	REST1	28	50
	74	36.85	36.94	35.76	82	3	CON	REST1	28	50
	75	36.85	36.92	35.76	82	3	CON	REST1	28	50
	76	36.86	36.92	35.76	83	3	CON	REST1	28	50
	77	36.86	36.92	35.76	81	3	CON	REST1	28	50
	78	36.85	36.91	35.77	87	3	CON	REST1	28	50
	79	36.86	36.90	35.78	84	3	CON	REST1	28	50
	80	36.87	36.93	35.79	83	3	CON	REST1	28	50
	81	36.87	36.93	35.77	91	3	CON	REST1	28	50
	82	36.86	36.92	35.75	83	3	CON	REST1	28	50
	83	36.86	36.94	35.74	80	3	CON	REST1	28	50
	84	36.86	36.95	35.74	82	3	CON	REST1	28	50
	85	36.86	36.95	35.75	86	3	CON	REST1	28	50
	86	36.86	36.91	35.77	88	3	CON	REST1	28	50
	87	36.87	36.90	35.78	81	3	CON	REST1	28	50
	88	36.87	36.89	35.77	85	3	CON	REST1	28	50
	89	36.86	36.87	35.78	91	3	CON	REST1	28	50
	90	36.86	36.89	35.80	80	3	CON	REST1	28	50
15	91	36.85	36.91	35.78	82	3	CON	REST1	28	50
	92	36.84	36.91	35.75	84	3	CON	REST1	28	50
	93	36.84	36.89	35.74	78	3	CON	REST1	28	50
	94	36.83	36.89	35.74	80	3	CON	REST1	28	50
	95	36.83	36.92	35.73	88	3	CON	REST1	28	50
	96	36.83	36.93	35.73	79	3	CON	REST1	28	50
	97	36.83	36.90	35.74	88	3	CON	REST1	28	50
	98	36.81	36.89	35.75	89	3	CON	REST1	28	50
	99	36.81	36.88	35.75	88	3	CON	REST1	28	50
	100	36.82	36.90	35.77	86	3	CON	REST1	28	50

min	number	Tre	Tes	Tsk-head	HR	ID	condition	period	Ta	RH
	101	36.82	36.90	35.78	83	3	CON	REST1	28	50
	102	36.81	36.86	35.78	91	3	CON	REST1	28	50
	103	36.81	36.87	35.80	84	3	CON	REST1	40	50
	104	36.82	36.82	35.92	94	3	CON	REST1	40	50
	105	36.83	36.82	36.15	92	3	CON	REST1	40	50
	106	36.82	36.86	36.31	95	3	CON	REST1	40	50
	107	36.81	36.88	36.40	95	3	CON	REST1	40	50
	108	36.81	36.91	36.47	89	3	CON	REST1	40	50
	109	36.81	36.91	36.53	95	3	CON	REST1	40	50
	110	36.81	36.91	36.61	90	3	CON	REST1	40	50
	111	36.80	36.95	36.65	90	3	CON	REST1	40	50
	112	36.81	37.02	36.66	83	3	CON	REST1	40	50
	113	36.82	36.96	36.69	89	3	CON	REST1	40	50
	114	36.82	36.89	36.71	89	3	CON	REST1	40	50
	115	36.82	36.84	36.73	89	3	CON	REST1	40	50
	116	36.82	36.81	36.74	84	3	CON	REST1	40	50
	117	36.82	36.83	36.76	83	3	CON	REST1	40	50
	118	36.82	36.85	36.77	86	3	CON	REST1	40	50
	119	36.83	36.84	36.79	95	3	CON	REST1	40	50
	120	36.83	36.82	36.80	86	3	CON	REST1	40	50
20	121	36.83	36.82	36.81	88	3	CON	REST1	40	50
	122	36.84	36.83	36.82	85	3	CON	REST1	40	50
	123	36.85	36.84	36.83	87	3	CON	REST1	40	50
	124	36.85	36.84	36.84	87	3	CON	REST1	40	50
	125	36.86	36.84	36.86	86	3	CON	REST1	40	50
	126	36.87	36.83	36.88	81	3	CON	REST1	40	50
	127	36.86	36.82	36.89	89	3	CON	REST1	40	50
	128	36.87	36.83	36.91	87	3	CON	REST1	40	50
	129	36.88	36.84	36.93	90	3	CON	REST1	40	50
	130	36.88	36.83	36.94	84	3	CON	REST1	40	50
	131	36.89	36.83	36.95	79	3	CON	REST1	40	50
	132	36.88	36.84	36.96	83	3	CON	REST1	40	50
	133	36.88	36.84	36.96	88	3	CON	REST1	40	50
	134	36.88	36.83	36.96	90	3	CON	REST1	40	50
	135	36.87	36.83	36.95	102	3	CON	REST1	40	50
	136	36.86	36.82	36.97	93	3	CON	REST1	40	50
	137	36.84	36.84	36.98	91	3	CON	REST1	40	50
	138	36.83	36.83	36.99	88	3	CON	REST1	40	50
	139	36.82	36.83	36.99	102	3	CON	EXERCISE1	40	50
	140	36.82	36.83	36.99	93	3	CON	EXERCISE1	40	50
	141	36.82	36.82	36.99	105	3	CON	EXERCISE1	40	50
	142	36.82	36.80	36.99	105	3	CON	EXERCISE1	40	50
	143	36.82	36.81	37.00	108	3	CON	EXERCISE1	40	50
	144	36.81	36.83	37.00	111	3	CON	EXERCISE1	40	50
	145	36.80	36.81	37.00	112	3	CON	EXERCISE1	40	50
	146	36.80	36.81	37.00	115	3	CON	EXERCISE1	40	50

min	number	Tre	Tes	Tsk-head	HR	ID	condition	period	Ta	RH
	147	36.80	36.81	37.00	115	3	CON	EXERCISE1	40	50
	148	36.80	36.80	37.00	115	3	CON	EXERCISE1	40	50
	149	36.80	36.79	37.01	115	3	CON	EXERCISE1	40	50
	150	36.81	36.80	37.01	117	3	CON	EXERCISE1	40	50
25	151	36.81	36.81	37.00	118	3	CON	EXERCISE1	40	50
	152	36.81	36.82	37.00	117	3	CON	EXERCISE1	40	50
	153	36.81	36.81	37.00	120	3	CON	EXERCISE1	40	50
	154	36.81	36.82	37.00	123	3	CON	EXERCISE1	40	50
	155	36.81	36.83	37.01	123	3	CON	EXERCISE1	40	50
	156	36.80	36.82	37.00	123	3	CON	EXERCISE1	40	50
	157	36.80	36.84	36.99	123	3	CON	EXERCISE1	40	50
	158	36.80	36.86	36.99	124	3	CON	EXERCISE1	40	50
	159	36.80	36.87	37.00	124	3	CON	EXERCISE1	40	50
	160	36.81	36.87	37.00	128	3	CON	EXERCISE1	40	50
	161	36.81	36.87	37.00	127	3	CON	EXERCISE1	40	50
	162	36.81	36.90	37.00	125	3	CON	EXERCISE1	40	50
	163	36.81	36.90	37.00	128	3	CON	EXERCISE1	40	50
	164	36.80	36.90	37.01	127	3	CON	EXERCISE1	40	50
	165	36.81	36.93	37.01	128	3	CON	EXERCISE1	40	50
	166	36.81	36.96	37.00	129	3	CON	EXERCISE1	40	50
	167	36.81	36.98	37.00	127	3	CON	EXERCISE1	40	50
	168	36.81	36.98	37.02	125	3	CON	EXERCISE1	40	50
	169	36.82	36.99	37.03	126	3	CON	EXERCISE1	40	50
	170	36.83	37.03	37.04	126	3	CON	EXERCISE1	40	50
	171	36.84	37.03	37.05	125	3	CON	EXERCISE1	40	50
	172	36.84	37.03	37.06	129	3	CON	EXERCISE1	40	50
	173	36.84	37.04	37.06	131	3	CON	EXERCISE1	40	50
	174	36.84	37.04	37.07	128	3	CON	EXERCISE1	40	50
	175	36.84	37.06	37.08	126	3	CON	EXERCISE1	40	50
	176	36.84	37.08	37.09	128	3	CON	EXERCISE1	40	50
	177	36.84	37.10	37.10	129	3	CON	EXERCISE1	40	50
	178	36.84	37.10	37.10	128	3	CON	EXERCISE1	40	50
	179	36.84	37.12	37.12	127	3	CON	EXERCISE1	40	50
	180	36.85	37.13	37.13	124	3	CON	EXERCISE1	40	50
30	181	36.85	37.14	37.14	128	3	CON	EXERCISE1	40	50
	182	36.86	37.15	37.16	131	3	CON	EXERCISE1	40	50
	183	36.88	37.16	37.16	131	3	CON	EXERCISE1	40	50
	184	36.89	37.16	37.15	128	3	CON	EXERCISE1	40	50
	185	36.90	37.14	37.16	136	3	CON	EXERCISE1	40	50
	186	36.90	37.14	37.16	134	3	CON	EXERCISE1	40	50
	187	36.90	37.17	37.17	131	3	CON	EXERCISE1	40	50
	188	36.89	37.17	37.18	130	3	CON	EXERCISE1	40	50
	189	36.88	37.16	37.19	130	3	CON	EXERCISE1	40	50
	190	36.86	37.15	37.19	132	3	CON	EXERCISE1	40	50
	191	36.86	37.15	37.19	136	3	CON	EXERCISE1	40	50
	192	36.87	37.15	37.19	133	3	CON	EXERCISE1	40	50

min	number	Tre	Tes	Tsk-head	HR	ID	condition	period	Ta	RH
	193	36.86	37.16	37.21	128	3	CON	EXERCISE1	40	50
	194	36.85	37.16	37.23	128	3	CON	EXERCISE1	40	50
	195	36.84	37.17	37.24	127	3	CON	EXERCISE1	40	50
	196	36.83	37.17	37.23	130	3	CON	EXERCISE1	40	50
	197	36.83	37.19	37.24	134	3	CON	EXERCISE1	40	50
	198	36.83	37.21	37.24	134	3	CON	EXERCISE1	40	50
	199	36.82	37.22	37.22	138	3	CON	EXERCISE1	40	50
	200	36.82	37.22	37.22	134	3	CON	EXERCISE1	40	50
	201	36.81	37.23	37.22	137	3	CON	EXERCISE1	40	50
	202	36.81	37.21	37.23	134	3	CON	EXERCISE1	40	50
	203	36.81	37.21	37.23	137	3	CON	EXERCISE1	40	50
	204	36.81	37.21	37.23	135	3	CON	EXERCISE1	40	50
	205	36.82	37.22	37.24	137	3	CON	EXERCISE1	40	50
	206	36.82	37.25	37.24	134	3	CON	EXERCISE1	40	50
	207	36.83	37.28	37.25	137	3	CON	EXERCISE1	40	50
	208	36.83	37.28	37.24	139	3	CON	EXERCISE1	40	50
	209	36.83	37.27	37.24	136	3	CON	EXERCISE1	40	50
	210	36.83	37.26	37.26	137	3	CON	EXERCISE1	40	50
35	211	36.84	37.25	37.26	139	3	CON	EXERCISE1	40	50
	212	36.85	37.26	37.26	140	3	CON	EXERCISE1	40	50
	213	36.85	37.27	37.26	140	3	CON	EXERCISE1	40	50
	214	36.85	37.26	37.25	139	3	CON	EXERCISE1	40	50
	215	36.86	37.27	37.24	137	3	CON	EXERCISE1	40	50
	216	36.87	37.31	37.25	141	3	CON	EXERCISE1	40	50
	217	36.86	37.31	37.26	140	3	CON	EXERCISE1	40	50
	218	36.85	37.31	37.27	140	3	CON	EXERCISE1	40	50
	219	36.85	37.30	37.29	162	3	CON	EXERCISE1	40	50
	220	36.86	37.30	37.29	140	3	CON	EXERCISE1	40	50
	221	36.86	37.31	37.29	142	3	CON	EXERCISE1	40	50
	222	36.86	37.32	37.30	143	3	CON	EXERCISE1	40	50
	223	36.87	37.34	37.30	143	3	CON	EXERCISE1	40	50
	224	36.88	37.35	37.31	143	3	CON	EXERCISE1	40	50
	225	36.88	37.35	37.31	145	3	CON	EXERCISE1	40	50
	226	36.89	37.35	37.31	163	3	CON	EXERCISE1	40	50
	227	36.89	37.36	37.32	141	3	CON	EXERCISE1	40	50
	228	36.89	37.38	37.34	142	3	CON	EXERCISE1	40	50
	229	36.90	37.36	37.35	145	3	CON	EXERCISE1	40	50
	230	36.90	37.36	37.35	145	3	CON	EXERCISE1	40	50
	231	36.90	37.40	37.36	143	3	CON	EXERCISE1	40	50
	232	36.90	37.40	37.37	147	3	CON	EXERCISE1	40	50
	233	36.90	37.39	37.38	143	3	CON	EXERCISE1	40	50
	234	36.90	37.41	37.39	143	3	CON	EXERCISE1	40	50
	235	36.91	37.43	37.40	143	3	CON	EXERCISE1	40	50
	236	36.91	37.43	37.40	146	3	CON	EXERCISE1	40	50
	237	36.92	37.43	37.41	143	3	CON	EXERCISE1	40	50
	238	36.92	37.44	37.41	145	3	CON	EXERCISE1	40	50

min	number	Tre	Tes	Tsk-head	HR	ID	condition	period	Ta	RH
	239	36.92	37.45	37.39	145	3	CON	EXERCISE1	40	50
	240	36.92	37.46	37.40	143	3	CON	EXERCISE1	40	50
40	241	36.93	37.47	37.43	144	3	CON	EXERCISE1	40	50
	242	36.95	37.47	37.45	143	3	CON	EXERCISE1	40	50
	243	36.96	37.48	37.46	144	3	CON	EXERCISE1	40	50
	244	36.97	37.50	37.46	145	3	CON	EXERCISE1	40	50
	245	36.96	37.49	37.48	140	3	CON	EXERCISE1	40	50
	246	36.95	37.49	37.47	147	3	CON	EXERCISE1	40	50
	247	36.95	37.50	37.46	147	3	CON	EXERCISE1	40	50
	248	36.95	37.50	37.47	145	3	CON	EXERCISE1	40	50
	249	36.96	37.49	37.48	145	3	CON	EXERCISE1	40	50
	250	36.97	37.49	37.49	148	3	CON	EXERCISE1	40	50
	251	36.98	37.50	37.50	147	3	CON	EXERCISE1	40	50
	252	36.99	37.50	37.51	145	3	CON	EXERCISE1	40	50
	253	36.98	37.52	37.51	147	3	CON	EXERCISE1	40	50
	254	36.98	37.55	37.50	146	3	CON	EXERCISE1	40	50
	255	36.99	37.54	37.51	150	3	CON	EXERCISE1	40	50
	256	37.00	37.55	37.52	147	3	CON	EXERCISE1	40	50
	257	37.01	37.55	37.51	150	3	CON	EXERCISE1	40	50
	258	37.01	37.52	37.52	153	3	CON	EXERCISE1	40	50
	259	37.02	37.51	37.53	153	3	CON	EXERCISE1	40	50
	260	37.03	37.55	37.52	153	3	CON	EXERCISE1	40	50
	261	37.04	37.56	37.52	157	3	CON	EXERCISE1	40	50
	262	37.05	37.55	37.52	157	3	CON	EXERCISE1	40	50
	263	37.05	37.59	37.52	179	3	CON	EXERCISE1	40	50
	264	37.05	37.61	37.51	157	3	CON	EXERCISE1	40	50
	265	37.06	37.60	37.53	157	3	CON	EXERCISE1	40	50
	266	37.07	37.59	37.55	152	3	CON	EXERCISE1	40	50
	267	37.07	37.60	37.56	153	3	CON	EXERCISE1	40	50
	268	37.08	37.62	37.59	153	3	CON	EXERCISE1	40	50
	269	37.08	37.66	37.59	150	3	CON	EXERCISE1	40	50
	270	37.07	37.66	37.59	151	3	CON	EXERCISE1	40	50
45	271	37.08	37.67	37.61	153	3	CON	EXERCISE1	40	50
	272	37.09	37.69	37.61	153	3	CON	EXERCISE1	40	50
	273	37.09	37.68	37.62	150	3	CON	EXERCISE1	40	50
	274	37.10	37.65	37.61	151	3	CON	EXERCISE1	40	50
	275	37.11	37.65	37.58	153	3	CON	EXERCISE1	40	50
	276	37.12	37.69	37.55	153	3	CON	EXERCISE1	40	50
	277	37.11	37.73	37.54	147	3	CON	EXERCISE1	40	50
	278	37.11	37.77	37.55	150	3	CON	EXERCISE1	40	50
	279	37.12	37.75	37.54	150	3	CON	EXERCISE1	40	50
	280	37.13	37.71	37.54	153	3	CON	EXERCISE1	40	50
	281	37.13	37.68	37.56	151	3	CON	EXERCISE1	40	50
	282	37.14	37.70	37.57	149	3	CON	EXERCISE1	40	50
	283	37.15	37.73	37.59	150	3	CON	EXERCISE1	40	50
	284	37.17	37.75	37.61	148	3	CON	EXERCISE1	40	50

min	number	Tre	Tes	Tsk-head	HR	ID	condition	period	Ta	RH
	285	37.18	37.77	37.61	153	3	CON	EXERCISE1	40	50
	286	37.18	37.76	37.59	153	3	CON	EXERCISE1	40	50
	287	37.18	37.77	37.57	154	3	CON	EXERCISE1	40	50
	288	37.19	37.77	37.59	153	3	CON	EXERCISE1	40	50
	289	37.20	37.79	37.63	153	3	CON	EXERCISE1	40	50
	290	37.20	37.82	37.64	150	3	CON	EXERCISE1	40	50
	291	37.21	37.83	37.66	150	3	CON	EXERCISE1	40	50
	292	37.22	37.82	37.68	150	3	CON	EXERCISE1	40	50
	293	37.23	37.84	37.69	150	3	CON	EXERCISE1	40	50
	294	37.24	37.85	37.70	150	3	CON	EXERCISE1	40	50
	295	37.25	37.84	37.70	153	3	CON	EXERCISE1	40	50
	296	37.25	37.84	37.71	150	3	CON	EXERCISE1	40	50
	297	37.26	37.85	37.73	150	3	CON	EXERCISE1	40	50
	298	37.26	37.87	37.73	152	3	CON	EXERCISE1	40	50
	299	37.27	37.88	37.74	153	3	CON	EXERCISE1	40	50
	300	37.27	37.87	37.76	154	3	CON	EXERCISE1	40	50
50	301	37.28	37.88	37.77	153	3	CON	EXERCISE1	40	50
	302	37.31	37.89	37.77	153	3	CON	EXERCISE1	40	50
	303	37.33	37.91	37.77	151	3	CON	EXERCISE1	40	50
	304	37.35	37.92	37.75	157	3	CON	EXERCISE1	40	50
	305	37.37	37.92	37.75	158	3	CON	EXERCISE1	40	50
	306	37.37	37.94	37.75	157	3	CON	EXERCISE1	40	50
	307	37.37	37.94	37.76	157	3	CON	EXERCISE1	40	50
	308	37.38	37.93	37.77	157	3	CON	EXERCISE1	40	50
	309	37.39	37.95	37.78	157	3	CON	EXERCISE1	40	50
	310	37.40	37.96	37.78	157	3	CON	EXERCISE1	40	50
	311	37.40	37.95	37.78	157	3	CON	EXERCISE1	40	50
	312	37.41	37.96	37.78	158	3	CON	EXERCISE1	40	50
	313	37.42	37.96	37.78	157	3	CON	EXERCISE1	40	50
	314	37.42	37.98	37.78	158	3	CON	EXERCISE1	40	50
	315	37.42	37.97	37.77	159	3	CON	EXERCISE1	40	50
	316	37.44	37.99	37.77	157	3	CON	EXERCISE1	40	50
	317	37.44	38.02	37.78	156	3	CON	EXERCISE1	40	50
	318	37.45	38.03	37.78	161	3	CON	EXERCISE1	40	50
	319	37.46	38.04	37.79	161	3	CON	EXERCISE1	40	50
	320	37.46	38.05	37.81	160	3	CON	REST2	28	50
	321	37.48	38.09	37.64	161	3	CON	REST2	28	50
	322	37.49	38.09	37.38	150	3	CON	REST2	28	50
	323	37.49	38.04	37.25	146	3	CON	REST2	28	50
	324	37.50	38.03	37.17	143	3	CON	REST2	28	50
	325	37.50	38.30	37.06	142	3	CON	REST2	28	50
	326	37.50	38.63	36.86	136	3	CON	REST2	28	50
	327	37.50	38.72	36.68	136	3	CON	REST2	28	50
	328	37.50	38.58	36.65	134	3	CON	REST2	28	50
	329	37.51	38.36	36.63	132	3	CON	REST2	28	50
	330	37.51	38.24	36.60	128	3	CON	REST2	28	50

min	number	Tre	Tes	Tsk-head	HR	ID	condition	period	Ta	RH
55	331	37.51	38.19	36.53	126	3	CON	REST2	28	50
	332	37.51	38.17	36.44	124	3	CON	REST2	28	50
	333	37.51	38.13	36.46	134	3	CON	REST2	28	50
	334	37.51	38.06	36.54	135	3	CON	REST2	28	50
	335	37.50	37.92	36.57	140	3	CON	REST2	28	50
	336	37.48	37.86	36.54	137	3	CON	REST2	28	50
	337	37.49	37.84	36.53	137	3	CON	REST2	28	50
	338	37.50	32.52	36.54	136	3	CON	REST2	28	50
	339	37.50	27.62	36.56	143	3	CON	REST2	28	50
	340	37.50	29.03	36.53	134	3	CON	REST2	28	50
	341	37.50	28.82	36.52	129	3	CON	REST2	28	50
	342	37.50	27.42	36.55	131	3	CON	REST2	28	50
	343	37.50	27.17	36.54	125	3	CON	REST2	28	50
	344	37.50	28.01	36.49	125	3	CON	REST2	28	50
	345	37.51	28.34	36.48	126	3	CON	REST2	28	50
	346	37.52	27.51	36.48	122	3	CON	REST2	28	50
	347	37.53	27.61	36.46	123	3	CON	REST2	28	50
	348	37.53	28.48	36.42	125	3	CON	REST2	28	50
	349	37.54	29.90	36.39	115	3	CON	REST2	28	50
	350	37.54	29.64	36.40	116	3	CON	REST2	28	50
	351	37.54	28.36	36.41	115	3	CON	REST2	28	50
	352	37.55	28.28	36.43	113	3	CON	REST2	28	50
	353	37.55	28.84	36.37	112	3	CON	REST2	28	50
	354	37.56	29.65	36.29	112	3	CON	REST2	28	50
	355	37.55	30.99	36.27	100	3	CON	REST2	28	50
	356	37.54	32.44	36.26	105	3	CON	REST2	28	50
	357	37.55	32.81	36.27	105	3	CON	REST2	28	50
	358	37.54	33.20	36.29	104	3	CON	REST2	28	50
	359	37.53	33.92	36.27	106	3	CON	REST2	28	50
	360	37.53	34.53	36.25	103	3	CON	REST2	28	50
60	361	37.53	35.07	36.23	98	3	CON	REST2	28	50
	362	37.52	35.40	36.18	102	3	CON	REST2	28	50
	363	37.53	35.80	36.11	108	3	CON	REST2	28	50
	364	37.54	36.13	36.07	99	3	CON	REST2	28	50
	365	37.55	36.35	36.06	100	3	CON	REST2	28	50
	366	37.54	36.53	36.04	102	3	CON	REST2	28	50
	367	37.53	36.52	36.02	105	3	CON	REST2	28	50
	368	37.52	36.67	35.99	105	3	CON	REST2	28	50
	369	37.52	36.85	35.98	107	3	CON	REST2	28	50
	370	37.52	36.83	36.03	105	3	CON	REST2	28	50
	371	37.52	36.85	36.03	103	3	CON	REST2	28	50
	372	37.52	36.91	36.03	101	3	CON	REST2	28	50
	373	37.51	37.04	36.05	98	3	CON	REST2	28	50
	374	37.51	37.12	36.03	106	3	CON	REST2	28	50
	375	37.51	37.16	36.03	106	3	CON	REST2	28	50
	376	37.51	37.13	36.05	108	3	CON	REST2	28	50

min	number	Tre	Tes	Tsk-head	HR	ID	condition	period	Ta	RH
	377	37.51	37.12	36.04	99	3	CON	REST2	28	50
	378	37.51	37.11	36.05	101	3	CON	REST2	28	50
	379	37.51	37.02	36.06	103	3	CON	REST2	28	50
	380	37.51	36.98	36.04	94	3	CON	REST2	28	50
	381	37.50	36.97	36.03	94	3	CON	REST2	28	50
	382	37.51	37.00	36.03	99	3	CON	REST2	28	50
	383	37.50	36.98	36.04	96	3	CON	REST2	28	50
	384	37.50	36.92	36.05	98	3	CON	REST2	28	50
	385	37.51	36.95	36.03	98	3	CON	REST2	28	50
	386	37.51	36.95	35.99	98	3	CON	REST2	28	50
	387	37.51	36.95	35.95	100	3	CON	REST2	28	50
	388	37.51	36.97	35.98	98	3	CON	REST2	28	50
	389	37.51	36.96	36.02	91	3	CON	REST2	28	50
	390	37.51	36.97	36.02	95	3	CON	REST2	28	50
65	391	37.50	36.97	36.02	92	3	CON	REST2	28	50
	392	37.50	36.97	36.04	93	3	CON	REST2	28	50
	393	37.50	36.98	36.06	96	3	CON	REST2	28	50
	394	37.50	36.98	36.04	107	3	CON	REST2	28	50
	395	37.49	36.99	36.03	95	3	CON	REST2	28	50
	396	37.49	37.00	36.03	93	3	CON	REST2	28	50
	397	37.49	37.02	36.01	98	3	CON	REST2	28	50
	398	37.48	37.02	36.01	100	3	CON	REST2	28	50
	399	37.48	37.03	36.00	94	3	CON	REST2	28	50
	400	37.48	37.04	36.01	95	3	CON	REST2	28	50
	401	37.48	37.02	36.06	100	3	CON	REST2	28	50
	402	37.48	37.02	36.06	94	3	CON	REST2	28	50
	403	37.49	37.03	36.05	96	3	CON	REST2	28	50
	404	37.49	37.04	36.05	95	3	CON	REST2	28	50
	405	37.49	37.06	36.01	96	3	CON	REST2	28	50
	406	37.50	37.07	36.03	97	3	CON	REST2	28	50
	407	37.49	37.04	36.09	95	3	CON	REST2	28	50
	408	37.49	37.03	36.03	97	3	CON	REST2	28	50
	409	37.48	37.05	36.04	97	3	CON	REST2	28	50
	410	37.48	36.98	36.11	90	3	CON	REST2	28	50
	411	37.48	36.96	36.07	88	3	CON	REST2	28	50
	412	37.48	37.01	36.04	92	3	CON	REST2	28	50
	413	37.48	37.02	36.03	89	3	CON	REST2	28	50
	414	37.48	37.05	36.01	92	3	CON	REST2	28	50
	415	37.47	37.07	36.00	92	3	CON	REST2	28	50
	416	37.47	37.05	36.00	87	3	CON	REST2	28	50
	417	37.47	37.03	36.00	93	3	CON	REST2	28	50
	418	37.48	37.03	36.00	94	3	CON	REST2	28	50
	419	37.47	37.01	36.04	100	3	CON	REST2	28	50
	420	37.47	37.02	36.06	92	3	CON	REST2	28	50
70	421	37.47	36.94	36.03	93	3	CON	REST2	28	50
	422	37.47	36.88	36.04	88	3	CON	REST2	28	50

min	number	Tre	Tes	Tsk-head	HR	ID	condition	period	Ta	RH
	423	37.47	36.95	36.02	89	3	CON	REST2	28	50
	424	37.47	37.00	35.99	93	3	CON	REST2	28	50
	425	37.46	37.01	35.99	96	3	CON	REST2	28	50
	426	37.45	37.01	35.99	97	3	CON	REST2	28	50
	427	37.45	37.03	35.97	99	3	CON	REST2	28	50
	428	37.43	37.04	35.96	106	3	CON	REST2	28	50
	429	37.42	37.06	35.97	101	3	CON	REST2	28	50
	430	37.43	37.12	35.97	95	3	CON	REST2	28	50
	431	37.43	37.16	35.96	93	3	CON	REST2	28	50
	432	37.43	37.21	35.93	96	3	CON	REST2	28	50
	433	37.42	37.23	35.94	96	3	CON	REST2	28	50
	434	37.42	37.23	35.99	100	3	CON	REST2	28	50
	435	37.42	37.24	36.02	97	3	CON	REST2	28	50
	436	37.42	37.26	36.03	104	3	CON	REST2	28	50
	437	37.42	37.26	36.05	101	3	CON	REST2	28	50
	438	37.41	37.26	36.07	93	3	CON	REST2	28	50
	439	37.41	37.27	36.07	96	3	CON	REST2	40	50
	440	37.41	37.29	36.29	108	3	CON	REST2	40	50
	441	37.42	37.31	36.58	112	3	CON	REST2	40	50
	442	37.43	37.31	36.74	113	3	CON	REST2	40	50
	443	37.44	37.31	36.83	109	3	CON	REST2	40	50
	444	37.45	37.32	36.89	107	3	CON	REST2	40	50
	445	37.45	37.31	36.93	107	3	CON	REST2	40	50
	446	37.45	37.32	36.96	100	3	CON	REST2	40	50
	447	37.45	37.30	36.98	99	3	CON	REST2	40	50
	448	37.45	37.28	37.01	98	3	CON	REST2	40	50
	449	37.46	37.26	37.03	99	3	CON	REST2	40	50
	450	37.46	37.24	37.04	102	3	CON	REST2	40	50
75	451	37.47	37.22	37.05	96	3	CON	REST2	40	50
	452	37.47	37.18	37.06	100	3	CON	REST2	40	50
	453	37.46	37.15	37.04	103	3	CON	REST2	40	50
	454	37.45	37.15	37.04	97	3	CON	REST2	40	50
	455	37.45	37.16	37.04	100	3	CON	REST2	40	50
	456	37.46	37.14	37.06	100	3	CON	REST2	40	50
	457	37.45	37.11	37.06	105	3	CON	REST2	40	50
	458	37.45	37.08	37.04	111	3	CON	REST2	40	50
	459	37.43	37.07	37.03	111	3	CON	REST2	40	50
	460	37.41	37.07	37.02	105	3	CON	REST2	40	50
	461	37.41	37.07	37.02	110	3	CON	REST2	40	50
	462	37.41	37.09	37.02	112	3	CON	REST2	40	50
	463	37.41	37.08	37.02	114	3	CON	EXERCISE2	40	50
	464	37.41	37.09	37.02	112	3	CON	EXERCISE2	40	50
	465	37.41	37.10	37.02	112	3	CON	EXERCISE2	40	50
	466	37.40	37.07	37.03	113	3	CON	EXERCISE2	40	50
	467	37.40	37.06	37.04	124	3	CON	EXERCISE2	40	50
	468	37.39	37.06	37.04	126	3	CON	EXERCISE2	40	50

min	number	Tre	Tes	Tsk-head	HR	ID	condition	period	Ta	RH
	469	37.38	37.07	37.06	126	3	CON	EXERCISE2	40	50
	470	37.38	37.07	37.07	125	3	CON	EXERCISE2	40	50
	471	37.38	37.08	37.08	127	3	CON	EXERCISE2	40	50
	472	37.39	37.11	37.10	129	3	CON	EXERCISE2	40	50
	473	37.40	37.13	37.11	130	3	CON	EXERCISE2	40	50
	474	37.41	37.15	37.13	128	3	CON	EXERCISE2	40	50
	475	37.41	37.17	37.16	130	3	CON	EXERCISE2	40	50
	476	37.41	37.19	37.17	134	3	CON	EXERCISE2	40	50
	477	37.41	37.20	37.17	134	3	CON	EXERCISE2	40	50
	478	37.41	37.20	37.19	134	3	CON	EXERCISE2	40	50
	479	37.41	37.21	37.20	131	3	CON	EXERCISE2	40	50
	480	37.41	37.24	37.21	133	3	CON	EXERCISE2	40	50
80	481	37.42	37.26	37.22	131	3	CON	EXERCISE2	40	50
	482	37.41	37.29	37.23	132	3	CON	EXERCISE2	40	50
	483	37.41	37.27	37.24	135	3	CON	EXERCISE2	40	50
	484	37.41	37.20	37.25	134	3	CON	EXERCISE2	40	50
	485	37.41	37.22	37.26	134	3	CON	EXERCISE2	40	50
	486	37.41	37.26	37.27	136	3	CON	EXERCISE2	40	50
	487	37.41	37.26	37.29	137	3	CON	EXERCISE2	40	50
	488	37.41	37.27	37.31	137	3	CON	EXERCISE2	40	50
	489	37.41	37.28	37.33	137	3	CON	EXERCISE2	40	50
	490	37.41	37.29	37.33	142	3	CON	EXERCISE2	40	50
	491	37.40	37.32	37.31	145	3	CON	EXERCISE2	40	50
	492	37.40	37.32	37.33	143	3	CON	EXERCISE2	40	50
	493	37.42	37.34	37.34	140	3	CON	EXERCISE2	40	50
	494	37.43	37.36	37.34	140	3	CON	EXERCISE2	40	50
	495	37.42	37.37	37.35	140	3	CON	EXERCISE2	40	50
	496	37.42	37.38	37.36	141	3	CON	EXERCISE2	40	50
	497	37.42	37.40	37.37	140	3	CON	EXERCISE2	40	50
	498	37.41	37.43	37.36	140	3	CON	EXERCISE2	40	50
	499	37.42	37.43	37.38	141	3	CON	EXERCISE2	40	50
	500	37.43	37.43	37.40	144	3	CON	EXERCISE2	40	50
	501	37.44	37.43	37.41	138	3	CON	EXERCISE2	40	50
	502	37.45	37.43	37.41	139	3	CON	EXERCISE2	40	50
	503	37.47	37.45	37.40	146	3	CON	EXERCISE2	40	50
	504	37.47	37.48	37.41	146	3	CON	EXERCISE2	40	50
	505	37.47	37.48	37.41	148	3	CON	EXERCISE2	40	50
	506	37.46	37.48	37.42	144	3	CON	EXERCISE2	40	50
	507	37.45	37.51	37.44	143	3	CON	EXERCISE2	40	50
	508	37.45	37.53	37.44	148	3	CON	EXERCISE2	40	50
	509	37.46	37.52	37.46	147	3	CON	EXERCISE2	40	50
	510	37.47	37.49	37.48	144	3	CON	EXERCISE2	40	50
85	511	37.46	37.50	37.49	143	3	CON	EXERCISE2	40	50
	512	37.46	37.56	37.47	146	3	CON	EXERCISE2	40	50
	513	37.47	37.57	37.46	151	3	CON	EXERCISE2	40	50
	514	37.47	37.57	37.47	150	3	CON	EXERCISE2	40	50

min	number	Tre	Tes	Tsk-head	HR	ID	condition	period	Ta	RH
	515	37.46	37.59	37.49	148	3	CON	EXERCISE2	40	50
	516	37.46	37.59	37.49	147	3	CON	EXERCISE2	40	50
	517	37.46	37.63	37.51	146	3	CON	EXERCISE2	40	50
	518	37.46	37.67	37.53	145	3	CON	EXERCISE2	40	50
	519	37.46	37.67	37.55	143	3	CON	EXERCISE2	40	50
	520	37.47	37.70	37.55	146	3	CON	EXERCISE2	40	50
	521	37.47	37.78	37.55	151	3	CON	EXERCISE2	40	50
	522	37.48	37.84	37.57	151	3	CON	EXERCISE2	40	50
	523	37.49	37.86	37.59	166	3	CON	EXERCISE2	40	50
	524	37.48	37.85	37.59	149	3	CON	EXERCISE2	40	50
	525	37.47	37.88	37.58	154	3	CON	EXERCISE2	40	50
	526	37.47	37.90	37.59	157	3	CON	EXERCISE2	40	50
	527	37.48	37.88	37.59	150	3	CON	EXERCISE2	40	50
	528	37.48	37.85	37.58	153	3	CON	EXERCISE2	40	50
	529	37.48	37.85	37.58	154	3	CON	EXERCISE2	40	50
	530	37.48	37.83	37.59	154	3	CON	EXERCISE2	40	50
	531	37.48	37.82	37.59	151	3	CON	EXERCISE2	40	50
	532	37.48	37.82	37.60	151	3	CON	EXERCISE2	40	50
	533	37.48	37.80	37.60	153	3	CON	EXERCISE2	40	50
	534	37.49	37.78	37.57	149	3	CON	EXERCISE2	40	50
	535	37.50	37.79	37.57	150	3	CON	EXERCISE2	40	50
	536	37.52	37.83	37.62	154	3	CON	EXERCISE2	40	50
	537	37.52	37.81	37.64	154	3	CON	EXERCISE2	40	50
	538	37.52	37.79	37.66	149	3	CON	EXERCISE2	40	50
	539	37.51	37.80	37.66	149	3	CON	EXERCISE2	40	50
	540	37.51	37.83	37.65	150	3	CON	EXERCISE2	40	50
90	541	37.53	37.84	37.65	153	3	CON	EXERCISE2	40	50
	542	37.54	37.83	37.67	154	3	CON	EXERCISE2	40	50
	543	37.55	37.82	37.69	149	3	CON	EXERCISE2	40	50
	544	37.55	37.82	37.69	149	3	CON	EXERCISE2	40	50
	545	37.54	37.83	37.70	148	3	CON	EXERCISE2	40	50
	546	37.54	37.84	37.70	150	3	CON	EXERCISE2	40	50
	547	37.54	37.85	37.69	154	3	CON	EXERCISE2	40	50
	548	37.55	37.87	37.69	154	3	CON	EXERCISE2	40	50
	549	37.55	37.89	37.69	154	3	CON	EXERCISE2	40	50
	550	37.56	37.90	37.71	157	3	CON	EXERCISE2	40	50
	551	37.58	37.90	37.74	156	3	CON	EXERCISE2	40	50
	552	37.58	37.86	37.75	155	3	CON	EXERCISE2	40	50
	553	37.59	37.87	37.76	154	3	CON	EXERCISE2	40	50
	554	37.60	37.91	37.76	154	3	CON	EXERCISE2	40	50
	555	37.61	37.92	37.77	154	3	CON	EXERCISE2	40	50
	556	37.63	37.90	37.79	157	3	CON	EXERCISE2	40	50
	557	37.63	37.92	37.77	154	3	CON	EXERCISE2	40	50
	558	37.65	37.95	37.75	158	3	CON	EXERCISE2	40	50
	559	37.67	37.95	37.76	157	3	CON	EXERCISE2	40	50
	560	37.67	37.89	37.76	157	3	CON	EXERCISE2	40	50

min	number	Tre	Tes	Tsk-head	HR	ID	condition	period	Ta	RH
	561	37.68	37.89	37.77	154	3	CON	EXERCISE2	40	50
	562	37.68	37.93	37.77	155	3	CON	EXERCISE2	40	50
	563	37.68	37.86	37.76	154	3	CON	EXERCISE2	40	50
	564	37.67	37.87	37.77	154	3	CON	EXERCISE2	40	50
	565	37.68	37.93	37.80	154	3	CON	EXERCISE2	40	50
	566	37.68	37.95	37.80	156	3	CON	EXERCISE2	40	50
	567	37.69	37.97	37.80	157	3	CON	EXERCISE2	40	50
	568	37.69	37.99	37.80	158	3	CON	EXERCISE2	40	50
	569	37.69	38.01	37.79	162	3	CON	EXERCISE2	40	50
	570	37.71	38.01	37.81	162	3	CON	EXERCISE2	40	50
95	571	37.72	38.02	37.83	159	3	CON	EXERCISE2	40	50
	572	37.72	38.02	37.84	162	3	CON	EXERCISE2	40	50
	573	37.72	37.99	37.84	162	3	CON	EXERCISE2	40	50
	574	37.73	38.00	37.83	164	3	CON	EXERCISE2	40	50
	575	37.74	38.01	37.83	164	3	CON	EXERCISE2	40	50
	576	37.74	38.03	37.84	162	3	CON	EXERCISE2	40	50
	577	37.75	38.05	37.85	163	3	CON	EXERCISE2	40	50
	578	37.75	38.03	37.85	165	3	CON	EXERCISE2	40	50
	579	37.75	38.02	37.82	162	3	CON	EXERCISE2	40	50
	580	37.76	38.03	37.77	165	3	CON	EXERCISE2	40	50
	581	37.76	38.03	37.78	162	3	CON	EXERCISE2	40	50
	582	37.76	38.03	37.81	165	3	CON	EXERCISE2	40	50
	583	37.78	38.02	37.83	165	3	CON	EXERCISE2	40	50
	584	37.79	38.02	37.87	166	3	CON	EXERCISE2	40	50
	585	37.79	38.01	37.86	166	3	CON	EXERCISE2	40	50
	586	37.79	38.05	37.85	166	3	CON	EXERCISE2	40	50
	587	37.79	38.08	37.87	165	3	CON	EXERCISE2	40	50
	588	37.79	38.07	37.89	166	3	CON	EXERCISE2	40	50
	589	37.79	38.07	37.91	166	3	CON	EXERCISE2	40	50
	590	37.80	38.08	37.92	166	3	CON	EXERCISE2	40	50
	591	37.81	38.09	37.93	166	3	CON	EXERCISE2	40	50
	592	37.81	38.11	37.94	166	3	CON	EXERCISE2	40	50
	593	37.81	38.12	37.94	163	3	CON	EXERCISE2	40	50
	594	37.81	38.13	37.95	166	3	CON	EXERCISE2	40	50
	595	37.82	38.13	37.95	169	3	CON	EXERCISE2	40	50
	596	37.82	38.15	37.96	167	3	CON	EXERCISE2	40	50
	597	37.83	38.15	37.97	169	3	CON	EXERCISE2	40	50
	598	37.83	38.15	37.98	166	3	CON	EXERCISE2	40	50
	599	37.83	38.17	37.99	166	3	CON	EXERCISE2	40	50
	600	37.83	38.18	37.99	166	3	CON	EXERCISE2	40	50
100	601	37.85	38.17	37.99	170	3	CON	EXERCISE2	40	50
	602	37.86	38.20	38.01	170	3	CON	EXERCISE2	40	50
	603	37.87	38.22	38.00	170	3	CON	EXERCISE2	40	50
	604	37.87	38.22	37.99	172	3	CON	EXERCISE2	40	50
	605	37.88	38.23	37.99	170	3	CON	EXERCISE2	40	50
	606	37.88	38.25	38.00	170	3	CON	EXERCISE2	40	50

min	number	Tre	Tes	Tsk-head	HR	ID	condition	period	Ta	RH
	607	37.88	38.23	38.00	169	3	CON	EXERCISE2	40	50
	608	37.90	38.23	38.02	167	3	CON	EXERCISE2	40	50
	609	37.91	38.27	38.02	166	3	CON	EXERCISE2	40	50
	610	37.91	38.30	38.02	169	3	CON	EXERCISE2	40	50
	611	37.91	38.33	38.03	170	3	CON	EXERCISE2	40	50
	612	37.91	38.36	38.03	170	3	CON	EXERCISE2	40	50
	613	37.92	38.38	38.03	170	3	CON	EXERCISE2	40	50
	614	37.93	38.41	38.04	170	3	CON	EXERCISE2	40	50
	615	37.94	38.41	38.06	170	3	CON	EXERCISE2	40	50
	616	37.94	38.40	38.07	170	3	CON	EXERCISE2	40	50
	617	37.94	38.42	38.08	171	3	CON	EXERCISE2	40	50
	618	37.94	38.40	38.09	170	3	CON	EXERCISE2	40	50
	619	37.94	38.41	38.10	175	3	CON	EXERCISE2	40	50
	620	37.95	38.44	38.09	171	3	CON	EXERCISE2	40	50
	621	37.96	38.45	38.09	173	3	CON	EXERCISE2	40	50
	622	37.97	38.45	38.11	170	3	CON	EXERCISE2	40	50
	623	37.98	38.48	38.13	171	3	CON	EXERCISE2	40	50
	624	37.98	38.52	38.13	170	3	CON	EXERCISE2	40	50
	625	37.99	38.49	38.14	172	3	CON	EXERCISE2	40	50
	626	38.00	38.46	38.16	170	3	CON	EXERCISE2	40	50
	627	37.99	38.47	38.18	170	3	CON	EXERCISE2	40	50
	628	37.99	38.49	38.19	166	3	CON	EXERCISE2	40	50
	629	38.00	38.48	38.19	170	3	CON	EXERCISE2	40	50
	630	38.01	38.45	38.17	170	3	CON	EXERCISE2	40	50
105	631	38.02	38.42	38.16	171	3	CON	EXERCISE2	40	50
	632	38.03	38.43	38.17	170	3	CON	EXERCISE2	40	50
	633	38.03	38.43	38.19	170	3	CON	EXERCISE2	40	50
	634	38.03	38.43	38.20	170	3	CON	EXERCISE2	40	50
	635	38.03	38.43	38.20	170	3	CON	EXERCISE2	40	50
	636	38.02	38.44	38.22	170	3	CON	EXERCISE2	40	50
	637	38.03	38.47	38.22	170	3	CON	EXERCISE2	40	50
	638	38.04	38.46	38.22	175	3	CON	EXERCISE2	40	50
	639	38.05	38.44	38.23	175	3	CON	EXERCISE2	40	50
	640	38.05	38.47	38.24	175	3	CON	EXERCISE2	40	50
	641	38.06	38.49	38.25	175	3	CON	EXERCISE2	40	50
	642	38.07	38.50	38.24	172	3	CON	EXERCISE2	40	50
	643	38.08	38.51	38.23	175	3	CON	EXERCISE2	40	50
	644	38.09	38.50	38.24	173	3	CON	REST3	28	50
	645	38.10	38.53	38.09	174	3	CON	REST3	28	50
	646	38.10	38.59	37.83	170	3	CON	REST3	28	50
	647	38.10	38.63	37.67	167	3	CON	REST3	28	50
	648	38.11	38.45	37.55	162	3	CON	REST3	28	50
	649	38.12	38.32	37.45	162	3	CON	REST3	28	50
	650	38.11	38.34	37.40	162	3	CON	REST3	28	50
	651	38.12	38.32	37.35	160	3	CON	REST3	28	50
	652	38.12	38.41	37.26	150	3	CON	REST3	28	50

min	number	Tre	Tes	Tsk-head	HR	ID	condition	period	Ta	RH
	653	38.12	38.45	37.19	140	3	CON	REST3	28	50
	654	38.13	38.40	37.18	158	3	CON	REST3	28	50
	655	38.15	38.34	37.20	154	3	CON	REST3	28	50
	656	38.16	38.39	37.16	147	3	CON	REST3	28	50
	657	38.17	38.38	37.14	145	3	CON	REST3	28	50
	658	38.18	38.27	37.16	143	3	CON	REST3	28	50
	659	38.18	38.20	37.14	147	3	CON	REST3	28	50
	660	38.19	38.21	37.09	150	3	CON	REST3	28	50
110	661	38.20	38.24	37.05	174	3	CON	REST3	28	50
	662	38.21	38.28	36.99	151	3	CON	REST3	28	50
	663	38.21	38.29	36.90	154	3	CON	REST3	28	50
	664	38.21	38.22	36.89	156	3	CON	REST3	28	50
	665	38.22	38.06	36.88	154	3	CON	REST3	28	50
	666	38.22	131.48	36.64	147	3	CON	REST3	28	50
	667	38.22	162.47	36.61	140	3	CON	REST3	28	50
	668	38.22	167.61	36.76	134	3	CON	REST3	28	50
	669	38.21	249.27	36.71	141	3	CON	REST3	28	50
	670	38.21	258.78	36.70	132	3	CON	REST3	28	50
	671	38.21	221.34	36.67	130	3	CON	REST3	28	50
	672	38.22	145.64	36.62	126	3	CON	REST3	28	50
	673	38.23	103.63	36.58	131	3	CON	REST3	28	50
	674	38.23	102.55	36.51	127	3	CON	REST3	28	50
	675	38.24	69.35	36.45	127	3	CON	REST3	28	50
	676	38.24	37.93	36.43	124	3	CON	REST3	28	50
	677	38.25	37.92	36.43	130	3	CON	REST3	28	50
	678	38.25	37.88	36.42	134	3	CON	REST3	28	50
	679	38.25	37.85	36.40	132	3	CON	REST3	28	50
	680	38.26	37.85	36.27	128	3	CON	REST3	28	50
	681	38.26	37.92	36.04	132	3	CON	REST3	28	50
	682	38.26	38.03	35.97	138	3	CON	REST3	28	50
	683	38.27	37.96	35.91	137	3	CON	REST3	28	50
	684	38.27	37.79	35.87	135	3	CON	REST3	28	50
	685	38.27	37.74	35.81	129	3	CON	REST3	28	50
	686	38.27	37.67	35.78	135	3	CON	REST3	28	50
	687	38.27	37.66	35.74	131	3	CON	REST3	28	50
	688	38.26	37.71	35.51	131	3	CON	REST3	28	50
	689	38.26	37.69	35.43	135	3	CON	REST3	28	50
	690	38.27	37.67	35.43	131	3	CON	REST3	28	50
115	691	38.27	37.67	35.42	125	3	CON	REST3	28	50
	692	38.27	37.70	35.45	122	3	CON	REST3	28	50
	693	38.26	37.71	35.48	125	3	CON	REST3	28	50
	694	38.27	37.67	35.52	117	3	CON	REST3	28	50
	695	38.27	37.64	35.50	123	3	CON	REST3	28	50
	696	38.27	37.63	35.46	119	3	CON	REST3	28	50
	697	38.26	37.64	35.39	122	3	CON	REST3	28	50
	698	38.26	37.77	35.35	122	3	CON	REST3	28	50

min	number	Tre	Tes	Tsk-head	HR	ID	condition	period	Ta	RH
	699	38.25	37.88	35.42	113	3	CON	REST3	28	50
	700	38.25	37.79	35.43	119	3	CON	REST3	28	50
	701	38.25	37.67	35.44	117	3	CON	REST3	28	50
	702	38.24	37.62	35.40	116	3	CON	REST3	28	50
	703	38.24	37.62	35.34	120	3	CON	REST3	28	50
	704	38.24	37.61	35.29	120	3	CON	REST3	28	50
	705	38.23	37.61	35.20	117	3	CON	REST3	28	50
	706	38.23	37.64	35.13	119	3	CON	REST3	28	50
	707	38.23	37.65	35.12	124	3	CON	REST3	28	50
	708	38.23	37.63	35.13	120	3	CON	REST3	28	50
	709	38.22	37.62	35.11	123	3	CON	REST3	28	50
0	1	36.92	36.82	34.99	81	4	CON	REST1	28	50
	2	36.91	36.82	34.97	76	4	CON	REST1	28	50
	3	36.91	36.81	34.98	84	4	CON	REST1	28	50
	4	36.91	36.81	34.96	80	4	CON	REST1	28	50
	5	36.90	36.82	34.96	80	4	CON	REST1	28	50
	6	36.90	36.83	34.96	86	4	CON	REST1	28	50
	7	36.90	36.83	34.95	78	4	CON	REST1	28	50
	8	36.91	36.85	34.96	78	4	CON	REST1	28	50
	9	36.91	36.87	34.97	78	4	CON	REST1	28	50
	10	36.91	36.87	34.96	76	4	CON	REST1	28	50
	11	36.91	36.86	34.96	77	4	CON	REST1	28	50
	12	36.90	36.85	34.99	74	4	CON	REST1	28	50
	13	36.91	36.88	34.98	75	4	CON	REST1	28	50
	14	36.91	36.87	34.91	74	4	CON	REST1	28	50
	15	36.91	36.87	34.90	76	4	CON	REST1	28	50
	16	36.91	36.88	34.94	75	4	CON	REST1	28	50
	17	36.90	36.86	34.95	78	4	CON	REST1	28	50
	18	36.91	36.87	34.96	77	4	CON	REST1	28	50
	19	36.90	36.88	34.95	79	4	CON	REST1	28	50
	20	36.90	36.87	34.95	80	4	CON	REST1	28	50
	21	36.91	36.88	34.96	81	4	CON	REST1	28	50
	22	36.91	36.89	34.96	77	4	CON	REST1	28	50
	23	36.92	36.88	34.95	77	4	CON	REST1	28	50
	24	36.91	36.88	34.96	77	4	CON	REST1	28	50
	25	36.90	36.87	34.96	77	4	CON	REST1	28	50
	26	36.91	36.89	34.96	75	4	CON	REST1	28	50
	27	36.91	36.90	34.97	76	4	CON	REST1	28	50
	28	36.91	36.88	34.95	79	4	CON	REST1	28	50
	29	36.91	36.88	34.94	79	4	CON	REST1	28	50
	30	36.91	36.89	34.94	78	4	CON	REST1	28	50
5	31	36.92	36.90	34.92	79	4	CON	REST1	28	50
	32	36.92	36.89	34.91	79	4	CON	REST1	28	50
	33	36.92	36.88	34.93	78	4	CON	REST1	28	50
	34	36.91	36.87	34.94	76	4	CON	REST1	28	50
	35	36.90	36.88	34.93	77	4	CON	REST1	28	50

min	number	Tre	Tes	Tsk-head	HR	ID	condition	period	Ta	RH
	36	36.90	36.90	34.93	75	4	CON	REST1	28	50
	37	36.90	36.89	34.93	75	4	CON	REST1	28	50
	38	36.90	36.87	34.94	81	4	CON	REST1	28	50
	39	36.90	36.88	34.93	76	4	CON	REST1	28	50
	40	36.90	36.88	34.92	79	4	CON	REST1	28	50
	41	36.90	36.88	34.95	82	4	CON	REST1	28	50
	42	36.90	36.88	34.97	78	4	CON	REST1	28	50
	43	36.90	36.88	34.97	78	4	CON	REST1	28	50
	44	36.90	36.89	34.96	81	4	CON	REST1	28	50
	45	36.90	36.90	34.96	83	4	CON	REST1	28	50
	46	36.90	36.89	34.94	84	4	CON	REST1	28	50
	47	36.90	36.89	34.93	83	4	CON	REST1	28	50
	48	36.90	36.90	34.92	82	4	CON	REST1	28	50
	49	36.90	36.91	34.93	81	4	CON	REST1	28	50
	50	36.91	36.86	34.95	86	4	CON	REST1	28	50
	51	36.91	36.82	34.97	81	4	CON	REST1	28	50
	52	36.91	36.86	34.97	81	4	CON	REST1	28	50
	53	36.91	36.87	34.95	82	4	CON	REST1	28	50
	54	36.91	36.88	34.96	82	4	CON	REST1	28	50
	55	36.92	36.89	34.95	82	4	CON	REST1	28	50
	56	36.91	36.89	34.96	83	4	CON	REST1	28	50
	57	36.91	36.91	34.96	81	4	CON	REST1	28	50
	58	36.92	36.91	34.94	77	4	CON	REST1	28	50
	59	36.91	36.90	34.93	78	4	CON	REST1	28	50
	60	36.91	36.89	34.93	77	4	CON	REST1	28	50
10	61	36.91	36.88	34.94	76	4	CON	REST1	28	50
	62	36.91	36.89	34.95	76	4	CON	REST1	28	50
	63	36.90	36.91	34.96	78	4	CON	REST1	28	50
	64	36.91	36.91	34.97	73	4	CON	REST1	28	50
	65	36.91	36.90	34.97	74	4	CON	REST1	28	50
	66	36.91	36.89	34.95	74	4	CON	REST1	28	50
	67	36.91	36.90	34.95	76	4	CON	REST1	28	50
	68	36.91	36.90	34.95	78	4	CON	REST1	28	50
	69	36.91	36.90	34.96	76	4	CON	REST1	28	50
	70	36.91	36.92	34.97	76	4	CON	REST1	28	50
	71	36.91	36.91	34.96	76	4	CON	REST1	28	50
	72	36.91	36.91	34.96	74	4	CON	REST1	28	50
	73	36.91	36.89	34.95	88	4	CON	REST1	28	50
	74	36.92	36.90	34.97	83	4	CON	REST1	28	50
	75	36.92	36.91	34.99	81	4	CON	REST1	28	50
	76	36.91	36.88	35.00	80	4	CON	REST1	28	50
	77	36.90	36.90	35.00	81	4	CON	REST1	28	50
	78	36.91	36.90	34.97	82	4	CON	REST1	28	50
	79	36.92	36.90	34.97	82	4	CON	REST1	28	50
	80	36.91	36.92	34.98	80	4	CON	REST1	28	50
	81	36.91	36.91	34.96	81	4	CON	REST1	28	50

min	number	Tre	Tes	Tsk-head	HR	ID	condition	period	Ta	RH
	82	36.91	36.88	34.95	83	4	CON	REST1	28	50
	83	36.91	36.89	34.97	85	4	CON	REST1	28	50
	84	36.92	36.91	34.99	80	4	CON	REST1	28	50
	85	36.92	36.90	34.97	82	4	CON	REST1	28	50
	86	36.92	36.89	34.97	79	4	CON	REST1	28	50
	87	36.92	36.90	34.99	78	4	CON	REST1	28	50
	88	36.92	36.89	34.99	78	4	CON	REST1	28	50
	89	36.92	36.89	34.98	77	4	CON	REST1	28	50
	90	36.93	36.90	34.98	77	4	CON	REST1	28	50
15	91	36.93	36.89	35.00	79	4	CON	REST1	28	50
	92	36.93	36.90	35.00	78	4	CON	REST1	28	50
	93	36.93	36.91	35.00	78	4	CON	REST1	28	50
	94	36.93	36.90	34.98	82	4	CON	REST1	28	50
	95	36.94	36.89	34.98	92	4	CON	REST1	28	50
	96	36.95	36.86	34.99	86	4	CON	REST1	28	50
	97	36.96	36.84	35.00	88	4	CON	REST1	28	50
	98	36.97	36.87	34.99	87	4	CON	REST1	28	50
	99	36.98	36.92	35.01	84	4	CON	REST1	28	50
	100	36.98	36.92	35.06	88	4	CON	REST1	28	50
	101	36.97	36.86	35.09	90	4	CON	REST1	28	50
	102	36.97	36.86	35.10	85	4	CON	REST1	28	50
	103	36.96	36.85	35.09	88	4	CON	REST1	40	50
	104	36.97	36.83	35.31	96	4	CON	REST1	40	50
	105	36.98	36.84	35.58	91	4	CON	REST1	40	50
	106	36.99	36.83	35.68	94	4	CON	REST1	40	50
	107	36.99	36.82	35.76	92	4	CON	REST1	40	50
	108	36.99	36.80	35.83	86	4	CON	REST1	40	50
	109	36.98	36.79	35.90	85	4	CON	REST1	40	50
	110	36.98	36.79	35.93	90	4	CON	REST1	40	50
	111	36.97	36.78	35.97	86	4	CON	REST1	40	50
	112	36.97	36.77	36.00	82	4	CON	REST1	40	50
	113	36.97	36.78	36.01	83	4	CON	REST1	40	50
	114	36.97	36.79	36.06	81	4	CON	REST1	40	50
	115	36.97	36.81	36.12	84	4	CON	REST1	40	50
	116	36.96	36.81	36.16	82	4	CON	REST1	40	50
	117	36.96	36.80	36.18	84	4	CON	REST1	40	50
	118	36.96	36.80	36.19	84	4	CON	REST1	40	50
	119	36.96	36.82	36.21	84	4	CON	REST1	40	50
	120	36.96	36.82	36.23	83	4	CON	REST1	40	50
20	121	36.96	36.82	36.27	84	4	CON	REST1	40	50
	122	36.96	36.79	36.32	89	4	CON	REST1	40	50
	123	36.96	36.79	36.35	87	4	CON	REST1	40	50
	124	36.96	36.81	36.38	81	4	CON	REST1	40	50
	125	36.96	36.81	36.41	81	4	CON	REST1	40	50
	126	36.96	36.85	36.45	83	4	CON	REST1	40	50
	127	36.96	36.86	36.46	84	4	CON	REST1	40	50

min	number	Tre	Tes	Tsk-head	HR	ID	condition	period	Ta	RH
	128	36.96	36.85	36.48	88	4	CON	REST1	40	50
	129	36.96	36.84	36.53	81	4	CON	REST1	40	50
	130	36.95	36.84	36.57	86	4	CON	REST1	40	50
	131	36.95	36.86	36.57	86	4	CON	REST1	40	50
	132	36.96	36.85	36.56	82	4	CON	REST1	40	50
	133	36.95	36.83	36.55	79	4	CON	REST1	40	50
	134	36.96	36.82	36.57	94	4	CON	REST1	40	50
	135	36.98	36.82	36.61	98	4	CON	REST1	40	50
	136	36.98	36.83	36.62	84	4	CON	REST1	40	50
	137	36.99	36.83	36.62	83	4	CON	REST1	40	50
	138	36.99	36.84	36.62	88	4	CON	REST1	40	50
	139	37.00	36.86	36.61	88	4	CON	EXERCISE1	40	50
	140	37.00	36.87	36.62	96	4	CON	EXERCISE1	40	50
	141	36.99	36.87	36.65	98	4	CON	EXERCISE1	40	50
	142	36.99	36.85	36.68	103	4	CON	EXERCISE1	40	50
	143	36.99	36.84	36.71	103	4	CON	EXERCISE1	40	50
	144	36.99	36.84	36.71	105	4	CON	EXERCISE1	40	50
	145	36.99	36.85	36.71	107	4	CON	EXERCISE1	40	50
	146	36.99	36.87	36.72	108	4	CON	EXERCISE1	40	50
	147	37.00	36.86	36.76	111	4	CON	EXERCISE1	40	50
	148	37.00	36.85	36.79	114	4	CON	EXERCISE1	40	50
	149	37.00	36.86	36.80	115	4	CON	EXERCISE1	40	50
	150	37.01	36.86	36.80	115	4	CON	EXERCISE1	40	50
25	151	37.02	36.87	36.80	116	4	CON	EXERCISE1	40	50
	152	37.02	36.87	36.80	117	4	CON	EXERCISE1	40	50
	153	37.02	36.87	36.82	113	4	CON	EXERCISE1	40	50
	154	37.02	36.90	36.83	114	4	CON	EXERCISE1	40	50
	155	37.02	36.92	36.83	118	4	CON	EXERCISE1	40	50
	156	37.03	36.92	36.84	121	4	CON	EXERCISE1	40	50
	157	37.03	36.92	36.83	118	4	CON	EXERCISE1	40	50
	158	37.03	36.95	36.86	119	4	CON	EXERCISE1	40	50
	159	37.05	36.98	36.89	118	4	CON	EXERCISE1	40	50
	160	37.04	37.00	36.88	116	4	CON	EXERCISE1	40	50
	161	37.05	37.03	36.89	118	4	CON	EXERCISE1	40	50
	162	37.06	37.05	36.90	115	4	CON	EXERCISE1	40	50
	163	37.06	37.06	36.91	116	4	CON	EXERCISE1	40	50
	164	37.06	37.08	36.92	118	4	CON	EXERCISE1	40	50
	165	37.07	37.10	36.93	117	4	CON	EXERCISE1	40	50
	166	37.07	37.12	36.92	120	4	CON	EXERCISE1	40	50
	167	37.08	37.12	36.93	122	4	CON	EXERCISE1	40	50
	168	37.09	37.13	36.96	120	4	CON	EXERCISE1	40	50
	169	37.10	37.16	36.97	121	4	CON	EXERCISE1	40	50
	170	37.10	37.19	36.97	119	4	CON	EXERCISE1	40	50
	171	37.11	37.21	36.97	122	4	CON	EXERCISE1	40	50
	172	37.12	37.22	36.99	125	4	CON	EXERCISE1	40	50
	173	37.12	37.23	37.01	125	4	CON	EXERCISE1	40	50

min	number	Tre	Tes	Tsk-head	HR	ID	condition	period	Ta	RH
	174	37.13	37.26	37.01	122	4	CON	EXERCISE1	40	50
	175	37.14	37.29	37.01	122	4	CON	EXERCISE1	40	50
	176	37.14	37.29	37.03	123	4	CON	EXERCISE1	40	50
	177	37.15	37.29	37.07	126	4	CON	EXERCISE1	40	50
	178	37.16	37.32	37.09	125	4	CON	EXERCISE1	40	50
	179	37.17	37.34	37.08	129	4	CON	EXERCISE1	40	50
	180	37.18	37.34	37.11	130	4	CON	EXERCISE1	40	50
30	181	37.19	37.34	37.14	131	4	CON	EXERCISE1	40	50
	182	37.19	37.31	37.15	135	4	CON	EXERCISE1	40	50
	183	37.20	37.32	37.15	130	4	CON	EXERCISE1	40	50
	184	37.21	37.38	37.15	127	4	CON	EXERCISE1	40	50
	185	37.22	37.39	37.16	131	4	CON	EXERCISE1	40	50
	186	37.22	37.39	37.18	129	4	CON	EXERCISE1	40	50
	187	37.23	37.40	37.20	128	4	CON	EXERCISE1	40	50
	188	37.23	37.42	37.20	132	4	CON	EXERCISE1	40	50
	189	37.24	37.43	37.21	134	4	CON	EXERCISE1	40	50
	190	37.24	37.41	37.21	135	4	CON	EXERCISE1	40	50
	191	37.25	37.41	37.21	134	4	CON	EXERCISE1	40	50
	192	37.26	37.43	37.23	134	4	CON	EXERCISE1	40	50
	193	37.27	37.45	37.24	134	4	CON	EXERCISE1	40	50
	194	37.27	37.48	37.23	134	4	CON	EXERCISE1	40	50
	195	37.28	37.48	37.26	132	4	CON	EXERCISE1	40	50
	196	37.29	37.47	37.29	130	4	CON	EXERCISE1	40	50
	197	37.30	37.39	37.29	133	4	CON	EXERCISE1	40	50
	198	37.31	37.39	37.29	134	4	CON	EXERCISE1	40	50
	199	37.32	37.49	37.31	134	4	CON	EXERCISE1	40	50
	200	37.33	37.51	37.31	136	4	CON	EXERCISE1	40	50
	201	37.34	37.51	37.33	137	4	CON	EXERCISE1	40	50
	202	37.34	37.52	37.32	138	4	CON	EXERCISE1	40	50
	203	37.34	37.52	37.33	140	4	CON	EXERCISE1	40	50
	204	37.35	37.54	37.35	142	4	CON	EXERCISE1	40	50
	205	37.35	37.56	37.35	139	4	CON	EXERCISE1	40	50
	206	37.35	37.55	37.37	139	4	CON	EXERCISE1	40	50
	207	37.36	37.57	37.38	139	4	CON	EXERCISE1	40	50
	208	37.37	37.58	37.38	137	4	CON	EXERCISE1	40	50
	209	37.38	37.57	37.41	136	4	CON	EXERCISE1	40	50
	210	37.39	37.60	37.41	137	4	CON	EXERCISE1	40	50
35	211	37.40	37.62	37.40	137	4	CON	EXERCISE1	40	50
	212	37.40	37.61	37.42	139	4	CON	EXERCISE1	40	50
	213	37.41	37.61	37.44	141	4	CON	EXERCISE1	40	50
	214	37.42	37.52	37.45	139	4	CON	EXERCISE1	40	50
	215	37.42	37.49	37.46	139	4	CON	EXERCISE1	40	50
	216	37.44	37.57	37.46	155	4	CON	EXERCISE1	40	50
	217	37.44	37.60	37.45	136	4	CON	EXERCISE1	40	50
	218	37.45	37.63	37.45	138	4	CON	EXERCISE1	40	50
	219	37.46	37.66	37.45	138	4	CON	EXERCISE1	40	50

min	number	Tre	Tes	Tsk-head	HR	ID	condition	period	Ta	RH
	220	37.46	37.68	37.44	141	4	CON	EXERCISE1	40	50
	221	37.47	37.68	37.46	141	4	CON	EXERCISE1	40	50
	222	37.47	37.62	37.48	143	4	CON	EXERCISE1	40	50
	223	37.48	37.62	37.49	140	4	CON	EXERCISE1	40	50
	224	37.49	37.69	37.48	138	4	CON	EXERCISE1	40	50
	225	37.50	37.71	37.47	139	4	CON	EXERCISE1	40	50
	226	37.50	37.71	37.50	143	4	CON	EXERCISE1	40	50
	227	37.50	37.71	37.53	144	4	CON	EXERCISE1	40	50
	228	37.51	37.73	37.54	144	4	CON	EXERCISE1	40	50
	229	37.53	37.75	37.52	144	4	CON	EXERCISE1	40	50
	230	37.54	37.70	37.52	147	4	CON	EXERCISE1	40	50
	231	37.55	37.68	37.55	144	4	CON	EXERCISE1	40	50
	232	37.55	37.74	37.56	143	4	CON	EXERCISE1	40	50
	233	37.56	37.76	37.55	145	4	CON	EXERCISE1	40	50
	234	37.56	37.76	37.56	147	4	CON	EXERCISE1	40	50
	235	37.57	37.76	37.59	146	4	CON	EXERCISE1	40	50
	236	37.58	37.75	37.59	146	4	CON	EXERCISE1	40	50
	237	37.57	37.76	37.57	145	4	CON	EXERCISE1	40	50
	238	37.57	37.84	37.57	161	4	CON	EXERCISE1	40	50
	239	37.57	37.84	37.60	145	4	CON	EXERCISE1	40	50
	240	37.58	37.79	37.60	146	4	CON	EXERCISE1	40	50
40	241	37.59	37.82	37.61	146	4	CON	EXERCISE1	40	50
	242	37.59	37.85	37.60	147	4	CON	EXERCISE1	40	50
	243	37.60	37.87	37.59	149	4	CON	EXERCISE1	40	50
	244	37.61	37.86	37.61	171	4	CON	EXERCISE1	40	50
	245	37.61	37.86	37.62	148	4	CON	EXERCISE1	40	50
	246	37.62	37.88	37.65	147	4	CON	EXERCISE1	40	50
	247	37.62	37.89	37.67	149	4	CON	EXERCISE1	40	50
	248	37.63	37.90	37.67	149	4	CON	EXERCISE1	40	50
	249	37.64	37.91	37.68	149	4	CON	EXERCISE1	40	50
	250	37.65	37.93	37.68	149	4	CON	EXERCISE1	40	50
	251	37.66	37.95	37.68	150	4	CON	EXERCISE1	40	50
	252	37.67	37.95	37.69	150	4	CON	EXERCISE1	40	50
	253	37.68	37.96	37.68	150	4	CON	EXERCISE1	40	50
	254	37.69	37.98	37.66	148	4	CON	EXERCISE1	40	50
	255	37.70	37.99	37.67	146	4	CON	EXERCISE1	40	50
	256	37.70	37.98	37.70	149	4	CON	EXERCISE1	40	50
	257	37.71	37.99	37.69	149	4	CON	EXERCISE1	40	50
	258	37.72	38.01	37.71	148	4	CON	EXERCISE1	40	50
	259	37.72	38.01	37.72	149	4	CON	EXERCISE1	40	50
	260	37.73	38.03	37.72	149	4	CON	EXERCISE1	40	50
	261	37.73	38.04	37.75	148	4	CON	EXERCISE1	40	50
	262	37.74	37.99	37.74	151	4	CON	EXERCISE1	40	50
	263	37.75	37.97	37.74	150	4	CON	EXERCISE1	40	50
	264	37.75	38.01	37.76	151	4	CON	EXERCISE1	40	50
	265	37.76	38.04	37.76	152	4	CON	EXERCISE1	40	50

min	number	Tre	Tes	Tsk-head	HR	ID	condition	period	Ta	RH
	266	37.77	38.07	37.75	149	4	CON	EXERCISE1	40	50
	267	37.78	38.08	37.75	152	4	CON	EXERCISE1	40	50
	268	37.79	38.09	37.75	149	4	CON	EXERCISE1	40	50
	269	37.79	38.04	37.75	151	4	CON	EXERCISE1	40	50
	270	37.80	38.03	37.75	150	4	CON	EXERCISE1	40	50
45	271	37.81	38.09	37.76	152	4	CON	EXERCISE1	40	50
	272	37.83	38.11	37.80	150	4	CON	EXERCISE1	40	50
	273	37.83	38.11	37.83	152	4	CON	EXERCISE1	40	50
	274	37.83	38.11	37.83	149	4	CON	EXERCISE1	40	50
	275	37.84	38.13	37.83	148	4	CON	EXERCISE1	40	50
	276	37.84	38.15	37.84	149	4	CON	EXERCISE1	40	50
	277	37.85	38.17	37.83	152	4	CON	EXERCISE1	40	50
	278	37.86	38.19	37.80	146	4	CON	EXERCISE1	40	50
	279	37.86	38.19	37.80	144	4	CON	EXERCISE1	40	50
	280	37.87	38.18	37.85	172	4	CON	EXERCISE1	40	50
	281	37.89	38.15	37.86	154	4	CON	EXERCISE1	40	50
	282	37.89	38.13	37.87	153	4	CON	EXERCISE1	40	50
	283	37.89	38.17	37.88	154	4	CON	EXERCISE1	40	50
	284	37.89	38.19	37.87	156	4	CON	EXERCISE1	40	50
	285	37.90	38.20	37.89	156	4	CON	EXERCISE1	40	50
	286	37.91	38.21	37.92	156	4	CON	EXERCISE1	40	50
	287	37.92	38.21	37.95	157	4	CON	EXERCISE1	40	50
	288	37.92	38.23	37.95	155	4	CON	EXERCISE1	40	50
	289	37.93	38.25	37.93	155	4	CON	EXERCISE1	40	50
	290	37.94	38.25	37.95	154	4	CON	EXERCISE1	40	50
	291	37.95	38.24	37.99	157	4	CON	EXERCISE1	40	50
	292	37.96	38.24	37.97	157	4	CON	EXERCISE1	40	50
	293	37.97	38.24	37.97	158	4	CON	EXERCISE1	40	50
	294	37.97	38.25	38.00	157	4	CON	EXERCISE1	40	50
	295	37.98	38.28	38.00	153	4	CON	EXERCISE1	40	50
	296	37.98	38.30	37.98	154	4	CON	EXERCISE1	40	50
	297	37.99	38.30	37.99	155	4	CON	EXERCISE1	40	50
	298	38.00	38.31	38.02	155	4	CON	EXERCISE1	40	50
	299	38.01	38.32	38.04	154	4	CON	EXERCISE1	40	50
	300	38.01	38.32	38.05	156	4	CON	EXERCISE1	40	50
50	301	38.02	38.33	38.03	156	4	CON	EXERCISE1	40	50
	302	38.02	38.35	38.01	154	4	CON	EXERCISE1	40	50
	303	38.03	38.36	38.01	158	4	CON	EXERCISE1	40	50
	304	38.04	38.36	38.01	155	4	CON	EXERCISE1	40	50
	305	38.05	38.31	38.03	156	4	CON	EXERCISE1	40	50
	306	38.06	38.29	38.04	154	4	CON	EXERCISE1	40	50
	307	38.06	38.34	38.04	157	4	CON	EXERCISE1	40	50
	308	38.07	38.35	38.05	155	4	CON	EXERCISE1	40	50
	309	38.07	38.38	38.08	157	4	CON	EXERCISE1	40	50
	310	38.08	38.42	38.08	157	4	CON	EXERCISE1	40	50
	311	38.09	38.42	38.05	160	4	CON	EXERCISE1	40	50

min	number	Tre	Tes	Tsk-head	HR	ID	condition	period	Ta	RH
	312	38.09	38.41	38.08	158	4	CON	EXERCISE1	40	50
	313	38.10	38.44	38.09	156	4	CON	EXERCISE1	40	50
	314	38.11	38.45	38.08	153	4	CON	EXERCISE1	40	50
	315	38.11	38.46	38.09	154	4	CON	EXERCISE1	40	50
	316	38.12	38.48	38.10	153	4	CON	EXERCISE1	40	50
	317	38.13	38.48	38.12	154	4	CON	EXERCISE1	40	50
	318	38.14	38.47	38.14	154	4	CON	EXERCISE1	40	50
	319	38.15	38.48	38.12	153	4	CON	EXERCISE1	40	50
	320	38.15	38.49	38.10	153	4	CON	REST2	28	50
	321	38.16	38.47	38.08	150	4	CON	REST2	28	50
	322	38.17	38.48	38.09	149	4	CON	REST2	28	50
	323	38.18	38.46	37.94	143	4	CON	REST2	28	50
	324	38.19	38.44	37.67	143	4	CON	REST2	28	50
	325	38.21	38.46	37.58	142	4	CON	REST2	28	50
	326	38.21	38.44	37.48	141	4	CON	REST2	28	50
	327	38.22	38.45	37.38	133	4	CON	REST2	28	50
	328	38.22	38.46	37.38	136	4	CON	REST2	28	50
	329	38.22	38.44	37.31	131	4	CON	REST2	28	50
	330	38.23	38.43	37.29	128	4	CON	REST2	28	50
55	331	38.23	38.42	37.29	130	4	CON	REST2	28	50
	332	38.23	38.38	37.22	133	4	CON	REST2	28	50
	333	38.23	38.34	37.13	131	4	CON	REST2	28	50
	334	38.22	38.33	37.07	135	4	CON	REST2	28	50
	335	38.23	38.26	37.04	132	4	CON	REST2	28	50
	336	38.24	38.21	36.99	133	4	CON	REST2	28	50
	337	38.24	38.25	37.00	128	4	CON	REST2	28	50
	338	38.23	33.08	37.05	132	4	CON	REST2	28	50
	339	38.23	27.72	37.05	135	4	CON	REST2	28	50
	340	38.22	29.16	37.03	133	4	CON	REST2	28	50
	341	38.22	31.05	36.99	129	4	CON	REST2	28	50
	342	38.22	29.88	36.93	130	4	CON	REST2	28	50
	343	38.20	29.54	36.88	126	4	CON	REST2	28	50
	344	38.20	30.47	36.83	128	4	CON	REST2	28	50
	345	38.21	29.68	36.79	124	4	CON	REST2	28	50
	346	38.20	29.67	36.70	125	4	CON	REST2	28	50
	347	38.19	31.06	36.61	125	4	CON	REST2	28	50
	348	38.20	32.21	36.61	124	4	CON	REST2	28	50
	349	38.20	33.22	36.57	125	4	CON	REST2	28	50
	350	38.19	34.02	36.49	121	4	CON	REST2	28	50
	351	38.19	33.83	36.45	130	4	CON	REST2	28	50
	352	38.18	33.87	36.40	120	4	CON	REST2	28	50
	353	38.17	34.46	36.39	119	4	CON	REST2	28	50
	354	38.17	34.89	36.39	119	4	CON	REST2	28	50
	355	38.16	35.48	36.37	123	4	CON	REST2	28	50
	356	38.16	35.89	36.36	119	4	CON	REST2	28	50
	357	38.16	36.12	36.37	120	4	CON	REST2	28	50

min	number	Tre	Tes	Tsk-head	HR	ID	condition	period	Ta	RH
	358	38.15	36.33	36.30	138	4	CON	REST2	28	50
	359	38.15	36.49	36.25	121	4	CON	REST2	28	50
	360	38.13	36.61	36.21	117	4	CON	REST2	28	50
60	361	38.12	36.64	36.15	121	4	CON	REST2	28	50
	362	38.11	36.82	36.18	115	4	CON	REST2	28	50
	363	38.10	37.02	36.22	115	4	CON	REST2	28	50
	364	38.10	37.10	36.21	113	4	CON	REST2	28	50
	365	38.10	37.16	36.21	113	4	CON	REST2	28	50
	366	38.09	37.21	36.19	114	4	CON	REST2	28	50
	367	38.08	37.21	36.09	115	4	CON	REST2	28	50
	368	38.08	37.22	36.01	116	4	CON	REST2	28	50
	369	38.07	37.25	35.97	113	4	CON	REST2	28	50
	370	38.06	37.27	35.92	114	4	CON	REST2	28	50
	371	38.06	37.29	35.89	111	4	CON	REST2	28	50
	372	38.06	37.30	35.85	112	4	CON	REST2	28	50
	373	38.06	37.27	35.82	119	4	CON	REST2	28	50
	374	38.05	37.27	35.84	114	4	CON	REST2	28	50
	375	38.04	37.32	35.89	113	4	CON	REST2	28	50
	376	38.03	37.34	35.92	112	4	CON	REST2	28	50
	377	38.03	37.34	35.89	114	4	CON	REST2	28	50
	378	38.02	37.36	35.86	112	4	CON	REST2	28	50
	379	38.02	37.37	35.84	112	4	CON	REST2	28	50
	380	38.01	37.35	35.82	112	4	CON	REST2	28	50
	381	38.01	37.34	35.82	116	4	CON	REST2	28	50
	382	38.00	37.34	35.82	114	4	CON	REST2	28	50
	383	37.99	37.33	35.77	111	4	CON	REST2	28	50
	384	37.99	37.31	35.76	106	4	CON	REST2	28	50
	385	37.98	37.30	35.74	108	4	CON	REST2	28	50
	386	37.97	37.31	35.71	111	4	CON	REST2	28	50
	387	37.97	37.32	35.72	108	4	CON	REST2	28	50
	388	37.96	37.31	35.71	108	4	CON	REST2	28	50
	389	37.96	37.31	35.70	108	4	CON	REST2	28	50
	390	37.96	37.32	35.68	110	4	CON	REST2	28	50
65	391	37.95	37.31	35.66	108	4	CON	REST2	28	50
	392	37.94	37.29	35.65	108	4	CON	REST2	28	50
	393	37.93	37.30	35.68	108	4	CON	REST2	28	50
	394	37.92	37.29	35.69	106	4	CON	REST2	28	50
	395	37.91	37.28	35.68	105	4	CON	REST2	28	50
	396	37.90	37.28	35.67	106	4	CON	REST2	28	50
	397	37.90	37.28	35.66	105	4	CON	REST2	28	50
	398	37.89	37.29	35.63	105	4	CON	REST2	28	50
	399	37.88	37.25	35.63	105	4	CON	REST2	28	50
	400	37.88	37.23	35.61	106	4	CON	REST2	28	50
	401	37.87	37.26	35.59	103	4	CON	REST2	28	50
	402	37.87	37.26	35.59	102	4	CON	REST2	28	50
	403	37.86	37.28	35.58	102	4	CON	REST2	28	50

min	number	Tre	Tes	Tsk-head	HR	ID	condition	period	Ta	RH
	404	37.85	37.28	35.59	103	4	CON	REST2	28	50
	405	37.84	37.27	35.58	104	4	CON	REST2	28	50
	406	37.84	37.28	35.56	101	4	CON	REST2	28	50
	407	37.84	37.30	35.55	100	4	CON	REST2	28	50
	408	37.84	37.30	35.55	101	4	CON	REST2	28	50
	409	37.83	37.28	35.56	101	4	CON	REST2	28	50
	410	37.83	37.28	35.59	101	4	CON	REST2	28	50
	411	37.83	37.28	35.62	101	4	CON	REST2	28	50
	412	37.82	37.29	35.59	101	4	CON	REST2	28	50
	413	37.81	37.30	35.57	102	4	CON	REST2	28	50
	414	37.80	37.31	35.57	100	4	CON	REST2	28	50
	415	37.80	37.31	35.61	101	4	CON	REST2	28	50
	416	37.79	37.30	35.62	98	4	CON	REST2	28	50
	417	37.79	37.31	35.61	98	4	CON	REST2	28	50
	418	37.79	37.31	35.60	100	4	CON	REST2	28	50
	419	37.78	37.30	35.59	102	4	CON	REST2	28	50
	420	37.78	37.29	35.55	104	4	CON	REST2	28	50
70	421	37.79	37.29	35.59	109	4	CON	REST2	28	50
	422	37.79	37.30	35.70	106	4	CON	REST2	28	50
	423	37.78	37.30	35.68	108	4	CON	REST2	28	50
	424	37.77	37.27	35.57	109	4	CON	REST2	28	50
	425	37.77	37.25	35.55	101	4	CON	REST2	28	50
	426	37.77	37.13	35.51	101	4	CON	REST2	28	50
	427	37.75	37.06	35.50	102	4	CON	REST2	28	50
	428	37.76	37.17	35.57	110	4	CON	REST2	28	50
	429	37.77	37.22	35.57	110	4	CON	REST2	28	50
	430	37.77	37.23	35.58	104	4	CON	REST2	28	50
	431	37.77	37.26	35.64	105	4	CON	REST2	28	50
	432	37.76	37.26	35.62	103	4	CON	REST2	28	50
	433	37.75	37.24	35.58	101	4	CON	REST2	28	50
	434	37.75	37.25	35.60	102	4	CON	REST2	28	50
	435	37.74	37.22	35.59	100	4	CON	REST2	28	50
	436	37.73	37.15	35.58	101	4	CON	REST2	28	50
	437	37.72	37.13	35.60	99	4	CON	REST2	28	50
	438	37.71	37.17	35.59	98	4	CON	REST2	28	50
	439	37.73	37.18	35.69	100	4	CON	REST2	40	50
	440	37.73	37.13	35.84	104	4	CON	REST2	40	50
	441	37.70	37.11	36.02	107	4	CON	REST2	40	50
	442	37.70	37.15	36.19	104	4	CON	REST2	40	50
	443	37.69	37.15	36.24	104	4	CON	REST2	40	50
	444	37.69	37.13	36.33	104	4	CON	REST2	40	50
	445	37.70	37.12	36.40	103	4	CON	REST2	40	50
	446	37.70	37.12	36.40	101	4	CON	REST2	40	50
	447	37.70	37.11	36.42	101	4	CON	REST2	40	50
	448	37.70	37.10	36.48	98	4	CON	REST2	40	50
	449	37.69	37.06	36.52	104	4	CON	REST2	40	50

min	number	Tre	Tes	Tsk-head	HR	ID	condition	period	Ta	RH
	450	37.68	37.04	36.56	102	4	CON	REST2	40	50
75	451	37.67	37.05	36.56	104	4	CON	REST2	40	50
	452	37.67	37.06	36.55	97	4	CON	REST2	40	50
	453	37.67	37.07	36.58	96	4	CON	REST2	40	50
	454	37.67	37.05	36.62	99	4	CON	REST2	40	50
	455	37.66	37.06	36.61	101	4	CON	REST2	40	50
	456	37.65	37.05	36.62	101	4	CON	REST2	40	50
	457	37.66	37.03	36.61	106	4	CON	REST2	40	50
	458	37.67	37.03	36.55	110	4	CON	REST2	40	50
	459	37.68	37.01	36.56	105	4	CON	REST2	40	50
	460	37.68	37.01	36.59	107	4	CON	REST2	40	50
	461	37.68	37.03	36.61	109	4	CON	REST2	40	50
	462	37.67	37.04	36.61	109	4	CON	REST2	40	50
	463	37.67	37.05	36.60	105	4	CON	EXERCISE2	40	50
	464	37.68	37.07	36.64	110	4	CON	EXERCISE2	40	50
	465	37.68	37.09	36.66	116	4	CON	EXERCISE2	40	50
	466	37.68	37.10	36.67	116	4	CON	EXERCISE2	40	50
	467	37.68	37.05	36.67	126	4	CON	EXERCISE2	40	50
	468	37.68	37.04	36.70	127	4	CON	EXERCISE2	40	50
	469	37.68	37.10	36.72	132	4	CON	EXERCISE2	40	50
	470	37.68	37.13	36.74	132	4	CON	EXERCISE2	40	50
	471	37.68	37.17	36.79	131	4	CON	EXERCISE2	40	50
	472	37.69	37.20	36.80	137	4	CON	EXERCISE2	40	50
	473	37.69	37.13	36.83	141	4	CON	EXERCISE2	40	50
	474	37.70	37.11	36.88	142	4	CON	EXERCISE2	40	50
	475	37.70	37.20	36.92	143	4	CON	EXERCISE2	40	50
	476	37.70	37.29	36.94	141	4	CON	EXERCISE2	40	50
	477	37.70	37.29	36.98	145	4	CON	EXERCISE2	40	50
	478	37.70	37.32	37.01	143	4	CON	EXERCISE2	40	50
	479	37.71	37.39	37.03	144	4	CON	EXERCISE2	40	50
	480	37.71	37.39	37.08	144	4	CON	EXERCISE2	40	50
80	481	37.72	37.43	37.09	141	4	CON	EXERCISE2	40	50
	482	37.73	37.46	37.09	142	4	CON	EXERCISE2	40	50
	483	37.73	37.47	37.12	161	4	CON	EXERCISE2	40	50
	484	37.73	37.48	37.16	143	4	CON	EXERCISE2	40	50
	485	37.73	37.50	37.18	142	4	CON	EXERCISE2	40	50
	486	37.74	37.52	37.19	145	4	CON	EXERCISE2	40	50
	487	37.75	37.54	37.20	143	4	CON	EXERCISE2	40	50
	488	37.76	37.54	37.22	146	4	CON	EXERCISE2	40	50
	489	37.76	37.54	37.23	145	4	CON	EXERCISE2	40	50
	490	37.76	37.56	37.25	149	4	CON	EXERCISE2	40	50
	491	37.77	37.59	37.27	149	4	CON	EXERCISE2	40	50
	492	37.78	37.60	37.29	162	4	CON	EXERCISE2	40	50
	493	37.79	37.62	37.30	146	4	CON	EXERCISE2	40	50
	494	37.79	37.64	37.31	168	4	CON	EXERCISE2	40	50
	495	37.79	37.67	37.31	148	4	CON	EXERCISE2	40	50

min	number	Tre	Tes	Tsk-head	HR	ID	condition	period	Ta	RH
	496	37.80	37.69	37.31	147	4	CON	EXERCISE2	40	50
	497	37.81	37.71	37.31	146	4	CON	EXERCISE2	40	50
	498	37.82	37.68	37.32	149	4	CON	EXERCISE2	40	50
	499	37.82	37.67	37.35	170	4	CON	EXERCISE2	40	50
	500	37.83	37.71	37.38	152	4	CON	EXERCISE2	40	50
	501	37.84	37.73	37.40	149	4	CON	EXERCISE2	40	50
	502	37.84	37.73	37.41	148	4	CON	EXERCISE2	40	50
	503	37.84	37.56	37.44	144	4	CON	EXERCISE2	40	50
	504	37.85	37.56	37.45	144	4	CON	EXERCISE2	40	50
	505	37.85	37.74	37.46	149	4	CON	EXERCISE2	40	50
	506	37.86	37.76	37.47	148	4	CON	EXERCISE2	40	50
	507	37.86	37.78	37.47	149	4	CON	EXERCISE2	40	50
	508	37.86	37.80	37.47	149	4	CON	EXERCISE2	40	50
	509	37.88	37.81	37.46	151	4	CON	EXERCISE2	40	50
	510	37.89	37.82	37.47	153	4	CON	EXERCISE2	40	50
85	511	37.89	37.85	37.50	153	4	CON	EXERCISE2	40	50
	512	37.90	37.86	37.53	151	4	CON	EXERCISE2	40	50
	513	37.91	37.87	37.55	150	4	CON	EXERCISE2	40	50
	514	37.91	37.88	37.55	150	4	CON	EXERCISE2	40	50
	515	37.92	37.91	37.54	151	4	CON	EXERCISE2	40	50
	516	37.93	37.93	37.54	152	4	CON	EXERCISE2	40	50
	517	37.93	37.93	37.57	151	4	CON	EXERCISE2	40	50
	518	37.94	37.93	37.59	153	4	CON	EXERCISE2	40	50
	519	37.94	37.94	37.59	153	4	CON	EXERCISE2	40	50
	520	37.94	37.98	37.60	152	4	CON	EXERCISE2	40	50
	521	37.96	37.97	37.63	153	4	CON	EXERCISE2	40	50
	522	37.96	37.95	37.66	154	4	CON	EXERCISE2	40	50
	523	37.96	37.96	37.66	154	4	CON	EXERCISE2	40	50
	524	37.97	38.00	37.65	154	4	CON	EXERCISE2	40	50
	525	37.98	38.03	37.65	155	4	CON	EXERCISE2	40	50
	526	37.99	38.02	37.67	153	4	CON	EXERCISE2	40	50
	527	37.98	37.96	37.67	157	4	CON	EXERCISE2	40	50
	528	37.99	37.97	37.69	154	4	CON	EXERCISE2	40	50
	529	38.00	38.04	37.72	153	4	CON	EXERCISE2	40	50
	530	38.01	38.06	37.73	152	4	CON	EXERCISE2	40	50
	531	38.01	38.06	37.76	150	4	CON	EXERCISE2	40	50
	532	38.01	38.04	37.77	151	4	CON	EXERCISE2	40	50
	533	38.03	38.05	37.78	150	4	CON	EXERCISE2	40	50
	534	38.03	38.08	37.79	147	4	CON	EXERCISE2	40	50
	535	38.03	38.08	37.81	153	4	CON	EXERCISE2	40	50
	536	38.04	38.10	37.84	153	4	CON	EXERCISE2	40	50
	537	38.05	38.11	37.85	156	4	CON	EXERCISE2	40	50
	538	38.06	38.09	37.83	160	4	CON	EXERCISE2	40	50
	539	38.06	38.08	37.84	159	4	CON	EXERCISE2	40	50
	540	38.06	38.11	37.88	160	4	CON	EXERCISE2	40	50
90	541	38.07	38.15	37.91	159	4	CON	EXERCISE2	40	50

min	number	Tre	Tes	Tsk-head	HR	ID	condition	period	Ta	RH
	542	38.08	38.19	37.91	160	4	CON	EXERCISE2	40	50
	543	38.09	38.15	37.91	161	4	CON	EXERCISE2	40	50
	544	38.09	38.14	37.91	159	4	CON	EXERCISE2	40	50
	545	38.10	38.18	37.91	157	4	CON	EXERCISE2	40	50
	546	38.10	38.19	37.93	157	4	CON	EXERCISE2	40	50
	547	38.11	38.20	37.97	157	4	CON	EXERCISE2	40	50
	548	38.12	38.22	37.99	156	4	CON	EXERCISE2	40	50
	549	38.12	38.24	38.00	155	4	CON	EXERCISE2	40	50
	550	38.13	38.25	38.01	155	4	CON	EXERCISE2	40	50
	551	38.14	38.25	38.02	155	4	CON	EXERCISE2	40	50
	552	38.15	38.26	38.02	152	4	CON	EXERCISE2	40	50
	553	38.15	38.27	38.01	156	4	CON	EXERCISE2	40	50
	554	38.15	38.29	37.99	158	4	CON	EXERCISE2	40	50
	555	38.16	38.31	37.99	157	4	CON	EXERCISE2	40	50
	556	38.16	38.32	38.00	158	4	CON	EXERCISE2	40	50
	557	38.17	38.34	38.02	158	4	CON	EXERCISE2	40	50
	558	38.18	38.35	38.02	159	4	CON	EXERCISE2	40	50
	559	38.18	38.35	38.02	153	4	CON	EXERCISE2	40	50
	560	38.18	38.34	38.04	159	4	CON	EXERCISE2	40	50
	561	38.19	38.26	38.05	156	4	CON	EXERCISE2	40	50
	562	38.20	38.23	38.06	158	4	CON	EXERCISE2	40	50
	563	38.20	38.31	38.06	156	4	CON	EXERCISE2	40	50
	564	38.20	38.34	38.08	157	4	CON	EXERCISE2	40	50
	565	38.21	38.36	38.11	158	4	CON	EXERCISE2	40	50
	566	38.22	38.38	38.10	157	4	CON	EXERCISE2	40	50
	567	38.23	38.38	38.12	157	4	CON	EXERCISE2	40	50
	568	38.23	38.34	38.11	155	4	CON	EXERCISE2	40	50
	569	38.24	38.35	38.09	157	4	CON	EXERCISE2	40	50
	570	38.25	38.41	38.10	158	4	CON	EXERCISE2	40	50
95	571	38.25	38.44	38.09	160	4	CON	EXERCISE2	40	50
	572	38.26	38.39	38.10	156	4	CON	EXERCISE2	40	50
	573	38.27	38.36	38.13	158	4	CON	EXERCISE2	40	50
	574	38.27	38.42	38.13	158	4	CON	EXERCISE2	40	50
	575	38.28	38.43	38.14	162	4	CON	EXERCISE2	40	50
	576	38.28	38.45	38.16	159	4	CON	EXERCISE2	40	50
	577	38.29	38.48	38.18	160	4	CON	EXERCISE2	40	50
	578	38.30	38.49	38.21	159	4	CON	EXERCISE2	40	50
	579	38.31	38.50	38.21	159	4	CON	EXERCISE2	40	50
	580	38.32	38.51	38.21	159	4	CON	EXERCISE2	40	50
	581	38.32	38.53	38.21	161	4	CON	EXERCISE2	40	50
	582	38.33	38.56	38.23	162	4	CON	EXERCISE2	40	50
	583	38.33	38.57	38.25	161	4	CON	EXERCISE2	40	50
	584	38.34	38.53	38.25	162	4	CON	EXERCISE2	40	50
	585	38.35	38.50	38.26	159	4	CON	EXERCISE2	40	50
	586	38.35	38.52	38.27	159	4	CON	EXERCISE2	40	50
	587	38.36	38.56	38.25	161	4	CON	EXERCISE2	40	50

min	number	Tre	Tes	Tsk-head	HR	ID	condition	period	Ta	RH
	588	38.37	38.57	38.26	161	4	CON	EXERCISE2	40	50
	589	38.38	38.58	38.28	161	4	CON	EXERCISE2	40	50
	590	38.38	38.59	38.29	162	4	CON	EXERCISE2	40	50
	591	38.38	38.60	38.29	161	4	CON	EXERCISE2	40	50
	592	38.39	38.62	38.30	161	4	CON	EXERCISE2	40	50
	593	38.40	38.59	38.33	163	4	CON	EXERCISE2	40	50
	594	38.41	38.59	38.30	163	4	CON	EXERCISE2	40	50
	595	38.42	38.62	38.30	162	4	CON	EXERCISE2	40	50
	596	38.42	38.64	38.32	163	4	CON	EXERCISE2	40	50
	597	38.42	38.66	38.33	163	4	CON	EXERCISE2	40	50
	598	38.43	38.66	38.36	161	4	CON	EXERCISE2	40	50
	599	38.44	38.67	38.38	161	4	CON	EXERCISE2	40	50
	600	38.45	38.68	38.38	163	4	CON	EXERCISE2	40	50
100	601	38.46	38.69	38.39	161	4	CON	EXERCISE2	40	50
	602	38.47	38.70	38.41	163	4	CON	EXERCISE2	40	50
	603	38.47	38.71	38.42	163	4	CON	EXERCISE2	40	50
	604	38.48	38.72	38.44	161	4	CON	EXERCISE2	40	50
	605	38.49	38.73	38.44	161	4	CON	EXERCISE2	40	50
	606	38.50	38.73	38.44	161	4	CON	EXERCISE2	40	50
	607	38.50	38.74	38.42	161	4	CON	EXERCISE2	40	50
	608	38.51	38.74	38.41	165	4	CON	EXERCISE2	40	50
	609	38.52	38.72	38.40	166	4	CON	EXERCISE2	40	50
	610	38.53	38.71	38.41	164	4	CON	EXERCISE2	40	50
	611	38.53	38.74	38.43	161	4	CON	EXERCISE2	40	50
	612	38.54	38.77	38.41	161	4	CON	EXERCISE2	40	50
	613	38.55	38.79	38.42	163	4	CON	EXERCISE2	40	50
	614	38.55	38.79	38.45	164	4	CON	EXERCISE2	40	50
	615	38.56	38.81	38.43	165	4	CON	EXERCISE2	40	50
	616	38.56	38.83	38.43	167	4	CON	EXERCISE2	40	50
	617	38.57	38.75	38.44	169	4	CON	EXERCISE2	40	50
	618	38.58	38.73	38.46	168	4	CON	EXERCISE2	40	50
	619	38.59	38.82	38.46	167	4	CON	EXERCISE2	40	50
	620	38.59	38.85	38.46	167	4	CON	EXERCISE2	40	50
	621	38.60	38.85	38.48	162	4	CON	EXERCISE2	40	50
	622	38.60	38.86	38.52	162	4	CON	EXERCISE2	40	50
	623	38.61	38.88	38.52	163	4	CON	EXERCISE2	40	50
	624	38.62	38.91	38.50	166	4	CON	EXERCISE2	40	50
	625	38.63	38.91	38.51	163	4	CON	EXERCISE2	40	50
	626	38.64	38.91	38.53	166	4	CON	EXERCISE2	40	50
	627	38.65	38.86	38.54	166	4	CON	EXERCISE2	40	50
	628	38.65	38.85	38.57	165	4	CON	EXERCISE2	40	50
	629	38.65	38.90	38.59	165	4	CON	EXERCISE2	40	50
	630	38.66	38.91	38.58	166	4	CON	EXERCISE2	40	50
105	631	38.68	38.93	38.59	170	4	CON	EXERCISE2	40	50
	632	38.69	38.95	38.60	170	4	CON	EXERCISE2	40	50
	633	38.70	38.91	38.60	170	4	CON	EXERCISE2	40	50

min	number	Tre	Tes	Tsk-head	HR	ID	condition	period	Ta	RH
	634	38.70	38.91	38.60	167	4	CON	EXERCISE2	40	50
	635	38.70	38.95	38.60	166	4	CON	EXERCISE2	40	50
	636	38.71	38.98	38.61	166	4	CON	EXERCISE2	40	50
	637	38.72	39.00	38.60	165	4	CON	EXERCISE2	40	50
	638	38.72	39.02	38.60	161	4	CON	EXERCISE2	40	50
	639	38.73	39.01	38.63	166	4	CON	EXERCISE2	40	50
	640	38.73	38.99	38.66	166	4	CON	EXERCISE2	40	50
	641	38.74	38.99	38.68	166	4	CON	EXERCISE2	40	50
	642	38.75	39.01	38.70	163	4	CON	EXERCISE2	40	50
	643	38.76	39.03	38.71	165	4	CON	EXERCISE2	40	50
	644	38.76	39.04	38.72	158	4	CON	REST3	28	50
	645	38.77	39.05	38.66	161	4	CON	REST3	28	50
	646	38.78	39.05	38.51	157	4	CON	REST3	28	50
	647	38.79	39.05	38.30	153	4	CON	REST3	28	50
	648	38.80	39.09	38.06	153	4	CON	REST3	28	50
	649	38.80	39.10	37.96	148	4	CON	REST3	28	50
	650	38.81	39.09	37.93	142	4	CON	REST3	28	50
	651	38.82	39.03	37.95	142	4	CON	REST3	28	50
	652	38.83	38.95	37.95	137	4	CON	REST3	28	50
	653	38.84	38.92	37.93	134	4	CON	REST3	28	50
	654	38.84	38.91	37.90	136	4	CON	REST3	28	50
	655	38.84	38.88	37.85	134	4	CON	REST3	28	50
	656	38.84	38.85	37.84	132	4	CON	REST3	28	50
	657	38.84	38.90	37.75	135	4	CON	REST3	28	50
	658	38.84	38.95	37.69	137	4	CON	REST3	28	50
	659	38.84	38.92	37.63	133	4	CON	REST3	28	50
	660	38.83	38.88	37.57	131	4	CON	REST3	28	50
110	661	38.83	38.86	37.58	132	4	CON	REST3	28	50
	662	38.83	38.84	37.49	133	4	CON	REST3	28	50
	663	38.83	38.80	37.44	131	4	CON	REST3	28	50
	664	38.84	38.78	37.41	131	4	CON	REST3	28	50
	665	38.85	38.75	37.37	131	4	CON	REST3	28	50
	666	38.84	38.72	37.40	130	4	CON	REST3	28	50
	667	38.84	38.71	37.35	129	4	CON	REST3	28	50
	668	38.84	38.69	37.28	128	4	CON	REST3	28	50
	669	38.83	38.67	37.27	128	4	CON	REST3	28	50
	670	38.84	38.65	37.25	129	4	CON	REST3	28	50
	671	38.83	38.62	37.24	129	4	CON	REST3	28	50
	672	38.82	38.61	37.25	128	4	CON	REST3	28	50
	673	38.82	38.61	37.24	129	4	CON	REST3	28	50
	674	38.81	38.55	37.20	129	4	CON	REST3	28	50
	675	38.81	38.50	37.18	127	4	CON	REST3	28	50
	676	38.81	38.51	37.18	128	4	CON	REST3	28	50
	677	38.81	38.50	37.16	128	4	CON	REST3	28	50
	678	38.81	38.48	37.15	126	4	CON	REST3	28	50
	679	38.81	38.47	37.16	127	4	CON	REST3	28	50

min	number	Tre	Tes	Tsk-head	HR	ID	condition	period	Ta	RH
	680	38.81	38.42	37.14	125	4	CON	REST3	28	50
	681	38.80	38.40	37.10	128	4	CON	REST3	28	50
	682	38.80	38.41	37.01	127	4	CON	REST3	28	50
	683	38.79	38.33	36.97	128	4	CON	REST3	28	50
	684	38.78	38.32	36.95	124	4	CON	REST3	28	50
	685	38.79	38.38	36.86	123	4	CON	REST3	28	50
	686	38.79	38.46	36.78	124	4	CON	REST3	28	50
	687	38.79	38.50	36.77	127	4	CON	REST3	28	50
	688	38.79	38.40	36.83	123	4	CON	REST3	28	50
	689	38.78	38.32	36.81	123	4	CON	REST3	28	50
	690	38.77	38.29	36.74	126	4	CON	REST3	28	50
115	691	38.76	38.28	36.74	123	4	CON	REST3	28	50
	692	38.75	38.27	36.74	123	4	CON	REST3	28	50
	693	38.75	38.26	36.73	122	4	CON	REST3	28	50
	694	38.75	38.26	36.73	123	4	CON	REST3	28	50
	695	38.74	38.22	36.71	124	4	CON	REST3	28	50
	696	38.74	38.18	36.68	123	4	CON	REST3	28	50
	697	38.74	38.19	36.65	126	4	CON	REST3	28	50
	698	38.74	38.21	36.67	126	4	CON	REST3	28	50
	699	38.73	38.21	36.68	123	4	CON	REST3	28	50
	700	38.72	38.19	36.63	122	4	CON	REST3	28	50
	701	38.72	38.19	36.66	123	4	CON	REST3	28	50
	702	38.73	38.18	36.68	123	4	CON	REST3	28	50
	703	38.72	38.17	36.65	120	4	CON	REST3	28	50
	704	38.72	38.16	36.66	120	4	CON	REST3	28	50
	705	38.71	38.15	36.64	120	4	CON	REST3	28	50
	706	38.71	38.15	36.65	120	4	CON	REST3	28	50
	707	38.72	38.14	36.65	120	4	CON	REST3	28	50
	708	38.71	38.12	36.61	118	4	CON	REST3	28	50
	709	38.71	38.11	36.59	121	4	CON	REST3	28	50
0	1	37.00	36.76	35.39	90	5	CON	REST1	28	50
	2	37.00	36.78	35.36	87	5	CON	REST1	28	50
	3	37.00	36.79	35.35	78	5	CON	REST1	28	50
	4	37.01	36.78	35.34	82	5	CON	REST1	28	50
	5	37.00	36.76	35.34	82	5	CON	REST1	28	50
	6	37.00	36.75	35.34	85	5	CON	REST1	28	50
	7	36.99	36.76	35.34	91	5	CON	REST1	28	50
	8	36.98	36.79	35.35	84	5	CON	REST1	28	50
	9	36.97	36.78	35.34	84	5	CON	REST1	28	50
	10	36.97	36.76	35.34	85	5	CON	REST1	28	50
	11	36.98	36.76	35.33	82	5	CON	REST1	28	50
	12	36.97	36.78	35.33	83	5	CON	REST1	28	50
	13	36.97	36.79	35.34	82	5	CON	REST1	28	50
	14	36.97	36.78	35.32	87	5	CON	REST1	28	50
	15	36.97	36.78	35.31	87	5	CON	REST1	28	50
	16	36.96	36.79	35.32	87	5	CON	REST1	28	50

min	number	Tre	Tes	Tsk-head	HR	ID	condition	period	Ta	RH
	17	36.96	36.78	35.33	82	5	CON	REST1	28	50
	18	36.95	36.76	35.33	86	5	CON	REST1	28	50
	19	36.95	36.77	35.32	88	5	CON	REST1	28	50
	20	36.96	36.78	35.32	93	5	CON	REST1	28	50
	21	36.97	36.76	35.31	83	5	CON	REST1	28	50
	22	36.96	36.73	35.30	82	5	CON	REST1	28	50
	23	36.96	36.73	35.28	84	5	CON	REST1	28	50
	24	36.96	36.75	35.29	82	5	CON	REST1	28	50
	25	36.96	36.75	35.29	92	5	CON	REST1	28	50
	26	36.96	36.75	35.31	87	5	CON	REST1	28	50
	27	36.96	36.75	35.32	93	5	CON	REST1	28	50
	28	36.95	36.76	35.30	90	5	CON	REST1	28	50
	29	36.96	36.78	35.30	91	5	CON	REST1	28	50
	30	36.96	36.76	35.33	87	5	CON	REST1	28	50
5	31	36.95	36.74	35.34	82	5	CON	REST1	28	50
	32	36.94	36.76	35.33	82	5	CON	REST1	28	50
	33	36.94	36.75	35.33	84	5	CON	REST1	28	50
	34	36.94	36.74	35.34	81	5	CON	REST1	28	50
	35	36.93	36.75	35.33	82	5	CON	REST1	28	50
	36	36.93	36.76	35.33	81	5	CON	REST1	28	50
	37	36.94	36.75	35.33	82	5	CON	REST1	28	50
	38	36.93	36.75	35.33	88	5	CON	REST1	28	50
	39	36.93	36.76	35.34	88	5	CON	REST1	28	50
	40	36.92	36.74	35.33	88	5	CON	REST1	28	50
	41	36.92	36.73	35.32	91	5	CON	REST1	28	50
	42	36.93	36.74	35.33	85	5	CON	REST1	28	50
	43	36.93	36.76	35.34	82	5	CON	REST1	28	50
	44	36.92	36.76	35.35	80	5	CON	REST1	28	50
	45	36.92	36.74	35.35	79	5	CON	REST1	28	50
	46	36.92	36.72	35.34	77	5	CON	REST1	28	50
	47	36.92	36.72	35.34	77	5	CON	REST1	28	50
	48	36.93	36.74	35.33	81	5	CON	REST1	28	50
	49	36.94	36.76	35.31	82	5	CON	REST1	28	50
	50	36.93	36.73	35.31	89	5	CON	REST1	28	50
	51	36.92	36.71	35.29	79	5	CON	REST1	28	50
	52	36.92	36.73	35.26	77	5	CON	REST1	28	50
	53	36.91	36.73	35.24	77	5	CON	REST1	28	50
	54	36.91	36.73	35.25	81	5	CON	REST1	28	50
	55	36.92	36.73	35.26	84	5	CON	REST1	28	50
	56	36.92	36.72	35.27	83	5	CON	REST1	28	50
	57	36.92	36.72	35.27	83	5	CON	REST1	28	50
	58	36.91	36.72	35.25	84	5	CON	REST1	28	50
	59	36.92	36.72	35.27	79	5	CON	REST1	28	50
	60	36.93	36.73	35.28	85	5	CON	REST1	28	50
10	61	36.93	36.75	35.28	81	5	CON	REST1	28	50
	62	36.92	36.74	35.29	88	5	CON	REST1	28	50

min	number	Tre	Tes	Tsk-head	HR	ID	condition	period	Ta	RH
	63	36.91	36.73	35.29	93	5	CON	REST1	28	50
	64	36.92	36.73	35.27	91	5	CON	REST1	28	50
	65	36.91	36.74	35.24	99	5	CON	REST1	28	50
	66	36.91	36.71	35.24	87	5	CON	REST1	28	50
	67	36.91	36.67	35.26	81	5	CON	REST1	28	50
	68	36.91	36.68	35.30	80	5	CON	REST1	28	50
	69	36.91	36.68	35.30	86	5	CON	REST1	28	50
	70	36.91	36.67	35.29	86	5	CON	REST1	28	50
	71	36.91	36.68	35.29	83	5	CON	REST1	28	50
	72	36.91	36.69	35.29	82	5	CON	REST1	28	50
	73	36.91	36.69	35.29	82	5	CON	REST1	28	50
	74	36.91	36.68	35.29	83	5	CON	REST1	28	50
	75	36.90	36.68	35.29	84	5	CON	REST1	28	50
	76	36.89	36.69	35.29	85	5	CON	REST1	28	50
	77	36.88	36.71	35.29	87	5	CON	REST1	28	50
	78	36.87	36.72	35.29	89	5	CON	REST1	28	50
	79	36.87	36.71	35.29	89	5	CON	REST1	28	50
	80	36.87	36.65	35.28	99	5	CON	REST1	28	50
	81	36.87	36.62	35.29	92	5	CON	REST1	28	50
	82	36.88	36.67	35.31	87	5	CON	REST1	28	50
	83	36.88	36.68	35.31	85	5	CON	REST1	28	50
	84	36.87	36.70	35.30	89	5	CON	REST1	28	50
	85	36.87	36.70	35.29	89	5	CON	REST1	28	50
	86	36.87	36.70	35.28	88	5	CON	REST1	28	50
	87	36.86	36.71	35.29	89	5	CON	REST1	28	50
	88	36.86	36.71	35.31	86	5	CON	REST1	28	50
	89	36.85	36.71	35.30	85	5	CON	REST1	28	50
	90	36.85	36.72	35.30	86	5	CON	REST1	28	50
15	91	36.86	36.74	35.30	88	5	CON	REST1	28	50
	92	36.86	36.74	35.31	87	5	CON	REST1	28	50
	93	36.85	36.72	35.32	89	5	CON	REST1	28	50
	94	36.85	36.70	35.32	87	5	CON	REST1	28	50
	95	36.86	36.72	35.29	94	5	CON	REST1	28	50
	96	36.85	36.74	35.25	89	5	CON	REST1	28	50
	97	36.84	36.74	35.25	97	5	CON	REST1	28	50
	98	36.84	36.75	35.25	107	5	CON	REST1	28	50
	99	36.84	36.75	35.23	110	5	CON	REST1	28	50
	100	36.84	36.74	35.24	101	5	CON	REST1	28	50
	101	36.83	36.74	35.29	99	5	CON	REST1	28	50
	102	36.83	36.73	35.30	99	5	CON	REST1	28	50
	103	36.84	36.74	35.28	97	5	CON	REST1	40	50
	104	36.85	36.74	35.48	101	5	CON	REST1	40	50
	105	36.84	36.67	35.73	104	5	CON	REST1	40	50
	106	36.85	36.66	35.84	101	5	CON	REST1	40	50
	107	36.87	36.68	35.92	104	5	CON	REST1	40	50
	108	36.86	36.66	35.99	100	5	CON	REST1	40	50

min	number	Tre	Tes	Tsk-head	HR	ID	condition	period	Ta	RH
	109	36.87	36.68	36.06	95	5	CON	REST1	40	50
	110	36.88	36.68	36.11	92	5	CON	REST1	40	50
	111	36.88	36.66	36.15	97	5	CON	REST1	40	50
	112	36.87	36.64	36.18	92	5	CON	REST1	40	50
	113	36.87	36.64	36.21	91	5	CON	REST1	40	50
	114	36.88	36.64	36.26	94	5	CON	REST1	40	50
	115	36.87	36.65	36.30	92	5	CON	REST1	40	50
	116	36.86	36.66	36.32	94	5	CON	REST1	40	50
	117	36.86	36.68	36.35	93	5	CON	REST1	40	50
	118	36.85	36.66	36.39	90	5	CON	REST1	40	50
	119	36.85	36.64	36.41	96	5	CON	REST1	40	50
	120	36.85	36.65	36.43	92	5	CON	REST1	40	50
20	121	36.85	36.66	36.46	90	5	CON	REST1	40	50
	122	36.85	36.66	36.49	83	5	CON	REST1	40	50
	123	36.85	36.68	36.53	91	5	CON	REST1	40	50
	124	36.86	36.67	36.55	84	5	CON	REST1	40	50
	125	36.85	36.66	36.55	87	5	CON	REST1	40	50
	126	36.85	36.68	36.57	85	5	CON	REST1	40	50
	127	36.85	36.68	36.59	87	5	CON	REST1	40	50
	128	36.85	36.69	36.62	94	5	CON	REST1	40	50
	129	36.84	36.68	36.64	89	5	CON	REST1	40	50
	130	36.84	36.67	36.65	94	5	CON	REST1	40	50
	131	36.84	36.68	36.67	92	5	CON	REST1	40	50
	132	36.84	36.69	36.70	100	5	CON	REST1	40	50
	133	36.84	36.67	36.72	99	5	CON	REST1	40	50
	134	36.84	36.66	36.75	111	5	CON	REST1	40	50
	135	36.83	36.68	36.76	92	5	CON	REST1	40	50
	136	36.82	36.68	36.77	90	5	CON	REST1	40	50
	137	36.82	36.71	36.78	103	5	CON	REST1	40	50
	138	36.82	36.73	36.79	102	5	CON	REST1	40	50
	139	36.81	36.73	36.79	98	5	CON	EXERCISE1	40	50
	140	36.81	36.74	36.80	100	5	CON	EXERCISE1	40	50
	141	36.82	36.66	36.82	112	5	CON	EXERCISE1	40	50
	142	36.82	36.63	36.83	110	5	CON	EXERCISE1	40	50
	143	36.83	36.69	36.84	111	5	CON	EXERCISE1	40	50
	144	36.83	36.68	36.85	115	5	CON	EXERCISE1	40	50
	145	36.83	36.69	36.86	117	5	CON	EXERCISE1	40	50
	146	36.84	36.70	36.87	118	5	CON	EXERCISE1	40	50
	147	36.85	36.72	36.88	119	5	CON	EXERCISE1	40	50
	148	36.85	36.73	36.88	119	5	CON	EXERCISE1	40	50
	149	36.85	36.69	36.89	120	5	CON	EXERCISE1	40	50
	150	36.85	36.67	36.91	117	5	CON	EXERCISE1	40	50
25	151	36.85	36.71	36.93	115	5	CON	EXERCISE1	40	50
	152	36.85	36.75	36.95	118	5	CON	EXERCISE1	40	50
	153	36.85	36.76	36.96	122	5	CON	EXERCISE1	40	50
	154	36.85	36.75	36.97	123	5	CON	EXERCISE1	40	50

min	number	Tre	Tes	Tsk-head	HR	ID	condition	period	Ta	RH
	155	36.85	36.76	36.98	123	5	CON	EXERCISE1	40	50
	156	36.84	36.76	36.97	126	5	CON	EXERCISE1	40	50
	157	36.83	36.75	36.98	125	5	CON	EXERCISE1	40	50
	158	36.83	36.76	37.00	125	5	CON	EXERCISE1	40	50
	159	36.84	36.78	37.00	125	5	CON	EXERCISE1	40	50
	160	36.85	36.80	37.00	126	5	CON	EXERCISE1	40	50
	161	36.85	36.79	37.00	128	5	CON	EXERCISE1	40	50
	162	36.86	36.80	37.00	127	5	CON	EXERCISE1	40	50
	163	36.87	36.82	36.99	127	5	CON	EXERCISE1	40	50
	164	36.87	36.82	36.99	123	5	CON	EXERCISE1	40	50
	165	36.86	36.81	37.00	126	5	CON	EXERCISE1	40	50
	166	36.87	36.82	37.02	124	5	CON	EXERCISE1	40	50
	167	36.87	36.83	37.03	126	5	CON	EXERCISE1	40	50
	168	36.86	36.85	37.03	129	5	CON	EXERCISE1	40	50
	169	36.86	36.88	37.04	129	5	CON	EXERCISE1	40	50
	170	36.86	36.88	37.03	129	5	CON	EXERCISE1	40	50
	171	36.86	36.90	37.03	130	5	CON	EXERCISE1	40	50
	172	36.87	36.91	37.04	128	5	CON	EXERCISE1	40	50
	173	36.87	36.91	37.05	126	5	CON	EXERCISE1	40	50
	174	36.86	36.93	37.03	126	5	CON	EXERCISE1	40	50
	175	36.87	36.94	37.03	130	5	CON	EXERCISE1	40	50
	176	36.87	36.93	37.05	134	5	CON	EXERCISE1	40	50
	177	36.87	36.92	37.05	134	5	CON	EXERCISE1	40	50
	178	36.88	36.94	37.05	135	5	CON	EXERCISE1	40	50
	179	36.88	36.97	37.07	137	5	CON	EXERCISE1	40	50
	180	36.89	36.99	37.09	136	5	CON	EXERCISE1	40	50
30	181	36.89	37.00	37.09	136	5	CON	EXERCISE1	40	50
	182	36.89	37.02	37.09	131	5	CON	EXERCISE1	40	50
	183	36.90	37.04	37.10	134	5	CON	EXERCISE1	40	50
	184	36.90	37.05	37.11	134	5	CON	EXERCISE1	40	50
	185	36.90	37.04	37.12	134	5	CON	EXERCISE1	40	50
	186	36.91	37.07	37.13	134	5	CON	EXERCISE1	40	50
	187	36.92	37.08	37.14	133	5	CON	EXERCISE1	40	50
	188	36.92	37.08	37.12	134	5	CON	EXERCISE1	40	50
	189	36.93	36.99	37.12	137	5	CON	EXERCISE1	40	50
	190	36.94	37.01	37.14	136	5	CON	EXERCISE1	40	50
	191	36.94	37.12	37.15	132	5	CON	EXERCISE1	40	50
	192	36.94	37.12	37.17	131	5	CON	EXERCISE1	40	50
	193	36.95	37.13	37.17	134	5	CON	EXERCISE1	40	50
	194	36.95	37.15	37.16	133	5	CON	EXERCISE1	40	50
	195	36.96	37.16	37.17	134	5	CON	EXERCISE1	40	50
	196	36.96	37.16	37.16	135	5	CON	EXERCISE1	40	50
	197	36.98	37.18	37.16	135	5	CON	EXERCISE1	40	50
	198	36.99	37.18	37.19	139	5	CON	EXERCISE1	40	50
	199	36.99	37.17	37.20	137	5	CON	EXERCISE1	40	50
	200	36.99	37.18	37.21	134	5	CON	EXERCISE1	40	50

min	number	Tre	Tes	Tsk-head	HR	ID	condition	period	Ta	RH
	201	36.99	37.20	37.22	136	5	CON	EXERCISE1	40	50
	202	36.99	37.20	37.22	136	5	CON	EXERCISE1	40	50
	203	36.99	37.23	37.21	137	5	CON	EXERCISE1	40	50
	204	36.99	37.23	37.21	137	5	CON	EXERCISE1	40	50
	205	37.00	37.23	37.23	132	5	CON	EXERCISE1	40	50
	206	37.01	37.25	37.24	138	5	CON	EXERCISE1	40	50
	207	37.01	37.22	37.25	144	5	CON	EXERCISE1	40	50
	208	37.02	37.22	37.25	145	5	CON	EXERCISE1	40	50
	209	37.02	37.24	37.25	146	5	CON	EXERCISE1	40	50
	210	37.02	37.25	37.26	146	5	CON	EXERCISE1	40	50
35	211	37.03	37.25	37.26	145	5	CON	EXERCISE1	40	50
	212	37.04	37.26	37.26	144	5	CON	EXERCISE1	40	50
	213	37.05	37.29	37.28	150	5	CON	EXERCISE1	40	50
	214	37.05	37.29	37.29	150	5	CON	EXERCISE1	40	50
	215	37.05	37.30	37.30	146	5	CON	EXERCISE1	40	50
	216	37.05	37.31	37.30	143	5	CON	EXERCISE1	40	50
	217	37.06	37.33	37.31	141	5	CON	EXERCISE1	40	50
	218	37.07	37.33	37.31	146	5	CON	EXERCISE1	40	50
	219	37.07	37.32	37.31	150	5	CON	EXERCISE1	40	50
	220	37.08	37.33	37.32	150	5	CON	EXERCISE1	40	50
	221	37.07	37.35	37.33	150	5	CON	EXERCISE1	40	50
	222	37.07	37.35	37.33	150	5	CON	EXERCISE1	40	50
	223	37.08	37.36	37.34	149	5	CON	EXERCISE1	40	50
	224	37.09	37.38	37.35	148	5	CON	EXERCISE1	40	50
	225	37.10	37.41	37.35	148	5	CON	EXERCISE1	40	50
	226	37.11	37.42	37.35	153	5	CON	EXERCISE1	40	50
	227	37.12	37.42	37.36	155	5	CON	EXERCISE1	40	50
	228	37.12	37.39	37.37	155	5	CON	EXERCISE1	40	50
	229	37.12	37.39	37.38	153	5	CON	EXERCISE1	40	50
	230	37.12	37.43	37.38	149	5	CON	EXERCISE1	40	50
	231	37.12	37.44	37.37	150	5	CON	EXERCISE1	40	50
	232	37.12	37.44	37.38	152	5	CON	EXERCISE1	40	50
	233	37.13	37.43	37.40	153	5	CON	EXERCISE1	40	50
	234	37.13	37.44	37.40	153	5	CON	EXERCISE1	40	50
	235	37.14	37.46	37.40	150	5	CON	EXERCISE1	40	50
	236	37.14	37.45	37.40	153	5	CON	EXERCISE1	40	50
	237	37.14	37.45	37.42	152	5	CON	EXERCISE1	40	50
	238	37.15	37.40	37.43	152	5	CON	EXERCISE1	40	50
	239	37.16	37.40	37.43	149	5	CON	EXERCISE1	40	50
	240	37.17	37.47	37.42	150	5	CON	EXERCISE1	40	50
40	241	37.18	37.49	37.43	149	5	CON	EXERCISE1	40	50
	242	37.17	37.52	37.44	143	5	CON	EXERCISE1	40	50
	243	37.17	37.52	37.45	143	5	CON	EXERCISE1	40	50
	244	37.18	37.52	37.46	146	5	CON	EXERCISE1	40	50
	245	37.19	37.52	37.46	146	5	CON	EXERCISE1	40	50
	246	37.20	37.53	37.46	149	5	CON	EXERCISE1	40	50

min	number	Tre	Tes	Tsk-head	HR	ID	condition	period	Ta	RH
	247	37.21	37.55	37.47	150	5	CON	EXERCISE1	40	50
	248	37.22	37.55	37.48	151	5	CON	EXERCISE1	40	50
	249	37.22	37.56	37.49	150	5	CON	EXERCISE1	40	50
	250	37.22	37.57	37.49	147	5	CON	EXERCISE1	40	50
	251	37.24	37.58	37.49	150	5	CON	EXERCISE1	40	50
	252	37.25	37.60	37.49	153	5	CON	EXERCISE1	40	50
	253	37.24	37.53	37.50	157	5	CON	EXERCISE1	40	50
	254	37.24	37.53	37.51	157	5	CON	EXERCISE1	40	50
	255	37.25	37.58	37.52	157	5	CON	EXERCISE1	40	50
	256	37.26	37.58	37.53	153	5	CON	EXERCISE1	40	50
	257	37.27	37.61	37.54	147	5	CON	EXERCISE1	40	50
	258	37.28	37.62	37.53	147	5	CON	EXERCISE1	40	50
	259	37.28	37.61	37.54	147	5	CON	EXERCISE1	40	50
	260	37.28	37.63	37.58	153	5	CON	EXERCISE1	40	50
	261	37.29	37.63	37.58	152	5	CON	EXERCISE1	40	50
	262	37.29	37.64	37.55	150	5	CON	EXERCISE1	40	50
	263	37.30	37.65	37.55	150	5	CON	EXERCISE1	40	50
	264	37.30	37.66	37.55	151	5	CON	EXERCISE1	40	50
	265	37.31	37.68	37.55	153	5	CON	EXERCISE1	40	50
	266	37.31	37.70	37.55	150	5	CON	EXERCISE1	40	50
	267	37.32	37.70	37.57	151	5	CON	EXERCISE1	40	50
	268	37.32	37.65	37.57	154	5	CON	EXERCISE1	40	50
	269	37.33	37.65	37.57	157	5	CON	EXERCISE1	40	50
	270	37.33	37.70	37.57	157	5	CON	EXERCISE1	40	50
45	271	37.33	37.68	37.57	158	5	CON	EXERCISE1	40	50
	272	37.33	37.67	37.59	157	5	CON	EXERCISE1	40	50
	273	37.34	37.70	37.65	158	5	CON	EXERCISE1	40	50
	274	37.35	37.71	37.65	156	5	CON	EXERCISE1	40	50
	275	37.35	37.73	37.61	147	5	CON	EXERCISE1	40	50
	276	37.36	37.75	37.62	146	5	CON	EXERCISE1	40	50
	277	37.36	37.74	37.63	153	5	CON	EXERCISE1	40	50
	278	37.36	37.72	37.62	157	5	CON	EXERCISE1	40	50
	279	37.35	37.73	37.62	158	5	CON	EXERCISE1	40	50
	280	37.36	37.77	37.64	158	5	CON	EXERCISE1	40	50
	281	37.37	37.79	37.65	158	5	CON	EXERCISE1	40	50
	282	37.38	37.78	37.63	161	5	CON	EXERCISE1	40	50
	283	37.38	37.78	37.64	161	5	CON	EXERCISE1	40	50
	284	37.39	37.81	37.65	161	5	CON	EXERCISE1	40	50
	285	37.40	37.82	37.65	161	5	CON	EXERCISE1	40	50
	286	37.40	37.82	37.66	161	5	CON	EXERCISE1	40	50
	287	37.41	37.83	37.67	161	5	CON	EXERCISE1	40	50
	288	37.42	37.85	37.69	157	5	CON	EXERCISE1	40	50
	289	37.43	37.87	37.69	161	5	CON	EXERCISE1	40	50
	290	37.43	37.87	37.70	161	5	CON	EXERCISE1	40	50
	291	37.43	37.86	37.69	161	5	CON	EXERCISE1	40	50
	292	37.44	37.83	37.69	162	5	CON	EXERCISE1	40	50

min	number	Tre	Tes	Tsk-head	HR	ID	condition	period	Ta	RH
	293	37.45	37.82	37.71	162	5	CON	EXERCISE1	40	50
	294	37.45	37.88	37.72	162	5	CON	EXERCISE1	40	50
	295	37.45	37.90	37.73	161	5	CON	EXERCISE1	40	50
	296	37.46	37.91	37.74	161	5	CON	EXERCISE1	40	50
	297	37.47	37.92	37.74	161	5	CON	EXERCISE1	40	50
	298	37.47	37.93	37.75	158	5	CON	EXERCISE1	40	50
	299	37.48	37.88	37.76	161	5	CON	EXERCISE1	40	50
	300	37.50	37.88	37.77	161	5	CON	EXERCISE1	40	50
50	301	37.50	37.90	37.78	162	5	CON	EXERCISE1	40	50
	302	37.50	37.91	37.78	165	5	CON	EXERCISE1	40	50
	303	37.50	37.94	37.79	165	5	CON	EXERCISE1	40	50
	304	37.50	37.94	37.79	164	5	CON	EXERCISE1	40	50
	305	37.51	37.96	37.80	161	5	CON	EXERCISE1	40	50
	306	37.51	37.98	37.80	162	5	CON	EXERCISE1	40	50
	307	37.53	37.99	37.80	166	5	CON	EXERCISE1	40	50
	308	37.53	38.00	37.80	166	5	CON	EXERCISE1	40	50
	309	37.54	38.00	37.80	165	5	CON	EXERCISE1	40	50
	310	37.55	38.02	37.81	165	5	CON	EXERCISE1	40	50
	311	37.55	38.03	37.81	165	5	CON	EXERCISE1	40	50
	312	37.55	38.03	37.81	165	5	CON	EXERCISE1	40	50
	313	37.55	38.04	37.82	166	5	CON	EXERCISE1	40	50
	314	37.56	38.05	37.83	162	5	CON	EXERCISE1	40	50
	315	37.56	38.06	37.83	161	5	CON	EXERCISE1	40	50
	316	37.56	38.07	37.82	157	5	CON	EXERCISE1	40	50
	317	37.57	38.05	37.83	159	5	CON	EXERCISE1	40	50
	318	37.58	38.03	37.84	161	5	CON	EXERCISE1	40	50
	319	37.59	38.07	37.87	161	5	CON	EXERCISE1	40	50
	320	37.60	38.09	37.88	157	5	CON	REST2	28	50
	321	37.60	38.10	37.88	156	5	CON	REST2	28	50
	322	37.58	38.10	37.69	156	5	CON	REST2	28	50
	323	37.58	38.14	37.45	150	5	CON	REST2	28	50
	324	37.59	38.17	37.34	146	5	CON	REST2	28	50
	325	37.60	38.18	37.26	143	5	CON	REST2	28	50
	326	37.63	38.19	37.23	140	5	CON	REST2	28	50
	327	37.63	38.15	37.21	134	5	CON	REST2	28	50
	328	37.63	38.11	37.16	135	5	CON	REST2	28	50
	329	37.64	38.12	37.10	132	5	CON	REST2	28	50
	330	37.65	38.13	37.01	134	5	CON	REST2	28	50
55	331	37.65	38.13	36.90	128	5	CON	REST2	28	50
	332	37.65	38.07	36.83	139	5	CON	REST2	28	50
	333	37.65	38.04	36.80	140	5	CON	REST2	28	50
	334	37.65	38.06	36.76	139	5	CON	REST2	28	50
	335	37.65	38.08	36.75	141	5	CON	REST2	28	50
	336	37.66	38.07	36.76	149	5	CON	REST2	28	50
	337	37.66	38.01	36.79	145	5	CON	REST2	28	50
	338	37.66	37.99	36.81	141	5	CON	REST2	28	50

min	number	Tre	Tes	Tsk-head	HR	ID	condition	period	Ta	RH
	339	37.66	32.75	36.83	142	5	CON	REST2	28	50
	340	37.67	30.07	36.81	143	5	CON	REST2	28	50
	341	37.66	33.06	36.79	135	5	CON	REST2	28	50
	342	37.67	33.97	36.75	128	5	CON	REST2	28	50
	343	37.68	34.41	36.75	123	5	CON	REST2	28	50
	344	37.68	34.85	36.80	127	5	CON	REST2	28	50
	345	37.68	35.50	36.78	132	5	CON	REST2	28	50
	346	37.69	35.82	36.74	134	5	CON	REST2	28	50
	347	37.70	35.56	36.71	129	5	CON	REST2	28	50
	348	37.70	33.59	36.74	137	5	CON	REST2	28	50
	349	37.70	30.15	36.77	137	5	CON	REST2	28	50
	350	37.70	28.45	36.79	140	5	CON	REST2	28	50
	351	37.70	29.09	36.81	139	5	CON	REST2	28	50
	352	37.70	29.41	36.81	140	5	CON	REST2	28	50
	353	37.71	29.38	36.80	140	5	CON	REST2	28	50
	354	37.72	29.26	36.81	143	5	CON	REST2	28	50
	355	37.73	28.91	36.80	140	5	CON	REST2	28	50
	356	37.73	29.84	36.79	139	5	CON	REST2	28	50
	357	37.73	30.48	36.80	138	5	CON	REST2	28	50
	358	37.73	30.02	36.79	137	5	CON	REST2	28	50
	359	37.73	29.59	36.78	134	5	CON	REST2	28	50
	360	37.74	29.34	36.74	134	5	CON	REST2	28	50
60	361	37.74	28.99	36.72	136	5	CON	REST2	28	50
	362	37.73	28.85	36.69	133	5	CON	REST2	28	50
	363	37.73	30.11	36.62	125	5	CON	REST2	28	50
	364	37.74	31.72	36.59	126	5	CON	REST2	28	50
	365	37.74	32.73	36.61	125	5	CON	REST2	28	50
	366	37.75	33.68	36.59	125	5	CON	REST2	28	50
	367	37.75	34.71	36.54	125	5	CON	REST2	28	50
	368	37.76	35.34	36.53	120	5	CON	REST2	28	50
	369	37.75	35.76	36.49	118	5	CON	REST2	28	50
	370	37.75	36.11	36.40	121	5	CON	REST2	28	50
	371	37.75	36.41	36.34	125	5	CON	REST2	28	50
	372	37.75	36.58	36.36	124	5	CON	REST2	28	50
	373	37.74	36.69	36.40	118	5	CON	REST2	28	50
	374	37.74	36.80	36.42	115	5	CON	REST2	28	50
	375	37.75	36.90	36.39	114	5	CON	REST2	28	50
	376	37.74	36.96	36.41	117	5	CON	REST2	28	50
	377	37.74	37.00	36.44	116	5	CON	REST2	28	50
	378	37.74	37.04	36.41	117	5	CON	REST2	28	50
	379	37.74	37.05	36.43	114	5	CON	REST2	28	50
	380	37.75	37.07	36.44	109	5	CON	REST2	28	50
	381	37.74	37.10	36.43	113	5	CON	REST2	28	50
	382	37.74	37.13	36.44	112	5	CON	REST2	28	50
	383	37.75	37.15	36.42	114	5	CON	REST2	28	50
	384	37.75	37.10	36.39	117	5	CON	REST2	28	50

min	number	Tre	Tes	Tsk-head	HR	ID	condition	period	Ta	RH
	385	37.74	37.08	36.35	112	5	CON	REST2	28	50
	386	37.74	37.12	36.33	116	5	CON	REST2	28	50
	387	37.74	37.14	36.34	114	5	CON	REST2	28	50
	388	37.76	37.14	36.34	113	5	CON	REST2	28	50
	389	37.76	37.15	36.37	116	5	CON	REST2	28	50
	390	37.75	37.15	36.41	112	5	CON	REST2	28	50
65	391	37.74	37.16	36.41	109	5	CON	REST2	28	50
	392	37.74	37.16	36.40	110	5	CON	REST2	28	50
	393	37.75	37.15	36.35	110	5	CON	REST2	28	50
	394	37.76	37.14	36.31	110	5	CON	REST2	28	50
	395	37.75	37.14	36.31	110	5	CON	REST2	28	50
	396	37.74	37.15	36.31	112	5	CON	REST2	28	50
	397	37.74	37.17	36.31	112	5	CON	REST2	28	50
	398	37.73	37.17	36.32	110	5	CON	REST2	28	50
	399	37.73	37.15	36.28	110	5	CON	REST2	28	50
	400	37.74	37.16	36.25	112	5	CON	REST2	28	50
	401	37.73	37.16	36.27	110	5	CON	REST2	28	50
	402	37.72	37.12	36.29	115	5	CON	REST2	28	50
	403	37.72	37.09	36.27	108	5	CON	REST2	28	50
	404	37.72	37.10	36.26	106	5	CON	REST2	28	50
	405	37.73	37.13	36.28	104	5	CON	REST2	28	50
	406	37.72	37.13	36.24	108	5	CON	REST2	28	50
	407	37.72	37.13	36.19	112	5	CON	REST2	28	50
	408	37.73	37.13	36.18	111	5	CON	REST2	28	50
	409	37.72	37.14	36.19	112	5	CON	REST2	28	50
	410	37.71	37.09	36.21	115	5	CON	REST2	28	50
	411	37.72	37.09	36.22	108	5	CON	REST2	28	50
	412	37.72	37.14	36.24	106	5	CON	REST2	28	50
	413	37.72	37.15	36.24	108	5	CON	REST2	28	50
	414	37.72	37.15	36.22	106	5	CON	REST2	28	50
	415	37.72	37.16	36.17	107	5	CON	REST2	28	50
	416	37.72	36.95	36.15	110	5	CON	REST2	28	50
	417	37.72	36.91	36.18	109	5	CON	REST2	28	50
	418	37.72	36.93	36.14	114	5	CON	REST2	28	50
	419	37.73	36.92	36.04	107	5	CON	REST2	28	50
	420	37.72	37.07	36.01	108	5	CON	REST2	28	50
70	421	37.72	37.08	36.03	108	5	CON	REST2	28	50
	422	37.72	37.10	36.03	113	5	CON	REST2	28	50
	423	37.72	37.11	36.03	111	5	CON	REST2	28	50
	424	37.72	37.12	36.00	110	5	CON	REST2	28	50
	425	37.71	37.13	36.03	113	5	CON	REST2	28	50
	426	37.71	37.16	36.04	110	5	CON	REST2	28	50
	427	37.71	37.17	36.00	110	5	CON	REST2	28	50
	428	37.70	37.16	36.04	104	5	CON	REST2	28	50
	429	37.70	37.16	36.04	111	5	CON	REST2	28	50
	430	37.70	37.16	36.03	112	5	CON	REST2	28	50

min	number	Tre	Tes	Tsk-head	HR	ID	condition	period	Ta	RH
	431	37.70	37.15	35.97	106	5	CON	REST2	28	50
	432	37.71	37.15	35.98	104	5	CON	REST2	28	50
	433	37.71	37.15	36.07	111	5	CON	REST2	28	50
	434	37.71	37.15	36.05	118	5	CON	REST2	28	50
	435	37.72	37.16	36.04	112	5	CON	REST2	28	50
	436	37.71	37.16	36.06	104	5	CON	REST2	28	50
	437	37.71	37.16	36.08	102	5	CON	REST2	28	50
	438	37.71	37.18	36.08	108	5	CON	REST2	28	50
	439	37.70	37.17	36.07	99	5	CON	REST2	40	50
	440	37.71	37.14	36.22	110	5	CON	REST2	40	50
	441	37.72	37.14	36.47	116	5	CON	REST2	40	50
	442	37.73	37.13	36.62	118	5	CON	REST2	40	50
	443	37.72	37.13	36.73	112	5	CON	REST2	40	50
	444	37.70	37.14	36.79	113	5	CON	REST2	40	50
	445	37.69	37.14	36.84	111	5	CON	REST2	40	50
	446	37.69	37.12	36.88	106	5	CON	REST2	40	50
	447	37.69	37.12	36.92	108	5	CON	REST2	40	50
	448	37.69	37.12	36.94	104	5	CON	REST2	40	50
	449	37.69	37.12	36.96	102	5	CON	REST2	40	50
	450	37.70	37.13	36.97	101	5	CON	REST2	40	50
75	451	37.70	37.13	36.98	105	5	CON	REST2	40	50
	452	37.69	37.14	36.99	108	5	CON	REST2	40	50
	453	37.70	37.13	37.00	110	5	CON	REST2	40	50
	454	37.70	37.13	37.01	103	5	CON	REST2	40	50
	455	37.69	37.14	37.01	102	5	CON	REST2	40	50
	456	37.68	37.13	37.02	104	5	CON	REST2	40	50
	457	37.68	37.13	37.02	106	5	CON	REST2	40	50
	458	37.68	37.13	37.03	105	5	CON	REST2	40	50
	459	37.68	37.12	37.04	123	5	CON	REST2	40	50
	460	37.68	37.08	37.04	127	5	CON	REST2	40	50
	461	37.68	37.06	37.04	118	5	CON	REST2	40	50
	462	37.69	37.09	37.05	120	5	CON	REST2	40	50
	463	37.68	37.07	37.06	126	5	CON	EXERCISE2	40	50
	464	37.68	37.06	37.09	122	5	CON	EXERCISE2	40	50
	465	37.68	37.11	37.11	122	5	CON	EXERCISE2	40	50
	466	37.67	37.14	37.10	126	5	CON	EXERCISE2	40	50
	467	37.67	37.17	37.10	129	5	CON	EXERCISE2	40	50
	468	37.67	37.19	37.11	132	5	CON	EXERCISE2	40	50
	469	37.66	37.20	37.11	134	5	CON	EXERCISE2	40	50
	470	37.65	37.21	37.12	135	5	CON	EXERCISE2	40	50
	471	37.66	37.22	37.13	134	5	CON	EXERCISE2	40	50
	472	37.65	37.24	37.15	134	5	CON	EXERCISE2	40	50
	473	37.66	37.21	37.16	137	5	CON	EXERCISE2	40	50
	474	37.66	37.21	37.19	129	5	CON	EXERCISE2	40	50
	475	37.65	37.27	37.21	130	5	CON	EXERCISE2	40	50
	476	37.64	37.31	37.21	133	5	CON	EXERCISE2	40	50

min	number	Tre	Tes	Tsk-head	HR	ID	condition	period	Ta	RH
	477	37.64	37.32	37.24	134	5	CON	EXERCISE2	40	50
	478	37.65	37.33	37.26	134	5	CON	EXERCISE2	40	50
	479	37.64	37.35	37.28	134	5	CON	EXERCISE2	40	50
	480	37.64	37.36	37.28	140	5	CON	EXERCISE2	40	50
80	481	37.66	37.37	37.30	137	5	CON	EXERCISE2	40	50
	482	37.68	37.38	37.32	143	5	CON	EXERCISE2	40	50
	483	37.67	37.37	37.33	142	5	CON	EXERCISE2	40	50
	484	37.66	37.40	37.34	142	5	CON	EXERCISE2	40	50
	485	37.65	37.41	37.36	143	5	CON	EXERCISE2	40	50
	486	37.65	37.42	37.38	143	5	CON	EXERCISE2	40	50
	487	37.66	37.45	37.39	145	5	CON	EXERCISE2	40	50
	488	37.66	37.45	37.40	146	5	CON	EXERCISE2	40	50
	489	37.65	37.45	37.40	147	5	CON	EXERCISE2	40	50
	490	37.65	37.46	37.40	150	5	CON	EXERCISE2	40	50
	491	37.66	37.39	37.42	147	5	CON	EXERCISE2	40	50
	492	37.67	37.38	37.44	146	5	CON	EXERCISE2	40	50
	493	37.67	37.45	37.45	146	5	CON	EXERCISE2	40	50
	494	37.67	37.46	37.46	146	5	CON	EXERCISE2	40	50
	495	37.68	37.47	37.45	150	5	CON	EXERCISE2	40	50
	496	37.68	37.32	37.45	147	5	CON	EXERCISE2	40	50
	497	37.68	37.32	37.47	154	5	CON	EXERCISE2	40	50
	498	37.68	37.50	37.47	151	5	CON	EXERCISE2	40	50
	499	37.69	37.54	37.46	150	5	CON	EXERCISE2	40	50
	500	37.68	37.54	37.47	154	5	CON	EXERCISE2	40	50
	501	37.68	37.55	37.51	154	5	CON	EXERCISE2	40	50
	502	37.69	37.57	37.52	151	5	CON	EXERCISE2	40	50
	503	37.69	37.59	37.50	154	5	CON	EXERCISE2	40	50
	504	37.69	37.59	37.51	154	5	CON	EXERCISE2	40	50
	505	37.70	37.60	37.52	153	5	CON	EXERCISE2	40	50
	506	37.70	37.62	37.52	154	5	CON	EXERCISE2	40	50
	507	37.70	37.57	37.53	155	5	CON	EXERCISE2	40	50
	508	37.70	37.54	37.55	154	5	CON	EXERCISE2	40	50
	509	37.70	37.58	37.56	154	5	CON	EXERCISE2	40	50
	510	37.70	37.60	37.57	154	5	CON	EXERCISE2	40	50
85	511	37.71	37.62	37.58	154	5	CON	EXERCISE2	40	50
	512	37.71	37.64	37.59	158	5	CON	EXERCISE2	40	50
	513	37.71	37.65	37.60	157	5	CON	EXERCISE2	40	50
	514	37.72	37.66	37.61	158	5	CON	EXERCISE2	40	50
	515	37.72	37.67	37.60	158	5	CON	EXERCISE2	40	50
	516	37.72	37.69	37.59	158	5	CON	EXERCISE2	40	50
	517	37.72	37.71	37.60	154	5	CON	EXERCISE2	40	50
	518	37.73	37.73	37.61	154	5	CON	EXERCISE2	40	50
	519	37.73	37.73	37.60	157	5	CON	EXERCISE2	40	50
	520	37.74	37.73	37.61	155	5	CON	EXERCISE2	40	50
	521	37.74	37.75	37.63	152	5	CON	EXERCISE2	40	50
	522	37.75	37.76	37.63	152	5	CON	EXERCISE2	40	50

min	number	Tre	Tes	Tsk-head	HR	ID	condition	period	Ta	RH
	523	37.75	37.75	37.63	154	5	CON	EXERCISE2	40	50
	524	37.76	37.72	37.64	155	5	CON	EXERCISE2	40	50
	525	37.76	37.72	37.63	154	5	CON	EXERCISE2	40	50
	526	37.77	37.75	37.64	155	5	CON	EXERCISE2	40	50
	527	37.77	37.77	37.65	157	5	CON	EXERCISE2	40	50
	528	37.78	37.80	37.66	157	5	CON	EXERCISE2	40	50
	529	37.78	37.80	37.66	157	5	CON	EXERCISE2	40	50
	530	37.78	37.82	37.67	155	5	CON	EXERCISE2	40	50
	531	37.79	37.86	37.68	155	5	CON	EXERCISE2	40	50
	532	37.79	37.87	37.68	155	5	CON	EXERCISE2	40	50
	533	37.79	37.87	37.68	154	5	CON	EXERCISE2	40	50
	534	37.79	37.89	37.69	154	5	CON	EXERCISE2	40	50
	535	37.80	37.90	37.70	157	5	CON	EXERCISE2	40	50
	536	37.81	37.90	37.70	156	5	CON	EXERCISE2	40	50
	537	37.82	37.91	37.70	159	5	CON	EXERCISE2	40	50
	538	37.82	37.90	37.70	157	5	CON	EXERCISE2	40	50
	539	37.82	37.92	37.71	155	5	CON	EXERCISE2	40	50
	540	37.82	37.93	37.72	157	5	CON	EXERCISE2	40	50
90	541	37.82	37.86	37.72	158	5	CON	EXERCISE2	40	50
	542	37.83	37.84	37.74	159	5	CON	EXERCISE2	40	50
	543	37.85	37.91	37.75	157	5	CON	EXERCISE2	40	50
	544	37.85	37.84	37.79	158	5	CON	EXERCISE2	40	50
	545	37.85	37.79	37.79	161	5	CON	EXERCISE2	40	50
	546	37.85	37.86	37.77	161	5	CON	EXERCISE2	40	50
	547	37.86	37.92	37.78	158	5	CON	EXERCISE2	40	50
	548	37.87	37.96	37.78	157	5	CON	EXERCISE2	40	50
	549	37.87	37.97	37.78	157	5	CON	EXERCISE2	40	50
	550	37.87	37.97	37.79	157	5	CON	EXERCISE2	40	50
	551	37.87	37.98	37.80	158	5	CON	EXERCISE2	40	50
	552	37.88	37.99	37.81	157	5	CON	EXERCISE2	40	50
	553	37.89	38.02	37.83	158	5	CON	EXERCISE2	40	50
	554	37.89	38.01	37.84	162	5	CON	EXERCISE2	40	50
	555	37.88	38.01	37.84	158	5	CON	EXERCISE2	40	50
	556	37.89	38.02	37.84	160	5	CON	EXERCISE2	40	50
	557	37.90	38.04	37.87	165	5	CON	EXERCISE2	40	50
	558	37.91	38.06	37.88	163	5	CON	EXERCISE2	40	50
	559	37.93	38.06	37.87	161	5	CON	EXERCISE2	40	50
	560	37.93	38.08	37.87	162	5	CON	EXERCISE2	40	50
	561	37.93	38.07	37.87	166	5	CON	EXERCISE2	40	50
	562	37.93	38.08	37.88	166	5	CON	EXERCISE2	40	50
	563	37.93	38.10	37.88	166	5	CON	EXERCISE2	40	50
	564	37.93	38.10	37.88	166	5	CON	EXERCISE2	40	50
	565	37.94	38.12	37.89	166	5	CON	EXERCISE2	40	50
	566	37.95	38.13	37.90	166	5	CON	EXERCISE2	40	50
	567	37.95	38.13	37.91	166	5	CON	EXERCISE2	40	50
	568	37.95	38.15	37.91	166	5	CON	EXERCISE2	40	50

min	number	Tre	Tes	Tsk-head	HR	ID	condition	period	Ta	RH
	569	37.96	38.09	37.91	169	5	CON	EXERCISE2	40	50
	570	37.97	38.05	37.93	170	5	CON	EXERCISE2	40	50
95	571	37.98	38.09	37.94	170	5	CON	EXERCISE2	40	50
	572	37.98	38.12	37.95	170	5	CON	EXERCISE2	40	50
	573	37.99	38.14	37.97	170	5	CON	EXERCISE2	40	50
	574	38.00	38.17	37.99	170	5	CON	EXERCISE2	40	50
	575	38.00	38.19	37.99	170	5	CON	EXERCISE2	40	50
	576	38.00	38.19	37.99	171	5	CON	EXERCISE2	40	50
	577	38.01	38.19	38.01	171	5	CON	EXERCISE2	40	50
	578	38.02	38.22	38.02	170	5	CON	EXERCISE2	40	50
	579	38.03	38.23	38.02	170	5	CON	EXERCISE2	40	50
	580	38.03	38.22	38.01	170	5	CON	EXERCISE2	40	50
	581	38.03	38.23	38.02	170	5	CON	EXERCISE2	40	50
	582	38.04	38.25	38.03	170	5	CON	EXERCISE2	40	50
	583	38.05	38.26	38.03	167	5	CON	EXERCISE2	40	50
	584	38.06	38.28	38.05	166	5	CON	EXERCISE2	40	50
	585	38.06	38.29	38.05	166	5	CON	EXERCISE2	40	50
	586	38.07	38.30	38.05	165	5	CON	EXERCISE2	40	50
	587	38.08	38.31	38.05	166	5	CON	EXERCISE2	40	50
	588	38.08	38.21	38.05	170	5	CON	EXERCISE2	40	50
	589	38.09	38.18	38.07	169	5	CON	EXERCISE2	40	50
	590	38.10	38.28	38.09	170	5	CON	EXERCISE2	40	50
	591	38.10	38.31	38.09	170	5	CON	EXERCISE2	40	50
	592	38.10	38.34	38.11	170	5	CON	EXERCISE2	40	50
	593	38.11	38.35	38.13	170	5	CON	EXERCISE2	40	50
	594	38.12	38.35	38.14	170	5	CON	EXERCISE2	40	50
	595	38.13	38.36	38.14	167	5	CON	EXERCISE2	40	50
	596	38.13	38.38	38.14	167	5	CON	EXERCISE2	40	50
	597	38.13	38.38	38.14	167	5	CON	EXERCISE2	40	50
	598	38.13	38.40	38.14	170	5	CON	EXERCISE2	40	50
	599	38.14	38.41	38.14	175	5	CON	EXERCISE2	40	50
	600	38.15	38.41	38.16	175	5	CON	EXERCISE2	40	50
100	601	38.15	38.42	38.19	175	5	CON	EXERCISE2	40	50
	602	38.15	38.43	38.19	176	5	CON	EXERCISE2	40	50
	603	38.16	38.44	38.20	175	5	CON	EXERCISE2	40	50
	604	38.18	38.46	38.21	175	5	CON	EXERCISE2	40	50
	605	38.19	38.43	38.22	175	5	CON	EXERCISE2	40	50
	606	38.20	38.42	38.24	175	5	CON	EXERCISE2	40	50
	607	38.20	38.46	38.25	175	5	CON	EXERCISE2	40	50
	608	38.21	38.47	38.25	175	5	CON	EXERCISE2	40	50
	609	38.21	38.49	38.26	175	5	CON	EXERCISE2	40	50
	610	38.21	38.50	38.27	175	5	CON	EXERCISE2	40	50
	611	38.21	38.51	38.26	175	5	CON	EXERCISE2	40	50
	612	38.23	38.52	38.27	175	5	CON	EXERCISE2	40	50
	613	38.24	38.54	38.27	175	5	CON	EXERCISE2	40	50
	614	38.23	38.55	38.27	175	5	CON	EXERCISE2	40	50

min	number	Tre	Tes	Tsk-head	HR	ID	condition	period	Ta	RH
	615	38.23	38.49	38.28	175	5	CON	EXERCISE2	40	50
	616	38.24	38.47	38.29	180	5	CON	EXERCISE2	40	50
	617	38.25	38.51	38.29	178	5	CON	EXERCISE2	40	50
	618	38.26	38.54	38.32	175	5	CON	EXERCISE2	40	50
	619	38.26	38.58	38.32	170	5	CON	EXERCISE2	40	50
	620	38.26	38.59	38.32	175	5	CON	EXERCISE2	40	50
	621	38.27	38.59	38.34	175	5	CON	EXERCISE2	40	50
	622	38.28	38.61	38.35	175	5	CON	EXERCISE2	40	50
	623	38.29	38.62	38.35	180	5	CON	EXERCISE2	40	50
	624	38.31	38.63	38.37	180	5	CON	EXERCISE2	40	50
	625	38.32	38.65	38.39	180	5	CON	EXERCISE2	40	50
	626	38.32	38.68	38.40	179	5	CON	EXERCISE2	40	50
	627	38.32	38.69	38.41	180	5	CON	EXERCISE2	40	50
	628	38.33	38.63	38.41	180	5	CON	EXERCISE2	40	50
	629	38.33	38.61	38.41	180	5	CON	EXERCISE2	40	50
	630	38.34	38.67	38.44	180	5	CON	EXERCISE2	40	50
105	631	38.35	38.69	38.45	180	5	CON	EXERCISE2	40	50
	632	38.36	38.70	38.46	181	5	CON	EXERCISE2	40	50
	633	38.36	38.72	38.46	180	5	CON	EXERCISE2	40	50
	634	38.35	38.73	38.45	180	5	CON	EXERCISE2	40	50
	635	38.35	38.73	38.44	181	5	CON	EXERCISE2	40	50
	636	38.35	38.74	38.44	182	5	CON	EXERCISE2	40	50
	637	38.36	38.78	38.44	180	5	CON	EXERCISE2	40	50
	638	38.37	38.80	38.44	180	5	CON	EXERCISE2	40	50
	639	38.38	38.81	38.43	180	5	CON	EXERCISE2	40	50
	640	38.39	38.82	38.44	183	5	CON	EXERCISE2	40	50
	641	38.40	38.84	38.47	181	5	CON	EXERCISE2	40	50
	642	38.40	38.85	38.48	180	5	CON	EXERCISE2	40	50
	643	38.40	38.85	38.47	180	5	CON	EXERCISE2	40	50
	644	38.41	38.87	38.49	179	5	CON	REST3	28	50
	645	38.42	38.83	38.52	175	5	CON	REST3	28	50
	646	38.42	38.80	38.53	170	5	CON	REST3	28	50
	647	38.43	38.82	38.54	170	5	CON	REST3	28	50
	648	38.45	38.86	38.56	168	5	CON	REST3	28	50
	649	38.45	38.88	38.56	166	5	CON	REST3	28	50
	650	38.45	38.73	38.52	169	5	CON	REST3	28	50
	651	38.45	38.68	38.35	166	5	CON	REST3	28	50
	652	38.45	38.82	38.10	163	5	CON	REST3	28	50
	653	38.45	38.88	37.96	158	5	CON	REST3	28	50
	654	38.46	38.90	37.91	154	5	CON	REST3	28	50
	655	38.47	38.90	37.85	152	5	CON	REST3	28	50
	656	38.47	38.91	37.76	156	5	CON	REST3	28	50
	657	38.48	38.92	37.69	156	5	CON	REST3	28	50
	658	38.49	38.91	37.66	158	5	CON	REST3	28	50
	659	38.50	38.83	37.67	157	5	CON	REST3	28	50
	660	38.50	38.78	37.66	150	5	CON	REST3	28	50

min	number	Tre	Tes	Tsk-head	HR	ID	condition	period	Ta	RH
110	661	38.50	38.83	37.62	150	5	CON	REST3	28	50
	662	38.51	38.85	37.56	150	5	CON	REST3	28	50
	663	38.52	38.85	37.53	146	5	CON	REST3	28	50
	664	38.53	38.84	37.51	147	5	CON	REST3	28	50
	665	38.54	38.81	37.47	150	5	CON	REST3	28	50
	666	38.54	38.81	37.49	146	5	CON	REST3	28	50
	667	38.54	38.81	37.51	143	5	CON	REST3	28	50
	668	38.54	38.80	37.50	143	5	CON	REST3	28	50
	669	38.55	38.78	37.47	140	5	CON	REST3	28	50
	670	38.55	38.78	37.44	141	5	CON	REST3	28	50
	671	38.55	38.75	37.40	143	5	CON	REST3	28	50
	672	38.57	38.73	37.42	144	5	CON	REST3	28	50
	673	38.58	38.71	37.42	146	5	CON	REST3	28	50
	674	38.59	38.74	37.40	146	5	CON	REST3	28	50
	675	38.60	38.75	37.38	139	5	CON	REST3	28	50
	676	38.60	38.73	37.37	135	5	CON	REST3	28	50
	677	38.60	38.71	37.38	140	5	CON	REST3	28	50
	678	38.60	38.68	37.34	142	5	CON	REST3	28	50
	679	38.59	38.64	37.31	143	5	CON	REST3	28	50
	680	38.59	38.63	37.22	140	5	CON	REST3	28	50
	681	38.59	38.53	37.13	139	5	CON	REST3	28	50
	682	38.59	38.47	37.08	140	5	CON	REST3	28	50
	683	38.60	38.51	37.07	140	5	CON	REST3	28	50
	684	38.60	38.46	37.11	140	5	CON	REST3	28	50
	685	38.60	38.46	37.14	140	5	CON	REST3	28	50
	686	38.61	38.23	37.16	142	5	CON	REST3	28	50
	687	38.61	38.13	37.15	137	5	CON	REST3	28	50
	688	38.61	38.32	37.14	140	5	CON	REST3	28	50
	689	38.61	38.13	37.14	138	5	CON	REST3	28	50
	690	38.62	38.14	37.14	135	5	CON	REST3	28	50
115	691	38.63	38.35	37.14	131	5	CON	REST3	28	50
	692	38.63	38.37	37.15	137	5	CON	REST3	28	50
	693	38.64	38.33	37.16	135	5	CON	REST3	28	50
	694	38.65	38.16	37.15	146	5	CON	REST3	28	50
	695	38.66	38.04	37.10	146	5	CON	REST3	28	50
	696	38.66	38.15	37.04	140	5	CON	REST3	28	50
	697	38.66	38.22	37.02	143	5	CON	REST3	28	50
	698	38.66	38.18	37.02	141	5	CON	REST3	28	50
	699	38.66	38.20	37.04	135	5	CON	REST3	28	50
	700	38.66	38.23	37.05	139	5	CON	REST3	28	50
	701	38.66	38.23	37.04	141	5	CON	REST3	28	50
	702	38.67	38.25	37.03	134	5	CON	REST3	28	50
	703	38.67	38.27	37.04	131	5	CON	REST3	28	50
	704	38.68	38.30	37.05	131	5	CON	REST3	28	50
	705	38.68	38.37	36.99	132	5	CON	REST3	28	50
	706	38.68	38.32	36.92	131	5	CON	REST3	28	50

min	number	Tre	Tes	Tsk-head	HR	ID	condition	period	Ta	RH
	707	38.68	38.25	36.93	128	5	CON	REST3	28	50
	708	38.68	38.26	36.95	128	5	CON	REST3	28	50
	709	38.68	38.28	36.94	128	5	CON	REST3	28	50
0	1	36.77	36.82	35.26	73	6	CON	REST1	28	50
	2	36.77	36.83	35.26	72	6	CON	REST1	28	50
	3	36.77	36.82	35.26	70	6	CON	REST1	28	50
	4	36.77	36.82	35.25	69	6	CON	REST1	28	50
	5	36.76	36.85	35.26	72	6	CON	REST1	28	50
	6	36.77	36.84	35.26	63	6	CON	REST1	28	50
	7	36.77	36.81	35.26	64	6	CON	REST1	28	50
	8	36.76	36.80	35.26	68	6	CON	REST1	28	50
	9	36.76	36.80	35.25	69	6	CON	REST1	28	50
	10	36.76	36.80	35.24	71	6	CON	REST1	28	50
	11	36.76	36.82	35.24	69	6	CON	REST1	28	50
	12	36.76	36.83	35.25	68	6	CON	REST1	28	50
	13	36.75	36.81	35.25	72	6	CON	REST1	28	50
	14	36.75	36.82	35.25	70	6	CON	REST1	28	50
	15	36.75	36.81	35.25	73	6	CON	REST1	28	50
	16	36.74	36.78	35.24	75	6	CON	REST1	28	50
	17	36.74	36.77	35.24	77	6	CON	REST1	28	50
	18	36.74	36.78	35.25	87	6	CON	REST1	28	50
	19	36.74	36.75	35.28	74	6	CON	REST1	28	50
	20	36.75	36.73	35.31	69	6	CON	REST1	28	50
	21	36.76	36.73	35.33	67	6	CON	REST1	28	50
	22	36.75	36.75	35.34	67	6	CON	REST1	28	50
	23	36.75	36.77	35.36	63	6	CON	REST1	28	50
	24	36.74	36.76	35.38	69	6	CON	REST1	28	50
	25	36.73	36.75	35.39	67	6	CON	REST1	28	50
	26	36.73	36.75	35.40	63	6	CON	REST1	28	50
	27	36.72	36.75	35.39	66	6	CON	REST1	28	50
	28	36.72	36.75	35.38	72	6	CON	REST1	28	50
	29	36.72	36.75	35.39	72	6	CON	REST1	28	50
	30	36.72	36.75	35.38	70	6	CON	REST1	28	50
5	31	36.72	36.74	35.37	71	6	CON	REST1	28	50
	32	36.72	36.74	35.35	64	6	CON	REST1	28	50
	33	36.72	36.76	35.34	68	6	CON	REST1	28	50
	34	36.71	36.75	35.35	71	6	CON	REST1	28	50
	35	36.71	36.72	35.34	68	6	CON	REST1	28	50
	36	36.72	36.72	35.33	66	6	CON	REST1	28	50
	37	36.72	36.73	35.33	68	6	CON	REST1	28	50
	38	36.72	36.75	35.32	76	6	CON	REST1	28	50
	39	36.72	36.75	35.32	74	6	CON	REST1	28	50
	40	36.72	36.74	35.33	63	6	CON	REST1	28	50
	41	36.72	36.74	35.34	71	6	CON	REST1	28	50
	42	36.73	36.76	35.36	65	6	CON	REST1	28	50
	43	36.73	36.75	35.37	67	6	CON	REST1	28	50

min	number	Tre	Tes	Tsk-head	HR	ID	condition	period	Ta	RH
	44	36.72	36.74	35.38	69	6	CON	REST1	28	50
	45	36.72	36.76	35.37	66	6	CON	REST1	28	50
	46	36.71	36.76	35.36	66	6	CON	REST1	28	50
	47	36.71	36.74	35.38	67	6	CON	REST1	28	50
	48	36.72	36.77	35.37	69	6	CON	REST1	28	50
	49	36.72	36.78	35.37	70	6	CON	REST1	28	50
	50	36.72	36.79	35.38	69	6	CON	REST1	28	50
	51	36.71	36.79	35.39	71	6	CON	REST1	28	50
	52	36.71	36.78	35.41	75	6	CON	REST1	28	50
	53	36.72	36.79	35.42	74	6	CON	REST1	28	50
	54	36.72	36.80	35.43	68	6	CON	REST1	28	50
	55	36.72	36.81	35.43	71	6	CON	REST1	28	50
	56	36.70	36.78	35.44	68	6	CON	REST1	28	50
	57	36.70	36.77	35.46	68	6	CON	REST1	28	50
	58	36.71	36.78	35.49	70	6	CON	REST1	28	50
	59	36.71	36.80	35.52	73	6	CON	REST1	28	50
	60	36.71	36.81	35.54	71	6	CON	REST1	28	50
10	61	36.71	36.82	35.54	71	6	CON	REST1	28	50
	62	36.70	36.84	35.53	71	6	CON	REST1	28	50
	63	36.70	36.83	35.53	66	6	CON	REST1	28	50
	64	36.70	36.80	35.54	71	6	CON	REST1	28	50
	65	36.70	36.82	35.54	68	6	CON	REST1	28	50
	66	36.70	36.85	35.54	75	6	CON	REST1	28	50
	67	36.71	36.82	35.55	79	6	CON	REST1	28	50
	68	36.71	36.79	35.53	73	6	CON	REST1	28	50
	69	36.71	36.81	35.53	75	6	CON	REST1	28	50
	70	36.72	36.80	35.54	72	6	CON	REST1	28	50
	71	36.72	36.81	35.53	78	6	CON	REST1	28	50
	72	36.72	36.80	35.56	74	6	CON	REST1	28	50
	73	36.72	36.79	35.59	66	6	CON	REST1	28	50
	74	36.72	36.81	35.59	68	6	CON	REST1	28	50
	75	36.72	36.80	35.59	72	6	CON	REST1	28	50
	76	36.72	36.78	35.59	74	6	CON	REST1	28	50
	77	36.73	36.78	35.61	74	6	CON	REST1	28	50
	78	36.73	36.79	35.60	68	6	CON	REST1	28	50
	79	36.73	36.79	35.57	69	6	CON	REST1	28	50
	80	36.72	36.78	35.55	69	6	CON	REST1	28	50
	81	36.72	36.79	35.52	73	6	CON	REST1	28	50
	82	36.73	36.77	35.50	68	6	CON	REST1	28	50
	83	36.74	36.75	35.48	67	6	CON	REST1	28	50
	84	36.74	36.75	35.46	69	6	CON	REST1	28	50
	85	36.74	36.75	35.45	74	6	CON	REST1	28	50
	86	36.74	36.77	35.44	74	6	CON	REST1	28	50
	87	36.73	36.77	35.44	63	6	CON	REST1	28	50
	88	36.73	36.78	35.44	73	6	CON	REST1	28	50
	89	36.72	36.80	35.42	81	6	CON	REST1	28	50

min	number	Tre	Tes	Tsk-head	HR	ID	condition	period	Ta	RH
	90	36.72	36.80	35.39	78	6	CON	REST1	28	50
15	91	36.71	36.81	35.37	84	6	CON	REST1	28	50
	92	36.69	36.81	35.38	72	6	CON	REST1	28	50
	93	36.68	36.80	35.41	70	6	CON	REST1	28	50
	94	36.67	36.81	35.42	65	6	CON	REST1	28	50
	95	36.66	36.83	35.43	69	6	CON	REST1	28	50
	96	36.67	36.83	35.46	69	6	CON	REST1	28	50
	97	36.68	36.83	35.48	77	6	CON	REST1	28	50
	98	36.68	36.84	35.49	81	6	CON	REST1	28	50
	99	36.66	36.82	35.50	86	6	CON	REST1	28	50
	100	36.64	36.82	35.52	73	6	CON	REST1	28	50
	101	36.62	36.84	35.53	80	6	CON	REST1	28	50
	102	36.62	36.84	35.52	63	6	CON	REST1	28	50
	103	36.62	36.83	35.53	63	6	CON	REST1	40	50
	104	36.62	36.82	35.50	79	6	CON	REST1	40	50
	105	36.62	36.82	35.57	88	6	CON	REST1	40	50
	106	36.61	36.78	35.71	70	6	CON	REST1	40	50
	107	36.61	36.75	35.79	72	6	CON	REST1	40	50
	108	36.61	36.77	35.88	72	6	CON	REST1	40	50
	109	36.60	36.79	35.95	65	6	CON	REST1	40	50
	110	36.59	36.79	36.00	65	6	CON	REST1	40	50
	111	36.60	36.80	36.06	71	6	CON	REST1	40	50
	112	36.60	36.78	36.10	74	6	CON	REST1	40	50
	113	36.60	36.77	36.14	68	6	CON	REST1	40	50
	114	36.60	36.80	36.18	69	6	CON	REST1	40	50
	115	36.59	36.80	36.20	70	6	CON	REST1	40	50
	116	36.59	36.80	36.23	73	6	CON	REST1	40	50
	117	36.59	36.80	36.27	74	6	CON	REST1	40	50
	118	36.59	36.80	36.30	69	6	CON	REST1	40	50
	119	36.59	36.80	36.33	71	6	CON	REST1	40	50
	120	36.58	36.81	36.34	73	6	CON	REST1	40	50
20	121	36.57	36.81	36.36	78	6	CON	REST1	40	50
	122	36.57	36.77	36.39	76	6	CON	REST1	40	50
	123	36.57	36.74	36.42	71	6	CON	REST1	40	50
	124	36.57	36.74	36.43	69	6	CON	REST1	40	50
	125	36.57	36.74	36.45	70	6	CON	REST1	40	50
	126	36.59	36.76	36.47	74	6	CON	REST1	40	50
	127	36.59	36.77	36.50	75	6	CON	REST1	40	50
	128	36.59	36.78	36.52	74	6	CON	REST1	40	50
	129	36.60	36.80	36.52	78	6	CON	REST1	40	50
	130	36.59	36.79	36.52	71	6	CON	REST1	40	50
	131	36.59	36.74	36.52	75	6	CON	REST1	40	50
	132	36.58	36.71	36.54	93	6	CON	REST1	40	50
	133	36.56	36.72	36.55	83	6	CON	REST1	40	50
	134	36.55	36.74	36.55	91	6	CON	REST1	40	50
	135	36.56	36.76	36.55	92	6	CON	REST1	40	50

min	number	Tre	Tes	Tsk-head	HR	ID	condition	period	Ta	RH
	136	36.56	36.77	36.55	82	6	CON	REST1	40	50
	137	36.56	36.76	36.57	79	6	CON	REST1	40	50
	138	36.56	36.72	36.58	71	6	CON	REST1	40	50
	139	36.55	36.73	36.57	79	6	CON	EXERCISE1	40	50
	140	36.55	36.74	36.57	82	6	CON	EXERCISE1	40	50
	141	36.54	36.73	36.58	78	6	CON	EXERCISE1	40	50
	142	36.54	36.72	36.59	89	6	CON	EXERCISE1	40	50
	143	36.53	36.75	36.62	97	6	CON	EXERCISE1	40	50
	144	36.53	36.76	36.62	99	6	CON	EXERCISE1	40	50
	145	36.52	36.73	36.60	98	6	CON	EXERCISE1	40	50
	146	36.51	36.72	36.61	103	6	CON	EXERCISE1	40	50
	147	36.51	36.74	36.61	104	6	CON	EXERCISE1	40	50
	148	36.50	36.75	36.61	101	6	CON	EXERCISE1	40	50
	149	36.50	36.72	36.62	103	6	CON	EXERCISE1	40	50
	150	36.49	36.72	36.61	107	6	CON	EXERCISE1	40	50
25	151	36.49	36.73	36.62	108	6	CON	EXERCISE1	40	50
	152	36.48	36.71	36.62	103	6	CON	EXERCISE1	40	50
	153	36.48	36.72	36.61	98	6	CON	EXERCISE1	40	50
	154	36.48	36.70	36.61	100	6	CON	EXERCISE1	40	50
	155	36.48	36.50	36.61	105	6	CON	EXERCISE1	40	50
	156	36.48	36.45	36.64	105	6	CON	EXERCISE1	40	50
	157	36.47	36.58	36.66	102	6	CON	EXERCISE1	40	50
	158	36.47	36.60	36.66	100	6	CON	EXERCISE1	40	50
	159	36.47	36.62	36.67	99	6	CON	EXERCISE1	40	50
	160	36.47	36.66	36.68	104	6	CON	EXERCISE1	40	50
	161	36.48	36.66	36.67	103	6	CON	EXERCISE1	40	50
	162	36.47	36.64	36.66	105	6	CON	EXERCISE1	40	50
	163	36.47	36.66	36.67	105	6	CON	EXERCISE1	40	50
	164	36.47	36.67	36.67	103	6	CON	EXERCISE1	40	50
	165	36.47	36.67	36.65	107	6	CON	EXERCISE1	40	50
	166	36.47	36.54	36.65	109	6	CON	EXERCISE1	40	50
	167	36.47	36.45	36.65	104	6	CON	EXERCISE1	40	50
	168	36.47	36.53	36.65	103	6	CON	EXERCISE1	40	50
	169	36.48	36.61	36.64	104	6	CON	EXERCISE1	40	50
	170	36.48	36.68	36.62	101	6	CON	EXERCISE1	40	50
	171	36.48	36.70	36.62	104	6	CON	EXERCISE1	40	50
	172	36.49	36.72	36.61	107	6	CON	EXERCISE1	40	50
	173	36.49	36.69	36.60	114	6	CON	EXERCISE1	40	50
	174	36.48	36.68	36.60	113	6	CON	EXERCISE1	40	50
	175	36.49	36.71	36.60	115	6	CON	EXERCISE1	40	50
	176	36.50	36.63	36.62	116	6	CON	EXERCISE1	40	50
	177	36.50	36.58	36.63	110	6	CON	EXERCISE1	40	50
	178	36.51	36.67	36.64	113	6	CON	EXERCISE1	40	50
	179	36.51	36.69	36.64	111	6	CON	EXERCISE1	40	50
	180	36.51	36.70	36.65	109	6	CON	EXERCISE1	40	50
30	181	36.51	36.72	36.66	111	6	CON	EXERCISE1	40	50

min	number	Tre	Tes	Tsk-head	HR	ID	condition	period	Ta	RH
	182	36.52	36.73	36.65	113	6	CON	EXERCISE1	40	50
	183	36.52	36.75	36.64	115	6	CON	EXERCISE1	40	50
	184	36.53	36.76	36.60	111	6	CON	EXERCISE1	40	50
	185	36.54	36.76	36.57	114	6	CON	EXERCISE1	40	50
	186	36.53	36.76	36.56	114	6	CON	EXERCISE1	40	50
	187	36.54	36.78	36.57	112	6	CON	EXERCISE1	40	50
	188	36.55	36.72	36.57	115	6	CON	EXERCISE1	40	50
	189	36.55	36.69	36.56	111	6	CON	EXERCISE1	40	50
	190	36.56	36.75	36.56	113	6	CON	EXERCISE1	40	50
	191	36.56	36.78	36.57	116	6	CON	EXERCISE1	40	50
	192	36.57	36.80	36.58	121	6	CON	EXERCISE1	40	50
	193	36.58	36.79	36.59	116	6	CON	EXERCISE1	40	50
	194	36.58	36.81	36.61	110	6	CON	EXERCISE1	40	50
	195	36.59	36.82	36.60	113	6	CON	EXERCISE1	40	50
	196	36.59	36.81	36.57	112	6	CON	EXERCISE1	40	50
	197	36.59	36.83	36.57	114	6	CON	EXERCISE1	40	50
	198	36.59	36.85	36.57	115	6	CON	EXERCISE1	40	50
	199	36.60	36.86	36.57	117	6	CON	EXERCISE1	40	50
	200	36.61	36.78	36.58	120	6	CON	EXERCISE1	40	50
	201	36.62	36.76	36.59	119	6	CON	EXERCISE1	40	50
	202	36.63	36.83	36.60	120	6	CON	EXERCISE1	40	50
	203	36.63	36.85	36.60	121	6	CON	EXERCISE1	40	50
	204	36.63	36.86	36.60	124	6	CON	EXERCISE1	40	50
	205	36.64	36.87	36.61	125	6	CON	EXERCISE1	40	50
	206	36.65	36.86	36.63	124	6	CON	EXERCISE1	40	50
	207	36.65	36.86	36.62	125	6	CON	EXERCISE1	40	50
	208	36.65	36.85	36.60	123	6	CON	EXERCISE1	40	50
	209	36.67	36.84	36.61	118	6	CON	EXERCISE1	40	50
	210	36.68	36.86	36.62	120	6	CON	EXERCISE1	40	50
35	211	36.68	36.86	36.60	120	6	CON	EXERCISE1	40	50
	212	36.68	36.86	36.60	121	6	CON	EXERCISE1	40	50
	213	36.69	36.88	36.60	123	6	CON	EXERCISE1	40	50
	214	36.70	36.93	36.61	126	6	CON	EXERCISE1	40	50
	215	36.71	36.95	36.62	125	6	CON	EXERCISE1	40	50
	216	36.71	36.92	36.62	124	6	CON	EXERCISE1	40	50
	217	36.71	36.90	36.62	120	6	CON	EXERCISE1	40	50
	218	36.71	36.91	36.62	121	6	CON	EXERCISE1	40	50
	219	36.71	36.94	36.63	126	6	CON	EXERCISE1	40	50
	220	36.72	36.93	36.64	128	6	CON	EXERCISE1	40	50
	221	36.73	36.94	36.66	126	6	CON	EXERCISE1	40	50
	222	36.74	36.98	36.67	128	6	CON	EXERCISE1	40	50
	223	36.75	36.99	36.67	128	6	CON	EXERCISE1	40	50
	224	36.75	36.99	36.66	129	6	CON	EXERCISE1	40	50
	225	36.75	37.00	36.65	129	6	CON	EXERCISE1	40	50
	226	36.75	36.99	36.66	129	6	CON	EXERCISE1	40	50
	227	36.76	37.00	36.68	127	6	CON	EXERCISE1	40	50

min	number	Tre	Tes	Tsk-head	HR	ID	condition	period	Ta	RH
	228	36.77	37.00	36.68	128	6	CON	EXERCISE1	40	50
	229	36.77	36.99	36.70	132	6	CON	EXERCISE1	40	50
	230	36.78	37.02	36.71	133	6	CON	EXERCISE1	40	50
	231	36.79	37.03	36.72	129	6	CON	EXERCISE1	40	50
	232	36.79	37.02	36.73	126	6	CON	EXERCISE1	40	50
	233	36.79	37.03	36.73	126	6	CON	EXERCISE1	40	50
	234	36.80	37.01	36.72	126	6	CON	EXERCISE1	40	50
	235	36.81	36.93	36.71	128	6	CON	EXERCISE1	40	50
	236	36.82	36.95	36.72	126	6	CON	EXERCISE1	40	50
	237	36.83	37.02	36.73	127	6	CON	EXERCISE1	40	50
	238	36.84	37.05	36.75	126	6	CON	EXERCISE1	40	50
	239	36.85	37.08	36.75	128	6	CON	EXERCISE1	40	50
	240	36.84	37.09	36.75	129	6	CON	EXERCISE1	40	50
40	241	36.84	37.04	36.76	132	6	CON	EXERCISE1	40	50
	242	36.85	37.00	36.77	131	6	CON	EXERCISE1	40	50
	243	36.85	37.04	36.78	131	6	CON	EXERCISE1	40	50
	244	36.86	37.07	36.79	132	6	CON	EXERCISE1	40	50
	245	36.88	37.08	36.78	133	6	CON	EXERCISE1	40	50
	246	36.88	37.10	36.79	132	6	CON	EXERCISE1	40	50
	247	36.88	37.12	36.80	134	6	CON	EXERCISE1	40	50
	248	36.88	37.08	36.79	134	6	CON	EXERCISE1	40	50
	249	36.89	37.06	36.81	130	6	CON	EXERCISE1	40	50
	250	36.90	37.08	36.81	128	6	CON	EXERCISE1	40	50
	251	36.91	37.11	36.81	129	6	CON	EXERCISE1	40	50
	252	36.92	37.13	36.81	131	6	CON	EXERCISE1	40	50
	253	36.93	37.16	36.81	132	6	CON	EXERCISE1	40	50
	254	36.94	37.17	36.83	133	6	CON	EXERCISE1	40	50
	255	36.94	37.16	36.83	134	6	CON	EXERCISE1	40	50
	256	36.95	37.19	36.84	137	6	CON	EXERCISE1	40	50
	257	36.96	37.21	36.82	137	6	CON	EXERCISE1	40	50
	258	36.96	37.24	36.80	134	6	CON	EXERCISE1	40	50
	259	36.96	37.26	36.79	137	6	CON	EXERCISE1	40	50
	260	36.97	37.25	36.79	135	6	CON	EXERCISE1	40	50
	261	36.98	37.25	36.81	134	6	CON	EXERCISE1	40	50
	262	36.99	37.26	36.84	132	6	CON	EXERCISE1	40	50
	263	37.00	37.26	36.86	134	6	CON	EXERCISE1	40	50
	264	37.00	37.26	36.87	139	6	CON	EXERCISE1	40	50
	265	37.00	37.25	36.89	137	6	CON	EXERCISE1	40	50
	266	37.01	37.25	36.87	140	6	CON	EXERCISE1	40	50
	267	37.01	37.25	36.85	139	6	CON	EXERCISE1	40	50
	268	37.01	37.24	36.84	135	6	CON	EXERCISE1	40	50
	269	37.03	37.27	36.86	137	6	CON	EXERCISE1	40	50
	270	37.04	37.27	36.88	137	6	CON	EXERCISE1	40	50
45	271	37.03	37.21	36.89	138	6	CON	EXERCISE1	40	50
	272	37.04	37.22	36.92	139	6	CON	EXERCISE1	40	50
	273	37.04	37.29	36.94	143	6	CON	EXERCISE1	40	50

min	number	Tre	Tes	Tsk-head	HR	ID	condition	period	Ta	RH
	274	37.06	37.30	36.96	143	6	CON	EXERCISE1	40	50
	275	37.07	37.33	36.97	140	6	CON	EXERCISE1	40	50
	276	37.07	37.35	36.98	137	6	CON	EXERCISE1	40	50
	277	37.09	37.37	37.00	139	6	CON	EXERCISE1	40	50
	278	37.09	37.38	37.01	140	6	CON	EXERCISE1	40	50
	279	37.09	37.38	37.01	140	6	CON	EXERCISE1	40	50
	280	37.10	37.39	37.02	138	6	CON	EXERCISE1	40	50
	281	37.11	37.39	37.03	139	6	CON	EXERCISE1	40	50
	282	37.12	37.42	37.04	140	6	CON	EXERCISE1	40	50
	283	37.13	37.43	37.05	137	6	CON	EXERCISE1	40	50
	284	37.13	37.43	37.06	137	6	CON	EXERCISE1	40	50
	285	37.14	37.45	37.08	140	6	CON	EXERCISE1	40	50
	286	37.16	37.47	37.09	140	6	CON	EXERCISE1	40	50
	287	37.16	37.46	37.08	140	6	CON	EXERCISE1	40	50
	288	37.16	37.47	37.09	141	6	CON	EXERCISE1	40	50
	289	37.17	37.48	37.09	141	6	CON	EXERCISE1	40	50
	290	37.18	37.47	37.07	140	6	CON	EXERCISE1	40	50
	291	37.19	37.50	37.06	137	6	CON	EXERCISE1	40	50
	292	37.19	37.51	37.08	138	6	CON	EXERCISE1	40	50
	293	37.19	37.52	37.09	141	6	CON	EXERCISE1	40	50
	294	37.20	37.48	37.11	143	6	CON	EXERCISE1	40	50
	295	37.22	37.45	37.12	143	6	CON	EXERCISE1	40	50
	296	37.22	37.47	37.13	145	6	CON	EXERCISE1	40	50
	297	37.23	37.50	37.15	143	6	CON	EXERCISE1	40	50
	298	37.24	37.53	37.15	142	6	CON	EXERCISE1	40	50
	299	37.25	37.52	37.16	140	6	CON	EXERCISE1	40	50
	300	37.25	37.54	37.17	143	6	CON	EXERCISE1	40	50
50	301	37.25	37.57	37.18	143	6	CON	EXERCISE1	40	50
	302	37.26	37.56	37.18	144	6	CON	EXERCISE1	40	50
	303	37.26	37.58	37.18	149	6	CON	EXERCISE1	40	50
	304	37.26	37.59	37.17	146	6	CON	EXERCISE1	40	50
	305	37.27	37.59	37.17	144	6	CON	EXERCISE1	40	50
	306	37.28	37.60	37.18	143	6	CON	EXERCISE1	40	50
	307	37.29	37.61	37.19	143	6	CON	EXERCISE1	40	50
	308	37.30	37.65	37.20	144	6	CON	EXERCISE1	40	50
	309	37.31	37.63	37.21	146	6	CON	EXERCISE1	40	50
	310	37.31	37.59	37.21	146	6	CON	EXERCISE1	40	50
	311	37.32	37.62	37.21	144	6	CON	EXERCISE1	40	50
	312	37.32	37.65	37.20	146	6	CON	EXERCISE1	40	50
	313	37.33	37.68	37.19	147	6	CON	EXERCISE1	40	50
	314	37.34	37.69	37.21	147	6	CON	EXERCISE1	40	50
	315	37.35	37.70	37.25	150	6	CON	EXERCISE1	40	50
	316	37.35	37.73	37.27	150	6	CON	EXERCISE1	40	50
	317	37.36	37.72	37.27	150	6	CON	EXERCISE1	40	50
	318	37.37	37.67	37.27	150	6	CON	EXERCISE1	40	50
	319	37.38	37.67	37.28	146	6	CON	EXERCISE1	40	50

min	number	Tre	Tes	Tsk-head	HR	ID	condition	period	Ta	RH
	320	37.39	37.71	37.30	141	6	CON	REST2	28	50
	321	37.40	37.73	37.27	145	6	CON	REST2	28	50
	322	37.40	37.78	37.11	144	6	CON	REST2	28	50
	323	37.40	37.80	36.99	137	6	CON	REST2	28	50
	324	37.40	37.81	37.01	128	6	CON	REST2	28	50
	325	37.41	37.79	37.02	118	6	CON	REST2	28	50
	326	37.41	37.77	37.00	117	6	CON	REST2	28	50
	327	37.41	37.79	36.99	117	6	CON	REST2	28	50
	328	37.42	37.77	36.99	121	6	CON	REST2	28	50
	329	37.43	37.77	36.96	119	6	CON	REST2	28	50
	330	37.44	37.84	36.90	117	6	CON	REST2	28	50
55	331	37.44	37.87	36.85	118	6	CON	REST2	28	50
	332	37.44	37.86	36.78	121	6	CON	REST2	28	50
	333	37.44	37.85	36.63	126	6	CON	REST2	28	50
	334	37.45	37.85	36.52	121	6	CON	REST2	28	50
	335	37.45	37.86	36.53	120	6	CON	REST2	28	50
	336	37.45	37.87	36.54	116	6	CON	REST2	28	50
	337	37.45	37.86	36.55	114	6	CON	REST2	28	50
	338	37.45	33.00	36.55	109	6	CON	REST2	28	50
	339	37.45	30.41	36.53	112	6	CON	REST2	28	50
	340	37.45	30.35	36.51	114	6	CON	REST2	28	50
	341	37.45	30.56	36.45	99	6	CON	REST2	28	50
	342	37.46	33.20	36.35	114	6	CON	REST2	28	50
	343	37.46	33.50	36.27	107	6	CON	REST2	28	50
	344	37.45	33.75	36.27	107	6	CON	REST2	28	50
	345	37.45	31.31	36.31	119	6	CON	REST2	28	50
	346	37.45	29.38	36.27	101	6	CON	REST2	28	50
	347	37.45	30.52	36.16	104	6	CON	REST2	28	50
	348	37.45	31.65	36.10	100	6	CON	REST2	28	50
	349	37.45	30.12	36.13	104	6	CON	REST2	28	50
	350	37.45	28.44	36.16	100	6	CON	REST2	28	50
	351	37.44	29.75	36.18	108	6	CON	REST2	28	50
	352	37.44	31.11	36.13	101	6	CON	REST2	28	50
	353	37.44	31.91	36.03	97	6	CON	REST2	28	50
	354	37.43	32.54	35.97	98	6	CON	REST2	28	50
	355	37.42	33.05	35.91	101	6	CON	REST2	28	50
	356	37.43	33.52	35.87	101	6	CON	REST2	28	50
	357	37.42	33.88	35.84	101	6	CON	REST2	28	50
	358	37.42	34.15	35.85	99	6	CON	REST2	28	50
	359	37.42	34.42	35.86	97	6	CON	REST2	28	50
	360	37.43	34.67	35.82	98	6	CON	REST2	28	50
60	361	37.42	34.88	35.81	97	6	CON	REST2	28	50
	362	37.42	34.73	35.80	96	6	CON	REST2	28	50
	363	37.41	34.85	35.81	103	6	CON	REST2	28	50
	364	37.40	31.96	35.87	106	6	CON	REST2	28	50
	365	37.40	28.63	35.94	94	6	CON	REST2	28	50

min	number	Tre	Tes	Tsk-head	HR	ID	condition	period	Ta	RH
	366	37.40	29.82	35.94	99	6	CON	REST2	28	50
	367	37.40	31.13	35.92	98	6	CON	REST2	28	50
	368	37.40	30.62	35.91	91	6	CON	REST2	28	50
	369	37.39	30.94	35.84	91	6	CON	REST2	28	50
	370	37.38	30.93	35.78	85	6	CON	REST2	28	50
	371	37.38	30.55	35.76	95	6	CON	REST2	28	50
	372	37.38	31.55	35.74	91	6	CON	REST2	28	50
	373	37.37	32.15	35.72	96	6	CON	REST2	28	50
	374	37.37	32.57	35.71	99	6	CON	REST2	28	50
	375	37.36	33.12	35.68	94	6	CON	REST2	28	50
	376	37.36	33.50	35.56	91	6	CON	REST2	28	50
	377	37.37	33.78	35.50	92	6	CON	REST2	28	50
	378	37.37	34.10	35.54	85	6	CON	REST2	28	50
	379	37.36	34.34	35.52	89	6	CON	REST2	28	50
	380	37.35	34.49	35.48	92	6	CON	REST2	28	50
	381	37.35	34.59	35.45	89	6	CON	REST2	28	50
	382	37.35	34.72	35.41	84	6	CON	REST2	28	50
	383	37.35	34.91	35.38	88	6	CON	REST2	28	50
	384	37.35	35.18	35.40	86	6	CON	REST2	28	50
	385	37.34	35.41	35.38	80	6	CON	REST2	28	50
	386	37.33	35.67	35.36	89	6	CON	REST2	28	50
	387	37.33	34.90	35.35	89	6	CON	REST2	28	50
	388	37.32	33.74	35.34	81	6	CON	REST2	28	50
	389	37.32	34.69	35.35	85	6	CON	REST2	28	50
	390	37.33	35.87	35.33	89	6	CON	REST2	28	50
65	391	37.34	36.00	35.34	89	6	CON	REST2	28	50
	392	37.33	36.11	35.37	88	6	CON	REST2	28	50
	393	37.33	36.18	35.33	90	6	CON	REST2	28	50
	394	37.32	36.24	35.32	85	6	CON	REST2	28	50
	395	37.32	36.30	35.31	83	6	CON	REST2	28	50
	396	37.33	36.41	35.31	84	6	CON	REST2	28	50
	397	37.33	36.49	35.31	86	6	CON	REST2	28	50
	398	37.32	36.54	35.26	81	6	CON	REST2	28	50
	399	37.32	36.60	35.25	84	6	CON	REST2	28	50
	400	37.32	36.58	35.24	88	6	CON	REST2	28	50
	401	37.32	36.63	35.22	86	6	CON	REST2	28	50
	402	37.33	36.75	35.21	81	6	CON	REST2	28	50
	403	37.32	36.78	35.21	83	6	CON	REST2	28	50
	404	37.31	36.76	35.23	85	6	CON	REST2	28	50
	405	37.32	36.77	35.22	88	6	CON	REST2	28	50
	406	37.32	36.82	35.17	82	6	CON	REST2	28	50
	407	37.31	36.84	35.16	84	6	CON	REST2	28	50
	408	37.30	36.85	35.14	86	6	CON	REST2	28	50
	409	37.30	36.90	35.15	88	6	CON	REST2	28	50
	410	37.31	36.91	35.17	83	6	CON	REST2	28	50
	411	37.32	36.94	35.19	84	6	CON	REST2	28	50

min	number	Tre	Tes	Tsk-head	HR	ID	condition	period	Ta	RH
	412	37.32	36.95	35.24	84	6	CON	REST2	28	50
	413	37.32	36.95	35.21	83	6	CON	REST2	28	50
	414	37.32	36.97	35.20	83	6	CON	REST2	28	50
	415	37.31	36.96	35.23	84	6	CON	REST2	28	50
	416	37.31	36.98	35.21	84	6	CON	REST2	28	50
	417	37.31	37.00	35.20	79	6	CON	REST2	28	50
	418	37.31	36.97	35.18	89	6	CON	REST2	28	50
	419	37.31	36.99	35.12	83	6	CON	REST2	28	50
	420	37.32	37.02	35.08	82	6	CON	REST2	28	50
70	421	37.32	37.03	35.09	84	6	CON	REST2	28	50
	422	37.32	37.07	35.09	89	6	CON	REST2	28	50
	423	37.32	37.03	35.10	92	6	CON	REST2	28	50
	424	37.32	37.04	35.13	95	6	CON	REST2	28	50
	425	37.33	37.09	35.17	90	6	CON	REST2	28	50
	426	37.33	37.08	35.22	95	6	CON	REST2	28	50
	427	37.33	36.74	35.24	97	6	CON	REST2	28	50
	428	37.33	36.73	35.24	102	6	CON	REST2	28	50
	429	37.33	37.10	35.09	94	6	CON	REST2	28	50
	430	37.33	37.15	34.92	87	6	CON	REST2	28	50
	431	37.34	37.17	34.93	87	6	CON	REST2	28	50
	432	37.33	37.15	34.94	87	6	CON	REST2	28	50
	433	37.33	37.13	34.94	89	6	CON	REST2	28	50
	434	37.33	37.13	34.97	93	6	CON	REST2	28	50
	435	37.33	37.12	34.99	90	6	CON	REST2	28	50
	436	37.33	37.15	34.96	85	6	CON	REST2	28	50
	437	37.34	37.12	34.93	110	6	CON	REST2	28	50
	438	37.35	37.08	34.95	90	6	CON	REST2	28	50
	439	37.35	37.10	34.97	90	6	CON	REST2	40	50
	440	37.35	37.12	35.02	96	6	CON	REST2	40	50
	441	37.35	37.12	35.12	109	6	CON	REST2	40	50
	442	37.35	37.14	35.19	93	6	CON	REST2	40	50
	443	37.35	37.16	35.27	95	6	CON	REST2	40	50
	444	37.36	37.16	35.36	88	6	CON	REST2	40	50
	445	37.36	37.15	35.45	90	6	CON	REST2	40	50
	446	37.37	37.15	35.56	87	6	CON	REST2	40	50
	447	37.37	37.15	35.63	85	6	CON	REST2	40	50
	448	37.37	37.16	35.71	89	6	CON	REST2	40	50
	449	37.38	37.18	35.78	84	6	CON	REST2	40	50
	450	37.39	37.20	35.81	86	6	CON	REST2	40	50
75	451	37.38	37.20	35.87	84	6	CON	REST2	40	50
	452	37.39	37.21	35.92	88	6	CON	REST2	40	50
	453	37.40	37.21	35.97	88	6	CON	REST2	40	50
	454	37.39	37.21	36.00	90	6	CON	REST2	40	50
	455	37.38	37.20	36.05	91	6	CON	REST2	40	50
	456	37.38	37.15	36.11	96	6	CON	REST2	40	50
	457	37.39	37.16	36.12	88	6	CON	REST2	40	50

min	number	Tre	Tes	Tsk-head	HR	ID	condition	period	Ta	RH
	458	37.38	37.18	36.13	99	6	CON	REST2	40	50
	459	37.37	37.20	36.18	104	6	CON	REST2	40	50
	460	37.38	37.22	36.21	93	6	CON	REST2	40	50
	461	37.39	37.21	36.23	98	6	CON	REST2	40	50
	462	37.39	37.20	36.26	99	6	CON	REST2	40	50
	463	37.38	37.22	36.28	93	6	CON	EXERCISE2	40	50
	464	37.37	37.22	36.30	90	6	CON	EXERCISE2	40	50
	465	37.38	37.22	36.33	97	6	CON	EXERCISE2	40	50
	466	37.37	37.21	36.36	109	6	CON	EXERCISE2	40	50
	467	37.36	37.21	36.38	110	6	CON	EXERCISE2	40	50
	468	37.36	37.18	36.42	116	6	CON	EXERCISE2	40	50
	469	37.35	37.15	36.46	117	6	CON	EXERCISE2	40	50
	470	37.35	37.16	36.49	116	6	CON	EXERCISE2	40	50
	471	37.34	37.17	36.49	117	6	CON	EXERCISE2	40	50
	472	37.33	37.19	36.48	119	6	CON	EXERCISE2	40	50
	473	37.33	37.20	36.50	117	6	CON	EXERCISE2	40	50
	474	37.34	37.20	36.53	115	6	CON	EXERCISE2	40	50
	475	37.33	37.11	36.54	116	6	CON	EXERCISE2	40	50
	476	37.33	36.80	36.56	112	6	CON	EXERCISE2	40	50
	477	37.34	36.69	36.59	117	6	CON	EXERCISE2	40	50
	478	37.34	36.92	36.60	120	6	CON	EXERCISE2	40	50
	479	37.35	37.05	36.61	120	6	CON	EXERCISE2	40	50
	480	37.35	37.08	36.62	119	6	CON	EXERCISE2	40	50
80	481	37.35	37.09	36.64	120	6	CON	EXERCISE2	40	50
	482	37.36	37.08	36.66	119	6	CON	EXERCISE2	40	50
	483	37.35	37.09	36.67	122	6	CON	EXERCISE2	40	50
	484	37.35	37.11	36.68	122	6	CON	EXERCISE2	40	50
	485	37.35	37.13	36.69	119	6	CON	EXERCISE2	40	50
	486	37.34	37.14	36.69	120	6	CON	EXERCISE2	40	50
	487	37.34	37.15	36.69	120	6	CON	EXERCISE2	40	50
	488	37.33	37.16	36.71	119	6	CON	EXERCISE2	40	50
	489	37.35	37.16	36.72	122	6	CON	EXERCISE2	40	50
	490	37.35	37.18	36.72	124	6	CON	EXERCISE2	40	50
	491	37.34	37.19	36.74	124	6	CON	EXERCISE2	40	50
	492	37.34	37.19	36.73	128	6	CON	EXERCISE2	40	50
	493	37.34	37.19	36.71	127	6	CON	EXERCISE2	40	50
	494	37.34	37.20	36.73	125	6	CON	EXERCISE2	40	50
	495	37.35	37.21	36.75	125	6	CON	EXERCISE2	40	50
	496	37.36	37.22	36.77	123	6	CON	EXERCISE2	40	50
	497	37.37	37.23	36.78	126	6	CON	EXERCISE2	40	50
	498	37.37	37.26	36.79	125	6	CON	EXERCISE2	40	50
	499	37.36	37.26	36.79	126	6	CON	EXERCISE2	40	50
	500	37.38	37.27	36.78	128	6	CON	EXERCISE2	40	50
	501	37.39	37.27	36.79	130	6	CON	EXERCISE2	40	50
	502	37.38	37.27	36.82	131	6	CON	EXERCISE2	40	50
	503	37.38	37.29	36.84	131	6	CON	EXERCISE2	40	50

min	number	Tre	Tes	Tsk-head	HR	ID	condition	period	Ta	RH
	504	37.37	37.29	36.85	131	6	CON	EXERCISE2	40	50
	505	37.37	37.30	36.87	130	6	CON	EXERCISE2	40	50
	506	37.38	37.30	36.87	130	6	CON	EXERCISE2	40	50
	507	37.37	37.28	36.88	129	6	CON	EXERCISE2	40	50
	508	37.37	37.28	36.88	129	6	CON	EXERCISE2	40	50
	509	37.38	37.27	36.89	131	6	CON	EXERCISE2	40	50
	510	37.38	37.30	36.93	130	6	CON	EXERCISE2	40	50
85	511	37.38	37.32	36.95	128	6	CON	EXERCISE2	40	50
	512	37.39	37.32	36.96	127	6	CON	EXERCISE2	40	50
	513	37.39	37.33	36.96	128	6	CON	EXERCISE2	40	50
	514	37.40	37.35	36.94	131	6	CON	EXERCISE2	40	50
	515	37.40	37.35	36.93	133	6	CON	EXERCISE2	40	50
	516	37.40	37.33	36.94	134	6	CON	EXERCISE2	40	50
	517	37.40	37.32	36.95	132	6	CON	EXERCISE2	40	50
	518	37.40	37.31	36.96	129	6	CON	EXERCISE2	40	50
	519	37.40	37.33	36.97	131	6	CON	EXERCISE2	40	50
	520	37.40	37.38	36.98	130	6	CON	EXERCISE2	40	50
	521	37.41	37.37	36.98	136	6	CON	EXERCISE2	40	50
	522	37.43	37.39	36.99	138	6	CON	EXERCISE2	40	50
	523	37.44	37.40	37.02	140	6	CON	EXERCISE2	40	50
	524	37.43	37.39	37.04	137	6	CON	EXERCISE2	40	50
	525	37.44	37.39	37.04	135	6	CON	EXERCISE2	40	50
	526	37.43	37.35	37.03	136	6	CON	EXERCISE2	40	50
	527	37.42	37.38	37.04	131	6	CON	EXERCISE2	40	50
	528	37.42	37.43	37.04	131	6	CON	EXERCISE2	40	50
	529	37.43	37.42	37.04	135	6	CON	EXERCISE2	40	50
	530	37.43	37.43	37.06	134	6	CON	EXERCISE2	40	50
	531	37.43	37.42	37.06	135	6	CON	EXERCISE2	40	50
	532	37.43	37.41	37.06	134	6	CON	EXERCISE2	40	50
	533	37.44	37.43	37.07	134	6	CON	EXERCISE2	40	50
	534	37.45	37.47	37.09	137	6	CON	EXERCISE2	40	50
	535	37.45	37.48	37.11	140	6	CON	EXERCISE2	40	50
	536	37.45	37.48	37.11	140	6	CON	EXERCISE2	40	50
	537	37.45	37.43	37.10	140	6	CON	EXERCISE2	40	50
	538	37.45	37.29	37.11	137	6	CON	EXERCISE2	40	50
	539	37.45	37.31	37.12	138	6	CON	EXERCISE2	40	50
	540	37.45	37.41	37.13	141	6	CON	EXERCISE2	40	50
90	541	37.46	37.42	37.14	139	6	CON	EXERCISE2	40	50
	542	37.47	37.43	37.15	138	6	CON	EXERCISE2	40	50
	543	37.47	37.45	37.16	139	6	CON	EXERCISE2	40	50
	544	37.47	37.48	37.17	139	6	CON	EXERCISE2	40	50
	545	37.47	37.44	37.18	141	6	CON	EXERCISE2	40	50
	546	37.48	37.45	37.19	141	6	CON	EXERCISE2	40	50
	547	37.48	37.52	37.19	140	6	CON	EXERCISE2	40	50
	548	37.49	37.55	37.18	144	6	CON	EXERCISE2	40	50
	549	37.49	37.54	37.17	146	6	CON	EXERCISE2	40	50

min	number	Tre	Tes	Tsk-head	HR	ID	condition	period	Ta	RH
	550	37.49	37.54	37.19	143	6	CON	EXERCISE2	40	50
	551	37.50	37.57	37.21	145	6	CON	EXERCISE2	40	50
	552	37.51	37.55	37.22	145	6	CON	EXERCISE2	40	50
	553	37.51	37.56	37.22	143	6	CON	EXERCISE2	40	50
	554	37.52	37.56	37.23	143	6	CON	EXERCISE2	40	50
	555	37.54	37.55	37.25	142	6	CON	EXERCISE2	40	50
	556	37.54	37.58	37.26	143	6	CON	EXERCISE2	40	50
	557	37.54	37.59	37.27	144	6	CON	EXERCISE2	40	50
	558	37.55	37.59	37.30	148	6	CON	EXERCISE2	40	50
	559	37.55	37.58	37.31	146	6	CON	EXERCISE2	40	50
	560	37.55	37.61	37.30	150	6	CON	EXERCISE2	40	50
	561	37.56	37.64	37.32	150	6	CON	EXERCISE2	40	50
	562	37.56	37.64	37.31	149	6	CON	EXERCISE2	40	50
	563	37.57	37.67	37.31	149	6	CON	EXERCISE2	40	50
	564	37.57	37.67	37.33	146	6	CON	EXERCISE2	40	50
	565	37.57	37.66	37.33	146	6	CON	EXERCISE2	40	50
	566	37.58	37.70	37.34	146	6	CON	EXERCISE2	40	50
	567	37.59	37.70	37.35	148	6	CON	EXERCISE2	40	50
	568	37.59	37.65	37.35	148	6	CON	EXERCISE2	40	50
	569	37.60	37.65	37.35	146	6	CON	EXERCISE2	40	50
	570	37.61	37.70	37.36	147	6	CON	EXERCISE2	40	50
95	571	37.62	37.73	37.36	150	6	CON	EXERCISE2	40	50
	572	37.63	37.73	37.37	150	6	CON	EXERCISE2	40	50
	573	37.63	37.73	37.39	148	6	CON	EXERCISE2	40	50
	574	37.63	37.69	37.38	149	6	CON	EXERCISE2	40	50
	575	37.64	37.71	37.37	150	6	CON	EXERCISE2	40	50
	576	37.65	37.76	37.37	150	6	CON	EXERCISE2	40	50
	577	37.66	37.75	37.39	152	6	CON	EXERCISE2	40	50
	578	37.67	37.77	37.40	150	6	CON	EXERCISE2	40	50
	579	37.67	37.80	37.41	150	6	CON	EXERCISE2	40	50
	580	37.68	37.81	37.41	150	6	CON	EXERCISE2	40	50
	581	37.68	37.82	37.42	150	6	CON	EXERCISE2	40	50
	582	37.69	37.83	37.42	150	6	CON	EXERCISE2	40	50
	583	37.69	37.85	37.40	150	6	CON	EXERCISE2	40	50
	584	37.70	37.86	37.41	150	6	CON	EXERCISE2	40	50
	585	37.70	37.85	37.43	150	6	CON	EXERCISE2	40	50
	586	37.70	37.85	37.43	151	6	CON	EXERCISE2	40	50
	587	37.71	37.88	37.43	153	6	CON	EXERCISE2	40	50
	588	37.72	37.81	37.46	150	6	CON	EXERCISE2	40	50
	589	37.72	37.78	37.48	151	6	CON	EXERCISE2	40	50
	590	37.73	37.82	37.48	151	6	CON	EXERCISE2	40	50
	591	37.73	37.85	37.48	153	6	CON	EXERCISE2	40	50
	592	37.74	37.87	37.49	154	6	CON	EXERCISE2	40	50
	593	37.74	37.86	37.50	154	6	CON	EXERCISE2	40	50
	594	37.74	37.89	37.50	152	6	CON	EXERCISE2	40	50
	595	37.74	37.85	37.51	154	6	CON	EXERCISE2	40	50

min	number	Tre	Tes	Tsk-head	HR	ID	condition	period	Ta	RH
	596	37.74	37.79	37.52	155	6	CON	EXERCISE2	40	50
	597	37.75	37.83	37.53	154	6	CON	EXERCISE2	40	50
	598	37.77	37.90	37.55	154	6	CON	EXERCISE2	40	50
	599	37.77	37.92	37.55	155	6	CON	EXERCISE2	40	50
	600	37.78	37.93	37.57	154	6	CON	EXERCISE2	40	50
100	601	37.79	37.95	37.58	154	6	CON	EXERCISE2	40	50
	602	37.79	37.92	37.58	154	6	CON	EXERCISE2	40	50
	603	37.80	37.92	37.60	150	6	CON	EXERCISE2	40	50
	604	37.80	37.95	37.61	154	6	CON	EXERCISE2	40	50
	605	37.81	37.98	37.59	156	6	CON	EXERCISE2	40	50
	606	37.82	38.00	37.59	156	6	CON	EXERCISE2	40	50
	607	37.82	37.99	37.60	157	6	CON	EXERCISE2	40	50
	608	37.82	38.00	37.61	158	6	CON	EXERCISE2	40	50
	609	37.83	38.04	37.61	157	6	CON	EXERCISE2	40	50
	610	37.84	38.06	37.61	156	6	CON	EXERCISE2	40	50
	611	37.84	38.06	37.63	155	6	CON	EXERCISE2	40	50
	612	37.84	38.07	37.64	154	6	CON	EXERCISE2	40	50
	613	37.84	38.06	37.64	154	6	CON	EXERCISE2	40	50
	614	37.85	38.05	37.65	157	6	CON	EXERCISE2	40	50
	615	37.85	38.04	37.66	172	6	CON	EXERCISE2	40	50
	616	37.86	38.04	37.66	153	6	CON	EXERCISE2	40	50
	617	37.86	38.07	37.67	156	6	CON	EXERCISE2	40	50
	618	37.87	38.09	37.68	157	6	CON	EXERCISE2	40	50
	619	37.88	38.09	37.69	157	6	CON	EXERCISE2	40	50
	620	37.88	38.09	37.69	153	6	CON	EXERCISE2	40	50
	621	37.88	38.13	37.68	155	6	CON	EXERCISE2	40	50
	622	37.89	38.11	37.67	157	6	CON	EXERCISE2	40	50
	623	37.90	38.05	37.68	157	6	CON	EXERCISE2	40	50
	624	37.90	38.07	37.69	161	6	CON	EXERCISE2	40	50
	625	37.90	38.12	37.68	162	6	CON	EXERCISE2	40	50
	626	37.91	38.12	37.68	161	6	CON	EXERCISE2	40	50
	627	37.91	38.10	37.70	158	6	CON	EXERCISE2	40	50
	628	37.91	38.11	37.72	157	6	CON	EXERCISE2	40	50
	629	37.92	38.12	37.72	153	6	CON	EXERCISE2	40	50
	630	37.93	38.09	37.73	158	6	CON	EXERCISE2	40	50
105	631	37.93	38.11	37.75	158	6	CON	EXERCISE2	40	50
	632	37.95	38.16	37.75	161	6	CON	EXERCISE2	40	50
	633	37.96	38.18	37.76	162	6	CON	EXERCISE2	40	50
	634	37.96	38.19	37.77	161	6	CON	EXERCISE2	40	50
	635	37.96	38.19	37.78	162	6	CON	EXERCISE2	40	50
	636	37.97	38.21	37.80	160	6	CON	EXERCISE2	40	50
	637	37.98	38.22	37.81	161	6	CON	EXERCISE2	40	50
	638	37.99	38.24	37.80	162	6	CON	EXERCISE2	40	50
	639	38.00	38.25	37.81	162	6	CON	EXERCISE2	40	50
	640	38.00	38.14	37.81	162	6	CON	EXERCISE2	40	50
	641	38.00	38.11	37.79	161	6	CON	EXERCISE2	40	50

min	number	Tre	Tes	Tsk-head	HR	ID	condition	period	Ta	RH
	642	38.01	38.20	37.79	160	6	CON	EXERCISE2	40	50
	643	38.02	38.24	37.81	158	6	CON	EXERCISE2	40	50
	644	38.03	38.25	37.84	157	6	CON	REST3	28	50
	645	38.04	38.32	37.69	156	6	CON	REST3	28	50
	646	38.03	38.35	37.48	149	6	CON	REST3	28	50
	647	38.02	38.30	37.45	142	6	CON	REST3	28	50
	648	38.02	38.32	37.45	140	6	CON	REST3	28	50
	649	38.03	38.32	37.40	142	6	CON	REST3	28	50
	650	38.03	38.31	37.33	138	6	CON	REST3	28	50
	651	38.03	38.32	37.32	137	6	CON	REST3	28	50
	652	38.04	38.34	37.33	132	6	CON	REST3	28	50
	653	38.04	38.36	37.27	130	6	CON	REST3	28	50
	654	38.04	38.36	37.15	127	6	CON	REST3	28	50
	655	38.04	38.35	37.03	129	6	CON	REST3	28	50
	656	38.04	38.34	36.82	131	6	CON	REST3	28	50
	657	38.05	38.35	36.73	130	6	CON	REST3	28	50
	658	38.05	38.35	36.83	128	6	CON	REST3	28	50
	659	38.04	38.35	36.85	125	6	CON	REST3	28	50
	660	38.04	38.34	36.86	127	6	CON	REST3	28	50
110	661	38.05	38.33	36.88	126	6	CON	REST3	28	50
	662	38.04	38.32	36.86	123	6	CON	REST3	28	50
	663	38.03	38.33	36.79	122	6	CON	REST3	28	50
	664	38.03	38.32	36.79	117	6	CON	REST3	28	50
	665	38.02	38.29	36.85	118	6	CON	REST3	28	50
	666	38.02	38.28	36.85	117	6	CON	REST3	28	50
	667	38.02	38.28	36.83	117	6	CON	REST3	28	50
	668	38.01	38.28	36.78	120	6	CON	REST3	28	50
	669	38.01	38.27	36.75	115	6	CON	REST3	28	50
	670	38.02	38.24	36.75	113	6	CON	REST3	28	50
	671	38.02	38.23	36.68	111	6	CON	REST3	28	50
	672	38.02	38.23	36.66	112	6	CON	REST3	28	50
	673	38.01	38.21	36.68	104	6	CON	REST3	28	50
	674	38.01	38.18	36.64	106	6	CON	REST3	28	50
	675	38.00	38.16	36.55	110	6	CON	REST3	28	50
	676	38.00	38.16	36.45	110	6	CON	REST3	28	50
	677	38.00	38.15	36.38	110	6	CON	REST3	28	50
	678	38.00	38.14	36.32	111	6	CON	REST3	28	50
	679	38.01	38.13	36.27	110	6	CON	REST3	28	50
	680	38.00	38.12	36.27	112	6	CON	REST3	28	50
	681	38.00	38.12	36.33	111	6	CON	REST3	28	50
	682	38.01	38.09	36.37	106	6	CON	REST3	28	50
	683	38.01	38.05	36.33	108	6	CON	REST3	28	50
	684	38.00	38.03	36.21	108	6	CON	REST3	28	50
	685	38.00	38.03	36.17	110	6	CON	REST3	28	50
	686	38.00	38.03	36.22	108	6	CON	REST3	28	50
	687	38.01	38.02	36.23	106	6	CON	REST3	28	50

min	number	Tre	Tes	Tsk-head	HR	ID	condition	period	Ta	RH
	688	38.01	38.00	36.18	109	6	CON	REST3	28	50
	689	38.01	38.00	36.18	104	6	CON	REST3	28	50
	690	38.01	37.93	36.22	106	6	CON	REST3	28	50
115	691	38.01	37.89	36.22	102	6	CON	REST3	28	50
	692	38.01	37.93	36.24	111	6	CON	REST3	28	50
	693	38.01	37.88	36.27	107	6	CON	REST3	28	50
	694	38.01	37.89	36.29	110	6	CON	REST3	28	50
	695	38.01	37.96	36.29	107	6	CON	REST3	28	50
	696	38.01	37.98	36.25	101	6	CON	REST3	28	50
	697	38.00	37.95	36.17	103	6	CON	REST3	28	50
	698	38.00	37.91	36.12	99	6	CON	REST3	28	50
	699	38.00	37.95	36.08	99	6	CON	REST3	28	50
	700	38.02	37.98	36.05	98	6	CON	REST3	28	50
	701	38.02	37.99	36.05	104	6	CON	REST3	28	50
	702	38.02	38.00	36.08	103	6	CON	REST3	28	50
	703	38.03	37.99	36.09	104	6	CON	REST3	28	50
	704	38.02	38.00	36.05	106	6	CON	REST3	28	50
	705	38.02	38.02	36.06	105	6	CON	REST3	28	50
	706	38.02	38.02	36.11	102	6	CON	REST3	28	50
	707	38.02	38.01	36.05	101	6	CON	REST3	28	50
	708	38.02	38.01	36.00	100	6	CON	REST3	28	50
	709	38.01	38.00	36.03	104	6	CON	REST3	28	50
0	1	36.94	36.59	35.48	84	7	CON	REST1	28	50
	2	36.94	36.58	35.48	74	7	CON	REST1	28	50
	3	36.94	36.55	35.49	75	7	CON	REST1	28	50
	4	36.93	36.52	35.49	75	7	CON	REST1	28	50
	5	36.92	36.53	35.48	75	7	CON	REST1	28	50
	6	36.92	36.54	35.48	74	7	CON	REST1	28	50
	7	36.93	36.55	35.49	76	7	CON	REST1	28	50
	8	36.92	36.54	35.49	78	7	CON	REST1	28	50
	9	36.92	36.53	35.49	76	7	CON	REST1	28	50
	10	36.92	36.55	35.49	79	7	CON	REST1	28	50
	11	36.91	36.51	35.49	76	7	CON	REST1	28	50
	12	36.90	36.44	35.50	87	7	CON	REST1	28	50
	13	36.90	36.47	35.49	80	7	CON	REST1	28	50
	14	36.90	36.51	35.49	72	7	CON	REST1	28	50
	15	36.91	36.52	35.50	72	7	CON	REST1	28	50
	16	36.91	36.52	35.50	74	7	CON	REST1	28	50
	17	36.91	36.52	35.50	74	7	CON	REST1	28	50
	18	36.90	36.52	35.49	76	7	CON	REST1	28	50
	19	36.90	36.52	35.49	74	7	CON	REST1	28	50
	20	36.90	36.44	35.49	80	7	CON	REST1	28	50
	21	36.90	36.42	35.49	74	7	CON	REST1	28	50
	22	36.90	36.49	35.51	72	7	CON	REST1	28	50
	23	36.90	36.51	35.51	77	7	CON	REST1	28	50
	24	36.90	36.52	35.51	78	7	CON	REST1	28	50

min	number	Tre	Tes	Tsk-head	HR	ID	condition	period	Ta	RH
	25	36.90	36.53	35.52	77	7	CON	REST1	28	50
	26	36.90	36.50	35.51	82	7	CON	REST1	28	50
	27	36.90	36.50	35.51	73	7	CON	REST1	28	50
	28	36.90	36.45	35.52	80	7	CON	REST1	28	50
	29	36.90	36.37	35.52	79	7	CON	REST1	28	50
	30	36.89	36.39	35.52	78	7	CON	REST1	28	50
5	31	36.89	36.41	35.53	76	7	CON	REST1	28	50
	32	36.90	36.44	35.52	75	7	CON	REST1	28	50
	33	36.90	36.46	35.50	75	7	CON	REST1	28	50
	34	36.90	36.45	35.50	75	7	CON	REST1	28	50
	35	36.90	36.44	35.48	74	7	CON	REST1	28	50
	36	36.90	36.44	35.47	78	7	CON	REST1	28	50
	37	36.90	36.45	35.47	68	7	CON	REST1	28	50
	38	36.90	36.44	35.47	72	7	CON	REST1	28	50
	39	36.90	36.41	35.48	79	7	CON	REST1	28	50
	40	36.91	36.42	35.50	78	7	CON	REST1	28	50
	41	36.91	36.44	35.51	74	7	CON	REST1	28	50
	42	36.90	36.42	35.51	74	7	CON	REST1	28	50
	43	36.90	36.39	35.51	86	7	CON	REST1	28	50
	44	36.89	36.35	35.49	75	7	CON	REST1	28	50
	45	36.89	36.34	35.46	72	7	CON	REST1	28	50
	46	36.89	36.36	35.45	67	7	CON	REST1	28	50
	47	36.89	36.38	35.46	79	7	CON	REST1	28	50
	48	36.88	36.40	35.46	77	7	CON	REST1	28	50
	49	36.88	36.41	35.45	76	7	CON	REST1	28	50
	50	36.88	36.42	35.46	80	7	CON	REST1	28	50
	51	36.88	36.42	35.46	83	7	CON	REST1	28	50
	52	36.87	36.39	35.45	74	7	CON	REST1	28	50
	53	36.86	36.38	35.45	76	7	CON	REST1	28	50
	54	36.86	36.41	35.46	72	7	CON	REST1	28	50
	55	36.86	36.41	35.46	77	7	CON	REST1	28	50
	56	36.86	36.40	35.46	78	7	CON	REST1	28	50
	57	36.86	36.44	35.47	77	7	CON	REST1	28	50
	58	36.86	36.45	35.48	78	7	CON	REST1	28	50
	59	36.86	36.45	35.47	78	7	CON	REST1	28	50
	60	36.85	36.45	35.46	77	7	CON	REST1	28	50
10	61	36.85	36.44	35.45	80	7	CON	REST1	28	50
	62	36.85	36.43	35.45	74	7	CON	REST1	28	50
	63	36.85	36.42	35.44	76	7	CON	REST1	28	50
	64	36.85	36.43	35.46	74	7	CON	REST1	28	50
	65	36.86	36.46	35.46	81	7	CON	REST1	28	50
	66	36.86	36.45	35.44	78	7	CON	REST1	28	50
	67	36.85	36.44	35.43	72	7	CON	REST1	28	50
	68	36.85	36.44	35.42	71	7	CON	REST1	28	50
	69	36.85	36.42	35.40	72	7	CON	REST1	28	50
	70	36.85	36.41	35.40	75	7	CON	REST1	28	50

min	number	Tre	Tes	Tsk-head	HR	ID	condition	period	Ta	RH
	71	36.84	36.43	35.41	79	7	CON	REST1	28	50
	72	36.84	36.43	35.42	77	7	CON	REST1	28	50
	73	36.84	36.41	35.43	77	7	CON	REST1	28	50
	74	36.85	36.43	35.43	76	7	CON	REST1	28	50
	75	36.85	36.45	35.44	78	7	CON	REST1	28	50
	76	36.86	36.44	35.46	75	7	CON	REST1	28	50
	77	36.85	36.41	35.46	80	7	CON	REST1	28	50
	78	36.84	36.39	35.47	80	7	CON	REST1	28	50
	79	36.84	36.39	35.46	74	7	CON	REST1	28	50
	80	36.84	36.39	35.45	77	7	CON	REST1	28	50
	81	36.84	36.36	35.46	74	7	CON	REST1	28	50
	82	36.84	36.33	35.45	71	7	CON	REST1	28	50
	83	36.84	36.32	35.45	72	7	CON	REST1	28	50
	84	36.83	36.34	35.45	78	7	CON	REST1	28	50
	85	36.84	36.37	35.45	73	7	CON	REST1	28	50
	86	36.85	36.36	35.45	80	7	CON	REST1	28	50
	87	36.84	36.35	35.45	78	7	CON	REST1	28	50
	88	36.84	36.37	35.45	80	7	CON	REST1	28	50
	89	36.86	36.39	35.46	93	7	CON	REST1	28	50
	90	36.86	36.39	35.47	76	7	CON	REST1	28	50
15	91	36.86	36.37	35.49	77	7	CON	REST1	28	50
	92	36.87	36.40	35.50	80	7	CON	REST1	28	50
	93	36.87	36.43	35.51	76	7	CON	REST1	28	50
	94	36.88	36.44	35.52	78	7	CON	REST1	28	50
	95	36.87	36.43	35.52	83	7	CON	REST1	28	50
	96	36.87	36.44	35.51	86	7	CON	REST1	28	50
	97	36.87	36.45	35.51	83	7	CON	REST1	28	50
	98	36.87	36.37	35.53	95	7	CON	REST1	28	50
	99	36.85	36.31	35.53	86	7	CON	REST1	28	50
	100	36.85	36.26	35.54	70	7	CON	REST1	28	50
	101	36.85	36.26	35.55	70	7	CON	REST1	28	50
	102	36.85	36.33	35.56	77	7	CON	REST1	28	50
	103	36.86	36.34	35.58	73	7	CON	REST1	40	50
	104	36.87	36.24	35.63	89	7	CON	REST1	40	50
	105	36.87	36.24	35.81	96	7	CON	REST1	40	50
	106	36.86	36.35	36.01	75	7	CON	REST1	40	50
	107	36.86	36.33	36.12	97	7	CON	REST1	40	50
	108	36.86	36.34	36.20	89	7	CON	REST1	40	50
	109	36.86	36.38	36.27	88	7	CON	REST1	40	50
	110	36.85	36.31	36.32	88	7	CON	REST1	40	50
	111	36.86	36.25	36.36	77	7	CON	REST1	40	50
	112	36.87	36.22	36.40	76	7	CON	REST1	40	50
	113	36.86	36.22	36.44	85	7	CON	REST1	40	50
	114	36.85	36.24	36.46	77	7	CON	REST1	40	50
	115	36.85	36.24	36.48	75	7	CON	REST1	40	50
	116	36.84	36.21	36.49	73	7	CON	REST1	40	50

min	number	Tre	Tes	Tsk-head	HR	ID	condition	period	Ta	RH
	117	36.85	36.26	36.51	77	7	CON	REST1	40	50
	118	36.85	36.35	36.54	71	7	CON	REST1	40	50
	119	36.85	36.35	36.56	75	7	CON	REST1	40	50
	120	36.84	36.34	36.57	81	7	CON	REST1	40	50
20	121	36.84	36.36	36.60	79	7	CON	REST1	40	50
	122	36.84	36.34	36.62	77	7	CON	REST1	40	50
	123	36.84	36.32	36.63	79	7	CON	REST1	40	50
	124	36.84	36.35	36.64	74	7	CON	REST1	40	50
	125	36.84	36.37	36.65	73	7	CON	REST1	40	50
	126	36.84	36.37	36.66	69	7	CON	REST1	40	50
	127	36.84	36.38	36.68	72	7	CON	REST1	40	50
	128	36.84	36.40	36.69	81	7	CON	REST1	40	50
	129	36.84	36.43	36.72	86	7	CON	REST1	40	50
	130	36.83	36.41	36.73	82	7	CON	REST1	40	50
	131	36.84	36.39	36.73	85	7	CON	REST1	40	50
	132	36.84	36.37	36.75	88	7	CON	REST1	40	50
	133	36.83	36.35	36.77	84	7	CON	REST1	40	50
	134	36.83	36.39	36.78	102	7	CON	REST1	40	50
	135	36.83	36.44	36.79	81	7	CON	REST1	40	50
	136	36.83	36.45	36.80	78	7	CON	REST1	40	50
	137	36.83	36.44	36.80	79	7	CON	REST1	40	50
	138	36.83	36.43	36.81	85	7	CON	REST1	40	50
	139	36.83	36.41	36.82	84	7	CON	EXERCISE1	40	50
	140	36.82	36.43	36.82	84	7	CON	EXERCISE1	40	50
	141	36.82	36.47	36.82	88	7	CON	EXERCISE1	40	50
	142	36.83	36.48	36.83	98	7	CON	EXERCISE1	40	50
	143	36.83	36.50	36.85	98	7	CON	EXERCISE1	40	50
	144	36.83	36.51	36.86	101	7	CON	EXERCISE1	40	50
	145	36.83	36.50	36.87	103	7	CON	EXERCISE1	40	50
	146	36.82	36.50	36.89	106	7	CON	EXERCISE1	40	50
	147	36.82	36.51	36.90	103	7	CON	EXERCISE1	40	50
	148	36.83	36.53	36.90	101	7	CON	EXERCISE1	40	50
	149	36.83	36.55	36.91	104	7	CON	EXERCISE1	40	50
	150	36.83	36.56	36.91	107	7	CON	EXERCISE1	40	50
25	151	36.83	36.57	36.93	103	7	CON	EXERCISE1	40	50
	152	36.83	36.56	36.94	103	7	CON	EXERCISE1	40	50
	153	36.82	36.56	36.95	105	7	CON	EXERCISE1	40	50
	154	36.82	36.57	36.96	105	7	CON	EXERCISE1	40	50
	155	36.82	36.57	36.96	103	7	CON	EXERCISE1	40	50
	156	36.82	36.56	36.96	104	7	CON	EXERCISE1	40	50
	157	36.82	36.54	36.97	106	7	CON	EXERCISE1	40	50
	158	36.82	36.52	36.98	104	7	CON	EXERCISE1	40	50
	159	36.83	36.53	36.97	105	7	CON	EXERCISE1	40	50
	160	36.83	36.54	36.97	106	7	CON	EXERCISE1	40	50
	161	36.83	36.53	36.97	105	7	CON	EXERCISE1	40	50
	162	36.84	36.55	36.98	103	7	CON	EXERCISE1	40	50

min	number	Tre	Tes	Tsk-head	HR	ID	condition	period	Ta	RH
	163	36.84	36.56	37.00	108	7	CON	EXERCISE1	40	50
	164	36.84	36.55	37.01	107	7	CON	EXERCISE1	40	50
	165	36.84	36.47	37.01	107	7	CON	EXERCISE1	40	50
	166	36.84	36.44	37.01	109	7	CON	EXERCISE1	40	50
	167	36.83	36.48	37.00	110	7	CON	EXERCISE1	40	50
	168	36.84	36.51	37.00	110	7	CON	EXERCISE1	40	50
	169	36.86	36.44	37.00	113	7	CON	EXERCISE1	40	50
	170	36.87	36.32	37.00	109	7	CON	EXERCISE1	40	50
	171	36.87	36.39	37.00	109	7	CON	EXERCISE1	40	50
	172	36.87	36.47	37.00	110	7	CON	EXERCISE1	40	50
	173	36.88	36.49	37.00	110	7	CON	EXERCISE1	40	50
	174	36.89	36.48	37.00	109	7	CON	EXERCISE1	40	50
	175	36.88	36.40	37.00	111	7	CON	EXERCISE1	40	50
	176	36.88	36.34	37.00	110	7	CON	EXERCISE1	40	50
	177	36.88	36.39	36.99	109	7	CON	EXERCISE1	40	50
	178	36.88	36.43	36.99	110	7	CON	EXERCISE1	40	50
	179	36.88	36.43	37.00	108	7	CON	EXERCISE1	40	50
	180	36.88	36.45	37.00	111	7	CON	EXERCISE1	40	50
30	181	36.89	36.43	37.00	112	7	CON	EXERCISE1	40	50
	182	36.89	36.38	37.00	111	7	CON	EXERCISE1	40	50
	183	36.88	36.34	37.00	108	7	CON	EXERCISE1	40	50
	184	36.88	36.41	36.99	108	7	CON	EXERCISE1	40	50
	185	36.89	36.44	36.98	111	7	CON	EXERCISE1	40	50
	186	36.89	36.40	36.99	110	7	CON	EXERCISE1	40	50
	187	36.88	36.41	36.98	108	7	CON	EXERCISE1	40	50
	188	36.89	36.43	36.97	108	7	CON	EXERCISE1	40	50
	189	36.89	36.43	36.98	112	7	CON	EXERCISE1	40	50
	190	36.89	36.44	36.97	107	7	CON	EXERCISE1	40	50
	191	36.90	36.45	36.98	110	7	CON	EXERCISE1	40	50
	192	36.90	36.47	36.98	111	7	CON	EXERCISE1	40	50
	193	36.90	36.46	36.96	111	7	CON	EXERCISE1	40	50
	194	36.90	36.47	36.96	112	7	CON	EXERCISE1	40	50
	195	36.91	36.45	36.96	114	7	CON	EXERCISE1	40	50
	196	36.91	36.32	36.97	111	7	CON	EXERCISE1	40	50
	197	36.92	36.33	36.97	111	7	CON	EXERCISE1	40	50
	198	36.93	36.42	36.97	113	7	CON	EXERCISE1	40	50
	199	36.93	36.42	36.96	115	7	CON	EXERCISE1	40	50
	200	36.94	36.42	36.95	118	7	CON	EXERCISE1	40	50
	201	36.95	36.45	36.92	113	7	CON	EXERCISE1	40	50
	202	36.95	36.42	36.91	115	7	CON	EXERCISE1	40	50
	203	36.95	36.42	36.92	116	7	CON	EXERCISE1	40	50
	204	36.95	36.48	36.92	111	7	CON	EXERCISE1	40	50
	205	36.95	36.52	36.93	111	7	CON	EXERCISE1	40	50
	206	36.95	36.53	36.93	113	7	CON	EXERCISE1	40	50
	207	36.95	36.54	36.92	115	7	CON	EXERCISE1	40	50
	208	36.96	36.57	36.93	115	7	CON	EXERCISE1	40	50

min	number	Tre	Tes	Tsk-head	HR	ID	condition	period	Ta	RH
	209	36.97	36.59	36.94	114	7	CON	EXERCISE1	40	50
	210	36.97	36.60	36.94	115	7	CON	EXERCISE1	40	50
35	211	36.97	36.60	36.96	113	7	CON	EXERCISE1	40	50
	212	36.97	36.61	36.97	114	7	CON	EXERCISE1	40	50
	213	36.96	36.61	36.95	115	7	CON	EXERCISE1	40	50
	214	36.96	36.61	36.95	113	7	CON	EXERCISE1	40	50
	215	36.97	36.62	36.95	114	7	CON	EXERCISE1	40	50
	216	36.97	36.62	36.96	118	7	CON	EXERCISE1	40	50
	217	36.97	36.61	36.96	117	7	CON	EXERCISE1	40	50
	218	36.97	36.62	36.96	117	7	CON	EXERCISE1	40	50
	219	36.98	36.64	36.96	119	7	CON	EXERCISE1	40	50
	220	36.99	36.64	36.96	116	7	CON	EXERCISE1	40	50
	221	36.99	36.62	36.96	116	7	CON	EXERCISE1	40	50
	222	37.00	36.62	36.97	115	7	CON	EXERCISE1	40	50
	223	37.00	36.60	36.98	116	7	CON	EXERCISE1	40	50
	224	36.99	36.59	36.99	119	7	CON	EXERCISE1	40	50
	225	36.99	36.62	36.99	118	7	CON	EXERCISE1	40	50
	226	37.00	36.61	37.00	117	7	CON	EXERCISE1	40	50
	227	37.01	36.58	37.00	117	7	CON	EXERCISE1	40	50
	228	37.01	36.59	37.00	118	7	CON	EXERCISE1	40	50
	229	37.01	36.57	37.00	113	7	CON	EXERCISE1	40	50
	230	37.01	36.55	37.00	116	7	CON	EXERCISE1	40	50
	231	37.01	36.61	37.00	116	7	CON	EXERCISE1	40	50
	232	37.02	36.62	37.01	116	7	CON	EXERCISE1	40	50
	233	37.02	36.61	37.01	120	7	CON	EXERCISE1	40	50
	234	37.03	36.56	37.02	118	7	CON	EXERCISE1	40	50
	235	37.03	36.56	37.02	134	7	CON	EXERCISE1	40	50
	236	37.03	36.56	37.04	116	7	CON	EXERCISE1	40	50
	237	37.04	36.52	37.06	116	7	CON	EXERCISE1	40	50
	238	37.05	36.57	37.05	117	7	CON	EXERCISE1	40	50
	239	37.05	36.62	37.05	118	7	CON	EXERCISE1	40	50
	240	37.05	36.61	37.06	118	7	CON	EXERCISE1	40	50
40	241	37.06	36.61	37.08	119	7	CON	EXERCISE1	40	50
	242	37.06	36.65	37.07	118	7	CON	EXERCISE1	40	50
	243	37.07	36.68	37.08	121	7	CON	EXERCISE1	40	50
	244	37.08	36.70	37.09	116	7	CON	EXERCISE1	40	50
	245	37.08	36.70	37.10	121	7	CON	EXERCISE1	40	50
	246	37.08	36.69	37.10	120	7	CON	EXERCISE1	40	50
	247	37.08	36.70	37.09	120	7	CON	EXERCISE1	40	50
	248	37.09	36.69	37.09	122	7	CON	EXERCISE1	40	50
	249	37.08	36.66	37.10	118	7	CON	EXERCISE1	40	50
	250	37.09	36.64	37.11	119	7	CON	EXERCISE1	40	50
	251	37.10	36.65	37.11	117	7	CON	EXERCISE1	40	50
	252	37.11	36.66	37.12	119	7	CON	EXERCISE1	40	50
	253	37.10	36.68	37.13	120	7	CON	EXERCISE1	40	50
	254	37.10	36.69	37.13	119	7	CON	EXERCISE1	40	50

min	number	Tre	Tes	Tsk-head	HR	ID	condition	period	Ta	RH
	255	37.10	36.69	37.13	119	7	CON	EXERCISE1	40	50
	256	37.11	36.70	37.14	120	7	CON	EXERCISE1	40	50
	257	37.11	36.68	37.15	120	7	CON	EXERCISE1	40	50
	258	37.10	36.70	37.15	120	7	CON	EXERCISE1	40	50
	259	37.10	36.71	37.15	119	7	CON	EXERCISE1	40	50
	260	37.11	36.63	37.15	121	7	CON	EXERCISE1	40	50
	261	37.12	36.63	37.16	127	7	CON	EXERCISE1	40	50
	262	37.12	36.69	37.17	120	7	CON	EXERCISE1	40	50
	263	37.12	36.73	37.17	124	7	CON	EXERCISE1	40	50
	264	37.12	36.75	37.18	122	7	CON	EXERCISE1	40	50
	265	37.13	36.77	37.18	119	7	CON	EXERCISE1	40	50
	266	37.13	36.78	37.18	121	7	CON	EXERCISE1	40	50
	267	37.14	36.78	37.19	121	7	CON	EXERCISE1	40	50
	268	37.13	36.77	37.19	116	7	CON	EXERCISE1	40	50
	269	37.13	36.78	37.20	119	7	CON	EXERCISE1	40	50
	270	37.13	36.79	37.21	121	7	CON	EXERCISE1	40	50
45	271	37.14	36.78	37.21	121	7	CON	EXERCISE1	40	50
	272	37.14	36.80	37.21	119	7	CON	EXERCISE1	40	50
	273	37.15	36.81	37.23	120	7	CON	EXERCISE1	40	50
	274	37.15	36.79	37.22	122	7	CON	EXERCISE1	40	50
	275	37.15	36.79	37.23	124	7	CON	EXERCISE1	40	50
	276	37.16	36.81	37.24	121	7	CON	EXERCISE1	40	50
	277	37.17	36.84	37.24	122	7	CON	EXERCISE1	40	50
	278	37.17	36.84	37.24	125	7	CON	EXERCISE1	40	50
	279	37.18	36.82	37.27	125	7	CON	EXERCISE1	40	50
	280	37.18	36.78	37.27	121	7	CON	EXERCISE1	40	50
	281	37.18	36.78	37.26	120	7	CON	EXERCISE1	40	50
	282	37.18	36.81	37.25	126	7	CON	EXERCISE1	40	50
	283	37.17	36.85	37.25	124	7	CON	EXERCISE1	40	50
	284	37.17	36.88	37.26	122	7	CON	EXERCISE1	40	50
	285	37.18	36.89	37.27	122	7	CON	EXERCISE1	40	50
	286	37.18	36.89	37.28	123	7	CON	EXERCISE1	40	50
	287	37.19	36.90	37.28	123	7	CON	EXERCISE1	40	50
	288	37.20	36.89	37.28	126	7	CON	EXERCISE1	40	50
	289	37.20	36.89	37.29	124	7	CON	EXERCISE1	40	50
	290	37.20	36.85	37.29	121	7	CON	EXERCISE1	40	50
	291	37.21	36.84	37.29	126	7	CON	EXERCISE1	40	50
	292	37.22	36.89	37.29	127	7	CON	EXERCISE1	40	50
	293	37.22	36.90	37.29	126	7	CON	EXERCISE1	40	50
	294	37.22	36.92	37.30	125	7	CON	EXERCISE1	40	50
	295	37.23	36.91	37.31	126	7	CON	EXERCISE1	40	50
	296	37.23	36.89	37.30	125	7	CON	EXERCISE1	40	50
	297	37.23	36.90	37.30	124	7	CON	EXERCISE1	40	50
	298	37.24	36.90	37.31	124	7	CON	EXERCISE1	40	50
	299	37.24	36.88	37.32	123	7	CON	EXERCISE1	40	50
	300	37.24	36.90	37.32	124	7	CON	EXERCISE1	40	50

min	number	Tre	Tes	Tsk-head	HR	ID	condition	period	Ta	RH
50	301	37.24	36.92	37.33	126	7	CON	EXERCISE1	40	50
	302	37.24	36.93	37.33	127	7	CON	EXERCISE1	40	50
	303	37.24	36.94	37.33	126	7	CON	EXERCISE1	40	50
	304	37.25	36.91	37.32	125	7	CON	EXERCISE1	40	50
	305	37.25	36.91	37.32	127	7	CON	EXERCISE1	40	50
	306	37.26	36.92	37.33	126	7	CON	EXERCISE1	40	50
	307	37.27	36.83	37.34	123	7	CON	EXERCISE1	40	50
	308	37.27	36.74	37.32	125	7	CON	EXERCISE1	40	50
	309	37.27	36.79	37.32	131	7	CON	EXERCISE1	40	50
	310	37.27	36.78	37.33	128	7	CON	EXERCISE1	40	50
	311	37.28	36.82	37.34	128	7	CON	EXERCISE1	40	50
	312	37.28	36.93	37.34	130	7	CON	EXERCISE1	40	50
	313	37.29	36.93	37.34	127	7	CON	EXERCISE1	40	50
	314	37.29	36.92	37.34	128	7	CON	EXERCISE1	40	50
	315	37.30	36.94	37.35	128	7	CON	EXERCISE1	40	50
	316	37.31	36.95	37.36	126	7	CON	EXERCISE1	40	50
	317	37.31	36.92	37.36	126	7	CON	EXERCISE1	40	50
	318	37.31	36.94	37.37	126	7	CON	EXERCISE1	40	50
	319	37.30	36.99	37.38	124	7	CON	EXERCISE1	40	50
	320	37.31	36.91	37.37	125	7	CON	REST2	28	50
	321	37.32	36.85	37.27	122	7	CON	REST2	28	50
	322	37.32	36.88	37.17	116	7	CON	REST2	28	50
	323	37.32	36.90	37.14	104	7	CON	REST2	28	50
	324	37.32	36.96	35.92	106	7	CON	REST2	28	50
	325	37.32	37.03	33.60	106	7	CON	REST2	28	50
	326	37.32	37.05	32.44	104	7	CON	REST2	28	50
	327	37.34	37.07	32.43	100	7	CON	REST2	28	50
	328	37.33	37.10	32.35	96	7	CON	REST2	28	50
	329	37.33	37.10	32.13	92	7	CON	REST2	28	50
	330	37.34	37.09	32.06	99	7	CON	REST2	28	50
55	331	37.34	37.09	32.05	99	7	CON	REST2	28	50
	332	37.34	37.03	32.01	113	7	CON	REST2	28	50
	333	37.35	36.92	31.63	113	7	CON	REST2	28	50
	334	37.35	36.92	32.53	107	7	CON	REST2	28	50
	335	37.36	36.95	34.63	104	7	CON	REST2	28	50
	336	37.37	36.89	35.59	96	7	CON	REST2	28	50
	337	37.38	36.85	35.79	97	7	CON	REST2	28	50
	338	37.38	36.86	34.21	96	7	CON	REST2	28	50
	339	37.38	36.84	34.09	98	7	CON	REST2	28	50
	340	37.38	36.83	35.69	98	7	CON	REST2	28	50
	341	37.38	33.32	35.92	106	7	CON	REST2	28	50
	342	37.39	31.22	36.12	106	7	CON	REST2	28	50
	343	37.39	31.44	36.20	97	7	CON	REST2	28	50
	344	37.38	30.03	36.23	96	7	CON	REST2	28	50
	345	37.38	29.91	36.26	90	7	CON	REST2	28	50
	346	37.38	31.83	36.28	94	7	CON	REST2	28	50

min	number	Tre	Tes	Tsk-head	HR	ID	condition	period	Ta	RH
	347	37.39	33.59	36.30	98	7	CON	REST2	28	50
	348	37.40	31.79	36.28	90	7	CON	REST2	28	50
	349	37.40	30.75	36.25	99	7	CON	REST2	28	50
	350	37.40	30.83	36.26	89	7	CON	REST2	28	50
	351	37.40	31.82	36.28	94	7	CON	REST2	28	50
	352	37.40	33.34	36.25	93	7	CON	REST2	28	50
	353	37.41	32.74	36.23	104	7	CON	REST2	28	50
	354	37.41	32.76	36.25	91	7	CON	REST2	28	50
	355	37.42	31.10	36.25	88	7	CON	REST2	28	50
	356	37.43	30.78	36.25	89	7	CON	REST2	28	50
	357	37.42	32.38	36.26	100	7	CON	REST2	28	50
	358	37.41	32.65	36.24	87	7	CON	REST2	28	50
	359	37.41	31.98	36.23	85	7	CON	REST2	28	50
	360	37.41	30.54	36.21	91	7	CON	REST2	28	50
60	361	37.42	31.42	36.21	100	7	CON	REST2	28	50
	362	37.42	33.01	36.24	93	7	CON	REST2	28	50
	363	37.41	32.89	36.27	92	7	CON	REST2	28	50
	364	37.41	31.32	36.27	90	7	CON	REST2	28	50
	365	37.40	30.27	36.25	85	7	CON	REST2	28	50
	366	37.40	31.69	36.23	84	7	CON	REST2	28	50
	367	37.41	33.25	36.25	90	7	CON	REST2	28	50
	368	37.41	33.53	36.24	87	7	CON	REST2	28	50
	369	37.41	33.95	36.20	77	7	CON	REST2	28	50
	370	37.41	34.21	36.18	95	7	CON	REST2	28	50
	371	37.41	34.39	36.17	90	7	CON	REST2	28	50
	372	37.42	34.55	36.16	82	7	CON	REST2	28	50
	373	37.41	34.67	36.16	81	7	CON	REST2	28	50
	374	37.41	34.79	36.14	79	7	CON	REST2	28	50
	375	37.41	34.93	36.12	87	7	CON	REST2	28	50
	376	37.41	35.04	36.12	81	7	CON	REST2	28	50
	377	37.42	35.11	36.12	81	7	CON	REST2	28	50
	378	37.41	35.16	36.11	82	7	CON	REST2	28	50
	379	37.41	35.27	36.10	83	7	CON	REST2	28	50
	380	37.41	35.37	36.11	84	7	CON	REST2	28	50
	381	37.41	35.41	36.12	86	7	CON	REST2	28	50
	382	37.41	35.46	36.11	83	7	CON	REST2	28	50
	383	37.41	35.52	36.10	82	7	CON	REST2	28	50
	384	37.41	35.58	36.10	83	7	CON	REST2	28	50
	385	37.42	35.63	36.10	85	7	CON	REST2	28	50
	386	37.43	35.66	36.10	77	7	CON	REST2	28	50
	387	37.42	35.68	36.11	89	7	CON	REST2	28	50
	388	37.41	35.72	36.11	85	7	CON	REST2	28	50
	389	37.41	35.75	36.10	78	7	CON	REST2	28	50
	390	37.41	35.81	36.09	79	7	CON	REST2	28	50
65	391	37.42	35.86	36.08	80	7	CON	REST2	28	50
	392	37.41	35.89	36.07	82	7	CON	REST2	28	50

min	number	Tre	Tes	Tsk-head	HR	ID	condition	period	Ta	RH
	393	37.41	35.93	36.07	78	7	CON	REST2	28	50
	394	37.41	35.96	36.06	95	7	CON	REST2	28	50
	395	37.41	35.99	36.05	83	7	CON	REST2	28	50
	396	37.42	36.03	36.04	86	7	CON	REST2	28	50
	397	37.42	36.05	36.03	84	7	CON	REST2	28	50
	398	37.41	36.07	36.03	79	7	CON	REST2	28	50
	399	37.40	36.09	36.04	82	7	CON	REST2	28	50
	400	37.41	36.11	36.04	75	7	CON	REST2	28	50
	401	37.41	36.15	36.01	75	7	CON	REST2	28	50
	402	37.41	36.16	36.00	75	7	CON	REST2	28	50
	403	37.40	36.16	35.99	82	7	CON	REST2	28	50
	404	37.40	36.21	35.97	79	7	CON	REST2	28	50
	405	37.40	36.26	35.95	78	7	CON	REST2	28	50
	406	37.41	36.28	35.96	77	7	CON	REST2	28	50
	407	37.40	36.30	35.98	77	7	CON	REST2	28	50
	408	37.40	36.31	35.99	76	7	CON	REST2	28	50
	409	37.40	36.32	35.99	76	7	CON	REST2	28	50
	410	37.40	36.34	35.99	77	7	CON	REST2	28	50
	411	37.40	36.35	35.99	81	7	CON	REST2	28	50
	412	37.40	36.37	35.99	77	7	CON	REST2	28	50
	413	37.40	36.38	35.99	78	7	CON	REST2	28	50
	414	37.39	36.32	35.99	75	7	CON	REST2	28	50
	415	37.38	36.30	35.98	79	7	CON	REST2	28	50
	416	37.38	36.34	35.98	85	7	CON	REST2	28	50
	417	37.38	36.34	35.98	84	7	CON	REST2	28	50
	418	37.38	36.34	36.01	94	7	CON	REST2	28	50
	419	37.37	36.34	36.02	100	7	CON	REST2	28	50
	420	37.37	36.34	36.02	75	7	CON	REST2	28	50
70	421	37.37	36.36	36.02	72	7	CON	REST2	28	50
	422	37.38	36.37	36.00	77	7	CON	REST2	28	50
	423	37.38	36.39	35.99	76	7	CON	REST2	28	50
	424	37.37	36.38	36.00	85	7	CON	REST2	28	50
	425	37.36	36.38	36.02	85	7	CON	REST2	28	50
	426	37.36	36.43	36.04	84	7	CON	REST2	28	50
	427	37.36	36.46	36.04	85	7	CON	REST2	28	50
	428	37.36	36.46	36.05	81	7	CON	REST2	28	50
	429	37.36	36.46	36.08	82	7	CON	REST2	28	50
	430	37.36	36.46	36.10	81	7	CON	REST2	28	50
	431	37.36	36.47	36.12	81	7	CON	REST2	28	50
	432	37.36	36.46	36.12	85	7	CON	REST2	28	50
	433	37.35	36.45	36.12	89	7	CON	REST2	28	50
	434	37.35	36.49	36.13	86	7	CON	REST2	28	50
	435	37.35	36.49	36.13	85	7	CON	REST2	28	50
	436	37.34	36.43	36.12	96	7	CON	REST2	28	50
	437	37.34	36.44	36.10	89	7	CON	REST2	28	50
	438	37.33	36.48	36.08	84	7	CON	REST2	28	50

min	number	Tre	Tes	Tsk-head	HR	ID	condition	period	Ta	RH
	439	37.33	36.47	36.06	82	7	CON	REST2	40	50
	440	37.33	36.32	36.09	102	7	CON	REST2	40	50
	441	37.34	36.37	36.22	90	7	CON	REST2	40	50
	442	37.35	36.55	36.36	88	7	CON	REST2	40	50
	443	37.35	36.53	36.45	83	7	CON	REST2	40	50
	444	37.35	36.49	36.50	81	7	CON	REST2	40	50
	445	37.34	36.48	36.54	91	7	CON	REST2	40	50
	446	37.33	36.47	36.58	81	7	CON	REST2	40	50
	447	37.33	36.47	36.62	82	7	CON	REST2	40	50
	448	37.34	36.49	36.67	82	7	CON	REST2	40	50
	449	37.35	36.50	36.71	86	7	CON	REST2	40	50
	450	37.34	36.50	36.74	80	7	CON	REST2	40	50
75	451	37.34	36.51	36.77	77	7	CON	REST2	40	50
	452	37.34	36.56	36.79	86	7	CON	REST2	40	50
	453	37.35	36.60	36.80	74	7	CON	REST2	40	50
	454	37.34	36.60	36.81	79	7	CON	REST2	40	50
	455	37.34	36.60	36.82	85	7	CON	REST2	40	50
	456	37.34	36.62	36.83	83	7	CON	REST2	40	50
	457	37.34	36.62	36.84	79	7	CON	REST2	40	50
	458	37.33	36.60	36.85	106	7	CON	REST2	40	50
	459	37.34	36.63	36.84	97	7	CON	REST2	40	50
	460	37.34	36.66	36.85	89	7	CON	REST2	40	50
	461	37.34	36.65	36.86	83	7	CON	REST2	40	50
	462	37.34	36.64	36.86	85	7	CON	REST2	40	50
	463	37.34	36.67	36.86	83	7	CON	EXERCISE2	40	50
	464	37.34	36.65	36.87	88	7	CON	EXERCISE2	40	50
	465	37.33	36.63	36.88	95	7	CON	EXERCISE2	40	50
	466	37.34	36.66	36.90	105	7	CON	EXERCISE2	40	50
	467	37.34	36.68	36.91	108	7	CON	EXERCISE2	40	50
	468	37.34	36.68	36.91	99	7	CON	EXERCISE2	40	50
	469	37.35	36.68	36.92	107	7	CON	EXERCISE2	40	50
	470	37.35	36.63	36.93	109	7	CON	EXERCISE2	40	50
	471	37.35	36.64	36.94	112	7	CON	EXERCISE2	40	50
	472	37.34	36.70	36.95	111	7	CON	EXERCISE2	40	50
	473	37.34	36.72	36.95	112	7	CON	EXERCISE2	40	50
	474	37.34	36.73	36.96	114	7	CON	EXERCISE2	40	50
	475	37.34	36.73	36.98	115	7	CON	EXERCISE2	40	50
	476	37.35	36.74	37.00	115	7	CON	EXERCISE2	40	50
	477	37.35	36.74	37.00	111	7	CON	EXERCISE2	40	50
	478	37.35	36.77	37.01	111	7	CON	EXERCISE2	40	50
	479	37.35	36.80	37.02	115	7	CON	EXERCISE2	40	50
	480	37.35	36.79	37.02	110	7	CON	EXERCISE2	40	50
80	481	37.36	36.77	37.04	112	7	CON	EXERCISE2	40	50
	482	37.35	36.75	37.05	114	7	CON	EXERCISE2	40	50
	483	37.35	36.74	37.05	114	7	CON	EXERCISE2	40	50
	484	37.35	36.76	37.05	113	7	CON	EXERCISE2	40	50

min	number	Tre	Tes	Tsk-head	HR	ID	condition	period	Ta	RH
	485	37.36	36.79	37.06	116	7	CON	EXERCISE2	40	50
	486	37.36	36.80	37.08	115	7	CON	EXERCISE2	40	50
	487	37.36	36.79	37.10	118	7	CON	EXERCISE2	40	50
	488	37.34	36.77	37.09	117	7	CON	EXERCISE2	40	50
	489	37.34	36.77	37.09	121	7	CON	EXERCISE2	40	50
	490	37.36	36.77	37.11	118	7	CON	EXERCISE2	40	50
	491	37.37	36.77	37.12	115	7	CON	EXERCISE2	40	50
	492	37.38	36.77	37.13	118	7	CON	EXERCISE2	40	50
	493	37.38	36.77	37.13	117	7	CON	EXERCISE2	40	50
	494	37.38	36.76	37.14	117	7	CON	EXERCISE2	40	50
	495	37.38	36.73	37.15	116	7	CON	EXERCISE2	40	50
	496	37.38	36.76	37.16	118	7	CON	EXERCISE2	40	50
	497	37.37	36.80	37.16	116	7	CON	EXERCISE2	40	50
	498	37.37	36.78	37.18	119	7	CON	EXERCISE2	40	50
	499	37.36	36.77	37.18	121	7	CON	EXERCISE2	40	50
	500	37.36	36.78	37.19	119	7	CON	EXERCISE2	40	50
	501	37.37	36.77	37.21	120	7	CON	EXERCISE2	40	50
	502	37.37	36.76	37.22	119	7	CON	EXERCISE2	40	50
	503	37.37	36.77	37.22	118	7	CON	EXERCISE2	40	50
	504	37.38	36.77	37.23	118	7	CON	EXERCISE2	40	50
	505	37.39	36.78	37.23	120	7	CON	EXERCISE2	40	50
	506	37.40	36.79	37.24	120	7	CON	EXERCISE2	40	50
	507	37.41	36.79	37.24	121	7	CON	EXERCISE2	40	50
	508	37.41	36.79	37.24	120	7	CON	EXERCISE2	40	50
	509	37.41	36.80	37.25	122	7	CON	EXERCISE2	40	50
	510	37.41	36.81	37.26	120	7	CON	EXERCISE2	40	50
85	511	37.41	36.79	37.26	122	7	CON	EXERCISE2	40	50
	512	37.40	36.72	37.27	119	7	CON	EXERCISE2	40	50
	513	37.40	36.73	37.27	128	7	CON	EXERCISE2	40	50
	514	37.41	36.81	37.28	119	7	CON	EXERCISE2	40	50
	515	37.42	36.84	37.29	119	7	CON	EXERCISE2	40	50
	516	37.42	36.85	37.30	120	7	CON	EXERCISE2	40	50
	517	37.42	36.83	37.32	119	7	CON	EXERCISE2	40	50
	518	37.43	36.84	37.32	118	7	CON	EXERCISE2	40	50
	519	37.44	36.86	37.32	123	7	CON	EXERCISE2	40	50
	520	37.44	36.85	37.33	123	7	CON	EXERCISE2	40	50
	521	37.44	36.86	37.34	124	7	CON	EXERCISE2	40	50
	522	37.44	36.88	37.35	122	7	CON	EXERCISE2	40	50
	523	37.45	36.84	37.34	124	7	CON	EXERCISE2	40	50
	524	37.44	36.78	37.35	120	7	CON	EXERCISE2	40	50
	525	37.44	36.75	37.35	127	7	CON	EXERCISE2	40	50
	526	37.45	36.78	37.35	125	7	CON	EXERCISE2	40	50
	527	37.45	36.78	37.35	125	7	CON	EXERCISE2	40	50
	528	37.45	36.75	37.36	123	7	CON	EXERCISE2	40	50
	529	37.45	36.78	37.37	125	7	CON	EXERCISE2	40	50
	530	37.46	36.83	37.39	123	7	CON	EXERCISE2	40	50

min	number	Tre	Tes	Tsk-head	HR	ID	condition	period	Ta	RH
	531	37.48	36.84	37.40	123	7	CON	EXERCISE2	40	50
	532	37.47	36.87	37.40	125	7	CON	EXERCISE2	40	50
	533	37.47	36.91	37.40	139	7	CON	EXERCISE2	40	50
	534	37.48	36.90	37.40	125	7	CON	EXERCISE2	40	50
	535	37.48	36.88	37.41	124	7	CON	EXERCISE2	40	50
	536	37.48	36.86	37.43	124	7	CON	EXERCISE2	40	50
	537	37.49	36.89	37.44	128	7	CON	EXERCISE2	40	50
	538	37.49	36.93	37.43	122	7	CON	EXERCISE2	40	50
	539	37.49	36.96	37.43	123	7	CON	EXERCISE2	40	50
	540	37.50	36.97	37.43	126	7	CON	EXERCISE2	40	50
90	541	37.50	36.95	37.45	123	7	CON	EXERCISE2	40	50
	542	37.51	36.93	37.45	123	7	CON	EXERCISE2	40	50
	543	37.51	36.93	37.46	133	7	CON	EXERCISE2	40	50
	544	37.52	36.96	37.47	126	7	CON	EXERCISE2	40	50
	545	37.52	36.97	37.47	125	7	CON	EXERCISE2	40	50
	546	37.53	36.97	37.48	126	7	CON	EXERCISE2	40	50
	547	37.53	36.98	37.49	123	7	CON	EXERCISE2	40	50
	548	37.53	37.00	37.49	124	7	CON	EXERCISE2	40	50
	549	37.54	37.02	37.49	123	7	CON	EXERCISE2	40	50
	550	37.55	37.01	37.50	123	7	CON	EXERCISE2	40	50
	551	37.55	36.96	37.50	123	7	CON	EXERCISE2	40	50
	552	37.55	36.97	37.52	129	7	CON	EXERCISE2	40	50
	553	37.55	37.01	37.53	126	7	CON	EXERCISE2	40	50
	554	37.56	36.92	37.52	125	7	CON	EXERCISE2	40	50
	555	37.56	36.78	37.51	127	7	CON	EXERCISE2	40	50
	556	37.56	36.81	37.51	129	7	CON	EXERCISE2	40	50
	557	37.56	36.86	37.52	134	7	CON	EXERCISE2	40	50
	558	37.56	36.79	37.53	133	7	CON	EXERCISE2	40	50
	559	37.57	36.75	37.54	134	7	CON	EXERCISE2	40	50
	560	37.56	36.68	37.54	132	7	CON	EXERCISE2	40	50
	561	37.56	36.72	37.55	133	7	CON	EXERCISE2	40	50
	562	37.56	36.90	37.56	129	7	CON	EXERCISE2	40	50
	563	37.57	36.99	37.57	131	7	CON	EXERCISE2	40	50
	564	37.59	37.03	37.59	124	7	CON	EXERCISE2	40	50
	565	37.60	37.07	37.59	125	7	CON	EXERCISE2	40	50
	566	37.60	37.04	37.59	129	7	CON	EXERCISE2	40	50
	567	37.61	36.78	37.59	126	7	CON	EXERCISE2	40	50
	568	37.62	36.77	37.59	133	7	CON	EXERCISE2	40	50
	569	37.62	36.96	37.60	127	7	CON	EXERCISE2	40	50
	570	37.63	36.91	37.61	125	7	CON	EXERCISE2	40	50
95	571	37.63	36.92	37.62	127	7	CON	EXERCISE2	40	50
	572	37.63	36.97	37.62	131	7	CON	EXERCISE2	40	50
	573	37.64	37.00	37.61	129	7	CON	EXERCISE2	40	50
	574	37.64	37.06	37.62	127	7	CON	EXERCISE2	40	50
	575	37.65	37.09	37.63	128	7	CON	EXERCISE2	40	50
	576	37.65	37.07	37.64	126	7	CON	EXERCISE2	40	50

min	number	Tre	Tes	Tsk-head	HR	ID	condition	period	Ta	RH
	577	37.66	37.06	37.64	129	7	CON	EXERCISE2	40	50
	578	37.66	37.08	37.64	131	7	CON	EXERCISE2	40	50
	579	37.67	37.06	37.66	134	7	CON	EXERCISE2	40	50
	580	37.68	37.04	37.66	129	7	CON	EXERCISE2	40	50
	581	37.69	37.07	37.66	131	7	CON	EXERCISE2	40	50
	582	37.69	37.09	37.67	129	7	CON	EXERCISE2	40	50
	583	37.69	37.08	37.67	131	7	CON	EXERCISE2	40	50
	584	37.69	37.10	37.68	135	7	CON	EXERCISE2	40	50
	585	37.70	37.13	37.69	136	7	CON	EXERCISE2	40	50
	586	37.71	37.15	37.69	134	7	CON	EXERCISE2	40	50
	587	37.72	37.12	37.70	140	7	CON	EXERCISE2	40	50
	588	37.72	37.06	37.70	131	7	CON	EXERCISE2	40	50
	589	37.71	37.07	37.70	131	7	CON	EXERCISE2	40	50
	590	37.71	37.11	37.70	131	7	CON	EXERCISE2	40	50
	591	37.72	37.14	37.71	130	7	CON	EXERCISE2	40	50
	592	37.73	37.15	37.71	133	7	CON	EXERCISE2	40	50
	593	37.74	37.17	37.72	158	7	CON	EXERCISE2	40	50
	594	37.74	37.19	37.73	135	7	CON	EXERCISE2	40	50
	595	37.74	37.19	37.73	134	7	CON	EXERCISE2	40	50
	596	37.75	37.19	37.74	132	7	CON	EXERCISE2	40	50
	597	37.74	37.19	37.74	138	7	CON	EXERCISE2	40	50
	598	37.72	37.19	37.75	138	7	CON	EXERCISE2	40	50
	599	37.72	37.18	37.76	147	7	CON	EXERCISE2	40	50
	600	37.75	37.19	37.77	138	7	CON	EXERCISE2	40	50
100	601	37.76	37.18	37.78	133	7	CON	EXERCISE2	40	50
	602	37.76	37.18	37.78	134	7	CON	EXERCISE2	40	50
	603	37.78	37.20	37.78	134	7	CON	EXERCISE2	40	50
	604	37.79	37.22	37.78	135	7	CON	EXERCISE2	40	50
	605	37.79	37.23	37.79	137	7	CON	EXERCISE2	40	50
	606	37.79	37.23	37.78	134	7	CON	EXERCISE2	40	50
	607	37.80	37.24	37.78	144	7	CON	EXERCISE2	40	50
	608	37.80	37.26	37.79	139	7	CON	EXERCISE2	40	50
	609	37.80	37.25	37.80	134	7	CON	EXERCISE2	40	50
	610	37.80	37.23	37.81	132	7	CON	EXERCISE2	40	50
	611	37.81	37.16	37.82	154	7	CON	EXERCISE2	40	50
	612	37.82	37.11	37.81	132	7	CON	EXERCISE2	40	50
	613	37.83	37.16	37.81	139	7	CON	EXERCISE2	40	50
	614	37.84	37.19	37.82	136	7	CON	EXERCISE2	40	50
	615	37.83	37.21	37.82	135	7	CON	EXERCISE2	40	50
	616	37.82	37.23	37.83	140	7	CON	EXERCISE2	40	50
	617	37.83	37.17	37.83	139	7	CON	EXERCISE2	40	50
	618	37.86	37.18	37.83	158	7	CON	EXERCISE2	40	50
	619	37.86	37.27	37.83	138	7	CON	EXERCISE2	40	50
	620	37.85	37.23	37.84	140	7	CON	EXERCISE2	40	50
	621	37.85	37.24	37.85	136	7	CON	EXERCISE2	40	50
	622	37.86	37.32	37.85	136	7	CON	EXERCISE2	40	50

min	number	Tre	Tes	Tsk-head	HR	ID	condition	period	Ta	RH
	623	37.87	37.35	37.86	133	7	CON	EXERCISE2	40	50
	624	37.87	37.35	37.86	133	7	CON	EXERCISE2	40	50
	625	37.88	37.37	37.86	134	7	CON	EXERCISE2	40	50
	626	37.89	37.38	37.87	135	7	CON	EXERCISE2	40	50
	627	37.90	37.37	37.89	137	7	CON	EXERCISE2	40	50
	628	37.91	37.38	37.90	136	7	CON	EXERCISE2	40	50
	629	37.91	37.41	37.89	138	7	CON	EXERCISE2	40	50
	630	37.91	37.41	37.89	136	7	CON	EXERCISE2	40	50
105	631	37.91	37.39	37.90	136	7	CON	EXERCISE2	40	50
	632	37.91	37.37	37.89	140	7	CON	EXERCISE2	40	50
	633	37.91	37.32	37.90	158	7	CON	EXERCISE2	40	50
	634	37.91	37.32	37.90	140	7	CON	EXERCISE2	40	50
	635	37.92	37.40	37.90	137	7	CON	EXERCISE2	40	50
	636	37.92	37.42	37.91	143	7	CON	EXERCISE2	40	50
	637	37.94	37.44	37.91	143	7	CON	EXERCISE2	40	50
	638	37.95	37.35	37.91	146	7	CON	EXERCISE2	40	50
	639	37.95	37.28	37.90	139	7	CON	EXERCISE2	40	50
	640	37.95	37.37	37.91	141	7	CON	EXERCISE2	40	50
	641	37.96	37.44	37.93	140	7	CON	EXERCISE2	40	50
	642	37.96	37.46	37.93	147	7	CON	EXERCISE2	40	50
	643	37.96	37.48	37.93	140	7	CON	EXERCISE2	40	50
	644	37.96	37.48	37.96	141	7	CON	REST3	28	50
	645	37.95	37.45	37.93	135	7	CON	REST3	28	50
	646	37.95	37.46	37.84	122	7	CON	REST3	28	50
	647	37.96	37.44	37.80	120	7	CON	REST3	28	50
	648	37.96	37.40	37.76	116	7	CON	REST3	28	50
	649	37.96	37.44	37.71	119	7	CON	REST3	28	50
	650	37.95	37.45	37.68	128	7	CON	REST3	28	50
	651	37.95	37.41	37.63	124	7	CON	REST3	28	50
	652	37.96	37.35	37.60	117	7	CON	REST3	28	50
	653	37.96	37.33	37.60	117	7	CON	REST3	28	50
	654	37.96	37.38	37.61	114	7	CON	REST3	28	50
	655	37.97	37.33	37.57	117	7	CON	REST3	28	50
	656	37.97	37.34	37.51	113	7	CON	REST3	28	50
	657	37.96	37.43	37.46	107	7	CON	REST3	28	50
	658	37.96	37.46	37.41	108	7	CON	REST3	28	50
	659	37.96	37.45	37.39	106	7	CON	REST3	28	50
	660	37.96	37.45	37.39	108	7	CON	REST3	28	50
110	661	37.96	37.48	37.35	107	7	CON	REST3	28	50
	662	37.96	37.47	37.29	110	7	CON	REST3	28	50
	663	37.96	37.45	37.27	108	7	CON	REST3	28	50
	664	37.95	37.45	37.24	106	7	CON	REST3	28	50
	665	37.95	37.43	37.24	108	7	CON	REST3	28	50
	666	37.96	37.42	37.22	107	7	CON	REST3	28	50
	667	37.95	37.40	37.17	107	7	CON	REST3	28	50
	668	37.95	37.31	37.14	110	7	CON	REST3	28	50

min	number	Tre	Tes	Tsk-head	HR	ID	condition	period	Ta	RH
	669	37.96	37.28	37.12	107	7	CON	REST3	28	50
	670	37.97	37.34	37.11	107	7	CON	REST3	28	50
	671	37.96	37.35	37.10	108	7	CON	REST3	28	50
	672	37.96	37.33	37.08	106	7	CON	REST3	28	50
	673	37.96	37.30	37.06	104	7	CON	REST3	28	50
	674	37.97	37.28	37.04	103	7	CON	REST3	28	50
	675	37.97	37.24	37.02	110	7	CON	REST3	28	50
	676	37.97	37.24	36.99	110	7	CON	REST3	28	50
	677	37.96	37.30	36.98	104	7	CON	REST3	28	50
	678	37.96	37.27	36.97	105	7	CON	REST3	28	50
	679	37.95	37.22	36.96	98	7	CON	REST3	28	50
	680	37.95	37.22	36.98	97	7	CON	REST3	28	50
	681	37.95	37.09	36.98	102	7	CON	REST3	28	50
	682	37.97	36.64	36.94	103	7	CON	REST3	28	50
	683	37.98	36.57	36.90	109	7	CON	REST3	28	50
	684	37.98	36.54	36.88	105	7	CON	REST3	28	50
	685	37.98	36.26	36.84	97	7	CON	REST3	28	50
	686	37.99	36.10	36.83	101	7	CON	REST3	28	50
	687	38.00	36.13	36.84	102	7	CON	REST3	28	50
	688	38.00	36.08	36.84	101	7	CON	REST3	28	50
	689	38.00	35.91	36.85	107	7	CON	REST3	28	50
	690	37.99	35.79	36.84	100	7	CON	REST3	28	50
115	691	37.99	35.69	36.82	105	7	CON	REST3	28	50
	692	38.00	36.00	36.79	101	7	CON	REST3	28	50
	693	37.99	36.58	36.75	105	7	CON	REST3	28	50
	694	37.99	37.02	36.75	106	7	CON	REST3	28	50
	695	37.99	37.12	36.75	101	7	CON	REST3	28	50
	696	37.99	37.13	36.74	101	7	CON	REST3	28	50
	697	37.99	37.12	36.71	100	7	CON	REST3	28	50
	698	37.98	37.08	36.70	87	7	CON	REST3	28	50
	699	37.97	37.02	36.72	90	7	CON	REST3	28	50
	700	37.97	36.99	36.73	96	7	CON	REST3	28	50
	701	37.96	37.02	36.69	101	7	CON	REST3	28	50
	702	37.95	37.10	36.65	100	7	CON	REST3	28	50
	703	37.94	37.15	36.65	98	7	CON	REST3	28	50
	704	37.94	37.06	36.66	104	7	CON	REST3	28	50
	705	37.94	37.04	36.67	102	7	CON	REST3	28	50
	706	37.93	37.09	36.67	98	7	CON	REST3	28	50
	707	37.93	37.00	36.67	98	7	CON	REST3	28	50
	708	37.93	36.96	36.63	101	7	CON	REST3	28	50
	709	37.91	37.05	36.60	92	7	CON	REST3	28	50
0	1	37.18	37.25	35.87	76	8	CON	REST1	28	50
	2	37.19	37.24	35.88	74	8	CON	REST1	28	50
	3	37.19	37.25	35.88	74	8	CON	REST1	28	50
	4	37.19	37.25	35.87	75	8	CON	REST1	28	50
	5	37.19	37.26	35.87	75	8	CON	REST1	28	50

min	number	Tre	Tes	Tsk-head	HR	ID	condition	period	Ta	RH
	6	37.19	37.23	35.87	78	8	CON	REST1	28	50
	7	37.19	37.20	35.89	74	8	CON	REST1	28	50
	8	37.19	37.20	35.90	73	8	CON	REST1	28	50
	9	37.18	37.20	35.91	72	8	CON	REST1	28	50
	10	37.18	37.24	35.90	71	8	CON	REST1	28	50
	11	37.19	37.26	35.91	68	8	CON	REST1	28	50
	12	37.20	37.26	35.92	65	8	CON	REST1	28	50
	13	37.20	37.27	35.91	70	8	CON	REST1	28	50
	14	37.19	37.27	35.90	71	8	CON	REST1	28	50
	15	37.19	37.26	35.90	69	8	CON	REST1	28	50
	16	37.19	37.22	35.90	70	8	CON	REST1	28	50
	17	37.19	37.19	35.89	74	8	CON	REST1	28	50
	18	37.19	37.18	35.88	73	8	CON	REST1	28	50
	19	37.20	37.19	35.88	72	8	CON	REST1	28	50
	20	37.20	37.20	35.88	74	8	CON	REST1	28	50
	21	37.20	37.19	35.88	73	8	CON	REST1	28	50
	22	37.20	37.20	35.88	74	8	CON	REST1	28	50
	23	37.20	37.20	35.88	75	8	CON	REST1	28	50
	24	37.21	37.20	35.89	75	8	CON	REST1	28	50
	25	37.21	37.18	35.88	78	8	CON	REST1	28	50
	26	37.21	37.16	35.88	70	8	CON	REST1	28	50
	27	37.21	37.19	35.87	68	8	CON	REST1	28	50
	28	37.21	37.22	35.86	72	8	CON	REST1	28	50
	29	37.22	37.22	35.86	78	8	CON	REST1	28	50
	30	37.22	37.20	35.86	78	8	CON	REST1	28	50
5	31	37.22	37.21	35.86	74	8	CON	REST1	28	50
	32	37.22	37.24	35.88	72	8	CON	REST1	28	50
	33	37.22	37.25	35.88	82	8	CON	REST1	28	50
	34	37.22	37.24	35.89	72	8	CON	REST1	28	50
	35	37.23	37.24	35.90	75	8	CON	REST1	28	50
	36	37.23	37.26	35.89	75	8	CON	REST1	28	50
	37	37.23	37.25	35.88	74	8	CON	REST1	28	50
	38	37.23	37.23	35.89	82	8	CON	REST1	28	50
	39	37.23	37.23	35.91	77	8	CON	REST1	28	50
	40	37.23	37.24	35.91	76	8	CON	REST1	28	50
	41	37.23	37.25	35.93	69	8	CON	REST1	28	50
	42	37.23	37.26	35.94	73	8	CON	REST1	28	50
	43	37.24	37.30	35.95	75	8	CON	REST1	28	50
	44	37.24	37.31	35.95	77	8	CON	REST1	28	50
	45	37.23	37.31	35.96	85	8	CON	REST1	28	50
	46	37.23	37.34	35.97	81	8	CON	REST1	28	50
	47	37.24	37.32	35.97	74	8	CON	REST1	28	50
	48	37.24	37.28	35.97	75	8	CON	REST1	28	50
	49	37.25	37.26	35.96	74	8	CON	REST1	28	50
	50	37.25	37.25	35.94	74	8	CON	REST1	28	50
	51	37.25	37.23	35.94	75	8	CON	REST1	28	50

min	number	Tre	Tes	Tsk-head	HR	ID	condition	period	Ta	RH
	52	37.25	37.21	35.95	75	8	CON	REST1	28	50
	53	37.25	37.21	35.94	71	8	CON	REST1	28	50
	54	37.24	37.22	35.92	76	8	CON	REST1	28	50
	55	37.24	37.22	35.91	73	8	CON	REST1	28	50
	56	37.24	37.19	35.90	75	8	CON	REST1	28	50
	57	37.24	37.16	35.91	72	8	CON	REST1	28	50
	58	37.24	37.17	35.91	71	8	CON	REST1	28	50
	59	37.24	37.17	35.90	74	8	CON	REST1	28	50
	60	37.23	37.16	35.90	71	8	CON	REST1	28	50
10	61	37.23	37.18	35.89	71	8	CON	REST1	28	50
	62	37.24	37.18	35.89	73	8	CON	REST1	28	50
	63	37.24	37.18	35.90	74	8	CON	REST1	28	50
	64	37.24	37.19	35.90	71	8	CON	REST1	28	50
	65	37.24	37.23	35.90	73	8	CON	REST1	28	50
	66	37.24	37.28	35.90	75	8	CON	REST1	28	50
	67	37.24	37.29	35.90	71	8	CON	REST1	28	50
	68	37.24	37.27	35.91	76	8	CON	REST1	28	50
	69	37.24	37.25	35.93	78	8	CON	REST1	28	50
	70	37.25	37.25	35.94	76	8	CON	REST1	28	50
	71	37.25	37.25	35.95	75	8	CON	REST1	28	50
	72	37.25	37.24	35.94	76	8	CON	REST1	28	50
	73	37.25	37.25	35.93	74	8	CON	REST1	28	50
	74	37.26	37.25	35.93	74	8	CON	REST1	28	50
	75	37.25	37.24	35.92	72	8	CON	REST1	28	50
	76	37.25	37.23	35.91	72	8	CON	REST1	28	50
	77	37.26	37.26	35.91	75	8	CON	REST1	28	50
	78	37.26	37.25	35.92	73	8	CON	REST1	28	50
	79	37.25	37.24	35.92	76	8	CON	REST1	28	50
	80	37.25	37.24	35.91	72	8	CON	REST1	28	50
	81	37.25	37.23	35.91	72	8	CON	REST1	28	50
	82	37.25	37.22	35.91	68	8	CON	REST1	28	50
	83	37.25	37.20	35.92	69	8	CON	REST1	28	50
	84	37.25	37.20	35.91	71	8	CON	REST1	28	50
	85	37.25	37.20	35.91	75	8	CON	REST1	28	50
	86	37.26	37.18	35.91	72	8	CON	REST1	28	50
	87	37.26	37.16	35.91	71	8	CON	REST1	28	50
	88	37.25	37.18	35.91	68	8	CON	REST1	28	50
	89	37.25	37.20	35.91	70	8	CON	REST1	28	50
	90	37.25	37.21	35.91	75	8	CON	REST1	28	50
15	91	37.26	37.21	35.91	75	8	CON	REST1	28	50
	92	37.26	37.19	35.91	74	8	CON	REST1	28	50
	93	37.26	37.19	35.90	70	8	CON	REST1	28	50
	94	37.26	37.19	35.90	71	8	CON	REST1	28	50
	95	37.25	37.20	35.91	73	8	CON	REST1	28	50
	96	37.25	37.21	35.93	84	8	CON	REST1	28	50
	97	37.25	37.19	35.94	82	8	CON	REST1	28	50

min	number	Tre	Tes	Tsk-head	HR	ID	condition	period	Ta	RH
	98	37.25	37.17	35.96	95	8	CON	REST1	28	50
	99	37.24	37.16	35.97	93	8	CON	REST1	28	50
	100	37.24	37.14	35.97	88	8	CON	REST1	28	50
	101	37.24	37.15	35.99	84	8	CON	REST1	28	50
	102	37.25	37.17	36.01	76	8	CON	REST1	28	50
	103	37.25	37.19	35.97	75	8	CON	REST1	40	50
	104	37.25	37.18	35.97	89	8	CON	REST1	40	50
	105	37.25	37.13	36.04	96	8	CON	REST1	40	50
	106	37.25	37.10	36.08	75	8	CON	REST1	40	50
	107	37.25	37.09	36.11	73	8	CON	REST1	40	50
	108	37.25	37.07	36.14	69	8	CON	REST1	40	50
	109	37.25	37.04	36.18	75	8	CON	REST1	40	50
	110	37.25	37.02	36.23	73	8	CON	REST1	40	50
	111	37.24	36.99	36.27	80	8	CON	REST1	40	50
	112	37.23	37.05	36.30	81	8	CON	REST1	40	50
	113	37.23	37.03	36.34	73	8	CON	REST1	40	50
	114	37.23	36.95	36.39	75	8	CON	REST1	40	50
	115	37.23	36.96	36.42	72	8	CON	REST1	40	50
	116	37.22	36.95	36.45	72	8	CON	REST1	40	50
	117	37.22	36.94	36.48	74	8	CON	REST1	40	50
	118	37.22	36.92	36.52	72	8	CON	REST1	40	50
	119	37.22	36.89	36.56	77	8	CON	REST1	40	50
	120	37.21	36.88	36.59	72	8	CON	REST1	40	50
20	121	37.21	36.89	36.61	70	8	CON	REST1	40	50
	122	37.22	36.90	36.62	73	8	CON	REST1	40	50
	123	37.22	36.91	36.63	78	8	CON	REST1	40	50
	124	37.22	36.90	36.65	75	8	CON	REST1	40	50
	125	37.22	36.91	36.66	74	8	CON	REST1	40	50
	126	37.22	36.93	36.68	78	8	CON	REST1	40	50
	127	37.22	36.92	36.69	71	8	CON	REST1	40	50
	128	37.21	36.90	36.71	81	8	CON	REST1	40	50
	129	37.22	36.90	36.74	86	8	CON	REST1	40	50
	130	37.23	36.91	36.75	86	8	CON	REST1	40	50
	131	37.22	36.92	36.77	76	8	CON	REST1	40	50
	132	37.22	36.92	36.79	73	8	CON	REST1	40	50
	133	37.22	36.92	36.80	80	8	CON	REST1	40	50
	134	37.24	36.92	36.80	100	8	CON	REST1	40	50
	135	37.24	36.89	36.82	95	8	CON	REST1	40	50
	136	37.23	36.88	36.82	90	8	CON	REST1	40	50
	137	37.23	36.90	36.84	86	8	CON	REST1	40	50
	138	37.23	36.90	36.86	87	8	CON	REST1	40	50
	139	37.23	36.84	36.86	89	8	CON	EXERCISE1	40	50
	140	37.22	36.81	36.86	89	8	CON	EXERCISE1	40	50
	141	37.22	36.81	36.86	96	8	CON	EXERCISE1	40	50
	142	37.23	36.82	36.87	102	8	CON	EXERCISE1	40	50
	143	37.22	36.79	36.88	102	8	CON	EXERCISE1	40	50

min	number	Tre	Tes	Tsk-head	HR	ID	condition	period	Ta	RH
	144	37.23	36.80	36.87	104	8	CON	EXERCISE1	40	50
	145	37.23	36.82	36.86	104	8	CON	EXERCISE1	40	50
	146	37.23	36.79	36.88	108	8	CON	EXERCISE1	40	50
	147	37.24	36.80	36.88	113	8	CON	EXERCISE1	40	50
	148	37.24	36.82	36.88	115	8	CON	EXERCISE1	40	50
	149	37.23	36.82	36.91	113	8	CON	EXERCISE1	40	50
	150	37.23	36.82	36.92	116	8	CON	EXERCISE1	40	50
25	151	37.23	36.84	36.92	117	8	CON	EXERCISE1	40	50
	152	37.23	36.86	36.95	118	8	CON	EXERCISE1	40	50
	153	37.24	36.86	36.97	117	8	CON	EXERCISE1	40	50
	154	37.24	36.86	36.97	119	8	CON	EXERCISE1	40	50
	155	37.23	36.84	36.97	122	8	CON	EXERCISE1	40	50
	156	37.23	36.87	36.98	121	8	CON	EXERCISE1	40	50
	157	37.24	36.90	36.99	122	8	CON	EXERCISE1	40	50
	158	37.24	36.87	36.99	123	8	CON	EXERCISE1	40	50
	159	37.23	36.88	36.99	122	8	CON	EXERCISE1	40	50
	160	37.24	36.88	37.01	124	8	CON	EXERCISE1	40	50
	161	37.24	36.89	37.01	123	8	CON	EXERCISE1	40	50
	162	37.23	36.90	37.00	124	8	CON	EXERCISE1	40	50
	163	37.23	36.91	37.01	125	8	CON	EXERCISE1	40	50
	164	37.24	36.92	37.01	127	8	CON	EXERCISE1	40	50
	165	37.25	36.91	37.01	124	8	CON	EXERCISE1	40	50
	166	37.25	36.91	37.00	124	8	CON	EXERCISE1	40	50
	167	37.25	36.92	37.01	127	8	CON	EXERCISE1	40	50
	168	37.24	36.92	37.02	126	8	CON	EXERCISE1	40	50
	169	37.25	36.89	37.04	127	8	CON	EXERCISE1	40	50
	170	37.26	36.88	37.04	129	8	CON	EXERCISE1	40	50
	171	37.26	36.93	37.04	130	8	CON	EXERCISE1	40	50
	172	37.27	36.95	37.04	129	8	CON	EXERCISE1	40	50
	173	37.27	36.97	37.04	132	8	CON	EXERCISE1	40	50
	174	37.27	36.97	37.05	131	8	CON	EXERCISE1	40	50
	175	37.27	36.97	37.04	133	8	CON	EXERCISE1	40	50
	176	37.28	37.00	37.05	132	8	CON	EXERCISE1	40	50
	177	37.29	37.01	37.07	131	8	CON	EXERCISE1	40	50
	178	37.28	36.98	37.06	134	8	CON	EXERCISE1	40	50
	179	37.28	36.97	37.07	134	8	CON	EXERCISE1	40	50
	180	37.29	36.98	37.05	136	8	CON	EXERCISE1	40	50
30	181	37.29	36.99	37.05	137	8	CON	EXERCISE1	40	50
	182	37.29	37.00	37.09	135	8	CON	EXERCISE1	40	50
	183	37.29	37.00	37.08	136	8	CON	EXERCISE1	40	50
	184	37.30	36.99	37.08	138	8	CON	EXERCISE1	40	50
	185	37.31	37.02	37.09	137	8	CON	EXERCISE1	40	50
	186	37.31	37.03	37.09	137	8	CON	EXERCISE1	40	50
	187	37.31	37.06	37.08	136	8	CON	EXERCISE1	40	50
	188	37.31	37.06	37.07	140	8	CON	EXERCISE1	40	50
	189	37.31	37.04	37.09	140	8	CON	EXERCISE1	40	50

min	number	Tre	Tes	Tsk-head	HR	ID	condition	period	Ta	RH
	190	37.32	36.98	37.11	141	8	CON	EXERCISE1	40	50
	191	37.32	36.99	37.11	137	8	CON	EXERCISE1	40	50
	192	37.32	37.08	37.11	139	8	CON	EXERCISE1	40	50
	193	37.32	37.09	37.11	140	8	CON	EXERCISE1	40	50
	194	37.33	37.08	37.10	140	8	CON	EXERCISE1	40	50
	195	37.33	37.09	37.09	140	8	CON	EXERCISE1	40	50
	196	37.33	37.12	37.10	140	8	CON	EXERCISE1	40	50
	197	37.34	37.09	37.12	143	8	CON	EXERCISE1	40	50
	198	37.33	37.08	37.11	140	8	CON	EXERCISE1	40	50
	199	37.33	37.14	37.12	143	8	CON	EXERCISE1	40	50
	200	37.34	37.17	37.14	142	8	CON	EXERCISE1	40	50
	201	37.34	37.17	37.13	140	8	CON	EXERCISE1	40	50
	202	37.34	37.20	37.14	142	8	CON	EXERCISE1	40	50
	203	37.34	37.23	37.15	142	8	CON	EXERCISE1	40	50
	204	37.35	37.21	37.16	142	8	CON	EXERCISE1	40	50
	205	37.35	37.20	37.14	143	8	CON	EXERCISE1	40	50
	206	37.35	37.26	37.12	146	8	CON	EXERCISE1	40	50
	207	37.36	37.19	37.14	146	8	CON	EXERCISE1	40	50
	208	37.36	37.17	37.13	143	8	CON	EXERCISE1	40	50
	209	37.36	37.24	37.15	146	8	CON	EXERCISE1	40	50
	210	37.37	37.22	37.20	144	8	CON	EXERCISE1	40	50
35	211	37.37	37.22	37.23	143	8	CON	EXERCISE1	40	50
	212	37.37	37.25	37.22	145	8	CON	EXERCISE1	40	50
	213	37.38	37.23	37.22	145	8	CON	EXERCISE1	40	50
	214	37.39	37.24	37.24	143	8	CON	EXERCISE1	40	50
	215	37.39	37.30	37.23	145	8	CON	EXERCISE1	40	50
	216	37.38	37.32	37.23	146	8	CON	EXERCISE1	40	50
	217	37.38	37.26	37.21	147	8	CON	EXERCISE1	40	50
	218	37.39	37.25	37.18	147	8	CON	EXERCISE1	40	50
	219	37.39	37.30	37.21	147	8	CON	EXERCISE1	40	50
	220	37.40	37.24	37.23	149	8	CON	EXERCISE1	40	50
	221	37.40	37.23	37.22	146	8	CON	EXERCISE1	40	50
	222	37.41	37.33	37.21	146	8	CON	EXERCISE1	40	50
	223	37.42	37.37	37.23	147	8	CON	EXERCISE1	40	50
	224	37.42	37.35	37.29	146	8	CON	EXERCISE1	40	50
	225	37.41	37.36	37.31	146	8	CON	EXERCISE1	40	50
	226	37.41	37.40	37.33	146	8	CON	EXERCISE1	40	50
	227	37.42	37.38	37.36	146	8	CON	EXERCISE1	40	50
	228	37.42	37.37	37.33	150	8	CON	EXERCISE1	40	50
	229	37.42	37.40	37.29	150	8	CON	EXERCISE1	40	50
	230	37.43	37.29	37.31	150	8	CON	EXERCISE1	40	50
	231	37.44	37.26	37.34	150	8	CON	EXERCISE1	40	50
	232	37.44	37.38	37.35	148	8	CON	EXERCISE1	40	50
	233	37.43	37.39	37.35	147	8	CON	EXERCISE1	40	50
	234	37.44	37.46	37.37	146	8	CON	EXERCISE1	40	50
	235	37.45	37.51	37.38	150	8	CON	EXERCISE1	40	50

min	number	Tre	Tes	Tsk-head	HR	ID	condition	period	Ta	RH
	236	37.45	37.49	37.37	152	8	CON	EXERCISE1	40	50
	237	37.45	37.40	37.35	153	8	CON	EXERCISE1	40	50
	238	37.45	37.38	37.32	151	8	CON	EXERCISE1	40	50
	239	37.46	37.48	37.31	146	8	CON	EXERCISE1	40	50
	240	37.47	37.52	37.32	149	8	CON	EXERCISE1	40	50
40	241	37.47	37.54	37.33	151	8	CON	EXERCISE1	40	50
	242	37.48	37.56	37.34	150	8	CON	EXERCISE1	40	50
	243	37.48	37.59	37.39	146	8	CON	EXERCISE1	40	50
	244	37.49	37.60	37.41	150	8	CON	EXERCISE1	40	50
	245	37.49	37.57	37.41	150	8	CON	EXERCISE1	40	50
	246	37.49	37.57	37.44	150	8	CON	EXERCISE1	40	50
	247	37.49	37.58	37.46	152	8	CON	EXERCISE1	40	50
	248	37.50	37.56	37.46	150	8	CON	EXERCISE1	40	50
	249	37.50	37.55	37.48	151	8	CON	EXERCISE1	40	50
	250	37.50	37.60	37.46	153	8	CON	EXERCISE1	40	50
	251	37.51	37.64	37.44	155	8	CON	EXERCISE1	40	50
	252	37.52	37.57	37.45	157	8	CON	EXERCISE1	40	50
	253	37.52	37.56	37.46	157	8	CON	EXERCISE1	40	50
	254	37.52	37.59	37.47	156	8	CON	EXERCISE1	40	50
	255	37.52	37.62	37.49	155	8	CON	EXERCISE1	40	50
	256	37.53	37.67	37.47	157	8	CON	EXERCISE1	40	50
	257	37.54	37.67	37.43	157	8	CON	EXERCISE1	40	50
	258	37.55	37.70	37.43	155	8	CON	EXERCISE1	40	50
	259	37.55	37.72	37.42	154	8	CON	EXERCISE1	40	50
	260	37.55	37.70	37.43	157	8	CON	EXERCISE1	40	50
	261	37.55	37.70	37.47	157	8	CON	EXERCISE1	40	50
	262	37.56	37.66	37.47	157	8	CON	EXERCISE1	40	50
	263	37.57	37.66	37.46	158	8	CON	EXERCISE1	40	50
	264	37.58	37.74	37.48	157	8	CON	EXERCISE1	40	50
	265	37.58	37.78	37.50	157	8	CON	EXERCISE1	40	50
	266	37.58	37.74	37.50	159	8	CON	EXERCISE1	40	50
	267	37.59	37.73	37.46	159	8	CON	EXERCISE1	40	50
	268	37.59	37.79	37.44	160	8	CON	EXERCISE1	40	50
	269	37.59	37.74	37.44	160	8	CON	EXERCISE1	40	50
	270	37.60	37.74	37.47	159	8	CON	EXERCISE1	40	50
45	271	37.60	37.78	37.50	158	8	CON	EXERCISE1	40	50
	272	37.60	37.80	37.52	157	8	CON	EXERCISE1	40	50
	273	37.60	37.83	37.52	160	8	CON	EXERCISE1	40	50
	274	37.61	37.84	37.51	161	8	CON	EXERCISE1	40	50
	275	37.62	37.82	37.48	161	8	CON	EXERCISE1	40	50
	276	37.63	37.83	37.47	161	8	CON	EXERCISE1	40	50
	277	37.63	37.88	37.53	161	8	CON	EXERCISE1	40	50
	278	37.64	37.90	37.58	161	8	CON	EXERCISE1	40	50
	279	37.65	37.82	37.60	162	8	CON	EXERCISE1	40	50
	280	37.65	37.80	37.61	161	8	CON	EXERCISE1	40	50
	281	37.66	37.86	37.63	161	8	CON	EXERCISE1	40	50

min	number	Tre	Tes	Tsk-head	HR	ID	condition	period	Ta	RH
	282	37.67	37.85	37.65	161	8	CON	EXERCISE1	40	50
	283	37.67	37.85	37.64	159	8	CON	EXERCISE1	40	50
	284	37.67	37.89	37.61	160	8	CON	EXERCISE1	40	50
	285	37.67	37.85	37.59	162	8	CON	EXERCISE1	40	50
	286	37.68	37.85	37.62	163	8	CON	EXERCISE1	40	50
	287	37.68	37.91	37.64	164	8	CON	EXERCISE1	40	50
	288	37.69	37.90	37.62	165	8	CON	EXERCISE1	40	50
	289	37.70	37.90	37.63	164	8	CON	EXERCISE1	40	50
	290	37.70	37.90	37.65	163	8	CON	EXERCISE1	40	50
	291	37.70	37.82	37.67	166	8	CON	EXERCISE1	40	50
	292	37.70	37.85	37.68	163	8	CON	EXERCISE1	40	50
	293	37.72	37.94	37.68	162	8	CON	EXERCISE1	40	50
	294	37.73	37.86	37.70	165	8	CON	EXERCISE1	40	50
	295	37.73	37.83	37.73	162	8	CON	EXERCISE1	40	50
	296	37.73	37.94	37.72	163	8	CON	EXERCISE1	40	50
	297	37.73	37.99	37.70	165	8	CON	EXERCISE1	40	50
	298	37.73	37.99	37.72	166	8	CON	EXERCISE1	40	50
	299	37.74	38.00	37.74	166	8	CON	EXERCISE1	40	50
	300	37.75	38.04	37.73	166	8	CON	EXERCISE1	40	50
50	301	37.75	38.06	37.74	166	8	CON	EXERCISE1	40	50
	302	37.76	38.07	37.80	166	8	CON	EXERCISE1	40	50
	303	37.76	38.02	37.81	166	8	CON	EXERCISE1	40	50
	304	37.78	37.99	37.78	166	8	CON	EXERCISE1	40	50
	305	37.78	38.04	37.75	166	8	CON	EXERCISE1	40	50
	306	37.78	38.04	37.73	166	8	CON	EXERCISE1	40	50
	307	37.79	38.06	37.73	166	8	CON	EXERCISE1	40	50
	308	37.80	38.08	37.76	166	8	CON	EXERCISE1	40	50
	309	37.80	38.09	37.79	168	8	CON	EXERCISE1	40	50
	310	37.81	38.09	37.81	166	8	CON	EXERCISE1	40	50
	311	37.81	38.10	37.80	167	8	CON	EXERCISE1	40	50
	312	37.82	38.15	37.80	167	8	CON	EXERCISE1	40	50
	313	37.83	38.10	37.83	170	8	CON	EXERCISE1	40	50
	314	37.84	38.02	37.86	166	8	CON	EXERCISE1	40	50
	315	37.84	38.05	37.93	166	8	CON	EXERCISE1	40	50
	316	37.84	38.06	37.93	167	8	CON	EXERCISE1	40	50
	317	37.85	38.07	37.90	168	8	CON	EXERCISE1	40	50
	318	37.85	37.94	37.91	170	8	CON	EXERCISE1	40	50
	319	37.86	37.91	37.94	167	8	CON	EXERCISE1	40	50
	320	37.87	38.06	37.95	166	8	CON	REST2	28	50
	321	37.87	38.10	37.95	165	8	CON	REST2	28	50
	322	37.87	38.09	37.97	166	8	CON	REST2	28	50
	323	37.87	38.10	37.97	161	8	CON	REST2	28	50
	324	37.89	38.11	38.00	161	8	CON	REST2	28	50
	325	37.89	38.11	38.00	157	8	CON	REST2	28	50
	326	37.89	38.16	37.92	159	8	CON	REST2	28	50
	327	37.90	38.17	37.86	157	8	CON	REST2	28	50

min	number	Tre	Tes	Tsk-head	HR	ID	condition	period	Ta	RH
	328	37.91	38.17	37.77	158	8	CON	REST2	28	50
	329	37.91	38.02	37.65	158	8	CON	REST2	28	50
	330	37.91	38.02	37.54	144	8	CON	REST2	28	50
55	331	37.93	38.22	37.44	135	8	CON	REST2	28	50
	332	37.94	38.23	37.39	144	8	CON	REST2	28	50
	333	37.94	38.24	37.37	148	8	CON	REST2	28	50
	334	37.94	38.29	37.38	145	8	CON	REST2	28	50
	335	37.93	38.29	37.40	136	8	CON	REST2	28	50
	336	37.94	38.28	37.28	137	8	CON	REST2	28	50
	337	37.94	33.01	37.17	145	8	CON	REST2	28	50
	338	37.95	30.43	37.11	145	8	CON	REST2	28	50
	339	37.96	31.35	37.04	143	8	CON	REST2	28	50
	340	37.96	30.76	36.99	140	8	CON	REST2	28	50
	341	37.97	32.48	36.92	133	8	CON	REST2	28	50
	342	37.98	33.36	36.93	130	8	CON	REST2	28	50
	343	37.97	34.16	36.90	128	8	CON	REST2	28	50
	344	37.98	35.15	36.82	123	8	CON	REST2	28	50
	345	37.99	33.61	36.85	134	8	CON	REST2	28	50
	346	38.00	32.34	36.90	132	8	CON	REST2	28	50
	347	38.00	33.50	36.87	126	8	CON	REST2	28	50
	348	38.00	34.17	36.82	126	8	CON	REST2	28	50
	349	38.01	34.75	36.82	123	8	CON	REST2	28	50
	350	38.02	35.34	36.83	121	8	CON	REST2	28	50
	351	38.02	35.88	36.78	124	8	CON	REST2	28	50
	352	38.02	36.30	36.73	122	8	CON	REST2	28	50
	353	38.03	34.68	36.73	124	8	CON	REST2	28	50
	354	38.03	33.64	36.72	124	8	CON	REST2	28	50
	355	38.03	34.72	36.68	117	8	CON	REST2	28	50
	356	38.03	35.25	36.59	120	8	CON	REST2	28	50
	357	38.04	35.55	36.58	117	8	CON	REST2	28	50
	358	38.04	35.53	36.56	114	8	CON	REST2	28	50
	359	38.03	35.42	36.54	119	8	CON	REST2	28	50
	360	38.03	35.39	36.58	116	8	CON	REST2	28	50
60	361	38.04	35.79	36.58	122	8	CON	REST2	28	50
	362	38.04	33.28	36.55	122	8	CON	REST2	28	50
	363	38.04	31.45	36.58	117	8	CON	REST2	28	50
	364	38.05	33.17	36.58	115	8	CON	REST2	28	50
	365	38.04	34.18	36.48	113	8	CON	REST2	28	50
	366	38.05	34.74	36.47	113	8	CON	REST2	28	50
	367	38.06	31.74	36.51	123	8	CON	REST2	28	50
	368	38.06	29.67	36.49	119	8	CON	REST2	28	50
	369	38.06	31.55	36.44	112	8	CON	REST2	28	50
	370	38.06	32.70	36.37	113	8	CON	REST2	28	50
	371	38.06	33.36	36.38	110	8	CON	REST2	28	50
	372	38.05	33.93	36.38	108	8	CON	REST2	28	50
	373	38.06	31.75	36.38	113	8	CON	REST2	28	50

min	number	Tre	Tes	Tsk-head	HR	ID	condition	period	Ta	RH
	374	38.07	29.71	36.37	116	8	CON	REST2	28	50
	375	38.06	31.84	36.29	112	8	CON	REST2	28	50
	376	38.06	33.87	36.27	113	8	CON	REST2	28	50
	377	38.07	31.66	36.30	117	8	CON	REST2	28	50
	378	38.07	30.36	36.30	113	8	CON	REST2	28	50
	379	38.08	31.99	36.30	110	8	CON	REST2	28	50
	380	38.08	32.52	36.30	108	8	CON	REST2	28	50
	381	38.08	32.20	36.30	117	8	CON	REST2	28	50
	382	38.08	32.71	36.32	111	8	CON	REST2	28	50
	383	38.07	34.15	36.33	107	8	CON	REST2	28	50
	384	38.07	33.71	36.32	107	8	CON	REST2	28	50
	385	38.07	30.77	36.34	107	8	CON	REST2	28	50
	386	38.07	29.76	36.30	112	8	CON	REST2	28	50
	387	38.08	31.36	36.26	108	8	CON	REST2	28	50
	388	38.07	32.40	36.24	103	8	CON	REST2	28	50
	389	38.05	33.26	36.23	101	8	CON	REST2	28	50
	390	38.06	33.66	36.24	103	8	CON	REST2	28	50
65	391	38.06	30.86	36.21	109	8	CON	REST2	28	50
	392	38.06	30.15	36.20	107	8	CON	REST2	28	50
	393	38.05	32.81	36.17	107	8	CON	REST2	28	50
	394	38.05	33.63	36.19	100	8	CON	REST2	28	50
	395	38.05	34.21	36.21	103	8	CON	REST2	28	50
	396	38.05	34.85	36.18	99	8	CON	REST2	28	50
	397	38.05	35.32	36.20	95	8	CON	REST2	28	50
	398	38.06	35.72	36.21	102	8	CON	REST2	28	50
	399	38.06	36.02	36.16	101	8	CON	REST2	28	50
	400	38.06	36.14	36.15	96	8	CON	REST2	28	50
	401	38.05	36.27	36.19	96	8	CON	REST2	28	50
	402	38.05	36.42	36.13	97	8	CON	REST2	28	50
	403	38.05	36.51	36.02	100	8	CON	REST2	28	50
	404	38.05	36.50	35.98	100	8	CON	REST2	28	50
	405	38.05	36.24	35.99	101	8	CON	REST2	28	50
	406	38.05	36.38	35.98	100	8	CON	REST2	28	50
	407	38.05	36.78	36.00	96	8	CON	REST2	28	50
	408	38.04	36.84	35.98	97	8	CON	REST2	28	50
	409	38.04	36.88	35.98	96	8	CON	REST2	28	50
	410	38.02	36.90	36.02	91	8	CON	REST2	28	50
	411	38.02	36.95	35.99	92	8	CON	REST2	28	50
	412	38.02	36.98	35.99	89	8	CON	REST2	28	50
	413	38.02	37.01	35.96	92	8	CON	REST2	28	50
	414	38.01	37.04	35.94	94	8	CON	REST2	28	50
	415	38.01	37.05	35.89	98	8	CON	REST2	28	50
	416	38.01	37.10	35.83	95	8	CON	REST2	28	50
	417	38.01	37.10	35.81	94	8	CON	REST2	28	50
	418	38.00	37.08	35.81	99	8	CON	REST2	28	50
	419	38.00	37.11	35.85	92	8	CON	REST2	28	50

min	number	Tre	Tes	Tsk-head	HR	ID	condition	period	Ta	RH
	420	37.99	37.12	35.87	90	8	CON	REST2	28	50
70	421	37.99	37.13	35.84	92	8	CON	REST2	28	50
	422	37.99	37.12	35.80	90	8	CON	REST2	28	50
	423	37.99	37.15	35.81	91	8	CON	REST2	28	50
	424	37.99	37.18	35.81	92	8	CON	REST2	28	50
	425	37.97	37.14	35.74	93	8	CON	REST2	28	50
	426	37.96	37.12	35.68	95	8	CON	REST2	28	50
	427	37.96	37.12	35.68	93	8	CON	REST2	28	50
	428	37.96	37.16	35.59	90	8	CON	REST2	28	50
	429	37.95	37.21	35.47	88	8	CON	REST2	28	50
	430	37.95	37.18	35.43	91	8	CON	REST2	28	50
	431	37.94	36.94	35.42	87	8	CON	REST2	28	50
	432	37.94	36.81	35.52	88	8	CON	REST2	28	50
	433	37.94	36.91	35.65	91	8	CON	REST2	28	50
	434	37.94	36.96	35.74	86	8	CON	REST2	28	50
	435	37.94	37.01	35.82	89	8	CON	REST2	28	50
	436	37.94	37.17	35.83	96	8	CON	REST2	28	50
	437	37.93	37.31	35.86	87	8	CON	REST2	28	50
	438	37.92	37.27	35.86	90	8	CON	REST2	28	50
	439	37.92	37.17	35.81	106	8	CON	REST2	40	50
	440	37.91	37.10	35.80	98	8	CON	REST2	40	50
	441	37.91	37.08	35.79	100	8	CON	REST2	40	50
	442	37.90	37.06	35.87	114	8	CON	REST2	40	50
	443	37.90	37.05	36.09	113	8	CON	REST2	40	50
	444	37.90	37.09	36.27	112	8	CON	REST2	40	50
	445	37.90	37.09	36.42	106	8	CON	REST2	40	50
	446	37.90	37.13	36.53	96	8	CON	REST2	40	50
	447	37.90	37.16	36.60	96	8	CON	REST2	40	50
	448	37.89	37.16	36.63	91	8	CON	REST2	40	50
	449	37.89	37.15	36.63	93	8	CON	REST2	40	50
	450	37.89	37.13	36.64	98	8	CON	REST2	40	50
75	451	37.88	37.08	36.64	97	8	CON	REST2	40	50
	452	37.88	37.02	36.64	98	8	CON	REST2	40	50
	453	37.88	37.03	36.65	101	8	CON	REST2	40	50
	454	37.87	37.04	36.64	97	8	CON	REST2	40	50
	455	37.86	37.03	36.65	100	8	CON	REST2	40	50
	456	37.86	37.00	36.68	99	8	CON	REST2	40	50
	457	37.87	36.98	36.67	102	8	CON	REST2	40	50
	458	37.86	36.96	36.63	114	8	CON	REST2	40	50
	459	37.86	36.92	36.62	113	8	CON	REST2	40	50
	460	37.86	36.91	36.66	102	8	CON	REST2	40	50
	461	37.86	36.92	36.68	104	8	CON	REST2	40	50
	462	37.86	36.92	36.70	105	8	CON	REST2	40	50
	463	37.85	36.92	36.73	107	8	CON	EXERCISE2	40	50
	464	37.85	36.90	36.73	106	8	CON	EXERCISE2	40	50
	465	37.85	36.91	36.72	113	8	CON	EXERCISE2	40	50

min	number	Tre	Tes	Tsk-head	HR	ID	condition	period	Ta	RH
	466	37.85	36.90	36.73	122	8	CON	EXERCISE2	40	50
	467	37.85	36.88	36.78	127	8	CON	EXERCISE2	40	50
	468	37.84	36.88	36.81	128	8	CON	EXERCISE2	40	50
	469	37.83	36.89	36.82	129	8	CON	EXERCISE2	40	50
	470	37.83	36.77	36.83	133	8	CON	EXERCISE2	40	50
	471	37.83	36.77	36.83	140	8	CON	EXERCISE2	40	50
	472	37.83	36.94	36.81	140	8	CON	EXERCISE2	40	50
	473	37.84	36.99	36.80	142	8	CON	EXERCISE2	40	50
	474	37.84	37.03	36.82	144	8	CON	EXERCISE2	40	50
	475	37.83	37.02	36.84	140	8	CON	EXERCISE2	40	50
	476	37.83	36.99	36.85	137	8	CON	EXERCISE2	40	50
	477	37.84	37.03	36.87	140	8	CON	EXERCISE2	40	50
	478	37.84	36.97	36.86	145	8	CON	EXERCISE2	40	50
	479	37.83	36.95	36.86	146	8	CON	EXERCISE2	40	50
	480	37.83	37.05	36.84	146	8	CON	EXERCISE2	40	50
80	481	37.83	37.09	36.86	147	8	CON	EXERCISE2	40	50
	482	37.83	37.13	36.90	146	8	CON	EXERCISE2	40	50
	483	37.83	37.17	36.92	143	8	CON	EXERCISE2	40	50
	484	37.84	37.19	36.96	145	8	CON	EXERCISE2	40	50
	485	37.84	37.12	36.98	150	8	CON	EXERCISE2	40	50
	486	37.84	37.09	36.98	150	8	CON	EXERCISE2	40	50
	487	37.84	37.16	36.99	149	8	CON	EXERCISE2	40	50
	488	37.84	37.20	36.99	150	8	CON	EXERCISE2	40	50
	489	37.84	37.19	36.99	151	8	CON	EXERCISE2	40	50
	490	37.85	37.20	37.01	150	8	CON	EXERCISE2	40	50
	491	37.84	37.16	37.02	154	8	CON	EXERCISE2	40	50
	492	37.84	37.16	37.02	154	8	CON	EXERCISE2	40	50
	493	37.84	37.24	37.04	151	8	CON	EXERCISE2	40	50
	494	37.84	37.28	37.04	151	8	CON	EXERCISE2	40	50
	495	37.84	37.31	37.04	150	8	CON	EXERCISE2	40	50
	496	37.84	37.25	37.06	156	8	CON	EXERCISE2	40	50
	497	37.84	37.25	37.07	155	8	CON	EXERCISE2	40	50
	498	37.84	37.32	37.10	153	8	CON	EXERCISE2	40	50
	499	37.84	37.34	37.12	153	8	CON	EXERCISE2	40	50
	500	37.84	37.36	37.13	155	8	CON	EXERCISE2	40	50
	501	37.84	37.39	37.10	156	8	CON	EXERCISE2	40	50
	502	37.85	37.42	37.11	158	8	CON	EXERCISE2	40	50
	503	37.85	37.29	37.14	158	8	CON	EXERCISE2	40	50
	504	37.84	37.22	37.13	158	8	CON	EXERCISE2	40	50
	505	37.84	37.26	37.14	159	8	CON	EXERCISE2	40	50
	506	37.84	37.28	37.16	160	8	CON	EXERCISE2	40	50
	507	37.84	37.36	37.18	159	8	CON	EXERCISE2	40	50
	508	37.83	37.39	37.18	158	8	CON	EXERCISE2	40	50
	509	37.84	37.31	37.19	159	8	CON	EXERCISE2	40	50
	510	37.83	37.29	37.19	158	8	CON	EXERCISE2	40	50
85	511	37.84	37.41	37.20	158	8	CON	EXERCISE2	40	50

min	number	Tre	Tes	Tsk-head	HR	ID	condition	period	Ta	RH
	512	37.85	37.45	37.22	159	8	CON	EXERCISE2	40	50
	513	37.85	37.46	37.24	160	8	CON	EXERCISE2	40	50
	514	37.86	37.49	37.23	160	8	CON	EXERCISE2	40	50
	515	37.85	37.40	37.23	161	8	CON	EXERCISE2	40	50
	516	37.85	37.38	37.23	160	8	CON	EXERCISE2	40	50
	517	37.85	37.50	37.24	160	8	CON	EXERCISE2	40	50
	518	37.85	37.62	37.27	159	8	CON	EXERCISE2	40	50
	519	37.86	37.70	37.29	158	8	CON	EXERCISE2	40	50
	520	37.86	37.70	37.29	160	8	CON	EXERCISE2	40	50
	521	37.86	37.65	37.31	161	8	CON	EXERCISE2	40	50
	522	37.86	37.64	37.32	161	8	CON	EXERCISE2	40	50
	523	37.86	37.69	37.33	163	8	CON	EXERCISE2	40	50
	524	37.85	37.72	37.33	163	8	CON	EXERCISE2	40	50
	525	37.86	37.75	37.33	163	8	CON	EXERCISE2	40	50
	526	37.87	37.75	37.36	162	8	CON	EXERCISE2	40	50
	527	37.87	37.65	37.39	165	8	CON	EXERCISE2	40	50
	528	37.87	37.62	37.41	163	8	CON	EXERCISE2	40	50
	529	37.88	37.72	37.41	164	8	CON	EXERCISE2	40	50
	530	37.88	37.64	37.40	164	8	CON	EXERCISE2	40	50
	531	37.87	37.63	37.41	162	8	CON	EXERCISE2	40	50
	532	37.87	37.66	37.41	165	8	CON	EXERCISE2	40	50
	533	37.87	37.66	37.42	165	8	CON	EXERCISE2	40	50
	534	37.88	37.73	37.42	165	8	CON	EXERCISE2	40	50
	535	37.88	37.75	37.42	165	8	CON	EXERCISE2	40	50
	536	37.88	37.72	37.44	163	8	CON	EXERCISE2	40	50
	537	37.88	37.71	37.44	164	8	CON	EXERCISE2	40	50
	538	37.89	37.76	37.45	165	8	CON	EXERCISE2	40	50
	539	37.89	37.73	37.46	167	8	CON	EXERCISE2	40	50
	540	37.90	37.69	37.48	165	8	CON	EXERCISE2	40	50
90	541	37.90	37.74	37.47	167	8	CON	EXERCISE2	40	50
	542	37.90	37.78	37.48	169	8	CON	EXERCISE2	40	50
	543	37.90	37.81	37.50	168	8	CON	EXERCISE2	40	50
	544	37.91	37.83	37.51	169	8	CON	EXERCISE2	40	50
	545	37.91	37.83	37.52	170	8	CON	EXERCISE2	40	50
	546	37.91	37.81	37.50	169	8	CON	EXERCISE2	40	50
	547	37.92	37.82	37.52	169	8	CON	EXERCISE2	40	50
	548	37.93	37.86	37.53	169	8	CON	EXERCISE2	40	50
	549	37.93	37.87	37.55	169	8	CON	EXERCISE2	40	50
	550	37.93	37.88	37.57	168	8	CON	EXERCISE2	40	50
	551	37.94	37.89	37.57	167	8	CON	EXERCISE2	40	50
	552	37.94	37.86	37.59	171	8	CON	EXERCISE2	40	50
	553	37.94	37.86	37.61	170	8	CON	EXERCISE2	40	50
	554	37.93	37.89	37.61	170	8	CON	EXERCISE2	40	50
	555	37.93	37.91	37.59	170	8	CON	EXERCISE2	40	50
	556	37.94	37.87	37.59	171	8	CON	EXERCISE2	40	50
	557	37.95	37.85	37.61	170	8	CON	EXERCISE2	40	50

min	number	Tre	Tes	Tsk-head	HR	ID	condition	period	Ta	RH
	558	37.94	37.91	37.62	169	8	CON	EXERCISE2	40	50
	559	37.94	37.95	37.62	171	8	CON	EXERCISE2	40	50
	560	37.95	37.97	37.63	170	8	CON	EXERCISE2	40	50
	561	37.95	37.92	37.63	172	8	CON	EXERCISE2	40	50
	562	37.95	37.91	37.63	171	8	CON	EXERCISE2	40	50
	563	37.96	37.95	37.66	173	8	CON	EXERCISE2	40	50
	564	37.96	37.93	37.69	174	8	CON	EXERCISE2	40	50
	565	37.96	37.95	37.67	172	8	CON	EXERCISE2	40	50
	566	37.97	37.99	37.69	172	8	CON	EXERCISE2	40	50
	567	37.98	38.01	37.73	172	8	CON	EXERCISE2	40	50
	568	37.98	37.99	37.74	171	8	CON	EXERCISE2	40	50
	569	37.98	37.98	37.73	170	8	CON	EXERCISE2	40	50
	570	37.99	38.00	37.73	171	8	CON	EXERCISE2	40	50
95	571	38.00	38.02	37.77	175	8	CON	EXERCISE2	40	50
	572	37.99	37.97	37.78	173	8	CON	EXERCISE2	40	50
	573	37.99	37.97	37.77	173	8	CON	EXERCISE2	40	50
	574	38.00	38.05	37.78	155	8	CON	EXERCISE2	40	50
	575	38.01	38.02	37.79	173	8	CON	EXERCISE2	40	50
	576	38.00	38.03	37.77	173	8	CON	EXERCISE2	40	50
	577	38.01	38.06	37.76	175	8	CON	EXERCISE2	40	50
	578	38.02	38.06	37.88	175	8	CON	EXERCISE2	40	50
	579	38.05	38.11	37.90	175	8	CON	EXERCISE2	40	50
	580	38.05	38.14	37.81	173	8	CON	EXERCISE2	40	50
	581	38.03	38.09	37.81	174	8	CON	EXERCISE2	40	50
	582	38.03	38.07	37.80	172	8	CON	EXERCISE2	40	50
	583	38.03	38.15	37.79	173	8	CON	EXERCISE2	40	50
	584	38.05	38.15	37.82	175	8	CON	EXERCISE2	40	50
	585	38.05	38.14	37.84	173	8	CON	EXERCISE2	40	50
	586	38.06	38.19	37.83	171	8	CON	EXERCISE2	40	50
	587	38.07	38.09	37.85	174	8	CON	EXERCISE2	40	50
	588	38.07	38.08	37.81	174	8	CON	EXERCISE2	40	50
	589	38.06	38.18	37.81	175	8	CON	EXERCISE2	40	50
	590	38.07	38.21	37.85	174	8	CON	EXERCISE2	40	50
	591	38.08	38.24	37.83	177	8	CON	EXERCISE2	40	50
	592	38.08	38.22	37.84	175	8	CON	EXERCISE2	40	50
	593	38.10	38.21	37.91	175	8	CON	EXERCISE2	40	50
	594	38.11	38.27	37.94	176	8	CON	EXERCISE2	40	50
	595	38.10	38.24	37.91	177	8	CON	EXERCISE2	40	50
	596	38.10	38.19	37.91	177	8	CON	EXERCISE2	40	50
	597	38.11	38.24	37.93	175	8	CON	EXERCISE2	40	50
	598	38.12	38.28	37.96	176	8	CON	EXERCISE2	40	50
	599	38.13	38.29	37.97	177	8	CON	EXERCISE2	40	50
	600	38.14	38.27	37.94	177	8	CON	EXERCISE2	40	50
100	601	38.14	38.27	37.83	177	8	CON	EXERCISE2	40	50
	602	38.15	38.20	37.87	178	8	CON	EXERCISE2	40	50
	603	38.15	38.21	37.98	179	8	CON	EXERCISE2	40	50

min	number	Tre	Tes	Tsk-head	HR	ID	condition	period	Ta	RH
	604	38.15	38.33	37.94	177	8	CON	EXERCISE2	40	50
	605	38.15	38.36	37.93	178	8	CON	EXERCISE2	40	50
	606	38.16	38.38	37.95	178	8	CON	EXERCISE2	40	50
	607	38.17	38.36	37.96	180	8	CON	EXERCISE2	40	50
	608	38.17	38.37	37.97	161	8	CON	EXERCISE2	40	50
	609	38.17	38.35	37.99	178	8	CON	EXERCISE2	40	50
	610	38.18	38.33	38.10	179	8	CON	EXERCISE2	40	50
	611	38.18	38.36	38.10	179	8	CON	EXERCISE2	40	50
	612	38.19	38.38	38.03	178	8	CON	EXERCISE2	40	50
	613	38.19	38.41	38.01	180	8	CON	EXERCISE2	40	50
	614	38.19	38.41	37.99	180	8	CON	EXERCISE2	40	50
	615	38.21	38.41	38.03	179	8	CON	EXERCISE2	40	50
	616	38.22	38.41	38.14	180	8	CON	EXERCISE2	40	50
	617	38.22	38.40	38.16	180	8	CON	EXERCISE2	40	50
	618	38.23	38.42	38.08	179	8	CON	EXERCISE2	40	50
	619	38.24	38.47	38.06	181	8	CON	EXERCISE2	40	50
	620	38.25	38.47	38.06	180	8	CON	EXERCISE2	40	50
	621	38.25	38.46	38.10	180	8	CON	EXERCISE2	40	50
	622	38.25	38.49	38.12	180	8	CON	EXERCISE2	40	50
	623	38.26	38.44	38.12	180	8	CON	EXERCISE2	40	50
	624	38.26	38.43	38.23	179	8	CON	EXERCISE2	40	50
	625	38.27	38.50	38.23	180	8	CON	EXERCISE2	40	50
	626	38.28	38.45	38.11	180	8	CON	EXERCISE2	40	50
	627	38.28	38.42	38.11	180	8	CON	EXERCISE2	40	50
	628	38.28	38.48	38.13	180	8	CON	EXERCISE2	40	50
	629	38.28	38.40	38.13	180	8	CON	EXERCISE2	40	50
	630	38.29	38.41	38.13	179	8	CON	EXERCISE2	40	50
105	631	38.30	38.49	38.14	180	8	CON	EXERCISE2	40	50
	632	38.31	38.51	38.16	177	8	CON	EXERCISE2	40	50
	633	38.31	38.58	38.16	180	8	CON	EXERCISE2	40	50
	634	38.32	38.60	38.18	180	8	CON	EXERCISE2	40	50
	635	38.32	38.61	38.20	180	8	CON	EXERCISE2	40	50
	636	38.33	38.63	38.22	180	8	CON	EXERCISE2	40	50
	637	38.34	38.65	38.23	180	8	CON	EXERCISE2	40	50
	638	38.34	38.66	38.22	180	8	CON	EXERCISE2	40	50
	639	38.35	38.67	38.23	179	8	CON	EXERCISE2	40	50
	640	38.35	38.70	38.23	181	8	CON	EXERCISE2	40	50
	641	38.35	38.70	38.24	179	8	CON	EXERCISE2	40	50
	642	38.36	38.70	38.26	179	8	CON	EXERCISE2	40	50
	643	38.36	38.58	38.26	180	8	CON	EXERCISE2	40	50
	644	38.37	38.54	38.26	180	8	CON	REST3	28	50
	645	38.38	38.67	38.25	158	8	CON	REST3	28	50
	646	38.39	38.71	38.23	174	8	CON	REST3	28	50
	647	38.39	38.70	38.18	176	8	CON	REST3	28	50
	648	38.38	38.69	38.04	174	8	CON	REST3	28	50
	649	38.39	38.74	37.83	164	8	CON	REST3	28	50

min	number	Tre	Tes	Tsk-head	HR	ID	condition	period	Ta	RH
	650	38.40	38.81	37.70	167	8	CON	REST3	28	50
	651	38.41	38.66	37.63	163	8	CON	REST3	28	50
	652	38.42	38.66	37.60	153	8	CON	REST3	28	50
	653	38.43	38.84	37.60	150	8	CON	REST3	28	50
	654	38.43	38.88	37.62	150	8	CON	REST3	28	50
	655	38.44	38.85	37.63	147	8	CON	REST3	28	50
	656	38.45	38.87	37.56	146	8	CON	REST3	28	50
	657	38.46	38.89	37.52	147	8	CON	REST3	28	50
	658	38.47	38.87	37.55	143	8	CON	REST3	28	50
	659	38.48	38.84	37.55	144	8	CON	REST3	28	50
	660	38.48	38.82	37.54	144	8	CON	REST3	28	50
110	661	38.48	38.83	37.50	140	8	CON	REST3	28	50
	662	38.49	38.80	37.47	140	8	CON	REST3	28	50
	663	38.49	38.80	37.46	141	8	CON	REST3	28	50
	664	38.50	38.71	37.40	140	8	CON	REST3	28	50
	665	38.50	38.67	37.31	138	8	CON	REST3	28	50
	666	38.51	38.69	37.31	136	8	CON	REST3	28	50
	667	38.51	38.63	37.33	140	8	CON	REST3	28	50
	668	38.51	38.56	37.31	142	8	CON	REST3	28	50
	669	38.52	38.51	37.34	140	8	CON	REST3	28	50
	670	38.52	38.54	37.38	139	8	CON	REST3	28	50
	671	38.54	38.58	37.36	137	8	CON	REST3	28	50
	672	38.54	38.57	37.30	134	8	CON	REST3	28	50
	673	38.54	38.55	37.30	132	8	CON	REST3	28	50
	674	38.54	38.48	37.29	137	8	CON	REST3	28	50
	675	38.55	38.43	37.27	135	8	CON	REST3	28	50
	676	38.57	38.49	37.25	136	8	CON	REST3	28	50
	677	38.57	38.44	37.22	133	8	CON	REST3	28	50
	678	38.57	38.33	37.18	131	8	CON	REST3	28	50
	679	38.58	38.32	37.12	134	8	CON	REST3	28	50
	680	38.58	38.34	37.12	130	8	CON	REST3	28	50
	681	38.59	38.19	37.07	134	8	CON	REST3	28	50
	682	38.60	38.13	37.03	134	8	CON	REST3	28	50
	683	38.60	38.25	37.04	139	8	CON	REST3	28	50
	684	38.60	38.23	36.99	135	8	CON	REST3	28	50
	685	38.60	38.17	36.93	134	8	CON	REST3	28	50
	686	38.61	38.15	36.88	132	8	CON	REST3	28	50
	687	38.61	38.16	36.89	127	8	CON	REST3	28	50
	688	38.61	38.13	36.90	128	8	CON	REST3	28	50
	689	38.62	38.14	36.81	133	8	CON	REST3	28	50
	690	38.63	38.13	36.73	132	8	CON	REST3	28	50
115	691	38.63	38.13	36.71	136	8	CON	REST3	28	50
	692	38.64	38.06	36.69	138	8	CON	REST3	28	50
	693	38.64	38.01	36.75	138	8	CON	REST3	28	50
	694	38.64	38.05	36.75	133	8	CON	REST3	28	50
	695	38.65	38.11	36.69	130	8	CON	REST3	28	50

min	number	Tre	Tes	Tsk-head	HR	ID	condition	period	Ta	RH
	696	38.64	38.12	36.70	129	8	CON	REST3	28	50
	697	38.63	38.09	36.70	131	8	CON	REST3	28	50
	698	38.64	38.15	36.57	127	8	CON	REST3	28	50
	699	38.64	38.14	36.41	129	8	CON	REST3	28	50
	700	38.64	38.10	36.39	133	8	CON	REST3	28	50
	701	38.65	38.04	36.45	130	8	CON	REST3	28	50
	702	38.66	38.00	36.51	126	8	CON	REST3	28	50
	703	38.67	38.02	36.49	125	8	CON	REST3	28	50
	704	38.66	38.00	36.47	126	8	CON	REST3	28	50
	705	38.66	37.95	36.47	126	8	CON	REST3	28	50
	706	38.66	37.89	36.48	123	8	CON	REST3	28	50
	707	38.66	37.87	36.50	123	8	CON	REST3	28	50
	708	38.66	37.94	36.42	124	8	CON	REST3	28	50
	709	38.66	37.95	36.27	120	8	CON	REST3	28	50
0	1	36.76	36.49	34.46	77	9	CON	REST1	28	50
	2	36.75	36.49	34.48	80	9	CON	REST1	28	50
	3	36.76	36.48	34.54	80	9	CON	REST1	28	50
	4	36.77	36.47	34.56	76	9	CON	REST1	28	50
	5	36.78	36.45	34.53	81	9	CON	REST1	28	50
	6	36.79	36.43	34.53	77	9	CON	REST1	28	50
	7	36.79	36.43	34.54	75	9	CON	REST1	28	50
	8	36.79	36.42	34.52	79	9	CON	REST1	28	50
	9	36.78	36.37	34.49	84	9	CON	REST1	28	50
	10	36.78	36.37	34.49	75	9	CON	REST1	28	50
	11	36.78	36.41	34.51	75	9	CON	REST1	28	50
	12	36.78	36.44	34.49	72	9	CON	REST1	28	50
	13	36.77	36.44	34.47	70	9	CON	REST1	28	50
	14	36.77	36.43	34.48	72	9	CON	REST1	28	50
	15	36.78	36.43	34.52	72	9	CON	REST1	28	50
	16	36.78	36.43	34.54	73	9	CON	REST1	28	50
	17	36.78	36.42	34.54	73	9	CON	REST1	28	50
	18	36.78	36.43	34.54	74	9	CON	REST1	28	50
	19	36.78	36.45	34.53	77	9	CON	REST1	28	50
	20	36.79	36.44	34.54	76	9	CON	REST1	28	50
	21	36.78	36.42	34.54	73	9	CON	REST1	28	50
	22	36.78	36.43	34.52	76	9	CON	REST1	28	50
	23	36.78	36.46	34.51	75	9	CON	REST1	28	50
	24	36.78	36.46	34.50	75	9	CON	REST1	28	50
	25	36.78	36.45	34.53	79	9	CON	REST1	28	50
	26	36.77	36.43	34.53	72	9	CON	REST1	28	50
	27	36.76	36.42	34.53	74	9	CON	REST1	28	50
	28	36.76	36.44	34.54	81	9	CON	REST1	28	50
	29	36.76	36.39	34.52	85	9	CON	REST1	28	50
	30	36.75	36.36	34.50	70	9	CON	REST1	28	50
5	31	36.74	36.40	34.50	72	9	CON	REST1	28	50
	32	36.72	36.42	34.46	68	9	CON	REST1	28	50

min	number	Tre	Tes	Tsk-head	HR	ID	condition	period	Ta	RH
	33	36.72	36.43	34.41	69	9	CON	REST1	28	50
	34	36.72	36.45	34.39	69	9	CON	REST1	28	50
	35	36.72	36.45	34.39	72	9	CON	REST1	28	50
	36	36.72	36.44	34.41	75	9	CON	REST1	28	50
	37	36.71	36.41	34.42	85	9	CON	REST1	28	50
	38	36.69	36.40	34.43	74	9	CON	REST1	28	50
	39	36.68	36.41	34.45	72	9	CON	REST1	28	50
	40	36.68	36.41	34.47	74	9	CON	REST1	28	50
	41	36.69	36.39	34.48	71	9	CON	REST1	28	50
	42	36.68	36.38	34.50	70	9	CON	REST1	28	50
	43	36.68	36.40	34.51	70	9	CON	REST1	28	50
	44	36.68	36.41	34.51	63	9	CON	REST1	28	50
	45	36.68	36.39	34.51	69	9	CON	REST1	28	50
	46	36.68	36.38	34.54	74	9	CON	REST1	28	50
	47	36.67	36.38	34.53	68	9	CON	REST1	28	50
	48	36.68	36.39	34.55	85	9	CON	REST1	28	50
	49	36.70	36.39	34.61	75	9	CON	REST1	28	50
	50	36.71	36.38	34.63	72	9	CON	REST1	28	50
	51	36.72	36.38	34.64	79	9	CON	REST1	28	50
	52	36.72	36.38	34.65	74	9	CON	REST1	28	50
	53	36.72	36.39	34.62	76	9	CON	REST1	28	50
	54	36.72	36.39	34.60	71	9	CON	REST1	28	50
	55	36.73	36.40	34.58	69	9	CON	REST1	28	50
	56	36.73	36.41	34.55	69	9	CON	REST1	28	50
	57	36.73	36.40	34.55	72	9	CON	REST1	28	50
	58	36.73	36.39	34.56	70	9	CON	REST1	28	50
	59	36.73	36.39	34.56	71	9	CON	REST1	28	50
	60	36.73	36.40	34.55	71	9	CON	REST1	28	50
10	61	36.73	36.41	34.51	71	9	CON	REST1	28	50
	62	36.73	36.40	34.50	69	9	CON	REST1	28	50
	63	36.73	36.39	34.51	70	9	CON	REST1	28	50
	64	36.73	36.40	34.52	68	9	CON	REST1	28	50
	65	36.73	36.42	34.52	67	9	CON	REST1	28	50
	66	36.74	36.42	34.55	77	9	CON	REST1	28	50
	67	36.74	36.38	34.57	71	9	CON	REST1	28	50
	68	36.74	36.36	34.58	67	9	CON	REST1	28	50
	69	36.73	36.38	34.58	70	9	CON	REST1	28	50
	70	36.72	36.39	34.58	68	9	CON	REST1	28	50
	71	36.72	36.40	34.57	65	9	CON	REST1	28	50
	72	36.72	36.40	34.55	65	9	CON	REST1	28	50
	73	36.73	36.39	34.52	71	9	CON	REST1	28	50
	74	36.73	36.41	34.52	63	9	CON	REST1	28	50
	75	36.73	36.40	34.53	71	9	CON	REST1	28	50
	76	36.73	36.41	34.51	65	9	CON	REST1	28	50
	77	36.73	36.41	34.52	67	9	CON	REST1	28	50
	78	36.73	36.40	34.51	71	9	CON	REST1	28	50

min	number	Tre	Tes	Tsk-head	HR	ID	condition	period	Ta	RH
	79	36.73	36.41	34.49	60	9	CON	REST1	28	50
	80	36.73	36.44	34.47	69	9	CON	REST1	28	50
	81	36.73	36.45	34.46	69	9	CON	REST1	28	50
	82	36.74	36.45	34.48	68	9	CON	REST1	28	50
	83	36.73	36.45	34.48	64	9	CON	REST1	28	50
	84	36.73	36.45	34.47	68	9	CON	REST1	28	50
	85	36.73	36.44	34.51	66	9	CON	REST1	28	50
	86	36.73	36.44	34.57	75	9	CON	REST1	28	50
	87	36.73	36.44	34.61	64	9	CON	REST1	28	50
	88	36.73	36.44	34.63	68	9	CON	REST1	28	50
	89	36.73	36.43	34.64	70	9	CON	REST1	28	50
	90	36.74	36.42	34.66	60	9	CON	REST1	28	50
15	91	36.74	36.42	34.67	62	9	CON	REST1	28	50
	92	36.73	36.43	34.65	61	9	CON	REST1	28	50
	93	36.73	36.43	34.62	80	9	CON	REST1	28	50
	94	36.74	36.42	34.65	79	9	CON	REST1	28	50
	95	36.74	36.40	34.70	79	9	CON	REST1	28	50
	96	36.75	36.40	34.68	83	9	CON	REST1	28	50
	97	36.74	36.39	34.66	85	9	CON	REST1	28	50
	98	36.72	36.35	34.64	97	9	CON	REST1	28	50
	99	36.71	36.35	34.61	95	9	CON	REST1	28	50
	100	36.72	36.38	34.60	92	9	CON	REST1	28	50
	101	36.71	36.40	34.60	87	9	CON	REST1	28	50
	102	36.70	36.45	34.60	81	9	CON	REST1	28	50
	103	36.70	36.44	34.67	80	9	CON	REST1	40	50
	104	36.69	36.36	34.71	93	9	CON	REST1	40	50
	105	36.68	36.34	34.80	101	9	CON	REST1	40	50
	106	36.68	36.30	34.95	83	9	CON	REST1	40	50
	107	36.68	36.26	35.03	78	9	CON	REST1	40	50
	108	36.67	36.28	35.12	83	9	CON	REST1	40	50
	109	36.65	36.27	35.22	81	9	CON	REST1	40	50
	110	36.64	36.26	35.32	76	9	CON	REST1	40	50
	111	36.65	36.26	35.44	74	9	CON	REST1	40	50
	112	36.65	36.26	35.54	74	9	CON	REST1	40	50
	113	36.64	36.27	35.62	75	9	CON	REST1	40	50
	114	36.64	36.26	35.67	74	9	CON	REST1	40	50
	115	36.64	36.25	35.71	75	9	CON	REST1	40	50
	116	36.64	36.24	35.76	77	9	CON	REST1	40	50
	117	36.65	36.24	35.79	78	9	CON	REST1	40	50
	118	36.65	36.23	35.82	79	9	CON	REST1	40	50
	119	36.64	36.21	35.85	79	9	CON	REST1	40	50
	120	36.64	36.19	35.87	77	9	CON	REST1	40	50
20	121	36.64	36.19	35.91	73	9	CON	REST1	40	50
	122	36.63	36.18	35.93	77	9	CON	REST1	40	50
	123	36.63	36.18	35.94	79	9	CON	REST1	40	50
	124	36.63	36.18	35.97	79	9	CON	REST1	40	50

min	number	Tre	Tes	Tsk-head	HR	ID	condition	period	Ta	RH
	125	36.64	36.19	35.99	90	9	CON	REST1	40	50
	126	36.65	36.19	36.01	74	9	CON	REST1	40	50
	127	36.66	36.18	36.02	75	9	CON	REST1	40	50
	128	36.67	36.18	36.03	78	9	CON	REST1	40	50
	129	36.67	36.18	36.04	81	9	CON	REST1	40	50
	130	36.67	36.19	36.05	85	9	CON	REST1	40	50
	131	36.68	36.21	36.06	83	9	CON	REST1	40	50
	132	36.68	36.22	36.07	80	9	CON	REST1	40	50
	133	36.67	36.23	36.07	83	9	CON	REST1	40	50
	134	36.67	36.23	36.06	120	9	CON	REST1	40	50
	135	36.65	36.23	36.08	102	9	CON	REST1	40	50
	136	36.63	36.23	36.10	97	9	CON	REST1	40	50
	137	36.62	36.23	36.09	100	9	CON	REST1	40	50
	138	36.63	36.23	36.07	98	9	CON	REST1	40	50
	139	36.63	36.23	36.07	97	9	CON	EXERCISE1	40	50
	140	36.64	36.23	36.08	83	9	CON	EXERCISE1	40	50
	141	36.63	36.24	36.09	96	9	CON	EXERCISE1	40	50
	142	36.62	36.24	36.10	100	9	CON	EXERCISE1	40	50
	143	36.62	36.27	36.11	106	9	CON	EXERCISE1	40	50
	144	36.62	36.28	36.11	108	9	CON	EXERCISE1	40	50
	145	36.61	36.26	36.11	111	9	CON	EXERCISE1	40	50
	146	36.61	36.27	36.11	109	9	CON	EXERCISE1	40	50
	147	36.61	36.29	36.11	106	9	CON	EXERCISE1	40	50
	148	36.61	36.29	36.10	107	9	CON	EXERCISE1	40	50
	149	36.62	36.31	36.10	110	9	CON	EXERCISE1	40	50
	150	36.62	36.32	36.11	110	9	CON	EXERCISE1	40	50
25	151	36.62	36.33	36.11	111	9	CON	EXERCISE1	40	50
	152	36.62	36.33	36.15	112	9	CON	EXERCISE1	40	50
	153	36.63	36.36	36.16	106	9	CON	EXERCISE1	40	50
	154	36.64	36.37	36.14	108	9	CON	EXERCISE1	40	50
	155	36.64	36.37	36.16	111	9	CON	EXERCISE1	40	50
	156	36.64	36.38	36.17	106	9	CON	EXERCISE1	40	50
	157	36.64	36.39	36.18	108	9	CON	EXERCISE1	40	50
	158	36.65	36.39	36.20	109	9	CON	EXERCISE1	40	50
	159	36.65	36.40	36.23	112	9	CON	EXERCISE1	40	50
	160	36.65	36.40	36.27	112	9	CON	EXERCISE1	40	50
	161	36.65	36.41	36.28	115	9	CON	EXERCISE1	40	50
	162	36.65	36.46	36.30	117	9	CON	EXERCISE1	40	50
	163	36.65	36.55	36.32	115	9	CON	EXERCISE1	40	50
	164	36.65	36.57	36.35	115	9	CON	EXERCISE1	40	50
	165	36.66	36.42	36.38	119	9	CON	EXERCISE1	40	50
	166	36.66	36.39	36.39	116	9	CON	EXERCISE1	40	50
	167	36.67	36.49	36.41	115	9	CON	EXERCISE1	40	50
	168	36.68	36.51	36.44	115	9	CON	EXERCISE1	40	50
	169	36.68	36.52	36.44	117	9	CON	EXERCISE1	40	50
	170	36.66	36.53	36.45	112	9	CON	EXERCISE1	40	50

min	number	Tre	Tes	Tsk-head	HR	ID	condition	period	Ta	RH
	171	36.66	36.53	36.48	114	9	CON	EXERCISE1	40	50
	172	36.67	36.52	36.44	118	9	CON	EXERCISE1	40	50
	173	36.68	36.53	36.43	117	9	CON	EXERCISE1	40	50
	174	36.68	36.52	36.48	120	9	CON	EXERCISE1	40	50
	175	36.68	36.52	36.50	118	9	CON	EXERCISE1	40	50
	176	36.68	36.55	36.53	112	9	CON	EXERCISE1	40	50
	177	36.68	36.53	36.54	117	9	CON	EXERCISE1	40	50
	178	36.68	36.53	36.54	119	9	CON	EXERCISE1	40	50
	179	36.68	36.54	36.55	122	9	CON	EXERCISE1	40	50
	180	36.69	36.55	36.56	116	9	CON	EXERCISE1	40	50
30	181	36.70	36.59	36.56	117	9	CON	EXERCISE1	40	50
	182	36.70	36.60	36.56	118	9	CON	EXERCISE1	40	50
	183	36.70	36.60	36.58	122	9	CON	EXERCISE1	40	50
	184	36.70	36.62	36.58	126	9	CON	EXERCISE1	40	50
	185	36.70	36.63	36.58	120	9	CON	EXERCISE1	40	50
	186	36.71	36.63	36.58	117	9	CON	EXERCISE1	40	50
	187	36.72	36.63	36.59	123	9	CON	EXERCISE1	40	50
	188	36.73	36.63	36.60	118	9	CON	EXERCISE1	40	50
	189	36.73	36.64	36.60	119	9	CON	EXERCISE1	40	50
	190	36.73	36.65	36.61	121	9	CON	EXERCISE1	40	50
	191	36.73	36.66	36.62	123	9	CON	EXERCISE1	40	50
	192	36.72	36.66	36.61	121	9	CON	EXERCISE1	40	50
	193	36.73	36.64	36.61	124	9	CON	EXERCISE1	40	50
	194	36.74	36.59	36.61	125	9	CON	EXERCISE1	40	50
	195	36.76	36.61	36.62	126	9	CON	EXERCISE1	40	50
	196	36.76	36.66	36.62	123	9	CON	EXERCISE1	40	50
	197	36.75	36.68	36.62	120	9	CON	EXERCISE1	40	50
	198	36.76	36.69	36.64	123	9	CON	EXERCISE1	40	50
	199	36.77	36.70	36.64	117	9	CON	EXERCISE1	40	50
	200	36.77	36.70	36.64	119	9	CON	EXERCISE1	40	50
	201	36.77	36.68	36.66	126	9	CON	EXERCISE1	40	50
	202	36.78	36.68	36.66	125	9	CON	EXERCISE1	40	50
	203	36.79	36.69	36.66	121	9	CON	EXERCISE1	40	50
	204	36.79	36.69	36.67	121	9	CON	EXERCISE1	40	50
	205	36.79	36.72	36.68	125	9	CON	EXERCISE1	40	50
	206	36.80	36.75	36.68	126	9	CON	EXERCISE1	40	50
	207	36.81	36.76	36.69	125	9	CON	EXERCISE1	40	50
	208	36.81	36.77	36.69	123	9	CON	EXERCISE1	40	50
	209	36.81	36.75	36.69	123	9	CON	EXERCISE1	40	50
	210	36.81	36.75	36.70	125	9	CON	EXERCISE1	40	50
35	211	36.81	36.77	36.70	124	9	CON	EXERCISE1	40	50
	212	36.81	36.77	36.69	123	9	CON	EXERCISE1	40	50
	213	36.81	36.79	36.69	124	9	CON	EXERCISE1	40	50
	214	36.82	36.80	36.70	123	9	CON	EXERCISE1	40	50
	215	36.82	36.80	36.72	119	9	CON	EXERCISE1	40	50
	216	36.82	36.80	36.73	121	9	CON	EXERCISE1	40	50

min	number	Tre	Tes	Tsk-head	HR	ID	condition	period	Ta	RH
	217	36.82	36.82	36.73	125	9	CON	EXERCISE1	40	50
	218	36.82	36.81	36.74	128	9	CON	EXERCISE1	40	50
	219	36.83	36.80	36.75	126	9	CON	EXERCISE1	40	50
	220	36.84	36.83	36.77	126	9	CON	EXERCISE1	40	50
	221	36.84	36.84	36.79	126	9	CON	EXERCISE1	40	50
	222	36.85	36.85	36.80	125	9	CON	EXERCISE1	40	50
	223	36.85	36.86	36.80	125	9	CON	EXERCISE1	40	50
	224	36.86	36.87	36.80	126	9	CON	EXERCISE1	40	50
	225	36.87	36.88	36.81	128	9	CON	EXERCISE1	40	50
	226	36.87	36.87	36.81	130	9	CON	EXERCISE1	40	50
	227	36.88	36.88	36.79	131	9	CON	EXERCISE1	40	50
	228	36.88	36.91	36.77	131	9	CON	EXERCISE1	40	50
	229	36.89	36.93	36.78	129	9	CON	EXERCISE1	40	50
	230	36.90	36.94	36.78	127	9	CON	EXERCISE1	40	50
	231	36.90	36.95	36.78	128	9	CON	EXERCISE1	40	50
	232	36.91	36.96	36.79	128	9	CON	EXERCISE1	40	50
	233	36.91	36.96	36.80	126	9	CON	EXERCISE1	40	50
	234	36.91	36.95	36.80	126	9	CON	EXERCISE1	40	50
	235	36.92	36.96	36.80	126	9	CON	EXERCISE1	40	50
	236	36.92	36.97	36.82	130	9	CON	EXERCISE1	40	50
	237	36.93	36.98	36.84	134	9	CON	EXERCISE1	40	50
	238	36.95	37.00	36.85	133	9	CON	EXERCISE1	40	50
	239	36.95	36.96	36.85	135	9	CON	EXERCISE1	40	50
	240	36.95	36.95	36.84	130	9	CON	EXERCISE1	40	50
40	241	36.96	37.00	36.85	130	9	CON	EXERCISE1	40	50
	242	36.96	37.01	36.86	130	9	CON	EXERCISE1	40	50
	243	36.97	37.03	36.86	132	9	CON	EXERCISE1	40	50
	244	36.97	37.04	36.87	131	9	CON	EXERCISE1	40	50
	245	36.98	37.04	36.89	129	9	CON	EXERCISE1	40	50
	246	36.99	37.05	36.90	130	9	CON	EXERCISE1	40	50
	247	36.99	37.07	36.90	128	9	CON	EXERCISE1	40	50
	248	37.00	37.08	36.90	127	9	CON	EXERCISE1	40	50
	249	37.01	37.08	36.90	128	9	CON	EXERCISE1	40	50
	250	37.01	37.09	36.91	131	9	CON	EXERCISE1	40	50
	251	37.02	37.09	36.91	134	9	CON	EXERCISE1	40	50
	252	37.03	37.09	36.90	136	9	CON	EXERCISE1	40	50
	253	37.02	37.10	36.91	131	9	CON	EXERCISE1	40	50
	254	37.03	37.11	36.92	128	9	CON	EXERCISE1	40	50
	255	37.04	37.12	36.94	129	9	CON	EXERCISE1	40	50
	256	37.04	37.07	36.95	133	9	CON	EXERCISE1	40	50
	257	37.05	37.06	36.94	132	9	CON	EXERCISE1	40	50
	258	37.06	37.12	36.95	128	9	CON	EXERCISE1	40	50
	259	37.06	37.14	36.95	130	9	CON	EXERCISE1	40	50
	260	37.07	37.15	36.94	129	9	CON	EXERCISE1	40	50
	261	37.08	37.16	36.96	130	9	CON	EXERCISE1	40	50
	262	37.09	37.16	36.97	129	9	CON	EXERCISE1	40	50

min	number	Tre	Tes	Tsk-head	HR	ID	condition	period	Ta	RH
	263	37.09	37.17	36.98	128	9	CON	EXERCISE1	40	50
	264	37.09	37.18	36.99	128	9	CON	EXERCISE1	40	50
	265	37.09	37.18	36.99	128	9	CON	EXERCISE1	40	50
	266	37.10	37.19	37.00	128	9	CON	EXERCISE1	40	50
	267	37.10	37.20	37.00	129	9	CON	EXERCISE1	40	50
	268	37.11	37.20	37.00	130	9	CON	EXERCISE1	40	50
	269	37.12	37.17	37.01	132	9	CON	EXERCISE1	40	50
	270	37.12	37.16	37.01	126	9	CON	EXERCISE1	40	50
45	271	37.12	37.19	37.03	129	9	CON	EXERCISE1	40	50
	272	37.13	37.21	37.03	133	9	CON	EXERCISE1	40	50
	273	37.14	37.24	37.03	130	9	CON	EXERCISE1	40	50
	274	37.14	37.25	37.03	131	9	CON	EXERCISE1	40	50
	275	37.15	37.24	37.01	132	9	CON	EXERCISE1	40	50
	276	37.15	37.25	37.03	132	9	CON	EXERCISE1	40	50
	277	37.15	37.25	37.05	131	9	CON	EXERCISE1	40	50
	278	37.16	37.26	37.06	131	9	CON	EXERCISE1	40	50
	279	37.17	37.26	37.07	129	9	CON	EXERCISE1	40	50
	280	37.18	37.26	37.06	129	9	CON	EXERCISE1	40	50
	281	37.18	37.27	37.05	134	9	CON	EXERCISE1	40	50
	282	37.19	37.28	37.04	134	9	CON	EXERCISE1	40	50
	283	37.19	37.29	37.05	131	9	CON	EXERCISE1	40	50
	284	37.20	37.27	37.05	134	9	CON	EXERCISE1	40	50
	285	37.21	37.27	37.05	133	9	CON	EXERCISE1	40	50
	286	37.22	37.31	37.06	134	9	CON	EXERCISE1	40	50
	287	37.22	37.31	37.08	135	9	CON	EXERCISE1	40	50
	288	37.23	37.31	37.09	135	9	CON	EXERCISE1	40	50
	289	37.24	37.33	37.10	134	9	CON	EXERCISE1	40	50
	290	37.24	37.33	37.11	131	9	CON	EXERCISE1	40	50
	291	37.24	37.35	37.11	132	9	CON	EXERCISE1	40	50
	292	37.25	37.37	37.12	137	9	CON	EXERCISE1	40	50
	293	37.25	37.38	37.14	136	9	CON	EXERCISE1	40	50
	294	37.25	37.38	37.14	137	9	CON	EXERCISE1	40	50
	295	37.26	37.39	37.14	134	9	CON	EXERCISE1	40	50
	296	37.27	37.39	37.15	131	9	CON	EXERCISE1	40	50
	297	37.27	37.39	37.14	131	9	CON	EXERCISE1	40	50
	298	37.27	37.40	37.15	132	9	CON	EXERCISE1	40	50
	299	37.27	37.41	37.16	132	9	CON	EXERCISE1	40	50
	300	37.28	37.43	37.17	134	9	CON	EXERCISE1	40	50
50	301	37.29	37.44	37.17	135	9	CON	EXERCISE1	40	50
	302	37.30	37.44	37.17	133	9	CON	EXERCISE1	40	50
	303	37.30	37.43	37.18	132	9	CON	EXERCISE1	40	50
	304	37.30	37.42	37.17	135	9	CON	EXERCISE1	40	50
	305	37.31	37.44	37.18	136	9	CON	EXERCISE1	40	50
	306	37.32	37.44	37.19	134	9	CON	EXERCISE1	40	50
	307	37.33	37.45	37.20	134	9	CON	EXERCISE1	40	50
	308	37.34	37.46	37.20	134	9	CON	EXERCISE1	40	50

min	number	Tre	Tes	Tsk-head	HR	ID	condition	period	Ta	RH
	309	37.34	37.45	37.20	136	9	CON	EXERCISE1	40	50
	310	37.36	37.46	37.22	135	9	CON	EXERCISE1	40	50
	311	37.37	37.41	37.24	138	9	CON	EXERCISE1	40	50
	312	37.37	37.40	37.25	134	9	CON	EXERCISE1	40	50
	313	37.37	37.46	37.25	134	9	CON	EXERCISE1	40	50
	314	37.37	37.48	37.26	137	9	CON	EXERCISE1	40	50
	315	37.38	37.51	37.26	140	9	CON	EXERCISE1	40	50
	316	37.39	37.52	37.26	140	9	CON	EXERCISE1	40	50
	317	37.39	37.52	37.26	136	9	CON	EXERCISE1	40	50
	318	37.39	37.50	37.26	137	9	CON	EXERCISE1	40	50
	319	37.39	37.49	37.25	132	9	CON	EXERCISE1	40	50
	320	37.40	37.52	37.28	134	9	CON	REST2	28	50
	321	37.41	37.52	37.33	132	9	CON	REST2	28	50
	322	37.41	37.52	37.18	130	9	CON	REST2	28	50
	323	37.41	37.53	36.92	116	9	CON	REST2	28	50
	324	37.42	37.55	36.79	110	9	CON	REST2	28	50
	325	37.42	37.53	36.52	116	9	CON	REST2	28	50
	326	37.42	37.41	36.35	115	9	CON	REST2	28	50
	327	37.43	37.39	36.40	115	9	CON	REST2	28	50
	328	37.43	37.45	36.37	113	9	CON	REST2	28	50
	329	37.44	37.44	36.35	105	9	CON	REST2	28	50
	330	37.45	37.42	36.37	104	9	CON	REST2	28	50
55	331	37.45	37.37	36.39	108	9	CON	REST2	28	50
	332	37.45	37.37	36.35	116	9	CON	REST2	28	50
	333	37.46	37.38	36.30	113	9	CON	REST2	28	50
	334	37.46	37.29	36.27	107	9	CON	REST2	28	50
	335	37.46	37.25	36.24	100	9	CON	REST2	28	50
	336	37.46	37.31	36.23	101	9	CON	REST2	28	50
	337	37.46	-10.08	36.17	99	9	CON	REST2	28	50
	338	37.46	-22.01	36.18	114	9	CON	REST2	28	50
	339	37.47	15.36	36.19	118	9	CON	REST2	28	50
	340	37.48	-41.97	36.11	110	9	CON	REST2	28	50
	341	37.48	-37.44	36.10	109	9	CON	REST2	28	50
	342	37.48	29.02	36.16	110	9	CON	REST2	28	50
	343	37.47	30.78	36.13	100	9	CON	REST2	28	50
	344	37.48	64.56	36.09	106	9	CON	REST2	28	50
	345	37.49	80.19	36.09	112	9	CON	REST2	28	50
	346	37.49	46.05	36.08	110	9	CON	REST2	28	50
	347	37.49	31.47	36.06	103	9	CON	REST2	28	50
	348	37.50	-0.48	36.05	104	9	CON	REST2	28	50
	349	37.50	-15.29	36.01	95	9	CON	REST2	28	50
	350	37.50	-48.90	35.96	99	9	CON	REST2	28	50
	351	37.50	-96.36	35.92	91	9	CON	REST2	28	50
	352	37.51	-146.31	35.90	94	9	CON	REST2	28	50
	353	37.50	-156.32	35.88	95	9	CON	REST2	28	50
	354	37.49	-153.37	35.82	91	9	CON	REST2	28	50

min	number	Tre	Tes	Tsk-head	HR	ID	condition	period	Ta	RH
	355	37.49	-165.87	35.77	101	9	CON	REST2	28	50
	356	37.49	-92.73	35.74	88	9	CON	REST2	28	50
	357	37.49	-89.93	35.73	95	9	CON	REST2	28	50
	358	37.50	-126.89	35.71	101	9	CON	REST2	28	50
	359	37.50	-140.70	35.67	105	9	CON	REST2	28	50
	360	37.49	-114.20	35.58	99	9	CON	REST2	28	50
60	361	37.49	-68.87	35.51	98	9	CON	REST2	28	50
	362	37.49	-79.22	35.48	92	9	CON	REST2	28	50
	363	37.49	-127.08	35.50	99	9	CON	REST2	28	50
	364	37.49	-104.85	35.53	97	9	CON	REST2	28	50
	365	37.49	-7.00	35.55	100	9	CON	REST2	28	50
	366	37.48	58.22	35.53	94	9	CON	REST2	28	50
	367	37.48	8.95	35.46	92	9	CON	REST2	28	50
	368	37.48	-105.47	35.44	98	9	CON	REST2	28	50
	369	37.48	-30.85	35.44	95	9	CON	REST2	28	50
	370	37.47	79.31	35.39	93	9	CON	REST2	28	50
	371	37.46	-61.53	35.39	90	9	CON	REST2	28	50
	372	37.46	-194.23	35.36	88	9	CON	REST2	28	50
	373	37.45	-194.23	35.30	98	9	CON	REST2	28	50
	374	37.45	-172.60	35.27	91	9	CON	REST2	28	50
	375	37.46	-144.78	35.24	90	9	CON	REST2	28	50
	376	37.46	-172.18	35.21	95	9	CON	REST2	28	50
	377	37.45	-69.86	35.24	87	9	CON	REST2	28	50
	378	37.44	41.45	35.25	83	9	CON	REST2	28	50
	379	37.44	26.04	35.25	84	9	CON	REST2	28	50
	380	37.44	31.69	35.23	85	9	CON	REST2	28	50
	381	37.44	-76.77	35.17	87	9	CON	REST2	28	50
	382	37.43	-193.73	35.15	86	9	CON	REST2	28	50
	383	37.43	-172.16	35.15	86	9	CON	REST2	28	50
	384	37.43	-118.68	35.13	86	9	CON	REST2	28	50
	385	37.42	-91.62	35.09	88	9	CON	REST2	28	50
	386	37.41	-107.68	35.05	87	9	CON	REST2	28	50
	387	37.41	-120.04	34.99	87	9	CON	REST2	28	50
	388	37.40	-127.07	34.95	86	9	CON	REST2	28	50
	389	37.40	-121.72	34.92	89	9	CON	REST2	28	50
	390	37.40	-103.54	34.92	96	9	CON	REST2	28	50
65	391	37.39	-120.18	34.94	93	9	CON	REST2	28	50
	392	37.37	-89.50	34.91	85	9	CON	REST2	28	50
	393	37.36	-71.60	34.85	87	9	CON	REST2	28	50
	394	37.35	-99.20	34.83	91	9	CON	REST2	28	50
	395	37.35	-69.12	34.86	95	9	CON	REST2	28	50
	396	37.34	-41.86	34.85	101	9	CON	REST2	28	50
	397	37.32	-30.30	34.82	97	9	CON	REST2	28	50
	398	37.32	-45.08	34.80	94	9	CON	REST2	28	50
	399	37.32	-34.68	34.76	90	9	CON	REST2	28	50
	400	37.31	48.37	34.69	86	9	CON	REST2	28	50

min	number	Tre	Tes	Tsk-head	HR	ID	condition	period	Ta	RH
	401	37.30	116.52	34.68	89	9	CON	REST2	28	50
	402	37.29	135.67	34.70	89	9	CON	REST2	28	50
	403	37.27	120.66	34.72	82	9	CON	REST2	28	50
	404	37.26	90.91	34.71	82	9	CON	REST2	28	50
	405	37.26	18.94	34.71	86	9	CON	REST2	28	50
	406	37.26	-117.61	34.73	85	9	CON	REST2	28	50
	407	37.26	-134.84	34.73	91	9	CON	REST2	28	50
	408	37.27	-0.62	34.70	81	9	CON	REST2	28	50
	409	37.28	33.01	34.71	86	9	CON	REST2	28	50
	410	37.27	32.31	34.70	84	9	CON	REST2	28	50
	411	37.27	65.74	34.69	84	9	CON	REST2	28	50
	412	37.26	64.98	34.74	92	9	CON	REST2	28	50
	413	37.26	-67.26	34.69	94	9	CON	REST2	28	50
	414	37.26	-192.73	34.62	84	9	CON	REST2	28	50
	415	37.26	-174.28	34.61	89	9	CON	REST2	28	50
	416	37.26	-28.89	34.62	89	9	CON	REST2	28	50
	417	37.26	-47.34	34.61	86	9	CON	REST2	28	50
	418	37.27	-200.00	34.68	91	9	CON	REST2	28	50
	419	37.27	-200.00	34.76	90	9	CON	REST2	28	50
	420	37.27	-200.00	34.70	86	9	CON	REST2	28	50
70	421	37.27	-200.00	34.64	87	9	CON	REST2	28	50
	422	37.27	-194.64	34.69	88	9	CON	REST2	28	50
	423	37.27	-194.64	34.71	84	9	CON	REST2	28	50
	424	37.26	-200.00	34.71	84	9	CON	REST2	28	50
	425	37.26	-174.03	34.77	86	9	CON	REST2	28	50
	426	37.25	-141.75	34.80	95	9	CON	REST2	28	50
	427	37.25	-104.77	34.79	85	9	CON	REST2	28	50
	428	37.25	-83.88	34.80	89	9	CON	REST2	28	50
	429	37.24	-146.83	34.84	88	9	CON	REST2	28	50
	430	37.23	-200.00	34.91	83	9	CON	REST2	28	50
	431	37.24	-200.00	34.92	86	9	CON	REST2	28	50
	432	37.23	-200.00	34.91	83	9	CON	REST2	28	50
	433	37.23	-200.00	34.96	79	9	CON	REST2	28	50
	434	37.22	-200.00	34.88	73	9	CON	REST2	28	50
	435	37.21	-133.23	34.80	76	9	CON	REST2	28	50
	436	37.22	-133.23	34.83	78	9	CON	REST2	28	50
	437	37.21	-200.00	34.91	77	9	CON	REST2	28	50
	438	37.20	-99.44	34.95	80	9	CON	REST2	28	50
	439	37.20	-8.34	34.95	79	9	CON	REST2	40	50
	440	37.21	-18.84	35.04	100	9	CON	REST2	40	50
	441	37.21	-109.94	35.21	110	9	CON	REST2	40	50
	442	37.21	-88.10	35.38	96	9	CON	REST2	40	50
	443	37.20	-88.10	35.51	93	9	CON	REST2	40	50
	444	37.20	-200.00	35.65	82	9	CON	REST2	40	50
	445	37.20	-196.94	35.82	81	9	CON	REST2	40	50
	446	37.20	-196.94	35.95	84	9	CON	REST2	40	50

min	number	Tre	Tes	Tsk-head	HR	ID	condition	period	Ta	RH
	447	37.20	-200.00	36.04	86	9	CON	REST2	40	50
	448	37.19	-200.00	36.10	89	9	CON	REST2	40	50
	449	37.19	-200.00	36.13	86	9	CON	REST2	40	50
	450	37.20	-200.00	36.15	87	9	CON	REST2	40	50
75	451	37.19	-96.12	36.17	91	9	CON	REST2	40	50
	452	37.19	-6.16	36.19	93	9	CON	REST2	40	50
	453	37.19	-110.04	36.22	86	9	CON	REST2	40	50
	454	37.19	-75.71	36.22	87	9	CON	REST2	40	50
	455	37.19	35.14	36.20	97	9	CON	REST2	40	50
	456	37.19	-89.16	36.20	94	9	CON	REST2	40	50
	457	37.18	-72.78	36.20	93	9	CON	REST2	40	50
	458	37.18	113.65	36.18	108	9	CON	REST2	40	50
	459	37.19	182.95	36.16	114	9	CON	REST2	40	50
	460	37.18	183.30	36.19	99	9	CON	REST2	40	50
	461	37.18	286.77	36.23	95	9	CON	REST2	40	50
	462	37.17	400.00	36.26	97	9	CON	REST2	40	50
	463	37.15	229.01	36.29	97	9	CON	EXERCISE2	40	50
	464	37.15	70.99	36.30	99	9	CON	EXERCISE2	40	50
	465	37.15	119.77	36.28	103	9	CON	EXERCISE2	40	50
	466	37.15	206.29	36.25	109	9	CON	EXERCISE2	40	50
	467	37.15	252.14	36.25	114	9	CON	EXERCISE2	40	50
	468	37.15	239.82	36.25	121	9	CON	EXERCISE2	40	50
	469	37.14	238.04	36.24	122	9	CON	EXERCISE2	40	50
	470	37.14	197.32	36.25	122	9	CON	EXERCISE2	40	50
	471	37.14	194.97	36.26	123	9	CON	EXERCISE2	40	50
	472	37.12	223.43	36.27	121	9	CON	EXERCISE2	40	50
	473	37.11	229.56	36.27	119	9	CON	EXERCISE2	40	50
	474	37.10	142.98	36.28	116	9	CON	EXERCISE2	40	50
	475	37.10	35.17	36.29	115	9	CON	EXERCISE2	40	50
	476	37.10	35.89	36.31	122	9	CON	EXERCISE2	40	50
	477	37.10	36.29	36.32	122	9	CON	EXERCISE2	40	50
	478	37.10	110.41	36.33	121	9	CON	EXERCISE2	40	50
	479	37.10	215.18	36.35	122	9	CON	EXERCISE2	40	50
	480	37.10	249.26	36.35	122	9	CON	EXERCISE2	40	50
80	481	37.10	249.81	36.36	121	9	CON	EXERCISE2	40	50
	482	37.11	252.80	36.39	123	9	CON	EXERCISE2	40	50
	483	37.11	253.14	36.40	125	9	CON	EXERCISE2	40	50
	484	37.12	85.30	36.40	126	9	CON	EXERCISE2	40	50
	485	37.11	27.93	36.39	122	9	CON	EXERCISE2	40	50
	486	37.11	124.55	36.40	122	9	CON	EXERCISE2	40	50
	487	37.10	153.70	36.42	122	9	CON	EXERCISE2	40	50
	488	37.10	135.07	36.44	124	9	CON	EXERCISE2	40	50
	489	37.11	96.25	36.46	122	9	CON	EXERCISE2	40	50
	490	37.11	256.83	36.48	126	9	CON	EXERCISE2	40	50
	491	37.11	400.00	36.48	127	9	CON	EXERCISE2	40	50
	492	37.12	400.00	36.50	128	9	CON	EXERCISE2	40	50

min	number	Tre	Tes	Tsk-head	HR	ID	condition	period	Ta	RH
	493	37.13	400.00	36.50	127	9	CON	EXERCISE2	40	50
	494	37.13	400.00	36.50	129	9	CON	EXERCISE2	40	50
	495	37.12	400.00	36.50	134	9	CON	EXERCISE2	40	50
	496	37.11	400.00	36.50	131	9	CON	EXERCISE2	40	50
	497	37.12	400.00	36.51	129	9	CON	EXERCISE2	40	50
	498	37.13	400.00	36.56	125	9	CON	EXERCISE2	40	50
	499	37.13	234.80	36.61	123	9	CON	EXERCISE2	40	50
	500	37.14	36.93	36.62	123	9	CON	EXERCISE2	40	50
	501	37.14	-0.10	36.60	128	9	CON	EXERCISE2	40	50
	502	37.14	-6.32	36.59	129	9	CON	EXERCISE2	40	50
	503	37.14	31.82	36.59	128	9	CON	EXERCISE2	40	50
	504	37.14	235.91	36.60	128	9	CON	EXERCISE2	40	50
	505	37.15	400.00	36.62	126	9	CON	EXERCISE2	40	50
	506	37.15	400.00	36.65	126	9	CON	EXERCISE2	40	50
	507	37.15	400.00	36.66	123	9	CON	EXERCISE2	40	50
	508	37.16	398.26	36.67	126	9	CON	EXERCISE2	40	50
	509	37.16	201.84	36.70	126	9	CON	EXERCISE2	40	50
	510	37.16	57.82	36.74	128	9	CON	EXERCISE2	40	50
85	511	37.17	47.19	36.74	127	9	CON	EXERCISE2	40	50
	512	37.17	131.62	36.73	127	9	CON	EXERCISE2	40	50
	513	37.17	249.76	36.73	126	9	CON	EXERCISE2	40	50
	514	37.18	231.78	36.72	128	9	CON	EXERCISE2	40	50
	515	37.18	320.69	36.70	131	9	CON	EXERCISE2	40	50
	516	37.18	400.00	36.71	134	9	CON	EXERCISE2	40	50
	517	37.18	333.91	36.71	130	9	CON	EXERCISE2	40	50
	518	37.18	253.97	36.71	129	9	CON	EXERCISE2	40	50
	519	37.19	243.27	36.72	131	9	CON	EXERCISE2	40	50
	520	37.19	323.21	36.74	131	9	CON	EXERCISE2	40	50
	521	37.19	400.00	36.74	130	9	CON	EXERCISE2	40	50
	522	37.19	400.00	36.72	127	9	CON	EXERCISE2	40	50
	523	37.20	398.81	36.71	130	9	CON	EXERCISE2	40	50
	524	37.20	346.11	36.70	131	9	CON	EXERCISE2	40	50
	525	37.21	347.30	36.70	128	9	CON	EXERCISE2	40	50
	526	37.21	400.00	36.71	128	9	CON	EXERCISE2	40	50
	527	37.23	400.00	36.74	130	9	CON	EXERCISE2	40	50
	528	37.24	386.05	36.76	127	9	CON	EXERCISE2	40	50
	529	37.24	386.05	36.76	127	9	CON	EXERCISE2	40	50
	530	37.25	400.00	36.79	128	9	CON	EXERCISE2	40	50
	531	37.24	281.47	36.81	126	9	CON	EXERCISE2	40	50
	532	37.25	175.04	36.82	127	9	CON	EXERCISE2	40	50
	533	37.25	213.28	36.82	128	9	CON	EXERCISE2	40	50
	534	37.25	257.43	36.82	134	9	CON	EXERCISE2	40	50
	535	37.26	259.51	36.83	135	9	CON	EXERCISE2	40	50
	536	37.26	319.09	36.83	134	9	CON	EXERCISE2	40	50
	537	37.26	397.30	36.84	134	9	CON	EXERCISE2	40	50
	538	37.26	400.00	36.86	133	9	CON	EXERCISE2	40	50

min	number	Tre	Tes	Tsk-head	HR	ID	condition	period	Ta	RH
	539	37.27	400.00	36.87	132	9	CON	EXERCISE2	40	50
	540	37.28	400.00	36.89	132	9	CON	EXERCISE2	40	50
90	541	37.28	379.75	36.88	131	9	CON	EXERCISE2	40	50
	542	37.28	379.75	36.87	131	9	CON	EXERCISE2	40	50
	543	37.29	399.69	36.86	130	9	CON	EXERCISE2	40	50
	544	37.29	399.69	36.86	134	9	CON	EXERCISE2	40	50
	545	37.30	400.00	36.90	131	9	CON	EXERCISE2	40	50
	546	37.30	400.00	36.93	131	9	CON	EXERCISE2	40	50
	547	37.30	400.00	36.93	133	9	CON	EXERCISE2	40	50
	548	37.31	400.00	36.94	134	9	CON	EXERCISE2	40	50
	549	37.32	400.00	36.95	134	9	CON	EXERCISE2	40	50
	550	37.32	400.00	36.95	133	9	CON	EXERCISE2	40	50
	551	37.33	400.00	36.96	134	9	CON	EXERCISE2	40	50
	552	37.34	400.00	36.97	134	9	CON	EXERCISE2	40	50
	553	37.34	400.00	36.98	136	9	CON	EXERCISE2	40	50
	554	37.34	400.00	36.97	137	9	CON	EXERCISE2	40	50
	555	37.35	400.00	36.97	134	9	CON	EXERCISE2	40	50
	556	37.35	400.00	36.98	135	9	CON	EXERCISE2	40	50
	557	37.35	397.85	36.99	137	9	CON	EXERCISE2	40	50
	558	37.36	397.85	36.99	135	9	CON	EXERCISE2	40	50
	559	37.38	400.00	37.01	136	9	CON	EXERCISE2	40	50
	560	37.38	400.00	37.01	134	9	CON	EXERCISE2	40	50
	561	37.38	400.00	37.01	133	9	CON	EXERCISE2	40	50
	562	37.39	400.00	37.02	135	9	CON	EXERCISE2	40	50
	563	37.39	400.00	37.02	135	9	CON	EXERCISE2	40	50
	564	37.39	394.72	37.02	134	9	CON	EXERCISE2	40	50
	565	37.39	394.72	37.03	134	9	CON	EXERCISE2	40	50
	566	37.40	400.00	37.03	134	9	CON	EXERCISE2	40	50
	567	37.41	400.00	37.04	137	9	CON	EXERCISE2	40	50
	568	37.41	400.00	37.03	137	9	CON	EXERCISE2	40	50
	569	37.41	400.00	37.03	137	9	CON	EXERCISE2	40	50
	570	37.42	400.00	37.06	140	9	CON	EXERCISE2	40	50
95	571	37.43	369.88	37.08	140	9	CON	EXERCISE2	40	50
	572	37.43	369.88	37.09	138	9	CON	EXERCISE2	40	50
	573	37.44	400.00	37.09	136	9	CON	EXERCISE2	40	50
	574	37.45	400.00	37.09	131	9	CON	EXERCISE2	40	50
	575	37.45	355.00	37.05	135	9	CON	EXERCISE2	40	50
	576	37.45	355.00	37.05	140	9	CON	EXERCISE2	40	50
	577	37.46	400.00	37.09	141	9	CON	EXERCISE2	40	50
	578	37.47	400.00	37.11	140	9	CON	EXERCISE2	40	50
	579	37.48	400.00	37.11	137	9	CON	EXERCISE2	40	50
	580	37.48	400.00	37.11	136	9	CON	EXERCISE2	40	50
	581	37.49	400.00	37.12	137	9	CON	EXERCISE2	40	50
	582	37.50	400.00	37.14	137	9	CON	EXERCISE2	40	50
	583	37.50	400.00	37.14	137	9	CON	EXERCISE2	40	50
	584	37.50	400.00	37.12	137	9	CON	EXERCISE2	40	50

min	number	Tre	Tes	Tsk-head	HR	ID	condition	period	Ta	RH
	585	37.51	349.40	37.09	140	9	CON	EXERCISE2	40	50
	586	37.51	349.40	37.10	140	9	CON	EXERCISE2	40	50
	587	37.52	400.00	37.13	140	9	CON	EXERCISE2	40	50
	588	37.53	400.00	37.15	140	9	CON	EXERCISE2	40	50
	589	37.53	364.21	37.16	140	9	CON	EXERCISE2	40	50
	590	37.54	350.58	37.17	138	9	CON	EXERCISE2	40	50
	591	37.54	386.37	37.18	137	9	CON	EXERCISE2	40	50
	592	37.55	357.26	37.19	136	9	CON	EXERCISE2	40	50
	593	37.55	293.90	37.19	136	9	CON	EXERCISE2	40	50
	594	37.56	336.64	37.19	137	9	CON	EXERCISE2	40	50
	595	37.55	373.38	37.18	139	9	CON	EXERCISE2	40	50
	596	37.56	321.95	37.20	137	9	CON	EXERCISE2	40	50
	597	37.58	328.90	37.22	130	9	CON	EXERCISE2	40	50
	598	37.59	300.58	37.22	135	9	CON	EXERCISE2	40	50
	599	37.59	273.33	37.25	140	9	CON	EXERCISE2	40	50
	600	37.59	333.71	37.27	142	9	CON	EXERCISE2	40	50
100	601	37.60	297.80	37.26	140	9	CON	EXERCISE2	40	50
	602	37.60	252.92	37.25	137	9	CON	EXERCISE2	40	50
	603	37.60	270.50	37.24	140	9	CON	EXERCISE2	40	50
	604	37.61	317.77	37.22	141	9	CON	EXERCISE2	40	50
	605	37.62	343.96	37.24	142	9	CON	EXERCISE2	40	50
	606	37.63	291.86	37.27	140	9	CON	EXERCISE2	40	50
	607	37.63	256.78	37.27	140	9	CON	EXERCISE2	40	50
	608	37.64	277.86	37.27	140	9	CON	EXERCISE2	40	50
	609	37.64	309.44	37.27	141	9	CON	EXERCISE2	40	50
	610	37.64	285.30	37.27	140	9	CON	EXERCISE2	40	50
	611	37.64	244.59	37.28	139	9	CON	EXERCISE2	40	50
	612	37.65	240.70	37.29	140	9	CON	EXERCISE2	40	50
	613	37.66	244.42	37.29	140	9	CON	EXERCISE2	40	50
	614	37.66	267.41	37.29	142	9	CON	EXERCISE2	40	50
	615	37.67	280.37	37.30	140	9	CON	EXERCISE2	40	50
	616	37.68	239.85	37.31	141	9	CON	EXERCISE2	40	50
	617	37.69	258.62	37.33	140	9	CON	EXERCISE2	40	50
	618	37.70	290.42	37.33	142	9	CON	EXERCISE2	40	50
	619	37.70	263.93	37.32	141	9	CON	EXERCISE2	40	50
	620	37.71	246.52	37.32	140	9	CON	EXERCISE2	40	50
	621	37.72	252.50	37.33	140	9	CON	EXERCISE2	40	50
	622	37.73	256.12	37.33	141	9	CON	EXERCISE2	40	50
	623	37.73	239.62	37.31	143	9	CON	EXERCISE2	40	50
	624	37.74	216.81	37.29	147	9	CON	EXERCISE2	40	50
	625	37.74	194.10	37.47	143	9	CON	EXERCISE2	40	50
	626	37.74	169.07	37.52	140	9	CON	EXERCISE2	40	50
	627	37.75	146.94	37.36	141	9	CON	EXERCISE2	40	50
	628	37.75	144.97	37.33	143	9	CON	EXERCISE2	40	50
	629	37.75	135.45	37.31	142	9	CON	EXERCISE2	40	50
	630	37.77	136.88	37.28	143	9	CON	EXERCISE2	40	50

min	number	Tre	Tes	Tsk-head	HR	ID	condition	period	Ta	RH
105	631	37.78	149.88	37.28	146	9	CON	EXERCISE2	40	50
	632	37.79	248.79	37.30	146	9	CON	EXERCISE2	40	50
	633	37.79	336.63	37.31	143	9	CON	EXERCISE2	40	50
	634	37.79	314.67	37.30	144	9	CON	EXERCISE2	40	50
	635	37.79	286.94	37.30	144	9	CON	EXERCISE2	40	50
	636	37.79	218.27	37.34	146	9	CON	EXERCISE2	40	50
	637	37.80	186.59	37.35	144	9	CON	EXERCISE2	40	50
	638	37.81	181.03	37.34	144	9	CON	EXERCISE2	40	50
	639	37.81	136.96	37.35	144	9	CON	EXERCISE2	40	50
	640	37.82	138.54	37.35	144	9	CON	EXERCISE2	40	50
	641	37.83	152.62	37.36	141	9	CON	EXERCISE2	40	50
	642	37.84	98.22	37.37	143	9	CON	EXERCISE2	40	50
	643	37.85	121.74	37.38	141	9	CON	EXERCISE2	40	50
	644	37.86	165.22	37.37	143	9	CON	REST3	28	50
	645	37.86	132.10	37.15	141	9	CON	REST3	28	50
	646	37.85	114.92	36.81	129	9	CON	REST3	28	50
	647	37.86	132.27	36.64	126	9	CON	REST3	28	50
	648	37.87	130.24	36.59	122	9	CON	REST3	28	50
	649	37.88	108.42	36.51	122	9	CON	REST3	28	50
	650	37.89	156.57	36.45	119	9	CON	REST3	28	50
	651	37.89	166.55	36.43	119	9	CON	REST3	28	50
	652	37.89	148.65	36.35	118	9	CON	REST3	28	50
	653	37.89	142.90	36.32	123	9	CON	REST3	28	50
	654	37.90	159.07	36.35	128	9	CON	REST3	28	50
	655	37.90	133.46	36.34	122	9	CON	REST3	28	50
	656	37.90	41.67	36.25	114	9	CON	REST3	28	50
	657	37.88	12.87	36.22	115	9	CON	REST3	28	50
	658	37.88	85.38	36.21	112	9	CON	REST3	28	50
	659	37.87	127.27	36.14	113	9	CON	REST3	28	50
	660	37.87	103.21	36.08	112	9	CON	REST3	28	50
110	661	37.87	103.65	36.08	110	9	CON	REST3	28	50
	662	37.87	92.05	36.08	110	9	CON	REST3	28	50
	663	37.87	73.14	36.07	110	9	CON	REST3	28	50
	664	37.87	59.93	36.08	108	9	CON	REST3	28	50
	665	37.86	60.03	36.08	113	9	CON	REST3	28	50
	666	37.86	76.79	36.11	108	9	CON	REST3	28	50
	667	37.86	89.78	36.02	109	9	CON	REST3	28	50
	668	37.86	80.96	35.94	109	9	CON	REST3	28	50
	669	37.85	74.37	35.95	109	9	CON	REST3	28	50
	670	37.85	68.96	35.97	106	9	CON	REST3	28	50
	671	37.85	68.01	35.96	103	9	CON	REST3	28	50
	672	37.85	67.88	35.88	104	9	CON	REST3	28	50
	673	37.84	63.68	35.78	104	9	CON	REST3	28	50
	674	37.84	66.54	35.77	101	9	CON	REST3	28	50
	675	37.84	62.20	35.71	103	9	CON	REST3	28	50
	676	37.84	53.14	35.69	102	9	CON	REST3	28	50

min	number	Tre	Tes	Tsk-head	HR	ID	condition	period	Ta	RH
	677	37.85	-59.48	35.79	102	9	CON	REST3	28	50
	678	37.85	-102.14	35.78	104	9	CON	REST3	28	50
	679	37.84	-49.18	35.73	102	9	CON	REST3	28	50
	680	37.84	168.79	35.75	104	9	CON	REST3	28	50
	681	37.84	400.00	35.75	105	9	CON	REST3	28	50
	682	37.84	400.00	35.68	106	9	CON	REST3	28	50
	683	37.84	381.95	35.57	103	9	CON	REST3	28	50
	684	37.84	381.95	35.51	106	9	CON	REST3	28	50
	685	37.82	400.00	35.58	99	9	CON	REST3	28	50
	686	37.80	375.91	35.50	98	9	CON	REST3	28	50
	687	37.80	359.51	35.24	95	9	CON	REST3	28	50
	688	37.79	334.19	35.26	98	9	CON	REST3	28	50
	689	37.78	350.59	35.40	97	9	CON	REST3	28	50
	690	37.78	381.52	35.43	96	9	CON	REST3	28	50
115	691	37.78	381.52	35.44	98	9	CON	REST3	28	50
	692	37.78	400.00	35.46	93	9	CON	REST3	28	50
	693	37.77	400.00	35.52	92	9	CON	REST3	28	50
	694	37.76	400.00	35.54	98	9	CON	REST3	28	50
	695	37.75	400.00	35.50	98	9	CON	REST3	28	50
	696	37.76	400.00	35.37	93	9	CON	REST3	28	50
	697	37.77	400.00	35.24	90	9	CON	REST3	28	50
	698	37.77	400.00	35.27	92	9	CON	REST3	28	50
	699	37.76	357.19	35.35	93	9	CON	REST3	28	50
	700	37.76	337.34	35.35	96	9	CON	REST3	28	50
	701	37.75	380.15	35.18	99	9	CON	REST3	28	50
	702	37.75	400.00	35.10	93	9	CON	REST3	28	50
	703	37.74	400.00	35.15	91	9	CON	REST3	28	50
	704	37.74	400.00	35.19	94	9	CON	REST3	28	50
	705	37.73	400.00	35.24	99	9	CON	REST3	28	50
	706	37.72	400.00	35.15	93	9	CON	REST3	28	50
	707	37.73	400.00	35.09	90	9	CON	REST3	28	50
	708	37.73	400.00	35.17	95	9	CON	REST3	28	50
	709	37.73	400.00	35.24	95	9	CON	REST3	28	50
0	1	36.45	36.49	35.26	83	1	VEST	REST1	28	50
	2	36.45	36.48	35.27	79	1	VEST	REST1	28	50
	3	36.44	36.49	35.27	78	1	VEST	REST1	28	50
	4	36.44	36.52	35.28	84	1	VEST	REST1	28	50
	5	36.44	36.52	35.29	81	1	VEST	REST1	28	50
	6	36.43	36.50	35.28	80	1	VEST	REST1	28	50
	7	36.43	36.50	35.26	80	1	VEST	REST1	28	50
	8	36.44	36.50	35.27	77	1	VEST	REST1	28	50
	9	36.43	36.50	35.25	75	1	VEST	REST1	28	50
	10	36.43	36.51	35.26	76	1	VEST	REST1	28	50
	11	36.42	36.51	35.29	75	1	VEST	REST1	28	50
	12	36.42	36.53	35.28	77	1	VEST	REST1	28	50
	13	36.43	36.56	35.28	76	1	VEST	REST1	28	50

min	number	Tre	Tes	Tsk-head	HR	ID	condition	period	Ta	RH
	14	36.43	36.55	35.29	73	1	VEST	REST1	28	50
	15	36.43	36.56	35.29	76	1	VEST	REST1	28	50
	16	36.43	36.56	35.30	79	1	VEST	REST1	28	50
	17	36.42	36.54	35.30	77	1	VEST	REST1	28	50
	18	36.42	36.53	35.29	78	1	VEST	REST1	28	50
	19	36.42	36.53	35.28	78	1	VEST	REST1	28	50
	20	36.42	36.54	35.29	77	1	VEST	REST1	28	50
	21	36.43	36.55	35.29	77	1	VEST	REST1	28	50
	22	36.42	36.56	35.29	85	1	VEST	REST1	28	50
	23	36.42	36.55	35.28	81	1	VEST	REST1	28	50
	24	36.42	36.53	35.26	77	1	VEST	REST1	28	50
	25	36.42	36.54	35.27	78	1	VEST	REST1	28	50
	26	36.43	36.54	35.28	81	1	VEST	REST1	28	50
	27	36.42	36.53	35.28	77	1	VEST	REST1	28	50
	28	36.42	36.53	35.27	78	1	VEST	REST1	28	50
	29	36.43	36.54	35.27	80	1	VEST	REST1	28	50
	30	36.42	36.54	35.28	81	1	VEST	REST1	28	50
5	31	36.42	36.53	35.28	81	1	VEST	REST1	28	50
	32	36.42	36.54	35.29	87	1	VEST	REST1	28	50
	33	36.42	36.53	35.30	74	1	VEST	REST1	28	50
	34	36.42	36.54	35.30	84	1	VEST	REST1	28	50
	35	36.42	36.53	35.30	83	1	VEST	REST1	28	50
	36	36.42	36.49	35.29	74	1	VEST	REST1	28	50
	37	36.42	36.52	35.29	72	1	VEST	REST1	28	50
	38	36.42	36.53	35.29	74	1	VEST	REST1	28	50
	39	36.42	36.52	35.28	74	1	VEST	REST1	28	50
	40	36.42	36.52	35.26	76	1	VEST	REST1	28	50
	41	36.42	36.52	35.26	81	1	VEST	REST1	28	50
	42	36.42	36.52	35.28	78	1	VEST	REST1	28	50
	43	36.42	36.54	35.31	74	1	VEST	REST1	28	50
	44	36.42	36.53	35.31	75	1	VEST	REST1	28	50
	45	36.42	36.52	35.32	76	1	VEST	REST1	28	50
	46	36.42	36.53	35.31	76	1	VEST	REST1	28	50
	47	36.41	36.52	35.30	76	1	VEST	REST1	28	50
	48	36.41	36.52	35.30	76	1	VEST	REST1	28	50
	49	36.41	36.52	35.30	74	1	VEST	REST1	28	50
	50	36.42	36.54	35.31	72	1	VEST	REST1	28	50
	51	36.42	36.53	35.32	80	1	VEST	REST1	28	50
	52	36.41	36.52	35.32	76	1	VEST	REST1	28	50
	53	36.42	36.52	35.32	75	1	VEST	REST1	28	50
	54	36.42	36.51	35.32	83	1	VEST	REST1	28	50
	55	36.42	36.52	35.32	72	1	VEST	REST1	28	50
	56	36.43	36.54	35.32	71	1	VEST	REST1	28	50
	57	36.43	36.55	35.32	66	1	VEST	REST1	28	50
	58	36.41	36.54	35.32	76	1	VEST	REST1	28	50
	59	36.41	36.52	35.35	78	1	VEST	REST1	28	50

min	number	Tre	Tes	Tsk-head	HR	ID	condition	period	Ta	RH
	60	36.42	36.52	35.36	82	1	VEST	REST1	28	50
10	61	36.41	36.54	35.36	76	1	VEST	REST1	28	50
	62	36.41	36.54	35.36	76	1	VEST	REST1	28	50
	63	36.41	36.53	35.34	73	1	VEST	REST1	28	50
	64	36.40	36.53	35.33	71	1	VEST	REST1	28	50
	65	36.40	36.55	35.32	78	1	VEST	REST1	28	50
	66	36.40	36.56	35.33	74	1	VEST	REST1	28	50
	67	36.40	36.56	35.33	76	1	VEST	REST1	28	50
	68	36.41	36.57	35.34	87	1	VEST	REST1	28	50
	69	36.41	36.57	35.35	81	1	VEST	REST1	28	50
	70	36.41	36.57	35.37	74	1	VEST	REST1	28	50
	71	36.41	36.56	35.36	73	1	VEST	REST1	28	50
	72	36.40	36.54	35.33	73	1	VEST	REST1	28	50
	73	36.40	36.53	35.32	74	1	VEST	REST1	28	50
	74	36.40	36.54	35.35	73	1	VEST	REST1	28	50
	75	36.40	36.55	35.32	74	1	VEST	REST1	28	50
	76	36.40	36.56	35.28	70	1	VEST	REST1	28	50
	77	36.40	36.56	35.31	76	1	VEST	REST1	28	50
	78	36.40	36.57	35.34	74	1	VEST	REST1	28	50
	79	36.40	36.57	35.32	71	1	VEST	REST1	28	50
	80	36.40	36.58	35.31	72	1	VEST	REST1	28	50
	81	36.40	36.59	35.28	68	1	VEST	REST1	28	50
	82	36.41	36.58	35.27	70	1	VEST	REST1	28	50
	83	36.41	36.57	35.27	68	1	VEST	REST1	28	50
	84	36.42	36.51	35.29	86	1	VEST	REST1	28	50
	85	36.43	36.49	35.33	80	1	VEST	REST1	28	50
	86	36.42	36.52	35.33	73	1	VEST	REST1	28	50
	87	36.41	36.54	35.32	92	1	VEST	REST1	28	50
	88	36.41	36.55	35.31	74	1	VEST	REST1	28	50
	89	36.41	36.54	35.33	91	1	VEST	REST1	28	50
	90	36.42	36.54	35.34	95	1	VEST	REST1	28	50
15	91	36.42	36.56	35.34	91	1	VEST	REST1	28	50
	92	36.41	36.57	35.34	83	1	VEST	REST1	28	50
	93	36.40	36.55	35.35	81	1	VEST	REST1	28	50
	94	36.40	36.56	35.37	73	1	VEST	REST1	28	50
	95	36.40	36.56	35.38	72	1	VEST	REST1	28	50
	96	36.40	36.56	35.41	71	1	VEST	REST1	28	50
	97	36.40	36.57	35.38	75	1	VEST	REST1	28	50
	98	36.41	36.56	35.34	78	1	VEST	REST1	28	50
	99	36.41	36.55	35.35	71	1	VEST	REST1	28	50
	100	36.40	36.57	35.33	71	1	VEST	REST1	28	50
	101	36.40	36.58	35.32	75	1	VEST	REST1	28	50
	102	36.40	36.56	35.35	78	1	VEST	REST1	28	50
	103	36.40	36.56	35.37	84	1	VEST	REST1	40	50
	104	36.40	36.56	35.54	93	1	VEST	REST1	40	50
	105	36.41	36.54	35.79	104	1	VEST	REST1	40	50

min	number	Tre	Tes	Tsk-head	HR	ID	condition	period	Ta	RH
	106	36.42	36.54	35.95	96	1	VEST	REST1	40	50
	107	36.42	36.52	36.05	101	1	VEST	REST1	40	50
	108	36.42	36.50	36.11	94	1	VEST	REST1	40	50
	109	36.42	36.51	36.18	93	1	VEST	REST1	40	50
	110	36.42	36.52	36.23	98	1	VEST	REST1	40	50
	111	36.42	36.50	36.25	91	1	VEST	REST1	40	50
	112	36.42	36.47	36.28	96	1	VEST	REST1	40	50
	113	36.41	36.45	36.30	100	1	VEST	REST1	40	50
	114	36.43	36.47	36.33	95	1	VEST	REST1	40	50
	115	36.44	36.49	36.36	94	1	VEST	REST1	40	50
	116	36.44	36.48	36.37	97	1	VEST	REST1	40	50
	117	36.44	36.48	36.39	93	1	VEST	REST1	40	50
	118	36.44	36.49	36.41	89	1	VEST	REST1	40	50
	119	36.43	36.48	36.43	85	1	VEST	REST1	40	50
	120	36.43	36.47	36.45	88	1	VEST	REST1	40	50
20	121	36.43	36.50	36.47	88	1	VEST	REST1	40	50
	122	36.44	36.50	36.48	91	1	VEST	REST1	40	50
	123	36.44	36.48	36.50	80	1	VEST	REST1	40	50
	124	36.44	36.49	36.51	85	1	VEST	REST1	40	50
	125	36.44	36.50	36.52	86	1	VEST	REST1	40	50
	126	36.44	36.51	36.53	85	1	VEST	REST1	40	50
	127	36.45	36.51	36.55	86	1	VEST	REST1	40	50
	128	36.45	36.51	36.57	93	1	VEST	REST1	40	50
	129	36.44	36.51	36.58	90	1	VEST	REST1	40	50
	130	36.44	36.50	36.59	84	1	VEST	REST1	40	50
	131	36.44	36.50	36.61	88	1	VEST	REST1	40	50
	132	36.44	36.51	36.63	83	1	VEST	REST1	40	50
	133	36.45	36.52	36.65	91	1	VEST	REST1	40	50
	134	36.46	36.53	36.68	103	1	VEST	REST1	40	50
	135	36.45	36.51	36.69	96	1	VEST	REST1	40	50
	136	36.43	36.50	36.68	95	1	VEST	REST1	40	50
	137	36.44	36.51	36.69	99	1	VEST	REST1	40	50
	138	36.44	36.51	36.70	92	1	VEST	REST1	40	50
	139	36.43	36.50	36.72	100	1	VEST	EXERCISE1	40	50
	140	36.44	36.52	36.73	89	1	VEST	EXERCISE1	40	50
	141	36.44	36.56	36.75	89	1	VEST	EXERCISE1	40	50
	142	36.44	36.58	36.76	104	1	VEST	EXERCISE1	40	50
	143	36.45	36.54	36.78	106	1	VEST	EXERCISE1	40	50
	144	36.44	36.52	36.79	110	1	VEST	EXERCISE1	40	50
	145	36.43	36.52	36.79	113	1	VEST	EXERCISE1	40	50
	146	36.44	36.51	36.81	113	1	VEST	EXERCISE1	40	50
	147	36.44	36.49	36.83	114	1	VEST	EXERCISE1	40	50
	148	36.44	36.50	36.83	115	1	VEST	EXERCISE1	40	50
	149	36.44	36.49	36.85	114	1	VEST	EXERCISE1	40	50
	150	36.44	36.47	36.86	113	1	VEST	EXERCISE1	40	50
25	151	36.44	36.48	36.85	114	1	VEST	EXERCISE1	40	50

min	number	Tre	Tes	Tsk-head	HR	ID	condition	period	Ta	RH
	152	36.44	36.50	36.85	116	1	VEST	EXERCISE1	40	50
	153	36.45	36.51	36.86	114	1	VEST	EXERCISE1	40	50
	154	36.45	36.50	36.88	108	1	VEST	EXERCISE1	40	50
	155	36.45	36.51	36.89	116	1	VEST	EXERCISE1	40	50
	156	36.45	36.52	36.88	113	1	VEST	EXERCISE1	40	50
	157	36.45	36.52	36.87	113	1	VEST	EXERCISE1	40	50
	158	36.45	36.53	36.86	116	1	VEST	EXERCISE1	40	50
	159	36.46	36.55	36.87	114	1	VEST	EXERCISE1	40	50
	160	36.46	36.57	36.85	117	1	VEST	EXERCISE1	40	50
	161	36.45	36.57	36.85	117	1	VEST	EXERCISE1	40	50
	162	36.46	36.60	36.86	117	1	VEST	EXERCISE1	40	50
	163	36.47	36.62	36.86	119	1	VEST	EXERCISE1	40	50
	164	36.47	36.63	36.84	118	1	VEST	EXERCISE1	40	50
	165	36.47	36.64	36.84	117	1	VEST	EXERCISE1	40	50
	166	36.47	36.66	36.84	119	1	VEST	EXERCISE1	40	50
	167	36.47	36.68	36.83	120	1	VEST	EXERCISE1	40	50
	168	36.47	36.69	36.85	121	1	VEST	EXERCISE1	40	50
	169	36.48	36.71	36.83	119	1	VEST	EXERCISE1	40	50
	170	36.48	36.72	36.83	120	1	VEST	EXERCISE1	40	50
	171	36.49	36.73	36.84	122	1	VEST	EXERCISE1	40	50
	172	36.49	36.75	36.83	120	1	VEST	EXERCISE1	40	50
	173	36.49	36.78	36.81	121	1	VEST	EXERCISE1	40	50
	174	36.49	36.80	36.82	122	1	VEST	EXERCISE1	40	50
	175	36.50	36.79	36.83	121	1	VEST	EXERCISE1	40	50
	176	36.50	36.80	36.84	120	1	VEST	EXERCISE1	40	50
	177	36.50	36.81	36.83	122	1	VEST	EXERCISE1	40	50
	178	36.51	36.83	36.81	122	1	VEST	EXERCISE1	40	50
	179	36.51	36.85	36.81	121	1	VEST	EXERCISE1	40	50
	180	36.51	36.85	36.81	119	1	VEST	EXERCISE1	40	50
30	181	36.53	36.88	36.81	121	1	VEST	EXERCISE1	40	50
	182	36.54	36.90	36.81	120	1	VEST	EXERCISE1	40	50
	183	36.54	36.91	36.80	118	1	VEST	EXERCISE1	40	50
	184	36.54	36.92	36.80	118	1	VEST	EXERCISE1	40	50
	185	36.54	36.93	36.79	118	1	VEST	EXERCISE1	40	50
	186	36.55	36.95	36.79	123	1	VEST	EXERCISE1	40	50
	187	36.56	36.95	36.80	124	1	VEST	EXERCISE1	40	50
	188	36.56	36.97	36.80	122	1	VEST	EXERCISE1	40	50
	189	36.57	36.99	36.82	119	1	VEST	EXERCISE1	40	50
	190	36.57	36.99	36.82	122	1	VEST	EXERCISE1	40	50
	191	36.58	37.02	36.82	122	1	VEST	EXERCISE1	40	50
	192	36.59	37.03	36.83	120	1	VEST	EXERCISE1	40	50
	193	36.60	37.04	36.83	122	1	VEST	EXERCISE1	40	50
	194	36.60	37.05	36.83	124	1	VEST	EXERCISE1	40	50
	195	36.60	37.06	36.83	125	1	VEST	EXERCISE1	40	50
	196	36.61	37.06	36.84	125	1	VEST	EXERCISE1	40	50
	197	36.62	37.05	36.85	123	1	VEST	EXERCISE1	40	50

min	number	Tre	Tes	Tsk-head	HR	ID	condition	period	Ta	RH
	198	36.63	37.06	36.85	123	1	VEST	EXERCISE1	40	50
	199	36.64	37.09	36.83	120	1	VEST	EXERCISE1	40	50
	200	36.65	37.08	36.83	120	1	VEST	EXERCISE1	40	50
	201	36.65	37.07	36.84	121	1	VEST	EXERCISE1	40	50
	202	36.65	37.08	36.84	120	1	VEST	EXERCISE1	40	50
	203	36.66	37.09	36.84	120	1	VEST	EXERCISE1	40	50
	204	36.66	37.08	36.84	122	1	VEST	EXERCISE1	40	50
	205	36.66	37.07	36.84	125	1	VEST	EXERCISE1	40	50
	206	36.67	37.10	36.83	125	1	VEST	EXERCISE1	40	50
	207	36.68	37.11	36.83	122	1	VEST	EXERCISE1	40	50
	208	36.68	37.10	36.84	123	1	VEST	EXERCISE1	40	50
	209	36.67	37.10	36.83	124	1	VEST	EXERCISE1	40	50
	210	36.67	37.11	36.82	121	1	VEST	EXERCISE1	40	50
35	211	36.68	37.13	36.82	119	1	VEST	EXERCISE1	40	50
	212	36.69	37.14	36.82	125	1	VEST	EXERCISE1	40	50
	213	36.70	37.14	36.83	123	1	VEST	EXERCISE1	40	50
	214	36.70	37.13	36.84	120	1	VEST	EXERCISE1	40	50
	215	36.70	37.13	36.83	116	1	VEST	EXERCISE1	40	50
	216	36.70	37.13	36.84	126	1	VEST	EXERCISE1	40	50
	217	36.70	37.14	36.85	124	1	VEST	EXERCISE1	40	50
	218	36.70	37.14	36.85	123	1	VEST	EXERCISE1	40	50
	219	36.71	37.13	36.85	122	1	VEST	EXERCISE1	40	50
	220	36.71	37.14	36.84	122	1	VEST	EXERCISE1	40	50
	221	36.72	37.16	36.83	124	1	VEST	EXERCISE1	40	50
	222	36.73	37.17	36.84	124	1	VEST	EXERCISE1	40	50
	223	36.74	37.18	36.84	123	1	VEST	EXERCISE1	40	50
	224	36.74	37.19	36.84	124	1	VEST	EXERCISE1	40	50
	225	36.74	37.18	36.84	123	1	VEST	EXERCISE1	40	50
	226	36.75	37.18	36.84	121	1	VEST	EXERCISE1	40	50
	227	36.75	37.17	36.84	124	1	VEST	EXERCISE1	40	50
	228	36.76	37.17	36.86	125	1	VEST	EXERCISE1	40	50
	229	36.76	37.17	36.86	126	1	VEST	EXERCISE1	40	50
	230	36.75	37.18	36.85	123	1	VEST	EXERCISE1	40	50
	231	36.76	37.18	36.86	121	1	VEST	EXERCISE1	40	50
	232	36.76	37.18	36.88	122	1	VEST	EXERCISE1	40	50
	233	36.77	37.21	36.89	124	1	VEST	EXERCISE1	40	50
	234	36.78	37.22	36.88	127	1	VEST	EXERCISE1	40	50
	235	36.79	37.21	36.88	125	1	VEST	EXERCISE1	40	50
	236	36.78	37.21	36.89	126	1	VEST	EXERCISE1	40	50
	237	36.79	37.22	36.90	125	1	VEST	EXERCISE1	40	50
	238	36.80	37.24	36.90	126	1	VEST	EXERCISE1	40	50
	239	36.81	37.25	36.91	126	1	VEST	EXERCISE1	40	50
	240	36.80	37.24	36.90	124	1	VEST	EXERCISE1	40	50
40	241	36.81	37.24	36.91	125	1	VEST	EXERCISE1	40	50
	242	36.82	37.25	36.92	124	1	VEST	EXERCISE1	40	50
	243	36.82	37.25	36.93	127	1	VEST	EXERCISE1	40	50

min	number	Tre	Tes	Tsk-head	HR	ID	condition	period	Ta	RH
	244	36.82	37.26	36.94	126	1	VEST	EXERCISE1	40	50
	245	36.82	37.28	36.94	126	1	VEST	EXERCISE1	40	50
	246	36.83	37.30	36.95	126	1	VEST	EXERCISE1	40	50
	247	36.84	37.30	36.96	126	1	VEST	EXERCISE1	40	50
	248	36.84	37.31	36.97	125	1	VEST	EXERCISE1	40	50
	249	36.84	37.32	36.98	123	1	VEST	EXERCISE1	40	50
	250	36.85	37.32	36.98	123	1	VEST	EXERCISE1	40	50
	251	36.86	37.34	36.97	126	1	VEST	EXERCISE1	40	50
	252	36.87	37.35	36.97	127	1	VEST	EXERCISE1	40	50
	253	36.87	37.36	36.96	128	1	VEST	EXERCISE1	40	50
	254	36.88	37.36	36.95	127	1	VEST	EXERCISE1	40	50
	255	36.88	37.36	36.96	128	1	VEST	EXERCISE1	40	50
	256	36.89	37.36	36.96	128	1	VEST	EXERCISE1	40	50
	257	36.90	37.37	36.96	129	1	VEST	EXERCISE1	40	50
	258	36.91	37.39	36.95	127	1	VEST	EXERCISE1	40	50
	259	36.91	37.39	36.94	126	1	VEST	EXERCISE1	40	50
	260	36.90	37.38	36.93	130	1	VEST	EXERCISE1	40	50
	261	36.90	37.37	36.91	127	1	VEST	EXERCISE1	40	50
	262	36.90	37.37	36.90	127	1	VEST	EXERCISE1	40	50
	263	36.92	37.38	36.90	128	1	VEST	EXERCISE1	40	50
	264	36.92	37.39	36.91	130	1	VEST	EXERCISE1	40	50
	265	36.93	37.39	36.92	127	1	VEST	EXERCISE1	40	50
	266	36.94	37.40	36.93	126	1	VEST	EXERCISE1	40	50
	267	36.94	37.39	36.94	127	1	VEST	EXERCISE1	40	50
	268	36.94	37.40	36.96	128	1	VEST	EXERCISE1	40	50
	269	36.95	37.40	36.96	130	1	VEST	EXERCISE1	40	50
	270	36.96	37.38	36.97	130	1	VEST	EXERCISE1	40	50
45	271	36.96	37.38	36.98	130	1	VEST	EXERCISE1	40	50
	272	36.96	37.36	36.98	131	1	VEST	EXERCISE1	40	50
	273	36.96	37.37	36.98	130	1	VEST	EXERCISE1	40	50
	274	36.97	37.17	36.97	134	1	VEST	EXERCISE1	40	50
	275	36.98	37.12	36.96	129	1	VEST	EXERCISE1	40	50
	276	36.99	37.29	36.95	126	1	VEST	EXERCISE1	40	50
	277	37.00	37.30	36.94	125	1	VEST	EXERCISE1	40	50
	278	37.01	37.33	36.92	125	1	VEST	EXERCISE1	40	50
	279	37.01	37.36	36.92	126	1	VEST	EXERCISE1	40	50
	280	37.02	37.36	36.93	127	1	VEST	EXERCISE1	40	50
	281	37.02	37.33	36.95	129	1	VEST	EXERCISE1	40	50
	282	37.03	37.32	36.96	131	1	VEST	EXERCISE1	40	50
	283	37.03	37.33	36.97	130	1	VEST	EXERCISE1	40	50
	284	37.03	37.38	36.97	128	1	VEST	EXERCISE1	40	50
	285	37.04	37.43	36.97	125	1	VEST	EXERCISE1	40	50
	286	37.04	37.42	36.97	132	1	VEST	EXERCISE1	40	50
	287	37.04	37.38	36.96	130	1	VEST	EXERCISE1	40	50
	288	37.04	37.37	36.96	131	1	VEST	EXERCISE1	40	50
	289	37.04	37.40	36.95	129	1	VEST	EXERCISE1	40	50

min	number	Tre	Tes	Tsk-head	HR	ID	condition	period	Ta	RH
	290	37.04	37.40	36.96	122	1	VEST	EXERCISE1	40	50
	291	37.04	37.39	36.98	126	1	VEST	EXERCISE1	40	50
	292	37.05	37.40	36.98	126	1	VEST	EXERCISE1	40	50
	293	37.06	37.42	36.98	130	1	VEST	EXERCISE1	40	50
	294	37.07	37.43	36.98	132	1	VEST	EXERCISE1	40	50
	295	37.06	37.43	36.97	129	1	VEST	EXERCISE1	40	50
	296	37.06	37.42	36.97	130	1	VEST	EXERCISE1	40	50
	297	37.07	37.43	36.99	129	1	VEST	EXERCISE1	40	50
	298	37.08	37.45	36.99	128	1	VEST	EXERCISE1	40	50
	299	37.08	37.41	36.99	128	1	VEST	EXERCISE1	40	50
	300	37.08	37.39	37.00	123	1	VEST	EXERCISE1	40	50
50	301	37.08	37.41	37.00	128	1	VEST	EXERCISE1	40	50
	302	37.09	37.42	37.00	130	1	VEST	EXERCISE1	40	50
	303	37.09	37.41	36.99	132	1	VEST	EXERCISE1	40	50
	304	37.09	37.40	36.99	132	1	VEST	EXERCISE1	40	50
	305	37.10	37.40	36.99	131	1	VEST	EXERCISE1	40	50
	306	37.10	37.41	37.00	131	1	VEST	EXERCISE1	40	50
	307	37.10	37.43	37.00	131	1	VEST	EXERCISE1	40	50
	308	37.09	37.45	36.99	131	1	VEST	EXERCISE1	40	50
	309	37.10	37.44	37.00	131	1	VEST	EXERCISE1	40	50
	310	37.10	37.44	37.00	129	1	VEST	EXERCISE1	40	50
	311	37.11	37.45	37.00	132	1	VEST	EXERCISE1	40	50
	312	37.12	37.44	37.01	134	1	VEST	EXERCISE1	40	50
	313	37.14	37.48	37.01	134	1	VEST	EXERCISE1	40	50
	314	37.15	37.47	37.01	134	1	VEST	EXERCISE1	40	50
	315	37.15	37.45	37.01	133	1	VEST	EXERCISE1	40	50
	316	37.15	37.45	37.02	132	1	VEST	EXERCISE1	40	50
	317	37.15	37.46	37.01	133	1	VEST	EXERCISE1	40	50
	318	37.15	37.46	37.00	133	1	VEST	EXERCISE1	40	50
	319	37.15	37.45	37.00	134	1	VEST	EXERCISE1	40	50
	320	37.16	37.48	37.00	133	1	VEST	REST2	28	50
	321	37.17	37.50	36.87	130	1	VEST	REST2	28	50
	322	37.17	37.51	36.63	124	1	VEST	REST2	28	50
	323	37.18	37.51	36.40	113	1	VEST	REST2	28	50
	324	37.19	37.51	36.26	122	1	VEST	REST2	28	50
	325	37.19	37.51	36.24	102	1	VEST	REST2	28	50
	326	37.20	37.52	36.21	107	1	VEST	REST2	28	50
	327	37.20	37.40	36.12	111	1	VEST	REST2	28	50
	328	37.20	37.34	36.10	105	1	VEST	REST2	28	50
	329	37.20	37.39	36.12	105	1	VEST	REST2	28	50
	330	37.21	37.39	36.13	106	1	VEST	REST2	28	50
55	331	37.22	37.40	36.10	107	1	VEST	REST2	28	50
	332	37.23	37.40	36.01	103	1	VEST	REST2	28	50
	333	37.23	37.39	35.95	101	1	VEST	REST2	28	50
	334	37.24	37.37	35.93	104	1	VEST	REST2	28	50
	335	37.24	37.38	35.86	103	1	VEST	REST2	28	50

min	number	Tre	Tes	Tsk-head	HR	ID	condition	period	Ta	RH
	336	37.24	37.42	35.82	106	1	VEST	REST2	28	50
	337	37.23	37.38	35.85	106	1	VEST	REST2	28	50
	338	37.23	31.86	35.88	113	1	VEST	REST2	28	50
	339	37.23	26.82	35.87	120	1	VEST	REST2	28	50
	340	37.23	29.41	35.81	114	1	VEST	REST2	28	50
	341	37.23	31.11	35.73	111	1	VEST	REST2	28	50
	342	37.22	31.31	35.70	104	1	VEST	REST2	28	50
	343	37.22	32.21	35.74	103	1	VEST	REST2	28	50
	344	37.23	29.38	35.77	109	1	VEST	REST2	28	50
	345	37.22	28.74	35.76	98	1	VEST	REST2	28	50
	346	37.21	31.58	35.74	99	1	VEST	REST2	28	50
	347	37.22	32.23	35.69	101	1	VEST	REST2	28	50
	348	37.22	32.71	35.67	100	1	VEST	REST2	28	50
	349	37.22	33.33	35.69	92	1	VEST	REST2	28	50
	350	37.23	33.79	35.69	92	1	VEST	REST2	28	50
	351	37.23	33.98	35.68	96	1	VEST	REST2	28	50
	352	37.23	34.24	35.65	93	1	VEST	REST2	28	50
	353	37.23	34.55	35.61	95	1	VEST	REST2	28	50
	354	37.23	34.80	35.61	95	1	VEST	REST2	28	50
	355	37.22	34.98	35.59	91	1	VEST	REST2	28	50
	356	37.22	35.16	35.55	92	1	VEST	REST2	28	50
	357	37.23	35.33	35.54	93	1	VEST	REST2	28	50
	358	37.24	35.49	35.54	91	1	VEST	REST2	28	50
	359	37.24	35.62	35.50	88	1	VEST	REST2	28	50
	360	37.25	35.73	35.46	89	1	VEST	REST2	28	50
60	361	37.24	35.84	35.45	90	1	VEST	REST2	28	50
	362	37.24	35.95	35.44	90	1	VEST	REST2	28	50
	363	37.24	36.07	35.44	92	1	VEST	REST2	28	50
	364	37.23	36.16	35.42	94	1	VEST	REST2	28	50
	365	37.22	36.30	35.39	98	1	VEST	REST2	28	50
	366	37.22	33.14	35.40	111	1	VEST	REST2	28	50
	367	37.23	30.27	35.44	98	1	VEST	REST2	28	50
	368	37.24	31.34	35.44	93	1	VEST	REST2	28	50
	369	37.23	32.28	35.40	87	1	VEST	REST2	28	50
	370	37.23	32.82	35.38	89	1	VEST	REST2	28	50
	371	37.23	33.18	35.37	80	1	VEST	REST2	28	50
	372	37.23	33.45	35.33	83	1	VEST	REST2	28	50
	373	37.23	32.89	35.32	93	1	VEST	REST2	28	50
	374	37.23	29.42	35.36	98	1	VEST	REST2	28	50
	375	37.23	28.58	35.39	99	1	VEST	REST2	28	50
	376	37.24	31.00	35.37	87	1	VEST	REST2	28	50
	377	37.23	32.03	35.33	96	1	VEST	REST2	28	50
	378	37.23	30.93	35.33	96	1	VEST	REST2	28	50
	379	37.23	28.59	35.35	95	1	VEST	REST2	28	50
	380	37.23	29.87	35.34	96	1	VEST	REST2	28	50
	381	37.23	29.98	35.32	102	1	VEST	REST2	28	50

min	number	Tre	Tes	Tsk-head	HR	ID	condition	period	Ta	RH
	382	37.23	29.80	35.31	94	1	VEST	REST2	28	50
	383	37.23	31.94	35.28	94	1	VEST	REST2	28	50
	384	37.22	32.81	35.27	92	1	VEST	REST2	28	50
	385	37.22	33.34	35.29	85	1	VEST	REST2	28	50
	386	37.22	33.59	35.27	86	1	VEST	REST2	28	50
	387	37.22	30.55	35.22	98	1	VEST	REST2	28	50
	388	37.23	30.09	35.20	97	1	VEST	REST2	28	50
	389	37.23	33.17	35.17	88	1	VEST	REST2	28	50
	390	37.22	33.67	35.17	96	1	VEST	REST2	28	50
65	391	37.22	34.21	35.17	76	1	VEST	REST2	28	50
	392	37.22	34.68	35.15	82	1	VEST	REST2	28	50
	393	37.22	34.92	35.15	85	1	VEST	REST2	28	50
	394	37.22	31.18	35.18	96	1	VEST	REST2	28	50
	395	37.22	29.90	35.15	92	1	VEST	REST2	28	50
	396	37.22	32.86	35.11	82	1	VEST	REST2	28	50
	397	37.22	33.48	35.15	90	1	VEST	REST2	28	50
	398	37.21	34.01	35.18	94	1	VEST	REST2	28	50
	399	37.21	32.06	35.15	97	1	VEST	REST2	28	50
	400	37.23	31.28	35.15	98	1	VEST	REST2	28	50
	401	37.24	33.23	35.17	88	1	VEST	REST2	28	50
	402	37.23	33.84	35.16	81	1	VEST	REST2	28	50
	403	37.23	34.18	35.18	80	1	VEST	REST2	28	50
	404	37.23	34.45	35.18	82	1	VEST	REST2	28	50
	405	37.23	34.70	35.16	77	1	VEST	REST2	28	50
	406	37.22	35.08	35.16	79	1	VEST	REST2	28	50
	407	37.22	35.31	35.15	81	1	VEST	REST2	28	50
	408	37.21	35.33	35.15	82	1	VEST	REST2	28	50
	409	37.22	35.40	35.13	84	1	VEST	REST2	28	50
	410	37.22	35.52	35.12	85	1	VEST	REST2	28	50
	411	37.22	35.68	35.13	82	1	VEST	REST2	28	50
	412	37.22	35.99	35.07	100	1	VEST	REST2	28	50
	413	37.23	36.10	35.08	103	1	VEST	REST2	28	50
	414	37.22	36.30	35.09	78	1	VEST	REST2	28	50
	415	37.22	36.53	35.08	88	1	VEST	REST2	28	50
	416	37.21	36.57	35.09	89	1	VEST	REST2	28	50
	417	37.21	36.57	35.08	93	1	VEST	REST2	28	50
	418	37.21	36.62	35.09	86	1	VEST	REST2	28	50
	419	37.20	36.71	35.11	85	1	VEST	REST2	28	50
	420	37.20	36.76	35.11	90	1	VEST	REST2	28	50
70	421	37.21	36.78	35.10	90	1	VEST	REST2	28	50
	422	37.21	36.80	35.10	83	1	VEST	REST2	28	50
	423	37.20	36.83	35.06	90	1	VEST	REST2	28	50
	424	37.20	36.88	35.05	92	1	VEST	REST2	28	50
	425	37.20	36.92	35.10	77	1	VEST	REST2	28	50
	426	37.19	36.92	35.10	86	1	VEST	REST2	28	50
	427	37.18	36.92	35.09	83	1	VEST	REST2	28	50

min	number	Tre	Tes	Tsk-head	HR	ID	condition	period	Ta	RH
	428	37.19	36.94	35.09	83	1	VEST	REST2	28	50
	429	37.19	36.95	35.07	82	1	VEST	REST2	28	50
	430	37.18	36.95	35.04	87	1	VEST	REST2	28	50
	431	37.18	36.97	35.04	93	1	VEST	REST2	28	50
	432	37.19	37.01	35.06	102	1	VEST	REST2	28	50
	433	37.18	37.03	35.07	98	1	VEST	REST2	28	50
	434	37.18	37.01	35.09	92	1	VEST	REST2	28	50
	435	37.18	36.69	35.13	90	1	VEST	REST2	28	50
	436	37.18	36.70	35.16	98	1	VEST	REST2	28	50
	437	37.17	37.02	35.20	87	1	VEST	REST2	28	50
	438	37.17	37.02	35.24	85	1	VEST	REST2	28	50
	439	37.17	37.02	35.28	85	1	VEST	REST2	40	50
	440	37.17	37.02	35.44	101	1	VEST	REST2	40	50
	441	37.18	37.01	35.70	99	1	VEST	REST2	40	50
	442	37.18	37.00	35.88	99	1	VEST	REST2	40	50
	443	37.18	37.01	36.03	98	1	VEST	REST2	40	50
	444	37.17	37.01	36.17	88	1	VEST	REST2	40	50
	445	37.17	36.99	36.25	86	1	VEST	REST2	40	50
	446	37.18	36.99	36.32	87	1	VEST	REST2	40	50
	447	37.18	36.99	36.38	86	1	VEST	REST2	40	50
	448	37.17	37.00	36.45	88	1	VEST	REST2	40	50
	449	37.17	37.01	36.50	86	1	VEST	REST2	40	50
	450	37.18	37.00	36.55	85	1	VEST	REST2	40	50
75	451	37.18	36.98	36.59	86	1	VEST	REST2	40	50
	452	37.19	36.99	36.60	88	1	VEST	REST2	40	50
	453	37.19	36.99	36.61	91	1	VEST	REST2	40	50
	454	37.19	36.97	36.64	91	1	VEST	REST2	40	50
	455	37.19	36.97	36.66	89	1	VEST	REST2	40	50
	456	37.18	36.98	36.67	85	1	VEST	REST2	40	50
	457	37.18	36.96	36.68	91	1	VEST	REST2	40	50
	458	37.18	36.96	36.69	103	1	VEST	REST2	40	50
	459	37.18	36.97	36.68	105	1	VEST	REST2	40	50
	460	37.18	36.96	36.68	101	1	VEST	REST2	40	50
	461	37.17	36.95	36.70	103	1	VEST	REST2	40	50
	462	37.16	36.93	36.71	102	1	VEST	REST2	40	50
	463	37.16	36.93	36.73	101	1	VEST	EXERCISE2	40	50
	464	37.16	36.95	36.73	91	1	VEST	EXERCISE2	40	50
	465	37.15	36.95	36.74	105	1	VEST	EXERCISE2	40	50
	466	37.16	36.95	36.75	109	1	VEST	EXERCISE2	40	50
	467	37.16	36.97	36.75	111	1	VEST	EXERCISE2	40	50
	468	37.15	36.97	36.76	111	1	VEST	EXERCISE2	40	50
	469	37.15	36.97	36.77	113	1	VEST	EXERCISE2	40	50
	470	37.15	36.98	36.76	117	1	VEST	EXERCISE2	40	50
	471	37.16	37.00	36.77	117	1	VEST	EXERCISE2	40	50
	472	37.17	37.01	36.78	115	1	VEST	EXERCISE2	40	50
	473	37.17	37.02	36.79	114	1	VEST	EXERCISE2	40	50

min	number	Tre	Tes	Tsk-head	HR	ID	condition	period	Ta	RH
	474	37.17	37.03	36.80	114	1	VEST	EXERCISE2	40	50
	475	37.17	36.98	36.82	112	1	VEST	EXERCISE2	40	50
	476	37.17	36.98	36.83	120	1	VEST	EXERCISE2	40	50
	477	37.16	37.05	36.85	118	1	VEST	EXERCISE2	40	50
	478	37.15	37.08	36.88	120	1	VEST	EXERCISE2	40	50
	479	37.15	37.12	36.89	120	1	VEST	EXERCISE2	40	50
	480	37.16	37.15	36.90	120	1	VEST	EXERCISE2	40	50
80	481	37.16	37.16	36.91	120	1	VEST	EXERCISE2	40	50
	482	37.16	37.16	36.93	122	1	VEST	EXERCISE2	40	50
	483	37.16	37.17	36.95	120	1	VEST	EXERCISE2	40	50
	484	37.16	37.18	36.97	118	1	VEST	EXERCISE2	40	50
	485	37.16	37.19	36.97	118	1	VEST	EXERCISE2	40	50
	486	37.16	37.20	36.98	117	1	VEST	EXERCISE2	40	50
	487	37.16	37.21	36.98	118	1	VEST	EXERCISE2	40	50
	488	37.16	37.22	36.98	120	1	VEST	EXERCISE2	40	50
	489	37.17	37.23	37.00	123	1	VEST	EXERCISE2	40	50
	490	37.18	37.24	37.02	122	1	VEST	EXERCISE2	40	50
	491	37.17	37.25	37.03	120	1	VEST	EXERCISE2	40	50
	492	37.18	37.26	37.04	119	1	VEST	EXERCISE2	40	50
	493	37.19	37.27	37.05	121	1	VEST	EXERCISE2	40	50
	494	37.19	37.29	37.05	123	1	VEST	EXERCISE2	40	50
	495	37.19	37.31	37.05	123	1	VEST	EXERCISE2	40	50
	496	37.19	37.30	37.06	122	1	VEST	EXERCISE2	40	50
	497	37.20	37.30	37.07	122	1	VEST	EXERCISE2	40	50
	498	37.21	37.30	37.09	125	1	VEST	EXERCISE2	40	50
	499	37.21	37.31	37.10	127	1	VEST	EXERCISE2	40	50
	500	37.21	37.33	37.11	129	1	VEST	EXERCISE2	40	50
	501	37.22	37.35	37.13	127	1	VEST	EXERCISE2	40	50
	502	37.23	37.36	37.15	127	1	VEST	EXERCISE2	40	50
	503	37.22	37.36	37.16	126	1	VEST	EXERCISE2	40	50
	504	37.22	37.36	37.16	127	1	VEST	EXERCISE2	40	50
	505	37.22	37.36	37.17	123	1	VEST	EXERCISE2	40	50
	506	37.22	37.37	37.18	123	1	VEST	EXERCISE2	40	50
	507	37.23	37.39	37.17	124	1	VEST	EXERCISE2	40	50
	508	37.24	37.39	37.18	124	1	VEST	EXERCISE2	40	50
	509	37.24	37.38	37.19	124	1	VEST	EXERCISE2	40	50
	510	37.24	37.38	37.19	123	1	VEST	EXERCISE2	40	50
85	511	37.24	37.38	37.19	123	1	VEST	EXERCISE2	40	50
	512	37.23	37.38	37.18	124	1	VEST	EXERCISE2	40	50
	513	37.24	37.39	37.16	125	1	VEST	EXERCISE2	40	50
	514	37.25	37.39	37.16	126	1	VEST	EXERCISE2	40	50
	515	37.26	37.38	37.17	128	1	VEST	EXERCISE2	40	50
	516	37.26	37.36	37.17	124	1	VEST	EXERCISE2	40	50
	517	37.26	37.36	37.17	124	1	VEST	EXERCISE2	40	50
	518	37.27	37.37	37.17	124	1	VEST	EXERCISE2	40	50
	519	37.27	37.36	37.16	124	1	VEST	EXERCISE2	40	50

min	number	Tre	Tes	Tsk-head	HR	ID	condition	period	Ta	RH
	520	37.28	37.38	37.16	125	1	VEST	EXERCISE2	40	50
	521	37.29	37.38	37.15	123	1	VEST	EXERCISE2	40	50
	522	37.29	37.38	37.15	126	1	VEST	EXERCISE2	40	50
	523	37.28	37.41	37.14	128	1	VEST	EXERCISE2	40	50
	524	37.28	37.34	37.13	127	1	VEST	EXERCISE2	40	50
	525	37.28	37.14	37.15	124	1	VEST	EXERCISE2	40	50
	526	37.29	37.17	37.14	129	1	VEST	EXERCISE2	40	50
	527	37.30	37.32	37.14	129	1	VEST	EXERCISE2	40	50
	528	37.30	37.33	37.14	129	1	VEST	EXERCISE2	40	50
	529	37.31	37.35	37.13	127	1	VEST	EXERCISE2	40	50
	530	37.31	37.36	37.13	127	1	VEST	EXERCISE2	40	50
	531	37.31	37.37	37.15	127	1	VEST	EXERCISE2	40	50
	532	37.32	37.37	37.16	129	1	VEST	EXERCISE2	40	50
	533	37.32	37.36	37.18	130	1	VEST	EXERCISE2	40	50
	534	37.31	37.36	37.18	129	1	VEST	EXERCISE2	40	50
	535	37.31	37.36	37.17	128	1	VEST	EXERCISE2	40	50
	536	37.32	37.37	37.18	127	1	VEST	EXERCISE2	40	50
	537	37.32	37.37	37.17	128	1	VEST	EXERCISE2	40	50
	538	37.32	37.37	37.16	128	1	VEST	EXERCISE2	40	50
	539	37.33	37.39	37.16	128	1	VEST	EXERCISE2	40	50
	540	37.34	37.39	37.17	125	1	VEST	EXERCISE2	40	50
90	541	37.34	37.40	37.17	124	1	VEST	EXERCISE2	40	50
	542	37.34	37.41	37.18	124	1	VEST	EXERCISE2	40	50
	543	37.34	37.41	37.20	127	1	VEST	EXERCISE2	40	50
	544	37.33	37.42	37.21	127	1	VEST	EXERCISE2	40	50
	545	37.33	37.43	37.22	128	1	VEST	EXERCISE2	40	50
	546	37.34	37.44	37.22	127	1	VEST	EXERCISE2	40	50
	547	37.35	37.44	37.22	127	1	VEST	EXERCISE2	40	50
	548	37.35	37.43	37.22	129	1	VEST	EXERCISE2	40	50
	549	37.35	37.44	37.23	131	1	VEST	EXERCISE2	40	50
	550	37.35	37.44	37.25	131	1	VEST	EXERCISE2	40	50
	551	37.35	37.44	37.23	132	1	VEST	EXERCISE2	40	50
	552	37.35	37.44	37.21	129	1	VEST	EXERCISE2	40	50
	553	37.35	37.43	37.21	129	1	VEST	EXERCISE2	40	50
	554	37.36	37.44	37.23	129	1	VEST	EXERCISE2	40	50
	555	37.36	37.45	37.23	130	1	VEST	EXERCISE2	40	50
	556	37.37	37.43	37.22	131	1	VEST	EXERCISE2	40	50
	557	37.37	37.42	37.21	130	1	VEST	EXERCISE2	40	50
	558	37.37	37.44	37.21	129	1	VEST	EXERCISE2	40	50
	559	37.37	37.43	37.22	130	1	VEST	EXERCISE2	40	50
	560	37.38	37.42	37.22	128	1	VEST	EXERCISE2	40	50
	561	37.39	37.43	37.23	128	1	VEST	EXERCISE2	40	50
	562	37.39	37.44	37.23	129	1	VEST	EXERCISE2	40	50
	563	37.38	37.43	37.23	129	1	VEST	EXERCISE2	40	50
	564	37.39	37.44	37.24	129	1	VEST	EXERCISE2	40	50
	565	37.40	37.45	37.25	130	1	VEST	EXERCISE2	40	50

min	number	Tre	Tes	Tsk-head	HR	ID	condition	period	Ta	RH
	566	37.40	37.44	37.25	129	1	VEST	EXERCISE2	40	50
	567	37.40	37.44	37.24	129	1	VEST	EXERCISE2	40	50
	568	37.40	37.44	37.23	128	1	VEST	EXERCISE2	40	50
	569	37.40	37.44	37.22	128	1	VEST	EXERCISE2	40	50
	570	37.40	37.45	37.23	129	1	VEST	EXERCISE2	40	50
95	571	37.40	37.45	37.23	130	1	VEST	EXERCISE2	40	50
	572	37.41	37.45	37.24	129	1	VEST	EXERCISE2	40	50
	573	37.41	37.45	37.24	130	1	VEST	EXERCISE2	40	50
	574	37.41	37.45	37.23	129	1	VEST	EXERCISE2	40	50
	575	37.40	37.45	37.23	128	1	VEST	EXERCISE2	40	50
	576	37.41	37.44	37.23	128	1	VEST	EXERCISE2	40	50
	577	37.41	37.45	37.23	130	1	VEST	EXERCISE2	40	50
	578	37.42	37.46	37.21	133	1	VEST	EXERCISE2	40	50
	579	37.42	37.45	37.21	134	1	VEST	EXERCISE2	40	50
	580	37.42	37.45	37.21	133	1	VEST	EXERCISE2	40	50
	581	37.42	37.45	37.20	134	1	VEST	EXERCISE2	40	50
	582	37.41	37.46	37.20	135	1	VEST	EXERCISE2	40	50
	583	37.42	37.47	37.21	134	1	VEST	EXERCISE2	40	50
	584	37.42	37.47	37.21	133	1	VEST	EXERCISE2	40	50
	585	37.42	37.46	37.21	131	1	VEST	EXERCISE2	40	50
	586	37.42	37.46	37.21	131	1	VEST	EXERCISE2	40	50
	587	37.42	37.45	37.21	133	1	VEST	EXERCISE2	40	50
	588	37.42	37.44	37.20	133	1	VEST	EXERCISE2	40	50
	589	37.43	37.45	37.19	134	1	VEST	EXERCISE2	40	50
	590	37.43	37.43	37.21	134	1	VEST	EXERCISE2	40	50
	591	37.43	37.43	37.22	133	1	VEST	EXERCISE2	40	50
	592	37.43	37.43	37.22	132	1	VEST	EXERCISE2	40	50
	593	37.43	37.44	37.21	127	1	VEST	EXERCISE2	40	50
	594	37.44	37.44	37.19	133	1	VEST	EXERCISE2	40	50
	595	37.44	37.44	37.19	132	1	VEST	EXERCISE2	40	50
	596	37.45	37.44	37.21	133	1	VEST	EXERCISE2	40	50
	597	37.45	37.44	37.23	133	1	VEST	EXERCISE2	40	50
	598	37.44	37.44	37.24	133	1	VEST	EXERCISE2	40	50
	599	37.44	37.43	37.23	136	1	VEST	EXERCISE2	40	50
	600	37.44	37.44	37.23	136	1	VEST	EXERCISE2	40	50
100	601	37.45	37.44	37.24	135	1	VEST	EXERCISE2	40	50
	602	37.44	37.44	37.24	134	1	VEST	EXERCISE2	40	50
	603	37.44	37.44	37.25	133	1	VEST	EXERCISE2	40	50
	604	37.45	37.45	37.25	134	1	VEST	EXERCISE2	40	50
	605	37.46	37.45	37.23	135	1	VEST	EXERCISE2	40	50
	606	37.46	37.44	37.21	133	1	VEST	EXERCISE2	40	50
	607	37.46	37.45	37.22	135	1	VEST	EXERCISE2	40	50
	608	37.46	37.45	37.22	132	1	VEST	EXERCISE2	40	50
	609	37.46	37.45	37.21	133	1	VEST	EXERCISE2	40	50
	610	37.46	37.33	37.21	139	1	VEST	EXERCISE2	40	50
	611	37.46	37.26	37.22	134	1	VEST	EXERCISE2	40	50

min	number	Tre	Tes	Tsk-head	HR	ID	condition	period	Ta	RH
	612	37.47	37.34	37.22	136	1	VEST	EXERCISE2	40	50
	613	37.48	37.39	37.24	134	1	VEST	EXERCISE2	40	50
	614	37.48	37.40	37.25	133	1	VEST	EXERCISE2	40	50
	615	37.48	37.41	37.25	133	1	VEST	EXERCISE2	40	50
	616	37.48	37.42	37.24	134	1	VEST	EXERCISE2	40	50
	617	37.47	37.43	37.24	134	1	VEST	EXERCISE2	40	50
	618	37.47	37.44	37.24	134	1	VEST	EXERCISE2	40	50
	619	37.48	37.45	37.24	133	1	VEST	EXERCISE2	40	50
	620	37.48	37.45	37.25	133	1	VEST	EXERCISE2	40	50
	621	37.49	37.46	37.25	133	1	VEST	EXERCISE2	40	50
	622	37.49	37.47	37.25	132	1	VEST	EXERCISE2	40	50
	623	37.49	37.47	37.25	134	1	VEST	EXERCISE2	40	50
	624	37.49	37.47	37.25	134	1	VEST	EXERCISE2	40	50
	625	37.49	37.47	37.26	135	1	VEST	EXERCISE2	40	50
	626	37.49	37.48	37.27	134	1	VEST	EXERCISE2	40	50
	627	37.49	37.49	37.28	134	1	VEST	EXERCISE2	40	50
	628	37.49	37.49	37.27	134	1	VEST	EXERCISE2	40	50
	629	37.50	37.50	37.27	134	1	VEST	EXERCISE2	40	50
	630	37.50	37.49	37.26	134	1	VEST	EXERCISE2	40	50
105	631	37.50	37.49	37.26	134	1	VEST	EXERCISE2	40	50
	632	37.50	37.49	37.27	134	1	VEST	EXERCISE2	40	50
	633	37.50	37.48	37.27	135	1	VEST	EXERCISE2	40	50
	634	37.51	37.48	37.26	134	1	VEST	EXERCISE2	40	50
	635	37.52	37.51	37.26	136	1	VEST	EXERCISE2	40	50
	636	37.52	37.51	37.27	136	1	VEST	EXERCISE2	40	50
	637	37.52	37.51	37.27	137	1	VEST	EXERCISE2	40	50
	638	37.53	37.51	37.26	137	1	VEST	EXERCISE2	40	50
	639	37.53	37.56	37.25	134	1	VEST	EXERCISE2	40	50
	640	37.53	37.56	37.26	137	1	VEST	EXERCISE2	40	50
	641	37.54	37.51	37.26	137	1	VEST	EXERCISE2	40	50
	642	37.53	37.50	37.26	137	1	VEST	EXERCISE2	40	50
	643	37.52	37.50	37.26	136	1	VEST	EXERCISE2	40	50
	644	37.53	37.49	37.25	135	1	VEST	REST3	28	50
	645	37.52	37.49	37.01	136	1	VEST	REST3	28	50
	646	37.51	37.53	36.65	127	1	VEST	REST3	28	50
	647	37.52	37.52	36.50	121	1	VEST	REST3	28	50
	648	37.53	37.46	36.46	126	1	VEST	REST3	28	50
	649	37.53	37.45	36.44	111	1	VEST	REST3	28	50
	650	37.53	37.47	36.40	120	1	VEST	REST3	28	50
	651	37.52	37.46	36.34	119	1	VEST	REST3	28	50
	652	37.52	37.45	36.25	117	1	VEST	REST3	28	50
	653	37.53	37.45	36.21	113	1	VEST	REST3	28	50
	654	37.53	37.44	36.25	113	1	VEST	REST3	28	50
	655	37.53	37.42	36.23	112	1	VEST	REST3	28	50
	656	37.54	37.38	36.18	108	1	VEST	REST3	28	50
	657	37.55	37.35	36.11	108	1	VEST	REST3	28	50

min	number	Tre	Tes	Tsk-head	HR	ID	condition	period	Ta	RH
	658	37.55	37.32	35.98	105	1	VEST	REST3	28	50
	659	37.55	37.29	35.90	105	1	VEST	REST3	28	50
	660	37.54	37.26	35.89	106	1	VEST	REST3	28	50
110	661	37.53	37.22	35.83	105	1	VEST	REST3	28	50
	662	37.51	37.19	35.81	105	1	VEST	REST3	28	50
	663	37.51	37.17	35.82	104	1	VEST	REST3	28	50
	664	37.52	37.16	35.79	106	1	VEST	REST3	28	50
	665	37.51	37.14	35.72	106	1	VEST	REST3	28	50
	666	37.50	37.12	35.65	106	1	VEST	REST3	28	50
	667	37.50	37.08	35.64	101	1	VEST	REST3	28	50
	668	37.49	37.07	35.65	100	1	VEST	REST3	28	50
	669	37.49	37.06	35.67	100	1	VEST	REST3	28	50
	670	37.49	37.03	35.67	100	1	VEST	REST3	28	50
	671	37.49	37.02	35.63	101	1	VEST	REST3	28	50
	672	37.49	37.03	35.57	99	1	VEST	REST3	28	50
	673	37.49	37.03	35.53	96	1	VEST	REST3	28	50
	674	37.50	37.02	35.53	98	1	VEST	REST3	28	50
	675	37.49	37.02	35.52	96	1	VEST	REST3	28	50
	676	37.48	37.00	35.52	104	1	VEST	REST3	28	50
	677	37.48	36.99	35.54	95	1	VEST	REST3	28	50
	678	37.47	37.00	35.54	99	1	VEST	REST3	28	50
	679	37.47	37.00	35.45	98	1	VEST	REST3	28	50
	680	37.48	37.00	35.37	103	1	VEST	REST3	28	50
	681	37.48	36.98	35.39	109	1	VEST	REST3	28	50
	682	37.47	36.99	35.40	85	1	VEST	REST3	28	50
	683	37.47	37.02	35.31	97	1	VEST	REST3	28	50
	684	37.47	37.00	35.26	99	1	VEST	REST3	28	50
	685	37.46	36.97	35.28	96	1	VEST	REST3	28	50
	686	37.46	36.98	35.27	95	1	VEST	REST3	28	50
	687	37.47	36.99	35.27	95	1	VEST	REST3	28	50
	688	37.47	37.01	35.26	95	1	VEST	REST3	28	50
	689	37.47	37.02	35.25	96	1	VEST	REST3	28	50
	690	37.48	37.04	35.25	93	1	VEST	REST3	28	50
115	691	37.48	37.04	35.23	93	1	VEST	REST3	28	50
	692	37.49	37.03	35.16	91	1	VEST	REST3	28	50
	693	37.50	37.04	35.12	94	1	VEST	REST3	28	50
	694	37.52	37.07	35.08	92	1	VEST	REST3	28	50
	695	37.52	37.08	35.01	91	1	VEST	REST3	28	50
	696	37.50	37.06	34.97	87	1	VEST	REST3	28	50
	697	37.50	37.07	34.96	92	1	VEST	REST3	28	50
	698	37.49	37.08	34.96	93	1	VEST	REST3	28	50
	699	37.47	37.08	34.92	93	1	VEST	REST3	28	50
	700	37.47	37.08	34.91	93	1	VEST	REST3	28	50
	701	37.47	37.08	34.92	90	1	VEST	REST3	28	50
	702	37.46	37.09	34.89	95	1	VEST	REST3	28	50
	703	37.47	37.07	34.86	99	1	VEST	REST3	28	50

min	number	Tre	Tes	Tsk-head	HR	ID	condition	period	Ta	RH
	704	37.48	37.07	34.86	97	1	VEST	REST3	28	50
	705	37.47	37.07	34.85	93	1	VEST	REST3	28	50
	706	37.47	37.08	34.83	92	1	VEST	REST3	28	50
	707	37.46	37.10	34.84	93	1	VEST	REST3	28	50
	708	37.45	37.09	34.79	91	1	VEST	REST3	28	50
	709	37.45	37.08	34.74	92	1	VEST	REST3	28	50
0	1	36.85	36.86	35.34	73	2	VEST	REST1	28	50
	2	36.85	36.84	35.34	72	2	VEST	REST1	28	50
	3	36.86	36.84	35.30	71	2	VEST	REST1	28	50
	4	36.86	36.84	35.29	71	2	VEST	REST1	28	50
	5	36.87	36.84	35.32	72	2	VEST	REST1	28	50
	6	36.87	36.84	35.32	74	2	VEST	REST1	28	50
	7	36.88	36.84	35.28	82	2	VEST	REST1	28	50
	8	36.87	36.83	35.31	88	2	VEST	REST1	28	50
	9	36.86	36.82	35.32	68	2	VEST	REST1	28	50
	10	36.86	36.82	35.28	65	2	VEST	REST1	28	50
	11	36.85	36.82	35.28	67	2	VEST	REST1	28	50
	12	36.85	36.68	35.24	70	2	VEST	REST1	28	50
	13	36.84	36.64	35.24	68	2	VEST	REST1	28	50
	14	36.83	36.75	35.25	65	2	VEST	REST1	28	50
	15	36.83	36.76	35.26	71	2	VEST	REST1	28	50
	16	36.83	36.74	35.26	71	2	VEST	REST1	28	50
	17	36.84	36.75	35.25	70	2	VEST	REST1	28	50
	18	36.84	36.77	35.29	66	2	VEST	REST1	28	50
	19	36.84	36.77	35.29	65	2	VEST	REST1	28	50
	20	36.83	36.76	35.32	70	2	VEST	REST1	28	50
	21	36.83	36.77	35.38	69	2	VEST	REST1	28	50
	22	36.83	36.79	35.38	67	2	VEST	REST1	28	50
	23	36.82	36.79	35.34	68	2	VEST	REST1	28	50
	24	36.82	36.78	35.36	70	2	VEST	REST1	28	50
	25	36.82	36.79	35.36	68	2	VEST	REST1	28	50
	26	36.83	36.78	35.34	71	2	VEST	REST1	28	50
	27	36.82	36.78	35.36	66	2	VEST	REST1	28	50
	28	36.81	36.79	35.37	68	2	VEST	REST1	28	50
	29	36.81	36.79	35.42	69	2	VEST	REST1	28	50
	30	36.81	36.78	35.43	67	2	VEST	REST1	28	50
5	31	36.82	36.79	35.37	68	2	VEST	REST1	28	50
	32	36.83	36.80	35.37	68	2	VEST	REST1	28	50
	33	36.83	36.80	35.41	71	2	VEST	REST1	28	50
	34	36.83	36.80	35.42	71	2	VEST	REST1	28	50
	35	36.83	36.81	35.38	75	2	VEST	REST1	28	50
	36	36.83	36.82	35.41	73	2	VEST	REST1	28	50
	37	36.82	36.82	35.41	76	2	VEST	REST1	28	50
	38	36.81	36.81	35.41	66	2	VEST	REST1	28	50
	39	36.81	36.81	35.45	71	2	VEST	REST1	28	50
	40	36.80	36.81	35.37	72	2	VEST	REST1	28	50

min	number	Tre	Tes	Tsk-head	HR	ID	condition	period	Ta	RH
	41	36.80	36.82	35.37	72	2	VEST	REST1	28	50
	42	36.79	36.82	35.45	71	2	VEST	REST1	28	50
	43	36.79	36.81	35.44	71	2	VEST	REST1	28	50
	44	36.79	36.81	35.44	75	2	VEST	REST1	28	50
	45	36.79	36.82	35.46	72	2	VEST	REST1	28	50
	46	36.79	36.82	35.45	71	2	VEST	REST1	28	50
	47	36.79	36.82	35.43	70	2	VEST	REST1	28	50
	48	36.78	36.82	35.43	73	2	VEST	REST1	28	50
	49	36.77	36.79	35.45	71	2	VEST	REST1	28	50
	50	36.78	36.78	35.49	70	2	VEST	REST1	28	50
	51	36.78	36.81	35.46	67	2	VEST	REST1	28	50
	52	36.77	36.82	35.39	68	2	VEST	REST1	28	50
	53	36.77	36.81	35.37	73	2	VEST	REST1	28	50
	54	36.77	36.80	35.42	74	2	VEST	REST1	28	50
	55	36.76	36.75	35.49	72	2	VEST	REST1	28	50
	56	36.77	36.71	35.50	69	2	VEST	REST1	28	50
	57	36.78	36.77	35.50	69	2	VEST	REST1	28	50
	58	36.77	36.80	35.50	71	2	VEST	REST1	28	50
	59	36.77	36.80	35.50	71	2	VEST	REST1	28	50
	60	36.77	36.82	35.49	73	2	VEST	REST1	28	50
10	61	36.77	36.82	35.46	72	2	VEST	REST1	28	50
	62	36.77	36.82	35.44	71	2	VEST	REST1	28	50
	63	36.77	36.81	35.44	69	2	VEST	REST1	28	50
	64	36.77	36.81	35.46	73	2	VEST	REST1	28	50
	65	36.78	36.80	35.47	69	2	VEST	REST1	28	50
	66	36.77	36.78	35.46	67	2	VEST	REST1	28	50
	67	36.76	36.78	35.41	69	2	VEST	REST1	28	50
	68	36.76	36.79	35.44	70	2	VEST	REST1	28	50
	69	36.76	36.80	35.47	68	2	VEST	REST1	28	50
	70	36.76	36.79	35.45	67	2	VEST	REST1	28	50
	71	36.76	36.79	35.40	75	2	VEST	REST1	28	50
	72	36.76	36.78	35.31	76	2	VEST	REST1	28	50
	73	36.75	36.78	35.31	77	2	VEST	REST1	28	50
	74	36.75	36.76	35.37	76	2	VEST	REST1	28	50
	75	36.74	36.76	35.41	76	2	VEST	REST1	28	50
	76	36.74	36.78	35.44	73	2	VEST	REST1	28	50
	77	36.75	36.79	35.42	71	2	VEST	REST1	28	50
	78	36.75	36.80	35.42	70	2	VEST	REST1	28	50
	79	36.75	36.80	35.45	71	2	VEST	REST1	28	50
	80	36.75	36.79	35.44	70	2	VEST	REST1	28	50
	81	36.74	36.79	35.46	68	2	VEST	REST1	28	50
	82	36.74	36.79	35.43	68	2	VEST	REST1	28	50
	83	36.74	36.80	35.36	69	2	VEST	REST1	28	50
	84	36.74	36.79	35.34	77	2	VEST	REST1	28	50
	85	36.74	36.78	35.39	80	2	VEST	REST1	28	50
	86	36.73	36.77	35.42	71	2	VEST	REST1	28	50

min	number	Tre	Tes	Tsk-head	HR	ID	condition	period	Ta	RH
	87	36.73	36.78	35.37	71	2	VEST	REST1	28	50
	88	36.73	36.80	35.36	71	2	VEST	REST1	28	50
	89	36.72	36.79	35.43	70	2	VEST	REST1	28	50
	90	36.72	36.78	35.38	78	2	VEST	REST1	28	50
15	91	36.72	36.70	35.30	91	2	VEST	REST1	28	50
	92	36.72	36.70	35.39	103	2	VEST	REST1	28	50
	93	36.71	36.58	35.46	72	2	VEST	REST1	28	50
	94	36.71	36.50	35.45	72	2	VEST	REST1	28	50
	95	36.71	36.65	35.42	81	2	VEST	REST1	28	50
	96	36.71	36.75	35.40	85	2	VEST	REST1	28	50
	97	36.70	36.75	35.40	75	2	VEST	REST1	28	50
	98	36.70	36.68	35.41	72	2	VEST	REST1	28	50
	99	36.70	36.68	35.39	74	2	VEST	REST1	28	50
	100	36.70	36.69	35.45	74	2	VEST	REST1	28	50
	101	36.71	36.70	35.44	76	2	VEST	REST1	28	50
	102	36.71	36.70	35.42	72	2	VEST	REST1	28	50
	103	36.71	36.82	35.50	72	2	VEST	REST1	40	50
	104	36.70	36.72	35.65	99	2	VEST	REST1	40	50
	105	36.69	36.57	35.85	70	2	VEST	REST1	40	50
	106	36.69	36.64	35.92	69	2	VEST	REST1	40	50
	107	36.69	36.64	35.94	74	2	VEST	REST1	40	50
	108	36.69	36.61	36.00	75	2	VEST	REST1	40	50
	109	36.69	36.60	36.01	74	2	VEST	REST1	40	50
	110	36.69	36.62	36.03	85	2	VEST	REST1	40	50
	111	36.68	36.60	36.07	81	2	VEST	REST1	40	50
	112	36.68	36.59	36.08	85	2	VEST	REST1	40	50
	113	36.67	36.60	36.08	86	2	VEST	REST1	40	50
	114	36.67	36.62	36.11	73	2	VEST	REST1	40	50
	115	36.68	36.62	36.14	73	2	VEST	REST1	40	50
	116	36.69	36.62	36.14	73	2	VEST	REST1	40	50
	117	36.69	36.61	36.15	75	2	VEST	REST1	40	50
	118	36.70	36.62	36.18	74	2	VEST	REST1	40	50
	119	36.69	36.63	36.21	71	2	VEST	REST1	40	50
	120	36.69	36.62	36.20	75	2	VEST	REST1	40	50
20	121	36.69	36.64	36.23	78	2	VEST	REST1	40	50
	122	36.69	36.66	36.28	80	2	VEST	REST1	40	50
	123	36.69	36.66	36.32	84	2	VEST	REST1	40	50
	124	36.69	36.65	36.35	73	2	VEST	REST1	40	50
	125	36.70	36.66	36.37	68	2	VEST	REST1	40	50
	126	36.69	36.67	36.37	69	2	VEST	REST1	40	50
	127	36.69	36.67	36.38	71	2	VEST	REST1	40	50
	128	36.70	36.68	36.41	82	2	VEST	REST1	40	50
	129	36.70	36.68	36.42	77	2	VEST	REST1	40	50
	130	36.71	36.69	36.45	74	2	VEST	REST1	40	50
	131	36.71	36.69	36.49	79	2	VEST	REST1	40	50
	132	36.71	36.70	36.48	77	2	VEST	REST1	40	50

min	number	Tre	Tes	Tsk-head	HR	ID	condition	period	Ta	RH
	133	36.70	36.70	36.49	76	2	VEST	REST1	40	50
	134	36.70	36.71	36.54	90	2	VEST	REST1	40	50
	135	36.70	36.73	36.54	95	2	VEST	REST1	40	50
	136	36.70	36.74	36.51	77	2	VEST	REST1	40	50
	137	36.70	36.78	36.53	89	2	VEST	REST1	40	50
	138	36.71	36.76	36.54	78	2	VEST	REST1	40	50
	139	36.72	36.70	36.53	76	2	VEST	EXERCISE1	40	50
	140	36.71	36.69	36.54	93	2	VEST	EXERCISE1	40	50
	141	36.71	36.63	36.58	100	2	VEST	EXERCISE1	40	50
	142	36.72	36.63	36.62	98	2	VEST	EXERCISE1	40	50
	143	36.72	36.67	36.64	102	2	VEST	EXERCISE1	40	50
	144	36.70	36.67	36.62	108	2	VEST	EXERCISE1	40	50
	145	36.70	36.66	36.61	107	2	VEST	EXERCISE1	40	50
	146	36.71	36.66	36.63	105	2	VEST	EXERCISE1	40	50
	147	36.71	36.65	36.67	106	2	VEST	EXERCISE1	40	50
	148	36.71	36.66	36.68	104	2	VEST	EXERCISE1	40	50
	149	36.70	36.66	36.67	102	2	VEST	EXERCISE1	40	50
	150	36.70	36.66	36.65	110	2	VEST	EXERCISE1	40	50
25	151	36.70	36.66	36.61	107	2	VEST	EXERCISE1	40	50
	152	36.71	36.67	36.60	111	2	VEST	EXERCISE1	40	50
	153	36.72	36.67	36.63	107	2	VEST	EXERCISE1	40	50
	154	36.72	36.68	36.67	107	2	VEST	EXERCISE1	40	50
	155	36.72	36.69	36.66	103	2	VEST	EXERCISE1	40	50
	156	36.72	36.69	36.66	107	2	VEST	EXERCISE1	40	50
	157	36.72	36.70	36.68	108	2	VEST	EXERCISE1	40	50
	158	36.72	36.70	36.66	108	2	VEST	EXERCISE1	40	50
	159	36.72	36.70	36.67	110	2	VEST	EXERCISE1	40	50
	160	36.72	36.72	36.69	110	2	VEST	EXERCISE1	40	50
	161	36.72	36.74	36.70	111	2	VEST	EXERCISE1	40	50
	162	36.71	36.75	36.69	110	2	VEST	EXERCISE1	40	50
	163	36.71	36.76	36.66	108	2	VEST	EXERCISE1	40	50
	164	36.72	36.78	36.66	108	2	VEST	EXERCISE1	40	50
	165	36.73	36.79	36.67	109	2	VEST	EXERCISE1	40	50
	166	36.73	36.80	36.65	110	2	VEST	EXERCISE1	40	50
	167	36.74	36.81	36.66	109	2	VEST	EXERCISE1	40	50
	168	36.74	36.83	36.68	109	2	VEST	EXERCISE1	40	50
	169	36.73	36.84	36.69	111	2	VEST	EXERCISE1	40	50
	170	36.73	36.85	36.71	111	2	VEST	EXERCISE1	40	50
	171	36.73	36.86	36.70	112	2	VEST	EXERCISE1	40	50
	172	36.74	36.87	36.67	113	2	VEST	EXERCISE1	40	50
	173	36.74	36.89	36.66	113	2	VEST	EXERCISE1	40	50
	174	36.75	36.94	36.68	114	2	VEST	EXERCISE1	40	50
	175	36.76	36.93	36.69	113	2	VEST	EXERCISE1	40	50
	176	36.76	36.89	36.68	112	2	VEST	EXERCISE1	40	50
	177	36.76	36.91	36.64	112	2	VEST	EXERCISE1	40	50
	178	36.77	36.94	36.66	112	2	VEST	EXERCISE1	40	50

min	number	Tre	Tes	Tsk-head	HR	ID	condition	period	Ta	RH
	179	36.78	36.96	36.67	113	2	VEST	EXERCISE1	40	50
	180	36.78	36.97	36.66	105	2	VEST	EXERCISE1	40	50
30	181	36.79	36.98	36.66	114	2	VEST	EXERCISE1	40	50
	182	36.79	36.99	36.66	113	2	VEST	EXERCISE1	40	50
	183	36.79	37.00	36.69	113	2	VEST	EXERCISE1	40	50
	184	36.81	37.01	36.69	117	2	VEST	EXERCISE1	40	50
	185	36.81	37.01	36.66	117	2	VEST	EXERCISE1	40	50
	186	36.81	37.01	36.64	117	2	VEST	EXERCISE1	40	50
	187	36.81	37.03	36.64	115	2	VEST	EXERCISE1	40	50
	188	36.81	37.05	36.67	115	2	VEST	EXERCISE1	40	50
	189	36.82	37.05	36.71	117	2	VEST	EXERCISE1	40	50
	190	36.83	37.07	36.71	117	2	VEST	EXERCISE1	40	50
	191	36.84	37.08	36.69	117	2	VEST	EXERCISE1	40	50
	192	36.84	37.09	36.71	117	2	VEST	EXERCISE1	40	50
	193	36.84	37.11	36.74	116	2	VEST	EXERCISE1	40	50
	194	36.85	37.10	36.74	117	2	VEST	EXERCISE1	40	50
	195	36.85	37.10	36.72	116	2	VEST	EXERCISE1	40	50
	196	36.85	37.11	36.71	117	2	VEST	EXERCISE1	40	50
	197	36.86	37.11	36.72	122	2	VEST	EXERCISE1	40	50
	198	36.86	37.09	36.71	121	2	VEST	EXERCISE1	40	50
	199	36.87	37.10	36.72	120	2	VEST	EXERCISE1	40	50
	200	36.87	37.11	36.71	122	2	VEST	EXERCISE1	40	50
	201	36.88	37.10	36.69	122	2	VEST	EXERCISE1	40	50
	202	36.89	37.10	36.71	117	2	VEST	EXERCISE1	40	50
	203	36.89	37.11	36.70	118	2	VEST	EXERCISE1	40	50
	204	36.89	37.12	36.69	119	2	VEST	EXERCISE1	40	50
	205	36.90	37.12	36.68	119	2	VEST	EXERCISE1	40	50
	206	36.90	37.10	36.64	117	2	VEST	EXERCISE1	40	50
	207	36.91	37.10	36.66	119	2	VEST	EXERCISE1	40	50
	208	36.91	37.13	36.65	122	2	VEST	EXERCISE1	40	50
	209	36.91	37.13	36.59	124	2	VEST	EXERCISE1	40	50
	210	36.91	37.14	36.54	122	2	VEST	EXERCISE1	40	50
35	211	36.92	37.14	36.52	121	2	VEST	EXERCISE1	40	50
	212	36.92	37.14	36.51	121	2	VEST	EXERCISE1	40	50
	213	36.93	37.15	36.51	122	2	VEST	EXERCISE1	40	50
	214	36.94	37.14	36.52	120	2	VEST	EXERCISE1	40	50
	215	36.93	37.15	36.51	121	2	VEST	EXERCISE1	40	50
	216	36.93	37.17	36.48	122	2	VEST	EXERCISE1	40	50
	217	36.95	37.15	36.47	122	2	VEST	EXERCISE1	40	50
	218	36.96	37.13	36.48	124	2	VEST	EXERCISE1	40	50
	219	36.96	37.22	36.48	124	2	VEST	EXERCISE1	40	50
	220	36.96	37.32	36.46	125	2	VEST	EXERCISE1	40	50
	221	36.96	37.22	36.45	123	2	VEST	EXERCISE1	40	50
	222	36.97	37.10	36.45	122	2	VEST	EXERCISE1	40	50
	223	36.97	37.09	36.44	121	2	VEST	EXERCISE1	40	50
	224	36.98	36.94	36.40	124	2	VEST	EXERCISE1	40	50

min	number	Tre	Tes	Tsk-head	HR	ID	condition	period	Ta	RH
	225	36.98	36.92	36.38	123	2	VEST	EXERCISE1	40	50
	226	36.98	37.08	36.38	121	2	VEST	EXERCISE1	40	50
	227	36.98	37.12	36.40	121	2	VEST	EXERCISE1	40	50
	228	36.99	37.15	36.44	122	2	VEST	EXERCISE1	40	50
	229	36.99	37.17	36.42	123	2	VEST	EXERCISE1	40	50
	230	36.99	37.19	36.40	124	2	VEST	EXERCISE1	40	50
	231	37.00	37.21	36.41	123	2	VEST	EXERCISE1	40	50
	232	37.01	37.21	36.40	121	2	VEST	EXERCISE1	40	50
	233	37.01	37.21	36.35	125	2	VEST	EXERCISE1	40	50
	234	37.02	37.22	36.28	123	2	VEST	EXERCISE1	40	50
	235	37.02	37.23	36.22	125	2	VEST	EXERCISE1	40	50
	236	37.02	37.23	36.17	126	2	VEST	EXERCISE1	40	50
	237	37.03	37.23	36.12	127	2	VEST	EXERCISE1	40	50
	238	37.03	37.23	36.10	127	2	VEST	EXERCISE1	40	50
	239	37.03	37.23	36.10	125	2	VEST	EXERCISE1	40	50
	240	37.04	37.25	36.10	126	2	VEST	EXERCISE1	40	50
40	241	37.04	37.27	36.10	124	2	VEST	EXERCISE1	40	50
	242	37.04	37.26	36.07	126	2	VEST	EXERCISE1	40	50
	243	37.04	37.25	36.08	124	2	VEST	EXERCISE1	40	50
	244	37.05	37.26	36.07	124	2	VEST	EXERCISE1	40	50
	245	37.06	37.26	36.04	122	2	VEST	EXERCISE1	40	50
	246	37.06	37.27	36.01	124	2	VEST	EXERCISE1	40	50
	247	37.07	37.27	36.00	126	2	VEST	EXERCISE1	40	50
	248	37.07	37.25	36.00	127	2	VEST	EXERCISE1	40	50
	249	37.07	37.27	36.03	126	2	VEST	EXERCISE1	40	50
	250	37.08	37.20	36.03	130	2	VEST	EXERCISE1	40	50
	251	37.08	37.13	35.99	129	2	VEST	EXERCISE1	40	50
	252	37.08	37.20	36.00	128	2	VEST	EXERCISE1	40	50
	253	37.08	37.23	36.05	129	2	VEST	EXERCISE1	40	50
	254	37.09	37.23	36.09	129	2	VEST	EXERCISE1	40	50
	255	37.09	37.22	36.11	125	2	VEST	EXERCISE1	40	50
	256	37.09	37.22	36.15	123	2	VEST	EXERCISE1	40	50
	257	37.10	37.25	36.15	124	2	VEST	EXERCISE1	40	50
	258	37.11	37.24	36.12	127	2	VEST	EXERCISE1	40	50
	259	37.11	37.13	36.12	131	2	VEST	EXERCISE1	40	50
	260	37.11	37.12	36.14	129	2	VEST	EXERCISE1	40	50
	261	37.12	37.20	36.10	130	2	VEST	EXERCISE1	40	50
	262	37.12	37.19	36.05	130	2	VEST	EXERCISE1	40	50
	263	37.12	37.17	36.02	128	2	VEST	EXERCISE1	40	50
	264	37.12	37.17	36.03	129	2	VEST	EXERCISE1	40	50
	265	37.12	37.18	36.05	130	2	VEST	EXERCISE1	40	50
	266	37.12	37.21	36.03	132	2	VEST	EXERCISE1	40	50
	267	37.13	37.23	36.00	129	2	VEST	EXERCISE1	40	50
	268	37.14	37.22	36.05	123	2	VEST	EXERCISE1	40	50
	269	37.14	37.23	36.11	127	2	VEST	EXERCISE1	40	50
	270	37.14	37.24	36.09	129	2	VEST	EXERCISE1	40	50

min	number	Tre	Tes	Tsk-head	HR	ID	condition	period	Ta	RH
45	271	37.14	37.24	36.05	130	2	VEST	EXERCISE1	40	50
	272	37.14	37.20	36.07	130	2	VEST	EXERCISE1	40	50
	273	37.14	37.17	36.07	129	2	VEST	EXERCISE1	40	50
	274	37.15	37.22	36.10	129	2	VEST	EXERCISE1	40	50
	275	37.16	37.24	36.18	128	2	VEST	EXERCISE1	40	50
	276	37.16	37.25	36.23	129	2	VEST	EXERCISE1	40	50
	277	37.16	37.27	36.23	130	2	VEST	EXERCISE1	40	50
	278	37.17	37.24	36.19	133	2	VEST	EXERCISE1	40	50
	279	37.17	37.24	36.15	131	2	VEST	EXERCISE1	40	50
	280	37.17	37.27	36.10	129	2	VEST	EXERCISE1	40	50
	281	37.17	37.26	36.05	130	2	VEST	EXERCISE1	40	50
	282	37.18	37.20	36.02	129	2	VEST	EXERCISE1	40	50
	283	37.18	37.18	36.04	128	2	VEST	EXERCISE1	40	50
	284	37.18	37.23	36.05	128	2	VEST	EXERCISE1	40	50
	285	37.18	37.25	36.06	129	2	VEST	EXERCISE1	40	50
	286	37.18	37.26	36.10	129	2	VEST	EXERCISE1	40	50
	287	37.19	37.28	36.10	129	2	VEST	EXERCISE1	40	50
	288	37.19	37.29	36.08	131	2	VEST	EXERCISE1	40	50
	289	37.19	37.29	36.06	130	2	VEST	EXERCISE1	40	50
	290	37.19	37.28	36.04	130	2	VEST	EXERCISE1	40	50
	291	37.21	37.28	36.04	130	2	VEST	EXERCISE1	40	50
	292	37.21	37.30	36.05	129	2	VEST	EXERCISE1	40	50
	293	37.21	37.33	36.06	129	2	VEST	EXERCISE1	40	50
	294	37.21	37.35	36.06	130	2	VEST	EXERCISE1	40	50
	295	37.20	37.34	36.06	130	2	VEST	EXERCISE1	40	50
	296	37.21	37.34	36.07	129	2	VEST	EXERCISE1	40	50
	297	37.21	37.35	36.07	131	2	VEST	EXERCISE1	40	50
	298	37.21	37.34	36.09	131	2	VEST	EXERCISE1	40	50
	299	37.22	37.33	36.09	133	2	VEST	EXERCISE1	40	50
	300	37.23	37.35	36.04	131	2	VEST	EXERCISE1	40	50
50	301	37.23	37.36	36.04	132	2	VEST	EXERCISE1	40	50
	302	37.23	37.35	36.02	134	2	VEST	EXERCISE1	40	50
	303	37.23	37.37	35.96	133	2	VEST	EXERCISE1	40	50
	304	37.24	37.52	35.94	133	2	VEST	EXERCISE1	40	50
	305	37.25	37.51	35.97	136	2	VEST	EXERCISE1	40	50
	306	37.25	37.31	36.03	133	2	VEST	EXERCISE1	40	50
	307	37.25	37.25	36.04	132	2	VEST	EXERCISE1	40	50
	308	37.26	37.26	36.03	131	2	VEST	EXERCISE1	40	50
	309	37.26	37.28	36.02	132	2	VEST	EXERCISE1	40	50
	310	37.26	37.29	36.02	131	2	VEST	EXERCISE1	40	50
	311	37.26	37.30	36.05	131	2	VEST	EXERCISE1	40	50
	312	37.26	37.32	36.08	128	2	VEST	EXERCISE1	40	50
	313	37.27	37.35	36.07	129	2	VEST	EXERCISE1	40	50
	314	37.26	37.41	36.06	131	2	VEST	EXERCISE1	40	50
	315	37.26	37.39	36.06	133	2	VEST	EXERCISE1	40	50
	316	37.27	37.35	36.13	132	2	VEST	EXERCISE1	40	50

min	number	Tre	Tes	Tsk-head	HR	ID	condition	period	Ta	RH
	317	37.27	37.37	36.17	132	2	VEST	EXERCISE1	40	50
	318	37.27	37.38	36.17	132	2	VEST	EXERCISE1	40	50
	319	37.28	37.39	36.19	132	2	VEST	EXERCISE1	40	50
	320	37.29	37.40	36.36	129	2	VEST	REST2	28	50
	321	37.29	37.41	36.40	143	2	VEST	REST2	28	50
	322	37.29	37.45	35.80	124	2	VEST	REST2	28	50
	323	37.29	37.49	35.14	122	2	VEST	REST2	28	50
	324	37.29	37.54	35.19	111	2	VEST	REST2	28	50
	325	37.29	37.55	35.27	111	2	VEST	REST2	28	50
	326	37.30	37.43	35.21	108	2	VEST	REST2	28	50
	327	37.31	37.36	35.25	110	2	VEST	REST2	28	50
	328	37.32	37.39	35.26	105	2	VEST	REST2	28	50
	329	37.32	37.39	35.23	94	2	VEST	REST2	28	50
	330	37.32	37.37	34.97	101	2	VEST	REST2	28	50
55	331	37.32	37.39	34.64	110	2	VEST	REST2	28	50
	332	37.31	37.33	34.62	109	2	VEST	REST2	28	50
	333	37.31	37.27	34.92	97	2	VEST	REST2	28	50
	334	37.32	37.25	35.19	95	2	VEST	REST2	28	50
	335	37.32	37.26	35.13	95	2	VEST	REST2	28	50
	336	37.32	37.24	34.99	101	2	VEST	REST2	28	50
	337	37.32	37.21	35.02	101	2	VEST	REST2	28	50
	338	37.32	37.16	35.12	102	2	VEST	REST2	28	50
	339	37.31	37.11	35.04	99	2	VEST	REST2	28	50
	340	37.31	34.85	34.84	110	2	VEST	REST2	28	50
	341	37.31	30.40	34.81	116	2	VEST	REST2	28	50
	342	37.30	29.81	34.93	106	2	VEST	REST2	28	50
	343	37.30	29.34	34.99	111	2	VEST	REST2	28	50
	344	37.31	29.05	34.86	95	2	VEST	REST2	28	50
	345	37.31	30.74	34.75	101	2	VEST	REST2	28	50
	346	37.31	29.21	34.83	112	2	VEST	REST2	28	50
	347	37.31	29.21	34.77	99	2	VEST	REST2	28	50
	348	37.31	28.56	34.65	103	2	VEST	REST2	28	50
	349	37.30	27.98	34.71	99	2	VEST	REST2	28	50
	350	37.30	29.00	34.77	101	2	VEST	REST2	28	50
	351	37.30	29.41	34.76	101	2	VEST	REST2	28	50
	352	37.30	30.73	34.79	104	2	VEST	REST2	28	50
	353	37.30	31.52	34.68	101	2	VEST	REST2	28	50
	354	37.29	31.62	34.44	94	2	VEST	REST2	28	50
	355	37.28	29.73	34.42	94	2	VEST	REST2	28	50
	356	37.28	29.60	34.58	97	2	VEST	REST2	28	50
	357	37.28	31.75	34.52	90	2	VEST	REST2	28	50
	358	37.28	31.03	34.39	99	2	VEST	REST2	28	50
	359	37.29	30.66	34.35	93	2	VEST	REST2	28	50
	360	37.29	31.42	34.17	100	2	VEST	REST2	28	50
60	361	37.29	31.76	34.11	91	2	VEST	REST2	28	50
	362	37.28	32.59	34.16	95	2	VEST	REST2	28	50

min	number	Tre	Tes	Tsk-head	HR	ID	condition	period	Ta	RH
	363	37.27	30.39	34.05	82	2	VEST	REST2	28	50
	364	37.27	29.98	33.97	90	2	VEST	REST2	28	50
	365	37.27	32.30	34.05	82	2	VEST	REST2	28	50
	366	37.27	33.00	34.24	96	2	VEST	REST2	28	50
	367	37.27	33.55	34.36	89	2	VEST	REST2	28	50
	368	37.26	33.88	34.31	96	2	VEST	REST2	28	50
	369	37.24	34.22	34.29	93	2	VEST	REST2	28	50
	370	37.24	34.57	34.31	93	2	VEST	REST2	28	50
	371	37.24	34.82	34.27	81	2	VEST	REST2	28	50
	372	37.24	35.05	34.32	91	2	VEST	REST2	28	50
	373	37.24	35.21	34.33	89	2	VEST	REST2	28	50
	374	37.24	33.73	34.28	92	2	VEST	REST2	28	50
	375	37.24	33.08	34.27	74	2	VEST	REST2	28	50
	376	37.23	34.17	34.23	86	2	VEST	REST2	28	50
	377	37.23	34.68	34.04	89	2	VEST	REST2	28	50
	378	37.22	35.03	34.03	81	2	VEST	REST2	28	50
	379	37.22	32.00	34.19	91	2	VEST	REST2	28	50
	380	37.22	30.48	33.98	94	2	VEST	REST2	28	50
	381	37.23	32.71	33.78	83	2	VEST	REST2	28	50
	382	37.22	33.54	33.90	78	2	VEST	REST2	28	50
	383	37.22	33.93	33.98	91	2	VEST	REST2	28	50
	384	37.22	34.28	34.03	81	2	VEST	REST2	28	50
	385	37.22	34.62	33.94	81	2	VEST	REST2	28	50
	386	37.22	34.89	33.61	78	2	VEST	REST2	28	50
	387	37.22	35.02	33.50	87	2	VEST	REST2	28	50
	388	37.22	35.19	33.78	92	2	VEST	REST2	28	50
	389	37.22	35.35	33.88	89	2	VEST	REST2	28	50
	390	37.22	35.57	33.88	82	2	VEST	REST2	28	50
65	391	37.23	35.70	33.82	86	2	VEST	REST2	28	50
	392	37.22	35.75	33.58	80	2	VEST	REST2	28	50
	393	37.21	35.83	33.55	85	2	VEST	REST2	28	50
	394	37.21	35.91	33.66	80	2	VEST	REST2	28	50
	395	37.20	36.03	33.58	78	2	VEST	REST2	28	50
	396	37.19	36.11	33.37	82	2	VEST	REST2	28	50
	397	37.20	36.15	33.21	80	2	VEST	REST2	28	50
	398	37.20	36.22	33.01	80	2	VEST	REST2	28	50
	399	37.20	36.27	32.83	87	2	VEST	REST2	28	50
	400	37.21	36.28	32.82	85	2	VEST	REST2	28	50
	401	37.21	36.30	32.86	77	2	VEST	REST2	28	50
	402	37.21	36.34	32.78	70	2	VEST	REST2	28	50
	403	37.22	36.39	32.71	74	2	VEST	REST2	28	50
	404	37.21	36.43	32.73	73	2	VEST	REST2	28	50
	405	37.21	36.45	32.71	76	2	VEST	REST2	28	50
	406	37.22	36.48	32.81	77	2	VEST	REST2	28	50
	407	37.22	36.52	32.87	77	2	VEST	REST2	28	50
	408	37.22	36.55	32.82	74	2	VEST	REST2	28	50

min	number	Tre	Tes	Tsk-head	HR	ID	condition	period	Ta	RH
	409	37.22	36.57	32.83	75	2	VEST	REST2	28	50
	410	37.21	36.59	32.81	75	2	VEST	REST2	28	50
	411	37.22	36.61	32.73	80	2	VEST	REST2	28	50
	412	37.23	36.62	32.69	78	2	VEST	REST2	28	50
	413	37.22	36.62	32.63	74	2	VEST	REST2	28	50
	414	37.22	36.64	32.51	73	2	VEST	REST2	28	50
	415	37.23	36.67	32.54	72	2	VEST	REST2	28	50
	416	37.23	36.69	32.71	73	2	VEST	REST2	28	50
	417	37.23	36.71	32.84	75	2	VEST	REST2	28	50
	418	37.23	36.65	32.88	81	2	VEST	REST2	28	50
	419	37.22	36.63	32.86	74	2	VEST	REST2	28	50
	420	37.22	36.71	32.58	74	2	VEST	REST2	28	50
70	421	37.22	36.74	32.33	75	2	VEST	REST2	28	50
	422	37.23	36.75	32.46	81	2	VEST	REST2	28	50
	423	37.24	36.77	32.53	71	2	VEST	REST2	28	50
	424	37.24	36.79	32.66	74	2	VEST	REST2	28	50
	425	37.24	36.80	32.72	75	2	VEST	REST2	28	50
	426	37.23	36.80	32.61	76	2	VEST	REST2	28	50
	427	37.23	36.82	32.54	70	2	VEST	REST2	28	50
	428	37.23	36.84	32.31	80	2	VEST	REST2	28	50
	429	37.23	36.86	32.14	75	2	VEST	REST2	28	50
	430	37.24	36.86	32.36	76	2	VEST	REST2	28	50
	431	37.24	36.80	32.60	81	2	VEST	REST2	28	50
	432	37.23	36.77	32.51	80	2	VEST	REST2	28	50
	433	37.23	36.83	32.47	75	2	VEST	REST2	28	50
	434	37.23	36.85	32.53	76	2	VEST	REST2	28	50
	435	37.23	36.87	32.36	75	2	VEST	REST2	28	50
	436	37.22	36.88	32.24	78	2	VEST	REST2	28	50
	437	37.22	36.88	32.19	84	2	VEST	REST2	28	50
	438	37.22	36.89	32.12	95	2	VEST	REST2	28	50
	439	37.21	36.89	32.26	80	2	VEST	REST2	40	50
	440	37.19	36.90	32.41	82	2	VEST	REST2	40	50
	441	37.18	36.63	32.83	99	2	VEST	REST2	40	50
	442	37.17	36.57	33.35	96	2	VEST	REST2	40	50
	443	37.18	36.81	33.56	74	2	VEST	REST2	40	50
	444	37.19	36.83	33.79	76	2	VEST	REST2	40	50
	445	37.19	36.84	33.99	77	2	VEST	REST2	40	50
	446	37.20	36.84	34.18	75	2	VEST	REST2	40	50
	447	37.20	36.84	34.40	76	2	VEST	REST2	40	50
	448	37.20	36.85	34.55	74	2	VEST	REST2	40	50
	449	37.21	36.86	34.60	77	2	VEST	REST2	40	50
	450	37.21	36.86	34.60	71	2	VEST	REST2	40	50
75	451	37.21	36.85	34.70	73	2	VEST	REST2	40	50
	452	37.22	36.85	34.82	75	2	VEST	REST2	40	50
	453	37.22	36.85	34.91	82	2	VEST	REST2	40	50
	454	37.21	36.86	34.94	77	2	VEST	REST2	40	50

min	number	Tre	Tes	Tsk-head	HR	ID	condition	period	Ta	RH
	455	37.21	36.87	34.94	82	2	VEST	REST2	40	50
	456	37.21	36.88	35.02	79	2	VEST	REST2	40	50
	457	37.21	36.87	35.04	82	2	VEST	REST2	40	50
	458	37.22	36.82	34.91	104	2	VEST	REST2	40	50
	459	37.21	36.79	34.85	98	2	VEST	REST2	40	50
	460	37.21	36.81	34.95	84	2	VEST	REST2	40	50
	461	37.21	36.81	35.04	81	2	VEST	REST2	40	50
	462	37.22	36.80	35.10	79	2	VEST	REST2	40	50
	463	37.22	36.80	35.17	86	2	VEST	EXERCISE2	40	50
	464	37.22	36.84	35.13	92	2	VEST	EXERCISE2	40	50
	465	37.22	36.52	34.98	105	2	VEST	EXERCISE2	40	50
	466	37.22	36.48	34.91	105	2	VEST	EXERCISE2	40	50
	467	37.22	36.82	34.94	108	2	VEST	EXERCISE2	40	50
	468	37.21	36.84	34.99	105	2	VEST	EXERCISE2	40	50
	469	37.21	36.86	34.99	104	2	VEST	EXERCISE2	40	50
	470	37.21	36.87	34.98	110	2	VEST	EXERCISE2	40	50
	471	37.20	36.88	35.00	110	2	VEST	EXERCISE2	40	50
	472	37.21	36.90	35.01	111	2	VEST	EXERCISE2	40	50
	473	37.21	36.92	35.06	110	2	VEST	EXERCISE2	40	50
	474	37.21	36.93	35.09	111	2	VEST	EXERCISE2	40	50
	475	37.21	36.94	35.08	111	2	VEST	EXERCISE2	40	50
	476	37.21	36.96	35.08	114	2	VEST	EXERCISE2	40	50
	477	37.21	36.97	35.12	112	2	VEST	EXERCISE2	40	50
	478	37.21	36.99	35.16	113	2	VEST	EXERCISE2	40	50
	479	37.20	37.01	35.26	114	2	VEST	EXERCISE2	40	50
	480	37.20	37.01	35.32	112	2	VEST	EXERCISE2	40	50
80	481	37.20	37.02	35.32	113	2	VEST	EXERCISE2	40	50
	482	37.21	37.04	35.33	115	2	VEST	EXERCISE2	40	50
	483	37.22	37.05	35.34	114	2	VEST	EXERCISE2	40	50
	484	37.22	37.06	35.37	112	2	VEST	EXERCISE2	40	50
	485	37.22	37.09	35.42	111	2	VEST	EXERCISE2	40	50
	486	37.22	36.78	35.46	114	2	VEST	EXERCISE2	40	50
	487	37.22	36.54	35.48	115	2	VEST	EXERCISE2	40	50
	488	37.22	36.80	35.54	118	2	VEST	EXERCISE2	40	50
	489	37.21	37.00	35.61	115	2	VEST	EXERCISE2	40	50
	490	37.22	37.04	35.63	116	2	VEST	EXERCISE2	40	50
	491	37.22	37.08	35.62	120	2	VEST	EXERCISE2	40	50
	492	37.22	37.09	35.68	118	2	VEST	EXERCISE2	40	50
	493	37.23	37.11	35.71	114	2	VEST	EXERCISE2	40	50
	494	37.24	37.13	35.67	116	2	VEST	EXERCISE2	40	50
	495	37.24	37.14	35.63	116	2	VEST	EXERCISE2	40	50
	496	37.23	37.16	35.64	117	2	VEST	EXERCISE2	40	50
	497	37.23	37.17	35.64	119	2	VEST	EXERCISE2	40	50
	498	37.23	37.18	35.67	120	2	VEST	EXERCISE2	40	50
	499	37.23	37.19	35.72	118	2	VEST	EXERCISE2	40	50
	500	37.24	37.18	35.74	118	2	VEST	EXERCISE2	40	50

min	number	Tre	Tes	Tsk-head	HR	ID	condition	period	Ta	RH
	501	37.24	37.17	35.76	116	2	VEST	EXERCISE2	40	50
	502	37.24	37.20	35.81	116	2	VEST	EXERCISE2	40	50
	503	37.24	37.22	35.82	117	2	VEST	EXERCISE2	40	50
	504	37.24	37.23	35.81	118	2	VEST	EXERCISE2	40	50
	505	37.25	37.24	35.86	118	2	VEST	EXERCISE2	40	50
	506	37.25	37.25	35.88	117	2	VEST	EXERCISE2	40	50
	507	37.25	37.26	35.88	117	2	VEST	EXERCISE2	40	50
	508	37.25	37.26	35.91	118	2	VEST	EXERCISE2	40	50
	509	37.25	37.28	35.91	117	2	VEST	EXERCISE2	40	50
	510	37.26	37.29	35.89	116	2	VEST	EXERCISE2	40	50
85	511	37.26	37.29	35.94	117	2	VEST	EXERCISE2	40	50
	512	37.27	37.29	35.98	116	2	VEST	EXERCISE2	40	50
	513	37.28	37.30	35.99	117	2	VEST	EXERCISE2	40	50
	514	37.28	37.31	36.00	118	2	VEST	EXERCISE2	40	50
	515	37.28	37.31	35.97	119	2	VEST	EXERCISE2	40	50
	516	37.28	37.31	35.94	118	2	VEST	EXERCISE2	40	50
	517	37.29	37.32	35.94	120	2	VEST	EXERCISE2	40	50
	518	37.30	37.33	35.95	125	2	VEST	EXERCISE2	40	50
	519	37.29	37.26	35.96	124	2	VEST	EXERCISE2	40	50
	520	37.29	37.23	35.98	115	2	VEST	EXERCISE2	40	50
	521	37.30	37.27	36.00	117	2	VEST	EXERCISE2	40	50
	522	37.29	37.29	36.03	120	2	VEST	EXERCISE2	40	50
	523	37.29	37.28	36.05	120	2	VEST	EXERCISE2	40	50
	524	37.31	37.28	36.04	122	2	VEST	EXERCISE2	40	50
	525	37.31	37.12	36.02	122	2	VEST	EXERCISE2	40	50
	526	37.31	37.11	35.99	120	2	VEST	EXERCISE2	40	50
	527	37.31	37.29	35.98	120	2	VEST	EXERCISE2	40	50
	528	37.31	37.30	36.00	120	2	VEST	EXERCISE2	40	50
	529	37.31	37.31	35.99	121	2	VEST	EXERCISE2	40	50
	530	37.31	37.31	36.00	120	2	VEST	EXERCISE2	40	50
	531	37.32	37.30	36.05	121	2	VEST	EXERCISE2	40	50
	532	37.32	37.31	36.10	122	2	VEST	EXERCISE2	40	50
	533	37.32	37.32	36.11	124	2	VEST	EXERCISE2	40	50
	534	37.33	37.36	36.13	119	2	VEST	EXERCISE2	40	50
	535	37.33	37.35	36.15	120	2	VEST	EXERCISE2	40	50
	536	37.33	37.31	36.11	126	2	VEST	EXERCISE2	40	50
	537	37.33	37.31	36.07	123	2	VEST	EXERCISE2	40	50
	538	37.33	37.33	36.08	123	2	VEST	EXERCISE2	40	50
	539	37.34	37.35	36.07	121	2	VEST	EXERCISE2	40	50
	540	37.34	37.35	36.02	123	2	VEST	EXERCISE2	40	50
90	541	37.34	37.37	36.01	118	2	VEST	EXERCISE2	40	50
	542	37.35	37.39	36.05	120	2	VEST	EXERCISE2	40	50
	543	37.35	37.39	36.07	120	2	VEST	EXERCISE2	40	50
	544	37.35	37.38	36.04	123	2	VEST	EXERCISE2	40	50
	545	37.35	37.38	36.06	121	2	VEST	EXERCISE2	40	50
	546	37.35	37.40	36.09	120	2	VEST	EXERCISE2	40	50

min	number	Tre	Tes	Tsk-head	HR	ID	condition	period	Ta	RH
	547	37.35	37.38	36.08	121	2	VEST	EXERCISE2	40	50
	548	37.36	37.37	36.08	122	2	VEST	EXERCISE2	40	50
	549	37.35	37.36	36.10	122	2	VEST	EXERCISE2	40	50
	550	37.36	37.36	36.10	125	2	VEST	EXERCISE2	40	50
	551	37.37	37.38	36.06	127	2	VEST	EXERCISE2	40	50
	552	37.37	37.31	36.03	126	2	VEST	EXERCISE2	40	50
	553	37.39	37.22	36.07	124	2	VEST	EXERCISE2	40	50
	554	37.39	37.26	36.10	123	2	VEST	EXERCISE2	40	50
	555	37.38	37.22	36.08	121	2	VEST	EXERCISE2	40	50
	556	37.38	37.19	36.05	120	2	VEST	EXERCISE2	40	50
	557	37.38	37.26	36.03	121	2	VEST	EXERCISE2	40	50
	558	37.38	37.29	36.05	123	2	VEST	EXERCISE2	40	50
	559	37.38	37.32	36.05	124	2	VEST	EXERCISE2	40	50
	560	37.38	37.33	36.07	123	2	VEST	EXERCISE2	40	50
	561	37.39	37.34	36.09	123	2	VEST	EXERCISE2	40	50
	562	37.39	37.35	36.06	124	2	VEST	EXERCISE2	40	50
	563	37.39	37.37	36.02	124	2	VEST	EXERCISE2	40	50
	564	37.39	37.38	35.98	126	2	VEST	EXERCISE2	40	50
	565	37.39	37.39	35.94	125	2	VEST	EXERCISE2	40	50
	566	37.39	37.38	35.89	125	2	VEST	EXERCISE2	40	50
	567	37.39	37.38	35.88	124	2	VEST	EXERCISE2	40	50
	568	37.39	37.37	35.91	130	2	VEST	EXERCISE2	40	50
	569	37.40	37.32	35.93	128	2	VEST	EXERCISE2	40	50
	570	37.41	37.26	35.93	120	2	VEST	EXERCISE2	40	50
95	571	37.41	37.32	35.95	124	2	VEST	EXERCISE2	40	50
	572	37.41	37.37	36.03	123	2	VEST	EXERCISE2	40	50
	573	37.41	37.36	36.11	124	2	VEST	EXERCISE2	40	50
	574	37.42	37.37	36.08	123	2	VEST	EXERCISE2	40	50
	575	37.42	37.38	36.05	125	2	VEST	EXERCISE2	40	50
	576	37.42	37.39	36.10	125	2	VEST	EXERCISE2	40	50
	577	37.42	37.37	36.14	126	2	VEST	EXERCISE2	40	50
	578	37.42	37.38	36.12	125	2	VEST	EXERCISE2	40	50
	579	37.43	37.43	36.08	125	2	VEST	EXERCISE2	40	50
	580	37.43	37.44	36.07	127	2	VEST	EXERCISE2	40	50
	581	37.43	37.44	36.09	128	2	VEST	EXERCISE2	40	50
	582	37.44	37.46	36.13	124	2	VEST	EXERCISE2	40	50
	583	37.43	37.46	36.11	127	2	VEST	EXERCISE2	40	50
	584	37.43	37.42	36.12	129	2	VEST	EXERCISE2	40	50
	585	37.44	37.42	36.09	125	2	VEST	EXERCISE2	40	50
	586	37.43	37.43	36.06	127	2	VEST	EXERCISE2	40	50
	587	37.43	37.44	36.12	128	2	VEST	EXERCISE2	40	50
	588	37.44	37.46	36.15	128	2	VEST	EXERCISE2	40	50
	589	37.45	37.45	36.18	128	2	VEST	EXERCISE2	40	50
	590	37.45	37.45	36.18	128	2	VEST	EXERCISE2	40	50
	591	37.45	37.44	36.17	129	2	VEST	EXERCISE2	40	50
	592	37.45	37.44	36.13	130	2	VEST	EXERCISE2	40	50

min	number	Tre	Tes	Tsk-head	HR	ID	condition	period	Ta	RH
	593	37.46	37.46	36.13	130	2	VEST	EXERCISE2	40	50
	594	37.47	37.45	36.17	129	2	VEST	EXERCISE2	40	50
	595	37.47	37.45	36.17	129	2	VEST	EXERCISE2	40	50
	596	37.47	37.47	36.17	130	2	VEST	EXERCISE2	40	50
	597	37.47	37.54	36.11	130	2	VEST	EXERCISE2	40	50
	598	37.47	37.45	36.11	132	2	VEST	EXERCISE2	40	50
	599	37.47	37.35	36.17	132	2	VEST	EXERCISE2	40	50
	600	37.47	37.40	36.19	127	2	VEST	EXERCISE2	40	50
100	601	37.48	37.42	36.21	126	2	VEST	EXERCISE2	40	50
	602	37.49	37.34	36.21	129	2	VEST	EXERCISE2	40	50
	603	37.49	37.27	36.21	130	2	VEST	EXERCISE2	40	50
	604	37.49	37.34	36.23	130	2	VEST	EXERCISE2	40	50
	605	37.48	37.39	36.23	129	2	VEST	EXERCISE2	40	50
	606	37.48	37.40	36.21	129	2	VEST	EXERCISE2	40	50
	607	37.49	37.41	36.18	132	2	VEST	EXERCISE2	40	50
	608	37.49	37.42	36.18	130	2	VEST	EXERCISE2	40	50
	609	37.48	37.43	36.15	130	2	VEST	EXERCISE2	40	50
	610	37.48	37.44	36.13	131	2	VEST	EXERCISE2	40	50
	611	37.49	37.45	36.17	130	2	VEST	EXERCISE2	40	50
	612	37.50	37.44	36.20	130	2	VEST	EXERCISE2	40	50
	613	37.50	37.44	36.21	128	2	VEST	EXERCISE2	40	50
	614	37.51	37.45	36.21	129	2	VEST	EXERCISE2	40	50
	615	37.52	37.46	36.23	133	2	VEST	EXERCISE2	40	50
	616	37.52	37.48	36.24	132	2	VEST	EXERCISE2	40	50
	617	37.52	37.49	36.25	133	2	VEST	EXERCISE2	40	50
	618	37.52	37.52	36.26	132	2	VEST	EXERCISE2	40	50
	619	37.53	37.53	36.21	130	2	VEST	EXERCISE2	40	50
	620	37.53	37.51	36.15	130	2	VEST	EXERCISE2	40	50
	621	37.53	37.49	36.13	132	2	VEST	EXERCISE2	40	50
	622	37.53	37.48	36.13	135	2	VEST	EXERCISE2	40	50
	623	37.54	37.19	36.13	137	2	VEST	EXERCISE2	40	50
	624	37.53	37.15	36.13	133	2	VEST	EXERCISE2	40	50
	625	37.52	37.42	36.16	132	2	VEST	EXERCISE2	40	50
	626	37.53	37.46	36.22	136	2	VEST	EXERCISE2	40	50
	627	37.54	37.42	36.27	136	2	VEST	EXERCISE2	40	50
	628	37.54	37.40	36.29	129	2	VEST	EXERCISE2	40	50
	629	37.54	37.45	36.30	132	2	VEST	EXERCISE2	40	50
	630	37.54	37.48	36.29	132	2	VEST	EXERCISE2	40	50
105	631	37.54	37.49	36.27	131	2	VEST	EXERCISE2	40	50
	632	37.54	37.47	36.25	132	2	VEST	EXERCISE2	40	50
	633	37.54	37.48	36.24	131	2	VEST	EXERCISE2	40	50
	634	37.56	37.52	36.22	133	2	VEST	EXERCISE2	40	50
	635	37.57	37.52	36.23	134	2	VEST	EXERCISE2	40	50
	636	37.58	37.52	36.26	135	2	VEST	EXERCISE2	40	50
	637	37.57	37.51	36.28	134	2	VEST	EXERCISE2	40	50
	638	37.56	37.51	36.29	134	2	VEST	EXERCISE2	40	50

min	number	Tre	Tes	Tsk-head	HR	ID	condition	period	Ta	RH
	639	37.56	37.53	36.29	133	2	VEST	EXERCISE2	40	50
	640	37.56	37.54	36.30	131	2	VEST	EXERCISE2	40	50
	641	37.56	37.54	36.29	132	2	VEST	EXERCISE2	40	50
	642	37.56	37.55	36.28	133	2	VEST	EXERCISE2	40	50
	643	37.57	37.55	36.26	131	2	VEST	EXERCISE2	40	50
	644	37.57	37.57	36.29	132	2	VEST	REST3	28	50
	645	37.57	37.59	36.25	134	2	VEST	REST3	28	50
	646	37.57	37.61	35.76	129	2	VEST	REST3	28	50
	647	37.57	37.62	35.12	123	2	VEST	REST3	28	50
	648	37.56	37.63	34.99	123	2	VEST	REST3	28	50
	649	37.56	37.64	35.02	115	2	VEST	REST3	28	50
	650	37.57	37.65	34.87	119	2	VEST	REST3	28	50
	651	37.57	37.65	34.82	112	2	VEST	REST3	28	50
	652	37.57	37.63	35.06	107	2	VEST	REST3	28	50
	653	37.58	37.63	35.28	106	2	VEST	REST3	28	50
	654	37.60	37.64	35.32	105	2	VEST	REST3	28	50
	655	37.60	37.62	35.36	102	2	VEST	REST3	28	50
	656	37.60	37.59	35.21	102	2	VEST	REST3	28	50
	657	37.60	37.59	35.04	99	2	VEST	REST3	28	50
	658	37.60	37.59	35.03	101	2	VEST	REST3	28	50
	659	37.61	37.58	35.02	97	2	VEST	REST3	28	50
	660	37.61	37.55	34.94	97	2	VEST	REST3	28	50
110	661	37.62	37.53	34.90	98	2	VEST	REST3	28	50
	662	37.62	37.51	34.94	96	2	VEST	REST3	28	50
	663	37.62	37.50	34.85	98	2	VEST	REST3	28	50
	664	37.62	37.48	34.71	95	2	VEST	REST3	28	50
	665	37.61	37.44	34.67	93	2	VEST	REST3	28	50
	666	37.61	37.45	34.59	101	2	VEST	REST3	28	50
	667	37.61	37.45	34.50	99	2	VEST	REST3	28	50
	668	37.60	37.41	34.32	94	2	VEST	REST3	28	50
	669	37.61	37.38	34.05	97	2	VEST	REST3	28	50
	670	37.61	37.38	33.77	97	2	VEST	REST3	28	50
	671	37.61	37.37	33.70	94	2	VEST	REST3	28	50
	672	37.61	37.36	33.81	91	2	VEST	REST3	28	50
	673	37.61	37.35	33.77	92	2	VEST	REST3	28	50
	674	37.60	37.32	33.79	91	2	VEST	REST3	28	50
	675	37.60	37.30	33.87	87	2	VEST	REST3	28	50
	676	37.60	37.29	33.87	87	2	VEST	REST3	28	50
	677	37.60	37.27	33.71	91	2	VEST	REST3	28	50
	678	37.59	37.26	33.50	90	2	VEST	REST3	28	50
	679	37.60	37.23	33.33	90	2	VEST	REST3	28	50
	680	37.59	36.94	33.29	94	2	VEST	REST3	28	50
	681	37.59	36.88	33.52	93	2	VEST	REST3	28	50
	682	37.59	37.12	33.59	83	2	VEST	REST3	28	50
	683	37.58	37.14	33.36	97	2	VEST	REST3	28	50
	684	37.58	37.15	33.18	93	2	VEST	REST3	28	50

min	number	Tre	Tes	Tsk-head	HR	ID	condition	period	Ta	RH
	685	37.58	36.98	33.13	91	2	VEST	REST3	28	50
	686	37.57	36.93	33.13	90	2	VEST	REST3	28	50
	687	37.57	37.03	33.29	92	2	VEST	REST3	28	50
	688	37.57	37.01	33.46	84	2	VEST	REST3	28	50
	689	37.56	37.07	33.44	89	2	VEST	REST3	28	50
	690	37.56	37.15	33.42	90	2	VEST	REST3	28	50
115	691	37.57	37.21	33.25	91	2	VEST	REST3	28	50
	692	37.57	37.11	32.78	93	2	VEST	REST3	28	50
	693	37.58	37.11	32.40	95	2	VEST	REST3	28	50
	694	37.57	37.25	32.30	79	2	VEST	REST3	28	50
	695	37.56	37.23	32.24	84	2	VEST	REST3	28	50
	696	37.57	37.23	32.13	89	2	VEST	REST3	28	50
	697	37.57	37.24	32.04	82	2	VEST	REST3	28	50
	698	37.58	37.24	31.90	81	2	VEST	REST3	28	50
	699	37.59	37.25	31.80	79	2	VEST	REST3	28	50
	700	37.58	37.24	31.72	83	2	VEST	REST3	28	50
	701	37.58	37.09	31.50	88	2	VEST	REST3	28	50
	702	37.58	37.04	31.30	85	2	VEST	REST3	28	50
	703	37.58	37.14	31.29	83	2	VEST	REST3	28	50
	704	37.58	37.18	31.26	84	2	VEST	REST3	28	50
	705	37.58	37.21	31.09	84	2	VEST	REST3	28	50
	706	37.58	37.23	31.12	84	2	VEST	REST3	28	50
	707	37.57	37.14	31.32	91	2	VEST	REST3	28	50
	708	37.57	37.08	31.58	99	2	VEST	REST3	28	50
	709	37.58	37.15	31.92	88	2	VEST	REST3	28	50
0	1	36.97	36.93	35.64	89	3	VEST	REST1	28	50
	2	36.96	36.92	35.63	88	3	VEST	REST1	28	50
	3	36.96	36.91	35.63	80	3	VEST	REST1	28	50
	4	36.96	36.91	35.65	81	3	VEST	REST1	28	50
	5	36.97	36.91	35.66	94	3	VEST	REST1	28	50
	6	36.97	36.92	35.65	86	3	VEST	REST1	28	50
	7	36.96	36.95	35.65	86	3	VEST	REST1	28	50
	8	36.96	36.94	35.65	85	3	VEST	REST1	28	50
	9	36.96	36.94	35.63	82	3	VEST	REST1	28	50
	10	36.98	36.95	35.63	86	3	VEST	REST1	28	50
	11	36.99	36.95	35.65	78	3	VEST	REST1	28	50
	12	36.98	36.95	35.65	87	3	VEST	REST1	28	50
	13	36.98	36.93	35.64	92	3	VEST	REST1	28	50
	14	36.98	36.93	35.65	86	3	VEST	REST1	28	50
	15	36.98	36.95	35.68	83	3	VEST	REST1	28	50
	16	36.99	36.94	35.68	88	3	VEST	REST1	28	50
	17	36.99	36.92	35.68	82	3	VEST	REST1	28	50
	18	36.99	36.92	35.68	81	3	VEST	REST1	28	50
	19	37.00	36.93	35.66	82	3	VEST	REST1	28	50
	20	36.99	36.93	35.65	77	3	VEST	REST1	28	50
	21	36.99	36.93	35.66	79	3	VEST	REST1	28	50

min	number	Tre	Tes	Tsk-head	HR	ID	condition	period	Ta	RH
	22	37.00	36.94	35.67	85	3	VEST	REST1	28	50
	23	36.99	36.94	35.67	79	3	VEST	REST1	28	50
	24	37.00	36.95	35.68	83	3	VEST	REST1	28	50
	25	37.01	36.95	35.69	87	3	VEST	REST1	28	50
	26	37.01	36.94	35.68	83	3	VEST	REST1	28	50
	27	37.00	36.93	35.69	89	3	VEST	REST1	28	50
	28	37.01	36.95	35.70	81	3	VEST	REST1	28	50
	29	37.01	36.94	35.70	79	3	VEST	REST1	28	50
	30	37.00	36.94	35.71	83	3	VEST	REST1	28	50
5	31	37.00	36.96	35.71	83	3	VEST	REST1	28	50
	32	37.00	36.97	35.72	79	3	VEST	REST1	28	50
	33	37.00	36.96	35.71	77	3	VEST	REST1	28	50
	34	36.99	36.96	35.72	78	3	VEST	REST1	28	50
	35	37.00	36.97	35.73	89	3	VEST	REST1	28	50
	36	37.00	36.98	35.74	87	3	VEST	REST1	28	50
	37	37.00	36.98	35.73	84	3	VEST	REST1	28	50
	38	37.01	36.97	35.73	85	3	VEST	REST1	28	50
	39	37.01	36.95	35.74	90	3	VEST	REST1	28	50
	40	37.00	36.94	35.74	85	3	VEST	REST1	28	50
	41	37.00	36.94	35.75	84	3	VEST	REST1	28	50
	42	37.01	36.95	35.75	79	3	VEST	REST1	28	50
	43	37.01	36.95	35.76	80	3	VEST	REST1	28	50
	44	37.01	36.94	35.76	81	3	VEST	REST1	28	50
	45	37.00	36.95	35.77	83	3	VEST	REST1	28	50
	46	37.01	36.95	35.78	88	3	VEST	REST1	28	50
	47	37.02	36.94	35.77	93	3	VEST	REST1	28	50
	48	37.01	36.92	35.76	86	3	VEST	REST1	28	50
	49	37.02	36.92	35.75	80	3	VEST	REST1	28	50
	50	37.02	36.93	35.75	81	3	VEST	REST1	28	50
	51	37.02	36.92	35.76	88	3	VEST	REST1	28	50
	52	37.02	36.92	35.76	84	3	VEST	REST1	28	50
	53	37.02	36.91	35.76	76	3	VEST	REST1	28	50
	54	37.02	36.92	35.77	77	3	VEST	REST1	28	50
	55	37.02	36.94	35.79	78	3	VEST	REST1	28	50
	56	37.02	36.94	35.79	76	3	VEST	REST1	28	50
	57	37.03	36.94	35.79	78	3	VEST	REST1	28	50
	58	37.02	36.94	35.79	80	3	VEST	REST1	28	50
	59	37.02	36.95	35.78	77	3	VEST	REST1	28	50
	60	37.02	36.92	35.78	81	3	VEST	REST1	28	50
10	61	37.02	36.90	35.77	85	3	VEST	REST1	28	50
	62	37.03	36.90	35.77	83	3	VEST	REST1	28	50
	63	37.03	36.92	35.79	79	3	VEST	REST1	28	50
	64	37.02	36.92	35.80	84	3	VEST	REST1	28	50
	65	37.02	36.91	35.80	77	3	VEST	REST1	28	50
	66	37.03	36.92	35.80	77	3	VEST	REST1	28	50
	67	37.04	36.93	35.80	83	3	VEST	REST1	28	50

min	number	Tre	Tes	Tsk-head	HR	ID	condition	period	Ta	RH
	68	37.03	36.93	35.81	78	3	VEST	REST1	28	50
	69	37.03	36.94	35.82	79	3	VEST	REST1	28	50
	70	37.03	36.94	35.82	78	3	VEST	REST1	28	50
	71	37.02	36.93	35.81	78	3	VEST	REST1	28	50
	72	37.02	36.92	35.81	74	3	VEST	REST1	28	50
	73	37.03	36.93	35.81	74	3	VEST	REST1	28	50
	74	37.03	36.93	35.82	81	3	VEST	REST1	28	50
	75	37.03	36.89	35.80	100	3	VEST	REST1	28	50
	76	37.02	36.88	35.78	93	3	VEST	REST1	28	50
	77	37.02	36.91	35.79	79	3	VEST	REST1	28	50
	78	37.02	36.90	35.79	79	3	VEST	REST1	28	50
	79	37.02	36.89	35.77	86	3	VEST	REST1	28	50
	80	37.02	36.89	35.77	79	3	VEST	REST1	28	50
	81	37.03	36.89	35.78	84	3	VEST	REST1	28	50
	82	37.03	36.89	35.77	73	3	VEST	REST1	28	50
	83	37.02	36.90	35.77	78	3	VEST	REST1	28	50
	84	37.02	36.90	35.78	79	3	VEST	REST1	28	50
	85	37.02	36.89	35.78	79	3	VEST	REST1	28	50
	86	37.02	36.89	35.77	77	3	VEST	REST1	28	50
	87	37.02	36.90	35.76	76	3	VEST	REST1	28	50
	88	37.03	36.92	35.78	79	3	VEST	REST1	28	50
	89	37.03	36.93	35.77	79	3	VEST	REST1	28	50
	90	37.04	36.92	35.74	88	3	VEST	REST1	28	50
15	91	37.03	36.91	35.74	87	3	VEST	REST1	28	50
	92	37.02	36.92	35.75	88	3	VEST	REST1	28	50
	93	37.03	36.91	35.74	83	3	VEST	REST1	28	50
	94	37.03	36.91	35.76	79	3	VEST	REST1	28	50
	95	37.03	36.92	35.79	87	3	VEST	REST1	28	50
	96	37.03	36.92	35.78	83	3	VEST	REST1	28	50
	97	37.03	36.91	35.76	102	3	VEST	REST1	28	50
	98	37.01	36.90	35.76	87	3	VEST	REST1	28	50
	99	37.01	36.89	35.77	87	3	VEST	REST1	28	50
	100	37.01	36.91	35.78	90	3	VEST	REST1	28	50
	101	37.02	36.93	35.79	88	3	VEST	REST1	28	50
	102	37.02	36.90	35.78	88	3	VEST	REST1	28	50
	103	37.01	36.90	35.79	79	3	VEST	REST1	40	50
	104	37.01	36.90	35.89	86	3	VEST	REST1	40	50
	105	37.02	36.90	36.09	100	3	VEST	REST1	40	50
	106	37.03	36.92	36.25	102	3	VEST	REST1	40	50
	107	37.02	36.93	36.34	110	3	VEST	REST1	40	50
	108	37.02	36.93	36.41	97	3	VEST	REST1	40	50
	109	37.03	36.92	36.51	84	3	VEST	REST1	40	50
	110	37.04	36.93	36.57	100	3	VEST	REST1	40	50
	111	37.05	36.92	36.61	98	3	VEST	REST1	40	50
	112	37.05	36.89	36.67	95	3	VEST	REST1	40	50
	113	37.03	36.89	36.69	101	3	VEST	REST1	40	50

min	number	Tre	Tes	Tsk-head	HR	ID	condition	period	Ta	RH
	114	37.03	36.89	36.73	106	3	VEST	REST1	40	50
	115	37.04	36.89	36.76	95	3	VEST	REST1	40	50
	116	37.04	36.89	36.78	104	3	VEST	REST1	40	50
	117	37.04	36.89	36.81	103	3	VEST	REST1	40	50
	118	37.03	36.90	36.80	101	3	VEST	REST1	40	50
	119	37.03	36.91	36.81	111	3	VEST	REST1	40	50
	120	37.04	36.91	36.85	97	3	VEST	REST1	40	50
20	121	37.04	36.89	36.88	93	3	VEST	REST1	40	50
	122	37.05	36.91	36.89	90	3	VEST	REST1	40	50
	123	37.05	36.93	36.91	85	3	VEST	REST1	40	50
	124	37.05	36.94	36.93	96	3	VEST	REST1	40	50
	125	37.05	36.95	36.95	101	3	VEST	REST1	40	50
	126	37.05	36.94	36.97	92	3	VEST	REST1	40	50
	127	37.06	36.94	36.99	96	3	VEST	REST1	40	50
	128	37.07	36.97	37.01	90	3	VEST	REST1	40	50
	129	37.07	36.99	37.03	96	3	VEST	REST1	40	50
	130	37.06	36.98	37.06	98	3	VEST	REST1	40	50
	131	37.06	36.99	37.06	111	3	VEST	REST1	40	50
	132	37.06	36.99	37.09	91	3	VEST	REST1	40	50
	133	37.06	36.99	37.13	95	3	VEST	REST1	40	50
	134	37.06	37.01	37.14	103	3	VEST	REST1	40	50
	135	37.06	37.02	37.14	104	3	VEST	REST1	40	50
	136	37.06	37.01	37.15	97	3	VEST	REST1	40	50
	137	37.06	37.04	37.14	93	3	VEST	REST1	40	50
	138	37.06	37.06	37.13	107	3	VEST	REST1	40	50
	139	37.06	36.99	37.14	118	3	VEST	EXERCISE1	40	50
	140	37.06	36.97	37.16	100	3	VEST	EXERCISE1	40	50
	141	37.06	37.00	37.16	103	3	VEST	EXERCISE1	40	50
	142	37.07	37.01	37.17	103	3	VEST	EXERCISE1	40	50
	143	37.07	37.00	37.19	105	3	VEST	EXERCISE1	40	50
	144	37.07	37.00	37.18	106	3	VEST	EXERCISE1	40	50
	145	37.08	37.01	37.17	105	3	VEST	EXERCISE1	40	50
	146	37.08	37.01	37.18	113	3	VEST	EXERCISE1	40	50
	147	37.08	37.00	37.20	111	3	VEST	EXERCISE1	40	50
	148	37.08	36.99	37.21	110	3	VEST	EXERCISE1	40	50
	149	37.08	36.99	37.20	110	3	VEST	EXERCISE1	40	50
	150	37.08	37.01	37.20	110	3	VEST	EXERCISE1	40	50
25	151	37.08	37.01	37.19	110	3	VEST	EXERCISE1	40	50
	152	37.08	37.00	37.21	113	3	VEST	EXERCISE1	40	50
	153	37.09	37.03	37.21	114	3	VEST	EXERCISE1	40	50
	154	37.09	37.03	37.21	114	3	VEST	EXERCISE1	40	50
	155	37.10	37.03	37.21	110	3	VEST	EXERCISE1	40	50
	156	37.10	37.04	37.21	113	3	VEST	EXERCISE1	40	50
	157	37.10	37.04	37.23	114	3	VEST	EXERCISE1	40	50
	158	37.10	37.04	37.24	115	3	VEST	EXERCISE1	40	50
	159	37.10	37.06	37.23	113	3	VEST	EXERCISE1	40	50

min	number	Tre	Tes	Tsk-head	HR	ID	condition	period	Ta	RH
	160	37.10	37.07	37.22	113	3	VEST	EXERCISE1	40	50
	161	37.11	37.09	37.22	113	3	VEST	EXERCISE1	40	50
	162	37.12	37.10	37.23	111	3	VEST	EXERCISE1	40	50
	163	37.12	37.10	37.24	114	3	VEST	EXERCISE1	40	50
	164	37.13	37.10	37.25	114	3	VEST	EXERCISE1	40	50
	165	37.12	37.11	37.25	114	3	VEST	EXERCISE1	40	50
	166	37.13	37.13	37.23	111	3	VEST	EXERCISE1	40	50
	167	37.14	37.14	37.23	113	3	VEST	EXERCISE1	40	50
	168	37.14	37.15	37.24	118	3	VEST	EXERCISE1	40	50
	169	37.15	37.16	37.24	118	3	VEST	EXERCISE1	40	50
	170	37.15	37.17	37.23	115	3	VEST	EXERCISE1	40	50
	171	37.15	37.17	37.23	116	3	VEST	EXERCISE1	40	50
	172	37.15	37.18	37.24	114	3	VEST	EXERCISE1	40	50
	173	37.16	37.20	37.23	114	3	VEST	EXERCISE1	40	50
	174	37.16	37.20	37.23	119	3	VEST	EXERCISE1	40	50
	175	37.17	37.20	37.23	118	3	VEST	EXERCISE1	40	50
	176	37.18	37.22	37.24	116	3	VEST	EXERCISE1	40	50
	177	37.18	37.23	37.25	115	3	VEST	EXERCISE1	40	50
	178	37.19	37.25	37.24	115	3	VEST	EXERCISE1	40	50
	179	37.20	37.25	37.23	118	3	VEST	EXERCISE1	40	50
	180	37.19	37.24	37.23	118	3	VEST	EXERCISE1	40	50
30	181	37.20	37.27	37.22	118	3	VEST	EXERCISE1	40	50
	182	37.21	37.29	37.23	118	3	VEST	EXERCISE1	40	50
	183	37.21	37.29	37.22	115	3	VEST	EXERCISE1	40	50
	184	37.21	37.29	37.22	116	3	VEST	EXERCISE1	40	50
	185	37.22	37.29	37.23	116	3	VEST	EXERCISE1	40	50
	186	37.23	37.30	37.23	119	3	VEST	EXERCISE1	40	50
	187	37.23	37.31	37.24	120	3	VEST	EXERCISE1	40	50
	188	37.24	37.30	37.24	118	3	VEST	EXERCISE1	40	50
	189	37.24	37.30	37.25	116	3	VEST	EXERCISE1	40	50
	190	37.24	37.29	37.25	120	3	VEST	EXERCISE1	40	50
	191	37.24	37.30	37.23	119	3	VEST	EXERCISE1	40	50
	192	37.24	37.32	37.22	120	3	VEST	EXERCISE1	40	50
	193	37.24	37.32	37.24	118	3	VEST	EXERCISE1	40	50
	194	37.25	37.32	37.25	118	3	VEST	EXERCISE1	40	50
	195	37.25	37.33	37.25	120	3	VEST	EXERCISE1	40	50
	196	37.26	37.34	37.25	120	3	VEST	EXERCISE1	40	50
	197	37.26	37.35	37.24	119	3	VEST	EXERCISE1	40	50
	198	37.26	37.35	37.23	122	3	VEST	EXERCISE1	40	50
	199	37.27	37.35	37.22	122	3	VEST	EXERCISE1	40	50
	200	37.27	37.36	37.23	123	3	VEST	EXERCISE1	40	50
	201	37.29	37.34	37.23	123	3	VEST	EXERCISE1	40	50
	202	37.29	37.32	37.23	120	3	VEST	EXERCISE1	40	50
	203	37.29	37.32	37.23	120	3	VEST	EXERCISE1	40	50
	204	37.30	37.33	37.22	118	3	VEST	EXERCISE1	40	50
	205	37.30	37.33	37.22	118	3	VEST	EXERCISE1	40	50

min	number	Tre	Tes	Tsk-head	HR	ID	condition	period	Ta	RH
	206	37.30	37.34	37.21	120	3	VEST	EXERCISE1	40	50
	207	37.29	37.34	37.20	119	3	VEST	EXERCISE1	40	50
	208	37.30	37.32	37.21	123	3	VEST	EXERCISE1	40	50
	209	37.31	37.31	37.21	122	3	VEST	EXERCISE1	40	50
	210	37.31	37.33	37.21	123	3	VEST	EXERCISE1	40	50
35	211	37.31	37.35	37.22	123	3	VEST	EXERCISE1	40	50
	212	37.31	37.37	37.21	127	3	VEST	EXERCISE1	40	50
	213	37.31	37.37	37.20	126	3	VEST	EXERCISE1	40	50
	214	37.31	37.37	37.21	122	3	VEST	EXERCISE1	40	50
	215	37.31	37.36	37.20	121	3	VEST	EXERCISE1	40	50
	216	37.31	37.34	37.20	121	3	VEST	EXERCISE1	40	50
	217	37.32	37.36	37.20	121	3	VEST	EXERCISE1	40	50
	218	37.32	37.38	37.20	120	3	VEST	EXERCISE1	40	50
	219	37.32	37.38	37.20	120	3	VEST	EXERCISE1	40	50
	220	37.33	37.37	37.19	122	3	VEST	EXERCISE1	40	50
	221	37.34	37.37	37.19	125	3	VEST	EXERCISE1	40	50
	222	37.35	37.36	37.19	128	3	VEST	EXERCISE1	40	50
	223	37.35	37.36	37.19	128	3	VEST	EXERCISE1	40	50
	224	37.35	37.38	37.20	125	3	VEST	EXERCISE1	40	50
	225	37.34	37.38	37.21	123	3	VEST	EXERCISE1	40	50
	226	37.35	37.40	37.21	129	3	VEST	EXERCISE1	40	50
	227	37.36	37.41	37.21	131	3	VEST	EXERCISE1	40	50
	228	37.36	37.40	37.21	131	3	VEST	EXERCISE1	40	50
	229	37.36	37.36	37.22	133	3	VEST	EXERCISE1	40	50
	230	37.36	37.34	37.21	132	3	VEST	EXERCISE1	40	50
	231	37.36	37.37	37.22	126	3	VEST	EXERCISE1	40	50
	232	37.37	37.39	37.23	126	3	VEST	EXERCISE1	40	50
	233	37.37	37.40	37.23	125	3	VEST	EXERCISE1	40	50
	234	37.37	37.40	37.24	126	3	VEST	EXERCISE1	40	50
	235	37.37	37.42	37.25	127	3	VEST	EXERCISE1	40	50
	236	37.38	37.43	37.25	128	3	VEST	EXERCISE1	40	50
	237	37.38	37.39	37.25	126	3	VEST	EXERCISE1	40	50
	238	37.37	37.40	37.25	123	3	VEST	EXERCISE1	40	50
	239	37.37	37.42	37.24	122	3	VEST	EXERCISE1	40	50
	240	37.38	37.43	37.25	123	3	VEST	EXERCISE1	40	50
40	241	37.39	37.42	37.25	124	3	VEST	EXERCISE1	40	50
	242	37.39	37.40	37.25	124	3	VEST	EXERCISE1	40	50
	243	37.40	37.42	37.25	122	3	VEST	EXERCISE1	40	50
	244	37.41	37.46	37.24	125	3	VEST	EXERCISE1	40	50
	245	37.41	37.48	37.24	123	3	VEST	EXERCISE1	40	50
	246	37.42	37.48	37.24	121	3	VEST	EXERCISE1	40	50
	247	37.41	37.46	37.24	131	3	VEST	EXERCISE1	40	50
	248	37.41	37.45	37.24	133	3	VEST	EXERCISE1	40	50
	249	37.41	37.43	37.24	133	3	VEST	EXERCISE1	40	50
	250	37.41	37.44	37.24	134	3	VEST	EXERCISE1	40	50
	251	37.42	37.45	37.24	137	3	VEST	EXERCISE1	40	50

min	number	Tre	Tes	Tsk-head	HR	ID	condition	period	Ta	RH
	252	37.42	37.40	37.26	135	3	VEST	EXERCISE1	40	50
	253	37.43	37.39	37.25	133	3	VEST	EXERCISE1	40	50
	254	37.43	37.44	37.24	130	3	VEST	EXERCISE1	40	50
	255	37.42	37.46	37.23	128	3	VEST	EXERCISE1	40	50
	256	37.41	37.47	37.22	128	3	VEST	EXERCISE1	40	50
	257	37.42	37.49	37.22	132	3	VEST	EXERCISE1	40	50
	258	37.42	37.47	37.22	133	3	VEST	EXERCISE1	40	50
	259	37.42	37.46	37.22	132	3	VEST	EXERCISE1	40	50
	260	37.43	37.46	37.22	138	3	VEST	EXERCISE1	40	50
	261	37.43	37.45	37.24	139	3	VEST	EXERCISE1	40	50
	262	37.44	37.42	37.25	137	3	VEST	EXERCISE1	40	50
	263	37.45	37.43	37.25	135	3	VEST	EXERCISE1	40	50
	264	37.45	37.46	37.25	131	3	VEST	EXERCISE1	40	50
	265	37.44	37.46	37.26	126	3	VEST	EXERCISE1	40	50
	266	37.45	37.46	37.25	134	3	VEST	EXERCISE1	40	50
	267	37.45	37.48	37.25	134	3	VEST	EXERCISE1	40	50
	268	37.45	37.48	37.26	131	3	VEST	EXERCISE1	40	50
	269	37.45	37.47	37.27	130	3	VEST	EXERCISE1	40	50
	270	37.45	37.49	37.26	129	3	VEST	EXERCISE1	40	50
45	271	37.46	37.50	37.26	129	3	VEST	EXERCISE1	40	50
	272	37.46	37.49	37.26	131	3	VEST	EXERCISE1	40	50
	273	37.47	37.48	37.25	134	3	VEST	EXERCISE1	40	50
	274	37.47	37.46	37.25	139	3	VEST	EXERCISE1	40	50
	275	37.47	37.44	37.25	135	3	VEST	EXERCISE1	40	50
	276	37.47	37.50	37.23	133	3	VEST	EXERCISE1	40	50
	277	37.47	37.58	37.23	130	3	VEST	EXERCISE1	40	50
	278	37.48	37.55	37.25	136	3	VEST	EXERCISE1	40	50
	279	37.49	37.51	37.26	143	3	VEST	EXERCISE1	40	50
	280	37.50	37.50	37.26	143	3	VEST	EXERCISE1	40	50
	281	37.50	37.45	37.27	146	3	VEST	EXERCISE1	40	50
	282	37.50	37.37	37.27	144	3	VEST	EXERCISE1	40	50
	283	37.49	37.39	37.27	143	3	VEST	EXERCISE1	40	50
	284	37.49	37.47	37.26	143	3	VEST	EXERCISE1	40	50
	285	37.50	37.50	37.26	143	3	VEST	EXERCISE1	40	50
	286	37.50	37.49	37.27	137	3	VEST	EXERCISE1	40	50
	287	37.50	37.49	37.27	136	3	VEST	EXERCISE1	40	50
	288	37.51	37.51	37.27	135	3	VEST	EXERCISE1	40	50
	289	37.51	37.53	37.25	152	3	VEST	EXERCISE1	40	50
	290	37.51	37.50	37.25	134	3	VEST	EXERCISE1	40	50
	291	37.52	37.48	37.25	136	3	VEST	EXERCISE1	40	50
	292	37.52	37.50	37.26	142	3	VEST	EXERCISE1	40	50
	293	37.52	37.51	37.26	143	3	VEST	EXERCISE1	40	50
	294	37.52	37.50	37.25	140	3	VEST	EXERCISE1	40	50
	295	37.52	37.51	37.25	135	3	VEST	EXERCISE1	40	50
	296	37.51	37.49	37.25	137	3	VEST	EXERCISE1	40	50
	297	37.49	37.48	37.25	143	3	VEST	EXERCISE1	40	50

min	number	Tre	Tes	Tsk-head	HR	ID	condition	period	Ta	RH
	298	37.49	37.49	37.26	142	3	VEST	EXERCISE1	40	50
	299	37.49	37.50	37.27	137	3	VEST	EXERCISE1	40	50
	300	37.48	37.54	37.27	138	3	VEST	EXERCISE1	40	50
50	301	37.48	37.55	37.27	134	3	VEST	EXERCISE1	40	50
	302	37.48	37.54	37.28	135	3	VEST	EXERCISE1	40	50
	303	37.47	37.53	37.28	136	3	VEST	EXERCISE1	40	50
	304	37.47	37.54	37.27	137	3	VEST	EXERCISE1	40	50
	305	37.47	37.55	37.27	134	3	VEST	EXERCISE1	40	50
	306	37.47	37.55	37.26	134	3	VEST	EXERCISE1	40	50
	307	37.47	37.56	37.26	136	3	VEST	EXERCISE1	40	50
	308	37.48	37.55	37.25	137	3	VEST	EXERCISE1	40	50
	309	37.48	37.53	37.26	134	3	VEST	EXERCISE1	40	50
	310	37.48	37.55	37.26	132	3	VEST	EXERCISE1	40	50
	311	37.48	37.56	37.26	140	3	VEST	EXERCISE1	40	50
	312	37.49	37.57	37.26	143	3	VEST	EXERCISE1	40	50
	313	37.49	37.58	37.26	143	3	VEST	EXERCISE1	40	50
	314	37.49	37.55	37.27	136	3	VEST	EXERCISE1	40	50
	315	37.49	37.55	37.27	161	3	VEST	EXERCISE1	40	50
	316	37.50	37.58	37.27	137	3	VEST	EXERCISE1	40	50
	317	37.51	37.58	37.27	138	3	VEST	EXERCISE1	40	50
	318	37.51	37.58	37.28	139	3	VEST	EXERCISE1	40	50
	319	37.51	37.60	37.27	139	3	VEST	EXERCISE1	40	50
	320	37.51	37.61	37.27	135	3	VEST	REST2	28	50
	321	37.51	37.60	37.24	141	3	VEST	REST2	28	50
	322	37.51	37.58	37.05	137	3	VEST	REST2	28	50
	323	37.51	37.56	36.84	127	3	VEST	REST2	28	50
	324	37.52	37.60	36.75	116	3	VEST	REST2	28	50
	325	37.51	37.62	36.68	113	3	VEST	REST2	28	50
	326	37.51	37.59	36.63	125	3	VEST	REST2	28	50
	327	37.52	37.59	36.55	126	3	VEST	REST2	28	50
	328	37.53	37.54	36.52	119	3	VEST	REST2	28	50
	329	37.52	37.52	36.49	122	3	VEST	REST2	28	50
	330	37.52	37.50	36.48	114	3	VEST	REST2	28	50
55	331	37.51	37.49	36.44	107	3	VEST	REST2	28	50
	332	37.51	37.49	36.39	126	3	VEST	REST2	28	50
	333	37.52	37.33	36.39	123	3	VEST	REST2	28	50
	334	37.51	37.21	36.38	117	3	VEST	REST2	28	50
	335	37.50	37.19	36.35	114	3	VEST	REST2	28	50
	336	37.51	37.21	36.34	114	3	VEST	REST2	28	50
	337	37.51	37.25	36.33	117	3	VEST	REST2	28	50
	338	37.50	35.19	36.31	114	3	VEST	REST2	28	50
	339	37.51	31.30	36.30	117	3	VEST	REST2	28	50
	340	37.51	27.81	36.29	113	3	VEST	REST2	28	50
	341	37.51	27.02	36.23	106	3	VEST	REST2	28	50
	342	37.51	29.98	36.17	104	3	VEST	REST2	28	50
	343	37.50	32.54	36.13	103	3	VEST	REST2	28	50

min	number	Tre	Tes	Tsk-head	HR	ID	condition	period	Ta	RH
	344	37.50	33.17	36.07	110	3	VEST	REST2	28	50
	345	37.50	30.47	36.03	112	3	VEST	REST2	28	50
	346	37.50	30.02	36.01	106	3	VEST	REST2	28	50
	347	37.49	32.86	36.00	94	3	VEST	REST2	28	50
	348	37.49	32.32	36.00	104	3	VEST	REST2	28	50
	349	37.48	30.45	35.98	105	3	VEST	REST2	28	50
	350	37.47	30.28	35.95	94	3	VEST	REST2	28	50
	351	37.46	29.92	35.93	101	3	VEST	REST2	28	50
	352	37.47	28.32	35.91	96	3	VEST	REST2	28	50
	353	37.47	27.75	35.88	99	3	VEST	REST2	28	50
	354	37.47	29.75	35.82	96	3	VEST	REST2	28	50
	355	37.47	32.24	35.77	98	3	VEST	REST2	28	50
	356	37.47	32.86	35.75	85	3	VEST	REST2	28	50
	357	37.47	32.36	35.70	105	3	VEST	REST2	28	50
	358	37.47	32.85	35.64	104	3	VEST	REST2	28	50
	359	37.47	34.30	35.57	91	3	VEST	REST2	28	50
	360	37.47	32.74	35.55	101	3	VEST	REST2	28	50
60	361	37.48	29.56	35.55	104	3	VEST	REST2	28	50
	362	37.47	29.62	35.52	105	3	VEST	REST2	28	50
	363	37.47	31.67	35.53	101	3	VEST	REST2	28	50
	364	37.47	32.50	35.56	99	3	VEST	REST2	28	50
	365	37.48	33.46	35.58	103	3	VEST	REST2	28	50
	366	37.48	30.67	35.57	92	3	VEST	REST2	28	50
	367	37.49	28.26	35.52	97	3	VEST	REST2	28	50
	368	37.49	30.04	35.47	96	3	VEST	REST2	28	50
	369	37.49	31.21	35.46	94	3	VEST	REST2	28	50
	370	37.48	32.56	35.45	86	3	VEST	REST2	28	50
	371	37.48	33.48	35.42	95	3	VEST	REST2	28	50
	372	37.48	33.97	35.39	96	3	VEST	REST2	28	50
	373	37.49	30.67	35.39	94	3	VEST	REST2	28	50
	374	37.50	28.34	35.37	93	3	VEST	REST2	28	50
	375	37.50	29.90	35.35	92	3	VEST	REST2	28	50
	376	37.50	30.98	35.31	84	3	VEST	REST2	28	50
	377	37.50	32.03	35.25	89	3	VEST	REST2	28	50
	378	37.50	32.75	35.24	80	3	VEST	REST2	28	50
	379	37.50	33.44	35.23	86	3	VEST	REST2	28	50
	380	37.50	34.01	35.21	83	3	VEST	REST2	28	50
	381	37.50	34.64	35.21	95	3	VEST	REST2	28	50
	382	37.50	35.33	35.20	90	3	VEST	REST2	28	50
	383	37.49	35.76	35.19	83	3	VEST	REST2	28	50
	384	37.49	35.91	35.19	84	3	VEST	REST2	28	50
	385	37.49	36.19	35.17	92	3	VEST	REST2	28	50
	386	37.49	36.40	35.14	84	3	VEST	REST2	28	50
	387	37.48	36.56	35.14	81	3	VEST	REST2	28	50
	388	37.48	36.62	35.13	78	3	VEST	REST2	28	50
	389	37.48	36.65	35.12	83	3	VEST	REST2	28	50

min	number	Tre	Tes	Tsk-head	HR	ID	condition	period	Ta	RH
	390	37.48	36.67	35.12	82	3	VEST	REST2	28	50
65	391	37.49	36.70	35.10	83	3	VEST	REST2	28	50
	392	37.48	36.71	35.10	78	3	VEST	REST2	28	50
	393	37.48	36.59	35.10	81	3	VEST	REST2	28	50
	394	37.48	36.67	35.10	88	3	VEST	REST2	28	50
	395	37.47	36.82	35.14	85	3	VEST	REST2	28	50
	396	37.47	36.85	35.12	89	3	VEST	REST2	28	50
	397	37.48	36.85	35.10	87	3	VEST	REST2	28	50
	398	37.47	36.83	35.14	81	3	VEST	REST2	28	50
	399	37.47	36.86	35.17	79	3	VEST	REST2	28	50
	400	37.48	36.87	35.17	78	3	VEST	REST2	28	50
	401	37.48	36.96	35.16	82	3	VEST	REST2	28	50
	402	37.48	37.08	35.18	87	3	VEST	REST2	28	50
	403	37.49	37.13	35.20	88	3	VEST	REST2	28	50
	404	37.49	37.14	35.21	85	3	VEST	REST2	28	50
	405	37.48	37.11	35.20	85	3	VEST	REST2	28	50
	406	37.47	37.11	35.20	93	3	VEST	REST2	28	50
	407	37.47	37.12	35.23	86	3	VEST	REST2	28	50
	408	37.48	37.11	35.25	97	3	VEST	REST2	28	50
	409	37.48	37.12	35.24	99	3	VEST	REST2	28	50
	410	37.48	37.15	35.26	84	3	VEST	REST2	28	50
	411	37.48	37.14	35.29	82	3	VEST	REST2	28	50
	412	37.48	37.12	35.30	82	3	VEST	REST2	28	50
	413	37.48	37.13	35.29	87	3	VEST	REST2	28	50
	414	37.48	37.15	35.27	84	3	VEST	REST2	28	50
	415	37.47	37.16	35.27	81	3	VEST	REST2	28	50
	416	37.46	37.16	35.27	84	3	VEST	REST2	28	50
	417	37.47	37.17	35.29	81	3	VEST	REST2	28	50
	418	37.48	37.19	35.32	81	3	VEST	REST2	28	50
	419	37.49	37.20	35.33	80	3	VEST	REST2	28	50
	420	37.48	37.20	35.31	99	3	VEST	REST2	28	50
70	421	37.48	37.21	35.31	82	3	VEST	REST2	28	50
	422	37.48	37.21	35.32	89	3	VEST	REST2	28	50
	423	37.48	37.22	35.33	93	3	VEST	REST2	28	50
	424	37.48	37.23	35.39	83	3	VEST	REST2	28	50
	425	37.48	37.25	35.46	84	3	VEST	REST2	28	50
	426	37.48	37.26	35.52	82	3	VEST	REST2	28	50
	427	37.48	37.25	35.56	98	3	VEST	REST2	28	50
	428	37.48	37.26	35.60	88	3	VEST	REST2	28	50
	429	37.48	37.24	35.64	92	3	VEST	REST2	28	50
	430	37.48	37.25	35.66	87	3	VEST	REST2	28	50
	431	37.48	37.27	35.68	85	3	VEST	REST2	28	50
	432	37.48	37.27	35.71	86	3	VEST	REST2	28	50
	433	37.49	37.27	35.72	93	3	VEST	REST2	28	50
	434	37.49	37.28	35.70	96	3	VEST	REST2	28	50
	435	37.49	37.27	35.73	84	3	VEST	REST2	28	50

min	number	Tre	Tes	Tsk-head	HR	ID	condition	period	Ta	RH
	436	37.49	37.27	35.74	80	3	VEST	REST2	28	50
	437	37.48	37.29	35.73	87	3	VEST	REST2	28	50
	438	37.48	37.28	35.77	85	3	VEST	REST2	28	50
	439	37.49	37.28	35.80	82	3	VEST	REST2	40	50
	440	37.49	37.30	35.95	99	3	VEST	REST2	40	50
	441	37.49	37.32	36.16	95	3	VEST	REST2	40	50
	442	37.50	37.33	36.30	98	3	VEST	REST2	40	50
	443	37.49	37.31	36.44	97	3	VEST	REST2	40	50
	444	37.49	37.31	36.52	96	3	VEST	REST2	40	50
	445	37.50	37.08	36.58	92	3	VEST	REST2	40	50
	446	37.51	37.07	36.65	96	3	VEST	REST2	40	50
	447	37.51	37.31	36.69	94	3	VEST	REST2	40	50
	448	37.51	37.33	36.73	92	3	VEST	REST2	40	50
	449	37.51	37.32	36.79	87	3	VEST	REST2	40	50
	450	37.51	37.31	36.83	87	3	VEST	REST2	40	50
75	451	37.52	37.31	36.85	93	3	VEST	REST2	40	50
	452	37.52	37.30	36.89	93	3	VEST	REST2	40	50
	453	37.52	37.30	36.94	92	3	VEST	REST2	40	50
	454	37.52	37.31	36.95	91	3	VEST	REST2	40	50
	455	37.52	37.29	36.97	92	3	VEST	REST2	40	50
	456	37.52	37.30	36.99	89	3	VEST	REST2	40	50
	457	37.53	37.31	37.01	85	3	VEST	REST2	40	50
	458	37.53	37.29	37.03	100	3	VEST	REST2	40	50
	459	37.52	37.28	37.04	94	3	VEST	REST2	40	50
	460	37.52	37.29	37.03	99	3	VEST	REST2	40	50
	461	37.52	37.28	37.04	98	3	VEST	REST2	40	50
	462	37.53	37.27	37.05	99	3	VEST	REST2	40	50
	463	37.52	37.26	37.05	101	3	VEST	EXERCISE2	40	50
	464	37.52	37.26	37.06	103	3	VEST	EXERCISE2	40	50
	465	37.53	37.28	37.06	90	3	VEST	EXERCISE2	40	50
	466	37.52	37.28	37.07	102	3	VEST	EXERCISE2	40	50
	467	37.52	37.28	37.08	101	3	VEST	EXERCISE2	40	50
	468	37.53	37.29	37.07	106	3	VEST	EXERCISE2	40	50
	469	37.53	37.29	37.08	108	3	VEST	EXERCISE2	40	50
	470	37.53	37.30	37.09	108	3	VEST	EXERCISE2	40	50
	471	37.53	37.30	37.10	108	3	VEST	EXERCISE2	40	50
	472	37.53	37.31	37.11	109	3	VEST	EXERCISE2	40	50
	473	37.53	37.32	37.12	113	3	VEST	EXERCISE2	40	50
	474	37.52	37.32	37.14	113	3	VEST	EXERCISE2	40	50
	475	37.52	37.33	37.14	111	3	VEST	EXERCISE2	40	50
	476	37.52	37.35	37.13	110	3	VEST	EXERCISE2	40	50
	477	37.53	37.37	37.13	110	3	VEST	EXERCISE2	40	50
	478	37.53	37.37	37.14	111	3	VEST	EXERCISE2	40	50
	479	37.53	37.37	37.17	113	3	VEST	EXERCISE2	40	50
	480	37.53	37.40	37.18	114	3	VEST	EXERCISE2	40	50
80	481	37.54	37.41	37.18	114	3	VEST	EXERCISE2	40	50

min	number	Tre	Tes	Tsk-head	HR	ID	condition	period	Ta	RH
	482	37.54	37.40	37.21	114	3	VEST	EXERCISE2	40	50
	483	37.54	37.41	37.20	115	3	VEST	EXERCISE2	40	50
	484	37.54	37.43	37.19	120	3	VEST	EXERCISE2	40	50
	485	37.54	37.44	37.20	118	3	VEST	EXERCISE2	40	50
	486	37.54	37.42	37.23	121	3	VEST	EXERCISE2	40	50
	487	37.55	37.43	37.24	119	3	VEST	EXERCISE2	40	50
	488	37.55	37.45	37.24	119	3	VEST	EXERCISE2	40	50
	489	37.56	37.46	37.25	121	3	VEST	EXERCISE2	40	50
	490	37.55	37.45	37.24	123	3	VEST	EXERCISE2	40	50
	491	37.55	37.46	37.24	117	3	VEST	EXERCISE2	40	50
	492	37.56	37.46	37.27	121	3	VEST	EXERCISE2	40	50
	493	37.57	37.46	37.29	121	3	VEST	EXERCISE2	40	50
	494	37.58	37.49	37.30	118	3	VEST	EXERCISE2	40	50
	495	37.58	37.51	37.31	119	3	VEST	EXERCISE2	40	50
	496	37.58	37.50	37.32	124	3	VEST	EXERCISE2	40	50
	497	37.59	37.51	37.33	122	3	VEST	EXERCISE2	40	50
	498	37.60	37.52	37.34	120	3	VEST	EXERCISE2	40	50
	499	37.60	37.52	37.34	120	3	VEST	EXERCISE2	40	50
	500	37.60	37.53	37.34	120	3	VEST	EXERCISE2	40	50
	501	37.60	37.52	37.34	117	3	VEST	EXERCISE2	40	50
	502	37.60	37.53	37.36	118	3	VEST	EXERCISE2	40	50
	503	37.61	37.54	37.36	121	3	VEST	EXERCISE2	40	50
	504	37.61	37.54	37.37	119	3	VEST	EXERCISE2	40	50
	505	37.62	37.54	37.38	123	3	VEST	EXERCISE2	40	50
	506	37.62	37.54	37.37	125	3	VEST	EXERCISE2	40	50
	507	37.62	37.54	37.38	124	3	VEST	EXERCISE2	40	50
	508	37.62	37.54	37.38	120	3	VEST	EXERCISE2	40	50
	509	37.63	37.53	37.37	121	3	VEST	EXERCISE2	40	50
	510	37.64	37.52	37.39	119	3	VEST	EXERCISE2	40	50
85	511	37.64	37.52	37.40	119	3	VEST	EXERCISE2	40	50
	512	37.64	37.53	37.38	119	3	VEST	EXERCISE2	40	50
	513	37.65	37.53	37.38	120	3	VEST	EXERCISE2	40	50
	514	37.65	37.52	37.40	121	3	VEST	EXERCISE2	40	50
	515	37.65	37.53	37.40	122	3	VEST	EXERCISE2	40	50
	516	37.65	37.53	37.37	121	3	VEST	EXERCISE2	40	50
	517	37.66	37.54	37.36	123	3	VEST	EXERCISE2	40	50
	518	37.65	37.54	37.38	126	3	VEST	EXERCISE2	40	50
	519	37.65	37.51	37.38	127	3	VEST	EXERCISE2	40	50
	520	37.65	37.50	37.37	126	3	VEST	EXERCISE2	40	50
	521	37.65	37.50	37.36	127	3	VEST	EXERCISE2	40	50
	522	37.66	37.51	37.37	126	3	VEST	EXERCISE2	40	50
	523	37.65	37.50	37.38	123	3	VEST	EXERCISE2	40	50
	524	37.65	37.49	37.37	122	3	VEST	EXERCISE2	40	50
	525	37.65	37.51	37.38	124	3	VEST	EXERCISE2	40	50
	526	37.65	37.50	37.40	123	3	VEST	EXERCISE2	40	50
	527	37.65	37.50	37.40	123	3	VEST	EXERCISE2	40	50

min	number	Tre	Tes	Tsk-head	HR	ID	condition	period	Ta	RH
	528	37.66	37.50	37.40	123	3	VEST	EXERCISE2	40	50
	529	37.66	37.47	37.39	124	3	VEST	EXERCISE2	40	50
	530	37.66	37.47	37.38	123	3	VEST	EXERCISE2	40	50
	531	37.67	37.48	37.40	124	3	VEST	EXERCISE2	40	50
	532	37.67	37.48	37.41	128	3	VEST	EXERCISE2	40	50
	533	37.68	37.50	37.40	128	3	VEST	EXERCISE2	40	50
	534	37.68	37.47	37.40	119	3	VEST	EXERCISE2	40	50
	535	37.69	37.48	37.41	121	3	VEST	EXERCISE2	40	50
	536	37.69	37.53	37.43	121	3	VEST	EXERCISE2	40	50
	537	37.69	37.53	37.43	123	3	VEST	EXERCISE2	40	50
	538	37.68	37.54	37.42	123	3	VEST	EXERCISE2	40	50
	539	37.67	37.54	37.41	128	3	VEST	EXERCISE2	40	50
	540	37.67	37.53	37.43	130	3	VEST	EXERCISE2	40	50
90	541	37.68	37.54	37.44	127	3	VEST	EXERCISE2	40	50
	542	37.67	37.55	37.44	126	3	VEST	EXERCISE2	40	50
	543	37.67	37.54	37.44	125	3	VEST	EXERCISE2	40	50
	544	37.66	37.54	37.46	122	3	VEST	EXERCISE2	40	50
	545	37.66	37.54	37.44	127	3	VEST	EXERCISE2	40	50
	546	37.67	37.54	37.43	126	3	VEST	EXERCISE2	40	50
	547	37.66	37.54	37.43	126	3	VEST	EXERCISE2	40	50
	548	37.66	37.55	37.43	131	3	VEST	EXERCISE2	40	50
	549	37.67	37.55	37.42	130	3	VEST	EXERCISE2	40	50
	550	37.67	37.56	37.43	126	3	VEST	EXERCISE2	40	50
	551	37.67	37.57	37.44	128	3	VEST	EXERCISE2	40	50
	552	37.67	37.56	37.44	128	3	VEST	EXERCISE2	40	50
	553	37.67	37.56	37.45	129	3	VEST	EXERCISE2	40	50
	554	37.67	37.55	37.45	129	3	VEST	EXERCISE2	40	50
	555	37.67	37.55	37.43	127	3	VEST	EXERCISE2	40	50
	556	37.67	37.57	37.43	127	3	VEST	EXERCISE2	40	50
	557	37.67	37.56	37.44	129	3	VEST	EXERCISE2	40	50
	558	37.68	37.56	37.44	128	3	VEST	EXERCISE2	40	50
	559	37.68	37.57	37.44	129	3	VEST	EXERCISE2	40	50
	560	37.68	37.58	37.43	125	3	VEST	EXERCISE2	40	50
	561	37.68	37.59	37.42	126	3	VEST	EXERCISE2	40	50
	562	37.67	37.58	37.44	125	3	VEST	EXERCISE2	40	50
	563	37.68	37.59	37.44	130	3	VEST	EXERCISE2	40	50
	564	37.68	37.57	37.42	131	3	VEST	EXERCISE2	40	50
	565	37.68	37.55	37.40	133	3	VEST	EXERCISE2	40	50
	566	37.67	37.56	37.38	131	3	VEST	EXERCISE2	40	50
	567	37.67	37.55	37.36	133	3	VEST	EXERCISE2	40	50
	568	37.67	37.53	37.36	151	3	VEST	EXERCISE2	40	50
	569	37.67	37.54	37.34	130	3	VEST	EXERCISE2	40	50
	570	37.67	37.54	37.33	129	3	VEST	EXERCISE2	40	50
95	571	37.67	37.55	37.32	127	3	VEST	EXERCISE2	40	50
	572	37.67	37.56	37.32	127	3	VEST	EXERCISE2	40	50
	573	37.66	37.52	37.32	130	3	VEST	EXERCISE2	40	50

min	number	Tre	Tes	Tsk-head	HR	ID	condition	period	Ta	RH
	574	37.66	37.52	37.30	125	3	VEST	EXERCISE2	40	50
	575	37.67	37.56	37.28	125	3	VEST	EXERCISE2	40	50
	576	37.67	37.56	37.29	128	3	VEST	EXERCISE2	40	50
	577	37.67	37.55	37.27	132	3	VEST	EXERCISE2	40	50
	578	37.67	37.53	37.25	135	3	VEST	EXERCISE2	40	50
	579	37.67	37.52	37.24	134	3	VEST	EXERCISE2	40	50
	580	37.67	37.52	37.25	132	3	VEST	EXERCISE2	40	50
	581	37.67	37.53	37.26	129	3	VEST	EXERCISE2	40	50
	582	37.67	37.54	37.24	131	3	VEST	EXERCISE2	40	50
	583	37.67	37.54	37.23	131	3	VEST	EXERCISE2	40	50
	584	37.67	37.54	37.22	131	3	VEST	EXERCISE2	40	50
	585	37.67	37.55	37.21	130	3	VEST	EXERCISE2	40	50
	586	37.67	37.55	37.20	134	3	VEST	EXERCISE2	40	50
	587	37.67	37.53	37.19	132	3	VEST	EXERCISE2	40	50
	588	37.66	37.50	37.18	133	3	VEST	EXERCISE2	40	50
	589	37.66	37.50	37.19	130	3	VEST	EXERCISE2	40	50
	590	37.65	37.51	37.19	130	3	VEST	EXERCISE2	40	50
	591	37.66	37.51	37.19	127	3	VEST	EXERCISE2	40	50
	592	37.67	37.52	37.18	126	3	VEST	EXERCISE2	40	50
	593	37.66	37.51	37.15	129	3	VEST	EXERCISE2	40	50
	594	37.66	37.51	37.12	129	3	VEST	EXERCISE2	40	50
	595	37.66	37.52	37.11	134	3	VEST	EXERCISE2	40	50
	596	37.66	37.53	37.12	135	3	VEST	EXERCISE2	40	50
	597	37.67	37.53	37.13	131	3	VEST	EXERCISE2	40	50
	598	37.66	37.53	37.11	134	3	VEST	EXERCISE2	40	50
	599	37.66	37.54	37.08	134	3	VEST	EXERCISE2	40	50
	600	37.67	37.55	37.07	135	3	VEST	EXERCISE2	40	50
100	601	37.66	37.56	37.06	134	3	VEST	EXERCISE2	40	50
	602	37.66	37.54	37.05	133	3	VEST	EXERCISE2	40	50
	603	37.66	37.53	37.04	134	3	VEST	EXERCISE2	40	50
	604	37.66	37.53	37.04	135	3	VEST	EXERCISE2	40	50
	605	37.67	37.51	37.05	131	3	VEST	EXERCISE2	40	50
	606	37.68	37.52	37.07	134	3	VEST	EXERCISE2	40	50
	607	37.67	37.51	37.06	135	3	VEST	EXERCISE2	40	50
	608	37.67	37.50	37.03	134	3	VEST	EXERCISE2	40	50
	609	37.68	37.51	37.01	132	3	VEST	EXERCISE2	40	50
	610	37.68	37.51	37.00	131	3	VEST	EXERCISE2	40	50
	611	37.68	37.53	36.99	131	3	VEST	EXERCISE2	40	50
	612	37.68	37.54	36.98	133	3	VEST	EXERCISE2	40	50
	613	37.68	37.54	36.97	131	3	VEST	EXERCISE2	40	50
	614	37.68	37.55	36.96	131	3	VEST	EXERCISE2	40	50
	615	37.69	37.56	36.96	134	3	VEST	EXERCISE2	40	50
	616	37.69	37.56	36.97	137	3	VEST	EXERCISE2	40	50
	617	37.70	37.56	36.99	138	3	VEST	EXERCISE2	40	50
	618	37.70	37.56	36.99	134	3	VEST	EXERCISE2	40	50
	619	37.70	37.56	36.97	131	3	VEST	EXERCISE2	40	50

min	number	Tre	Tes	Tsk-head	HR	ID	condition	period	Ta	RH
	620	37.70	37.57	36.95	134	3	VEST	EXERCISE2	40	50
	621	37.71	37.57	36.96	134	3	VEST	EXERCISE2	40	50
	622	37.70	37.55	36.98	134	3	VEST	EXERCISE2	40	50
	623	37.70	37.56	36.95	132	3	VEST	EXERCISE2	40	50
	624	37.70	37.57	36.94	133	3	VEST	EXERCISE2	40	50
	625	37.70	37.58	36.94	128	3	VEST	EXERCISE2	40	50
	626	37.71	37.57	36.96	131	3	VEST	EXERCISE2	40	50
	627	37.72	37.58	36.99	130	3	VEST	EXERCISE2	40	50
	628	37.71	37.57	36.98	132	3	VEST	EXERCISE2	40	50
	629	37.71	37.54	36.95	137	3	VEST	EXERCISE2	40	50
	630	37.70	37.54	36.96	135	3	VEST	EXERCISE2	40	50
105	631	37.70	37.56	36.98	135	3	VEST	EXERCISE2	40	50
	632	37.71	37.57	36.99	137	3	VEST	EXERCISE2	40	50
	633	37.71	37.57	37.00	133	3	VEST	EXERCISE2	40	50
	634	37.72	37.56	36.99	131	3	VEST	EXERCISE2	40	50
	635	37.72	37.57	36.98	137	3	VEST	EXERCISE2	40	50
	636	37.72	37.57	36.97	139	3	VEST	EXERCISE2	40	50
	637	37.71	37.55	36.95	136	3	VEST	EXERCISE2	40	50
	638	37.71	37.56	36.91	134	3	VEST	EXERCISE2	40	50
	639	37.71	37.57	36.88	138	3	VEST	EXERCISE2	40	50
	640	37.70	37.57	36.87	137	3	VEST	EXERCISE2	40	50
	641	37.70	37.58	36.86	131	3	VEST	EXERCISE2	40	50
	642	37.70	37.58	36.85	132	3	VEST	EXERCISE2	40	50
	643	37.71	37.60	36.86	131	3	VEST	EXERCISE2	40	50
	644	37.71	37.59	36.91	131	3	VEST	REST3	28	50
	645	37.72	37.59	36.54	126	3	VEST	REST3	28	50
	646	37.70	37.60	36.10	124	3	VEST	REST3	28	50
	647	37.69	37.59	35.97	124	3	VEST	REST3	28	50
	648	37.70	37.58	35.87	118	3	VEST	REST3	28	50
	649	37.70	37.55	35.81	122	3	VEST	REST3	28	50
	650	37.70	37.53	35.72	122	3	VEST	REST3	28	50
	651	37.70	37.58	35.67	126	3	VEST	REST3	28	50
	652	37.70	37.54	35.65	129	3	VEST	REST3	28	50
	653	37.70	37.45	35.66	117	3	VEST	REST3	28	50
	654	37.70	37.44	35.66	116	3	VEST	REST3	28	50
	655	37.69	37.37	35.67	116	3	VEST	REST3	28	50
	656	37.69	37.33	35.70	115	3	VEST	REST3	28	50
	657	37.69	37.37	35.74	110	3	VEST	REST3	28	50
	658	37.70	37.35	35.73	113	3	VEST	REST3	28	50
	659	37.70	37.27	35.69	115	3	VEST	REST3	28	50
	660	37.70	37.25	35.71	105	3	VEST	REST3	28	50
110	661	37.70	37.26	35.71	105	3	VEST	REST3	28	50
	662	37.71	37.25	35.59	103	3	VEST	REST3	28	50
	663	37.71	37.21	35.57	111	3	VEST	REST3	28	50
	664	37.71	37.20	35.62	109	3	VEST	REST3	28	50
	665	37.71	37.21	35.55	108	3	VEST	REST3	28	50

min	number	Tre	Tes	Tsk-head	HR	ID	condition	period	Ta	RH
	666	37.71	37.23	35.53	108	3	VEST	REST3	28	50
	667	37.71	37.21	35.55	104	3	VEST	REST3	28	50
	668	37.70	37.18	35.55	103	3	VEST	REST3	28	50
	669	37.69	37.19	35.54	103	3	VEST	REST3	28	50
	670	37.69	37.18	35.49	104	3	VEST	REST3	28	50
	671	37.69	37.18	35.46	101	3	VEST	REST3	28	50
	672	37.69	37.19	35.44	99	3	VEST	REST3	28	50
	673	37.69	37.20	35.39	104	3	VEST	REST3	28	50
	674	37.67	37.20	35.36	104	3	VEST	REST3	28	50
	675	37.67	37.20	35.35	99	3	VEST	REST3	28	50
	676	37.68	37.26	35.32	102	3	VEST	REST3	28	50
	677	37.68	37.37	35.27	99	3	VEST	REST3	28	50
	678	37.68	37.34	35.26	109	3	VEST	REST3	28	50
	679	37.67	37.25	35.22	101	3	VEST	REST3	28	50
	680	37.67	37.25	35.20	96	3	VEST	REST3	28	50
	681	37.66	37.23	35.18	100	3	VEST	REST3	28	50
	682	37.65	37.23	35.14	98	3	VEST	REST3	28	50
	683	37.66	37.27	35.11	99	3	VEST	REST3	28	50
	684	37.67	37.27	35.07	98	3	VEST	REST3	28	50
	685	37.67	37.28	34.97	96	3	VEST	REST3	28	50
	686	37.67	37.31	34.88	99	3	VEST	REST3	28	50
	687	37.67	37.30	34.79	105	3	VEST	REST3	28	50
	688	37.68	37.30	34.74	109	3	VEST	REST3	28	50
	689	37.69	37.33	34.77	106	3	VEST	REST3	28	50
	690	37.69	37.36	34.72	86	3	VEST	REST3	28	50
115	691	37.68	37.36	34.61	95	3	VEST	REST3	28	50
	692	37.67	37.32	34.48	112	3	VEST	REST3	28	50
	693	37.67	37.31	34.50	110	3	VEST	REST3	28	50
	694	37.68	37.34	34.54	110	3	VEST	REST3	28	50
	695	37.68	37.38	34.46	107	3	VEST	REST3	28	50
	696	37.68	37.37	34.45	100	3	VEST	REST3	28	50
	697	37.68	37.36	34.44	101	3	VEST	REST3	28	50
	698	37.68	37.36	34.45	99	3	VEST	REST3	28	50
	699	37.68	37.35	34.34	81	3	VEST	REST3	28	50
	700	37.68	37.36	34.32	90	3	VEST	REST3	28	50
	701	37.68	37.37	34.46	94	3	VEST	REST3	28	50
	702	37.68	37.37	34.51	91	3	VEST	REST3	28	50
	703	37.67	37.38	34.54	91	3	VEST	REST3	28	50
	704	37.67	37.38	34.49	99	3	VEST	REST3	28	50
	705	37.67	37.40	34.44	92	3	VEST	REST3	28	50
	706	37.68	37.41	34.49	88	3	VEST	REST3	28	50
	707	37.68	37.40	34.42	92	3	VEST	REST3	28	50
	708	37.67	37.40	34.40	94	3	VEST	REST3	28	50
	709	37.67	37.41	34.54	88	3	VEST	REST3	28	50
0	1	36.88	36.75	35.18	69	4	VEST	REST1	28	50
	2	36.89	36.73	35.17	74	4	VEST	REST1	28	50

min	number	Tre	Tes	Tsk-head	HR	ID	condition	period	Ta	RH
	3	36.89	36.71	35.18	71	4	VEST	REST1	28	50
	4	36.90	36.74	35.17	71	4	VEST	REST1	28	50
	5	36.91	36.75	35.17	69	4	VEST	REST1	28	50
	6	36.90	36.72	35.17	72	4	VEST	REST1	28	50
	7	36.88	36.72	35.17	70	4	VEST	REST1	28	50
	8	36.88	36.75	35.15	71	4	VEST	REST1	28	50
	9	36.89	36.75	35.15	70	4	VEST	REST1	28	50
	10	36.90	36.72	35.17	73	4	VEST	REST1	28	50
	11	36.90	36.74	35.15	70	4	VEST	REST1	28	50
	12	36.90	36.75	35.15	72	4	VEST	REST1	28	50
	13	36.90	36.75	35.15	71	4	VEST	REST1	28	50
	14	36.90	36.76	35.15	71	4	VEST	REST1	28	50
	15	36.90	36.77	35.13	68	4	VEST	REST1	28	50
	16	36.89	36.77	35.11	69	4	VEST	REST1	28	50
	17	36.89	36.78	35.10	67	4	VEST	REST1	28	50
	18	36.89	36.79	35.10	68	4	VEST	REST1	28	50
	19	36.89	36.76	35.11	71	4	VEST	REST1	28	50
	20	36.89	36.77	35.09	73	4	VEST	REST1	28	50
	21	36.89	36.77	35.09	67	4	VEST	REST1	28	50
	22	36.90	36.77	35.10	69	4	VEST	REST1	28	50
	23	36.89	36.79	35.08	69	4	VEST	REST1	28	50
	24	36.89	36.79	35.08	75	4	VEST	REST1	28	50
	25	36.90	36.78	35.09	68	4	VEST	REST1	28	50
	26	36.90	36.77	35.09	67	4	VEST	REST1	28	50
	27	36.90	36.78	35.07	68	4	VEST	REST1	28	50
	28	36.90	36.79	35.09	67	4	VEST	REST1	28	50
	29	36.92	36.80	35.10	70	4	VEST	REST1	28	50
	30	36.92	36.81	35.07	66	4	VEST	REST1	28	50
5	31	36.92	36.81	35.07	67	4	VEST	REST1	28	50
	32	36.92	36.81	35.07	70	4	VEST	REST1	28	50
	33	36.92	36.81	35.07	68	4	VEST	REST1	28	50
	34	36.92	36.81	35.06	78	4	VEST	REST1	28	50
	35	36.92	36.81	35.05	66	4	VEST	REST1	28	50
	36	36.92	36.83	35.04	69	4	VEST	REST1	28	50
	37	36.92	36.84	35.05	71	4	VEST	REST1	28	50
	38	36.92	36.86	35.08	71	4	VEST	REST1	28	50
	39	36.92	36.85	35.09	68	4	VEST	REST1	28	50
	40	36.92	36.85	35.07	68	4	VEST	REST1	28	50
	41	36.93	36.86	35.05	71	4	VEST	REST1	28	50
	42	36.93	36.87	35.05	71	4	VEST	REST1	28	50
	43	36.93	36.89	35.04	71	4	VEST	REST1	28	50
	44	36.93	36.88	35.04	68	4	VEST	REST1	28	50
	45	36.93	36.87	35.03	68	4	VEST	REST1	28	50
	46	36.92	36.87	35.01	69	4	VEST	REST1	28	50
	47	36.92	36.88	35.01	67	4	VEST	REST1	28	50
	48	36.93	36.88	35.03	71	4	VEST	REST1	28	50

min	number	Tre	Tes	Tsk-head	HR	ID	condition	period	Ta	RH
	49	36.93	36.87	35.04	68	4	VEST	REST1	28	50
	50	36.93	36.87	35.03	70	4	VEST	REST1	28	50
	51	36.93	36.89	35.02	71	4	VEST	REST1	28	50
	52	36.93	36.89	35.01	71	4	VEST	REST1	28	50
	53	36.93	36.87	35.04	67	4	VEST	REST1	28	50
	54	36.93	36.88	35.06	64	4	VEST	REST1	28	50
	55	36.93	36.90	35.06	69	4	VEST	REST1	28	50
	56	36.94	36.89	35.03	74	4	VEST	REST1	28	50
	57	36.94	36.85	35.04	67	4	VEST	REST1	28	50
	58	36.94	36.86	35.06	68	4	VEST	REST1	28	50
	59	36.94	36.87	35.06	70	4	VEST	REST1	28	50
	60	36.94	36.88	35.04	63	4	VEST	REST1	28	50
10	61	36.94	36.89	35.04	66	4	VEST	REST1	28	50
	62	36.93	36.88	35.07	68	4	VEST	REST1	28	50
	63	36.94	36.88	35.07	65	4	VEST	REST1	28	50
	64	36.95	36.88	35.08	68	4	VEST	REST1	28	50
	65	36.95	36.88	35.10	68	4	VEST	REST1	28	50
	66	36.95	36.88	35.11	66	4	VEST	REST1	28	50
	67	36.95	36.89	35.12	66	4	VEST	REST1	28	50
	68	36.95	36.90	35.13	67	4	VEST	REST1	28	50
	69	36.95	36.91	35.11	68	4	VEST	REST1	28	50
	70	36.95	36.92	35.09	68	4	VEST	REST1	28	50
	71	36.95	36.89	35.12	73	4	VEST	REST1	28	50
	72	36.95	36.87	35.13	66	4	VEST	REST1	28	50
	73	36.95	36.89	35.10	67	4	VEST	REST1	28	50
	74	36.95	36.91	35.07	71	4	VEST	REST1	28	50
	75	36.95	36.91	35.07	66	4	VEST	REST1	28	50
	76	36.95	36.92	35.08	68	4	VEST	REST1	28	50
	77	36.95	36.91	35.06	73	4	VEST	REST1	28	50
	78	36.95	36.90	35.08	65	4	VEST	REST1	28	50
	79	36.95	36.87	35.10	80	4	VEST	REST1	28	50
	80	36.95	36.85	35.10	72	4	VEST	REST1	28	50
	81	36.95	36.87	35.12	76	4	VEST	REST1	28	50
	82	36.96	36.86	35.12	87	4	VEST	REST1	28	50
	83	36.96	36.84	35.16	83	4	VEST	REST1	28	50
	84	36.96	36.86	35.18	77	4	VEST	REST1	28	50
	85	36.96	36.88	35.17	74	4	VEST	REST1	28	50
	86	36.95	36.87	35.20	71	4	VEST	REST1	28	50
	87	36.95	36.86	35.19	72	4	VEST	REST1	28	50
	88	36.96	36.88	35.17	72	4	VEST	REST1	28	50
	89	36.96	36.88	35.19	69	4	VEST	REST1	28	50
	90	36.96	36.88	35.18	72	4	VEST	REST1	28	50
15	91	36.96	36.86	35.18	71	4	VEST	REST1	28	50
	92	36.95	36.84	35.21	72	4	VEST	REST1	28	50
	93	36.94	36.87	35.21	69	4	VEST	REST1	28	50
	94	36.94	36.87	35.22	69	4	VEST	REST1	28	50

min	number	Tre	Tes	Tsk-head	HR	ID	condition	period	Ta	RH
	95	36.95	36.88	35.22	71	4	VEST	REST1	28	50
	96	36.95	36.88	35.21	78	4	VEST	REST1	28	50
	97	36.95	36.86	35.23	78	4	VEST	REST1	28	50
	98	36.96	36.85	35.24	74	4	VEST	REST1	28	50
	99	36.96	36.85	35.23	74	4	VEST	REST1	28	50
	100	36.96	36.86	35.23	78	4	VEST	REST1	28	50
	101	36.96	36.85	35.24	74	4	VEST	REST1	28	50
	102	36.97	36.85	35.26	77	4	VEST	REST1	28	50
	103	36.97	36.88	35.28	90	4	VEST	REST1	40	50
	104	36.97	36.88	35.39	94	4	VEST	REST1	40	50
	105	36.97	36.83	35.53	93	4	VEST	REST1	40	50
	106	36.97	36.84	35.61	89	4	VEST	REST1	40	50
	107	36.96	36.82	35.67	90	4	VEST	REST1	40	50
	108	36.95	36.76	35.71	87	4	VEST	REST1	40	50
	109	36.97	36.74	35.76	86	4	VEST	REST1	40	50
	110	36.98	36.73	35.80	87	4	VEST	REST1	40	50
	111	36.97	36.74	35.81	82	4	VEST	REST1	40	50
	112	36.97	36.79	35.83	85	4	VEST	REST1	40	50
	113	36.97	36.78	35.88	87	4	VEST	REST1	40	50
	114	36.97	36.74	35.91	84	4	VEST	REST1	40	50
	115	36.96	36.73	35.91	82	4	VEST	REST1	40	50
	116	36.95	36.72	35.95	86	4	VEST	REST1	40	50
	117	36.96	36.72	35.96	81	4	VEST	REST1	40	50
	118	36.96	36.72	35.97	78	4	VEST	REST1	40	50
	119	36.96	36.72	36.00	84	4	VEST	REST1	40	50
	120	36.96	36.71	35.99	78	4	VEST	REST1	40	50
20	121	36.95	36.71	36.01	77	4	VEST	REST1	40	50
	122	36.96	36.71	36.05	75	4	VEST	REST1	40	50
	123	36.96	36.71	36.07	83	4	VEST	REST1	40	50
	124	36.96	36.71	36.08	79	4	VEST	REST1	40	50
	125	36.96	36.71	36.10	78	4	VEST	REST1	40	50
	126	36.95	36.71	36.10	81	4	VEST	REST1	40	50
	127	36.95	36.71	36.12	79	4	VEST	REST1	40	50
	128	36.96	36.71	36.15	77	4	VEST	REST1	40	50
	129	36.96	36.74	36.15	83	4	VEST	REST1	40	50
	130	36.96	36.75	36.16	80	4	VEST	REST1	40	50
	131	36.96	36.76	36.20	80	4	VEST	REST1	40	50
	132	36.96	36.74	36.24	81	4	VEST	REST1	40	50
	133	36.95	36.75	36.25	87	4	VEST	REST1	40	50
	134	36.96	36.76	36.25	91	4	VEST	REST1	40	50
	135	36.97	36.75	36.26	86	4	VEST	REST1	40	50
	136	36.97	36.78	36.32	83	4	VEST	REST1	40	50
	137	36.96	36.80	36.33	88	4	VEST	REST1	40	50
	138	36.96	36.77	36.31	92	4	VEST	REST1	40	50
	139	36.97	36.76	36.34	84	4	VEST	EXERCISE1	40	50
	140	36.97	36.75	36.39	93	4	VEST	EXERCISE1	40	50

min	number	Tre	Tes	Tsk-head	HR	ID	condition	period	Ta	RH
	141	36.96	36.76	36.41	96	4	VEST	EXERCISE1	40	50
	142	36.96	36.79	36.41	99	4	VEST	EXERCISE1	40	50
	143	36.96	36.74	36.41	105	4	VEST	EXERCISE1	40	50
	144	36.95	36.67	36.43	109	4	VEST	EXERCISE1	40	50
	145	36.95	36.69	36.47	105	4	VEST	EXERCISE1	40	50
	146	36.95	36.72	36.48	105	4	VEST	EXERCISE1	40	50
	147	36.95	36.73	36.49	110	4	VEST	EXERCISE1	40	50
	148	36.94	36.75	36.51	112	4	VEST	EXERCISE1	40	50
	149	36.94	36.76	36.52	116	4	VEST	EXERCISE1	40	50
	150	36.93	36.76	36.52	112	4	VEST	EXERCISE1	40	50
25	151	36.93	36.77	36.53	113	4	VEST	EXERCISE1	40	50
	152	36.93	36.80	36.54	113	4	VEST	EXERCISE1	40	50
	153	36.94	36.82	36.55	112	4	VEST	EXERCISE1	40	50
	154	36.95	36.79	36.59	115	4	VEST	EXERCISE1	40	50
	155	36.96	36.77	36.60	116	4	VEST	EXERCISE1	40	50
	156	36.96	36.79	36.61	114	4	VEST	EXERCISE1	40	50
	157	36.96	36.83	36.62	114	4	VEST	EXERCISE1	40	50
	158	36.97	36.87	36.61	111	4	VEST	EXERCISE1	40	50
	159	36.97	36.87	36.62	112	4	VEST	EXERCISE1	40	50
	160	36.98	36.86	36.66	113	4	VEST	EXERCISE1	40	50
	161	36.98	36.89	36.66	115	4	VEST	EXERCISE1	40	50
	162	36.99	36.92	36.65	115	4	VEST	EXERCISE1	40	50
	163	36.99	36.92	36.67	116	4	VEST	EXERCISE1	40	50
	164	36.99	36.93	36.69	119	4	VEST	EXERCISE1	40	50
	165	37.00	36.95	36.70	118	4	VEST	EXERCISE1	40	50
	166	36.99	36.87	36.69	119	4	VEST	EXERCISE1	40	50
	167	36.99	36.86	36.70	115	4	VEST	EXERCISE1	40	50
	168	37.00	36.97	36.73	115	4	VEST	EXERCISE1	40	50
	169	37.01	37.00	36.75	115	4	VEST	EXERCISE1	40	50
	170	37.02	37.04	36.75	116	4	VEST	EXERCISE1	40	50
	171	37.02	37.06	36.75	116	4	VEST	EXERCISE1	40	50
	172	37.03	37.07	36.77	115	4	VEST	EXERCISE1	40	50
	173	37.04	37.09	36.79	115	4	VEST	EXERCISE1	40	50
	174	37.04	37.10	36.81	118	4	VEST	EXERCISE1	40	50
	175	37.04	37.10	36.82	119	4	VEST	EXERCISE1	40	50
	176	37.03	37.10	36.82	121	4	VEST	EXERCISE1	40	50
	177	37.04	37.14	36.81	118	4	VEST	EXERCISE1	40	50
	178	37.06	37.18	36.84	118	4	VEST	EXERCISE1	40	50
	179	37.07	37.18	36.88	119	4	VEST	EXERCISE1	40	50
	180	37.08	37.19	36.92	118	4	VEST	EXERCISE1	40	50
30	181	37.09	37.20	36.95	121	4	VEST	EXERCISE1	40	50
	182	37.09	37.21	36.98	119	4	VEST	EXERCISE1	40	50
	183	37.10	37.22	36.99	117	4	VEST	EXERCISE1	40	50
	184	37.09	37.25	36.99	116	4	VEST	EXERCISE1	40	50
	185	37.09	37.28	36.98	122	4	VEST	EXERCISE1	40	50
	186	37.10	37.27	37.00	117	4	VEST	EXERCISE1	40	50

min	number	Tre	Tes	Tsk-head	HR	ID	condition	period	Ta	RH
	187	37.11	37.24	37.04	120	4	VEST	EXERCISE1	40	50
	188	37.12	37.26	37.06	119	4	VEST	EXERCISE1	40	50
	189	37.13	37.28	37.08	117	4	VEST	EXERCISE1	40	50
	190	37.14	37.31	37.10	115	4	VEST	EXERCISE1	40	50
	191	37.14	37.32	37.11	116	4	VEST	EXERCISE1	40	50
	192	37.14	37.31	37.14	115	4	VEST	EXERCISE1	40	50
	193	37.14	37.34	37.15	116	4	VEST	EXERCISE1	40	50
	194	37.14	37.35	37.17	115	4	VEST	EXERCISE1	40	50
	195	37.15	37.37	37.16	116	4	VEST	EXERCISE1	40	50
	196	37.16	37.37	37.14	117	4	VEST	EXERCISE1	40	50
	197	37.17	37.37	37.13	117	4	VEST	EXERCISE1	40	50
	198	37.18	37.39	37.14	117	4	VEST	EXERCISE1	40	50
	199	37.19	37.37	37.17	113	4	VEST	EXERCISE1	40	50
	200	37.20	37.38	37.18	117	4	VEST	EXERCISE1	40	50
	201	37.21	37.39	37.18	119	4	VEST	EXERCISE1	40	50
	202	37.21	37.40	37.19	122	4	VEST	EXERCISE1	40	50
	203	37.22	37.42	37.18	123	4	VEST	EXERCISE1	40	50
	204	37.23	37.43	37.21	125	4	VEST	EXERCISE1	40	50
	205	37.23	37.43	37.23	126	4	VEST	EXERCISE1	40	50
	206	37.25	37.41	37.23	125	4	VEST	EXERCISE1	40	50
	207	37.26	37.40	37.25	125	4	VEST	EXERCISE1	40	50
	208	37.26	37.43	37.25	126	4	VEST	EXERCISE1	40	50
	209	37.26	37.43	37.27	126	4	VEST	EXERCISE1	40	50
	210	37.27	37.45	37.29	126	4	VEST	EXERCISE1	40	50
35	211	37.28	37.45	37.28	125	4	VEST	EXERCISE1	40	50
	212	37.28	37.46	37.29	126	4	VEST	EXERCISE1	40	50
	213	37.29	37.46	37.30	126	4	VEST	EXERCISE1	40	50
	214	37.30	37.46	37.28	124	4	VEST	EXERCISE1	40	50
	215	37.30	37.50	37.28	125	4	VEST	EXERCISE1	40	50
	216	37.31	37.49	37.30	127	4	VEST	EXERCISE1	40	50
	217	37.32	37.49	37.29	123	4	VEST	EXERCISE1	40	50
	218	37.32	37.50	37.28	126	4	VEST	EXERCISE1	40	50
	219	37.33	37.51	37.27	125	4	VEST	EXERCISE1	40	50
	220	37.34	37.52	37.27	126	4	VEST	EXERCISE1	40	50
	221	37.34	37.51	37.26	128	4	VEST	EXERCISE1	40	50
	222	37.34	37.52	37.27	128	4	VEST	EXERCISE1	40	50
	223	37.35	37.54	37.27	131	4	VEST	EXERCISE1	40	50
	224	37.36	37.55	37.27	133	4	VEST	EXERCISE1	40	50
	225	37.36	37.55	37.28	128	4	VEST	EXERCISE1	40	50
	226	37.35	37.53	37.27	128	4	VEST	EXERCISE1	40	50
	227	37.36	37.51	37.29	129	4	VEST	EXERCISE1	40	50
	228	37.37	37.55	37.30	127	4	VEST	EXERCISE1	40	50
	229	37.38	37.55	37.30	124	4	VEST	EXERCISE1	40	50
	230	37.39	37.51	37.33	126	4	VEST	EXERCISE1	40	50
	231	37.39	37.52	37.35	121	4	VEST	EXERCISE1	40	50
	232	37.39	37.53	37.34	123	4	VEST	EXERCISE1	40	50

min	number	Tre	Tes	Tsk-head	HR	ID	condition	period	Ta	RH
	233	37.40	37.54	37.33	126	4	VEST	EXERCISE1	40	50
	234	37.41	37.55	37.33	125	4	VEST	EXERCISE1	40	50
	235	37.41	37.56	37.33	126	4	VEST	EXERCISE1	40	50
	236	37.41	37.55	37.34	127	4	VEST	EXERCISE1	40	50
	237	37.43	37.54	37.36	129	4	VEST	EXERCISE1	40	50
	238	37.44	37.57	37.36	124	4	VEST	EXERCISE1	40	50
	239	37.43	37.60	37.35	125	4	VEST	EXERCISE1	40	50
	240	37.43	37.58	37.36	128	4	VEST	EXERCISE1	40	50
40	241	37.44	37.60	37.38	129	4	VEST	EXERCISE1	40	50
	242	37.45	37.61	37.38	127	4	VEST	EXERCISE1	40	50
	243	37.45	37.60	37.39	128	4	VEST	EXERCISE1	40	50
	244	37.46	37.63	37.38	130	4	VEST	EXERCISE1	40	50
	245	37.46	37.61	37.38	132	4	VEST	EXERCISE1	40	50
	246	37.47	37.57	37.40	129	4	VEST	EXERCISE1	40	50
	247	37.47	37.58	37.38	129	4	VEST	EXERCISE1	40	50
	248	37.46	37.58	37.38	125	4	VEST	EXERCISE1	40	50
	249	37.46	37.61	37.40	125	4	VEST	EXERCISE1	40	50
	250	37.47	37.62	37.41	127	4	VEST	EXERCISE1	40	50
	251	37.48	37.62	37.41	126	4	VEST	EXERCISE1	40	50
	252	37.49	37.63	37.39	129	4	VEST	EXERCISE1	40	50
	253	37.49	37.64	37.41	127	4	VEST	EXERCISE1	40	50
	254	37.50	37.62	37.41	126	4	VEST	EXERCISE1	40	50
	255	37.50	37.62	37.38	124	4	VEST	EXERCISE1	40	50
	256	37.51	37.66	37.37	128	4	VEST	EXERCISE1	40	50
	257	37.52	37.68	37.37	126	4	VEST	EXERCISE1	40	50
	258	37.53	37.68	37.38	127	4	VEST	EXERCISE1	40	50
	259	37.53	37.70	37.40	128	4	VEST	EXERCISE1	40	50
	260	37.53	37.68	37.43	127	4	VEST	EXERCISE1	40	50
	261	37.53	37.66	37.42	128	4	VEST	EXERCISE1	40	50
	262	37.54	37.68	37.42	130	4	VEST	EXERCISE1	40	50
	263	37.55	37.68	37.45	132	4	VEST	EXERCISE1	40	50
	264	37.55	37.69	37.44	131	4	VEST	EXERCISE1	40	50
	265	37.55	37.70	37.44	133	4	VEST	EXERCISE1	40	50
	266	37.56	37.68	37.46	129	4	VEST	EXERCISE1	40	50
	267	37.57	37.70	37.46	126	4	VEST	EXERCISE1	40	50
	268	37.58	37.70	37.46	132	4	VEST	EXERCISE1	40	50
	269	37.59	37.69	37.46	132	4	VEST	EXERCISE1	40	50
	270	37.59	37.69	37.46	132	4	VEST	EXERCISE1	40	50
45	271	37.58	37.68	37.48	130	4	VEST	EXERCISE1	40	50
	272	37.59	37.71	37.46	130	4	VEST	EXERCISE1	40	50
	273	37.59	37.73	37.47	133	4	VEST	EXERCISE1	40	50
	274	37.59	37.72	37.48	129	4	VEST	EXERCISE1	40	50
	275	37.60	37.70	37.48	130	4	VEST	EXERCISE1	40	50
	276	37.60	37.72	37.48	132	4	VEST	EXERCISE1	40	50
	277	37.61	37.72	37.48	133	4	VEST	EXERCISE1	40	50
	278	37.62	37.72	37.49	134	4	VEST	EXERCISE1	40	50

min	number	Tre	Tes	Tsk-head	HR	ID	condition	period	Ta	RH
	279	37.63	37.74	37.50	135	4	VEST	EXERCISE1	40	50
	280	37.63	37.73	37.49	136	4	VEST	EXERCISE1	40	50
	281	37.64	37.72	37.50	136	4	VEST	EXERCISE1	40	50
	282	37.64	37.72	37.51	136	4	VEST	EXERCISE1	40	50
	283	37.64	37.74	37.52	133	4	VEST	EXERCISE1	40	50
	284	37.65	37.75	37.54	135	4	VEST	EXERCISE1	40	50
	285	37.66	37.76	37.53	150	4	VEST	EXERCISE1	40	50
	286	37.67	37.79	37.52	134	4	VEST	EXERCISE1	40	50
	287	37.67	37.79	37.51	135	4	VEST	EXERCISE1	40	50
	288	37.67	37.77	37.51	132	4	VEST	EXERCISE1	40	50
	289	37.68	37.79	37.52	132	4	VEST	EXERCISE1	40	50
	290	37.68	37.75	37.53	134	4	VEST	EXERCISE1	40	50
	291	37.68	37.72	37.54	130	4	VEST	EXERCISE1	40	50
	292	37.68	37.75	37.55	135	4	VEST	EXERCISE1	40	50
	293	37.68	37.75	37.55	134	4	VEST	EXERCISE1	40	50
	294	37.68	37.75	37.55	134	4	VEST	EXERCISE1	40	50
	295	37.69	37.76	37.53	133	4	VEST	EXERCISE1	40	50
	296	37.69	37.78	37.54	131	4	VEST	EXERCISE1	40	50
	297	37.69	37.77	37.55	134	4	VEST	EXERCISE1	40	50
	298	37.70	37.79	37.55	137	4	VEST	EXERCISE1	40	50
	299	37.71	37.80	37.57	138	4	VEST	EXERCISE1	40	50
	300	37.71	37.77	37.57	137	4	VEST	EXERCISE1	40	50
50	301	37.71	37.79	37.56	139	4	VEST	EXERCISE1	40	50
	302	37.72	37.81	37.55	137	4	VEST	EXERCISE1	40	50
	303	37.72	37.80	37.54	135	4	VEST	EXERCISE1	40	50
	304	37.73	37.81	37.56	135	4	VEST	EXERCISE1	40	50
	305	37.73	37.81	37.59	134	4	VEST	EXERCISE1	40	50
	306	37.74	37.80	37.58	128	4	VEST	EXERCISE1	40	50
	307	37.75	37.81	37.56	133	4	VEST	EXERCISE1	40	50
	308	37.76	37.82	37.56	135	4	VEST	EXERCISE1	40	50
	309	37.77	37.82	37.56	136	4	VEST	EXERCISE1	40	50
	310	37.77	37.83	37.56	140	4	VEST	EXERCISE1	40	50
	311	37.78	37.85	37.58	138	4	VEST	EXERCISE1	40	50
	312	37.78	37.84	37.61	137	4	VEST	EXERCISE1	40	50
	313	37.78	37.85	37.60	137	4	VEST	EXERCISE1	40	50
	314	37.79	37.85	37.58	132	4	VEST	EXERCISE1	40	50
	315	37.79	37.85	37.56	131	4	VEST	EXERCISE1	40	50
	316	37.81	37.88	37.56	132	4	VEST	EXERCISE1	40	50
	317	37.81	37.65	37.58	133	4	VEST	EXERCISE1	40	50
	318	37.81	37.64	37.59	134	4	VEST	EXERCISE1	40	50
	319	37.82	37.85	37.59	133	4	VEST	EXERCISE1	40	50
	320	37.82	37.87	37.59	129	4	VEST	REST2	28	50
	321	37.82	37.88	37.60	127	4	VEST	REST2	28	50
	322	37.83	37.86	37.60	125	4	VEST	REST2	28	50
	323	37.84	37.90	37.49	124	4	VEST	REST2	28	50
	324	37.85	37.90	37.33	120	4	VEST	REST2	28	50

min	number	Tre	Tes	Tsk-head	HR	ID	condition	period	Ta	RH
	325	37.84	37.87	37.23	116	4	VEST	REST2	28	50
	326	37.83	37.89	37.18	116	4	VEST	REST2	28	50
	327	37.84	37.93	37.10	116	4	VEST	REST2	28	50
	328	37.85	37.93	37.05	116	4	VEST	REST2	28	50
	329	37.85	37.83	37.03	114	4	VEST	REST2	28	50
	330	37.85	33.58	37.00	115	4	VEST	REST2	28	50
55	331	37.85	28.71	37.01	112	4	VEST	REST2	28	50
	332	37.85	28.51	37.03	116	4	VEST	REST2	28	50
	333	37.85	28.55	37.03	114	4	VEST	REST2	28	50
	334	37.86	28.17	37.01	113	4	VEST	REST2	28	50
	335	37.86	27.98	36.98	111	4	VEST	REST2	28	50
	336	37.86	28.02	36.96	110	4	VEST	REST2	28	50
	337	37.86	29.20	36.95	106	4	VEST	REST2	28	50
	338	37.86	30.42	36.91	102	4	VEST	REST2	28	50
	339	37.86	30.98	36.87	102	4	VEST	REST2	28	50
	340	37.87	31.61	36.82	101	4	VEST	REST2	28	50
	341	37.87	32.56	36.76	106	4	VEST	REST2	28	50
	342	37.87	33.28	36.73	101	4	VEST	REST2	28	50
	343	37.86	33.80	36.73	102	4	VEST	REST2	28	50
	344	37.86	34.24	36.69	106	4	VEST	REST2	28	50
	345	37.85	34.57	36.62	103	4	VEST	REST2	28	50
	346	37.85	34.86	36.61	104	4	VEST	REST2	28	50
	347	37.84	35.02	36.62	103	4	VEST	REST2	28	50
	348	37.83	35.22	36.60	102	4	VEST	REST2	28	50
	349	37.82	35.50	36.55	107	4	VEST	REST2	28	50
	350	37.82	35.72	36.52	103	4	VEST	REST2	28	50
	351	37.83	35.78	36.53	103	4	VEST	REST2	28	50
	352	37.83	35.88	36.52	101	4	VEST	REST2	28	50
	353	37.83	36.01	36.48	104	4	VEST	REST2	28	50
	354	37.83	36.10	36.46	100	4	VEST	REST2	28	50
	355	37.82	36.17	36.46	99	4	VEST	REST2	28	50
	356	37.82	36.25	36.46	98	4	VEST	REST2	28	50
	357	37.82	36.32	36.42	98	4	VEST	REST2	28	50
	358	37.81	36.39	36.39	98	4	VEST	REST2	28	50
	359	37.81	36.44	36.38	98	4	VEST	REST2	28	50
	360	37.81	36.49	36.33	94	4	VEST	REST2	28	50
60	361	37.80	36.54	36.30	93	4	VEST	REST2	28	50
	362	37.80	36.58	36.29	92	4	VEST	REST2	28	50
	363	37.79	36.60	36.28	92	4	VEST	REST2	28	50
	364	37.79	36.61	36.27	93	4	VEST	REST2	28	50
	365	37.79	36.65	36.23	93	4	VEST	REST2	28	50
	366	37.78	36.68	36.20	92	4	VEST	REST2	28	50
	367	37.77	36.69	36.20	89	4	VEST	REST2	28	50
	368	37.77	36.71	36.19	90	4	VEST	REST2	28	50
	369	37.76	36.74	36.16	88	4	VEST	REST2	28	50
	370	37.75	36.74	36.12	89	4	VEST	REST2	28	50

min	number	Tre	Tes	Tsk-head	HR	ID	condition	period	Ta	RH
	371	37.75	36.74	36.11	89	4	VEST	REST2	28	50
	372	37.75	36.77	36.07	88	4	VEST	REST2	28	50
	373	37.75	36.78	36.05	89	4	VEST	REST2	28	50
	374	37.74	36.81	36.03	89	4	VEST	REST2	28	50
	375	37.72	36.81	35.98	88	4	VEST	REST2	28	50
	376	37.71	36.80	35.97	89	4	VEST	REST2	28	50
	377	37.70	36.82	35.98	89	4	VEST	REST2	28	50
	378	37.70	36.85	35.97	89	4	VEST	REST2	28	50
	379	37.69	36.88	35.93	89	4	VEST	REST2	28	50
	380	37.69	36.90	35.89	92	4	VEST	REST2	28	50
	381	37.69	36.89	35.88	88	4	VEST	REST2	28	50
	382	37.69	36.88	35.89	88	4	VEST	REST2	28	50
	383	37.69	36.89	35.87	89	4	VEST	REST2	28	50
	384	37.69	36.91	35.85	86	4	VEST	REST2	28	50
	385	37.69	36.92	35.85	87	4	VEST	REST2	28	50
	386	37.68	36.92	35.84	87	4	VEST	REST2	28	50
	387	37.68	36.94	35.81	88	4	VEST	REST2	28	50
	388	37.67	36.94	35.78	91	4	VEST	REST2	28	50
	389	37.68	36.92	35.78	95	4	VEST	REST2	28	50
	390	37.68	36.91	35.78	90	4	VEST	REST2	28	50
65	391	37.67	36.94	35.77	88	4	VEST	REST2	28	50
	392	37.68	36.98	35.75	88	4	VEST	REST2	28	50
	393	37.67	37.00	35.74	88	4	VEST	REST2	28	50
	394	37.67	37.01	35.73	87	4	VEST	REST2	28	50
	395	37.66	37.01	35.72	85	4	VEST	REST2	28	50
	396	37.65	37.02	35.69	86	4	VEST	REST2	28	50
	397	37.66	37.04	35.66	84	4	VEST	REST2	28	50
	398	37.66	37.04	35.68	85	4	VEST	REST2	28	50
	399	37.66	37.04	35.70	87	4	VEST	REST2	28	50
	400	37.65	37.04	35.67	85	4	VEST	REST2	28	50
	401	37.64	37.06	35.63	82	4	VEST	REST2	28	50
	402	37.65	37.08	35.63	83	4	VEST	REST2	28	50
	403	37.65	37.07	35.65	81	4	VEST	REST2	28	50
	404	37.65	37.06	35.65	82	4	VEST	REST2	28	50
	405	37.64	37.08	35.63	83	4	VEST	REST2	28	50
	406	37.64	37.10	35.59	83	4	VEST	REST2	28	50
	407	37.64	37.10	35.56	86	4	VEST	REST2	28	50
	408	37.63	37.09	35.56	86	4	VEST	REST2	28	50
	409	37.63	37.10	35.56	82	4	VEST	REST2	28	50
	410	37.63	37.12	35.56	87	4	VEST	REST2	28	50
	411	37.63	37.13	35.56	89	4	VEST	REST2	28	50
	412	37.63	37.13	35.55	88	4	VEST	REST2	28	50
	413	37.63	37.13	35.56	89	4	VEST	REST2	28	50
	414	37.63	37.12	35.59	90	4	VEST	REST2	28	50
	415	37.63	37.13	35.60	90	4	VEST	REST2	28	50
	416	37.63	37.14	35.58	91	4	VEST	REST2	28	50

min	number	Tre	Tes	Tsk-head	HR	ID	condition	period	Ta	RH
	417	37.63	37.16	35.56	88	4	VEST	REST2	28	50
	418	37.63	37.17	35.59	86	4	VEST	REST2	28	50
	419	37.63	37.17	35.60	84	4	VEST	REST2	28	50
	420	37.62	37.17	35.59	86	4	VEST	REST2	28	50
70	421	37.62	37.19	35.58	85	4	VEST	REST2	28	50
	422	37.62	37.20	35.56	84	4	VEST	REST2	28	50
	423	37.62	37.20	35.57	82	4	VEST	REST2	28	50
	424	37.62	37.19	35.59	81	4	VEST	REST2	28	50
	425	37.62	37.17	35.60	83	4	VEST	REST2	28	50
	426	37.62	37.17	35.61	83	4	VEST	REST2	28	50
	427	37.62	37.18	35.60	88	4	VEST	REST2	28	50
	428	37.62	37.15	35.55	93	4	VEST	REST2	28	50
	429	37.61	37.14	35.55	88	4	VEST	REST2	28	50
	430	37.61	37.16	35.58	89	4	VEST	REST2	28	50
	431	37.61	37.08	35.59	88	4	VEST	REST2	28	50
	432	37.61	37.09	35.61	87	4	VEST	REST2	28	50
	433	37.60	37.17	35.64	88	4	VEST	REST2	28	50
	434	37.60	37.16	35.67	89	4	VEST	REST2	28	50
	435	37.60	37.18	35.64	92	4	VEST	REST2	28	50
	436	37.60	37.19	35.58	95	4	VEST	REST2	28	50
	437	37.60	37.17	35.58	91	4	VEST	REST2	28	50
	438	37.60	37.16	35.59	90	4	VEST	REST2	28	50
	439	37.60	37.16	35.59	91	4	VEST	REST2	40	50
	440	37.59	37.15	35.67	98	4	VEST	REST2	40	50
	441	37.59	37.14	35.79	94	4	VEST	REST2	40	50
	442	37.61	37.08	35.88	95	4	VEST	REST2	40	50
	443	37.62	37.09	35.92	87	4	VEST	REST2	40	50
	444	37.62	37.12	35.95	85	4	VEST	REST2	40	50
	445	37.61	37.10	36.00	84	4	VEST	REST2	40	50
	446	37.61	37.10	36.05	87	4	VEST	REST2	40	50
	447	37.60	37.10	36.07	87	4	VEST	REST2	40	50
	448	37.60	37.11	36.08	83	4	VEST	REST2	40	50
	449	37.60	37.12	36.09	86	4	VEST	REST2	40	50
	450	37.60	37.12	36.10	82	4	VEST	REST2	40	50
75	451	37.59	37.15	36.14	87	4	VEST	REST2	40	50
	452	37.59	37.14	36.18	84	4	VEST	REST2	40	50
	453	37.60	37.13	36.19	84	4	VEST	REST2	40	50
	454	37.59	37.11	36.21	84	4	VEST	REST2	40	50
	455	37.59	37.10	36.23	85	4	VEST	REST2	40	50
	456	37.60	37.12	36.23	85	4	VEST	REST2	40	50
	457	37.60	37.12	36.25	97	4	VEST	REST2	40	50
	458	37.60	37.13	36.28	101	4	VEST	REST2	40	50
	459	37.60	36.95	36.31	88	4	VEST	REST2	40	50
	460	37.59	36.91	36.30	92	4	VEST	REST2	40	50
	461	37.59	37.04	36.31	95	4	VEST	REST2	40	50
	462	37.59	37.07	36.35	86	4	VEST	REST2	40	50

min	number	Tre	Tes	Tsk-head	HR	ID	condition	period	Ta	RH
	463	37.58	37.09	36.35	88	4	VEST	EXERCISE2	40	50
	464	37.58	37.07	36.37	98	4	VEST	EXERCISE2	40	50
	465	37.58	37.04	36.38	105	4	VEST	EXERCISE2	40	50
	466	37.57	37.05	36.37	106	4	VEST	EXERCISE2	40	50
	467	37.57	37.07	36.39	111	4	VEST	EXERCISE2	40	50
	468	37.56	37.08	36.40	114	4	VEST	EXERCISE2	40	50
	469	37.56	37.09	36.43	107	4	VEST	EXERCISE2	40	50
	470	37.56	37.08	36.46	120	4	VEST	EXERCISE2	40	50
	471	37.56	37.09	36.48	141	4	VEST	EXERCISE2	40	50
	472	37.57	37.07	36.49	124	4	VEST	EXERCISE2	40	50
	473	37.56	37.05	36.51	121	4	VEST	EXERCISE2	40	50
	474	37.56	37.10	36.53	137	4	VEST	EXERCISE2	40	50
	475	37.57	37.14	36.53	123	4	VEST	EXERCISE2	40	50
	476	37.57	37.16	36.57	121	4	VEST	EXERCISE2	40	50
	477	37.57	37.17	36.61	114	4	VEST	EXERCISE2	40	50
	478	37.57	37.18	36.62	121	4	VEST	EXERCISE2	40	50
	479	37.57	37.21	36.62	126	4	VEST	EXERCISE2	40	50
	480	37.58	37.22	36.65	126	4	VEST	EXERCISE2	40	50
80	481	37.58	37.24	36.68	124	4	VEST	EXERCISE2	40	50
	482	37.59	37.26	36.71	125	4	VEST	EXERCISE2	40	50
	483	37.59	37.26	36.77	126	4	VEST	EXERCISE2	40	50
	484	37.59	37.27	36.81	130	4	VEST	EXERCISE2	40	50
	485	37.60	37.28	36.85	132	4	VEST	EXERCISE2	40	50
	486	37.61	37.32	36.88	130	4	VEST	EXERCISE2	40	50
	487	37.62	37.34	36.94	128	4	VEST	EXERCISE2	40	50
	488	37.62	37.34	36.99	125	4	VEST	EXERCISE2	40	50
	489	37.62	37.34	37.03	127	4	VEST	EXERCISE2	40	50
	490	37.62	37.35	37.06	127	4	VEST	EXERCISE2	40	50
	491	37.62	37.37	37.08	128	4	VEST	EXERCISE2	40	50
	492	37.63	37.39	37.09	127	4	VEST	EXERCISE2	40	50
	493	37.63	37.41	37.10	127	4	VEST	EXERCISE2	40	50
	494	37.64	37.42	37.13	128	4	VEST	EXERCISE2	40	50
	495	37.64	37.42	37.16	130	4	VEST	EXERCISE2	40	50
	496	37.64	37.44	37.16	130	4	VEST	EXERCISE2	40	50
	497	37.65	37.45	37.17	131	4	VEST	EXERCISE2	40	50
	498	37.65	37.45	37.21	132	4	VEST	EXERCISE2	40	50
	499	37.66	37.45	37.22	132	4	VEST	EXERCISE2	40	50
	500	37.67	37.48	37.22	132	4	VEST	EXERCISE2	40	50
	501	37.67	37.49	37.24	133	4	VEST	EXERCISE2	40	50
	502	37.67	37.47	37.27	135	4	VEST	EXERCISE2	40	50
	503	37.67	37.48	37.26	139	4	VEST	EXERCISE2	40	50
	504	37.67	37.50	37.25	139	4	VEST	EXERCISE2	40	50
	505	37.68	37.51	37.28	140	4	VEST	EXERCISE2	40	50
	506	37.68	37.50	37.31	142	4	VEST	EXERCISE2	40	50
	507	37.67	37.50	37.32	141	4	VEST	EXERCISE2	40	50
	508	37.67	37.51	37.32	140	4	VEST	EXERCISE2	40	50

min	number	Tre	Tes	Tsk-head	HR	ID	condition	period	Ta	RH
	509	37.67	37.53	37.32	139	4	VEST	EXERCISE2	40	50
	510	37.67	37.54	37.33	139	4	VEST	EXERCISE2	40	50
85	511	37.67	37.54	37.34	136	4	VEST	EXERCISE2	40	50
	512	37.68	37.54	37.35	136	4	VEST	EXERCISE2	40	50
	513	37.69	37.56	37.35	140	4	VEST	EXERCISE2	40	50
	514	37.69	37.58	37.35	138	4	VEST	EXERCISE2	40	50
	515	37.69	37.57	37.38	141	4	VEST	EXERCISE2	40	50
	516	37.69	37.58	37.38	139	4	VEST	EXERCISE2	40	50
	517	37.70	37.58	37.39	139	4	VEST	EXERCISE2	40	50
	518	37.70	37.58	37.40	142	4	VEST	EXERCISE2	40	50
	519	37.70	37.60	37.40	140	4	VEST	EXERCISE2	40	50
	520	37.71	37.61	37.41	137	4	VEST	EXERCISE2	40	50
	521	37.70	37.62	37.41	140	4	VEST	EXERCISE2	40	50
	522	37.70	37.61	37.44	139	4	VEST	EXERCISE2	40	50
	523	37.71	37.63	37.42	133	4	VEST	EXERCISE2	40	50
	524	37.72	37.63	37.43	129	4	VEST	EXERCISE2	40	50
	525	37.73	37.63	37.44	130	4	VEST	EXERCISE2	40	50
	526	37.74	37.46	37.41	127	4	VEST	EXERCISE2	40	50
	527	37.75	37.44	37.44	130	4	VEST	EXERCISE2	40	50
	528	37.75	37.63	37.45	130	4	VEST	EXERCISE2	40	50
	529	37.74	37.46	37.44	138	4	VEST	EXERCISE2	40	50
	530	37.74	37.42	37.46	143	4	VEST	EXERCISE2	40	50
	531	37.75	37.59	37.48	149	4	VEST	EXERCISE2	40	50
	532	37.76	37.61	37.50	147	4	VEST	EXERCISE2	40	50
	533	37.77	37.62	37.51	147	4	VEST	EXERCISE2	40	50
	534	37.77	37.63	37.52	148	4	VEST	EXERCISE2	40	50
	535	37.78	37.65	37.53	144	4	VEST	EXERCISE2	40	50
	536	37.78	37.67	37.52	143	4	VEST	EXERCISE2	40	50
	537	37.78	37.69	37.50	143	4	VEST	EXERCISE2	40	50
	538	37.78	37.69	37.50	143	4	VEST	EXERCISE2	40	50
	539	37.79	37.70	37.50	144	4	VEST	EXERCISE2	40	50
	540	37.79	37.62	37.52	145	4	VEST	EXERCISE2	40	50
90	541	37.79	37.58	37.56	142	4	VEST	EXERCISE2	40	50
	542	37.79	37.65	37.57	143	4	VEST	EXERCISE2	40	50
	543	37.80	37.69	37.55	142	4	VEST	EXERCISE2	40	50
	544	37.81	37.70	37.56	140	4	VEST	EXERCISE2	40	50
	545	37.83	37.70	37.59	139	4	VEST	EXERCISE2	40	50
	546	37.82	37.70	37.59	142	4	VEST	EXERCISE2	40	50
	547	37.82	37.72	37.58	143	4	VEST	EXERCISE2	40	50
	548	37.82	37.75	37.56	142	4	VEST	EXERCISE2	40	50
	549	37.82	37.77	37.57	143	4	VEST	EXERCISE2	40	50
	550	37.84	37.76	37.60	146	4	VEST	EXERCISE2	40	50
	551	37.85	37.75	37.62	146	4	VEST	EXERCISE2	40	50
	552	37.86	37.75	37.63	148	4	VEST	EXERCISE2	40	50
	553	37.86	37.76	37.63	147	4	VEST	EXERCISE2	40	50
	554	37.86	37.77	37.62	146	4	VEST	EXERCISE2	40	50

min	number	Tre	Tes	Tsk-head	HR	ID	condition	period	Ta	RH
	555	37.86	37.79	37.62	146	4	VEST	EXERCISE2	40	50
	556	37.86	37.82	37.60	145	4	VEST	EXERCISE2	40	50
	557	37.87	37.82	37.58	148	4	VEST	EXERCISE2	40	50
	558	37.87	37.79	37.61	145	4	VEST	EXERCISE2	40	50
	559	37.87	37.78	37.64	145	4	VEST	EXERCISE2	40	50
	560	37.88	37.80	37.63	145	4	VEST	EXERCISE2	40	50
	561	37.89	37.82	37.62	144	4	VEST	EXERCISE2	40	50
	562	37.89	37.79	37.65	145	4	VEST	EXERCISE2	40	50
	563	37.90	37.77	37.65	145	4	VEST	EXERCISE2	40	50
	564	37.89	37.81	37.64	146	4	VEST	EXERCISE2	40	50
	565	37.89	37.82	37.65	146	4	VEST	EXERCISE2	40	50
	566	37.90	37.82	37.65	144	4	VEST	EXERCISE2	40	50
	567	37.90	37.85	37.62	145	4	VEST	EXERCISE2	40	50
	568	37.90	37.84	37.61	149	4	VEST	EXERCISE2	40	50
	569	37.91	37.83	37.61	151	4	VEST	EXERCISE2	40	50
	570	37.91	37.86	37.62	151	4	VEST	EXERCISE2	40	50
95	571	37.91	37.85	37.63	149	4	VEST	EXERCISE2	40	50
	572	37.91	37.85	37.63	149	4	VEST	EXERCISE2	40	50
	573	37.91	37.89	37.63	149	4	VEST	EXERCISE2	40	50
	574	37.93	37.90	37.64	150	4	VEST	EXERCISE2	40	50
	575	37.95	37.91	37.65	152	4	VEST	EXERCISE2	40	50
	576	37.95	37.91	37.66	148	4	VEST	EXERCISE2	40	50
	577	37.95	37.89	37.68	149	4	VEST	EXERCISE2	40	50
	578	37.95	37.88	37.68	148	4	VEST	EXERCISE2	40	50
	579	37.95	37.90	37.68	149	4	VEST	EXERCISE2	40	50
	580	37.96	37.86	37.68	144	4	VEST	EXERCISE2	40	50
	581	37.96	37.80	37.69	138	4	VEST	EXERCISE2	40	50
	582	37.97	37.83	37.69	139	4	VEST	EXERCISE2	40	50
	583	37.98	37.85	37.68	137	4	VEST	EXERCISE2	40	50
	584	37.98	37.85	37.67	143	4	VEST	EXERCISE2	40	50
	585	37.97	37.87	37.66	148	4	VEST	EXERCISE2	40	50
	586	37.98	37.90	37.68	148	4	VEST	EXERCISE2	40	50
	587	37.98	37.91	37.69	152	4	VEST	EXERCISE2	40	50
	588	37.99	37.90	37.68	149	4	VEST	EXERCISE2	40	50
	589	37.99	37.91	37.68	146	4	VEST	EXERCISE2	40	50
	590	38.00	37.91	37.69	142	4	VEST	EXERCISE2	40	50
	591	38.01	37.89	37.70	141	4	VEST	EXERCISE2	40	50
	592	38.01	37.90	37.70	144	4	VEST	EXERCISE2	40	50
	593	38.01	37.91	37.72	148	4	VEST	EXERCISE2	40	50
	594	38.02	37.89	37.75	146	4	VEST	EXERCISE2	40	50
	595	38.01	37.90	37.73	148	4	VEST	EXERCISE2	40	50
	596	38.01	37.91	37.71	148	4	VEST	EXERCISE2	40	50
	597	38.00	37.90	37.73	149	4	VEST	EXERCISE2	40	50
	598	38.00	37.81	37.72	149	4	VEST	EXERCISE2	40	50
	599	38.00	37.77	37.73	146	4	VEST	EXERCISE2	40	50
	600	38.02	37.85	37.75	148	4	VEST	EXERCISE2	40	50

min	number	Tre	Tes	Tsk-head	HR	ID	condition	period	Ta	RH
100	601	38.02	37.88	37.72	150	4	VEST	EXERCISE2	40	50
	602	38.02	37.90	37.71	149	4	VEST	EXERCISE2	40	50
	603	38.03	37.91	37.74	148	4	VEST	EXERCISE2	40	50
	604	38.04	37.92	37.75	147	4	VEST	EXERCISE2	40	50
	605	38.05	37.93	37.75	150	4	VEST	EXERCISE2	40	50
	606	38.06	37.94	37.75	149	4	VEST	EXERCISE2	40	50
	607	38.06	37.86	37.76	144	4	VEST	EXERCISE2	40	50
	608	38.06	37.86	37.75	149	4	VEST	EXERCISE2	40	50
	609	38.06	37.93	37.75	150	4	VEST	EXERCISE2	40	50
	610	38.07	37.92	37.77	147	4	VEST	EXERCISE2	40	50
	611	38.07	37.91	37.79	146	4	VEST	EXERCISE2	40	50
	612	38.08	37.93	37.80	149	4	VEST	EXERCISE2	40	50
	613	38.08	37.94	37.78	154	4	VEST	EXERCISE2	40	50
	614	38.06	37.91	37.79	158	4	VEST	EXERCISE2	40	50
	615	38.06	37.92	37.81	154	4	VEST	EXERCISE2	40	50
	616	38.07	37.96	37.78	153	4	VEST	EXERCISE2	40	50
	617	38.08	37.97	37.79	155	4	VEST	EXERCISE2	40	50
	618	38.09	37.97	37.81	155	4	VEST	EXERCISE2	40	50
	619	38.09	37.98	37.79	154	4	VEST	EXERCISE2	40	50
	620	38.11	37.99	37.78	152	4	VEST	EXERCISE2	40	50
	621	38.11	37.99	37.79	152	4	VEST	EXERCISE2	40	50
	622	38.10	37.99	37.80	153	4	VEST	EXERCISE2	40	50
	623	38.11	38.01	37.80	150	4	VEST	EXERCISE2	40	50
	624	38.11	38.01	37.80	153	4	VEST	EXERCISE2	40	50
	625	38.12	38.01	37.82	154	4	VEST	EXERCISE2	40	50
	626	38.12	38.01	37.81	153	4	VEST	EXERCISE2	40	50
	627	38.12	38.00	37.81	147	4	VEST	EXERCISE2	40	50
	628	38.12	38.01	37.82	144	4	VEST	EXERCISE2	40	50
	629	38.12	37.77	37.81	144	4	VEST	EXERCISE2	40	50
	630	38.12	37.76	37.82	147	4	VEST	EXERCISE2	40	50
105	631	38.12	37.98	37.82	146	4	VEST	EXERCISE2	40	50
	632	38.13	37.98	37.83	146	4	VEST	EXERCISE2	40	50
	633	38.14	38.01	37.82	146	4	VEST	EXERCISE2	40	50
	634	38.14	38.00	37.80	145	4	VEST	EXERCISE2	40	50
	635	38.13	38.00	37.81	144	4	VEST	EXERCISE2	40	50
	636	38.14	38.01	37.83	144	4	VEST	EXERCISE2	40	50
	637	38.13	37.99	37.84	142	4	VEST	EXERCISE2	40	50
	638	38.14	38.01	37.82	142	4	VEST	EXERCISE2	40	50
	639	38.15	38.01	37.83	144	4	VEST	EXERCISE2	40	50
	640	38.16	37.97	37.84	148	4	VEST	EXERCISE2	40	50
	641	38.16	37.97	37.83	146	4	VEST	EXERCISE2	40	50
	642	38.17	37.99	37.85	146	4	VEST	EXERCISE2	40	50
	643	38.17	37.99	37.83	144	4	VEST	EXERCISE2	40	50
	644	38.17	38.02	37.81	139	4	VEST	REST3	28	50
	645	38.18	38.03	37.83	159	4	VEST	REST3	28	50
	646	38.18	38.04	37.72	134	4	VEST	REST3	28	50

min	number	Tre	Tes	Tsk-head	HR	ID	condition	period	Ta	RH
	647	38.18	38.08	37.56	131	4	VEST	REST3	28	50
	648	38.18	38.12	37.48	127	4	VEST	REST3	28	50
	649	38.19	38.11	37.43	131	4	VEST	REST3	28	50
	650	38.20	38.09	37.36	149	4	VEST	REST3	28	50
	651	38.19	38.09	37.32	131	4	VEST	REST3	28	50
	652	38.19	38.10	37.28	124	4	VEST	REST3	28	50
	653	38.19	38.05	37.26	121	4	VEST	REST3	28	50
	654	38.20	38.03	37.25	119	4	VEST	REST3	28	50
	655	38.20	38.04	37.19	115	4	VEST	REST3	28	50
	656	38.19	38.04	37.13	116	4	VEST	REST3	28	50
	657	38.19	37.96	37.10	124	4	VEST	REST3	28	50
	658	38.19	37.94	37.08	122	4	VEST	REST3	28	50
	659	38.19	37.92	37.02	121	4	VEST	REST3	28	50
	660	38.19	37.84	36.96	118	4	VEST	REST3	28	50
110	661	38.19	37.84	36.96	117	4	VEST	REST3	28	50
	662	38.19	37.82	36.94	117	4	VEST	REST3	28	50
	663	38.18	37.81	36.91	116	4	VEST	REST3	28	50
	664	38.18	37.85	36.88	117	4	VEST	REST3	28	50
	665	38.17	37.83	36.84	117	4	VEST	REST3	28	50
	666	38.18	37.77	36.81	114	4	VEST	REST3	28	50
	667	38.18	37.71	36.78	121	4	VEST	REST3	28	50
	668	38.19	37.75	36.74	116	4	VEST	REST3	28	50
	669	38.18	37.74	36.71	115	4	VEST	REST3	28	50
	670	38.16	37.65	36.70	114	4	VEST	REST3	28	50
	671	38.16	37.68	36.67	115	4	VEST	REST3	28	50
	672	38.16	37.67	36.65	111	4	VEST	REST3	28	50
	673	38.15	37.64	36.63	109	4	VEST	REST3	28	50
	674	38.15	37.61	36.57	109	4	VEST	REST3	28	50
	675	38.14	37.58	36.56	107	4	VEST	REST3	28	50
	676	38.14	37.59	36.55	106	4	VEST	REST3	28	50
	677	38.14	37.60	36.50	107	4	VEST	REST3	28	50
	678	38.13	37.59	36.48	107	4	VEST	REST3	28	50
	679	38.13	37.57	36.45	105	4	VEST	REST3	28	50
	680	38.13	37.56	36.42	103	4	VEST	REST3	28	50
	681	38.12	37.52	36.39	108	4	VEST	REST3	28	50
	682	38.11	37.55	36.36	107	4	VEST	REST3	28	50
	683	38.11	37.58	36.31	111	4	VEST	REST3	28	50
	684	38.11	37.43	36.27	109	4	VEST	REST3	28	50
	685	38.10	37.35	36.28	102	4	VEST	REST3	28	50
	686	38.09	37.41	36.28	100	4	VEST	REST3	28	50
	687	38.08	37.45	36.28	102	4	VEST	REST3	28	50
	688	38.08	37.50	36.26	104	4	VEST	REST3	28	50
	689	38.09	37.48	36.27	103	4	VEST	REST3	28	50
	690	38.09	37.34	36.24	109	4	VEST	REST3	28	50
115	691	38.08	37.34	36.21	104	4	VEST	REST3	28	50
	692	38.08	37.39	36.22	106	4	VEST	REST3	28	50

min	number	Tre	Tes	Tsk-head	HR	ID	condition	period	Ta	RH
	693	38.09	37.37	36.21	105	4	VEST	REST3	28	50
	694	38.08	37.28	36.20	106	4	VEST	REST3	28	50
	695	38.07	37.24	36.19	108	4	VEST	REST3	28	50
	696	38.06	37.30	36.18	111	4	VEST	REST3	28	50
	697	38.06	37.34	36.14	107	4	VEST	REST3	28	50
	698	38.06	37.31	36.08	109	4	VEST	REST3	28	50
	699	38.05	37.25	36.07	105	4	VEST	REST3	28	50
	700	38.05	37.28	36.00	102	4	VEST	REST3	28	50
	701	38.05	37.32	35.92	103	4	VEST	REST3	28	50
	702	38.04	37.29	35.89	97	4	VEST	REST3	28	50
	703	38.04	37.27	35.89	106	4	VEST	REST3	28	50
	704	38.03	37.25	35.91	97	4	VEST	REST3	28	50
	705	38.03	37.23	35.89	101	4	VEST	REST3	28	50
	706	38.02	37.28	35.86	101	4	VEST	REST3	28	50
	707	38.03	37.30	35.83	106	4	VEST	REST3	28	50
	708	38.03	37.37	35.82	99	4	VEST	REST3	28	50
	709	38.03	37.38	35.84	100	4	VEST	REST3	28	50
0	1	37.38	37.08	35.53	103	5	VEST	REST1	28	50
	2	37.38	37.10	35.53	102	5	VEST	REST1	28	50
	3	37.37	37.10	35.53	102	5	VEST	REST1	28	50
	4	37.37	37.13	35.51	103	5	VEST	REST1	28	50
	5	37.37	37.13	35.50	105	5	VEST	REST1	28	50
	6	37.37	37.11	35.53	107	5	VEST	REST1	28	50
	7	37.38	37.11	35.54	105	5	VEST	REST1	28	50
	8	37.38	37.07	35.53	105	5	VEST	REST1	28	50
	9	37.37	37.07	35.51	97	5	VEST	REST1	28	50
	10	37.37	37.12	35.51	102	5	VEST	REST1	28	50
	11	37.37	37.13	35.53	100	5	VEST	REST1	28	50
	12	37.36	37.12	35.52	101	5	VEST	REST1	28	50
	13	37.36	37.11	35.50	99	5	VEST	REST1	28	50
	14	37.36	37.12	35.50	101	5	VEST	REST1	28	50
	15	37.35	37.13	35.52	101	5	VEST	REST1	28	50
	16	37.35	37.13	35.53	103	5	VEST	REST1	28	50
	17	37.36	37.13	35.54	102	5	VEST	REST1	28	50
	18	37.36	37.13	35.54	103	5	VEST	REST1	28	50
	19	37.35	37.11	35.55	98	5	VEST	REST1	28	50
	20	37.35	37.11	35.56	100	5	VEST	REST1	28	50
	21	37.34	37.13	35.56	98	5	VEST	REST1	28	50
	22	37.35	37.11	35.53	99	5	VEST	REST1	28	50
	23	37.34	37.10	35.48	99	5	VEST	REST1	28	50
	24	37.34	37.10	35.46	99	5	VEST	REST1	28	50
	25	37.34	37.11	35.45	105	5	VEST	REST1	28	50
	26	37.34	37.11	35.49	103	5	VEST	REST1	28	50
	27	37.34	37.10	35.55	104	5	VEST	REST1	28	50
	28	37.34	37.09	35.60	105	5	VEST	REST1	28	50
	29	37.33	37.07	35.63	102	5	VEST	REST1	28	50

min	number	Tre	Tes	Tsk-head	HR	ID	condition	period	Ta	RH
	30	37.33	37.06	35.66	103	5	VEST	REST1	28	50
5	31	37.32	37.05	35.64	102	5	VEST	REST1	28	50
	32	37.32	37.06	35.61	108	5	VEST	REST1	28	50
	33	37.32	37.06	35.64	114	5	VEST	REST1	28	50
	34	37.32	37.10	35.64	104	5	VEST	REST1	28	50
	35	37.32	37.10	35.64	105	5	VEST	REST1	28	50
	36	37.32	37.07	35.67	107	5	VEST	REST1	28	50
	37	37.32	37.08	35.66	102	5	VEST	REST1	28	50
	38	37.31	37.07	35.60	99	5	VEST	REST1	28	50
	39	37.31	37.07	35.57	101	5	VEST	REST1	28	50
	40	37.32	37.07	35.58	104	5	VEST	REST1	28	50
	41	37.32	37.07	35.58	100	5	VEST	REST1	28	50
	42	37.31	37.10	35.56	100	5	VEST	REST1	28	50
	43	37.32	37.12	35.53	104	5	VEST	REST1	28	50
	44	37.32	37.11	35.52	104	5	VEST	REST1	28	50
	45	37.32	37.10	35.52	107	5	VEST	REST1	28	50
	46	37.33	37.09	35.46	115	5	VEST	REST1	28	50
	47	37.34	37.11	35.47	114	5	VEST	REST1	28	50
	48	37.34	37.12	35.49	106	5	VEST	REST1	28	50
	49	37.33	37.10	35.47	104	5	VEST	REST1	28	50
	50	37.33	37.11	35.48	105	5	VEST	REST1	28	50
	51	37.34	37.11	35.49	104	5	VEST	REST1	28	50
	52	37.34	37.10	35.50	105	5	VEST	REST1	28	50
	53	37.33	37.10	35.51	106	5	VEST	REST1	28	50
	54	37.33	37.10	35.52	106	5	VEST	REST1	28	50
	55	37.33	37.10	35.52	100	5	VEST	REST1	28	50
	56	37.33	37.11	35.53	101	5	VEST	REST1	28	50
	57	37.32	37.13	35.55	102	5	VEST	REST1	28	50
	58	37.32	37.13	35.54	103	5	VEST	REST1	28	50
	59	37.31	37.12	35.52	107	5	VEST	REST1	28	50
	60	37.30	37.11	35.51	100	5	VEST	REST1	28	50
10	61	37.30	37.07	35.53	108	5	VEST	REST1	28	50
	62	37.30	37.06	35.56	99	5	VEST	REST1	28	50
	63	37.31	37.09	35.56	102	5	VEST	REST1	28	50
	64	37.31	37.09	35.57	92	5	VEST	REST1	28	50
	65	37.31	37.12	35.59	94	5	VEST	REST1	28	50
	66	37.31	37.14	35.57	101	5	VEST	REST1	28	50
	67	37.30	37.11	35.54	102	5	VEST	REST1	28	50
	68	37.30	37.11	35.50	103	5	VEST	REST1	28	50
	69	37.30	37.13	35.45	102	5	VEST	REST1	28	50
	70	37.30	37.09	35.44	110	5	VEST	REST1	28	50
	71	37.29	37.05	35.43	105	5	VEST	REST1	28	50
	72	37.29	37.08	35.43	106	5	VEST	REST1	28	50
	73	37.30	37.09	35.43	103	5	VEST	REST1	28	50
	74	37.30	37.09	35.40	101	5	VEST	REST1	28	50
	75	37.30	37.08	35.41	101	5	VEST	REST1	28	50

min	number	Tre	Tes	Tsk-head	HR	ID	condition	period	Ta	RH
	76	37.29	37.07	35.41	101	5	VEST	REST1	28	50
	77	37.29	37.09	35.38	98	5	VEST	REST1	28	50
	78	37.29	37.08	35.36	99	5	VEST	REST1	28	50
	79	37.29	37.09	35.38	101	5	VEST	REST1	28	50
	80	37.29	37.10	35.38	101	5	VEST	REST1	28	50
	81	37.29	37.11	35.41	103	5	VEST	REST1	28	50
	82	37.29	37.09	35.44	98	5	VEST	REST1	28	50
	83	37.29	37.07	35.42	101	5	VEST	REST1	28	50
	84	37.29	37.07	35.36	95	5	VEST	REST1	28	50
	85	37.29	37.08	35.33	100	5	VEST	REST1	28	50
	86	37.29	37.09	35.35	95	5	VEST	REST1	28	50
	87	37.29	37.11	35.36	98	5	VEST	REST1	28	50
	88	37.29	37.10	35.39	98	5	VEST	REST1	28	50
	89	37.29	37.10	35.44	101	5	VEST	REST1	28	50
	90	37.28	37.10	35.45	98	5	VEST	REST1	28	50
15	91	37.28	37.10	35.44	88	5	VEST	REST1	28	50
	92	37.28	37.10	35.46	93	5	VEST	REST1	28	50
	93	37.28	37.09	35.47	94	5	VEST	REST1	28	50
	94	37.29	37.10	35.46	102	5	VEST	REST1	28	50
	95	37.29	37.10	35.46	101	5	VEST	REST1	28	50
	96	37.27	37.08	35.41	97	5	VEST	REST1	28	50
	97	37.27	37.09	35.41	101	5	VEST	REST1	28	50
	98	37.27	37.10	35.46	106	5	VEST	REST1	28	50
	99	37.28	37.10	35.49	94	5	VEST	REST1	28	50
	100	37.28	37.08	35.50	89	5	VEST	REST1	28	50
	101	37.28	37.08	35.50	94	5	VEST	REST1	28	50
	102	37.28	37.09	35.50	105	5	VEST	REST1	28	50
	103	37.28	37.09	35.53	107	5	VEST	REST1	40	50
	104	37.29	37.08	35.75	117	5	VEST	REST1	40	50
	105	37.29	37.06	36.10	119	5	VEST	REST1	40	50
	106	37.30	37.06	36.32	118	5	VEST	REST1	40	50
	107	37.30	37.07	36.41	108	5	VEST	REST1	40	50
	108	37.30	37.07	36.47	112	5	VEST	REST1	40	50
	109	37.30	37.07	36.53	107	5	VEST	REST1	40	50
	110	37.30	37.07	36.58	116	5	VEST	REST1	40	50
	111	37.31	37.07	36.60	108	5	VEST	REST1	40	50
	112	37.32	37.04	36.62	118	5	VEST	REST1	40	50
	113	37.31	37.00	36.66	119	5	VEST	REST1	40	50
	114	37.31	36.99	36.68	106	5	VEST	REST1	40	50
	115	37.32	37.02	36.71	111	5	VEST	REST1	40	50
	116	37.33	37.04	36.73	107	5	VEST	REST1	40	50
	117	37.33	36.99	36.76	103	5	VEST	REST1	40	50
	118	37.32	36.97	36.77	103	5	VEST	REST1	40	50
	119	37.32	37.02	36.78	106	5	VEST	REST1	40	50
	120	37.32	37.05	36.82	106	5	VEST	REST1	40	50
20	121	37.31	37.05	36.86	108	5	VEST	REST1	40	50

min	number	Tre	Tes	Tsk-head	HR	ID	condition	period	Ta	RH
	122	37.31	37.07	36.87	111	5	VEST	REST1	40	50
	123	37.31	37.05	36.88	107	5	VEST	REST1	40	50
	124	37.32	37.04	36.90	111	5	VEST	REST1	40	50
	125	37.33	37.08	36.91	109	5	VEST	REST1	40	50
	126	37.33	37.11	36.94	112	5	VEST	REST1	40	50
	127	37.33	37.11	36.96	110	5	VEST	REST1	40	50
	128	37.32	37.11	36.96	124	5	VEST	REST1	40	50
	129	37.31	37.08	36.99	115	5	VEST	REST1	40	50
	130	37.32	37.05	37.02	119	5	VEST	REST1	40	50
	131	37.32	37.06	37.02	112	5	VEST	REST1	40	50
	132	37.32	37.10	37.02	112	5	VEST	REST1	40	50
	133	37.33	37.14	37.04	118	5	VEST	REST1	40	50
	134	37.33	37.16	37.07	123	5	VEST	REST1	40	50
	135	37.32	37.15	37.10	128	5	VEST	REST1	40	50
	136	37.32	37.15	37.12	124	5	VEST	REST1	40	50
	137	37.32	37.15	37.12	114	5	VEST	REST1	40	50
	138	37.32	37.16	37.14	118	5	VEST	REST1	40	50
	139	37.33	37.18	37.16	118	5	VEST	EXERCISE1	40	50
	140	37.33	37.16	37.17	118	5	VEST	EXERCISE1	40	50
	141	37.33	37.14	37.17	122	5	VEST	EXERCISE1	40	50
	142	37.33	37.14	37.19	127	5	VEST	EXERCISE1	40	50
	143	37.33	37.16	37.21	128	5	VEST	EXERCISE1	40	50
	144	37.34	37.17	37.22	126	5	VEST	EXERCISE1	40	50
	145	37.34	37.17	37.23	127	5	VEST	EXERCISE1	40	50
	146	37.34	37.16	37.25	125	5	VEST	EXERCISE1	40	50
	147	37.35	37.15	37.26	130	5	VEST	EXERCISE1	40	50
	148	37.35	37.15	37.27	133	5	VEST	EXERCISE1	40	50
	149	37.35	37.16	37.28	132	5	VEST	EXERCISE1	40	50
	150	37.36	37.16	37.29	130	5	VEST	EXERCISE1	40	50
25	151	37.37	37.16	37.29	131	5	VEST	EXERCISE1	40	50
	152	37.37	37.16	37.30	132	5	VEST	EXERCISE1	40	50
	153	37.37	37.18	37.30	133	5	VEST	EXERCISE1	40	50
	154	37.37	37.17	37.30	134	5	VEST	EXERCISE1	40	50
	155	37.37	37.18	37.29	133	5	VEST	EXERCISE1	40	50
	156	37.37	37.21	37.29	131	5	VEST	EXERCISE1	40	50
	157	37.38	37.19	37.30	131	5	VEST	EXERCISE1	40	50
	158	37.39	37.18	37.31	134	5	VEST	EXERCISE1	40	50
	159	37.39	37.20	37.32	136	5	VEST	EXERCISE1	40	50
	160	37.39	37.21	37.33	136	5	VEST	EXERCISE1	40	50
	161	37.39	37.24	37.32	134	5	VEST	EXERCISE1	40	50
	162	37.38	37.26	37.31	134	5	VEST	EXERCISE1	40	50
	163	37.38	37.28	37.32	134	5	VEST	EXERCISE1	40	50
	164	37.39	37.29	37.32	133	5	VEST	EXERCISE1	40	50
	165	37.39	37.31	37.31	138	5	VEST	EXERCISE1	40	50
	166	37.40	37.34	37.32	137	5	VEST	EXERCISE1	40	50
	167	37.40	37.36	37.33	138	5	VEST	EXERCISE1	40	50

min	number	Tre	Tes	Tsk-head	HR	ID	condition	period	Ta	RH
	168	37.40	37.38	37.34	137	5	VEST	EXERCISE1	40	50
	169	37.41	37.39	37.34	135	5	VEST	EXERCISE1	40	50
	170	37.41	37.40	37.33	132	5	VEST	EXERCISE1	40	50
	171	37.42	37.42	37.34	137	5	VEST	EXERCISE1	40	50
	172	37.43	37.43	37.35	136	5	VEST	EXERCISE1	40	50
	173	37.43	37.29	37.36	137	5	VEST	EXERCISE1	40	50
	174	37.44	37.29	37.38	137	5	VEST	EXERCISE1	40	50
	175	37.45	37.43	37.39	139	5	VEST	EXERCISE1	40	50
	176	37.45	37.45	37.40	133	5	VEST	EXERCISE1	40	50
	177	37.45	37.48	37.41	137	5	VEST	EXERCISE1	40	50
	178	37.45	37.51	37.42	135	5	VEST	EXERCISE1	40	50
	179	37.45	37.52	37.43	134	5	VEST	EXERCISE1	40	50
	180	37.45	37.52	37.43	135	5	VEST	EXERCISE1	40	50
30	181	37.46	37.53	37.45	136	5	VEST	EXERCISE1	40	50
	182	37.47	37.54	37.46	138	5	VEST	EXERCISE1	40	50
	183	37.47	37.55	37.47	138	5	VEST	EXERCISE1	40	50
	184	37.48	37.56	37.49	143	5	VEST	EXERCISE1	40	50
	185	37.49	37.56	37.51	143	5	VEST	EXERCISE1	40	50
	186	37.49	37.57	37.52	145	5	VEST	EXERCISE1	40	50
	187	37.49	37.59	37.52	145	5	VEST	EXERCISE1	40	50
	188	37.49	37.60	37.53	145	5	VEST	EXERCISE1	40	50
	189	37.49	37.60	37.53	143	5	VEST	EXERCISE1	40	50
	190	37.50	37.59	37.55	143	5	VEST	EXERCISE1	40	50
	191	37.51	37.60	37.55	143	5	VEST	EXERCISE1	40	50
	192	37.52	37.62	37.55	145	5	VEST	EXERCISE1	40	50
	193	37.52	37.63	37.57	148	5	VEST	EXERCISE1	40	50
	194	37.52	37.65	37.59	146	5	VEST	EXERCISE1	40	50
	195	37.53	37.66	37.59	149	5	VEST	EXERCISE1	40	50
	196	37.53	37.66	37.59	150	5	VEST	EXERCISE1	40	50
	197	37.53	37.66	37.61	150	5	VEST	EXERCISE1	40	50
	198	37.53	37.67	37.62	148	5	VEST	EXERCISE1	40	50
	199	37.54	37.69	37.63	146	5	VEST	EXERCISE1	40	50
	200	37.54	37.53	37.64	150	5	VEST	EXERCISE1	40	50
	201	37.54	37.49	37.65	149	5	VEST	EXERCISE1	40	50
	202	37.55	37.64	37.67	143	5	VEST	EXERCISE1	40	50
	203	37.55	37.67	37.69	141	5	VEST	EXERCISE1	40	50
	204	37.55	37.70	37.69	140	5	VEST	EXERCISE1	40	50
	205	37.57	37.69	37.70	143	5	VEST	EXERCISE1	40	50
	206	37.58	37.68	37.71	144	5	VEST	EXERCISE1	40	50
	207	37.58	37.70	37.72	144	5	VEST	EXERCISE1	40	50
	208	37.58	37.72	37.73	142	5	VEST	EXERCISE1	40	50
	209	37.58	37.73	37.72	144	5	VEST	EXERCISE1	40	50
	210	37.59	37.74	37.73	143	5	VEST	EXERCISE1	40	50
35	211	37.59	37.73	37.73	145	5	VEST	EXERCISE1	40	50
	212	37.59	37.73	37.74	145	5	VEST	EXERCISE1	40	50
	213	37.60	37.73	37.75	146	5	VEST	EXERCISE1	40	50

min	number	Tre	Tes	Tsk-head	HR	ID	condition	period	Ta	RH
	214	37.61	37.71	37.75	146	5	VEST	EXERCISE1	40	50
	215	37.61	37.71	37.74	145	5	VEST	EXERCISE1	40	50
	216	37.62	37.72	37.74	143	5	VEST	EXERCISE1	40	50
	217	37.63	37.73	37.76	143	5	VEST	EXERCISE1	40	50
	218	37.64	37.69	37.76	146	5	VEST	EXERCISE1	40	50
	219	37.64	37.68	37.76	144	5	VEST	EXERCISE1	40	50
	220	37.64	37.70	37.77	146	5	VEST	EXERCISE1	40	50
	221	37.63	37.71	37.77	145	5	VEST	EXERCISE1	40	50
	222	37.63	37.70	37.77	146	5	VEST	EXERCISE1	40	50
	223	37.64	37.71	37.78	149	5	VEST	EXERCISE1	40	50
	224	37.64	37.72	37.78	150	5	VEST	EXERCISE1	40	50
	225	37.64	37.72	37.78	149	5	VEST	EXERCISE1	40	50
	226	37.65	37.72	37.79	150	5	VEST	EXERCISE1	40	50
	227	37.65	37.73	37.80	150	5	VEST	EXERCISE1	40	50
	228	37.66	37.73	37.80	153	5	VEST	EXERCISE1	40	50
	229	37.67	37.74	37.80	153	5	VEST	EXERCISE1	40	50
	230	37.67	37.75	37.81	153	5	VEST	EXERCISE1	40	50
	231	37.67	37.76	37.81	153	5	VEST	EXERCISE1	40	50
	232	37.67	37.73	37.82	152	5	VEST	EXERCISE1	40	50
	233	37.67	37.70	37.83	150	5	VEST	EXERCISE1	40	50
	234	37.69	37.71	37.83	150	5	VEST	EXERCISE1	40	50
	235	37.69	37.72	37.83	150	5	VEST	EXERCISE1	40	50
	236	37.69	37.72	37.82	150	5	VEST	EXERCISE1	40	50
	237	37.70	37.73	37.83	150	5	VEST	EXERCISE1	40	50
	238	37.71	37.74	37.83	150	5	VEST	EXERCISE1	40	50
	239	37.72	37.74	37.79	153	5	VEST	EXERCISE1	40	50
	240	37.71	37.75	37.78	153	5	VEST	EXERCISE1	40	50
40	241	37.71	37.75	37.81	153	5	VEST	EXERCISE1	40	50
	242	37.71	37.76	37.80	153	5	VEST	EXERCISE1	40	50
	243	37.73	37.77	37.82	152	5	VEST	EXERCISE1	40	50
	244	37.73	37.77	37.84	148	5	VEST	EXERCISE1	40	50
	245	37.73	37.77	37.83	147	5	VEST	EXERCISE1	40	50
	246	37.73	37.78	37.83	149	5	VEST	EXERCISE1	40	50
	247	37.73	37.79	37.83	147	5	VEST	EXERCISE1	40	50
	248	37.74	37.79	37.83	150	5	VEST	EXERCISE1	40	50
	249	37.74	37.80	37.83	153	5	VEST	EXERCISE1	40	50
	250	37.74	37.81	37.83	153	5	VEST	EXERCISE1	40	50
	251	37.75	37.81	37.83	153	5	VEST	EXERCISE1	40	50
	252	37.76	37.81	37.84	153	5	VEST	EXERCISE1	40	50
	253	37.77	37.81	37.86	153	5	VEST	EXERCISE1	40	50
	254	37.78	37.82	37.88	154	5	VEST	EXERCISE1	40	50
	255	37.78	37.82	37.89	157	5	VEST	EXERCISE1	40	50
	256	37.78	37.81	37.89	157	5	VEST	EXERCISE1	40	50
	257	37.78	37.82	37.87	158	5	VEST	EXERCISE1	40	50
	258	37.78	37.83	37.85	157	5	VEST	EXERCISE1	40	50
	259	37.79	37.83	37.85	157	5	VEST	EXERCISE1	40	50

min	number	Tre	Tes	Tsk-head	HR	ID	condition	period	Ta	RH
	260	37.80	37.81	37.87	158	5	VEST	EXERCISE1	40	50
	261	37.80	37.80	37.88	155	5	VEST	EXERCISE1	40	50
	262	37.80	37.83	37.89	150	5	VEST	EXERCISE1	40	50
	263	37.80	37.83	37.90	149	5	VEST	EXERCISE1	40	50
	264	37.80	37.82	37.92	152	5	VEST	EXERCISE1	40	50
	265	37.81	37.84	37.92	152	5	VEST	EXERCISE1	40	50
	266	37.81	37.86	37.92	153	5	VEST	EXERCISE1	40	50
	267	37.81	37.87	37.92	153	5	VEST	EXERCISE1	40	50
	268	37.82	37.88	37.91	157	5	VEST	EXERCISE1	40	50
	269	37.83	37.89	37.91	157	5	VEST	EXERCISE1	40	50
	270	37.84	37.88	37.91	157	5	VEST	EXERCISE1	40	50
45	271	37.85	37.87	37.91	157	5	VEST	EXERCISE1	40	50
	272	37.84	37.86	37.90	154	5	VEST	EXERCISE1	40	50
	273	37.84	37.86	37.90	153	5	VEST	EXERCISE1	40	50
	274	37.85	37.87	37.90	154	5	VEST	EXERCISE1	40	50
	275	37.85	37.88	37.90	153	5	VEST	EXERCISE1	40	50
	276	37.86	37.88	37.90	155	5	VEST	EXERCISE1	40	50
	277	37.86	37.86	37.89	157	5	VEST	EXERCISE1	40	50
	278	37.86	37.83	37.88	157	5	VEST	EXERCISE1	40	50
	279	37.87	37.84	37.88	158	5	VEST	EXERCISE1	40	50
	280	37.87	37.86	37.88	160	5	VEST	EXERCISE1	40	50
	281	37.87	37.85	37.89	161	5	VEST	EXERCISE1	40	50
	282	37.88	37.84	37.89	157	5	VEST	EXERCISE1	40	50
	283	37.89	37.85	37.89	158	5	VEST	EXERCISE1	40	50
	284	37.90	37.85	37.90	159	5	VEST	EXERCISE1	40	50
	285	37.90	37.84	37.90	159	5	VEST	EXERCISE1	40	50
	286	37.90	37.85	37.91	155	5	VEST	EXERCISE1	40	50
	287	37.91	37.87	37.91	153	5	VEST	EXERCISE1	40	50
	288	37.91	37.86	37.90	154	5	VEST	EXERCISE1	40	50
	289	37.92	37.87	37.90	154	5	VEST	EXERCISE1	40	50
	290	37.93	37.87	37.91	154	5	VEST	EXERCISE1	40	50
	291	37.93	37.87	37.91	157	5	VEST	EXERCISE1	40	50
	292	37.94	37.88	37.92	157	5	VEST	EXERCISE1	40	50
	293	37.94	37.90	37.94	157	5	VEST	EXERCISE1	40	50
	294	37.95	37.90	37.94	157	5	VEST	EXERCISE1	40	50
	295	37.95	37.90	37.94	157	5	VEST	EXERCISE1	40	50
	296	37.95	37.91	37.95	157	5	VEST	EXERCISE1	40	50
	297	37.95	37.92	37.96	153	5	VEST	EXERCISE1	40	50
	298	37.96	37.93	37.95	156	5	VEST	EXERCISE1	40	50
	299	37.96	37.93	37.95	159	5	VEST	EXERCISE1	40	50
	300	37.96	37.93	37.90	158	5	VEST	EXERCISE1	40	50
50	301	37.97	37.94	37.87	157	5	VEST	EXERCISE1	40	50
	302	37.97	37.93	37.86	158	5	VEST	EXERCISE1	40	50
	303	37.97	37.92	37.85	160	5	VEST	EXERCISE1	40	50
	304	37.97	37.91	37.90	158	5	VEST	EXERCISE1	40	50
	305	37.97	37.92	37.92	158	5	VEST	EXERCISE1	40	50

min	number	Tre	Tes	Tsk-head	HR	ID	condition	period	Ta	RH
	306	37.98	37.93	37.94	157	5	VEST	EXERCISE1	40	50
	307	37.99	37.93	37.95	157	5	VEST	EXERCISE1	40	50
	308	37.99	37.83	37.93	162	5	VEST	EXERCISE1	40	50
	309	38.00	37.81	37.95	159	5	VEST	EXERCISE1	40	50
	310	38.01	37.87	37.95	159	5	VEST	EXERCISE1	40	50
	311	38.00	37.89	37.95	157	5	VEST	EXERCISE1	40	50
	312	38.00	37.90	37.95	157	5	VEST	EXERCISE1	40	50
	313	38.00	37.90	37.95	157	5	VEST	EXERCISE1	40	50
	314	38.00	37.92	37.95	157	5	VEST	EXERCISE1	40	50
	315	38.01	37.93	37.96	155	5	VEST	EXERCISE1	40	50
	316	38.02	37.93	37.95	153	5	VEST	EXERCISE1	40	50
	317	38.03	37.94	37.94	156	5	VEST	EXERCISE1	40	50
	318	38.03	37.95	37.95	157	5	VEST	EXERCISE1	40	50
	319	38.04	37.95	37.95	159	5	VEST	EXERCISE1	40	50
	320	38.05	37.95	37.94	155	5	VEST	REST2	28	50
	321	38.05	37.91	37.93	157	5	VEST	REST2	28	50
	322	38.05	37.89	37.74	155	5	VEST	REST2	28	50
	323	38.05	37.85	37.51	154	5	VEST	REST2	28	50
	324	38.06	37.86	37.42	150	5	VEST	REST2	28	50
	325	38.07	37.95	37.35	153	5	VEST	REST2	28	50
	326	38.07	37.98	37.29	153	5	VEST	REST2	28	50
	327	38.07	37.97	37.25	146	5	VEST	REST2	28	50
	328	38.07	37.95	37.24	144	5	VEST	REST2	28	50
	329	38.07	37.94	37.21	147	5	VEST	REST2	28	50
	330	38.08	37.91	37.19	140	5	VEST	REST2	28	50
55	331	38.08	37.86	37.16	140	5	VEST	REST2	28	50
	332	38.08	37.84	37.13	139	5	VEST	REST2	28	50
	333	38.08	37.83	37.12	138	5	VEST	REST2	28	50
	334	38.08	37.78	37.11	134	5	VEST	REST2	28	50
	335	38.08	37.76	37.10	131	5	VEST	REST2	28	50
	336	38.09	37.73	37.04	132	5	VEST	REST2	28	50
	337	38.09	37.65	36.98	130	5	VEST	REST2	28	50
	338	38.09	37.61	36.97	131	5	VEST	REST2	28	50
	339	38.09	34.24	36.96	142	5	VEST	REST2	28	50
	340	38.10	29.98	36.92	140	5	VEST	REST2	28	50
	341	38.10	28.98	36.87	141	5	VEST	REST2	28	50
	342	38.10	30.04	36.84	135	5	VEST	REST2	28	50
	343	38.11	32.26	36.79	137	5	VEST	REST2	28	50
	344	38.10	33.73	36.75	134	5	VEST	REST2	28	50
	345	38.10	30.39	36.76	135	5	VEST	REST2	28	50
	346	38.10	26.40	36.72	140	5	VEST	REST2	28	50
	347	38.10	27.81	36.69	139	5	VEST	REST2	28	50
	348	38.10	30.17	36.68	131	5	VEST	REST2	28	50
	349	38.10	28.50	36.65	129	5	VEST	REST2	28	50
	350	38.10	26.58	36.63	140	5	VEST	REST2	28	50
	351	38.11	28.20	36.64	122	5	VEST	REST2	28	50

min	number	Tre	Tes	Tsk-head	HR	ID	condition	period	Ta	RH
	352	38.12	28.64	36.57	135	5	VEST	REST2	28	50
	353	38.12	28.91	36.52	127	5	VEST	REST2	28	50
	354	38.11	28.72	36.54	128	5	VEST	REST2	28	50
	355	38.10	27.91	36.51	137	5	VEST	REST2	28	50
	356	38.09	29.55	36.46	133	5	VEST	REST2	28	50
	357	38.09	31.48	36.42	131	5	VEST	REST2	28	50
	358	38.09	32.51	36.39	126	5	VEST	REST2	28	50
	359	38.09	33.45	36.34	127	5	VEST	REST2	28	50
	360	38.09	34.20	36.29	127	5	VEST	REST2	28	50
60	361	38.09	34.68	36.29	125	5	VEST	REST2	28	50
	362	38.09	35.15	36.32	126	5	VEST	REST2	28	50
	363	38.08	35.47	36.32	122	5	VEST	REST2	28	50
	364	38.07	35.74	36.34	122	5	VEST	REST2	28	50
	365	38.07	35.98	36.37	125	5	VEST	REST2	28	50
	366	38.08	35.59	36.31	121	5	VEST	REST2	28	50
	367	38.09	35.29	36.28	125	5	VEST	REST2	28	50
	368	38.08	35.79	36.28	125	5	VEST	REST2	28	50
	369	38.07	36.14	36.23	121	5	VEST	REST2	28	50
	370	38.07	36.37	36.21	119	5	VEST	REST2	28	50
	371	38.07	36.67	36.25	128	5	VEST	REST2	28	50
	372	38.07	36.83	36.25	118	5	VEST	REST2	28	50
	373	38.07	36.84	36.20	116	5	VEST	REST2	28	50
	374	38.07	36.91	36.19	107	5	VEST	REST2	28	50
	375	38.06	37.02	36.17	117	5	VEST	REST2	28	50
	376	38.05	37.03	36.12	112	5	VEST	REST2	28	50
	377	38.04	37.02	36.09	115	5	VEST	REST2	28	50
	378	38.03	37.04	36.06	115	5	VEST	REST2	28	50
	379	38.02	37.08	36.04	124	5	VEST	REST2	28	50
	380	38.01	37.07	36.01	114	5	VEST	REST2	28	50
	381	38.00	37.09	35.95	113	5	VEST	REST2	28	50
	382	37.98	37.10	35.92	109	5	VEST	REST2	28	50
	383	37.98	37.11	35.95	109	5	VEST	REST2	28	50
	384	37.98	37.15	35.97	113	5	VEST	REST2	28	50
	385	37.97	37.14	35.97	116	5	VEST	REST2	28	50
	386	37.96	37.12	35.94	109	5	VEST	REST2	28	50
	387	37.94	37.14	35.90	110	5	VEST	REST2	28	50
	388	37.94	37.17	35.90	108	5	VEST	REST2	28	50
	389	37.93	37.16	35.92	109	5	VEST	REST2	28	50
	390	37.93	37.14	35.89	106	5	VEST	REST2	28	50
65	391	37.92	37.19	35.83	104	5	VEST	REST2	28	50
	392	37.92	37.18	35.85	115	5	VEST	REST2	28	50
	393	37.91	37.18	35.87	111	5	VEST	REST2	28	50
	394	37.91	37.23	35.87	110	5	VEST	REST2	28	50
	395	37.91	37.25	35.88	107	5	VEST	REST2	28	50
	396	37.90	37.25	35.86	107	5	VEST	REST2	28	50
	397	37.89	37.25	35.86	104	5	VEST	REST2	28	50

min	number	Tre	Tes	Tsk-head	HR	ID	condition	period	Ta	RH
	398	37.88	37.26	35.84	106	5	VEST	REST2	28	50
	399	37.87	37.16	35.85	112	5	VEST	REST2	28	50
	400	37.86	37.14	35.89	112	5	VEST	REST2	28	50
	401	37.85	37.24	35.93	100	5	VEST	REST2	28	50
	402	37.84	37.25	35.96	110	5	VEST	REST2	28	50
	403	37.84	37.26	35.98	102	5	VEST	REST2	28	50
	404	37.85	37.27	36.00	102	5	VEST	REST2	28	50
	405	37.84	37.27	35.98	105	5	VEST	REST2	28	50
	406	37.84	37.29	35.97	105	5	VEST	REST2	28	50
	407	37.83	37.32	35.98	104	5	VEST	REST2	28	50
	408	37.83	37.33	35.97	109	5	VEST	REST2	28	50
	409	37.83	37.31	35.95	106	5	VEST	REST2	28	50
	410	37.84	37.32	35.98	101	5	VEST	REST2	28	50
	411	37.83	37.35	36.01	103	5	VEST	REST2	28	50
	412	37.82	37.36	35.98	106	5	VEST	REST2	28	50
	413	37.83	37.37	35.93	107	5	VEST	REST2	28	50
	414	37.83	37.37	35.91	104	5	VEST	REST2	28	50
	415	37.82	37.36	35.87	105	5	VEST	REST2	28	50
	416	37.82	37.39	35.80	108	5	VEST	REST2	28	50
	417	37.81	37.37	35.77	109	5	VEST	REST2	28	50
	418	37.81	37.33	35.78	106	5	VEST	REST2	28	50
	419	37.80	37.33	35.77	106	5	VEST	REST2	28	50
	420	37.79	37.33	35.81	107	5	VEST	REST2	28	50
70	421	37.81	37.30	35.90	102	5	VEST	REST2	28	50
	422	37.82	37.29	35.94	109	5	VEST	REST2	28	50
	423	37.82	37.32	35.93	110	5	VEST	REST2	28	50
	424	37.80	37.31	35.93	110	5	VEST	REST2	28	50
	425	37.80	37.32	35.98	109	5	VEST	REST2	28	50
	426	37.80	37.35	36.03	104	5	VEST	REST2	28	50
	427	37.80	37.37	36.04	107	5	VEST	REST2	28	50
	428	37.80	37.37	36.05	120	5	VEST	REST2	28	50
	429	37.81	37.35	36.11	107	5	VEST	REST2	28	50
	430	37.82	37.35	36.16	109	5	VEST	REST2	28	50
	431	37.82	37.36	36.16	104	5	VEST	REST2	28	50
	432	37.82	37.35	36.16	112	5	VEST	REST2	28	50
	433	37.82	37.34	36.17	109	5	VEST	REST2	28	50
	434	37.81	37.28	36.13	111	5	VEST	REST2	28	50
	435	37.81	37.27	36.09	110	5	VEST	REST2	28	50
	436	37.81	37.32	36.08	111	5	VEST	REST2	28	50
	437	37.81	37.35	36.09	103	5	VEST	REST2	28	50
	438	37.81	37.34	36.13	101	5	VEST	REST2	28	50
	439	37.81	37.35	36.16	103	5	VEST	REST2	40	50
	440	37.81	37.38	36.24	115	5	VEST	REST2	40	50
	441	37.82	37.33	36.50	122	5	VEST	REST2	40	50
	442	37.82	37.30	36.78	121	5	VEST	REST2	40	50
	443	37.81	37.34	36.90	120	5	VEST	REST2	40	50

min	number	Tre	Tes	Tsk-head	HR	ID	condition	period	Ta	RH
	444	37.80	37.35	36.96	121	5	VEST	REST2	40	50
	445	37.79	37.36	37.01	121	5	VEST	REST2	40	50
	446	37.79	37.33	37.07	110	5	VEST	REST2	40	50
	447	37.78	37.25	37.11	105	5	VEST	REST2	40	50
	448	37.78	37.21	37.14	109	5	VEST	REST2	40	50
	449	37.78	37.24	37.18	102	5	VEST	REST2	40	50
	450	37.78	37.07	37.19	126	5	VEST	REST2	40	50
75	451	37.78	36.95	37.20	122	5	VEST	REST2	40	50
	452	37.77	37.11	37.24	118	5	VEST	REST2	40	50
	453	37.77	37.19	37.25	111	5	VEST	REST2	40	50
	454	37.76	37.20	37.27	114	5	VEST	REST2	40	50
	455	37.76	37.21	37.27	110	5	VEST	REST2	40	50
	456	37.77	37.23	37.27	112	5	VEST	REST2	40	50
	457	37.77	37.23	37.27	112	5	VEST	REST2	40	50
	458	37.77	37.18	37.25	121	5	VEST	REST2	40	50
	459	37.78	37.21	37.25	127	5	VEST	REST2	40	50
	460	37.79	37.29	37.27	129	5	VEST	REST2	40	50
	461	37.79	37.29	37.29	117	5	VEST	REST2	40	50
	462	37.79	37.31	37.29	117	5	VEST	REST2	40	50
	463	37.80	37.35	37.29	121	5	VEST	EXERCISE2	40	50
	464	37.80	37.35	37.30	125	5	VEST	EXERCISE2	40	50
	465	37.81	37.35	37.31	126	5	VEST	EXERCISE2	40	50
	466	37.81	37.35	37.32	132	5	VEST	EXERCISE2	40	50
	467	37.81	37.35	37.34	131	5	VEST	EXERCISE2	40	50
	468	37.81	37.39	37.35	127	5	VEST	EXERCISE2	40	50
	469	37.80	37.41	37.35	132	5	VEST	EXERCISE2	40	50
	470	37.80	37.41	37.36	132	5	VEST	EXERCISE2	40	50
	471	37.80	37.43	37.37	134	5	VEST	EXERCISE2	40	50
	472	37.80	37.45	37.36	137	5	VEST	EXERCISE2	40	50
	473	37.80	37.48	37.36	137	5	VEST	EXERCISE2	40	50
	474	37.81	37.50	37.38	137	5	VEST	EXERCISE2	40	50
	475	37.81	37.53	37.39	140	5	VEST	EXERCISE2	40	50
	476	37.80	37.52	37.41	141	5	VEST	EXERCISE2	40	50
	477	37.81	37.45	37.42	140	5	VEST	EXERCISE2	40	50
	478	37.81	37.45	37.42	140	5	VEST	EXERCISE2	40	50
	479	37.82	37.51	37.44	140	5	VEST	EXERCISE2	40	50
	480	37.82	37.54	37.46	138	5	VEST	EXERCISE2	40	50
80	481	37.82	37.58	37.46	140	5	VEST	EXERCISE2	40	50
	482	37.83	37.60	37.48	140	5	VEST	EXERCISE2	40	50
	483	37.83	37.62	37.50	138	5	VEST	EXERCISE2	40	50
	484	37.83	37.63	37.51	141	5	VEST	EXERCISE2	40	50
	485	37.84	37.64	37.52	136	5	VEST	EXERCISE2	40	50
	486	37.84	37.66	37.54	136	5	VEST	EXERCISE2	40	50
	487	37.84	37.67	37.54	137	5	VEST	EXERCISE2	40	50
	488	37.84	37.68	37.56	138	5	VEST	EXERCISE2	40	50
	489	37.84	37.68	37.56	140	5	VEST	EXERCISE2	40	50

min	number	Tre	Tes	Tsk-head	HR	ID	condition	period	Ta	RH
	490	37.85	37.69	37.59	140	5	VEST	EXERCISE2	40	50
	491	37.84	37.70	37.61	140	5	VEST	EXERCISE2	40	50
	492	37.84	37.71	37.61	143	5	VEST	EXERCISE2	40	50
	493	37.85	37.71	37.63	143	5	VEST	EXERCISE2	40	50
	494	37.86	37.72	37.64	144	5	VEST	EXERCISE2	40	50
	495	37.86	37.72	37.65	144	5	VEST	EXERCISE2	40	50
	496	37.86	37.71	37.67	144	5	VEST	EXERCISE2	40	50
	497	37.85	37.71	37.69	138	5	VEST	EXERCISE2	40	50
	498	37.85	37.72	37.69	146	5	VEST	EXERCISE2	40	50
	499	37.86	37.74	37.69	144	5	VEST	EXERCISE2	40	50
	500	37.87	37.74	37.67	143	5	VEST	EXERCISE2	40	50
	501	37.86	37.73	37.65	142	5	VEST	EXERCISE2	40	50
	502	37.86	37.73	37.66	138	5	VEST	EXERCISE2	40	50
	503	37.86	37.76	37.65	140	5	VEST	EXERCISE2	40	50
	504	37.86	37.76	37.66	140	5	VEST	EXERCISE2	40	50
	505	37.87	37.76	37.67	142	5	VEST	EXERCISE2	40	50
	506	37.88	37.75	37.68	143	5	VEST	EXERCISE2	40	50
	507	37.88	37.73	37.70	143	5	VEST	EXERCISE2	40	50
	508	37.88	37.72	37.68	143	5	VEST	EXERCISE2	40	50
	509	37.88	37.72	37.68	144	5	VEST	EXERCISE2	40	50
	510	37.89	37.74	37.70	146	5	VEST	EXERCISE2	40	50
85	511	37.89	37.75	37.70	146	5	VEST	EXERCISE2	40	50
	512	37.89	37.74	37.69	144	5	VEST	EXERCISE2	40	50
	513	37.89	37.72	37.71	145	5	VEST	EXERCISE2	40	50
	514	37.90	37.74	37.73	149	5	VEST	EXERCISE2	40	50
	515	37.90	37.75	37.74	148	5	VEST	EXERCISE2	40	50
	516	37.90	37.75	37.74	146	5	VEST	EXERCISE2	40	50
	517	37.91	37.76	37.75	143	5	VEST	EXERCISE2	40	50
	518	37.92	37.78	37.75	146	5	VEST	EXERCISE2	40	50
	519	37.92	37.77	37.71	146	5	VEST	EXERCISE2	40	50
	520	37.93	37.78	37.66	147	5	VEST	EXERCISE2	40	50
	521	37.94	37.79	37.66	150	5	VEST	EXERCISE2	40	50
	522	37.93	37.78	37.68	150	5	VEST	EXERCISE2	40	50
	523	37.93	37.78	37.69	150	5	VEST	EXERCISE2	40	50
	524	37.93	37.80	37.70	148	5	VEST	EXERCISE2	40	50
	525	37.93	37.82	37.69	145	5	VEST	EXERCISE2	40	50
	526	37.94	37.84	37.68	147	5	VEST	EXERCISE2	40	50
	527	37.95	37.80	37.70	150	5	VEST	EXERCISE2	40	50
	528	37.95	37.77	37.74	150	5	VEST	EXERCISE2	40	50
	529	37.95	37.82	37.75	150	5	VEST	EXERCISE2	40	50
	530	37.96	37.86	37.75	147	5	VEST	EXERCISE2	40	50
	531	37.95	37.86	37.75	146	5	VEST	EXERCISE2	40	50
	532	37.95	37.85	37.76	146	5	VEST	EXERCISE2	40	50
	533	37.96	37.84	37.78	145	5	VEST	EXERCISE2	40	50
	534	37.96	37.84	37.80	146	5	VEST	EXERCISE2	40	50
	535	37.97	37.84	37.80	150	5	VEST	EXERCISE2	40	50

min	number	Tre	Tes	Tsk-head	HR	ID	condition	period	Ta	RH
	536	37.97	37.84	37.79	147	5	VEST	EXERCISE2	40	50
	537	37.98	37.84	37.79	149	5	VEST	EXERCISE2	40	50
	538	37.98	37.82	37.79	146	5	VEST	EXERCISE2	40	50
	539	37.97	37.80	37.77	149	5	VEST	EXERCISE2	40	50
	540	37.98	37.79	37.75	150	5	VEST	EXERCISE2	40	50
90	541	37.99	37.80	37.71	151	5	VEST	EXERCISE2	40	50
	542	37.99	37.80	37.68	154	5	VEST	EXERCISE2	40	50
	543	37.99	37.81	37.66	149	5	VEST	EXERCISE2	40	50
	544	38.00	37.82	37.64	149	5	VEST	EXERCISE2	40	50
	545	38.00	37.82	37.63	150	5	VEST	EXERCISE2	40	50
	546	38.00	37.81	37.62	150	5	VEST	EXERCISE2	40	50
	547	38.01	37.82	37.65	143	5	VEST	EXERCISE2	40	50
	548	38.02	37.85	37.65	143	5	VEST	EXERCISE2	40	50
	549	38.01	37.84	37.62	143	5	VEST	EXERCISE2	40	50
	550	38.01	37.84	37.62	147	5	VEST	EXERCISE2	40	50
	551	38.02	37.85	37.63	151	5	VEST	EXERCISE2	40	50
	552	38.03	37.85	37.65	151	5	VEST	EXERCISE2	40	50
	553	38.03	37.85	37.68	152	5	VEST	EXERCISE2	40	50
	554	38.04	37.85	37.69	150	5	VEST	EXERCISE2	40	50
	555	38.04	37.83	37.68	150	5	VEST	EXERCISE2	40	50
	556	38.04	37.82	37.67	150	5	VEST	EXERCISE2	40	50
	557	38.05	37.83	37.66	150	5	VEST	EXERCISE2	40	50
	558	38.05	37.84	37.67	150	5	VEST	EXERCISE2	40	50
	559	38.05	37.85	37.67	148	5	VEST	EXERCISE2	40	50
	560	38.06	37.85	37.67	147	5	VEST	EXERCISE2	40	50
	561	38.07	37.85	37.68	144	5	VEST	EXERCISE2	40	50
	562	38.07	37.85	37.69	146	5	VEST	EXERCISE2	40	50
	563	38.08	37.84	37.68	146	5	VEST	EXERCISE2	40	50
	564	38.08	37.84	37.65	150	5	VEST	EXERCISE2	40	50
	565	38.09	37.86	37.64	154	5	VEST	EXERCISE2	40	50
	566	38.10	37.86	37.64	154	5	VEST	EXERCISE2	40	50
	567	38.10	37.85	37.64	152	5	VEST	EXERCISE2	40	50
	568	38.10	37.79	37.63	153	5	VEST	EXERCISE2	40	50
	569	38.10	37.71	37.61	153	5	VEST	EXERCISE2	40	50
	570	38.11	37.74	37.59	151	5	VEST	EXERCISE2	40	50
95	571	38.12	37.80	37.59	151	5	VEST	EXERCISE2	40	50
	572	38.12	37.82	37.60	150	5	VEST	EXERCISE2	40	50
	573	38.12	37.83	37.61	150	5	VEST	EXERCISE2	40	50
	574	38.13	37.85	37.62	150	5	VEST	EXERCISE2	40	50
	575	38.14	37.85	37.63	150	5	VEST	EXERCISE2	40	50
	576	38.14	37.85	37.64	154	5	VEST	EXERCISE2	40	50
	577	38.14	37.87	37.67	150	5	VEST	EXERCISE2	40	50
	578	38.15	37.89	37.70	150	5	VEST	EXERCISE2	40	50
	579	38.15	37.91	37.71	150	5	VEST	EXERCISE2	40	50
	580	38.15	37.90	37.70	150	5	VEST	EXERCISE2	40	50
	581	38.16	37.90	37.70	149	5	VEST	EXERCISE2	40	50

min	number	Tre	Tes	Tsk-head	HR	ID	condition	period	Ta	RH
	582	38.16	37.91	37.70	150	5	VEST	EXERCISE2	40	50
	583	38.16	37.92	37.70	152	5	VEST	EXERCISE2	40	50
	584	38.17	37.93	37.71	154	5	VEST	EXERCISE2	40	50
	585	38.18	37.89	37.71	154	5	VEST	EXERCISE2	40	50
	586	38.18	37.87	37.72	153	5	VEST	EXERCISE2	40	50
	587	38.18	37.89	37.71	154	5	VEST	EXERCISE2	40	50
	588	38.19	37.92	37.71	154	5	VEST	EXERCISE2	40	50
	589	38.20	37.93	37.72	154	5	VEST	EXERCISE2	40	50
	590	38.20	37.93	37.71	154	5	VEST	EXERCISE2	40	50
	591	38.20	37.93	37.71	154	5	VEST	EXERCISE2	40	50
	592	38.20	37.94	37.73	151	5	VEST	EXERCISE2	40	50
	593	38.21	37.94	37.73	154	5	VEST	EXERCISE2	40	50
	594	38.22	37.93	37.74	151	5	VEST	EXERCISE2	40	50
	595	38.23	37.94	37.76	154	5	VEST	EXERCISE2	40	50
	596	38.23	37.94	37.76	155	5	VEST	EXERCISE2	40	50
	597	38.22	37.94	37.75	154	5	VEST	EXERCISE2	40	50
	598	38.23	37.91	37.72	154	5	VEST	EXERCISE2	40	50
	599	38.23	37.89	37.71	150	5	VEST	EXERCISE2	40	50
	600	38.23	37.90	37.72	154	5	VEST	EXERCISE2	40	50
100	601	38.22	37.90	37.73	154	5	VEST	EXERCISE2	40	50
	602	38.22	37.91	37.74	154	5	VEST	EXERCISE2	40	50
	603	38.23	37.93	37.74	150	5	VEST	EXERCISE2	40	50
	604	38.24	37.93	37.73	150	5	VEST	EXERCISE2	40	50
	605	38.24	37.93	37.73	151	5	VEST	EXERCISE2	40	50
	606	38.24	37.94	37.73	151	5	VEST	EXERCISE2	40	50
	607	38.25	37.95	37.72	151	5	VEST	EXERCISE2	40	50
	608	38.25	37.94	37.71	153	5	VEST	EXERCISE2	40	50
	609	38.25	37.93	37.70	154	5	VEST	EXERCISE2	40	50
	610	38.26	37.94	37.72	154	5	VEST	EXERCISE2	40	50
	611	38.26	37.95	37.74	154	5	VEST	EXERCISE2	40	50
	612	38.26	37.95	37.75	154	5	VEST	EXERCISE2	40	50
	613	38.27	37.95	37.76	153	5	VEST	EXERCISE2	40	50
	614	38.27	37.95	37.76	154	5	VEST	EXERCISE2	40	50
	615	38.27	37.95	37.77	154	5	VEST	EXERCISE2	40	50
	616	38.29	37.95	37.77	156	5	VEST	EXERCISE2	40	50
	617	38.29	37.95	37.77	158	5	VEST	EXERCISE2	40	50
	618	38.28	37.95	37.78	158	5	VEST	EXERCISE2	40	50
	619	38.27	37.83	37.78	157	5	VEST	EXERCISE2	40	50
	620	38.28	37.81	37.77	158	5	VEST	EXERCISE2	40	50
	621	38.29	37.91	37.77	158	5	VEST	EXERCISE2	40	50
	622	38.30	37.84	37.78	158	5	VEST	EXERCISE2	40	50
	623	38.30	37.83	37.79	157	5	VEST	EXERCISE2	40	50
	624	38.30	37.90	37.78	158	5	VEST	EXERCISE2	40	50
	625	38.29	37.91	37.77	158	5	VEST	EXERCISE2	40	50
	626	38.29	37.92	37.77	158	5	VEST	EXERCISE2	40	50
	627	38.30	37.95	37.79	159	5	VEST	EXERCISE2	40	50

min	number	Tre	Tes	Tsk-head	HR	ID	condition	period	Ta	RH
	628	38.30	37.96	37.80	160	5	VEST	EXERCISE2	40	50
	629	38.31	37.96	37.80	158	5	VEST	EXERCISE2	40	50
	630	38.31	37.95	37.80	161	5	VEST	EXERCISE2	40	50
105	631	38.30	37.95	37.81	162	5	VEST	EXERCISE2	40	50
	632	38.30	37.96	37.82	162	5	VEST	EXERCISE2	40	50
	633	38.30	37.96	37.81	163	5	VEST	EXERCISE2	40	50
	634	38.31	37.95	37.81	166	5	VEST	EXERCISE2	40	50
	635	38.31	37.95	37.82	164	5	VEST	EXERCISE2	40	50
	636	38.32	37.96	37.83	162	5	VEST	EXERCISE2	40	50
	637	38.31	37.97	37.82	162	5	VEST	EXERCISE2	40	50
	638	38.32	37.87	37.81	163	5	VEST	EXERCISE2	40	50
	639	38.34	37.86	37.81	162	5	VEST	EXERCISE2	40	50
	640	38.35	37.89	37.81	162	5	VEST	EXERCISE2	40	50
	641	38.35	37.75	37.82	165	5	VEST	EXERCISE2	40	50
	642	38.36	37.75	37.82	164	5	VEST	EXERCISE2	40	50
	643	38.36	37.86	37.80	162	5	VEST	EXERCISE2	40	50
	644	38.36	37.92	37.76	158	5	VEST	REST3	28	50
	645	38.37	37.97	37.71	158	5	VEST	REST3	28	50
	646	38.37	37.99	37.65	157	5	VEST	REST3	28	50
	647	38.38	37.95	37.32	156	5	VEST	REST3	28	50
	648	38.39	37.93	36.95	149	5	VEST	REST3	28	50
	649	38.40	37.98	36.80	150	5	VEST	REST3	28	50
	650	38.40	38.00	36.71	151	5	VEST	REST3	28	50
	651	38.40	37.99	36.63	154	5	VEST	REST3	28	50
	652	38.40	37.97	36.61	146	5	VEST	REST3	28	50
	653	38.40	37.96	36.63	141	5	VEST	REST3	28	50
	654	38.39	37.94	36.60	134	5	VEST	REST3	28	50
	655	38.39	37.91	36.53	129	5	VEST	REST3	28	50
	656	38.40	37.88	36.36	132	5	VEST	REST3	28	50
	657	38.39	37.85	36.26	132	5	VEST	REST3	28	50
	658	38.39	37.83	36.25	129	5	VEST	REST3	28	50
	659	38.39	37.79	36.18	129	5	VEST	REST3	28	50
	660	38.39	37.76	36.04	128	5	VEST	REST3	28	50
110	661	38.39	37.72	35.99	131	5	VEST	REST3	28	50
	662	38.39	37.70	36.07	121	5	VEST	REST3	28	50
	663	38.39	37.70	36.02	122	5	VEST	REST3	28	50
	664	38.39	37.68	35.80	121	5	VEST	REST3	28	50
	665	38.39	37.66	35.75	125	5	VEST	REST3	28	50
	666	38.39	37.65	35.85	123	5	VEST	REST3	28	50
	667	38.38	37.63	35.85	122	5	VEST	REST3	28	50
	668	38.38	37.63	35.89	121	5	VEST	REST3	28	50
	669	38.37	37.64	35.93	119	5	VEST	REST3	28	50
	670	38.36	37.63	35.90	118	5	VEST	REST3	28	50
	671	38.36	37.62	35.85	118	5	VEST	REST3	28	50
	672	38.36	37.63	35.69	126	5	VEST	REST3	28	50
	673	38.36	37.63	35.60	123	5	VEST	REST3	28	50

min	number	Tre	Tes	Tsk-head	HR	ID	condition	period	Ta	RH
	674	38.35	37.64	35.60	121	5	VEST	REST3	28	50
	675	38.35	37.66	35.63	121	5	VEST	REST3	28	50
	676	38.34	37.67	35.70	119	5	VEST	REST3	28	50
	677	38.33	37.66	35.71	117	5	VEST	REST3	28	50
	678	38.33	37.67	35.69	117	5	VEST	REST3	28	50
	679	38.33	37.68	35.65	115	5	VEST	REST3	28	50
	680	38.32	37.67	35.65	119	5	VEST	REST3	28	50
	681	38.32	37.63	35.67	118	5	VEST	REST3	28	50
	682	38.31	37.62	35.68	118	5	VEST	REST3	28	50
	683	38.30	37.65	35.69	113	5	VEST	REST3	28	50
	684	38.28	37.66	35.65	120	5	VEST	REST3	28	50
	685	38.27	37.65	35.62	120	5	VEST	REST3	28	50
	686	38.27	37.64	35.64	114	5	VEST	REST3	28	50
	687	38.26	37.49	35.61	121	5	VEST	REST3	28	50
	688	38.25	37.45	35.58	118	5	VEST	REST3	28	50
	689	38.25	37.57	35.50	115	5	VEST	REST3	28	50
	690	38.24	37.59	35.36	113	5	VEST	REST3	28	50
115	691	38.24	37.62	35.26	118	5	VEST	REST3	28	50
	692	38.23	37.63	35.25	112	5	VEST	REST3	28	50
	693	38.22	37.64	35.28	108	5	VEST	REST3	28	50
	694	38.22	37.66	35.18	113	5	VEST	REST3	28	50
	695	38.22	37.65	35.07	116	5	VEST	REST3	28	50
	696	38.22	37.62	35.09	114	5	VEST	REST3	28	50
	697	38.22	37.63	35.20	115	5	VEST	REST3	28	50
	698	38.22	37.67	35.22	115	5	VEST	REST3	28	50
	699	38.22	37.69	35.15	113	5	VEST	REST3	28	50
	700	38.22	37.70	35.20	111	5	VEST	REST3	28	50
	701	38.23	37.71	35.35	116	5	VEST	REST3	28	50
	702	38.22	37.73	35.42	120	5	VEST	REST3	28	50
	703	38.20	37.62	35.38	113	5	VEST	REST3	28	50
	704	38.21	37.60	35.41	113	5	VEST	REST3	28	50
	705	38.21	37.70	35.39	102	5	VEST	REST3	28	50
	706	38.21	37.69	35.32	111	5	VEST	REST3	28	50
	707	38.21	37.67	35.37	107	5	VEST	REST3	28	50
	708	38.20	37.66	35.38	117	5	VEST	REST3	28	50
	709	38.20	37.69	35.37	116	5	VEST	REST3	28	50
0	1	36.74	36.91	35.33	77	6	VEST	REST1	28	50
	2	36.73	36.90	35.31	84	6	VEST	REST1	28	50
	3	36.73	36.88	35.30	87	6	VEST	REST1	28	50
	4	36.73	36.89	35.30	74	6	VEST	REST1	28	50
	5	36.74	36.87	35.30	76	6	VEST	REST1	28	50
	6	36.75	36.87	35.29	80	6	VEST	REST1	28	50
	7	36.75	36.88	35.28	81	6	VEST	REST1	28	50
	8	36.74	36.87	35.26	72	6	VEST	REST1	28	50
	9	36.74	36.89	35.25	72	6	VEST	REST1	28	50
	10	36.74	36.89	35.26	73	6	VEST	REST1	28	50

min	number	Tre	Tes	Tsk-head	HR	ID	condition	period	Ta	RH
	11	36.74	36.89	35.25	75	6	VEST	REST1	28	50
	12	36.74	36.87	35.25	78	6	VEST	REST1	28	50
	13	36.74	36.87	35.26	73	6	VEST	REST1	28	50
	14	36.74	36.87	35.25	71	6	VEST	REST1	28	50
	15	36.73	36.87	35.24	73	6	VEST	REST1	28	50
	16	36.73	36.86	35.24	73	6	VEST	REST1	28	50
	17	36.74	36.85	35.24	74	6	VEST	REST1	28	50
	18	36.74	36.84	35.24	76	6	VEST	REST1	28	50
	19	36.73	36.81	35.23	79	6	VEST	REST1	28	50
	20	36.73	36.82	35.23	73	6	VEST	REST1	28	50
	21	36.73	36.82	35.23	83	6	VEST	REST1	28	50
	22	36.73	36.78	35.22	79	6	VEST	REST1	28	50
	23	36.73	36.81	35.23	79	6	VEST	REST1	28	50
	24	36.73	36.81	35.22	82	6	VEST	REST1	28	50
	25	36.73	36.81	35.21	77	6	VEST	REST1	28	50
	26	36.73	36.82	35.21	71	6	VEST	REST1	28	50
	27	36.72	36.81	35.20	72	6	VEST	REST1	28	50
	28	36.73	36.84	35.20	73	6	VEST	REST1	28	50
	29	36.73	36.85	35.20	70	6	VEST	REST1	28	50
	30	36.73	36.83	35.20	77	6	VEST	REST1	28	50
5	31	36.73	36.76	35.21	81	6	VEST	REST1	28	50
	32	36.72	36.74	35.21	69	6	VEST	REST1	28	50
	33	36.72	36.79	35.21	74	6	VEST	REST1	28	50
	34	36.73	36.83	35.21	74	6	VEST	REST1	28	50
	35	36.73	36.82	35.21	73	6	VEST	REST1	28	50
	36	36.73	36.81	35.21	75	6	VEST	REST1	28	50
	37	36.73	36.82	35.22	74	6	VEST	REST1	28	50
	38	36.73	36.82	35.23	78	6	VEST	REST1	28	50
	39	36.73	36.70	35.24	81	6	VEST	REST1	28	50
	40	36.74	36.66	35.24	79	6	VEST	REST1	28	50
	41	36.74	36.76	35.25	86	6	VEST	REST1	28	50
	42	36.73	36.79	35.25	86	6	VEST	REST1	28	50
	43	36.73	36.79	35.26	79	6	VEST	REST1	28	50
	44	36.73	36.79	35.26	85	6	VEST	REST1	28	50
	45	36.73	36.80	35.27	81	6	VEST	REST1	28	50
	46	36.73	36.81	35.29	79	6	VEST	REST1	28	50
	47	36.73	36.81	35.30	74	6	VEST	REST1	28	50
	48	36.73	36.80	35.30	71	6	VEST	REST1	28	50
	49	36.73	36.82	35.30	74	6	VEST	REST1	28	50
	50	36.73	36.84	35.32	73	6	VEST	REST1	28	50
	51	36.72	36.86	35.34	75	6	VEST	REST1	28	50
	52	36.72	36.87	35.35	76	6	VEST	REST1	28	50
	53	36.74	36.87	35.38	72	6	VEST	REST1	28	50
	54	36.74	36.78	35.39	79	6	VEST	REST1	28	50
	55	36.74	36.74	35.40	79	6	VEST	REST1	28	50
	56	36.73	36.80	35.41	79	6	VEST	REST1	28	50

min	number	Tre	Tes	Tsk-head	HR	ID	condition	period	Ta	RH
	57	36.72	36.82	35.42	73	6	VEST	REST1	28	50
	58	36.72	36.83	35.45	76	6	VEST	REST1	28	50
	59	36.72	36.83	35.46	75	6	VEST	REST1	28	50
	60	36.72	36.83	35.47	75	6	VEST	REST1	28	50
10	61	36.74	36.85	35.49	74	6	VEST	REST1	28	50
	62	36.75	36.87	35.50	70	6	VEST	REST1	28	50
	63	36.75	36.87	35.50	74	6	VEST	REST1	28	50
	64	36.74	36.75	35.47	78	6	VEST	REST1	28	50
	65	36.74	36.71	35.46	77	6	VEST	REST1	28	50
	66	36.75	36.79	35.46	77	6	VEST	REST1	28	50
	67	36.75	36.82	35.45	76	6	VEST	REST1	28	50
	68	36.76	36.83	35.44	76	6	VEST	REST1	28	50
	69	36.76	36.84	35.43	74	6	VEST	REST1	28	50
	70	36.77	36.84	35.42	70	6	VEST	REST1	28	50
	71	36.77	36.84	35.42	75	6	VEST	REST1	28	50
	72	36.77	36.84	35.43	73	6	VEST	REST1	28	50
	73	36.77	36.86	35.46	69	6	VEST	REST1	28	50
	74	36.77	36.87	35.48	70	6	VEST	REST1	28	50
	75	36.75	36.86	35.47	72	6	VEST	REST1	28	50
	76	36.75	36.86	35.47	71	6	VEST	REST1	28	50
	77	36.76	36.86	35.46	72	6	VEST	REST1	28	50
	78	36.76	36.84	35.46	73	6	VEST	REST1	28	50
	79	36.76	36.83	35.46	74	6	VEST	REST1	28	50
	80	36.75	36.86	35.45	77	6	VEST	REST1	28	50
	81	36.75	36.84	35.45	78	6	VEST	REST1	28	50
	82	36.75	36.83	35.45	78	6	VEST	REST1	28	50
	83	36.75	36.84	35.46	78	6	VEST	REST1	28	50
	84	36.75	36.81	35.45	84	6	VEST	REST1	28	50
	85	36.74	36.78	35.44	95	6	VEST	REST1	28	50
	86	36.72	36.79	35.43	68	6	VEST	REST1	28	50
	87	36.71	36.79	35.42	82	6	VEST	REST1	28	50
	88	36.71	36.81	35.44	81	6	VEST	REST1	28	50
	89	36.73	36.75	35.45	78	6	VEST	REST1	28	50
	90	36.75	36.71	35.47	74	6	VEST	REST1	28	50
15	91	36.77	36.75	35.50	74	6	VEST	REST1	28	50
	92	36.78	36.77	35.52	74	6	VEST	REST1	28	50
	93	36.79	36.78	35.54	81	6	VEST	REST1	28	50
	94	36.79	36.79	35.56	82	6	VEST	REST1	28	50
	95	36.79	36.78	35.55	80	6	VEST	REST1	28	50
	96	36.80	36.79	35.54	77	6	VEST	REST1	28	50
	97	36.79	36.79	35.52	80	6	VEST	REST1	28	50
	98	36.79	36.76	35.51	77	6	VEST	REST1	28	50
	99	36.79	36.75	35.50	77	6	VEST	REST1	28	50
	100	36.80	36.77	35.48	78	6	VEST	REST1	28	50
	101	36.80	36.79	35.47	79	6	VEST	REST1	28	50
	102	36.79	36.79	35.45	79	6	VEST	REST1	28	50

min	number	Tre	Tes	Tsk-head	HR	ID	condition	period	Ta	RH
	103	36.78	36.77	35.43	78	6	VEST	REST1	40	50
	104	36.75	36.79	35.52	100	6	VEST	REST1	40	50
	105	36.74	36.82	35.69	85	6	VEST	REST1	40	50
	106	36.77	36.83	35.80	82	6	VEST	REST1	40	50
	107	36.79	36.83	35.86	82	6	VEST	REST1	40	50
	108	36.80	36.79	35.93	80	6	VEST	REST1	40	50
	109	36.81	36.76	35.99	80	6	VEST	REST1	40	50
	110	36.78	36.76	36.02	86	6	VEST	REST1	40	50
	111	36.74	36.75	36.05	80	6	VEST	REST1	40	50
	112	36.72	36.73	36.09	89	6	VEST	REST1	40	50
	113	36.71	36.72	36.13	91	6	VEST	REST1	40	50
	114	36.71	36.71	36.17	86	6	VEST	REST1	40	50
	115	36.74	36.69	36.20	85	6	VEST	REST1	40	50
	116	36.78	36.70	36.24	81	6	VEST	REST1	40	50
	117	36.81	36.72	36.26	82	6	VEST	REST1	40	50
	118	36.83	36.75	36.26	81	6	VEST	REST1	40	50
	119	36.84	36.75	36.28	79	6	VEST	REST1	40	50
	120	36.83	36.73	36.31	80	6	VEST	REST1	40	50
20	121	36.84	36.69	36.34	82	6	VEST	REST1	40	50
	122	36.86	36.70	36.34	78	6	VEST	REST1	40	50
	123	36.86	36.74	36.35	78	6	VEST	REST1	40	50
	124	36.86	36.74	36.37	78	6	VEST	REST1	40	50
	125	36.86	36.74	36.39	84	6	VEST	REST1	40	50
	126	36.85	36.76	36.38	82	6	VEST	REST1	40	50
	127	36.86	36.77	36.40	76	6	VEST	REST1	40	50
	128	36.87	36.79	36.41	83	6	VEST	REST1	40	50
	129	36.86	36.80	36.42	82	6	VEST	REST1	40	50
	130	36.86	36.79	36.43	79	6	VEST	REST1	40	50
	131	36.86	36.79	36.45	83	6	VEST	REST1	40	50
	132	36.86	36.78	36.47	80	6	VEST	REST1	40	50
	133	36.86	36.79	36.47	85	6	VEST	REST1	40	50
	134	36.83	36.80	36.46	98	6	VEST	REST1	40	50
	135	36.78	36.72	36.46	90	6	VEST	REST1	40	50
	136	36.78	36.70	36.48	83	6	VEST	REST1	40	50
	137	36.80	36.75	36.49	86	6	VEST	REST1	40	50
	138	36.81	36.77	36.52	85	6	VEST	REST1	40	50
	139	36.81	36.76	36.54	79	6	VEST	EXERCISE1	40	50
	140	36.80	36.76	36.54	89	6	VEST	EXERCISE1	40	50
	141	36.77	36.77	36.55	96	6	VEST	EXERCISE1	40	50
	142	36.74	36.74	36.53	99	6	VEST	EXERCISE1	40	50
	143	36.73	36.75	36.54	107	6	VEST	EXERCISE1	40	50
	144	36.72	36.80	36.59	110	6	VEST	EXERCISE1	40	50
	145	36.71	36.80	36.61	107	6	VEST	EXERCISE1	40	50
	146	36.71	36.73	36.61	110	6	VEST	EXERCISE1	40	50
	147	36.70	36.66	36.59	110	6	VEST	EXERCISE1	40	50
	148	36.70	36.64	36.58	113	6	VEST	EXERCISE1	40	50

min	number	Tre	Tes	Tsk-head	HR	ID	condition	period	Ta	RH
	149	36.69	36.65	36.60	112	6	VEST	EXERCISE1	40	50
	150	36.69	36.64	36.58	111	6	VEST	EXERCISE1	40	50
25	151	36.69	36.62	36.58	113	6	VEST	EXERCISE1	40	50
	152	36.69	36.58	36.58	112	6	VEST	EXERCISE1	40	50
	153	36.68	36.57	36.58	102	6	VEST	EXERCISE1	40	50
	154	36.68	36.59	36.59	105	6	VEST	EXERCISE1	40	50
	155	36.68	36.62	36.61	108	6	VEST	EXERCISE1	40	50
	156	36.68	36.66	36.63	108	6	VEST	EXERCISE1	40	50
	157	36.67	36.66	36.63	110	6	VEST	EXERCISE1	40	50
	158	36.67	36.60	36.63	110	6	VEST	EXERCISE1	40	50
	159	36.68	36.60	36.63	108	6	VEST	EXERCISE1	40	50
	160	36.68	36.66	36.64	109	6	VEST	EXERCISE1	40	50
	161	36.68	36.68	36.65	111	6	VEST	EXERCISE1	40	50
	162	36.67	36.68	36.64	111	6	VEST	EXERCISE1	40	50
	163	36.67	36.68	36.62	112	6	VEST	EXERCISE1	40	50
	164	36.68	36.69	36.62	113	6	VEST	EXERCISE1	40	50
	165	36.68	36.71	36.64	110	6	VEST	EXERCISE1	40	50
	166	36.68	36.73	36.65	109	6	VEST	EXERCISE1	40	50
	167	36.69	36.75	36.66	111	6	VEST	EXERCISE1	40	50
	168	36.70	36.76	36.65	111	6	VEST	EXERCISE1	40	50
	169	36.70	36.77	36.65	111	6	VEST	EXERCISE1	40	50
	170	36.69	36.77	36.63	112	6	VEST	EXERCISE1	40	50
	171	36.69	36.75	36.62	115	6	VEST	EXERCISE1	40	50
	172	36.69	36.76	36.64	115	6	VEST	EXERCISE1	40	50
	173	36.69	36.78	36.68	112	6	VEST	EXERCISE1	40	50
	174	36.70	36.78	36.70	112	6	VEST	EXERCISE1	40	50
	175	36.71	36.78	36.69	115	6	VEST	EXERCISE1	40	50
	176	36.71	36.80	36.71	118	6	VEST	EXERCISE1	40	50
	177	36.72	36.84	36.75	117	6	VEST	EXERCISE1	40	50
	178	36.72	36.85	36.76	120	6	VEST	EXERCISE1	40	50
	179	36.71	36.77	36.76	117	6	VEST	EXERCISE1	40	50
	180	36.71	36.74	36.75	113	6	VEST	EXERCISE1	40	50
30	181	36.71	36.80	36.75	112	6	VEST	EXERCISE1	40	50
	182	36.71	36.84	36.78	115	6	VEST	EXERCISE1	40	50
	183	36.72	36.84	36.80	116	6	VEST	EXERCISE1	40	50
	184	36.72	36.84	36.79	119	6	VEST	EXERCISE1	40	50
	185	36.73	36.85	36.79	121	6	VEST	EXERCISE1	40	50
	186	36.73	36.86	36.80	120	6	VEST	EXERCISE1	40	50
	187	36.74	36.72	36.82	120	6	VEST	EXERCISE1	40	50
	188	36.74	36.72	36.86	115	6	VEST	EXERCISE1	40	50
	189	36.74	36.85	36.86	115	6	VEST	EXERCISE1	40	50
	190	36.74	36.86	36.85	118	6	VEST	EXERCISE1	40	50
	191	36.75	36.89	36.87	118	6	VEST	EXERCISE1	40	50
	192	36.74	36.90	36.86	117	6	VEST	EXERCISE1	40	50
	193	36.76	36.91	36.87	118	6	VEST	EXERCISE1	40	50
	194	36.76	36.92	36.88	116	6	VEST	EXERCISE1	40	50

min	number	Tre	Tes	Tsk-head	HR	ID	condition	period	Ta	RH
	195	36.77	36.89	36.88	117	6	VEST	EXERCISE1	40	50
	196	36.77	36.88	36.86	120	6	VEST	EXERCISE1	40	50
	197	36.76	36.91	36.84	120	6	VEST	EXERCISE1	40	50
	198	36.77	36.94	36.86	122	6	VEST	EXERCISE1	40	50
	199	36.78	36.96	36.87	124	6	VEST	EXERCISE1	40	50
	200	36.78	36.95	36.84	125	6	VEST	EXERCISE1	40	50
	201	36.77	36.95	36.85	123	6	VEST	EXERCISE1	40	50
	202	36.77	36.96	36.87	124	6	VEST	EXERCISE1	40	50
	203	36.78	36.94	36.85	123	6	VEST	EXERCISE1	40	50
	204	36.78	36.90	36.85	126	6	VEST	EXERCISE1	40	50
	205	36.78	36.91	36.88	123	6	VEST	EXERCISE1	40	50
	206	36.79	36.94	36.90	123	6	VEST	EXERCISE1	40	50
	207	36.80	36.93	36.88	124	6	VEST	EXERCISE1	40	50
	208	36.80	36.92	36.87	123	6	VEST	EXERCISE1	40	50
	209	36.81	36.90	36.86	119	6	VEST	EXERCISE1	40	50
	210	36.82	36.89	36.83	119	6	VEST	EXERCISE1	40	50
35	211	36.82	36.91	36.83	121	6	VEST	EXERCISE1	40	50
	212	36.82	36.91	36.85	125	6	VEST	EXERCISE1	40	50
	213	36.83	36.89	36.83	126	6	VEST	EXERCISE1	40	50
	214	36.83	36.90	36.82	127	6	VEST	EXERCISE1	40	50
	215	36.83	36.91	36.84	123	6	VEST	EXERCISE1	40	50
	216	36.83	36.92	36.85	122	6	VEST	EXERCISE1	40	50
	217	36.84	36.90	36.83	123	6	VEST	EXERCISE1	40	50
	218	36.85	36.98	36.80	124	6	VEST	EXERCISE1	40	50
	219	36.85	37.02	36.79	127	6	VEST	EXERCISE1	40	50
	220	36.85	36.86	36.81	131	6	VEST	EXERCISE1	40	50
	221	36.86	36.83	36.83	128	6	VEST	EXERCISE1	40	50
	222	36.86	36.91	36.81	127	6	VEST	EXERCISE1	40	50
	223	36.87	36.92	36.82	127	6	VEST	EXERCISE1	40	50
	224	36.88	36.93	36.83	128	6	VEST	EXERCISE1	40	50
	225	36.88	36.96	36.83	123	6	VEST	EXERCISE1	40	50
	226	36.88	36.95	36.83	123	6	VEST	EXERCISE1	40	50
	227	36.89	36.93	36.81	121	6	VEST	EXERCISE1	40	50
	228	36.90	36.86	36.84	123	6	VEST	EXERCISE1	40	50
	229	36.90	36.86	36.85	123	6	VEST	EXERCISE1	40	50
	230	36.90	36.96	36.84	125	6	VEST	EXERCISE1	40	50
	231	36.90	36.97	36.84	129	6	VEST	EXERCISE1	40	50
	232	36.91	36.97	36.83	131	6	VEST	EXERCISE1	40	50
	233	36.91	36.97	36.82	129	6	VEST	EXERCISE1	40	50
	234	36.91	37.00	36.83	129	6	VEST	EXERCISE1	40	50
	235	36.92	37.02	36.85	128	6	VEST	EXERCISE1	40	50
	236	36.93	37.01	36.86	126	6	VEST	EXERCISE1	40	50
	237	36.93	37.01	36.84	121	6	VEST	EXERCISE1	40	50
	238	36.93	37.03	36.82	122	6	VEST	EXERCISE1	40	50
	239	36.94	36.99	36.84	126	6	VEST	EXERCISE1	40	50
	240	36.94	36.95	36.85	124	6	VEST	EXERCISE1	40	50

min	number	Tre	Tes	Tsk-head	HR	ID	condition	period	Ta	RH
40	241	36.94	36.99	36.82	122	6	VEST	EXERCISE1	40	50
	242	36.95	37.03	36.85	121	6	VEST	EXERCISE1	40	50
	243	36.95	37.03	36.85	125	6	VEST	EXERCISE1	40	50
	244	36.96	37.04	36.85	128	6	VEST	EXERCISE1	40	50
	245	36.96	37.03	36.86	133	6	VEST	EXERCISE1	40	50
	246	36.96	37.02	36.85	134	6	VEST	EXERCISE1	40	50
	247	36.96	37.06	36.86	131	6	VEST	EXERCISE1	40	50
	248	36.97	37.06	36.85	131	6	VEST	EXERCISE1	40	50
	249	36.98	37.06	36.85	129	6	VEST	EXERCISE1	40	50
	250	36.98	37.04	36.88	127	6	VEST	EXERCISE1	40	50
	251	36.99	37.03	36.88	122	6	VEST	EXERCISE1	40	50
	252	36.99	37.05	36.88	126	6	VEST	EXERCISE1	40	50
	253	37.00	37.06	36.91	132	6	VEST	EXERCISE1	40	50
	254	37.00	37.07	36.91	133	6	VEST	EXERCISE1	40	50
	255	37.01	37.09	36.90	133	6	VEST	EXERCISE1	40	50
	256	37.02	37.11	36.91	134	6	VEST	EXERCISE1	40	50
	257	37.02	37.10	36.91	134	6	VEST	EXERCISE1	40	50
	258	37.03	37.10	36.89	131	6	VEST	EXERCISE1	40	50
	259	37.04	37.12	36.90	128	6	VEST	EXERCISE1	40	50
	260	37.05	37.13	36.90	131	6	VEST	EXERCISE1	40	50
	261	37.05	37.12	36.87	131	6	VEST	EXERCISE1	40	50
	262	37.05	37.11	36.88	133	6	VEST	EXERCISE1	40	50
	263	37.06	37.05	36.91	131	6	VEST	EXERCISE1	40	50
	264	37.05	37.03	36.93	130	6	VEST	EXERCISE1	40	50
	265	37.05	37.08	36.93	134	6	VEST	EXERCISE1	40	50
	266	37.06	37.09	36.91	135	6	VEST	EXERCISE1	40	50
	267	37.07	37.10	36.91	135	6	VEST	EXERCISE1	40	50
	268	37.08	36.95	36.91	134	6	VEST	EXERCISE1	40	50
	269	37.09	36.91	36.90	134	6	VEST	EXERCISE1	40	50
	270	37.09	36.96	36.90	133	6	VEST	EXERCISE1	40	50
45	271	37.09	36.97	36.91	131	6	VEST	EXERCISE1	40	50
	272	37.09	37.06	36.90	131	6	VEST	EXERCISE1	40	50
	273	37.10	37.09	36.90	133	6	VEST	EXERCISE1	40	50
	274	37.10	37.12	36.96	131	6	VEST	EXERCISE1	40	50
	275	37.11	37.14	36.99	133	6	VEST	EXERCISE1	40	50
	276	37.12	37.12	36.96	136	6	VEST	EXERCISE1	40	50
	277	37.12	37.13	36.97	137	6	VEST	EXERCISE1	40	50
	278	37.13	37.14	36.98	134	6	VEST	EXERCISE1	40	50
	279	37.14	37.14	36.98	134	6	VEST	EXERCISE1	40	50
	280	37.14	37.16	36.95	133	6	VEST	EXERCISE1	40	50
	281	37.14	37.17	36.93	132	6	VEST	EXERCISE1	40	50
	282	37.15	37.20	36.97	133	6	VEST	EXERCISE1	40	50
	283	37.15	37.20	36.96	136	6	VEST	EXERCISE1	40	50
	284	37.15	37.01	36.93	140	6	VEST	EXERCISE1	40	50
	285	37.15	36.97	36.95	140	6	VEST	EXERCISE1	40	50
	286	37.14	37.12	36.96	137	6	VEST	EXERCISE1	40	50

min	number	Tre	Tes	Tsk-head	HR	ID	condition	period	Ta	RH
	287	37.14	37.15	36.96	134	6	VEST	EXERCISE1	40	50
	288	37.15	37.19	36.99	133	6	VEST	EXERCISE1	40	50
	289	37.15	37.21	37.01	132	6	VEST	EXERCISE1	40	50
	290	37.15	37.22	37.01	130	6	VEST	EXERCISE1	40	50
	291	37.16	37.23	37.02	130	6	VEST	EXERCISE1	40	50
	292	37.16	37.22	37.00	131	6	VEST	EXERCISE1	40	50
	293	37.17	37.24	36.99	132	6	VEST	EXERCISE1	40	50
	294	37.17	37.20	37.01	134	6	VEST	EXERCISE1	40	50
	295	37.17	37.17	37.03	134	6	VEST	EXERCISE1	40	50
	296	37.18	37.21	37.03	137	6	VEST	EXERCISE1	40	50
	297	37.19	37.22	37.01	137	6	VEST	EXERCISE1	40	50
	298	37.19	37.22	37.02	137	6	VEST	EXERCISE1	40	50
	299	37.19	37.23	37.03	137	6	VEST	EXERCISE1	40	50
	300	37.20	37.26	37.04	135	6	VEST	EXERCISE1	40	50
50	301	37.20	37.27	37.02	137	6	VEST	EXERCISE1	40	50
	302	37.20	37.28	37.00	136	6	VEST	EXERCISE1	40	50
	303	37.22	37.28	37.01	132	6	VEST	EXERCISE1	40	50
	304	37.24	37.29	37.02	131	6	VEST	EXERCISE1	40	50
	305	37.24	37.28	37.02	133	6	VEST	EXERCISE1	40	50
	306	37.25	37.25	37.00	134	6	VEST	EXERCISE1	40	50
	307	37.25	37.24	36.99	137	6	VEST	EXERCISE1	40	50
	308	37.25	37.26	37.00	138	6	VEST	EXERCISE1	40	50
	309	37.26	37.17	37.02	137	6	VEST	EXERCISE1	40	50
	310	37.25	37.14	37.05	134	6	VEST	EXERCISE1	40	50
	311	37.25	37.23	37.05	134	6	VEST	EXERCISE1	40	50
	312	37.25	37.26	37.03	133	6	VEST	EXERCISE1	40	50
	313	37.26	37.28	37.02	136	6	VEST	EXERCISE1	40	50
	314	37.26	37.30	37.03	137	6	VEST	EXERCISE1	40	50
	315	37.26	37.29	37.05	135	6	VEST	EXERCISE1	40	50
	316	37.27	37.24	37.03	134	6	VEST	EXERCISE1	40	50
	317	37.27	37.24	37.03	133	6	VEST	EXERCISE1	40	50
	318	37.28	37.26	37.05	134	6	VEST	EXERCISE1	40	50
	319	37.28	37.28	37.04	134	6	VEST	EXERCISE1	40	50
	320	37.28	37.31	37.05	128	6	VEST	REST2	28	50
	321	37.29	37.31	37.02	131	6	VEST	REST2	28	50
	322	37.29	37.31	36.89	122	6	VEST	REST2	28	50
	323	37.28	37.35	36.74	125	6	VEST	REST2	28	50
	324	37.29	37.31	36.65	122	6	VEST	REST2	28	50
	325	37.30	37.29	36.57	112	6	VEST	REST2	28	50
	326	37.30	37.30	36.56	106	6	VEST	REST2	28	50
	327	37.30	37.25	36.57	109	6	VEST	REST2	28	50
	328	37.30	37.24	36.48	110	6	VEST	REST2	28	50
	329	37.31	37.30	36.44	109	6	VEST	REST2	28	50
	330	37.31	37.36	36.44	115	6	VEST	REST2	28	50
55	331	37.31	37.38	36.39	111	6	VEST	REST2	28	50
	332	37.32	37.35	36.38	108	6	VEST	REST2	28	50

min	number	Tre	Tes	Tsk-head	HR	ID	condition	period	Ta	RH
	333	37.32	37.31	36.37	106	6	VEST	REST2	28	50
	334	37.32	37.28	36.25	107	6	VEST	REST2	28	50
	335	37.33	37.22	36.14	116	6	VEST	REST2	28	50
	336	37.34	37.22	36.10	109	6	VEST	REST2	28	50
	337	37.33	37.24	36.08	104	6	VEST	REST2	28	50
	338	37.33	33.98	36.03	105	6	VEST	REST2	28	50
	339	37.33	32.44	35.98	103	6	VEST	REST2	28	50
	340	37.33	30.79	35.99	104	6	VEST	REST2	28	50
	341	37.33	29.55	35.93	95	6	VEST	REST2	28	50
	342	37.34	32.21	35.85	107	6	VEST	REST2	28	50
	343	37.35	33.39	35.86	97	6	VEST	REST2	28	50
	344	37.34	34.26	35.89	99	6	VEST	REST2	28	50
	345	37.34	34.63	35.92	89	6	VEST	REST2	28	50
	346	37.34	34.94	35.91	90	6	VEST	REST2	28	50
	347	37.35	35.23	35.92	87	6	VEST	REST2	28	50
	348	37.35	35.41	35.97	91	6	VEST	REST2	28	50
	349	37.35	35.52	35.97	88	6	VEST	REST2	28	50
	350	37.35	31.89	35.96	91	6	VEST	REST2	28	50
	351	37.35	28.32	35.95	94	6	VEST	REST2	28	50
	352	37.36	29.99	35.92	95	6	VEST	REST2	28	50
	353	37.36	32.11	35.88	91	6	VEST	REST2	28	50
	354	37.35	33.02	35.87	86	6	VEST	REST2	28	50
	355	37.35	33.44	35.87	90	6	VEST	REST2	28	50
	356	37.35	33.72	35.87	92	6	VEST	REST2	28	50
	357	37.35	34.20	35.82	92	6	VEST	REST2	28	50
	358	37.35	34.76	35.79	94	6	VEST	REST2	28	50
	359	37.35	35.10	35.81	92	6	VEST	REST2	28	50
	360	37.36	35.16	35.82	87	6	VEST	REST2	28	50
60	361	37.36	35.24	35.82	84	6	VEST	REST2	28	50
	362	37.36	35.59	35.80	86	6	VEST	REST2	28	50
	363	37.36	35.87	35.80	80	6	VEST	REST2	28	50
	364	37.35	36.00	35.79	91	6	VEST	REST2	28	50
	365	37.35	36.13	35.78	90	6	VEST	REST2	28	50
	366	37.36	36.21	35.79	82	6	VEST	REST2	28	50
	367	37.36	32.45	35.75	86	6	VEST	REST2	28	50
	368	37.36	27.44	35.73	93	6	VEST	REST2	28	50
	369	37.36	27.74	35.72	96	6	VEST	REST2	28	50
	370	37.36	29.47	35.70	86	6	VEST	REST2	28	50
	371	37.37	30.62	35.68	86	6	VEST	REST2	28	50
	372	37.37	31.81	35.67	86	6	VEST	REST2	28	50
	373	37.36	32.32	35.65	85	6	VEST	REST2	28	50
	374	37.36	32.77	35.63	89	6	VEST	REST2	28	50
	375	37.36	33.17	35.62	90	6	VEST	REST2	28	50
	376	37.36	33.68	35.60	90	6	VEST	REST2	28	50
	377	37.35	34.26	35.54	86	6	VEST	REST2	28	50
	378	37.35	34.60	35.48	88	6	VEST	REST2	28	50

min	number	Tre	Tes	Tsk-head	HR	ID	condition	period	Ta	RH
	379	37.36	34.81	35.45	88	6	VEST	REST2	28	50
	380	37.35	35.16	35.47	88	6	VEST	REST2	28	50
	381	37.35	35.57	35.46	87	6	VEST	REST2	28	50
	382	37.36	31.56	35.44	93	6	VEST	REST2	28	50
	383	37.36	29.80	35.43	92	6	VEST	REST2	28	50
	384	37.35	32.72	35.39	87	6	VEST	REST2	28	50
	385	37.35	33.34	35.37	85	6	VEST	REST2	28	50
	386	37.35	30.62	35.35	81	6	VEST	REST2	28	50
	387	37.35	28.25	35.33	84	6	VEST	REST2	28	50
	388	37.35	30.65	35.35	84	6	VEST	REST2	28	50
	389	37.34	30.76	35.33	91	6	VEST	REST2	28	50
	390	37.34	29.44	35.29	86	6	VEST	REST2	28	50
65	391	37.35	30.23	35.29	82	6	VEST	REST2	28	50
	392	37.35	30.87	35.29	79	6	VEST	REST2	28	50
	393	37.36	31.36	35.27	81	6	VEST	REST2	28	50
	394	37.36	31.98	35.25	82	6	VEST	REST2	28	50
	395	37.35	32.61	35.22	80	6	VEST	REST2	28	50
	396	37.35	33.25	35.17	80	6	VEST	REST2	28	50
	397	37.34	33.87	35.13	79	6	VEST	REST2	28	50
	398	37.34	34.17	35.11	81	6	VEST	REST2	28	50
	399	37.35	34.56	35.09	80	6	VEST	REST2	28	50
	400	37.34	34.89	35.06	80	6	VEST	REST2	28	50
	401	37.34	35.08	35.02	79	6	VEST	REST2	28	50
	402	37.35	35.23	34.97	79	6	VEST	REST2	28	50
	403	37.35	35.39	34.94	81	6	VEST	REST2	28	50
	404	37.35	35.50	34.91	80	6	VEST	REST2	28	50
	405	37.34	35.62	34.88	81	6	VEST	REST2	28	50
	406	37.34	35.75	34.87	80	6	VEST	REST2	28	50
	407	37.35	35.83	34.87	80	6	VEST	REST2	28	50
	408	37.35	35.93	34.86	79	6	VEST	REST2	28	50
	409	37.34	36.03	34.86	81	6	VEST	REST2	28	50
	410	37.33	36.12	34.88	82	6	VEST	REST2	28	50
	411	37.33	36.20	34.89	80	6	VEST	REST2	28	50
	412	37.32	36.35	34.89	85	6	VEST	REST2	28	50
	413	37.32	36.43	34.89	83	6	VEST	REST2	28	50
	414	37.32	36.43	34.87	81	6	VEST	REST2	28	50
	415	37.32	36.55	34.88	81	6	VEST	REST2	28	50
	416	37.32	36.63	34.84	80	6	VEST	REST2	28	50
	417	37.31	36.69	34.75	77	6	VEST	REST2	28	50
	418	37.31	36.73	34.74	83	6	VEST	REST2	28	50
	419	37.31	36.77	34.78	84	6	VEST	REST2	28	50
	420	37.30	36.82	34.80	81	6	VEST	REST2	28	50
70	421	37.30	36.86	34.82	81	6	VEST	REST2	28	50
	422	37.31	36.89	34.83	78	6	VEST	REST2	28	50
	423	37.31	36.42	34.84	78	6	VEST	REST2	28	50
	424	37.31	36.06	34.84	75	6	VEST	REST2	28	50

min	number	Tre	Tes	Tsk-head	HR	ID	condition	period	Ta	RH
	425	37.32	36.47	34.83	89	6	VEST	REST2	28	50
	426	37.33	36.81	34.83	83	6	VEST	REST2	28	50
	427	37.33	36.86	34.84	82	6	VEST	REST2	28	50
	428	37.33	36.88	34.85	82	6	VEST	REST2	28	50
	429	37.33	36.91	34.84	77	6	VEST	REST2	28	50
	430	37.33	36.93	34.83	78	6	VEST	REST2	28	50
	431	37.34	36.96	34.81	79	6	VEST	REST2	28	50
	432	37.34	36.97	34.80	80	6	VEST	REST2	28	50
	433	37.35	36.97	34.77	81	6	VEST	REST2	28	50
	434	37.34	36.98	34.74	95	6	VEST	REST2	28	50
	435	37.32	37.00	34.76	87	6	VEST	REST2	28	50
	436	37.32	37.00	34.76	85	6	VEST	REST2	28	50
	437	37.33	37.00	34.75	77	6	VEST	REST2	28	50
	438	37.32	37.00	34.75	75	6	VEST	REST2	28	50
	439	37.33	37.01	34.74	77	6	VEST	REST2	40	50
	440	37.33	37.01	34.80	92	6	VEST	REST2	40	50
	441	37.33	37.01	34.96	94	6	VEST	REST2	40	50
	442	37.34	37.01	35.12	93	6	VEST	REST2	40	50
	443	37.34	37.02	35.24	87	6	VEST	REST2	40	50
	444	37.33	37.02	35.34	88	6	VEST	REST2	40	50
	445	37.33	37.03	35.43	80	6	VEST	REST2	40	50
	446	37.32	37.03	35.49	83	6	VEST	REST2	40	50
	447	37.32	36.99	35.54	87	6	VEST	REST2	40	50
	448	37.32	36.98	35.62	84	6	VEST	REST2	40	50
	449	37.32	36.99	35.67	83	6	VEST	REST2	40	50
	450	37.33	36.98	35.70	84	6	VEST	REST2	40	50
75	451	37.32	37.00	35.75	83	6	VEST	REST2	40	50
	452	37.32	37.02	35.79	86	6	VEST	REST2	40	50
	453	37.32	36.99	35.82	85	6	VEST	REST2	40	50
	454	37.32	36.96	35.85	87	6	VEST	REST2	40	50
	455	37.32	36.97	35.88	86	6	VEST	REST2	40	50
	456	37.32	36.98	35.92	86	6	VEST	REST2	40	50
	457	37.32	37.02	35.95	91	6	VEST	REST2	40	50
	458	37.34	37.05	35.97	118	6	VEST	REST2	40	50
	459	37.35	37.02	35.99	105	6	VEST	REST2	40	50
	460	37.35	37.01	36.00	103	6	VEST	REST2	40	50
	461	37.35	37.00	36.05	94	6	VEST	REST2	40	50
	462	37.36	36.95	36.09	85	6	VEST	REST2	40	50
	463	37.37	36.94	36.13	86	6	VEST	EXERCISE2	40	50
	464	37.38	36.97	36.16	93	6	VEST	EXERCISE2	40	50
	465	37.37	36.98	36.16	102	6	VEST	EXERCISE2	40	50
	466	37.37	37.00	36.15	102	6	VEST	EXERCISE2	40	50
	467	37.37	37.01	36.14	108	6	VEST	EXERCISE2	40	50
	468	37.37	36.96	36.17	108	6	VEST	EXERCISE2	40	50
	469	37.37	36.96	36.20	110	6	VEST	EXERCISE2	40	50
	470	37.37	36.97	36.22	109	6	VEST	EXERCISE2	40	50

min	number	Tre	Tes	Tsk-head	HR	ID	condition	period	Ta	RH
	471	37.37	36.95	36.24	109	6	VEST	EXERCISE2	40	50
	472	37.37	36.99	36.27	110	6	VEST	EXERCISE2	40	50
	473	37.36	37.01	36.29	113	6	VEST	EXERCISE2	40	50
	474	37.35	37.00	36.30	111	6	VEST	EXERCISE2	40	50
	475	37.35	37.01	36.33	110	6	VEST	EXERCISE2	40	50
	476	37.35	37.04	36.36	113	6	VEST	EXERCISE2	40	50
	477	37.36	37.05	36.35	115	6	VEST	EXERCISE2	40	50
	478	37.35	37.05	36.35	115	6	VEST	EXERCISE2	40	50
	479	37.35	37.04	36.40	112	6	VEST	EXERCISE2	40	50
	480	37.35	37.01	36.43	112	6	VEST	EXERCISE2	40	50
80	481	37.36	37.01	36.43	112	6	VEST	EXERCISE2	40	50
	482	37.35	37.03	36.43	113	6	VEST	EXERCISE2	40	50
	483	37.34	37.05	36.46	115	6	VEST	EXERCISE2	40	50
	484	37.34	37.07	36.50	114	6	VEST	EXERCISE2	40	50
	485	37.35	37.08	36.51	115	6	VEST	EXERCISE2	40	50
	486	37.34	37.09	36.54	115	6	VEST	EXERCISE2	40	50
	487	37.33	37.09	36.57	115	6	VEST	EXERCISE2	40	50
	488	37.34	37.04	36.59	117	6	VEST	EXERCISE2	40	50
	489	37.35	37.02	36.61	113	6	VEST	EXERCISE2	40	50
	490	37.35	37.08	36.63	114	6	VEST	EXERCISE2	40	50
	491	37.35	37.10	36.66	113	6	VEST	EXERCISE2	40	50
	492	37.36	37.12	36.69	114	6	VEST	EXERCISE2	40	50
	493	37.36	37.13	36.69	116	6	VEST	EXERCISE2	40	50
	494	37.36	37.12	36.69	115	6	VEST	EXERCISE2	40	50
	495	37.36	37.13	36.71	131	6	VEST	EXERCISE2	40	50
	496	37.37	37.14	36.74	117	6	VEST	EXERCISE2	40	50
	497	37.37	37.14	36.75	117	6	VEST	EXERCISE2	40	50
	498	37.37	37.09	36.74	120	6	VEST	EXERCISE2	40	50
	499	37.36	37.05	36.74	119	6	VEST	EXERCISE2	40	50
	500	37.36	37.07	36.76	136	6	VEST	EXERCISE2	40	50
	501	37.36	37.10	36.78	120	6	VEST	EXERCISE2	40	50
	502	37.35	37.13	36.81	122	6	VEST	EXERCISE2	40	50
	503	37.36	37.14	36.82	117	6	VEST	EXERCISE2	40	50
	504	37.36	37.15	36.83	120	6	VEST	EXERCISE2	40	50
	505	37.35	37.09	36.83	123	6	VEST	EXERCISE2	40	50
	506	37.36	37.07	36.83	117	6	VEST	EXERCISE2	40	50
	507	37.36	37.12	36.84	118	6	VEST	EXERCISE2	40	50
	508	37.37	37.10	36.84	119	6	VEST	EXERCISE2	40	50
	509	37.36	37.09	36.83	123	6	VEST	EXERCISE2	40	50
	510	37.35	37.12	36.81	122	6	VEST	EXERCISE2	40	50
85	511	37.36	37.14	36.80	122	6	VEST	EXERCISE2	40	50
	512	37.37	37.14	36.82	119	6	VEST	EXERCISE2	40	50
	513	37.36	37.15	36.82	118	6	VEST	EXERCISE2	40	50
	514	37.36	37.05	36.82	120	6	VEST	EXERCISE2	40	50
	515	37.37	37.03	36.82	115	6	VEST	EXERCISE2	40	50
	516	37.37	37.13	36.81	116	6	VEST	EXERCISE2	40	50

min	number	Tre	Tes	Tsk-head	HR	ID	condition	period	Ta	RH
	517	37.37	37.15	36.82	116	6	VEST	EXERCISE2	40	50
	518	37.37	37.16	36.81	120	6	VEST	EXERCISE2	40	50
	519	37.37	37.18	36.80	119	6	VEST	EXERCISE2	40	50
	520	37.37	37.19	36.82	121	6	VEST	EXERCISE2	40	50
	521	37.37	37.19	36.83	121	6	VEST	EXERCISE2	40	50
	522	37.37	37.15	36.83	123	6	VEST	EXERCISE2	40	50
	523	37.37	37.13	36.84	126	6	VEST	EXERCISE2	40	50
	524	37.38	37.16	36.82	110	6	VEST	EXERCISE2	40	50
	525	37.37	37.16	36.80	124	6	VEST	EXERCISE2	40	50
	526	37.37	37.17	36.82	122	6	VEST	EXERCISE2	40	50
	527	37.38	37.19	36.84	122	6	VEST	EXERCISE2	40	50
	528	37.38	37.20	36.85	123	6	VEST	EXERCISE2	40	50
	529	37.38	37.20	36.86	123	6	VEST	EXERCISE2	40	50
	530	37.38	37.19	36.86	123	6	VEST	EXERCISE2	40	50
	531	37.38	37.18	36.84	124	6	VEST	EXERCISE2	40	50
	532	37.38	37.15	36.82	125	6	VEST	EXERCISE2	40	50
	533	37.39	37.13	36.84	132	6	VEST	EXERCISE2	40	50
	534	37.40	37.15	36.86	123	6	VEST	EXERCISE2	40	50
	535	37.39	37.16	36.85	123	6	VEST	EXERCISE2	40	50
	536	37.39	37.18	36.85	124	6	VEST	EXERCISE2	40	50
	537	37.39	37.24	36.84	121	6	VEST	EXERCISE2	40	50
	538	37.39	37.27	36.84	124	6	VEST	EXERCISE2	40	50
	539	37.39	37.23	36.84	127	6	VEST	EXERCISE2	40	50
	540	37.39	37.13	36.86	126	6	VEST	EXERCISE2	40	50
90	541	37.40	37.09	36.83	125	6	VEST	EXERCISE2	40	50
	542	37.39	37.13	36.82	124	6	VEST	EXERCISE2	40	50
	543	37.40	37.16	36.84	124	6	VEST	EXERCISE2	40	50
	544	37.41	37.18	36.85	125	6	VEST	EXERCISE2	40	50
	545	37.41	37.17	36.87	148	6	VEST	EXERCISE2	40	50
	546	37.41	37.16	36.87	128	6	VEST	EXERCISE2	40	50
	547	37.41	37.18	36.86	127	6	VEST	EXERCISE2	40	50
	548	37.42	37.06	36.84	131	6	VEST	EXERCISE2	40	50
	549	37.41	37.04	36.84	130	6	VEST	EXERCISE2	40	50
	550	37.41	37.15	36.87	124	6	VEST	EXERCISE2	40	50
	551	37.41	37.17	36.87	124	6	VEST	EXERCISE2	40	50
	552	37.41	37.20	36.87	124	6	VEST	EXERCISE2	40	50
	553	37.42	37.22	36.89	122	6	VEST	EXERCISE2	40	50
	554	37.41	37.23	36.90	124	6	VEST	EXERCISE2	40	50
	555	37.41	37.24	36.92	126	6	VEST	EXERCISE2	40	50
	556	37.41	37.18	36.93	146	6	VEST	EXERCISE2	40	50
	557	37.41	37.15	36.91	131	6	VEST	EXERCISE2	40	50
	558	37.41	37.19	36.91	131	6	VEST	EXERCISE2	40	50
	559	37.42	37.21	36.91	133	6	VEST	EXERCISE2	40	50
	560	37.42	37.22	36.88	151	6	VEST	EXERCISE2	40	50
	561	37.43	37.12	36.89	131	6	VEST	EXERCISE2	40	50
	562	37.43	37.12	36.86	128	6	VEST	EXERCISE2	40	50

min	number	Tre	Tes	Tsk-head	HR	ID	condition	period	Ta	RH
	563	37.43	37.22	36.85	144	6	VEST	EXERCISE2	40	50
	564	37.43	37.24	36.89	129	6	VEST	EXERCISE2	40	50
	565	37.43	37.25	36.91	130	6	VEST	EXERCISE2	40	50
	566	37.43	37.25	36.91	129	6	VEST	EXERCISE2	40	50
	567	37.42	37.25	36.90	128	6	VEST	EXERCISE2	40	50
	568	37.42	37.27	36.89	126	6	VEST	EXERCISE2	40	50
	569	37.42	37.27	36.88	126	6	VEST	EXERCISE2	40	50
	570	37.42	37.26	36.87	125	6	VEST	EXERCISE2	40	50
95	571	37.42	37.27	36.89	125	6	VEST	EXERCISE2	40	50
	572	37.43	37.27	36.89	125	6	VEST	EXERCISE2	40	50
	573	37.43	37.28	36.87	129	6	VEST	EXERCISE2	40	50
	574	37.44	37.26	36.86	134	6	VEST	EXERCISE2	40	50
	575	37.44	37.24	36.87	132	6	VEST	EXERCISE2	40	50
	576	37.44	37.26	36.90	134	6	VEST	EXERCISE2	40	50
	577	37.44	37.27	36.91	133	6	VEST	EXERCISE2	40	50
	578	37.44	37.29	36.92	134	6	VEST	EXERCISE2	40	50
	579	37.44	37.30	36.93	131	6	VEST	EXERCISE2	40	50
	580	37.44	37.32	36.94	131	6	VEST	EXERCISE2	40	50
	581	37.45	37.32	36.93	130	6	VEST	EXERCISE2	40	50
	582	37.45	37.31	36.93	131	6	VEST	EXERCISE2	40	50
	583	37.45	37.23	36.94	131	6	VEST	EXERCISE2	40	50
	584	37.45	37.20	36.93	134	6	VEST	EXERCISE2	40	50
	585	37.46	37.26	36.93	135	6	VEST	EXERCISE2	40	50
	586	37.45	37.29	36.94	133	6	VEST	EXERCISE2	40	50
	587	37.46	37.31	36.95	132	6	VEST	EXERCISE2	40	50
	588	37.47	37.32	36.95	132	6	VEST	EXERCISE2	40	50
	589	37.48	37.23	36.94	133	6	VEST	EXERCISE2	40	50
	590	37.47	37.19	36.93	129	6	VEST	EXERCISE2	40	50
	591	37.47	37.27	36.95	127	6	VEST	EXERCISE2	40	50
	592	37.48	37.31	36.97	128	6	VEST	EXERCISE2	40	50
	593	37.49	37.34	36.97	133	6	VEST	EXERCISE2	40	50
	594	37.49	37.34	36.98	134	6	VEST	EXERCISE2	40	50
	595	37.50	37.33	36.99	135	6	VEST	EXERCISE2	40	50
	596	37.51	37.31	36.98	152	6	VEST	EXERCISE2	40	50
	597	37.52	37.30	36.96	133	6	VEST	EXERCISE2	40	50
	598	37.51	37.34	36.96	131	6	VEST	EXERCISE2	40	50
	599	37.50	37.37	36.97	131	6	VEST	EXERCISE2	40	50
	600	37.51	37.37	36.97	129	6	VEST	EXERCISE2	40	50
100	601	37.51	37.34	36.95	134	6	VEST	EXERCISE2	40	50
	602	37.51	37.29	36.94	137	6	VEST	EXERCISE2	40	50
	603	37.52	37.30	36.96	138	6	VEST	EXERCISE2	40	50
	604	37.53	37.34	36.97	137	6	VEST	EXERCISE2	40	50
	605	37.53	37.34	36.96	136	6	VEST	EXERCISE2	40	50
	606	37.54	37.33	36.96	134	6	VEST	EXERCISE2	40	50
	607	37.55	37.34	36.98	132	6	VEST	EXERCISE2	40	50
	608	37.55	37.35	36.99	131	6	VEST	EXERCISE2	40	50

min	number	Tre	Tes	Tsk-head	HR	ID	condition	period	Ta	RH
	609	37.55	37.35	36.97	131	6	VEST	EXERCISE2	40	50
	610	37.55	37.35	36.97	132	6	VEST	EXERCISE2	40	50
	611	37.55	37.37	36.97	134	6	VEST	EXERCISE2	40	50
	612	37.56	37.28	36.95	137	6	VEST	EXERCISE2	40	50
	613	37.57	37.24	36.94	136	6	VEST	EXERCISE2	40	50
	614	37.57	37.33	36.96	137	6	VEST	EXERCISE2	40	50
	615	37.56	37.35	36.98	137	6	VEST	EXERCISE2	40	50
	616	37.57	37.32	36.98	135	6	VEST	EXERCISE2	40	50
	617	37.57	37.32	36.98	135	6	VEST	EXERCISE2	40	50
	618	37.57	37.35	36.98	135	6	VEST	EXERCISE2	40	50
	619	37.58	37.34	36.99	136	6	VEST	EXERCISE2	40	50
	620	37.58	37.33	37.01	136	6	VEST	EXERCISE2	40	50
	621	37.59	37.33	37.01	134	6	VEST	EXERCISE2	40	50
	622	37.59	37.35	37.02	133	6	VEST	EXERCISE2	40	50
	623	37.60	37.39	37.03	133	6	VEST	EXERCISE2	40	50
	624	37.61	37.41	37.03	150	6	VEST	EXERCISE2	40	50
	625	37.61	37.40	37.03	135	6	VEST	EXERCISE2	40	50
	626	37.62	37.28	37.03	138	6	VEST	EXERCISE2	40	50
	627	37.62	37.25	37.03	134	6	VEST	EXERCISE2	40	50
	628	37.62	37.34	37.04	135	6	VEST	EXERCISE2	40	50
	629	37.62	37.39	37.06	135	6	VEST	EXERCISE2	40	50
	630	37.63	37.41	37.08	136	6	VEST	EXERCISE2	40	50
105	631	37.63	37.40	37.11	134	6	VEST	EXERCISE2	40	50
	632	37.63	37.38	37.12	136	6	VEST	EXERCISE2	40	50
	633	37.64	37.39	37.12	135	6	VEST	EXERCISE2	40	50
	634	37.64	37.42	37.12	134	6	VEST	EXERCISE2	40	50
	635	37.64	37.41	37.10	137	6	VEST	EXERCISE2	40	50
	636	37.64	37.44	37.11	141	6	VEST	EXERCISE2	40	50
	637	37.63	37.49	37.12	140	6	VEST	EXERCISE2	40	50
	638	37.63	37.51	37.11	138	6	VEST	EXERCISE2	40	50
	639	37.64	37.45	37.09	134	6	VEST	EXERCISE2	40	50
	640	37.65	37.45	37.11	133	6	VEST	EXERCISE2	40	50
	641	37.64	37.48	37.12	134	6	VEST	EXERCISE2	40	50
	642	37.64	37.47	37.11	134	6	VEST	EXERCISE2	40	50
	643	37.65	37.47	37.10	136	6	VEST	EXERCISE2	40	50
	644	37.65	37.50	37.10	134	6	VEST	REST3	28	50
	645	37.66	37.54	37.07	135	6	VEST	REST3	28	50
	646	37.67	37.56	36.95	130	6	VEST	REST3	28	50
	647	37.68	37.56	36.77	114	6	VEST	REST3	28	50
	648	37.69	37.50	36.66	117	6	VEST	REST3	28	50
	649	37.68	37.46	36.59	107	6	VEST	REST3	28	50
	650	37.67	37.36	36.49	115	6	VEST	REST3	28	50
	651	37.66	37.31	36.45	112	6	VEST	REST3	28	50
	652	37.65	37.40	36.42	110	6	VEST	REST3	28	50
	653	37.65	37.42	36.38	103	6	VEST	REST3	28	50
	654	37.65	37.43	36.36	100	6	VEST	REST3	28	50

min	number	Tre	Tes	Tsk-head	HR	ID	condition	period	Ta	RH
	655	37.64	37.39	36.32	101	6	VEST	REST3	28	50
	656	37.63	37.38	36.27	102	6	VEST	REST3	28	50
	657	37.63	37.42	36.24	100	6	VEST	REST3	28	50
	658	37.64	37.42	36.19	101	6	VEST	REST3	28	50
	659	37.64	37.40	36.16	99	6	VEST	REST3	28	50
	660	37.63	37.38	36.13	100	6	VEST	REST3	28	50
110	661	37.62	37.37	36.08	99	6	VEST	REST3	28	50
	662	37.62	37.38	36.03	99	6	VEST	REST3	28	50
	663	37.62	37.36	35.97	99	6	VEST	REST3	28	50
	664	37.62	37.35	35.93	98	6	VEST	REST3	28	50
	665	37.62	37.32	35.89	101	6	VEST	REST3	28	50
	666	37.62	37.30	35.84	98	6	VEST	REST3	28	50
	667	37.63	37.31	35.82	96	6	VEST	REST3	28	50
	668	37.63	37.33	35.78	96	6	VEST	REST3	28	50
	669	37.63	37.33	35.74	97	6	VEST	REST3	28	50
	670	37.64	37.32	35.72	97	6	VEST	REST3	28	50
	671	37.65	37.31	35.69	100	6	VEST	REST3	28	50
	672	37.65	37.28	35.60	95	6	VEST	REST3	28	50
	673	37.63	37.29	35.51	97	6	VEST	REST3	28	50
	674	37.63	37.23	35.49	99	6	VEST	REST3	28	50
	675	37.63	37.20	35.46	94	6	VEST	REST3	28	50
	676	37.63	37.28	35.41	92	6	VEST	REST3	28	50
	677	37.63	37.30	35.41	95	6	VEST	REST3	28	50
	678	37.63	37.29	35.40	93	6	VEST	REST3	28	50
	679	37.64	37.28	35.34	93	6	VEST	REST3	28	50
	680	37.64	37.31	35.32	90	6	VEST	REST3	28	50
	681	37.65	37.36	35.29	92	6	VEST	REST3	28	50
	682	37.64	37.36	35.20	91	6	VEST	REST3	28	50
	683	37.64	37.17	35.17	98	6	VEST	REST3	28	50
	684	37.64	37.12	35.10	101	6	VEST	REST3	28	50
	685	37.63	37.25	35.03	84	6	VEST	REST3	28	50
	686	37.63	37.26	35.06	91	6	VEST	REST3	28	50
	687	37.62	37.23	35.04	95	6	VEST	REST3	28	50
	688	37.63	37.23	35.01	90	6	VEST	REST3	28	50
	689	37.64	37.20	35.02	86	6	VEST	REST3	28	50
	690	37.64	37.19	35.06	88	6	VEST	REST3	28	50
115	691	37.65	37.24	35.07	90	6	VEST	REST3	28	50
	692	37.66	37.27	35.08	87	6	VEST	REST3	28	50
	693	37.67	37.31	35.08	90	6	VEST	REST3	28	50
	694	37.66	37.31	35.07	91	6	VEST	REST3	28	50
	695	37.66	37.30	35.06	90	6	VEST	REST3	28	50
	696	37.66	37.30	35.03	90	6	VEST	REST3	28	50
	697	37.66	37.30	35.04	90	6	VEST	REST3	28	50
	698	37.67	37.31	35.05	93	6	VEST	REST3	28	50
	699	37.67	37.34	35.00	90	6	VEST	REST3	28	50
	700	37.67	37.33	34.98	89	6	VEST	REST3	28	50

min	number	Tre	Tes	Tsk-head	HR	ID	condition	period	Ta	RH
	701	37.67	37.33	35.01	94	6	VEST	REST3	28	50
	702	37.66	37.35	34.99	91	6	VEST	REST3	28	50
	703	37.66	37.34	34.99	85	6	VEST	REST3	28	50
	704	37.66	37.32	35.03	88	6	VEST	REST3	28	50
	705	37.65	37.33	35.04	88	6	VEST	REST3	28	50
	706	37.66	37.33	35.03	89	6	VEST	REST3	28	50
	707	37.65	37.33	35.05	87	6	VEST	REST3	28	50
	708	37.66	37.33	35.07	89	6	VEST	REST3	28	50
	709	37.66	37.33	35.06	88	6	VEST	REST3	28	50
0	1	37.34	37.07	35.93	73	7	VEST	REST1	28	50
	2	37.34	37.04	35.91	73	7	VEST	REST1	28	50
	3	37.34	37.03	35.90	74	7	VEST	REST1	28	50
	4	37.34	37.05	35.91	72	7	VEST	REST1	28	50
	5	37.34	37.06	35.91	75	7	VEST	REST1	28	50
	6	37.33	37.05	35.91	75	7	VEST	REST1	28	50
	7	37.33	37.04	35.90	78	7	VEST	REST1	28	50
	8	37.33	37.02	35.90	75	7	VEST	REST1	28	50
	9	37.33	37.01	35.90	71	7	VEST	REST1	28	50
	10	37.33	37.01	35.90	71	7	VEST	REST1	28	50
	11	37.32	37.01	35.90	70	7	VEST	REST1	28	50
	12	37.32	37.00	35.89	77	7	VEST	REST1	28	50
	13	37.32	36.96	35.88	77	7	VEST	REST1	28	50
	14	37.32	36.92	35.88	77	7	VEST	REST1	28	50
	15	37.32	36.96	35.89	75	7	VEST	REST1	28	50
	16	37.32	36.99	35.90	74	7	VEST	REST1	28	50
	17	37.32	36.92	35.90	87	7	VEST	REST1	28	50
	18	37.33	36.92	35.90	90	7	VEST	REST1	28	50
	19	37.33	37.00	35.89	78	7	VEST	REST1	28	50
	20	37.34	37.01	35.89	77	7	VEST	REST1	28	50
	21	37.33	37.01	35.90	77	7	VEST	REST1	28	50
	22	37.32	37.00	35.89	82	7	VEST	REST1	28	50
	23	37.32	36.99	35.88	78	7	VEST	REST1	28	50
	24	37.31	37.00	35.89	78	7	VEST	REST1	28	50
	25	37.31	37.01	35.90	84	7	VEST	REST1	28	50
	26	37.31	37.01	35.90	80	7	VEST	REST1	28	50
	27	37.30	37.00	35.89	74	7	VEST	REST1	28	50
	28	37.30	37.00	35.88	75	7	VEST	REST1	28	50
	29	37.31	37.01	35.88	73	7	VEST	REST1	28	50
	30	37.32	37.02	35.88	73	7	VEST	REST1	28	50
5	31	37.32	37.02	35.88	74	7	VEST	REST1	28	50
	32	37.32	37.02	35.88	67	7	VEST	REST1	28	50
	33	37.32	37.04	35.88	69	7	VEST	REST1	28	50
	34	37.31	37.04	35.88	75	7	VEST	REST1	28	50
	35	37.31	37.04	35.89	72	7	VEST	REST1	28	50
	36	37.31	37.03	35.88	74	7	VEST	REST1	28	50
	37	37.30	37.02	35.87	70	7	VEST	REST1	28	50

min	number	Tre	Tes	Tsk-head	HR	ID	condition	period	Ta	RH
	38	37.31	37.03	35.88	74	7	VEST	REST1	28	50
	39	37.30	37.05	35.89	71	7	VEST	REST1	28	50
	40	37.29	37.04	35.89	74	7	VEST	REST1	28	50
	41	37.29	37.04	35.89	68	7	VEST	REST1	28	50
	42	37.29	37.05	35.90	68	7	VEST	REST1	28	50
	43	37.30	37.06	35.90	74	7	VEST	REST1	28	50
	44	37.29	37.06	35.91	71	7	VEST	REST1	28	50
	45	37.30	37.06	35.91	72	7	VEST	REST1	28	50
	46	37.30	37.05	35.90	68	7	VEST	REST1	28	50
	47	37.29	37.05	35.90	73	7	VEST	REST1	28	50
	48	37.29	37.01	35.90	88	7	VEST	REST1	28	50
	49	37.29	37.00	35.92	88	7	VEST	REST1	28	50
	50	37.28	37.03	35.93	78	7	VEST	REST1	28	50
	51	37.27	37.02	35.92	74	7	VEST	REST1	28	50
	52	37.27	37.01	35.92	72	7	VEST	REST1	28	50
	53	37.27	37.01	35.91	69	7	VEST	REST1	28	50
	54	37.27	37.02	35.91	72	7	VEST	REST1	28	50
	55	37.27	37.04	35.91	72	7	VEST	REST1	28	50
	56	37.27	37.04	35.91	70	7	VEST	REST1	28	50
	57	37.27	37.04	35.91	62	7	VEST	REST1	28	50
	58	37.28	37.04	35.91	63	7	VEST	REST1	28	50
	59	37.28	37.02	35.90	66	7	VEST	REST1	28	50
	60	37.27	37.02	35.90	67	7	VEST	REST1	28	50
10	61	37.27	37.04	35.90	63	7	VEST	REST1	28	50
	62	37.28	37.03	35.89	68	7	VEST	REST1	28	50
	63	37.28	37.03	35.89	66	7	VEST	REST1	28	50
	64	37.28	37.04	35.88	68	7	VEST	REST1	28	50
	65	37.28	37.03	35.88	62	7	VEST	REST1	28	50
	66	37.28	37.05	35.87	71	7	VEST	REST1	28	50
	67	37.27	37.05	35.86	68	7	VEST	REST1	28	50
	68	37.27	37.04	35.87	70	7	VEST	REST1	28	50
	69	37.27	37.06	35.89	61	7	VEST	REST1	28	50
	70	37.28	37.06	35.89	62	7	VEST	REST1	28	50
	71	37.28	37.04	35.89	64	7	VEST	REST1	28	50
	72	37.28	37.06	35.89	64	7	VEST	REST1	28	50
	73	37.28	37.06	35.89	68	7	VEST	REST1	28	50
	74	37.29	37.06	35.88	64	7	VEST	REST1	28	50
	75	37.28	37.07	35.88	65	7	VEST	REST1	28	50
	76	37.28	37.06	35.88	60	7	VEST	REST1	28	50
	77	37.28	37.06	35.87	63	7	VEST	REST1	28	50
	78	37.28	37.06	35.87	57	7	VEST	REST1	28	50
	79	37.29	37.09	35.87	68	7	VEST	REST1	28	50
	80	37.29	37.07	35.85	61	7	VEST	REST1	28	50
	81	37.29	37.07	35.86	65	7	VEST	REST1	28	50
	82	37.29	37.07	35.87	62	7	VEST	REST1	28	50
	83	37.29	37.06	35.87	69	7	VEST	REST1	28	50

min	number	Tre	Tes	Tsk-head	HR	ID	condition	period	Ta	RH
	84	37.29	37.08	35.88	60	7	VEST	REST1	28	50
	85	37.29	37.08	35.87	69	7	VEST	REST1	28	50
	86	37.28	37.07	35.85	75	7	VEST	REST1	28	50
	87	37.28	37.06	35.84	81	7	VEST	REST1	28	50
	88	37.28	37.05	35.84	90	7	VEST	REST1	28	50
	89	37.28	37.06	35.85	89	7	VEST	REST1	28	50
	90	37.28	37.05	35.86	85	7	VEST	REST1	28	50
15	91	37.28	37.03	35.87	75	7	VEST	REST1	28	50
	92	37.29	37.03	35.88	77	7	VEST	REST1	28	50
	93	37.29	37.02	35.88	86	7	VEST	REST1	28	50
	94	37.29	37.02	35.89	77	7	VEST	REST1	28	50
	95	37.29	37.01	35.89	76	7	VEST	REST1	28	50
	96	37.28	37.01	35.89	77	7	VEST	REST1	28	50
	97	37.28	37.01	35.91	69	7	VEST	REST1	28	50
	98	37.28	37.00	35.90	82	7	VEST	REST1	28	50
	99	37.28	37.01	35.91	80	7	VEST	REST1	28	50
	100	37.28	37.00	35.92	83	7	VEST	REST1	28	50
	101	37.28	37.00	35.91	70	7	VEST	REST1	28	50
	102	37.28	37.01	35.91	72	7	VEST	REST1	28	50
	103	37.29	37.01	35.90	83	7	VEST	REST1	40	50
	104	37.28	37.00	35.96	99	7	VEST	REST1	40	50
	105	37.25	36.94	36.10	90	7	VEST	REST1	40	50
	106	37.24	36.89	36.22	69	7	VEST	REST1	40	50
	107	37.24	36.87	36.29	76	7	VEST	REST1	40	50
	108	37.25	36.85	36.34	82	7	VEST	REST1	40	50
	109	37.26	36.85	36.38	76	7	VEST	REST1	40	50
	110	37.26	36.82	36.41	73	7	VEST	REST1	40	50
	111	37.26	36.82	36.45	77	7	VEST	REST1	40	50
	112	37.25	36.82	36.50	92	7	VEST	REST1	40	50
	113	37.23	36.82	36.54	83	7	VEST	REST1	40	50
	114	37.20	36.88	36.55	86	7	VEST	REST1	40	50
	115	37.19	36.94	36.57	81	7	VEST	REST1	40	50
	116	37.18	36.88	36.60	79	7	VEST	REST1	40	50
	117	37.19	36.84	36.62	81	7	VEST	REST1	40	50
	118	37.21	36.84	36.63	79	7	VEST	REST1	40	50
	119	37.23	36.86	36.65	74	7	VEST	REST1	40	50
	120	37.24	36.86	36.66	72	7	VEST	REST1	40	50
20	121	37.24	36.86	36.68	73	7	VEST	REST1	40	50
	122	37.25	36.93	36.72	76	7	VEST	REST1	40	50
	123	37.25	36.93	36.73	70	7	VEST	REST1	40	50
	124	37.24	36.88	36.74	72	7	VEST	REST1	40	50
	125	37.25	36.89	36.75	70	7	VEST	REST1	40	50
	126	37.25	36.89	36.77	76	7	VEST	REST1	40	50
	127	37.25	36.90	36.78	74	7	VEST	REST1	40	50
	128	37.25	36.92	36.81	79	7	VEST	REST1	40	50
	129	37.25	36.94	36.84	86	7	VEST	REST1	40	50

min	number	Tre	Tes	Tsk-head	HR	ID	condition	period	Ta	RH
	130	37.25	36.95	36.85	79	7	VEST	REST1	40	50
	131	37.25	36.96	36.87	76	7	VEST	REST1	40	50
	132	37.25	36.96	36.88	71	7	VEST	REST1	40	50
	133	37.25	36.95	36.90	77	7	VEST	REST1	40	50
	134	37.24	36.95	36.92	105	7	VEST	REST1	40	50
	135	37.21	36.94	36.93	81	7	VEST	REST1	40	50
	136	37.19	36.93	36.94	77	7	VEST	REST1	40	50
	137	37.18	36.92	36.95	87	7	VEST	REST1	40	50
	138	37.19	36.93	36.97	79	7	VEST	REST1	40	50
	139	37.21	36.94	36.98	80	7	VEST	EXERCISE1	40	50
	140	37.23	36.93	36.99	91	7	VEST	EXERCISE1	40	50
	141	37.23	36.89	37.00	87	7	VEST	EXERCISE1	40	50
	142	37.23	36.86	37.02	98	7	VEST	EXERCISE1	40	50
	143	37.24	36.86	37.03	102	7	VEST	EXERCISE1	40	50
	144	37.24	36.86	37.04	101	7	VEST	EXERCISE1	40	50
	145	37.24	36.86	37.06	105	7	VEST	EXERCISE1	40	50
	146	37.24	36.85	37.07	106	7	VEST	EXERCISE1	40	50
	147	37.25	36.84	37.07	102	7	VEST	EXERCISE1	40	50
	148	37.25	36.85	37.09	102	7	VEST	EXERCISE1	40	50
	149	37.25	36.87	37.10	107	7	VEST	EXERCISE1	40	50
	150	37.25	36.87	37.10	104	7	VEST	EXERCISE1	40	50
25	151	37.25	36.87	37.10	105	7	VEST	EXERCISE1	40	50
	152	37.25	36.87	37.11	105	7	VEST	EXERCISE1	40	50
	153	37.25	36.89	37.12	103	7	VEST	EXERCISE1	40	50
	154	37.25	36.83	37.13	104	7	VEST	EXERCISE1	40	50
	155	37.25	36.81	37.13	104	7	VEST	EXERCISE1	40	50
	156	37.25	36.86	37.15	108	7	VEST	EXERCISE1	40	50
	157	37.25	36.88	37.15	104	7	VEST	EXERCISE1	40	50
	158	37.25	36.88	37.16	105	7	VEST	EXERCISE1	40	50
	159	37.25	36.78	37.17	109	7	VEST	EXERCISE1	40	50
	160	37.25	36.78	37.18	106	7	VEST	EXERCISE1	40	50
	161	37.25	36.91	37.20	99	7	VEST	EXERCISE1	40	50
	162	37.25	36.93	37.22	105	7	VEST	EXERCISE1	40	50
	163	37.25	36.95	37.22	106	7	VEST	EXERCISE1	40	50
	164	37.25	36.96	37.22	106	7	VEST	EXERCISE1	40	50
	165	37.26	36.97	37.23	107	7	VEST	EXERCISE1	40	50
	166	37.26	36.99	37.23	108	7	VEST	EXERCISE1	40	50
	167	37.27	37.00	37.24	110	7	VEST	EXERCISE1	40	50
	168	37.26	37.02	37.25	107	7	VEST	EXERCISE1	40	50
	169	37.26	37.03	37.25	108	7	VEST	EXERCISE1	40	50
	170	37.27	37.04	37.26	105	7	VEST	EXERCISE1	40	50
	171	37.27	37.06	37.27	107	7	VEST	EXERCISE1	40	50
	172	37.26	37.06	37.27	107	7	VEST	EXERCISE1	40	50
	173	37.27	37.00	37.29	108	7	VEST	EXERCISE1	40	50
	174	37.27	36.98	37.30	108	7	VEST	EXERCISE1	40	50
	175	37.27	37.02	37.30	108	7	VEST	EXERCISE1	40	50

min	number	Tre	Tes	Tsk-head	HR	ID	condition	period	Ta	RH
	176	37.27	37.05	37.31	107	7	VEST	EXERCISE1	40	50
	177	37.27	37.09	37.33	108	7	VEST	EXERCISE1	40	50
	178	37.28	37.10	37.34	109	7	VEST	EXERCISE1	40	50
	179	37.28	37.11	37.35	110	7	VEST	EXERCISE1	40	50
	180	37.28	37.12	37.35	111	7	VEST	EXERCISE1	40	50
30	181	37.28	37.13	37.35	110	7	VEST	EXERCISE1	40	50
	182	37.28	37.14	37.35	109	7	VEST	EXERCISE1	40	50
	183	37.28	37.16	37.36	110	7	VEST	EXERCISE1	40	50
	184	37.28	37.17	37.36	111	7	VEST	EXERCISE1	40	50
	185	37.28	37.18	37.35	111	7	VEST	EXERCISE1	40	50
	186	37.28	37.19	37.36	112	7	VEST	EXERCISE1	40	50
	187	37.29	37.19	37.36	112	7	VEST	EXERCISE1	40	50
	188	37.29	37.21	37.37	110	7	VEST	EXERCISE1	40	50
	189	37.29	37.21	37.37	112	7	VEST	EXERCISE1	40	50
	190	37.29	37.20	37.36	111	7	VEST	EXERCISE1	40	50
	191	37.29	37.22	37.37	109	7	VEST	EXERCISE1	40	50
	192	37.29	37.23	37.38	112	7	VEST	EXERCISE1	40	50
	193	37.30	37.24	37.38	114	7	VEST	EXERCISE1	40	50
	194	37.30	37.25	37.37	111	7	VEST	EXERCISE1	40	50
	195	37.31	37.24	37.37	111	7	VEST	EXERCISE1	40	50
	196	37.32	37.25	37.37	111	7	VEST	EXERCISE1	40	50
	197	37.31	37.26	37.37	109	7	VEST	EXERCISE1	40	50
	198	37.32	37.15	37.36	114	7	VEST	EXERCISE1	40	50
	199	37.33	37.07	37.36	112	7	VEST	EXERCISE1	40	50
	200	37.33	37.13	37.37	115	7	VEST	EXERCISE1	40	50
	201	37.34	37.18	37.37	112	7	VEST	EXERCISE1	40	50
	202	37.34	37.21	37.37	112	7	VEST	EXERCISE1	40	50
	203	37.34	37.24	37.36	112	7	VEST	EXERCISE1	40	50
	204	37.34	37.27	37.36	111	7	VEST	EXERCISE1	40	50
	205	37.34	37.27	37.36	111	7	VEST	EXERCISE1	40	50
	206	37.35	37.27	37.36	110	7	VEST	EXERCISE1	40	50
	207	37.35	37.28	37.37	112	7	VEST	EXERCISE1	40	50
	208	37.34	37.28	37.37	112	7	VEST	EXERCISE1	40	50
	209	37.34	37.29	37.37	110	7	VEST	EXERCISE1	40	50
	210	37.34	37.30	37.36	112	7	VEST	EXERCISE1	40	50
35	211	37.34	37.30	37.36	115	7	VEST	EXERCISE1	40	50
	212	37.35	37.31	37.37	113	7	VEST	EXERCISE1	40	50
	213	37.35	37.31	37.36	111	7	VEST	EXERCISE1	40	50
	214	37.35	37.31	37.36	112	7	VEST	EXERCISE1	40	50
	215	37.35	37.31	37.36	111	7	VEST	EXERCISE1	40	50
	216	37.35	37.31	37.36	110	7	VEST	EXERCISE1	40	50
	217	37.35	37.32	37.36	109	7	VEST	EXERCISE1	40	50
	218	37.36	37.32	37.36	111	7	VEST	EXERCISE1	40	50
	219	37.36	37.32	37.35	109	7	VEST	EXERCISE1	40	50
	220	37.36	37.33	37.35	112	7	VEST	EXERCISE1	40	50
	221	37.37	37.33	37.35	111	7	VEST	EXERCISE1	40	50

min	number	Tre	Tes	Tsk-head	HR	ID	condition	period	Ta	RH
	222	37.38	37.35	37.36	113	7	VEST	EXERCISE1	40	50
	223	37.38	37.36	37.36	113	7	VEST	EXERCISE1	40	50
	224	37.39	37.36	37.37	112	7	VEST	EXERCISE1	40	50
	225	37.39	37.36	37.36	111	7	VEST	EXERCISE1	40	50
	226	37.39	37.36	37.36	112	7	VEST	EXERCISE1	40	50
	227	37.39	37.36	37.37	116	7	VEST	EXERCISE1	40	50
	228	37.39	37.36	37.36	110	7	VEST	EXERCISE1	40	50
	229	37.40	37.36	37.36	110	7	VEST	EXERCISE1	40	50
	230	37.40	37.36	37.37	111	7	VEST	EXERCISE1	40	50
	231	37.41	37.35	37.36	111	7	VEST	EXERCISE1	40	50
	232	37.41	37.35	37.35	116	7	VEST	EXERCISE1	40	50
	233	37.41	37.36	37.36	113	7	VEST	EXERCISE1	40	50
	234	37.41	37.36	37.36	112	7	VEST	EXERCISE1	40	50
	235	37.41	37.36	37.36	112	7	VEST	EXERCISE1	40	50
	236	37.41	37.37	37.36	113	7	VEST	EXERCISE1	40	50
	237	37.42	37.37	37.37	112	7	VEST	EXERCISE1	40	50
	238	37.42	37.38	37.38	112	7	VEST	EXERCISE1	40	50
	239	37.43	37.39	37.37	110	7	VEST	EXERCISE1	40	50
	240	37.43	37.38	37.37	112	7	VEST	EXERCISE1	40	50
40	241	37.43	37.38	37.38	117	7	VEST	EXERCISE1	40	50
	242	37.43	37.39	37.38	115	7	VEST	EXERCISE1	40	50
	243	37.44	37.43	37.37	109	7	VEST	EXERCISE1	40	50
	244	37.44	37.43	37.36	113	7	VEST	EXERCISE1	40	50
	245	37.44	37.33	37.36	113	7	VEST	EXERCISE1	40	50
	246	37.43	37.29	37.37	110	7	VEST	EXERCISE1	40	50
	247	37.44	37.34	37.37	108	7	VEST	EXERCISE1	40	50
	248	37.45	37.37	37.38	111	7	VEST	EXERCISE1	40	50
	249	37.44	37.38	37.37	113	7	VEST	EXERCISE1	40	50
	250	37.45	37.39	37.37	111	7	VEST	EXERCISE1	40	50
	251	37.45	37.40	37.38	111	7	VEST	EXERCISE1	40	50
	252	37.45	37.41	37.39	111	7	VEST	EXERCISE1	40	50
	253	37.45	37.41	37.39	112	7	VEST	EXERCISE1	40	50
	254	37.45	37.42	37.40	111	7	VEST	EXERCISE1	40	50
	255	37.45	37.42	37.41	114	7	VEST	EXERCISE1	40	50
	256	37.45	37.41	37.40	109	7	VEST	EXERCISE1	40	50
	257	37.46	37.41	37.40	116	7	VEST	EXERCISE1	40	50
	258	37.46	37.41	37.41	112	7	VEST	EXERCISE1	40	50
	259	37.47	37.41	37.41	111	7	VEST	EXERCISE1	40	50
	260	37.48	37.42	37.42	109	7	VEST	EXERCISE1	40	50
	261	37.48	37.42	37.43	110	7	VEST	EXERCISE1	40	50
	262	37.48	37.42	37.43	110	7	VEST	EXERCISE1	40	50
	263	37.48	37.41	37.43	111	7	VEST	EXERCISE1	40	50
	264	37.48	37.40	37.42	113	7	VEST	EXERCISE1	40	50
	265	37.49	37.40	37.42	111	7	VEST	EXERCISE1	40	50
	266	37.50	37.41	37.43	111	7	VEST	EXERCISE1	40	50
	267	37.49	37.40	37.42	112	7	VEST	EXERCISE1	40	50

min	number	Tre	Tes	Tsk-head	HR	ID	condition	period	Ta	RH
	268	37.49	37.40	37.41	110	7	VEST	EXERCISE1	40	50
	269	37.51	37.41	37.42	110	7	VEST	EXERCISE1	40	50
	270	37.51	37.42	37.43	113	7	VEST	EXERCISE1	40	50
45	271	37.51	37.43	37.43	114	7	VEST	EXERCISE1	40	50
	272	37.51	37.43	37.43	114	7	VEST	EXERCISE1	40	50
	273	37.52	37.43	37.44	113	7	VEST	EXERCISE1	40	50
	274	37.51	37.43	37.44	115	7	VEST	EXERCISE1	40	50
	275	37.52	37.44	37.44	114	7	VEST	EXERCISE1	40	50
	276	37.53	37.45	37.45	114	7	VEST	EXERCISE1	40	50
	277	37.54	37.45	37.45	115	7	VEST	EXERCISE1	40	50
	278	37.53	37.44	37.45	114	7	VEST	EXERCISE1	40	50
	279	37.54	37.39	37.45	113	7	VEST	EXERCISE1	40	50
	280	37.55	37.36	37.45	113	7	VEST	EXERCISE1	40	50
	281	37.54	37.40	37.45	114	7	VEST	EXERCISE1	40	50
	282	37.54	37.41	37.45	114	7	VEST	EXERCISE1	40	50
	283	37.54	37.42	37.45	114	7	VEST	EXERCISE1	40	50
	284	37.54	37.43	37.45	113	7	VEST	EXERCISE1	40	50
	285	37.55	37.44	37.45	118	7	VEST	EXERCISE1	40	50
	286	37.55	37.45	37.46	113	7	VEST	EXERCISE1	40	50
	287	37.56	37.45	37.47	117	7	VEST	EXERCISE1	40	50
	288	37.56	37.46	37.46	117	7	VEST	EXERCISE1	40	50
	289	37.57	37.47	37.46	116	7	VEST	EXERCISE1	40	50
	290	37.57	37.48	37.46	113	7	VEST	EXERCISE1	40	50
	291	37.57	37.48	37.45	112	7	VEST	EXERCISE1	40	50
	292	37.57	37.47	37.44	113	7	VEST	EXERCISE1	40	50
	293	37.57	37.47	37.44	113	7	VEST	EXERCISE1	40	50
	294	37.58	37.47	37.45	118	7	VEST	EXERCISE1	40	50
	295	37.58	37.39	37.45	118	7	VEST	EXERCISE1	40	50
	296	37.58	37.33	37.45	114	7	VEST	EXERCISE1	40	50
	297	37.58	37.37	37.45	112	7	VEST	EXERCISE1	40	50
	298	37.59	37.42	37.46	113	7	VEST	EXERCISE1	40	50
	299	37.60	37.45	37.48	116	7	VEST	EXERCISE1	40	50
	300	37.60	37.45	37.48	117	7	VEST	EXERCISE1	40	50
50	301	37.60	37.45	37.48	114	7	VEST	EXERCISE1	40	50
	302	37.60	37.46	37.48	114	7	VEST	EXERCISE1	40	50
	303	37.60	37.46	37.49	114	7	VEST	EXERCISE1	40	50
	304	37.61	37.47	37.50	113	7	VEST	EXERCISE1	40	50
	305	37.61	37.47	37.50	113	7	VEST	EXERCISE1	40	50
	306	37.61	37.47	37.50	114	7	VEST	EXERCISE1	40	50
	307	37.62	37.47	37.50	115	7	VEST	EXERCISE1	40	50
	308	37.63	37.47	37.50	117	7	VEST	EXERCISE1	40	50
	309	37.63	37.47	37.50	118	7	VEST	EXERCISE1	40	50
	310	37.64	37.48	37.49	116	7	VEST	EXERCISE1	40	50
	311	37.64	37.48	37.49	115	7	VEST	EXERCISE1	40	50
	312	37.63	37.47	37.50	116	7	VEST	EXERCISE1	40	50
	313	37.63	37.48	37.49	113	7	VEST	EXERCISE1	40	50

min	number	Tre	Tes	Tsk-head	HR	ID	condition	period	Ta	RH
	314	37.64	37.46	37.49	116	7	VEST	EXERCISE1	40	50
	315	37.65	37.39	37.50	117	7	VEST	EXERCISE1	40	50
	316	37.65	37.36	37.50	114	7	VEST	EXERCISE1	40	50
	317	37.66	37.38	37.51	114	7	VEST	EXERCISE1	40	50
	318	37.66	37.41	37.50	114	7	VEST	EXERCISE1	40	50
	319	37.66	37.43	37.49	111	7	VEST	EXERCISE1	40	50
	320	37.66	37.42	37.50	117	7	VEST	REST2	28	50
	321	37.65	37.43	37.49	121	7	VEST	REST2	28	50
	322	37.65	37.35	37.39	135	7	VEST	REST2	28	50
	323	37.64	37.32	37.27	106	7	VEST	REST2	28	50
	324	37.63	37.42	37.23	105	7	VEST	REST2	28	50
	325	37.62	37.47	37.20	101	7	VEST	REST2	28	50
	326	37.63	37.50	37.13	107	7	VEST	REST2	28	50
	327	37.64	37.52	37.01	103	7	VEST	REST2	28	50
	328	37.63	37.51	36.94	100	7	VEST	REST2	28	50
	329	37.63	37.50	36.94	96	7	VEST	REST2	28	50
	330	37.64	37.49	36.93	93	7	VEST	REST2	28	50
55	331	37.65	37.47	36.90	95	7	VEST	REST2	28	50
	332	37.65	37.45	36.88	99	7	VEST	REST2	28	50
	333	37.66	37.44	36.85	84	7	VEST	REST2	28	50
	334	37.65	37.43	36.82	96	7	VEST	REST2	28	50
	335	37.65	37.42	36.79	89	7	VEST	REST2	28	50
	336	37.66	37.38	36.77	86	7	VEST	REST2	28	50
	337	37.66	37.35	36.74	88	7	VEST	REST2	28	50
	338	37.67	33.06	36.70	96	7	VEST	REST2	28	50
	339	37.66	29.23	36.69	104	7	VEST	REST2	28	50
	340	37.66	29.45	36.68	104	7	VEST	REST2	28	50
	341	37.66	29.32	36.68	99	7	VEST	REST2	28	50
	342	37.67	30.83	36.65	94	7	VEST	REST2	28	50
	343	37.68	32.50	36.61	100	7	VEST	REST2	28	50
	344	37.67	33.21	36.59	93	7	VEST	REST2	28	50
	345	37.67	33.75	36.57	92	7	VEST	REST2	28	50
	346	37.67	30.74	36.55	108	7	VEST	REST2	28	50
	347	37.67	29.35	36.52	87	7	VEST	REST2	28	50
	348	37.68	29.29	36.51	91	7	VEST	REST2	28	50
	349	37.68	28.36	36.49	93	7	VEST	REST2	28	50
	350	37.68	28.63	36.46	91	7	VEST	REST2	28	50
	351	37.69	30.09	36.45	88	7	VEST	REST2	28	50
	352	37.70	29.49	36.44	94	7	VEST	REST2	28	50
	353	37.69	28.75	36.42	80	7	VEST	REST2	28	50
	354	37.68	29.70	36.38	95	7	VEST	REST2	28	50
	355	37.67	30.27	36.35	86	7	VEST	REST2	28	50
	356	37.67	30.04	36.34	91	7	VEST	REST2	28	50
	357	37.67	28.37	36.34	85	7	VEST	REST2	28	50
	358	37.67	29.73	36.32	85	7	VEST	REST2	28	50
	359	37.68	31.98	36.30	85	7	VEST	REST2	28	50

min	number	Tre	Tes	Tsk-head	HR	ID	condition	period	Ta	RH
	360	37.68	33.00	36.28	84	7	VEST	REST2	28	50
60	361	37.67	33.38	36.27	82	7	VEST	REST2	28	50
	362	37.67	33.29	36.23	76	7	VEST	REST2	28	50
	363	37.67	33.59	36.19	78	7	VEST	REST2	28	50
	364	37.67	34.75	36.14	79	7	VEST	REST2	28	50
	365	37.68	35.38	36.10	84	7	VEST	REST2	28	50
	366	37.68	35.67	36.07	82	7	VEST	REST2	28	50
	367	37.67	35.77	36.05	84	7	VEST	REST2	28	50
	368	37.66	35.99	36.03	80	7	VEST	REST2	28	50
	369	37.67	36.30	36.00	81	7	VEST	REST2	28	50
	370	37.67	36.47	35.97	82	7	VEST	REST2	28	50
	371	37.66	36.47	35.94	85	7	VEST	REST2	28	50
	372	37.65	36.51	35.94	89	7	VEST	REST2	28	50
	373	37.65	36.66	35.95	82	7	VEST	REST2	28	50
	374	37.64	36.74	35.92	78	7	VEST	REST2	28	50
	375	37.64	36.83	35.90	80	7	VEST	REST2	28	50
	376	37.64	36.89	35.90	80	7	VEST	REST2	28	50
	377	37.64	36.93	35.88	82	7	VEST	REST2	28	50
	378	37.64	36.97	35.86	81	7	VEST	REST2	28	50
	379	37.64	37.00	35.85	80	7	VEST	REST2	28	50
	380	37.64	37.03	35.83	80	7	VEST	REST2	28	50
	381	37.63	36.87	35.81	78	7	VEST	REST2	28	50
	382	37.63	35.65	35.80	74	7	VEST	REST2	28	50
	383	37.63	35.21	35.80	91	7	VEST	REST2	28	50
	384	37.63	36.09	35.81	80	7	VEST	REST2	28	50
	385	37.62	36.50	35.82	79	7	VEST	REST2	28	50
	386	37.61	36.70	35.82	76	7	VEST	REST2	28	50
	387	37.62	36.82	35.80	80	7	VEST	REST2	28	50
	388	37.62	36.89	35.78	79	7	VEST	REST2	28	50
	389	37.62	36.93	35.78	76	7	VEST	REST2	28	50
	390	37.62	36.97	35.78	72	7	VEST	REST2	28	50
65	391	37.62	37.01	35.77	72	7	VEST	REST2	28	50
	392	37.62	37.04	35.76	73	7	VEST	REST2	28	50
	393	37.62	37.07	35.76	73	7	VEST	REST2	28	50
	394	37.62	37.08	35.76	73	7	VEST	REST2	28	50
	395	37.61	37.08	35.77	75	7	VEST	REST2	28	50
	396	37.60	37.10	35.76	75	7	VEST	REST2	28	50
	397	37.61	37.11	35.75	74	7	VEST	REST2	28	50
	398	37.61	37.10	35.74	74	7	VEST	REST2	28	50
	399	37.62	37.10	35.73	75	7	VEST	REST2	28	50
	400	37.62	37.12	35.72	77	7	VEST	REST2	28	50
	401	37.61	37.13	35.72	78	7	VEST	REST2	28	50
	402	37.62	37.14	35.74	76	7	VEST	REST2	28	50
	403	37.62	37.14	35.73	75	7	VEST	REST2	28	50
	404	37.61	37.07	35.72	74	7	VEST	REST2	28	50
	405	37.61	37.04	35.71	74	7	VEST	REST2	28	50

min	number	Tre	Tes	Tsk-head	HR	ID	condition	period	Ta	RH
	406	37.61	37.10	35.72	76	7	VEST	REST2	28	50
	407	37.61	37.12	35.73	78	7	VEST	REST2	28	50
	408	37.60	37.01	35.75	90	7	VEST	REST2	28	50
	409	37.61	36.99	35.80	89	7	VEST	REST2	28	50
	410	37.61	37.08	35.83	92	7	VEST	REST2	28	50
	411	37.61	37.09	35.85	94	7	VEST	REST2	28	50
	412	37.61	37.11	35.88	90	7	VEST	REST2	28	50
	413	37.60	37.13	35.90	87	7	VEST	REST2	28	50
	414	37.59	37.13	35.92	83	7	VEST	REST2	28	50
	415	37.59	37.13	35.94	84	7	VEST	REST2	28	50
	416	37.59	37.13	35.93	86	7	VEST	REST2	28	50
	417	37.59	37.15	35.89	82	7	VEST	REST2	28	50
	418	37.59	37.15	35.88	84	7	VEST	REST2	28	50
	419	37.60	37.16	35.92	88	7	VEST	REST2	28	50
	420	37.59	37.15	35.95	87	7	VEST	REST2	28	50
70	421	37.59	37.15	35.96	90	7	VEST	REST2	28	50
	422	37.60	37.14	35.97	86	7	VEST	REST2	28	50
	423	37.60	37.12	35.98	79	7	VEST	REST2	28	50
	424	37.60	37.14	35.99	84	7	VEST	REST2	28	50
	425	37.60	37.15	36.00	84	7	VEST	REST2	28	50
	426	37.60	37.15	36.02	79	7	VEST	REST2	28	50
	427	37.60	37.17	36.03	80	7	VEST	REST2	28	50
	428	37.59	37.18	36.03	83	7	VEST	REST2	28	50
	429	37.59	37.17	36.04	90	7	VEST	REST2	28	50
	430	37.59	37.18	36.05	89	7	VEST	REST2	28	50
	431	37.58	37.18	36.06	84	7	VEST	REST2	28	50
	432	37.58	37.17	36.07	79	7	VEST	REST2	28	50
	433	37.58	37.18	36.06	79	7	VEST	REST2	28	50
	434	37.58	37.19	36.06	81	7	VEST	REST2	28	50
	435	37.59	37.13	36.06	93	7	VEST	REST2	28	50
	436	37.60	37.10	36.06	98	7	VEST	REST2	28	50
	437	37.61	37.14	36.06	85	7	VEST	REST2	28	50
	438	37.62	37.11	36.06	72	7	VEST	REST2	28	50
	439	37.63	37.08	36.07	84	7	VEST	REST2	40	50
	440	37.64	37.01	36.15	104	7	VEST	REST2	40	50
	441	37.64	36.99	36.27	85	7	VEST	REST2	40	50
	442	37.63	37.07	36.36	80	7	VEST	REST2	40	50
	443	37.62	37.07	36.42	83	7	VEST	REST2	40	50
	444	37.62	37.10	36.48	79	7	VEST	REST2	40	50
	445	37.61	37.11	36.53	86	7	VEST	REST2	40	50
	446	37.61	37.11	36.57	78	7	VEST	REST2	40	50
	447	37.61	37.06	36.61	86	7	VEST	REST2	40	50
	448	37.61	37.04	36.65	81	7	VEST	REST2	40	50
	449	37.60	37.09	36.68	78	7	VEST	REST2	40	50
	450	37.61	37.11	36.71	71	7	VEST	REST2	40	50
75	451	37.62	37.12	36.74	70	7	VEST	REST2	40	50

min	number	Tre	Tes	Tsk-head	HR	ID	condition	period	Ta	RH
	452	37.61	37.11	36.76	81	7	VEST	REST2	40	50
	453	37.61	37.10	36.79	72	7	VEST	REST2	40	50
	454	37.62	37.11	36.82	76	7	VEST	REST2	40	50
	455	37.61	37.11	36.85	76	7	VEST	REST2	40	50
	456	37.61	37.09	36.87	83	7	VEST	REST2	40	50
	457	37.60	37.08	36.89	78	7	VEST	REST2	40	50
	458	37.60	37.10	36.90	103	7	VEST	REST2	40	50
	459	37.61	37.10	36.90	89	7	VEST	REST2	40	50
	460	37.61	37.10	36.91	88	7	VEST	REST2	40	50
	461	37.60	37.11	36.93	85	7	VEST	REST2	40	50
	462	37.61	37.12	36.96	89	7	VEST	REST2	40	50
	463	37.61	37.12	36.97	83	7	VEST	EXERCISE2	40	50
	464	37.61	37.10	36.98	90	7	VEST	EXERCISE2	40	50
	465	37.62	37.12	37.02	94	7	VEST	EXERCISE2	40	50
	466	37.62	37.14	37.04	104	7	VEST	EXERCISE2	40	50
	467	37.62	37.12	37.04	97	7	VEST	EXERCISE2	40	50
	468	37.62	37.12	37.05	106	7	VEST	EXERCISE2	40	50
	469	37.62	37.14	37.07	109	7	VEST	EXERCISE2	40	50
	470	37.62	37.09	37.08	101	7	VEST	EXERCISE2	40	50
	471	37.62	37.01	37.09	108	7	VEST	EXERCISE2	40	50
	472	37.62	37.05	37.11	109	7	VEST	EXERCISE2	40	50
	473	37.62	37.13	37.12	110	7	VEST	EXERCISE2	40	50
	474	37.63	37.15	37.13	113	7	VEST	EXERCISE2	40	50
	475	37.62	37.18	37.14	114	7	VEST	EXERCISE2	40	50
	476	37.62	37.20	37.14	113	7	VEST	EXERCISE2	40	50
	477	37.62	37.22	37.15	113	7	VEST	EXERCISE2	40	50
	478	37.63	37.24	37.16	112	7	VEST	EXERCISE2	40	50
	479	37.63	37.26	37.16	113	7	VEST	EXERCISE2	40	50
	480	37.62	37.28	37.17	112	7	VEST	EXERCISE2	40	50
80	481	37.63	37.29	37.17	111	7	VEST	EXERCISE2	40	50
	482	37.63	37.31	37.18	112	7	VEST	EXERCISE2	40	50
	483	37.63	37.31	37.18	113	7	VEST	EXERCISE2	40	50
	484	37.62	37.32	37.18	108	7	VEST	EXERCISE2	40	50
	485	37.62	37.33	37.18	114	7	VEST	EXERCISE2	40	50
	486	37.62	37.33	37.18	112	7	VEST	EXERCISE2	40	50
	487	37.62	37.35	37.19	112	7	VEST	EXERCISE2	40	50
	488	37.63	37.36	37.20	113	7	VEST	EXERCISE2	40	50
	489	37.63	37.28	37.20	118	7	VEST	EXERCISE2	40	50
	490	37.64	37.23	37.20	116	7	VEST	EXERCISE2	40	50
	491	37.64	37.29	37.20	114	7	VEST	EXERCISE2	40	50
	492	37.64	37.32	37.21	114	7	VEST	EXERCISE2	40	50
	493	37.65	37.34	37.22	112	7	VEST	EXERCISE2	40	50
	494	37.65	37.36	37.23	114	7	VEST	EXERCISE2	40	50
	495	37.65	37.36	37.24	114	7	VEST	EXERCISE2	40	50
	496	37.65	37.37	37.24	113	7	VEST	EXERCISE2	40	50
	497	37.64	37.37	37.25	115	7	VEST	EXERCISE2	40	50

min	number	Tre	Tes	Tsk-head	HR	ID	condition	period	Ta	RH
	498	37.64	37.38	37.25	115	7	VEST	EXERCISE2	40	50
	499	37.64	37.39	37.25	117	7	VEST	EXERCISE2	40	50
	500	37.64	37.49	37.26	116	7	VEST	EXERCISE2	40	50
	501	37.64	37.57	37.27	111	7	VEST	EXERCISE2	40	50
	502	37.64	37.54	37.27	115	7	VEST	EXERCISE2	40	50
	503	37.64	37.51	37.27	116	7	VEST	EXERCISE2	40	50
	504	37.65	37.47	37.28	117	7	VEST	EXERCISE2	40	50
	505	37.65	37.45	37.28	122	7	VEST	EXERCISE2	40	50
	506	37.64	37.45	37.29	118	7	VEST	EXERCISE2	40	50
	507	37.64	37.47	37.30	117	7	VEST	EXERCISE2	40	50
	508	37.64	37.47	37.31	116	7	VEST	EXERCISE2	40	50
	509	37.64	37.45	37.30	115	7	VEST	EXERCISE2	40	50
	510	37.65	37.45	37.30	115	7	VEST	EXERCISE2	40	50
85	511	37.65	37.44	37.31	115	7	VEST	EXERCISE2	40	50
	512	37.65	37.42	37.31	117	7	VEST	EXERCISE2	40	50
	513	37.65	37.42	37.31	117	7	VEST	EXERCISE2	40	50
	514	37.66	37.42	37.32	117	7	VEST	EXERCISE2	40	50
	515	37.67	37.42	37.33	117	7	VEST	EXERCISE2	40	50
	516	37.67	37.42	37.34	116	7	VEST	EXERCISE2	40	50
	517	37.67	37.43	37.34	115	7	VEST	EXERCISE2	40	50
	518	37.67	37.34	37.36	118	7	VEST	EXERCISE2	40	50
	519	37.68	37.30	37.36	119	7	VEST	EXERCISE2	40	50
	520	37.67	37.35	37.36	116	7	VEST	EXERCISE2	40	50
	521	37.67	37.38	37.37	117	7	VEST	EXERCISE2	40	50
	522	37.68	37.41	37.38	117	7	VEST	EXERCISE2	40	50
	523	37.67	37.42	37.38	116	7	VEST	EXERCISE2	40	50
	524	37.68	37.42	37.38	118	7	VEST	EXERCISE2	40	50
	525	37.69	37.42	37.38	121	7	VEST	EXERCISE2	40	50
	526	37.69	37.42	37.39	120	7	VEST	EXERCISE2	40	50
	527	37.68	37.42	37.38	118	7	VEST	EXERCISE2	40	50
	528	37.68	37.43	37.36	115	7	VEST	EXERCISE2	40	50
	529	37.68	37.43	37.36	115	7	VEST	EXERCISE2	40	50
	530	37.69	37.44	37.37	119	7	VEST	EXERCISE2	40	50
	531	37.70	37.45	37.36	117	7	VEST	EXERCISE2	40	50
	532	37.70	37.45	37.34	117	7	VEST	EXERCISE2	40	50
	533	37.70	37.45	37.34	115	7	VEST	EXERCISE2	40	50
	534	37.70	37.45	37.34	113	7	VEST	EXERCISE2	40	50
	535	37.71	37.45	37.34	116	7	VEST	EXERCISE2	40	50
	536	37.71	37.45	37.34	116	7	VEST	EXERCISE2	40	50
	537	37.71	37.45	37.34	114	7	VEST	EXERCISE2	40	50
	538	37.71	37.46	37.35	114	7	VEST	EXERCISE2	40	50
	539	37.71	37.47	37.36	116	7	VEST	EXERCISE2	40	50
	540	37.72	37.46	37.37	117	7	VEST	EXERCISE2	40	50
90	541	37.72	37.46	37.38	118	7	VEST	EXERCISE2	40	50
	542	37.72	37.46	37.39	117	7	VEST	EXERCISE2	40	50
	543	37.72	37.48	37.40	118	7	VEST	EXERCISE2	40	50

min	number	Tre	Tes	Tsk-head	HR	ID	condition	period	Ta	RH
	544	37.72	37.49	37.38	119	7	VEST	EXERCISE2	40	50
	545	37.71	37.49	37.37	121	7	VEST	EXERCISE2	40	50
	546	37.71	37.49	37.37	120	7	VEST	EXERCISE2	40	50
	547	37.72	37.49	37.37	119	7	VEST	EXERCISE2	40	50
	548	37.72	37.49	37.37	119	7	VEST	EXERCISE2	40	50
	549	37.72	37.50	37.39	120	7	VEST	EXERCISE2	40	50
	550	37.72	37.50	37.39	118	7	VEST	EXERCISE2	40	50
	551	37.71	37.50	37.39	116	7	VEST	EXERCISE2	40	50
	552	37.72	37.50	37.40	119	7	VEST	EXERCISE2	40	50
	553	37.72	37.50	37.39	119	7	VEST	EXERCISE2	40	50
	554	37.72	37.49	37.38	117	7	VEST	EXERCISE2	40	50
	555	37.73	37.50	37.39	118	7	VEST	EXERCISE2	40	50
	556	37.74	37.50	37.39	115	7	VEST	EXERCISE2	40	50
	557	37.75	37.51	37.39	116	7	VEST	EXERCISE2	40	50
	558	37.75	37.50	37.40	115	7	VEST	EXERCISE2	40	50
	559	37.75	37.51	37.40	118	7	VEST	EXERCISE2	40	50
	560	37.75	37.51	37.41	118	7	VEST	EXERCISE2	40	50
	561	37.74	37.50	37.40	119	7	VEST	EXERCISE2	40	50
	562	37.74	37.42	37.40	120	7	VEST	EXERCISE2	40	50
	563	37.75	37.37	37.41	120	7	VEST	EXERCISE2	40	50
	564	37.75	37.43	37.41	117	7	VEST	EXERCISE2	40	50
	565	37.75	37.46	37.42	118	7	VEST	EXERCISE2	40	50
	566	37.74	37.47	37.41	119	7	VEST	EXERCISE2	40	50
	567	37.74	37.47	37.41	117	7	VEST	EXERCISE2	40	50
	568	37.75	37.48	37.42	119	7	VEST	EXERCISE2	40	50
	569	37.76	37.49	37.41	121	7	VEST	EXERCISE2	40	50
	570	37.76	37.50	37.40	117	7	VEST	EXERCISE2	40	50
95	571	37.77	37.50	37.40	117	7	VEST	EXERCISE2	40	50
	572	37.78	37.50	37.41	117	7	VEST	EXERCISE2	40	50
	573	37.78	37.50	37.41	119	7	VEST	EXERCISE2	40	50
	574	37.78	37.50	37.42	121	7	VEST	EXERCISE2	40	50
	575	37.78	37.49	37.43	121	7	VEST	EXERCISE2	40	50
	576	37.79	37.49	37.44	120	7	VEST	EXERCISE2	40	50
	577	37.80	37.49	37.44	118	7	VEST	EXERCISE2	40	50
	578	37.80	37.42	37.44	123	7	VEST	EXERCISE2	40	50
	579	37.80	37.39	37.44	121	7	VEST	EXERCISE2	40	50
	580	37.80	37.44	37.45	125	7	VEST	EXERCISE2	40	50
	581	37.81	37.47	37.45	124	7	VEST	EXERCISE2	40	50
	582	37.80	37.49	37.45	120	7	VEST	EXERCISE2	40	50
	583	37.80	37.49	37.45	118	7	VEST	EXERCISE2	40	50
	584	37.81	37.50	37.46	119	7	VEST	EXERCISE2	40	50
	585	37.82	37.50	37.46	116	7	VEST	EXERCISE2	40	50
	586	37.81	37.51	37.45	119	7	VEST	EXERCISE2	40	50
	587	37.81	37.51	37.45	119	7	VEST	EXERCISE2	40	50
	588	37.82	37.51	37.45	119	7	VEST	EXERCISE2	40	50
	589	37.82	37.52	37.46	117	7	VEST	EXERCISE2	40	50

min	number	Tre	Tes	Tsk-head	HR	ID	condition	period	Ta	RH
	590	37.83	37.51	37.46	116	7	VEST	EXERCISE2	40	50
	591	37.83	37.51	37.46	123	7	VEST	EXERCISE2	40	50
	592	37.83	37.51	37.46	121	7	VEST	EXERCISE2	40	50
	593	37.82	37.51	37.46	123	7	VEST	EXERCISE2	40	50
	594	37.82	37.52	37.45	119	7	VEST	EXERCISE2	40	50
	595	37.83	37.51	37.46	119	7	VEST	EXERCISE2	40	50
	596	37.83	37.51	37.46	119	7	VEST	EXERCISE2	40	50
	597	37.83	37.51	37.46	117	7	VEST	EXERCISE2	40	50
	598	37.83	37.51	37.47	121	7	VEST	EXERCISE2	40	50
	599	37.84	37.51	37.47	119	7	VEST	EXERCISE2	40	50
	600	37.84	37.51	37.47	120	7	VEST	EXERCISE2	40	50
100	601	37.84	37.52	37.47	119	7	VEST	EXERCISE2	40	50
	602	37.84	37.52	37.47	122	7	VEST	EXERCISE2	40	50
	603	37.84	37.52	37.47	123	7	VEST	EXERCISE2	40	50
	604	37.85	37.52	37.47	121	7	VEST	EXERCISE2	40	50
	605	37.85	37.52	37.46	122	7	VEST	EXERCISE2	40	50
	606	37.85	37.53	37.46	124	7	VEST	EXERCISE2	40	50
	607	37.84	37.52	37.46	127	7	VEST	EXERCISE2	40	50
	608	37.84	37.52	37.45	125	7	VEST	EXERCISE2	40	50
	609	37.85	37.52	37.45	124	7	VEST	EXERCISE2	40	50
	610	37.86	37.51	37.45	121	7	VEST	EXERCISE2	40	50
	611	37.87	37.41	37.46	123	7	VEST	EXERCISE2	40	50
	612	37.86	37.38	37.46	121	7	VEST	EXERCISE2	40	50
	613	37.85	37.45	37.46	118	7	VEST	EXERCISE2	40	50
	614	37.85	37.47	37.47	121	7	VEST	EXERCISE2	40	50
	615	37.85	37.48	37.47	121	7	VEST	EXERCISE2	40	50
	616	37.86	37.48	37.46	120	7	VEST	EXERCISE2	40	50
	617	37.86	37.49	37.45	121	7	VEST	EXERCISE2	40	50
	618	37.86	37.49	37.45	119	7	VEST	EXERCISE2	40	50
	619	37.86	37.50	37.46	120	7	VEST	EXERCISE2	40	50
	620	37.87	37.50	37.46	116	7	VEST	EXERCISE2	40	50
	621	37.87	37.53	37.45	114	7	VEST	EXERCISE2	40	50
	622	37.88	37.55	37.46	121	7	VEST	EXERCISE2	40	50
	623	37.88	37.53	37.46	123	7	VEST	EXERCISE2	40	50
	624	37.88	37.51	37.46	124	7	VEST	EXERCISE2	40	50
	625	37.87	37.51	37.46	123	7	VEST	EXERCISE2	40	50
	626	37.87	37.51	37.46	124	7	VEST	EXERCISE2	40	50
	627	37.88	37.52	37.46	124	7	VEST	EXERCISE2	40	50
	628	37.89	37.52	37.46	124	7	VEST	EXERCISE2	40	50
	629	37.88	37.52	37.46	119	7	VEST	EXERCISE2	40	50
	630	37.88	37.52	37.46	121	7	VEST	EXERCISE2	40	50
105	631	37.89	37.53	37.46	121	7	VEST	EXERCISE2	40	50
	632	37.90	37.53	37.46	121	7	VEST	EXERCISE2	40	50
	633	37.90	37.53	37.46	122	7	VEST	EXERCISE2	40	50
	634	37.90	37.53	37.45	123	7	VEST	EXERCISE2	40	50
	635	37.90	37.53	37.45	122	7	VEST	EXERCISE2	40	50

min	number	Tre	Tes	Tsk-head	HR	ID	condition	period	Ta	RH
	636	37.90	37.54	37.47	124	7	VEST	EXERCISE2	40	50
	637	37.90	37.54	37.48	123	7	VEST	EXERCISE2	40	50
	638	37.90	37.54	37.47	123	7	VEST	EXERCISE2	40	50
	639	37.91	37.46	37.46	126	7	VEST	EXERCISE2	40	50
	640	37.92	37.43	37.46	121	7	VEST	EXERCISE2	40	50
	641	37.92	37.49	37.46	124	7	VEST	EXERCISE2	40	50
	642	37.91	37.50	37.47	123	7	VEST	EXERCISE2	40	50
	643	37.91	37.51	37.47	121	7	VEST	EXERCISE2	40	50
	644	37.92	37.51	37.46	118	7	VEST	REST3	28	50
	645	37.92	37.53	37.42	128	7	VEST	REST3	28	50
	646	37.92	37.55	37.31	121	7	VEST	REST3	28	50
	647	37.92	37.56	37.19	124	7	VEST	REST3	28	50
	648	37.92	37.58	37.10	122	7	VEST	REST3	28	50
	649	37.92	37.59	37.05	113	7	VEST	REST3	28	50
	650	37.92	37.59	37.05	100	7	VEST	REST3	28	50
	651	37.92	37.58	37.04	98	7	VEST	REST3	28	50
	652	37.92	37.58	37.02	103	7	VEST	REST3	28	50
	653	37.92	37.57	37.00	105	7	VEST	REST3	28	50
	654	37.92	37.53	36.99	102	7	VEST	REST3	28	50
	655	37.92	37.51	36.96	99	7	VEST	REST3	28	50
	656	37.92	37.49	36.94	103	7	VEST	REST3	28	50
	657	37.93	37.46	36.90	110	7	VEST	REST3	28	50
	658	37.93	37.43	36.85	104	7	VEST	REST3	28	50
	659	37.93	37.41	36.78	90	7	VEST	REST3	28	50
	660	37.92	37.37	36.72	89	7	VEST	REST3	28	50
110	661	37.92	37.27	36.70	103	7	VEST	REST3	28	50
	662	37.92	37.13	36.66	95	7	VEST	REST3	28	50
	663	37.92	37.11	36.58	89	7	VEST	REST3	28	50
	664	37.92	37.23	36.51	91	7	VEST	REST3	28	50
	665	37.92	37.22	36.49	89	7	VEST	REST3	28	50
	666	37.92	37.13	36.49	97	7	VEST	REST3	28	50
	667	37.93	37.15	36.50	87	7	VEST	REST3	28	50
	668	37.93	37.23	36.48	88	7	VEST	REST3	28	50
	669	37.93	37.21	36.46	88	7	VEST	REST3	28	50
	670	37.92	37.21	36.45	86	7	VEST	REST3	28	50
	671	37.92	37.22	36.42	87	7	VEST	REST3	28	50
	672	37.92	37.22	36.40	86	7	VEST	REST3	28	50
	673	37.93	37.25	36.40	87	7	VEST	REST3	28	50
	674	37.93	37.26	36.39	87	7	VEST	REST3	28	50
	675	37.93	37.18	36.37	90	7	VEST	REST3	28	50
	676	37.93	37.15	36.33	86	7	VEST	REST3	28	50
	677	37.93	37.23	36.31	87	7	VEST	REST3	28	50
	678	37.93	37.22	36.30	87	7	VEST	REST3	28	50
	679	37.93	37.19	36.29	86	7	VEST	REST3	28	50
	680	37.92	37.23	36.27	87	7	VEST	REST3	28	50
	681	37.91	37.23	36.25	94	7	VEST	REST3	28	50

min	number	Tre	Tes	Tsk-head	HR	ID	condition	period	Ta	RH
	682	37.91	37.23	36.23	92	7	VEST	REST3	28	50
	683	37.91	37.18	36.19	101	7	VEST	REST3	28	50
	684	37.90	37.20	36.13	91	7	VEST	REST3	28	50
	685	37.89	37.25	36.06	93	7	VEST	REST3	28	50
	686	37.88	37.24	36.03	88	7	VEST	REST3	28	50
	687	37.88	37.27	36.03	91	7	VEST	REST3	28	50
	688	37.87	37.29	35.99	83	7	VEST	REST3	28	50
	689	37.86	37.30	35.98	83	7	VEST	REST3	28	50
	690	37.86	37.29	35.97	87	7	VEST	REST3	28	50
115	691	37.86	37.30	35.97	84	7	VEST	REST3	28	50
	692	37.85	37.31	35.98	86	7	VEST	REST3	28	50
	693	37.84	37.30	35.99	82	7	VEST	REST3	28	50
	694	37.83	37.30	35.99	83	7	VEST	REST3	28	50
	695	37.82	37.26	35.96	91	7	VEST	REST3	28	50
	696	37.80	37.24	35.93	92	7	VEST	REST3	28	50
	697	37.79	37.27	35.89	91	7	VEST	REST3	28	50
	698	37.78	37.24	35.87	91	7	VEST	REST3	28	50
	699	37.77	37.25	35.89	87	7	VEST	REST3	28	50
	700	37.76	37.29	35.86	89	7	VEST	REST3	28	50
	701	37.75	37.29	35.83	89	7	VEST	REST3	28	50
	702	37.74	37.30	35.82	83	7	VEST	REST3	28	50
	703	37.73	37.31	35.82	84	7	VEST	REST3	28	50
	704	37.72	37.31	35.83	80	7	VEST	REST3	28	50
	705	37.71	37.31	35.83	79	7	VEST	REST3	28	50
	706	37.71	37.24	35.80	88	7	VEST	REST3	28	50
	707	37.71	37.21	35.79	87	7	VEST	REST3	28	50
	708	37.71	37.26	35.79	86	7	VEST	REST3	28	50
	709	37.70	37.27	35.78	84	7	VEST	REST3	28	50
0	1	36.99	36.76	35.86	65	8	VEST	REST1	28	50
	2	36.99	36.78	35.88	70	8	VEST	REST1	28	50
	3	37.00	36.79	35.90	65	8	VEST	REST1	28	50
	4	37.00	36.79	35.91	66	8	VEST	REST1	28	50
	5	37.00	36.76	35.91	71	8	VEST	REST1	28	50
	6	37.00	36.74	35.92	69	8	VEST	REST1	28	50
	7	37.00	36.75	35.94	68	8	VEST	REST1	28	50
	8	37.00	36.76	35.95	70	8	VEST	REST1	28	50
	9	37.00	36.78	35.95	70	8	VEST	REST1	28	50
	10	37.00	36.77	35.94	69	8	VEST	REST1	28	50
	11	37.00	36.77	35.93	70	8	VEST	REST1	28	50
	12	37.00	36.79	35.92	68	8	VEST	REST1	28	50
	13	37.01	36.78	35.92	69	8	VEST	REST1	28	50
	14	37.01	36.76	35.91	72	8	VEST	REST1	28	50
	15	37.00	36.75	35.91	68	8	VEST	REST1	28	50
	16	37.01	36.76	35.92	68	8	VEST	REST1	28	50
	17	37.02	36.77	35.94	77	8	VEST	REST1	28	50
	18	37.02	36.75	35.95	75	8	VEST	REST1	28	50

min	number	Tre	Tes	Tsk-head	HR	ID	condition	period	Ta	RH
	19	37.01	36.77	35.98	70	8	VEST	REST1	28	50
	20	37.01	36.77	36.03	79	8	VEST	REST1	28	50
	21	37.01	36.76	36.06	75	8	VEST	REST1	28	50
	22	37.01	36.77	36.10	75	8	VEST	REST1	28	50
	23	37.01	36.76	36.15	70	8	VEST	REST1	28	50
	24	37.01	36.75	36.19	70	8	VEST	REST1	28	50
	25	37.00	36.72	36.19	74	8	VEST	REST1	28	50
	26	37.00	36.71	36.17	71	8	VEST	REST1	28	50
	27	37.01	36.74	36.14	65	8	VEST	REST1	28	50
	28	37.01	36.73	36.11	69	8	VEST	REST1	28	50
	29	37.01	36.73	36.08	70	8	VEST	REST1	28	50
	30	37.01	36.72	36.04	69	8	VEST	REST1	28	50
5	31	37.00	36.73	36.00	70	8	VEST	REST1	28	50
	32	37.01	36.76	35.97	71	8	VEST	REST1	28	50
	33	37.02	36.75	35.96	81	8	VEST	REST1	28	50
	34	37.02	36.74	35.94	73	8	VEST	REST1	28	50
	35	37.01	36.72	35.93	67	8	VEST	REST1	28	50
	36	37.00	36.73	35.91	68	8	VEST	REST1	28	50
	37	37.01	36.75	35.88	67	8	VEST	REST1	28	50
	38	37.01	36.76	35.87	70	8	VEST	REST1	28	50
	39	37.01	36.77	35.87	70	8	VEST	REST1	28	50
	40	37.02	36.76	35.88	75	8	VEST	REST1	28	50
	41	37.02	36.75	35.89	72	8	VEST	REST1	28	50
	42	37.01	36.76	35.88	68	8	VEST	REST1	28	50
	43	37.01	36.78	35.87	68	8	VEST	REST1	28	50
	44	37.02	36.77	35.88	74	8	VEST	REST1	28	50
	45	37.02	36.77	35.88	71	8	VEST	REST1	28	50
	46	37.02	36.76	35.87	77	8	VEST	REST1	28	50
	47	37.02	36.75	35.88	85	8	VEST	REST1	28	50
	48	37.02	36.77	35.87	78	8	VEST	REST1	28	50
	49	37.03	36.78	35.87	67	8	VEST	REST1	28	50
	50	37.02	36.69	35.88	76	8	VEST	REST1	28	50
	51	37.02	36.65	35.88	70	8	VEST	REST1	28	50
	52	37.03	36.73	35.88	65	8	VEST	REST1	28	50
	53	37.03	36.74	35.88	72	8	VEST	REST1	28	50
	54	37.02	36.73	35.89	74	8	VEST	REST1	28	50
	55	37.02	36.73	35.89	68	8	VEST	REST1	28	50
	56	37.02	36.74	35.89	68	8	VEST	REST1	28	50
	57	36.98	36.76	35.90	76	8	VEST	REST1	28	50
	58	36.91	36.76	35.90	74	8	VEST	REST1	28	50
	59	36.86	36.75	35.91	73	8	VEST	REST1	28	50
	60	36.83	36.75	35.91	68	8	VEST	REST1	28	50
10	61	36.86	36.77	35.92	79	8	VEST	REST1	28	50
	62	36.92	36.76	35.92	65	8	VEST	REST1	28	50
	63	36.96	36.75	35.92	70	8	VEST	REST1	28	50
	64	36.99	36.75	35.92	69	8	VEST	REST1	28	50

min	number	Tre	Tes	Tsk-head	HR	ID	condition	period	Ta	RH
	65	37.00	36.75	35.92	69	8	VEST	REST1	28	50
	66	37.00	36.76	35.92	67	8	VEST	REST1	28	50
	67	37.00	36.77	35.93	70	8	VEST	REST1	28	50
	68	37.00	36.76	35.93	72	8	VEST	REST1	28	50
	69	37.00	36.75	35.93	69	8	VEST	REST1	28	50
	70	37.00	36.76	35.94	84	8	VEST	REST1	28	50
	71	37.00	36.79	35.95	73	8	VEST	REST1	28	50
	72	37.00	36.80	35.96	72	8	VEST	REST1	28	50
	73	37.00	36.79	35.97	79	8	VEST	REST1	28	50
	74	36.99	36.77	35.96	69	8	VEST	REST1	28	50
	75	36.99	36.76	35.96	82	8	VEST	REST1	28	50
	76	37.00	36.77	35.97	81	8	VEST	REST1	28	50
	77	37.00	36.76	35.97	74	8	VEST	REST1	28	50
	78	36.99	36.77	35.97	67	8	VEST	REST1	28	50
	79	36.98	36.78	35.98	69	8	VEST	REST1	28	50
	80	36.98	36.79	35.99	70	8	VEST	REST1	28	50
	81	36.99	36.80	36.00	73	8	VEST	REST1	28	50
	82	36.99	36.79	35.99	78	8	VEST	REST1	28	50
	83	36.99	36.81	35.99	82	8	VEST	REST1	28	50
	84	37.00	36.82	36.00	68	8	VEST	REST1	28	50
	85	37.00	36.81	35.99	71	8	VEST	REST1	28	50
	86	37.00	36.81	35.99	70	8	VEST	REST1	28	50
	87	37.00	36.80	35.99	75	8	VEST	REST1	28	50
	88	36.99	36.80	35.98	72	8	VEST	REST1	28	50
	89	36.99	36.81	35.99	71	8	VEST	REST1	28	50
	90	37.00	36.82	36.00	79	8	VEST	REST1	28	50
15	91	37.00	36.80	36.00	84	8	VEST	REST1	28	50
	92	37.01	36.79	36.00	77	8	VEST	REST1	28	50
	93	37.01	36.80	36.03	76	8	VEST	REST1	28	50
	94	37.00	36.80	36.07	85	8	VEST	REST1	28	50
	95	37.00	36.80	36.07	76	8	VEST	REST1	28	50
	96	37.00	36.80	36.05	65	8	VEST	REST1	28	50
	97	37.00	36.80	36.04	69	8	VEST	REST1	28	50
	98	37.00	36.80	36.03	75	8	VEST	REST1	28	50
	99	37.00	36.81	36.07	74	8	VEST	REST1	28	50
	100	37.00	36.82	36.11	73	8	VEST	REST1	28	50
	101	37.00	36.83	36.10	71	8	VEST	REST1	28	50
	102	36.99	36.82	36.09	87	8	VEST	REST1	28	50
	103	36.99	36.81	36.12	74	8	VEST	REST1	40	50
	104	36.98	36.83	36.16	83	8	VEST	REST1	40	50
	105	36.96	36.82	36.23	90	8	VEST	REST1	40	50
	106	36.94	36.83	36.34	71	8	VEST	REST1	40	50
	107	36.92	36.88	36.44	75	8	VEST	REST1	40	50
	108	36.91	36.82	36.51	77	8	VEST	REST1	40	50
	109	36.90	36.75	36.57	71	8	VEST	REST1	40	50
	110	36.90	36.77	36.60	77	8	VEST	REST1	40	50

min	number	Tre	Tes	Tsk-head	HR	ID	condition	period	Ta	RH
	111	36.90	36.77	36.65	75	8	VEST	REST1	40	50
	112	36.91	36.77	36.69	70	8	VEST	REST1	40	50
	113	36.90	36.77	36.70	71	8	VEST	REST1	40	50
	114	36.89	36.73	36.71	79	8	VEST	REST1	40	50
	115	36.89	36.68	36.74	78	8	VEST	REST1	40	50
	116	36.88	36.66	36.75	76	8	VEST	REST1	40	50
	117	36.88	36.70	36.76	69	8	VEST	REST1	40	50
	118	36.89	36.72	36.78	68	8	VEST	REST1	40	50
	119	36.90	36.72	36.78	77	8	VEST	REST1	40	50
	120	36.91	36.74	36.79	76	8	VEST	REST1	40	50
20	121	36.93	36.76	36.80	70	8	VEST	REST1	40	50
	122	36.96	36.76	36.82	69	8	VEST	REST1	40	50
	123	36.97	36.76	36.82	70	8	VEST	REST1	40	50
	124	36.97	36.78	36.82	73	8	VEST	REST1	40	50
	125	36.97	36.78	36.84	68	8	VEST	REST1	40	50
	126	36.98	36.78	36.87	68	8	VEST	REST1	40	50
	127	36.98	36.79	36.87	68	8	VEST	REST1	40	50
	128	36.98	36.79	36.88	76	8	VEST	REST1	40	50
	129	36.98	36.79	36.89	76	8	VEST	REST1	40	50
	130	36.98	36.79	36.88	82	8	VEST	REST1	40	50
	131	36.99	36.78	36.89	72	8	VEST	REST1	40	50
	132	37.00	36.81	36.92	69	8	VEST	REST1	40	50
	133	36.99	36.82	36.91	71	8	VEST	REST1	40	50
	134	36.99	36.83	36.92	83	8	VEST	REST1	40	50
	135	36.99	36.82	36.94	99	8	VEST	REST1	40	50
	136	36.98	36.81	36.95	82	8	VEST	REST1	40	50
	137	36.98	36.81	36.96	79	8	VEST	REST1	40	50
	138	36.99	36.81	36.96	81	8	VEST	REST1	40	50
	139	36.99	36.81	36.98	78	8	VEST	EXERCISE1	40	50
	140	37.00	36.83	36.99	79	8	VEST	EXERCISE1	40	50
	141	37.00	36.83	37.00	89	8	VEST	EXERCISE1	40	50
	142	37.00	36.81	37.03	91	8	VEST	EXERCISE1	40	50
	143	37.00	36.82	37.04	96	8	VEST	EXERCISE1	40	50
	144	37.00	36.80	37.06	97	8	VEST	EXERCISE1	40	50
	145	37.00	36.78	37.07	98	8	VEST	EXERCISE1	40	50
	146	37.00	36.79	37.08	94	8	VEST	EXERCISE1	40	50
	147	37.00	36.80	37.08	97	8	VEST	EXERCISE1	40	50
	148	37.00	36.79	37.09	98	8	VEST	EXERCISE1	40	50
	149	37.01	36.76	37.10	101	8	VEST	EXERCISE1	40	50
	150	37.02	36.75	37.12	96	8	VEST	EXERCISE1	40	50
25	151	37.02	36.79	37.12	97	8	VEST	EXERCISE1	40	50
	152	37.02	36.79	37.12	97	8	VEST	EXERCISE1	40	50
	153	37.01	36.79	37.13	100	8	VEST	EXERCISE1	40	50
	154	37.02	36.81	37.13	96	8	VEST	EXERCISE1	40	50
	155	37.01	36.83	37.12	97	8	VEST	EXERCISE1	40	50
	156	37.01	36.84	37.12	98	8	VEST	EXERCISE1	40	50

min	number	Tre	Tes	Tsk-head	HR	ID	condition	period	Ta	RH
	157	37.02	36.85	37.13	97	8	VEST	EXERCISE1	40	50
	158	37.02	36.86	37.11	104	8	VEST	EXERCISE1	40	50
	159	37.02	36.85	37.11	103	8	VEST	EXERCISE1	40	50
	160	37.02	36.85	37.13	103	8	VEST	EXERCISE1	40	50
	161	37.02	36.88	37.14	100	8	VEST	EXERCISE1	40	50
	162	37.02	36.87	37.14	104	8	VEST	EXERCISE1	40	50
	163	37.03	36.87	37.16	100	8	VEST	EXERCISE1	40	50
	164	37.03	36.90	37.15	101	8	VEST	EXERCISE1	40	50
	165	37.03	36.93	37.14	101	8	VEST	EXERCISE1	40	50
	166	37.04	36.95	37.14	103	8	VEST	EXERCISE1	40	50
	167	37.04	36.94	37.15	103	8	VEST	EXERCISE1	40	50
	168	37.04	36.95	37.15	102	8	VEST	EXERCISE1	40	50
	169	37.04	36.98	37.14	104	8	VEST	EXERCISE1	40	50
	170	37.04	36.97	37.15	105	8	VEST	EXERCISE1	40	50
	171	37.04	36.97	37.14	104	8	VEST	EXERCISE1	40	50
	172	37.04	36.99	37.15	101	8	VEST	EXERCISE1	40	50
	173	37.04	36.98	37.16	103	8	VEST	EXERCISE1	40	50
	174	37.05	36.98	37.16	103	8	VEST	EXERCISE1	40	50
	175	37.05	37.00	37.16	99	8	VEST	EXERCISE1	40	50
	176	37.06	37.02	37.14	105	8	VEST	EXERCISE1	40	50
	177	37.06	37.03	37.14	102	8	VEST	EXERCISE1	40	50
	178	37.06	37.03	37.15	104	8	VEST	EXERCISE1	40	50
	179	37.06	37.05	37.14	104	8	VEST	EXERCISE1	40	50
	180	37.06	37.06	37.14	103	8	VEST	EXERCISE1	40	50
30	181	37.06	37.05	37.16	102	8	VEST	EXERCISE1	40	50
	182	37.06	37.02	37.15	106	8	VEST	EXERCISE1	40	50
	183	37.07	37.04	37.15	103	8	VEST	EXERCISE1	40	50
	184	37.08	37.08	37.16	106	8	VEST	EXERCISE1	40	50
	185	37.09	37.07	37.16	106	8	VEST	EXERCISE1	40	50
	186	37.09	37.07	37.15	105	8	VEST	EXERCISE1	40	50
	187	37.09	37.05	37.17	109	8	VEST	EXERCISE1	40	50
	188	37.10	37.02	37.16	106	8	VEST	EXERCISE1	40	50
	189	37.09	37.02	37.15	108	8	VEST	EXERCISE1	40	50
	190	37.09	37.02	37.14	109	8	VEST	EXERCISE1	40	50
	191	37.09	37.03	37.12	109	8	VEST	EXERCISE1	40	50
	192	37.09	37.04	37.10	107	8	VEST	EXERCISE1	40	50
	193	37.09	36.99	37.11	109	8	VEST	EXERCISE1	40	50
	194	37.09	36.97	37.11	102	8	VEST	EXERCISE1	40	50
	195	37.10	37.00	37.10	103	8	VEST	EXERCISE1	40	50
	196	37.10	37.00	37.09	106	8	VEST	EXERCISE1	40	50
	197	37.10	37.00	37.08	106	8	VEST	EXERCISE1	40	50
	198	37.12	37.03	37.05	106	8	VEST	EXERCISE1	40	50
	199	37.12	37.05	37.02	107	8	VEST	EXERCISE1	40	50
	200	37.12	36.98	37.01	108	8	VEST	EXERCISE1	40	50
	201	37.12	36.94	36.99	106	8	VEST	EXERCISE1	40	50
	202	37.12	36.98	36.97	106	8	VEST	EXERCISE1	40	50

min	number	Tre	Tes	Tsk-head	HR	ID	condition	period	Ta	RH
	203	37.13	36.96	36.98	110	8	VEST	EXERCISE1	40	50
	204	37.13	36.92	36.99	108	8	VEST	EXERCISE1	40	50
	205	37.14	36.97	36.98	103	8	VEST	EXERCISE1	40	50
	206	37.14	37.01	36.96	102	8	VEST	EXERCISE1	40	50
	207	37.15	36.97	36.95	107	8	VEST	EXERCISE1	40	50
	208	37.15	36.94	36.96	105	8	VEST	EXERCISE1	40	50
	209	37.15	36.97	36.99	104	8	VEST	EXERCISE1	40	50
	210	37.15	37.08	36.99	104	8	VEST	EXERCISE1	40	50
35	211	37.15	37.08	36.97	104	8	VEST	EXERCISE1	40	50
	212	37.15	36.98	36.97	106	8	VEST	EXERCISE1	40	50
	213	37.16	36.96	36.99	106	8	VEST	EXERCISE1	40	50
	214	37.16	36.97	37.01	108	8	VEST	EXERCISE1	40	50
	215	37.16	36.97	37.01	109	8	VEST	EXERCISE1	40	50
	216	37.16	36.99	37.00	106	8	VEST	EXERCISE1	40	50
	217	37.16	37.02	37.01	106	8	VEST	EXERCISE1	40	50
	218	37.16	36.96	37.03	110	8	VEST	EXERCISE1	40	50
	219	37.17	36.96	37.03	108	8	VEST	EXERCISE1	40	50
	220	37.17	37.03	37.03	109	8	VEST	EXERCISE1	40	50
	221	37.17	37.05	37.04	110	8	VEST	EXERCISE1	40	50
	222	37.16	37.06	37.05	110	8	VEST	EXERCISE1	40	50
	223	37.17	37.07	37.03	106	8	VEST	EXERCISE1	40	50
	224	37.17	37.07	37.01	109	8	VEST	EXERCISE1	40	50
	225	37.17	37.08	37.01	110	8	VEST	EXERCISE1	40	50
	226	37.18	36.99	37.04	114	8	VEST	EXERCISE1	40	50
	227	37.18	36.97	37.05	111	8	VEST	EXERCISE1	40	50
	228	37.18	37.06	37.04	114	8	VEST	EXERCISE1	40	50
	229	37.17	37.04	37.03	113	8	VEST	EXERCISE1	40	50
	230	37.18	37.03	37.03	109	8	VEST	EXERCISE1	40	50
	231	37.19	36.98	37.04	111	8	VEST	EXERCISE1	40	50
	232	37.20	36.99	37.03	112	8	VEST	EXERCISE1	40	50
	233	37.20	37.04	37.02	114	8	VEST	EXERCISE1	40	50
	234	37.20	36.95	37.03	116	8	VEST	EXERCISE1	40	50
	235	37.20	36.96	37.04	112	8	VEST	EXERCISE1	40	50
	236	37.20	37.05	37.04	111	8	VEST	EXERCISE1	40	50
	237	37.21	37.07	37.03	113	8	VEST	EXERCISE1	40	50
	238	37.20	37.06	37.03	114	8	VEST	EXERCISE1	40	50
	239	37.20	37.05	37.02	113	8	VEST	EXERCISE1	40	50
	240	37.20	36.99	37.01	114	8	VEST	EXERCISE1	40	50
40	241	37.21	36.99	37.00	112	8	VEST	EXERCISE1	40	50
	242	37.22	37.05	37.00	114	8	VEST	EXERCISE1	40	50
	243	37.22	37.05	37.00	115	8	VEST	EXERCISE1	40	50
	244	37.22	37.09	37.01	111	8	VEST	EXERCISE1	40	50
	245	37.23	37.08	37.02	114	8	VEST	EXERCISE1	40	50
	246	37.22	37.05	37.04	113	8	VEST	EXERCISE1	40	50
	247	37.22	37.06	37.02	114	8	VEST	EXERCISE1	40	50
	248	37.21	37.07	37.01	113	8	VEST	EXERCISE1	40	50

min	number	Tre	Tes	Tsk-head	HR	ID	condition	period	Ta	RH
	249	37.21	37.07	37.03	111	8	VEST	EXERCISE1	40	50
	250	37.21	37.08	37.04	113	8	VEST	EXERCISE1	40	50
	251	37.22	37.10	37.06	116	8	VEST	EXERCISE1	40	50
	252	37.23	37.11	37.06	115	8	VEST	EXERCISE1	40	50
	253	37.23	37.07	37.05	115	8	VEST	EXERCISE1	40	50
	254	37.23	37.06	37.04	112	8	VEST	EXERCISE1	40	50
	255	37.23	37.09	37.06	110	8	VEST	EXERCISE1	40	50
	256	37.24	37.09	37.07	113	8	VEST	EXERCISE1	40	50
	257	37.24	37.11	37.05	116	8	VEST	EXERCISE1	40	50
	258	37.25	37.09	37.06	109	8	VEST	EXERCISE1	40	50
	259	37.25	37.08	37.06	109	8	VEST	EXERCISE1	40	50
	260	37.25	37.09	37.07	111	8	VEST	EXERCISE1	40	50
	261	37.26	37.11	37.09	112	8	VEST	EXERCISE1	40	50
	262	37.26	37.11	37.10	115	8	VEST	EXERCISE1	40	50
	263	37.26	37.08	37.10	108	8	VEST	EXERCISE1	40	50
	264	37.26	37.09	37.09	110	8	VEST	EXERCISE1	40	50
	265	37.27	37.13	37.07	115	8	VEST	EXERCISE1	40	50
	266	37.28	37.15	37.06	117	8	VEST	EXERCISE1	40	50
	267	37.27	37.15	37.06	115	8	VEST	EXERCISE1	40	50
	268	37.27	37.01	37.06	120	8	VEST	EXERCISE1	40	50
	269	37.28	37.01	37.04	116	8	VEST	EXERCISE1	40	50
	270	37.28	37.15	37.02	115	8	VEST	EXERCISE1	40	50
45	271	37.28	37.14	37.03	118	8	VEST	EXERCISE1	40	50
	272	37.28	37.12	37.04	113	8	VEST	EXERCISE1	40	50
	273	37.29	37.11	37.05	112	8	VEST	EXERCISE1	40	50
	274	37.30	37.13	37.03	112	8	VEST	EXERCISE1	40	50
	275	37.29	37.12	37.03	114	8	VEST	EXERCISE1	40	50
	276	37.30	37.13	37.04	114	8	VEST	EXERCISE1	40	50
	277	37.30	37.11	37.04	121	8	VEST	EXERCISE1	40	50
	278	37.30	37.08	37.04	117	8	VEST	EXERCISE1	40	50
	279	37.30	37.11	37.05	114	8	VEST	EXERCISE1	40	50
	280	37.31	37.06	37.07	119	8	VEST	EXERCISE1	40	50
	281	37.32	37.06	37.06	117	8	VEST	EXERCISE1	40	50
	282	37.32	37.12	37.05	117	8	VEST	EXERCISE1	40	50
	283	37.32	37.14	37.06	118	8	VEST	EXERCISE1	40	50
	284	37.32	37.09	37.07	120	8	VEST	EXERCISE1	40	50
	285	37.32	37.06	37.06	117	8	VEST	EXERCISE1	40	50
	286	37.32	36.97	37.04	119	8	VEST	EXERCISE1	40	50
	287	37.32	36.97	37.04	118	8	VEST	EXERCISE1	40	50
	288	37.32	37.09	37.06	119	8	VEST	EXERCISE1	40	50
	289	37.31	37.09	37.08	120	8	VEST	EXERCISE1	40	50
	290	37.32	37.10	37.09	119	8	VEST	EXERCISE1	40	50
	291	37.33	37.03	37.09	121	8	VEST	EXERCISE1	40	50
	292	37.33	37.05	37.07	118	8	VEST	EXERCISE1	40	50
	293	37.34	37.13	37.08	118	8	VEST	EXERCISE1	40	50
	294	37.34	37.10	37.10	116	8	VEST	EXERCISE1	40	50

min	number	Tre	Tes	Tsk-head	HR	ID	condition	period	Ta	RH
	295	37.33	37.12	37.10	112	8	VEST	EXERCISE1	40	50
	296	37.33	37.14	37.09	113	8	VEST	EXERCISE1	40	50
	297	37.34	37.13	37.09	117	8	VEST	EXERCISE1	40	50
	298	37.34	37.15	37.09	118	8	VEST	EXERCISE1	40	50
	299	37.33	37.13	37.09	122	8	VEST	EXERCISE1	40	50
	300	37.33	37.12	37.09	122	8	VEST	EXERCISE1	40	50
50	301	37.33	37.12	37.10	119	8	VEST	EXERCISE1	40	50
	302	37.34	37.13	37.11	117	8	VEST	EXERCISE1	40	50
	303	37.34	37.17	37.10	120	8	VEST	EXERCISE1	40	50
	304	37.35	37.14	37.09	122	8	VEST	EXERCISE1	40	50
	305	37.35	37.10	37.10	118	8	VEST	EXERCISE1	40	50
	306	37.35	37.15	37.09	117	8	VEST	EXERCISE1	40	50
	307	37.35	37.19	37.09	119	8	VEST	EXERCISE1	40	50
	308	37.36	37.16	37.12	123	8	VEST	EXERCISE1	40	50
	309	37.36	37.15	37.13	121	8	VEST	EXERCISE1	40	50
	310	37.35	37.16	37.13	121	8	VEST	EXERCISE1	40	50
	311	37.36	37.13	37.13	121	8	VEST	EXERCISE1	40	50
	312	37.36	37.13	37.11	120	8	VEST	EXERCISE1	40	50
	313	37.36	37.13	37.10	122	8	VEST	EXERCISE1	40	50
	314	37.36	37.13	37.10	122	8	VEST	EXERCISE1	40	50
	315	37.36	37.14	37.13	120	8	VEST	EXERCISE1	40	50
	316	37.36	37.13	37.15	120	8	VEST	EXERCISE1	40	50
	317	37.37	37.10	37.15	121	8	VEST	EXERCISE1	40	50
	318	37.37	37.09	37.14	120	8	VEST	EXERCISE1	40	50
	319	37.38	37.15	37.12	124	8	VEST	EXERCISE1	40	50
	320	37.38	37.16	37.12	117	8	VEST	REST2	28	50
	321	37.37	37.16	37.05	123	8	VEST	REST2	28	50
	322	37.37	37.20	36.92	116	8	VEST	REST2	28	50
	323	37.37	37.22	36.86	105	8	VEST	REST2	28	50
	324	37.37	37.22	36.83	97	8	VEST	REST2	28	50
	325	37.37	37.20	36.79	99	8	VEST	REST2	28	50
	326	37.37	37.11	36.69	104	8	VEST	REST2	28	50
	327	37.38	37.08	36.61	103	8	VEST	REST2	28	50
	328	37.37	37.10	36.60	97	8	VEST	REST2	28	50
	329	37.37	37.11	36.57	88	8	VEST	REST2	28	50
	330	37.37	37.09	36.54	84	8	VEST	REST2	28	50
55	331	37.38	37.08	36.46	89	8	VEST	REST2	28	50
	332	37.38	37.08	36.36	87	8	VEST	REST2	28	50
	333	37.38	37.04	36.36	82	8	VEST	REST2	28	50
	334	37.38	37.02	36.35	85	8	VEST	REST2	28	50
	335	37.38	37.01	36.31	89	8	VEST	REST2	28	50
	336	37.39	37.01	36.28	88	8	VEST	REST2	28	50
	337	37.38	36.99	36.22	89	8	VEST	REST2	28	50
	338	37.38	32.98	36.18	84	8	VEST	REST2	28	50
	339	37.38	29.74	36.20	96	8	VEST	REST2	28	50
	340	37.38	30.79	36.23	91	8	VEST	REST2	28	50

min	number	Tre	Tes	Tsk-head	HR	ID	condition	period	Ta	RH
	341	37.38	31.90	36.22	93	8	VEST	REST2	28	50
	342	37.38	33.06	36.17	81	8	VEST	REST2	28	50
	343	37.38	33.72	36.11	85	8	VEST	REST2	28	50
	344	37.39	34.30	36.06	87	8	VEST	REST2	28	50
	345	37.39	34.76	36.02	84	8	VEST	REST2	28	50
	346	37.40	35.06	36.04	82	8	VEST	REST2	28	50
	347	37.40	35.33	36.07	82	8	VEST	REST2	28	50
	348	37.40	35.56	36.07	89	8	VEST	REST2	28	50
	349	37.40	35.71	36.04	86	8	VEST	REST2	28	50
	350	37.40	35.85	36.05	88	8	VEST	REST2	28	50
	351	37.40	34.91	36.09	89	8	VEST	REST2	28	50
	352	37.39	34.34	36.03	83	8	VEST	REST2	28	50
	353	37.39	34.98	35.99	82	8	VEST	REST2	28	50
	354	37.39	35.31	36.02	84	8	VEST	REST2	28	50
	355	37.38	35.47	36.02	83	8	VEST	REST2	28	50
	356	37.37	35.61	36.00	83	8	VEST	REST2	28	50
	357	37.38	35.83	35.96	79	8	VEST	REST2	28	50
	358	37.39	36.02	35.97	84	8	VEST	REST2	28	50
	359	37.38	36.15	35.97	77	8	VEST	REST2	28	50
	360	37.38	36.25	35.93	81	8	VEST	REST2	28	50
60	361	37.38	33.08	35.92	84	8	VEST	REST2	28	50
	362	37.38	31.02	35.94	83	8	VEST	REST2	28	50
	363	37.38	32.98	35.92	82	8	VEST	REST2	28	50
	364	37.37	34.09	35.93	73	8	VEST	REST2	28	50
	365	37.36	34.60	35.89	74	8	VEST	REST2	28	50
	366	37.36	34.94	35.88	80	8	VEST	REST2	28	50
	367	37.37	35.29	35.94	76	8	VEST	REST2	28	50
	368	37.37	35.57	35.96	81	8	VEST	REST2	28	50
	369	37.37	35.78	35.98	80	8	VEST	REST2	28	50
	370	37.37	35.96	35.97	81	8	VEST	REST2	28	50
	371	37.36	36.07	35.95	77	8	VEST	REST2	28	50
	372	37.35	36.17	35.90	80	8	VEST	REST2	28	50
	373	37.35	36.29	35.83	73	8	VEST	REST2	28	50
	374	37.35	36.39	35.86	79	8	VEST	REST2	28	50
	375	37.35	36.45	35.92	82	8	VEST	REST2	28	50
	376	37.36	32.90	35.91	91	8	VEST	REST2	28	50
	377	37.35	31.03	35.89	68	8	VEST	REST2	28	50
	378	37.35	33.13	35.92	80	8	VEST	REST2	28	50
	379	37.35	33.92	35.94	83	8	VEST	REST2	28	50
	380	37.34	34.59	35.95	80	8	VEST	REST2	28	50
	381	37.34	34.79	35.96	78	8	VEST	REST2	28	50
	382	37.34	35.02	35.96	81	8	VEST	REST2	28	50
	383	37.33	35.41	35.92	82	8	VEST	REST2	28	50
	384	37.32	35.55	35.92	86	8	VEST	REST2	28	50
	385	37.32	35.76	35.95	83	8	VEST	REST2	28	50
	386	37.34	36.01	35.96	75	8	VEST	REST2	28	50

min	number	Tre	Tes	Tsk-head	HR	ID	condition	period	Ta	RH
	387	37.34	36.17	35.97	88	8	VEST	REST2	28	50
	388	37.34	36.15	35.97	71	8	VEST	REST2	28	50
	389	37.33	36.24	35.97	83	8	VEST	REST2	28	50
	390	37.33	36.33	35.94	81	8	VEST	REST2	28	50
65	391	37.33	36.04	35.92	80	8	VEST	REST2	28	50
	392	37.34	36.19	35.92	84	8	VEST	REST2	28	50
	393	37.34	32.85	35.95	87	8	VEST	REST2	28	50
	394	37.33	30.78	35.97	85	8	VEST	REST2	28	50
	395	37.33	31.85	35.94	84	8	VEST	REST2	28	50
	396	37.33	29.51	35.94	86	8	VEST	REST2	28	50
	397	37.34	29.29	35.94	88	8	VEST	REST2	28	50
	398	37.34	31.45	35.94	81	8	VEST	REST2	28	50
	399	37.33	32.44	35.94	80	8	VEST	REST2	28	50
	400	37.32	33.05	35.94	82	8	VEST	REST2	28	50
	401	37.31	33.61	35.96	79	8	VEST	REST2	28	50
	402	37.32	33.96	35.97	84	8	VEST	REST2	28	50
	403	37.31	34.26	35.95	83	8	VEST	REST2	28	50
	404	37.31	34.76	35.97	75	8	VEST	REST2	28	50
	405	37.30	35.09	35.96	83	8	VEST	REST2	28	50
	406	37.30	35.34	35.93	80	8	VEST	REST2	28	50
	407	37.29	35.66	35.92	74	8	VEST	REST2	28	50
	408	37.29	35.96	35.92	80	8	VEST	REST2	28	50
	409	37.29	35.43	35.89	80	8	VEST	REST2	28	50
	410	37.28	35.50	35.85	72	8	VEST	REST2	28	50
	411	37.27	36.10	35.84	81	8	VEST	REST2	28	50
	412	37.27	36.08	35.82	73	8	VEST	REST2	28	50
	413	37.29	36.23	35.82	73	8	VEST	REST2	28	50
	414	37.30	36.34	35.85	78	8	VEST	REST2	28	50
	415	37.29	36.46	35.86	78	8	VEST	REST2	28	50
	416	37.29	36.59	35.84	78	8	VEST	REST2	28	50
	417	37.30	36.65	35.88	84	8	VEST	REST2	28	50
	418	37.30	36.59	35.95	84	8	VEST	REST2	28	50
	419	37.31	36.59	35.97	75	8	VEST	REST2	28	50
	420	37.30	36.66	35.97	77	8	VEST	REST2	28	50
70	421	37.30	36.71	35.99	84	8	VEST	REST2	28	50
	422	37.29	36.71	35.99	83	8	VEST	REST2	28	50
	423	37.28	36.73	35.98	81	8	VEST	REST2	28	50
	424	37.28	36.79	35.98	80	8	VEST	REST2	28	50
	425	37.28	36.81	35.98	71	8	VEST	REST2	28	50
	426	37.28	36.83	35.99	79	8	VEST	REST2	28	50
	427	37.28	36.85	35.99	73	8	VEST	REST2	28	50
	428	37.28	36.86	35.95	69	8	VEST	REST2	28	50
	429	37.28	36.86	35.94	79	8	VEST	REST2	28	50
	430	37.28	36.87	35.91	80	8	VEST	REST2	28	50
	431	37.27	36.88	35.69	71	8	VEST	REST2	28	50
	432	37.27	36.88	35.60	70	8	VEST	REST2	28	50

min	number	Tre	Tes	Tsk-head	HR	ID	condition	period	Ta	RH
	433	37.27	36.88	35.64	68	8	VEST	REST2	28	50
	434	37.27	36.89	35.57	81	8	VEST	REST2	28	50
	435	37.28	36.91	35.54	88	8	VEST	REST2	28	50
	436	37.29	36.92	35.55	86	8	VEST	REST2	28	50
	437	37.28	36.92	35.57	85	8	VEST	REST2	28	50
	438	37.27	36.93	35.58	83	8	VEST	REST2	28	50
	439	37.25	36.89	35.59	78	8	VEST	REST2	40	50
	440	37.26	36.87	35.67	91	8	VEST	REST2	40	50
	441	37.27	36.88	35.81	81	8	VEST	REST2	40	50
	442	37.27	36.89	35.93	89	8	VEST	REST2	40	50
	443	37.28	36.90	36.02	83	8	VEST	REST2	40	50
	444	37.28	36.91	36.08	75	8	VEST	REST2	40	50
	445	37.28	36.90	36.15	72	8	VEST	REST2	40	50
	446	37.28	36.89	36.23	70	8	VEST	REST2	40	50
	447	37.27	36.90	36.28	74	8	VEST	REST2	40	50
	448	37.27	36.92	36.30	72	8	VEST	REST2	40	50
	449	37.27	36.92	36.31	68	8	VEST	REST2	40	50
	450	37.27	36.90	36.34	70	8	VEST	REST2	40	50
75	451	37.27	36.89	36.37	75	8	VEST	REST2	40	50
	452	37.26	36.90	36.37	73	8	VEST	REST2	40	50
	453	37.26	36.91	36.39	77	8	VEST	REST2	40	50
	454	37.26	36.90	36.41	73	8	VEST	REST2	40	50
	455	37.26	36.90	36.43	74	8	VEST	REST2	40	50
	456	37.26	36.89	36.45	80	8	VEST	REST2	40	50
	457	37.26	36.88	36.44	75	8	VEST	REST2	40	50
	458	37.27	36.86	36.44	97	8	VEST	REST2	40	50
	459	37.27	36.85	36.45	76	8	VEST	REST2	40	50
	460	37.27	36.87	36.48	82	8	VEST	REST2	40	50
	461	37.27	36.88	36.47	83	8	VEST	REST2	40	50
	462	37.29	36.88	36.48	76	8	VEST	REST2	40	50
	463	37.29	36.89	36.51	76	8	VEST	EXERCISE2	40	50
	464	37.28	36.90	36.50	75	8	VEST	EXERCISE2	40	50
	465	37.28	36.90	36.49	87	8	VEST	EXERCISE2	40	50
	466	37.28	36.68	36.50	93	8	VEST	EXERCISE2	40	50
	467	37.27	36.64	36.50	100	8	VEST	EXERCISE2	40	50
	468	37.25	36.85	36.51	97	8	VEST	EXERCISE2	40	50
	469	37.25	36.88	36.53	97	8	VEST	EXERCISE2	40	50
	470	37.25	36.88	36.56	101	8	VEST	EXERCISE2	40	50
	471	37.25	36.88	36.58	95	8	VEST	EXERCISE2	40	50
	472	37.27	36.90	36.60	97	8	VEST	EXERCISE2	40	50
	473	37.28	36.91	36.61	97	8	VEST	EXERCISE2	40	50
	474	37.28	36.94	36.63	102	8	VEST	EXERCISE2	40	50
	475	37.28	36.95	36.64	105	8	VEST	EXERCISE2	40	50
	476	37.28	36.94	36.65	104	8	VEST	EXERCISE2	40	50
	477	37.28	36.96	36.66	102	8	VEST	EXERCISE2	40	50
	478	37.29	36.96	36.66	101	8	VEST	EXERCISE2	40	50

min	number	Tre	Tes	Tsk-head	HR	ID	condition	period	Ta	RH
	479	37.29	36.97	36.66	100	8	VEST	EXERCISE2	40	50
	480	37.29	37.00	36.68	102	8	VEST	EXERCISE2	40	50
80	481	37.30	37.00	36.70	102	8	VEST	EXERCISE2	40	50
	482	37.30	37.02	36.72	104	8	VEST	EXERCISE2	40	50
	483	37.30	37.02	36.75	101	8	VEST	EXERCISE2	40	50
	484	37.31	37.03	36.76	101	8	VEST	EXERCISE2	40	50
	485	37.32	37.05	36.78	101	8	VEST	EXERCISE2	40	50
	486	37.31	37.06	36.81	103	8	VEST	EXERCISE2	40	50
	487	37.30	37.08	36.83	103	8	VEST	EXERCISE2	40	50
	488	37.30	37.08	36.86	106	8	VEST	EXERCISE2	40	50
	489	37.30	37.06	36.87	102	8	VEST	EXERCISE2	40	50
	490	37.30	37.06	36.87	105	8	VEST	EXERCISE2	40	50
	491	37.30	37.07	36.88	105	8	VEST	EXERCISE2	40	50
	492	37.29	37.07	36.90	106	8	VEST	EXERCISE2	40	50
	493	37.30	37.08	36.93	105	8	VEST	EXERCISE2	40	50
	494	37.31	37.09	36.95	109	8	VEST	EXERCISE2	40	50
	495	37.31	37.10	36.96	107	8	VEST	EXERCISE2	40	50
	496	37.32	37.10	36.97	107	8	VEST	EXERCISE2	40	50
	497	37.32	37.08	36.98	105	8	VEST	EXERCISE2	40	50
	498	37.32	37.08	36.99	106	8	VEST	EXERCISE2	40	50
	499	37.33	37.11	37.00	106	8	VEST	EXERCISE2	40	50
	500	37.32	37.11	36.99	108	8	VEST	EXERCISE2	40	50
	501	37.32	37.10	36.99	106	8	VEST	EXERCISE2	40	50
	502	37.32	37.09	36.98	105	8	VEST	EXERCISE2	40	50
	503	37.32	37.08	36.95	104	8	VEST	EXERCISE2	40	50
	504	37.33	37.09	36.94	106	8	VEST	EXERCISE2	40	50
	505	37.33	37.10	36.96	109	8	VEST	EXERCISE2	40	50
	506	37.33	37.08	36.96	110	8	VEST	EXERCISE2	40	50
	507	37.33	37.05	36.95	108	8	VEST	EXERCISE2	40	50
	508	37.33	37.04	36.94	111	8	VEST	EXERCISE2	40	50
	509	37.32	37.07	36.96	111	8	VEST	EXERCISE2	40	50
	510	37.32	37.05	36.95	112	8	VEST	EXERCISE2	40	50
85	511	37.33	37.04	36.93	111	8	VEST	EXERCISE2	40	50
	512	37.34	37.06	36.93	110	8	VEST	EXERCISE2	40	50
	513	37.34	37.07	36.92	112	8	VEST	EXERCISE2	40	50
	514	37.35	37.09	36.94	111	8	VEST	EXERCISE2	40	50
	515	37.35	37.08	36.94	113	8	VEST	EXERCISE2	40	50
	516	37.35	37.05	36.93	116	8	VEST	EXERCISE2	40	50
	517	37.34	37.05	36.95	115	8	VEST	EXERCISE2	40	50
	518	37.34	36.92	36.93	116	8	VEST	EXERCISE2	40	50
	519	37.34	36.87	36.89	112	8	VEST	EXERCISE2	40	50
	520	37.34	36.98	36.88	110	8	VEST	EXERCISE2	40	50
	521	37.35	36.99	36.88	109	8	VEST	EXERCISE2	40	50
	522	37.35	37.01	36.88	108	8	VEST	EXERCISE2	40	50
	523	37.35	37.05	36.89	114	8	VEST	EXERCISE2	40	50
	524	37.36	37.03	36.90	114	8	VEST	EXERCISE2	40	50

min	number	Tre	Tes	Tsk-head	HR	ID	condition	period	Ta	RH
	525	37.36	37.01	36.89	110	8	VEST	EXERCISE2	40	50
	526	37.35	37.05	36.89	107	8	VEST	EXERCISE2	40	50
	527	37.36	37.08	36.91	114	8	VEST	EXERCISE2	40	50
	528	37.36	37.06	36.89	115	8	VEST	EXERCISE2	40	50
	529	37.36	37.04	36.89	115	8	VEST	EXERCISE2	40	50
	530	37.37	37.07	36.89	116	8	VEST	EXERCISE2	40	50
	531	37.36	37.06	36.85	115	8	VEST	EXERCISE2	40	50
	532	37.36	36.98	36.83	118	8	VEST	EXERCISE2	40	50
	533	37.37	36.97	36.83	116	8	VEST	EXERCISE2	40	50
	534	37.37	37.05	36.83	115	8	VEST	EXERCISE2	40	50
	535	37.37	37.06	36.85	115	8	VEST	EXERCISE2	40	50
	536	37.38	37.02	36.85	114	8	VEST	EXERCISE2	40	50
	537	37.38	37.02	36.86	113	8	VEST	EXERCISE2	40	50
	538	37.38	37.06	36.88	115	8	VEST	EXERCISE2	40	50
	539	37.38	37.05	36.86	117	8	VEST	EXERCISE2	40	50
	540	37.38	37.02	36.88	118	8	VEST	EXERCISE2	40	50
90	541	37.38	36.99	36.88	115	8	VEST	EXERCISE2	40	50
	542	37.38	37.02	36.84	113	8	VEST	EXERCISE2	40	50
	543	37.38	37.05	36.83	114	8	VEST	EXERCISE2	40	50
	544	37.38	37.08	36.84	117	8	VEST	EXERCISE2	40	50
	545	37.38	37.08	36.87	116	8	VEST	EXERCISE2	40	50
	546	37.39	37.07	36.87	115	8	VEST	EXERCISE2	40	50
	547	37.39	37.07	36.83	119	8	VEST	EXERCISE2	40	50
	548	37.38	37.07	36.83	117	8	VEST	EXERCISE2	40	50
	549	37.38	37.09	36.84	118	8	VEST	EXERCISE2	40	50
	550	37.38	37.10	36.84	118	8	VEST	EXERCISE2	40	50
	551	37.38	37.08	36.85	123	8	VEST	EXERCISE2	40	50
	552	37.38	37.01	36.84	122	8	VEST	EXERCISE2	40	50
	553	37.38	37.03	36.84	118	8	VEST	EXERCISE2	40	50
	554	37.38	37.09	36.85	121	8	VEST	EXERCISE2	40	50
	555	37.38	37.01	36.83	119	8	VEST	EXERCISE2	40	50
	556	37.39	37.00	36.82	121	8	VEST	EXERCISE2	40	50
	557	37.39	37.08	36.85	122	8	VEST	EXERCISE2	40	50
	558	37.39	37.07	36.89	122	8	VEST	EXERCISE2	40	50
	559	37.39	37.09	36.90	123	8	VEST	EXERCISE2	40	50
	560	37.39	37.12	36.88	123	8	VEST	EXERCISE2	40	50
	561	37.40	37.11	36.86	122	8	VEST	EXERCISE2	40	50
	562	37.40	37.09	36.84	122	8	VEST	EXERCISE2	40	50
	563	37.40	37.09	36.82	123	8	VEST	EXERCISE2	40	50
	564	37.40	37.09	36.82	122	8	VEST	EXERCISE2	40	50
	565	37.40	37.11	36.83	123	8	VEST	EXERCISE2	40	50
	566	37.40	37.12	36.86	123	8	VEST	EXERCISE2	40	50
	567	37.40	37.14	36.87	124	8	VEST	EXERCISE2	40	50
	568	37.40	37.13	36.87	121	8	VEST	EXERCISE2	40	50
	569	37.40	37.12	36.86	119	8	VEST	EXERCISE2	40	50
	570	37.40	37.08	36.84	125	8	VEST	EXERCISE2	40	50

min	number	Tre	Tes	Tsk-head	HR	ID	condition	period	Ta	RH
95	571	37.41	37.06	36.85	121	8	VEST	EXERCISE2	40	50
	572	37.41	37.10	36.87	120	8	VEST	EXERCISE2	40	50
	573	37.42	37.11	36.87	121	8	VEST	EXERCISE2	40	50
	574	37.41	37.15	36.88	120	8	VEST	EXERCISE2	40	50
	575	37.42	37.11	36.90	125	8	VEST	EXERCISE2	40	50
	576	37.42	37.08	36.92	124	8	VEST	EXERCISE2	40	50
	577	37.41	37.09	36.93	123	8	VEST	EXERCISE2	40	50
	578	37.42	37.09	36.93	121	8	VEST	EXERCISE2	40	50
	579	37.42	37.10	36.91	121	8	VEST	EXERCISE2	40	50
	580	37.42	37.09	36.90	122	8	VEST	EXERCISE2	40	50
	581	37.43	37.10	36.91	121	8	VEST	EXERCISE2	40	50
	582	37.44	37.11	36.91	118	8	VEST	EXERCISE2	40	50
	583	37.43	37.12	36.90	123	8	VEST	EXERCISE2	40	50
	584	37.43	37.13	36.92	124	8	VEST	EXERCISE2	40	50
	585	37.43	37.13	36.95	120	8	VEST	EXERCISE2	40	50
	586	37.43	37.01	36.95	123	8	VEST	EXERCISE2	40	50
	587	37.44	36.99	36.94	121	8	VEST	EXERCISE2	40	50
	588	37.44	37.12	36.95	120	8	VEST	EXERCISE2	40	50
	589	37.44	37.14	36.96	123	8	VEST	EXERCISE2	40	50
	590	37.44	37.14	36.97	125	8	VEST	EXERCISE2	40	50
	591	37.44	37.19	36.97	126	8	VEST	EXERCISE2	40	50
	592	37.44	37.18	36.96	127	8	VEST	EXERCISE2	40	50
	593	37.44	37.09	36.97	125	8	VEST	EXERCISE2	40	50
	594	37.44	37.09	36.96	126	8	VEST	EXERCISE2	40	50
	595	37.45	37.10	36.96	120	8	VEST	EXERCISE2	40	50
	596	37.45	37.11	36.97	125	8	VEST	EXERCISE2	40	50
	597	37.46	37.13	36.98	123	8	VEST	EXERCISE2	40	50
	598	37.46	37.14	36.99	121	8	VEST	EXERCISE2	40	50
	599	37.46	37.13	36.98	123	8	VEST	EXERCISE2	40	50
	600	37.46	37.13	36.97	120	8	VEST	EXERCISE2	40	50
100	601	37.47	37.15	36.96	122	8	VEST	EXERCISE2	40	50
	602	37.48	37.14	36.95	126	8	VEST	EXERCISE2	40	50
	603	37.48	37.14	36.96	123	8	VEST	EXERCISE2	40	50
	604	37.48	37.17	36.99	123	8	VEST	EXERCISE2	40	50
	605	37.49	37.16	37.01	126	8	VEST	EXERCISE2	40	50
	606	37.49	37.14	36.98	122	8	VEST	EXERCISE2	40	50
	607	37.49	37.12	36.97	124	8	VEST	EXERCISE2	40	50
	608	37.50	37.08	36.97	119	8	VEST	EXERCISE2	40	50
	609	37.50	37.10	36.97	117	8	VEST	EXERCISE2	40	50
	610	37.50	37.12	36.98	120	8	VEST	EXERCISE2	40	50
	611	37.51	37.13	36.99	118	8	VEST	EXERCISE2	40	50
	612	37.50	37.15	36.99	118	8	VEST	EXERCISE2	40	50
	613	37.50	37.14	36.97	116	8	VEST	EXERCISE2	40	50
	614	37.50	37.13	36.98	121	8	VEST	EXERCISE2	40	50
	615	37.50	37.13	36.99	120	8	VEST	EXERCISE2	40	50
	616	37.50	37.13	36.97	122	8	VEST	EXERCISE2	40	50

min	number	Tre	Tes	Tsk-head	HR	ID	condition	period	Ta	RH
	617	37.50	37.12	36.96	121	8	VEST	EXERCISE2	40	50
	618	37.50	37.12	36.96	122	8	VEST	EXERCISE2	40	50
	619	37.51	37.14	36.96	123	8	VEST	EXERCISE2	40	50
	620	37.51	37.14	36.95	125	8	VEST	EXERCISE2	40	50
	621	37.52	37.15	36.95	125	8	VEST	EXERCISE2	40	50
	622	37.52	37.19	36.96	126	8	VEST	EXERCISE2	40	50
	623	37.52	37.21	36.98	125	8	VEST	EXERCISE2	40	50
	624	37.51	37.20	36.98	123	8	VEST	EXERCISE2	40	50
	625	37.51	37.20	36.98	123	8	VEST	EXERCISE2	40	50
	626	37.51	37.21	36.97	123	8	VEST	EXERCISE2	40	50
	627	37.50	37.16	36.96	126	8	VEST	EXERCISE2	40	50
	628	37.51	37.16	36.97	127	8	VEST	EXERCISE2	40	50
	629	37.52	37.16	36.97	127	8	VEST	EXERCISE2	40	50
	630	37.52	37.14	36.97	125	8	VEST	EXERCISE2	40	50
105	631	37.53	37.19	36.99	123	8	VEST	EXERCISE2	40	50
	632	37.54	37.14	37.00	123	8	VEST	EXERCISE2	40	50
	633	37.53	37.11	36.99	121	8	VEST	EXERCISE2	40	50
	634	37.53	37.17	36.99	125	8	VEST	EXERCISE2	40	50
	635	37.54	37.19	36.99	126	8	VEST	EXERCISE2	40	50
	636	37.54	37.15	36.98	126	8	VEST	EXERCISE2	40	50
	637	37.54	37.13	36.96	123	8	VEST	EXERCISE2	40	50
	638	37.54	37.16	36.95	125	8	VEST	EXERCISE2	40	50
	639	37.54	37.19	36.96	123	8	VEST	EXERCISE2	40	50
	640	37.54	37.19	36.97	123	8	VEST	EXERCISE2	40	50
	641	37.55	37.18	36.98	123	8	VEST	EXERCISE2	40	50
	642	37.55	37.13	37.01	128	8	VEST	EXERCISE2	40	50
	643	37.55	37.12	37.00	125	8	VEST	EXERCISE2	40	50
	644	37.55	37.18	36.98	123	8	VEST	REST3	28	50
	645	37.55	37.16	36.89	130	8	VEST	REST3	28	50
	646	37.56	37.18	36.80	126	8	VEST	REST3	28	50
	647	37.55	37.21	36.75	120	8	VEST	REST3	28	50
	648	37.55	37.22	36.67	117	8	VEST	REST3	28	50
	649	37.55	37.18	36.58	122	8	VEST	REST3	28	50
	650	37.54	37.15	36.59	117	8	VEST	REST3	28	50
	651	37.53	37.18	36.59	114	8	VEST	REST3	28	50
	652	37.52	37.17	36.58	96	8	VEST	REST3	28	50
	653	37.51	37.18	36.68	95	8	VEST	REST3	28	50
	654	37.51	37.19	36.72	95	8	VEST	REST3	28	50
	655	37.53	37.10	36.69	94	8	VEST	REST3	28	50
	656	37.53	37.06	36.72	90	8	VEST	REST3	28	50
	657	37.52	37.10	36.73	86	8	VEST	REST3	28	50
	658	37.52	37.07	36.73	86	8	VEST	REST3	28	50
	659	37.53	37.04	36.73	88	8	VEST	REST3	28	50
	660	37.53	37.01	36.72	87	8	VEST	REST3	28	50
110	661	37.53	36.98	36.69	85	8	VEST	REST3	28	50
	662	37.52	36.96	36.67	85	8	VEST	REST3	28	50

min	number	Tre	Tes	Tsk-head	HR	ID	condition	period	Ta	RH
	663	37.52	36.96	36.66	85	8	VEST	REST3	28	50
	664	37.51	36.97	36.67	86	8	VEST	REST3	28	50
	665	37.51	36.95	36.69	84	8	VEST	REST3	28	50
	666	37.51	36.94	36.67	83	8	VEST	REST3	28	50
	667	37.51	36.93	36.64	84	8	VEST	REST3	28	50
	668	37.51	36.93	36.63	91	8	VEST	REST3	28	50
	669	37.50	36.90	36.62	89	8	VEST	REST3	28	50
	670	37.50	36.91	36.60	81	8	VEST	REST3	28	50
	671	37.49	36.95	36.58	82	8	VEST	REST3	28	50
	672	37.50	36.95	36.56	81	8	VEST	REST3	28	50
	673	37.50	36.96	36.54	78	8	VEST	REST3	28	50
	674	37.50	36.96	36.52	76	8	VEST	REST3	28	50
	675	37.50	36.94	36.50	86	8	VEST	REST3	28	50
	676	37.49	36.93	36.49	89	8	VEST	REST3	28	50
	677	37.49	36.90	36.48	85	8	VEST	REST3	28	50
	678	37.49	36.90	36.46	78	8	VEST	REST3	28	50
	679	37.50	36.95	36.43	77	8	VEST	REST3	28	50
	680	37.50	36.96	36.36	75	8	VEST	REST3	28	50
	681	37.50	36.93	36.11	84	8	VEST	REST3	28	50
	682	37.50	36.94	35.82	84	8	VEST	REST3	28	50
	683	37.50	36.92	35.65	86	8	VEST	REST3	28	50
	684	37.50	36.91	35.55	78	8	VEST	REST3	28	50
	685	37.50	36.94	35.50	88	8	VEST	REST3	28	50
	686	37.50	36.96	35.48	86	8	VEST	REST3	28	50
	687	37.50	36.99	35.47	80	8	VEST	REST3	28	50
	688	37.50	37.00	35.45	82	8	VEST	REST3	28	50
	689	37.50	36.94	35.47	81	8	VEST	REST3	28	50
	690	37.50	36.93	35.53	75	8	VEST	REST3	28	50
115	691	37.50	36.99	35.56	82	8	VEST	REST3	28	50
	692	37.50	37.02	35.52	86	8	VEST	REST3	28	50
	693	37.49	37.05	35.52	80	8	VEST	REST3	28	50
	694	37.48	37.07	35.55	79	8	VEST	REST3	28	50
	695	37.49	37.07	35.58	95	8	VEST	REST3	28	50
	696	37.49	37.08	35.60	80	8	VEST	REST3	28	50
	697	37.49	37.08	35.56	74	8	VEST	REST3	28	50
	698	37.48	37.08	35.53	74	8	VEST	REST3	28	50
	699	37.47	37.09	35.52	74	8	VEST	REST3	28	50
	700	37.47	37.10	35.52	80	8	VEST	REST3	28	50
	701	37.47	37.12	35.47	75	8	VEST	REST3	28	50
	702	37.48	37.13	35.46	81	8	VEST	REST3	28	50
	703	37.48	37.13	35.50	81	8	VEST	REST3	28	50
	704	37.48	37.13	35.54	80	8	VEST	REST3	28	50
	705	37.49	37.11	35.58	79	8	VEST	REST3	28	50
	706	37.49	37.10	35.56	85	8	VEST	REST3	28	50
	707	37.49	37.12	35.52	81	8	VEST	REST3	28	50
	708	37.49	37.16	35.53	82	8	VEST	REST3	28	50

min	number	Tre	Tes	Tsk-head	HR	ID	condition	period	Ta	RH
	709	37.48	37.17	35.53	80	8	VEST	REST3	28	50
0	1	36.77	36.28	35.15	78	9	VEST	REST1	28	50
	2	36.77	36.29	35.14	80	9	VEST	REST1	28	50
	3	36.77	36.28	35.15	76	9	VEST	REST1	28	50
	4	36.78	36.27	35.16	90	9	VEST	REST1	28	50
	5	36.77	36.28	35.16	80	9	VEST	REST1	28	50
	6	36.76	36.27	35.14	76	9	VEST	REST1	28	50
	7	36.76	36.26	35.10	79	9	VEST	REST1	28	50
	8	36.77	36.27	35.10	73	9	VEST	REST1	28	50
	9	36.77	36.28	35.13	71	9	VEST	REST1	28	50
	10	36.78	36.25	35.12	74	9	VEST	REST1	28	50
	11	36.78	36.24	35.11	70	9	VEST	REST1	28	50
	12	36.78	36.27	35.10	72	9	VEST	REST1	28	50
	13	36.77	36.27	35.10	72	9	VEST	REST1	28	50
	14	36.76	36.22	35.10	77	9	VEST	REST1	28	50
	15	36.74	36.20	35.10	77	9	VEST	REST1	28	50
	16	36.72	36.24	35.08	72	9	VEST	REST1	28	50
	17	36.70	36.25	35.06	76	9	VEST	REST1	28	50
	18	36.69	36.24	35.07	78	9	VEST	REST1	28	50
	19	36.68	36.23	35.09	74	9	VEST	REST1	28	50
	20	36.68	36.24	35.12	77	9	VEST	REST1	28	50
	21	36.68	36.24	35.12	75	9	VEST	REST1	28	50
	22	36.67	36.24	35.09	75	9	VEST	REST1	28	50
	23	36.67	36.24	35.07	75	9	VEST	REST1	28	50
	24	36.68	36.24	35.10	70	9	VEST	REST1	28	50
	25	36.69	36.25	35.14	74	9	VEST	REST1	28	50
	26	36.68	36.25	35.16	70	9	VEST	REST1	28	50
	27	36.68	36.21	35.15	78	9	VEST	REST1	28	50
	28	36.68	36.19	35.14	75	9	VEST	REST1	28	50
	29	36.67	36.21	35.16	72	9	VEST	REST1	28	50
	30	36.67	36.21	35.17	74	9	VEST	REST1	28	50
5	31	36.67	36.20	35.16	87	9	VEST	REST1	28	50
	32	36.66	36.20	35.17	97	9	VEST	REST1	28	50
	33	36.65	36.21	35.18	75	9	VEST	REST1	28	50
	34	36.65	36.20	35.17	74	9	VEST	REST1	28	50
	35	36.66	36.20	35.16	73	9	VEST	REST1	28	50
	36	36.66	36.20	35.15	69	9	VEST	REST1	28	50
	37	36.66	36.20	35.13	72	9	VEST	REST1	28	50
	38	36.66	36.20	35.15	72	9	VEST	REST1	28	50
	39	36.66	36.21	35.17	72	9	VEST	REST1	28	50
	40	36.66	36.21	35.17	72	9	VEST	REST1	28	50
	41	36.65	36.21	35.15	78	9	VEST	REST1	28	50
	42	36.64	36.18	35.15	72	9	VEST	REST1	28	50
	43	36.65	36.17	35.17	70	9	VEST	REST1	28	50
	44	36.65	36.19	35.16	71	9	VEST	REST1	28	50
	45	36.65	36.19	35.13	72	9	VEST	REST1	28	50

min	number	Tre	Tes	Tsk-head	HR	ID	condition	period	Ta	RH
	46	36.64	36.18	35.12	73	9	VEST	REST1	28	50
	47	36.64	36.18	35.11	72	9	VEST	REST1	28	50
	48	36.65	36.20	35.12	78	9	VEST	REST1	28	50
	49	36.65	36.19	35.15	71	9	VEST	REST1	28	50
	50	36.65	36.17	35.16	75	9	VEST	REST1	28	50
	51	36.65	36.19	35.17	75	9	VEST	REST1	28	50
	52	36.65	36.20	35.20	74	9	VEST	REST1	28	50
	53	36.64	36.19	35.20	74	9	VEST	REST1	28	50
	54	36.63	36.18	35.20	74	9	VEST	REST1	28	50
	55	36.65	36.18	35.18	73	9	VEST	REST1	28	50
	56	36.65	36.17	35.17	73	9	VEST	REST1	28	50
	57	36.65	36.17	35.20	72	9	VEST	REST1	28	50
	58	36.66	36.18	35.22	73	9	VEST	REST1	28	50
	59	36.66	36.18	35.19	70	9	VEST	REST1	28	50
	60	36.65	36.16	35.17	71	9	VEST	REST1	28	50
10	61	36.63	36.16	35.16	70	9	VEST	REST1	28	50
	62	36.64	36.17	35.14	72	9	VEST	REST1	28	50
	63	36.64	36.17	35.15	75	9	VEST	REST1	28	50
	64	36.62	36.17	35.15	67	9	VEST	REST1	28	50
	65	36.62	36.17	35.17	72	9	VEST	REST1	28	50
	66	36.61	36.16	35.17	75	9	VEST	REST1	28	50
	67	36.61	36.16	35.16	67	9	VEST	REST1	28	50
	68	36.61	36.17	35.15	70	9	VEST	REST1	28	50
	69	36.61	36.17	35.16	68	9	VEST	REST1	28	50
	70	36.61	36.18	35.19	73	9	VEST	REST1	28	50
	71	36.61	36.17	35.17	74	9	VEST	REST1	28	50
	72	36.61	36.18	35.14	74	9	VEST	REST1	28	50
	73	36.62	36.19	35.13	79	9	VEST	REST1	28	50
	74	36.63	36.16	35.16	85	9	VEST	REST1	28	50
	75	36.64	36.12	35.20	86	9	VEST	REST1	28	50
	76	36.64	36.12	35.20	78	9	VEST	REST1	28	50
	77	36.64	36.14	35.19	74	9	VEST	REST1	28	50
	78	36.64	36.16	35.19	76	9	VEST	REST1	28	50
	79	36.64	36.15	35.20	71	9	VEST	REST1	28	50
	80	36.63	36.15	35.22	73	9	VEST	REST1	28	50
	81	36.63	36.16	35.22	79	9	VEST	REST1	28	50
	82	36.62	36.16	35.18	71	9	VEST	REST1	28	50
	83	36.61	36.15	35.16	71	9	VEST	REST1	28	50
	84	36.61	36.15	35.13	76	9	VEST	REST1	28	50
	85	36.62	36.16	35.11	82	9	VEST	REST1	28	50
	86	36.63	36.17	35.12	74	9	VEST	REST1	28	50
	87	36.63	36.18	35.11	75	9	VEST	REST1	28	50
	88	36.63	36.18	35.13	82	9	VEST	REST1	28	50
	89	36.63	36.15	35.13	75	9	VEST	REST1	28	50
	90	36.62	36.12	35.10	75	9	VEST	REST1	28	50
15	91	36.61	36.14	35.12	72	9	VEST	REST1	28	50

min	number	Tre	Tes	Tsk-head	HR	ID	condition	period	Ta	RH
	92	36.61	36.15	35.13	70	9	VEST	REST1	28	50
	93	36.62	36.16	35.14	73	9	VEST	REST1	28	50
	94	36.62	36.16	35.17	71	9	VEST	REST1	28	50
	95	36.61	36.16	35.18	72	9	VEST	REST1	28	50
	96	36.60	36.17	35.17	75	9	VEST	REST1	28	50
	97	36.59	36.17	35.14	72	9	VEST	REST1	28	50
	98	36.59	36.17	35.12	70	9	VEST	REST1	28	50
	99	36.60	36.17	35.11	76	9	VEST	REST1	28	50
	100	36.60	36.15	35.09	78	9	VEST	REST1	28	50
	101	36.61	36.12	35.07	81	9	VEST	REST1	28	50
	102	36.61	36.10	35.06	79	9	VEST	REST1	28	50
	103	36.61	36.12	35.09	71	9	VEST	REST1	40	50
	104	36.62	36.14	35.10	81	9	VEST	REST1	40	50
	105	36.62	36.15	35.21	103	9	VEST	REST1	40	50
	106	36.62	36.14	35.40	93	9	VEST	REST1	40	50
	107	36.61	36.11	35.48	90	9	VEST	REST1	40	50
	108	36.61	36.07	35.56	82	9	VEST	REST1	40	50
	109	36.62	36.02	35.63	83	9	VEST	REST1	40	50
	110	36.62	36.04	35.69	81	9	VEST	REST1	40	50
	111	36.62	36.04	35.74	89	9	VEST	REST1	40	50
	112	36.61	36.00	35.78	83	9	VEST	REST1	40	50
	113	36.59	35.99	35.80	80	9	VEST	REST1	40	50
	114	36.59	36.01	35.83	78	9	VEST	REST1	40	50
	115	36.59	36.13	35.87	75	9	VEST	REST1	40	50
	116	36.58	36.13	35.88	80	9	VEST	REST1	40	50
	117	36.58	36.03	35.87	84	9	VEST	REST1	40	50
	118	36.59	36.01	35.88	72	9	VEST	REST1	40	50
	119	36.61	36.02	35.92	65	9	VEST	REST1	40	50
	120	36.62	36.05	35.93	66	9	VEST	REST1	40	50
20	121	36.63	36.07	35.95	67	9	VEST	REST1	40	50
	122	36.63	36.09	35.96	66	9	VEST	REST1	40	50
	123	36.63	36.09	35.97	65	9	VEST	REST1	40	50
	124	36.63	36.07	35.98	74	9	VEST	REST1	40	50
	125	36.62	36.06	35.98	69	9	VEST	REST1	40	50
	126	36.61	36.08	35.99	71	9	VEST	REST1	40	50
	127	36.62	36.11	36.01	71	9	VEST	REST1	40	50
	128	36.61	36.12	36.02	74	9	VEST	REST1	40	50
	129	36.60	36.12	36.04	76	9	VEST	REST1	40	50
	130	36.59	36.12	36.06	70	9	VEST	REST1	40	50
	131	36.59	36.14	36.07	71	9	VEST	REST1	40	50
	132	36.59	36.14	36.10	71	9	VEST	REST1	40	50
	133	36.59	36.13	36.13	77	9	VEST	REST1	40	50
	134	36.60	36.15	36.16	91	9	VEST	REST1	40	50
	135	36.60	36.17	36.18	99	9	VEST	REST1	40	50
	136	36.60	36.16	36.19	83	9	VEST	REST1	40	50
	137	36.59	36.17	36.20	78	9	VEST	REST1	40	50

min	number	Tre	Tes	Tsk-head	HR	ID	condition	period	Ta	RH
	138	36.58	36.18	36.21	70	9	VEST	REST1	40	50
	139	36.58	36.17	36.20	73	9	VEST	EXERCISE1	40	50
	140	36.59	36.17	36.21	88	9	VEST	EXERCISE1	40	50
	141	36.60	36.19	36.24	91	9	VEST	EXERCISE1	40	50
	142	36.60	36.20	36.26	101	9	VEST	EXERCISE1	40	50
	143	36.60	36.18	36.28	105	9	VEST	EXERCISE1	40	50
	144	36.60	36.19	36.30	107	9	VEST	EXERCISE1	40	50
	145	36.60	36.17	36.30	108	9	VEST	EXERCISE1	40	50
	146	36.60	36.15	36.29	109	9	VEST	EXERCISE1	40	50
	147	36.60	36.17	36.31	106	9	VEST	EXERCISE1	40	50
	148	36.60	36.17	36.32	108	9	VEST	EXERCISE1	40	50
	149	36.59	36.17	36.31	108	9	VEST	EXERCISE1	40	50
	150	36.59	36.19	36.31	109	9	VEST	EXERCISE1	40	50
25	151	36.59	36.21	36.32	107	9	VEST	EXERCISE1	40	50
	152	36.59	36.21	36.32	110	9	VEST	EXERCISE1	40	50
	153	36.59	36.23	36.33	111	9	VEST	EXERCISE1	40	50
	154	36.59	36.23	36.34	113	9	VEST	EXERCISE1	40	50
	155	36.59	36.24	36.35	108	9	VEST	EXERCISE1	40	50
	156	36.59	36.27	36.36	105	9	VEST	EXERCISE1	40	50
	157	36.59	36.28	36.37	107	9	VEST	EXERCISE1	40	50
	158	36.58	36.30	36.38	108	9	VEST	EXERCISE1	40	50
	159	36.58	36.32	36.39	107	9	VEST	EXERCISE1	40	50
	160	36.57	36.32	36.40	108	9	VEST	EXERCISE1	40	50
	161	36.58	36.34	36.42	108	9	VEST	EXERCISE1	40	50
	162	36.58	36.36	36.45	109	9	VEST	EXERCISE1	40	50
	163	36.58	36.37	36.46	111	9	VEST	EXERCISE1	40	50
	164	36.58	36.35	36.46	112	9	VEST	EXERCISE1	40	50
	165	36.58	36.35	36.47	112	9	VEST	EXERCISE1	40	50
	166	36.59	36.39	36.48	113	9	VEST	EXERCISE1	40	50
	167	36.59	36.41	36.49	112	9	VEST	EXERCISE1	40	50
	168	36.59	36.41	36.50	108	9	VEST	EXERCISE1	40	50
	169	36.59	36.41	36.52	110	9	VEST	EXERCISE1	40	50
	170	36.59	36.40	36.53	113	9	VEST	EXERCISE1	40	50
	171	36.59	36.39	36.51	113	9	VEST	EXERCISE1	40	50
	172	36.59	36.40	36.52	110	9	VEST	EXERCISE1	40	50
	173	36.59	36.37	36.53	112	9	VEST	EXERCISE1	40	50
	174	36.60	36.36	36.53	111	9	VEST	EXERCISE1	40	50
	175	36.60	36.39	36.54	112	9	VEST	EXERCISE1	40	50
	176	36.60	36.39	36.54	114	9	VEST	EXERCISE1	40	50
	177	36.61	36.40	36.54	116	9	VEST	EXERCISE1	40	50
	178	36.61	36.39	36.55	116	9	VEST	EXERCISE1	40	50
	179	36.61	36.37	36.55	113	9	VEST	EXERCISE1	40	50
	180	36.61	36.38	36.55	113	9	VEST	EXERCISE1	40	50
30	181	36.61	36.41	36.56	113	9	VEST	EXERCISE1	40	50
	182	36.61	36.40	36.56	114	9	VEST	EXERCISE1	40	50
	183	36.62	36.39	36.56	115	9	VEST	EXERCISE1	40	50

min	number	Tre	Tes	Tsk-head	HR	ID	condition	period	Ta	RH
	184	36.63	36.38	36.57	114	9	VEST	EXERCISE1	40	50
	185	36.63	36.33	36.56	114	9	VEST	EXERCISE1	40	50
	186	36.64	36.32	36.56	115	9	VEST	EXERCISE1	40	50
	187	36.65	36.38	36.58	112	9	VEST	EXERCISE1	40	50
	188	36.64	36.39	36.58	116	9	VEST	EXERCISE1	40	50
	189	36.65	36.39	36.59	113	9	VEST	EXERCISE1	40	50
	190	36.66	36.39	36.59	115	9	VEST	EXERCISE1	40	50
	191	36.65	36.40	36.59	115	9	VEST	EXERCISE1	40	50
	192	36.65	36.40	36.58	111	9	VEST	EXERCISE1	40	50
	193	36.66	36.41	36.56	117	9	VEST	EXERCISE1	40	50
	194	36.66	36.43	36.56	118	9	VEST	EXERCISE1	40	50
	195	36.66	36.41	36.58	120	9	VEST	EXERCISE1	40	50
	196	36.67	36.38	36.57	118	9	VEST	EXERCISE1	40	50
	197	36.67	36.37	36.56	122	9	VEST	EXERCISE1	40	50
	198	36.66	36.38	36.57	119	9	VEST	EXERCISE1	40	50
	199	36.67	36.40	36.58	116	9	VEST	EXERCISE1	40	50
	200	36.68	36.42	36.58	116	9	VEST	EXERCISE1	40	50
	201	36.68	36.43	36.58	117	9	VEST	EXERCISE1	40	50
	202	36.68	36.43	36.58	117	9	VEST	EXERCISE1	40	50
	203	36.69	36.44	36.59	115	9	VEST	EXERCISE1	40	50
	204	36.69	36.42	36.60	114	9	VEST	EXERCISE1	40	50
	205	36.69	36.44	36.60	118	9	VEST	EXERCISE1	40	50
	206	36.68	36.45	36.59	117	9	VEST	EXERCISE1	40	50
	207	36.69	36.45	36.60	115	9	VEST	EXERCISE1	40	50
	208	36.70	36.49	36.61	116	9	VEST	EXERCISE1	40	50
	209	36.70	36.50	36.61	118	9	VEST	EXERCISE1	40	50
	210	36.70	36.52	36.60	119	9	VEST	EXERCISE1	40	50
35	211	36.70	36.52	36.60	118	9	VEST	EXERCISE1	40	50
	212	36.70	36.51	36.60	115	9	VEST	EXERCISE1	40	50
	213	36.71	36.54	36.60	115	9	VEST	EXERCISE1	40	50
	214	36.71	36.55	36.61	118	9	VEST	EXERCISE1	40	50
	215	36.71	36.52	36.60	117	9	VEST	EXERCISE1	40	50
	216	36.72	36.45	36.60	117	9	VEST	EXERCISE1	40	50
	217	36.73	36.44	36.60	113	9	VEST	EXERCISE1	40	50
	218	36.73	36.49	36.61	114	9	VEST	EXERCISE1	40	50
	219	36.73	36.51	36.61	118	9	VEST	EXERCISE1	40	50
	220	36.72	36.53	36.62	117	9	VEST	EXERCISE1	40	50
	221	36.72	36.54	36.62	118	9	VEST	EXERCISE1	40	50
	222	36.72	36.55	36.62	120	9	VEST	EXERCISE1	40	50
	223	36.73	36.55	36.62	118	9	VEST	EXERCISE1	40	50
	224	36.73	36.54	36.62	115	9	VEST	EXERCISE1	40	50
	225	36.74	36.54	36.63	116	9	VEST	EXERCISE1	40	50
	226	36.75	36.57	36.64	117	9	VEST	EXERCISE1	40	50
	227	36.75	36.58	36.64	118	9	VEST	EXERCISE1	40	50
	228	36.75	36.59	36.65	119	9	VEST	EXERCISE1	40	50
	229	36.75	36.59	36.65	119	9	VEST	EXERCISE1	40	50

min	number	Tre	Tes	Tsk-head	HR	ID	condition	period	Ta	RH
	230	36.76	36.60	36.65	120	9	VEST	EXERCISE1	40	50
	231	36.76	36.62	36.67	121	9	VEST	EXERCISE1	40	50
	232	36.76	36.63	36.67	118	9	VEST	EXERCISE1	40	50
	233	36.76	36.62	36.66	116	9	VEST	EXERCISE1	40	50
	234	36.76	36.62	36.66	120	9	VEST	EXERCISE1	40	50
	235	36.77	36.63	36.67	120	9	VEST	EXERCISE1	40	50
	236	36.77	36.63	36.66	120	9	VEST	EXERCISE1	40	50
	237	36.77	36.64	36.66	118	9	VEST	EXERCISE1	40	50
	238	36.76	36.64	36.67	117	9	VEST	EXERCISE1	40	50
	239	36.76	36.64	36.67	117	9	VEST	EXERCISE1	40	50
	240	36.76	36.60	36.67	120	9	VEST	EXERCISE1	40	50
40	241	36.76	36.58	36.67	118	9	VEST	EXERCISE1	40	50
	242	36.77	36.54	36.66	121	9	VEST	EXERCISE1	40	50
	243	36.78	36.54	36.66	123	9	VEST	EXERCISE1	40	50
	244	36.79	36.62	36.66	122	9	VEST	EXERCISE1	40	50
	245	36.79	36.64	36.66	120	9	VEST	EXERCISE1	40	50
	246	36.80	36.64	36.65	122	9	VEST	EXERCISE1	40	50
	247	36.79	36.63	36.63	124	9	VEST	EXERCISE1	40	50
	248	36.79	36.63	36.63	123	9	VEST	EXERCISE1	40	50
	249	36.80	36.63	36.64	123	9	VEST	EXERCISE1	40	50
	250	36.80	36.65	36.64	123	9	VEST	EXERCISE1	40	50
	251	36.81	36.67	36.66	123	9	VEST	EXERCISE1	40	50
	252	36.81	36.67	36.66	120	9	VEST	EXERCISE1	40	50
	253	36.82	36.67	36.66	122	9	VEST	EXERCISE1	40	50
	254	36.82	36.69	36.67	120	9	VEST	EXERCISE1	40	50
	255	36.82	36.70	36.68	118	9	VEST	EXERCISE1	40	50
	256	36.81	36.70	36.67	118	9	VEST	EXERCISE1	40	50
	257	36.82	36.71	36.67	119	9	VEST	EXERCISE1	40	50
	258	36.83	36.72	36.68	119	9	VEST	EXERCISE1	40	50
	259	36.83	36.72	36.68	118	9	VEST	EXERCISE1	40	50
	260	36.83	36.72	36.68	120	9	VEST	EXERCISE1	40	50
	261	36.83	36.73	36.68	122	9	VEST	EXERCISE1	40	50
	262	36.83	36.73	36.69	122	9	VEST	EXERCISE1	40	50
	263	36.84	36.74	36.69	123	9	VEST	EXERCISE1	40	50
	264	36.85	36.72	36.68	125	9	VEST	EXERCISE1	40	50
	265	36.86	36.71	36.69	126	9	VEST	EXERCISE1	40	50
	266	36.87	36.74	36.70	125	9	VEST	EXERCISE1	40	50
	267	36.87	36.75	36.70	124	9	VEST	EXERCISE1	40	50
	268	36.88	36.75	36.70	127	9	VEST	EXERCISE1	40	50
	269	36.89	36.76	36.70	126	9	VEST	EXERCISE1	40	50
	270	36.89	36.77	36.71	127	9	VEST	EXERCISE1	40	50
45	271	36.89	36.77	36.72	128	9	VEST	EXERCISE1	40	50
	272	36.89	36.78	36.72	122	9	VEST	EXERCISE1	40	50
	273	36.89	36.78	36.72	119	9	VEST	EXERCISE1	40	50
	274	36.90	36.80	36.72	116	9	VEST	EXERCISE1	40	50
	275	36.92	36.81	36.72	119	9	VEST	EXERCISE1	40	50

min	number	Tre	Tes	Tsk-head	HR	ID	condition	period	Ta	RH
	276	36.92	36.82	36.73	124	9	VEST	EXERCISE1	40	50
	277	36.92	36.83	36.74	124	9	VEST	EXERCISE1	40	50
	278	36.93	36.83	36.74	122	9	VEST	EXERCISE1	40	50
	279	36.93	36.84	36.75	123	9	VEST	EXERCISE1	40	50
	280	36.94	36.85	36.76	123	9	VEST	EXERCISE1	40	50
	281	36.95	36.86	36.77	123	9	VEST	EXERCISE1	40	50
	282	36.95	36.87	36.78	126	9	VEST	EXERCISE1	40	50
	283	36.95	36.86	36.78	123	9	VEST	EXERCISE1	40	50
	284	36.95	36.81	36.77	125	9	VEST	EXERCISE1	40	50
	285	36.95	36.80	36.76	121	9	VEST	EXERCISE1	40	50
	286	36.95	36.84	36.76	121	9	VEST	EXERCISE1	40	50
	287	36.96	36.85	36.77	122	9	VEST	EXERCISE1	40	50
	288	36.97	36.86	36.78	123	9	VEST	EXERCISE1	40	50
	289	36.98	36.88	36.78	128	9	VEST	EXERCISE1	40	50
	290	36.98	36.89	36.77	129	9	VEST	EXERCISE1	40	50
	291	36.98	36.88	36.77	128	9	VEST	EXERCISE1	40	50
	292	36.97	36.88	36.78	128	9	VEST	EXERCISE1	40	50
	293	36.97	36.87	36.78	128	9	VEST	EXERCISE1	40	50
	294	36.98	36.86	36.78	125	9	VEST	EXERCISE1	40	50
	295	36.99	36.78	36.78	126	9	VEST	EXERCISE1	40	50
	296	37.00	36.77	36.79	124	9	VEST	EXERCISE1	40	50
	297	37.02	36.83	36.80	125	9	VEST	EXERCISE1	40	50
	298	37.02	36.86	36.81	125	9	VEST	EXERCISE1	40	50
	299	37.02	36.89	36.81	125	9	VEST	EXERCISE1	40	50
	300	37.02	36.81	36.81	124	9	VEST	EXERCISE1	40	50
50	301	37.02	36.78	36.83	121	9	VEST	EXERCISE1	40	50
	302	37.03	36.86	36.84	122	9	VEST	EXERCISE1	40	50
	303	37.03	36.88	36.85	126	9	VEST	EXERCISE1	40	50
	304	37.03	36.89	36.86	125	9	VEST	EXERCISE1	40	50
	305	37.04	36.91	36.87	125	9	VEST	EXERCISE1	40	50
	306	37.05	36.92	36.88	129	9	VEST	EXERCISE1	40	50
	307	37.05	36.93	36.87	126	9	VEST	EXERCISE1	40	50
	308	37.05	36.92	36.87	124	9	VEST	EXERCISE1	40	50
	309	37.07	36.93	36.86	128	9	VEST	EXERCISE1	40	50
	310	37.07	36.93	36.85	128	9	VEST	EXERCISE1	40	50
	311	37.07	36.92	36.86	130	9	VEST	EXERCISE1	40	50
	312	37.08	36.93	36.86	126	9	VEST	EXERCISE1	40	50
	313	37.08	36.95	36.86	127	9	VEST	EXERCISE1	40	50
	314	37.09	36.95	36.87	125	9	VEST	EXERCISE1	40	50
	315	37.09	36.96	36.86	123	9	VEST	EXERCISE1	40	50
	316	37.10	36.97	36.87	122	9	VEST	EXERCISE1	40	50
	317	37.11	36.98	36.86	125	9	VEST	EXERCISE1	40	50
	318	37.11	36.98	36.83	125	9	VEST	EXERCISE1	40	50
	319	37.12	36.98	36.85	124	9	VEST	EXERCISE1	40	50
	320	37.13	36.98	36.88	123	9	VEST	REST2	28	50
	321	37.13	36.98	36.89	119	9	VEST	REST2	28	50

min	number	Tre	Tes	Tsk-head	HR	ID	condition	period	Ta	RH
	322	37.13	36.99	36.85	114	9	VEST	REST2	28	50
	323	37.12	37.03	36.71	110	9	VEST	REST2	28	50
	324	37.12	37.03	36.51	112	9	VEST	REST2	28	50
	325	37.13	37.01	36.41	104	9	VEST	REST2	28	50
	326	37.13	36.99	36.37	95	9	VEST	REST2	28	50
	327	37.14	36.96	36.26	94	9	VEST	REST2	28	50
	328	37.15	36.85	36.18	97	9	VEST	REST2	28	50
	329	37.15	36.84	36.16	96	9	VEST	REST2	28	50
	330	37.14	36.91	36.13	95	9	VEST	REST2	28	50
55	331	37.13	36.89	36.13	93	9	VEST	REST2	28	50
	332	37.14	36.87	36.08	92	9	VEST	REST2	28	50
	333	37.14	36.88	36.04	90	9	VEST	REST2	28	50
	334	37.15	36.77	36.05	94	9	VEST	REST2	28	50
	335	37.15	36.73	36.05	87	9	VEST	REST2	28	50
	336	37.16	36.81	36.02	83	9	VEST	REST2	28	50
	337	37.17	36.80	35.97	89	9	VEST	REST2	28	50
	338	37.17	32.05	35.95	89	9	VEST	REST2	28	50
	339	37.17	26.93	35.94	104	9	VEST	REST2	28	50
	340	37.18	28.03	35.94	98	9	VEST	REST2	28	50
	341	37.18	31.19	35.93	89	9	VEST	REST2	28	50
	342	37.17	29.89	35.91	97	9	VEST	REST2	28	50
	343	37.17	29.20	35.91	100	9	VEST	REST2	28	50
	344	37.18	30.43	35.91	100	9	VEST	REST2	28	50
	345	37.18	30.39	35.91	83	9	VEST	REST2	28	50
	346	37.18	31.86	35.92	83	9	VEST	REST2	28	50
	347	37.18	33.05	35.92	83	9	VEST	REST2	28	50
	348	37.18	31.80	35.91	88	9	VEST	REST2	28	50
	349	37.18	30.62	35.89	84	9	VEST	REST2	28	50
	350	37.18	32.33	35.85	84	9	VEST	REST2	28	50
	351	37.17	33.45	35.82	87	9	VEST	REST2	28	50
	352	37.17	33.83	35.79	85	9	VEST	REST2	28	50
	353	37.17	34.57	35.76	73	9	VEST	REST2	28	50
	354	37.19	31.31	35.75	83	9	VEST	REST2	28	50
	355	37.20	28.09	35.74	91	9	VEST	REST2	28	50
	356	37.20	29.21	35.69	83	9	VEST	REST2	28	50
	357	37.19	31.02	35.65	85	9	VEST	REST2	28	50
	358	37.19	32.61	35.58	70	9	VEST	REST2	28	50
	359	37.19	33.07	35.51	75	9	VEST	REST2	28	50
	360	37.19	33.58	35.52	84	9	VEST	REST2	28	50
60	361	37.19	34.30	35.53	85	9	VEST	REST2	28	50
	362	37.19	31.76	35.51	88	9	VEST	REST2	28	50
	363	37.19	30.05	35.50	81	9	VEST	REST2	28	50
	364	37.19	31.82	35.49	82	9	VEST	REST2	28	50
	365	37.18	32.77	35.49	86	9	VEST	REST2	28	50
	366	37.18	33.25	35.44	84	9	VEST	REST2	28	50
	367	37.18	32.77	35.40	84	9	VEST	REST2	28	50

min	number	Tre	Tes	Tsk-head	HR	ID	condition	period	Ta	RH
	368	37.17	32.51	35.38	83	9	VEST	REST2	28	50
	369	37.17	32.94	35.33	81	9	VEST	REST2	28	50
	370	37.17	33.24	35.34	78	9	VEST	REST2	28	50
	371	37.17	33.62	35.34	85	9	VEST	REST2	28	50
	372	37.16	34.47	35.32	79	9	VEST	REST2	28	50
	373	37.17	31.38	35.32	82	9	VEST	REST2	28	50
	374	37.17	28.76	35.29	87	9	VEST	REST2	28	50
	375	37.16	31.12	35.26	79	9	VEST	REST2	28	50
	376	37.17	32.76	35.24	78	9	VEST	REST2	28	50
	377	37.17	33.54	35.20	78	9	VEST	REST2	28	50
	378	37.17	34.01	35.18	78	9	VEST	REST2	28	50
	379	37.16	34.40	35.17	82	9	VEST	REST2	28	50
	380	37.16	34.88	35.19	82	9	VEST	REST2	28	50
	381	37.17	35.22	35.23	83	9	VEST	REST2	28	50
	382	37.16	35.34	35.25	81	9	VEST	REST2	28	50
	383	37.16	35.57	35.24	84	9	VEST	REST2	28	50
	384	37.16	34.06	35.21	89	9	VEST	REST2	28	50
	385	37.16	33.02	35.18	85	9	VEST	REST2	28	50
	386	37.15	33.82	35.19	81	9	VEST	REST2	28	50
	387	37.15	34.16	35.15	84	9	VEST	REST2	28	50
	388	37.15	34.55	35.09	83	9	VEST	REST2	28	50
	389	37.15	33.76	35.09	84	9	VEST	REST2	28	50
	390	37.15	33.74	35.09	78	9	VEST	REST2	28	50
65	391	37.14	34.85	35.11	79	9	VEST	REST2	28	50
	392	37.14	35.26	35.12	78	9	VEST	REST2	28	50
	393	37.15	35.48	35.08	78	9	VEST	REST2	28	50
	394	37.15	35.60	35.07	74	9	VEST	REST2	28	50
	395	37.15	35.81	35.06	79	9	VEST	REST2	28	50
	396	37.15	35.89	35.04	71	9	VEST	REST2	28	50
	397	37.15	35.93	35.06	74	9	VEST	REST2	28	50
	398	37.15	36.12	35.07	74	9	VEST	REST2	28	50
	399	37.15	36.28	35.09	84	9	VEST	REST2	28	50
	400	37.14	36.29	35.15	87	9	VEST	REST2	28	50
	401	37.14	36.28	35.21	83	9	VEST	REST2	28	50
	402	37.15	36.27	35.25	83	9	VEST	REST2	28	50
	403	37.15	36.31	35.32	79	9	VEST	REST2	28	50
	404	37.15	36.38	35.34	77	9	VEST	REST2	28	50
	405	37.16	36.42	35.37	77	9	VEST	REST2	28	50
	406	37.16	36.43	35.37	81	9	VEST	REST2	28	50
	407	37.16	36.42	35.37	76	9	VEST	REST2	28	50
	408	37.15	36.42	35.39	73	9	VEST	REST2	28	50
	409	37.14	36.46	35.43	70	9	VEST	REST2	28	50
	410	37.14	36.41	35.49	74	9	VEST	REST2	28	50
	411	37.13	36.40	35.48	75	9	VEST	REST2	28	50
	412	37.13	36.49	35.48	79	9	VEST	REST2	28	50
	413	37.13	36.51	35.50	81	9	VEST	REST2	28	50

min	number	Tre	Tes	Tsk-head	HR	ID	condition	period	Ta	RH
	414	37.14	36.52	35.52	81	9	VEST	REST2	28	50
	415	37.13	36.53	35.52	79	9	VEST	REST2	28	50
	416	37.12	36.46	35.50	79	9	VEST	REST2	28	50
	417	37.13	36.45	35.50	85	9	VEST	REST2	28	50
	418	37.12	36.50	35.53	84	9	VEST	REST2	28	50
	419	37.12	36.52	35.55	76	9	VEST	REST2	28	50
	420	37.12	35.94	35.56	76	9	VEST	REST2	28	50
70	421	37.11	35.22	35.54	79	9	VEST	REST2	28	50
	422	37.11	35.47	35.52	81	9	VEST	REST2	28	50
	423	37.12	36.05	35.47	80	9	VEST	REST2	28	50
	424	37.12	36.26	35.47	79	9	VEST	REST2	28	50
	425	37.12	36.32	35.47	77	9	VEST	REST2	28	50
	426	37.12	36.40	35.43	73	9	VEST	REST2	28	50
	427	37.12	36.42	35.44	74	9	VEST	REST2	28	50
	428	37.12	36.43	35.49	77	9	VEST	REST2	28	50
	429	37.12	36.48	35.52	79	9	VEST	REST2	28	50
	430	37.13	36.50	35.52	81	9	VEST	REST2	28	50
	431	37.12	36.51	35.51	77	9	VEST	REST2	28	50
	432	37.12	36.51	35.49	78	9	VEST	REST2	28	50
	433	37.12	36.43	35.47	78	9	VEST	REST2	28	50
	434	37.12	36.43	35.47	81	9	VEST	REST2	28	50
	435	37.12	36.52	35.46	76	9	VEST	REST2	28	50
	436	37.12	36.54	35.46	84	9	VEST	REST2	28	50
	437	37.13	36.54	35.44	90	9	VEST	REST2	28	50
	438	37.12	36.56	35.41	87	9	VEST	REST2	28	50
	439	37.11	36.55	35.42	75	9	VEST	REST2	40	50
	440	37.11	36.53	35.45	86	9	VEST	REST2	40	50
	441	37.11	36.53	35.60	98	9	VEST	REST2	40	50
	442	37.12	36.55	35.81	92	9	VEST	REST2	40	50
	443	37.12	36.56	35.92	97	9	VEST	REST2	40	50
	444	37.12	36.56	36.03	93	9	VEST	REST2	40	50
	445	37.13	36.58	36.12	74	9	VEST	REST2	40	50
	446	37.14	36.60	36.19	73	9	VEST	REST2	40	50
	447	37.13	36.61	36.25	72	9	VEST	REST2	40	50
	448	37.13	36.60	36.29	70	9	VEST	REST2	40	50
	449	37.12	36.59	36.28	68	9	VEST	REST2	40	50
	450	37.11	36.58	36.25	70	9	VEST	REST2	40	50
75	451	37.10	36.58	36.24	70	9	VEST	REST2	40	50
	452	37.10	36.57	36.25	78	9	VEST	REST2	40	50
	453	37.10	36.55	36.28	80	9	VEST	REST2	40	50
	454	37.10	36.53	36.30	73	9	VEST	REST2	40	50
	455	37.10	36.53	36.32	74	9	VEST	REST2	40	50
	456	37.10	36.52	36.33	75	9	VEST	REST2	40	50
	457	37.09	36.52	36.33	75	9	VEST	REST2	40	50
	458	37.10	36.52	36.34	93	9	VEST	REST2	40	50
	459	37.10	36.52	36.33	101	9	VEST	REST2	40	50

min	number	Tre	Tes	Tsk-head	HR	ID	condition	period	Ta	RH
	460	37.10	36.51	36.33	94	9	VEST	REST2	40	50
	461	37.11	36.51	36.35	103	9	VEST	REST2	40	50
	462	37.11	36.50	36.37	100	9	VEST	REST2	40	50
	463	37.11	36.46	36.39	92	9	VEST	EXERCISE2	40	50
	464	37.10	36.45	36.40	89	9	VEST	EXERCISE2	40	50
	465	37.10	36.48	36.40	86	9	VEST	EXERCISE2	40	50
	466	37.09	36.48	36.40	85	9	VEST	EXERCISE2	40	50
	467	37.09	36.50	36.41	92	9	VEST	EXERCISE2	40	50
	468	37.09	36.51	36.42	101	9	VEST	EXERCISE2	40	50
	469	37.09	36.50	36.42	105	9	VEST	EXERCISE2	40	50
	470	37.09	36.51	36.43	105	9	VEST	EXERCISE2	40	50
	471	37.09	36.52	36.44	106	9	VEST	EXERCISE2	40	50
	472	37.09	36.54	36.45	109	9	VEST	EXERCISE2	40	50
	473	37.09	36.55	36.44	109	9	VEST	EXERCISE2	40	50
	474	37.10	36.56	36.41	107	9	VEST	EXERCISE2	40	50
	475	37.10	36.55	36.40	111	9	VEST	EXERCISE2	40	50
	476	37.09	36.56	36.40	111	9	VEST	EXERCISE2	40	50
	477	37.09	36.59	36.41	109	9	VEST	EXERCISE2	40	50
	478	37.08	36.54	36.41	112	9	VEST	EXERCISE2	40	50
	479	37.07	36.54	36.40	111	9	VEST	EXERCISE2	40	50
	480	37.08	36.61	36.40	108	9	VEST	EXERCISE2	40	50
80	481	37.08	36.63	36.41	108	9	VEST	EXERCISE2	40	50
	482	37.08	36.65	36.42	107	9	VEST	EXERCISE2	40	50
	483	37.08	36.67	36.44	110	9	VEST	EXERCISE2	40	50
	484	37.08	36.68	36.45	114	9	VEST	EXERCISE2	40	50
	485	37.07	36.68	36.46	117	9	VEST	EXERCISE2	40	50
	486	37.07	36.68	36.48	115	9	VEST	EXERCISE2	40	50
	487	37.08	36.69	36.49	112	9	VEST	EXERCISE2	40	50
	488	37.09	36.70	36.49	113	9	VEST	EXERCISE2	40	50
	489	37.09	36.70	36.50	114	9	VEST	EXERCISE2	40	50
	490	37.08	36.71	36.50	115	9	VEST	EXERCISE2	40	50
	491	37.08	36.69	36.51	114	9	VEST	EXERCISE2	40	50
	492	37.07	36.69	36.49	117	9	VEST	EXERCISE2	40	50
	493	37.07	36.70	36.48	115	9	VEST	EXERCISE2	40	50
	494	37.08	36.70	36.49	113	9	VEST	EXERCISE2	40	50
	495	37.08	36.70	36.50	113	9	VEST	EXERCISE2	40	50
	496	37.09	36.71	36.51	115	9	VEST	EXERCISE2	40	50
	497	37.09	36.68	36.52	117	9	VEST	EXERCISE2	40	50
	498	37.09	36.67	36.53	119	9	VEST	EXERCISE2	40	50
	499	37.09	36.69	36.53	120	9	VEST	EXERCISE2	40	50
	500	37.08	36.70	36.53	119	9	VEST	EXERCISE2	40	50
	501	37.08	36.71	36.53	119	9	VEST	EXERCISE2	40	50
	502	37.09	36.72	36.52	119	9	VEST	EXERCISE2	40	50
	503	37.09	36.71	36.54	122	9	VEST	EXERCISE2	40	50
	504	37.08	36.70	36.55	121	9	VEST	EXERCISE2	40	50
	505	37.08	36.72	36.55	121	9	VEST	EXERCISE2	40	50

min	number	Tre	Tes	Tsk-head	HR	ID	condition	period	Ta	RH
	506	37.09	36.74	36.56	119	9	VEST	EXERCISE2	40	50
	507	37.10	36.75	36.56	117	9	VEST	EXERCISE2	40	50
	508	37.10	36.75	36.57	119	9	VEST	EXERCISE2	40	50
	509	37.09	36.75	36.57	122	9	VEST	EXERCISE2	40	50
	510	37.08	36.80	36.56	121	9	VEST	EXERCISE2	40	50
85	511	37.08	36.80	36.55	118	9	VEST	EXERCISE2	40	50
	512	37.07	36.74	36.55	117	9	VEST	EXERCISE2	40	50
	513	37.08	36.73	36.56	120	9	VEST	EXERCISE2	40	50
	514	37.09	36.72	36.56	120	9	VEST	EXERCISE2	40	50
	515	37.09	36.71	36.56	120	9	VEST	EXERCISE2	40	50
	516	37.09	36.70	36.57	121	9	VEST	EXERCISE2	40	50
	517	37.09	36.69	36.58	120	9	VEST	EXERCISE2	40	50
	518	37.09	36.67	36.57	121	9	VEST	EXERCISE2	40	50
	519	37.09	36.68	36.56	118	9	VEST	EXERCISE2	40	50
	520	37.10	36.70	36.53	119	9	VEST	EXERCISE2	40	50
	521	37.09	36.71	36.52	119	9	VEST	EXERCISE2	40	50
	522	37.09	36.69	36.54	121	9	VEST	EXERCISE2	40	50
	523	37.09	36.66	36.56	121	9	VEST	EXERCISE2	40	50
	524	37.09	36.68	36.57	123	9	VEST	EXERCISE2	40	50
	525	37.09	36.69	36.58	122	9	VEST	EXERCISE2	40	50
	526	37.09	36.69	36.59	122	9	VEST	EXERCISE2	40	50
	527	37.09	36.69	36.59	122	9	VEST	EXERCISE2	40	50
	528	37.10	36.69	36.60	122	9	VEST	EXERCISE2	40	50
	529	37.10	36.69	36.60	121	9	VEST	EXERCISE2	40	50
	530	37.10	36.69	36.60	121	9	VEST	EXERCISE2	40	50
	531	37.10	36.70	36.61	121	9	VEST	EXERCISE2	40	50
	532	37.10	36.71	36.61	121	9	VEST	EXERCISE2	40	50
	533	37.09	36.72	36.60	120	9	VEST	EXERCISE2	40	50
	534	37.09	36.73	36.60	121	9	VEST	EXERCISE2	40	50
	535	37.09	36.75	36.61	122	9	VEST	EXERCISE2	40	50
	536	37.09	36.74	36.60	120	9	VEST	EXERCISE2	40	50
	537	37.09	36.69	36.60	119	9	VEST	EXERCISE2	40	50
	538	37.09	36.68	36.61	117	9	VEST	EXERCISE2	40	50
	539	37.09	36.74	36.61	119	9	VEST	EXERCISE2	40	50
	540	37.09	36.73	36.62	122	9	VEST	EXERCISE2	40	50
90	541	37.10	36.72	36.62	123	9	VEST	EXERCISE2	40	50
	542	37.10	36.74	36.62	125	9	VEST	EXERCISE2	40	50
	543	37.10	36.75	36.62	123	9	VEST	EXERCISE2	40	50
	544	37.10	36.76	36.63	121	9	VEST	EXERCISE2	40	50
	545	37.10	36.76	36.63	121	9	VEST	EXERCISE2	40	50
	546	37.10	36.78	36.63	122	9	VEST	EXERCISE2	40	50
	547	37.10	36.79	36.63	123	9	VEST	EXERCISE2	40	50
	548	37.10	36.79	36.64	122	9	VEST	EXERCISE2	40	50
	549	37.09	36.79	36.64	121	9	VEST	EXERCISE2	40	50
	550	37.10	36.76	36.64	123	9	VEST	EXERCISE2	40	50
	551	37.10	36.76	36.64	121	9	VEST	EXERCISE2	40	50

min	number	Tre	Tes	Tsk-head	HR	ID	condition	period	Ta	RH
	552	37.10	36.79	36.63	121	9	VEST	EXERCISE2	40	50
	553	37.09	36.79	36.63	120	9	VEST	EXERCISE2	40	50
	554	37.10	36.81	36.63	118	9	VEST	EXERCISE2	40	50
	555	37.11	36.82	36.64	119	9	VEST	EXERCISE2	40	50
	556	37.11	36.82	36.64	122	9	VEST	EXERCISE2	40	50
	557	37.11	36.84	36.65	121	9	VEST	EXERCISE2	40	50
	558	37.11	36.86	36.64	121	9	VEST	EXERCISE2	40	50
	559	37.11	36.87	36.65	123	9	VEST	EXERCISE2	40	50
	560	37.11	36.87	36.65	124	9	VEST	EXERCISE2	40	50
	561	37.11	36.87	36.65	124	9	VEST	EXERCISE2	40	50
	562	37.12	36.88	36.67	124	9	VEST	EXERCISE2	40	50
	563	37.12	36.89	36.68	125	9	VEST	EXERCISE2	40	50
	564	37.12	36.90	36.67	126	9	VEST	EXERCISE2	40	50
	565	37.12	36.89	36.66	125	9	VEST	EXERCISE2	40	50
	566	37.13	36.89	36.67	126	9	VEST	EXERCISE2	40	50
	567	37.14	36.81	36.69	127	9	VEST	EXERCISE2	40	50
	568	37.13	36.78	36.69	126	9	VEST	EXERCISE2	40	50
	569	37.14	36.86	36.69	119	9	VEST	EXERCISE2	40	50
	570	37.15	36.80	36.71	123	9	VEST	EXERCISE2	40	50
95	571	37.15	36.77	36.73	119	9	VEST	EXERCISE2	40	50
	572	37.15	36.83	36.74	123	9	VEST	EXERCISE2	40	50
	573	37.15	36.83	36.74	128	9	VEST	EXERCISE2	40	50
	574	37.15	36.84	36.73	128	9	VEST	EXERCISE2	40	50
	575	37.15	36.87	36.73	126	9	VEST	EXERCISE2	40	50
	576	37.15	36.88	36.73	126	9	VEST	EXERCISE2	40	50
	577	37.15	36.89	36.72	123	9	VEST	EXERCISE2	40	50
	578	37.16	36.81	36.74	126	9	VEST	EXERCISE2	40	50
	579	37.16	36.79	36.74	124	9	VEST	EXERCISE2	40	50
	580	37.16	36.88	36.73	123	9	VEST	EXERCISE2	40	50
	581	37.16	36.90	36.73	120	9	VEST	EXERCISE2	40	50
	582	37.17	36.91	36.74	125	9	VEST	EXERCISE2	40	50
	583	37.18	36.94	36.75	122	9	VEST	EXERCISE2	40	50
	584	37.17	36.95	36.76	124	9	VEST	EXERCISE2	40	50
	585	37.18	36.96	36.75	124	9	VEST	EXERCISE2	40	50
	586	37.18	36.93	36.73	127	9	VEST	EXERCISE2	40	50
	587	37.18	36.91	36.74	126	9	VEST	EXERCISE2	40	50
	588	37.19	36.94	36.75	126	9	VEST	EXERCISE2	40	50
	589	37.19	36.97	36.77	128	9	VEST	EXERCISE2	40	50
	590	37.19	36.98	36.77	128	9	VEST	EXERCISE2	40	50
	591	37.19	36.99	36.77	128	9	VEST	EXERCISE2	40	50
	592	37.20	37.00	36.78	130	9	VEST	EXERCISE2	40	50
	593	37.20	37.01	36.78	126	9	VEST	EXERCISE2	40	50
	594	37.21	37.02	36.79	126	9	VEST	EXERCISE2	40	50
	595	37.20	37.01	36.78	126	9	VEST	EXERCISE2	40	50
	596	37.20	37.01	36.77	128	9	VEST	EXERCISE2	40	50
	597	37.21	37.03	36.78	130	9	VEST	EXERCISE2	40	50

min	number	Tre	Tes	Tsk-head	HR	ID	condition	period	Ta	RH
	598	37.21	37.05	36.79	131	9	VEST	EXERCISE2	40	50
	599	37.21	37.05	36.81	127	9	VEST	EXERCISE2	40	50
	600	37.22	36.92	36.81	129	9	VEST	EXERCISE2	40	50
100	601	37.22	36.90	36.82	128	9	VEST	EXERCISE2	40	50
	602	37.22	37.02	36.84	129	9	VEST	EXERCISE2	40	50
	603	37.23	37.04	36.85	127	9	VEST	EXERCISE2	40	50
	604	37.25	37.05	36.86	131	9	VEST	EXERCISE2	40	50
	605	37.25	37.06	36.86	134	9	VEST	EXERCISE2	40	50
	606	37.25	37.09	36.88	134	9	VEST	EXERCISE2	40	50
	607	37.25	37.09	36.88	134	9	VEST	EXERCISE2	40	50
	608	37.26	37.09	36.87	136	9	VEST	EXERCISE2	40	50
	609	37.26	37.11	36.87	136	9	VEST	EXERCISE2	40	50
	610	37.26	37.12	36.88	134	9	VEST	EXERCISE2	40	50
	611	37.27	37.13	36.90	134	9	VEST	EXERCISE2	40	50
	612	37.27	37.14	36.92	136	9	VEST	EXERCISE2	40	50
	613	37.27	37.14	36.92	137	9	VEST	EXERCISE2	40	50
	614	37.27	37.14	36.91	137	9	VEST	EXERCISE2	40	50
	615	37.28	37.15	36.93	134	9	VEST	EXERCISE2	40	50
	616	37.29	37.15	36.94	135	9	VEST	EXERCISE2	40	50
	617	37.29	37.15	36.94	131	9	VEST	EXERCISE2	40	50
	618	37.29	37.16	36.94	134	9	VEST	EXERCISE2	40	50
	619	37.30	37.17	36.95	139	9	VEST	EXERCISE2	40	50
	620	37.30	37.16	36.96	141	9	VEST	EXERCISE2	40	50
	621	37.30	37.15	36.97	143	9	VEST	EXERCISE2	40	50
	622	37.30	37.18	36.98	142	9	VEST	EXERCISE2	40	50
	623	37.31	37.19	36.99	143	9	VEST	EXERCISE2	40	50
	624	37.32	37.21	37.00	141	9	VEST	EXERCISE2	40	50
	625	37.32	37.22	37.01	141	9	VEST	EXERCISE2	40	50
	626	37.33	37.22	37.02	145	9	VEST	EXERCISE2	40	50
	627	37.34	37.23	37.02	142	9	VEST	EXERCISE2	40	50
	628	37.34	37.25	37.05	143	9	VEST	EXERCISE2	40	50
	629	37.35	37.27	37.07	143	9	VEST	EXERCISE2	40	50
	630	37.35	37.28	37.07	143	9	VEST	EXERCISE2	40	50
105	631	37.35	37.29	37.06	144	9	VEST	EXERCISE2	40	50
	632	37.36	37.29	37.07	144	9	VEST	EXERCISE2	40	50
	633	37.36	37.31	37.08	146	9	VEST	EXERCISE2	40	50
	634	37.37	37.31	37.09	149	9	VEST	EXERCISE2	40	50
	635	37.37	37.31	37.09	148	9	VEST	EXERCISE2	40	50
	636	37.38	37.32	37.10	146	9	VEST	EXERCISE2	40	50
	637	37.38	37.28	37.11	147	9	VEST	EXERCISE2	40	50
	638	37.38	37.27	37.12	143	9	VEST	EXERCISE2	40	50
	639	37.39	37.33	37.13	143	9	VEST	EXERCISE2	40	50
	640	37.40	37.35	37.13	143	9	VEST	EXERCISE2	40	50
	641	37.41	37.36	37.13	147	9	VEST	EXERCISE2	40	50
	642	37.41	37.37	37.14	150	9	VEST	EXERCISE2	40	50
	643	37.41	37.37	37.15	148	9	VEST	EXERCISE2	40	50

min	number	Tre	Tes	Tsk-head	HR	ID	condition	period	Ta	RH
	644	37.42	37.37	37.15	142	9	VEST	REST3	28	50
	645	37.42	37.38	37.16	137	9	VEST	REST3	28	50
	646	37.42	37.40	37.10	132	9	VEST	REST3	28	50
	647	37.43	37.40	36.88	126	9	VEST	REST3	28	50
	648	37.44	37.37	36.70	117	9	VEST	REST3	28	50
	649	37.44	37.34	36.63	112	9	VEST	REST3	28	50
	650	37.44	37.34	36.56	114	9	VEST	REST3	28	50
	651	37.45	37.34	36.48	117	9	VEST	REST3	28	50
	652	37.45	37.32	36.42	106	9	VEST	REST3	28	50
	653	37.46	37.29	36.40	108	9	VEST	REST3	28	50
	654	37.48	37.28	36.37	108	9	VEST	REST3	28	50
	655	37.48	37.25	36.32	107	9	VEST	REST3	28	50
	656	37.48	37.19	36.27	101	9	VEST	REST3	28	50
	657	37.48	37.15	36.20	104	9	VEST	REST3	28	50
	658	37.47	37.08	36.05	98	9	VEST	REST3	28	50
	659	37.47	37.04	35.97	91	9	VEST	REST3	28	50
	660	37.47	37.07	36.01	93	9	VEST	REST3	28	50
110	661	37.47	37.06	36.04	91	9	VEST	REST3	28	50
	662	37.48	37.07	36.05	90	9	VEST	REST3	28	50
	663	37.48	37.06	36.03	93	9	VEST	REST3	28	50
	664	37.48	36.99	35.98	95	9	VEST	REST3	28	50
	665	37.48	36.95	35.92	93	9	VEST	REST3	28	50
	666	37.48	36.97	35.89	88	9	VEST	REST3	28	50
	667	37.49	36.97	35.85	91	9	VEST	REST3	28	50
	668	37.50	36.97	35.78	91	9	VEST	REST3	28	50
	669	37.50	36.96	35.74	89	9	VEST	REST3	28	50
	670	37.50	36.94	35.73	85	9	VEST	REST3	28	50
	671	37.50	36.94	35.73	90	9	VEST	REST3	28	50
	672	37.50	36.92	35.72	92	9	VEST	REST3	28	50
	673	37.49	36.87	35.71	83	9	VEST	REST3	28	50
	674	37.49	36.86	35.70	87	9	VEST	REST3	28	50
	675	37.50	36.88	35.71	89	9	VEST	REST3	28	50
	676	37.50	36.89	35.70	87	9	VEST	REST3	28	50
	677	37.49	36.89	35.64	86	9	VEST	REST3	28	50
	678	37.49	36.90	35.60	90	9	VEST	REST3	28	50
	679	37.49	36.90	35.60	90	9	VEST	REST3	28	50
	680	37.50	36.91	35.56	93	9	VEST	REST3	28	50
	681	37.50	36.91	35.45	95	9	VEST	REST3	28	50
	682	37.50	36.86	35.38	98	9	VEST	REST3	28	50
	683	37.50	36.84	35.37	89	9	VEST	REST3	28	50
	684	37.49	36.88	35.31	86	9	VEST	REST3	28	50
	685	37.48	36.90	35.30	84	9	VEST	REST3	28	50
	686	37.48	36.91	35.30	88	9	VEST	REST3	28	50
	687	37.48	36.92	35.26	81	9	VEST	REST3	28	50
	688	37.48	36.94	35.23	86	9	VEST	REST3	28	50
	689	37.49	36.95	35.22	84	9	VEST	REST3	28	50

min	number	Tre	Tes	Tsk-head	HR	ID	condition	period	Ta	RH
	690	37.48	36.91	35.19	84	9	VEST	REST3	28	50
115	691	37.47	36.89	35.17	87	9	VEST	REST3	28	50
	692	37.48	36.92	35.15	83	9	VEST	REST3	28	50
	693	37.48	36.94	35.13	83	9	VEST	REST3	28	50
	694	37.49	36.94	35.10	84	9	VEST	REST3	28	50
	695	37.49	36.96	35.06	86	9	VEST	REST3	28	50
	696	37.49	36.98	35.05	84	9	VEST	REST3	28	50
	697	37.48	36.93	35.05	85	9	VEST	REST3	28	50
	698	37.48	36.90	35.05	78	9	VEST	REST3	28	50
	699	37.48	36.95	35.08	81	9	VEST	REST3	28	50
	700	37.48	36.97	35.11	82	9	VEST	REST3	28	50
	701	37.48	36.98	35.10	80	9	VEST	REST3	28	50
	702	37.49	36.99	35.08	81	9	VEST	REST3	28	50
	703	37.49	36.99	35.06	81	9	VEST	REST3	28	50
	704	37.47	36.97	35.03	85	9	VEST	REST3	28	50
	705	37.47	36.96	35.02	81	9	VEST	REST3	28	50
	706	37.47	36.94	35.01	82	9	VEST	REST3	28	50
	707	37.48	36.93	35.00	73	9	VEST	REST3	28	50
	708	37.48	36.96	34.97	77	9	VEST	REST3	28	50
	709	37.47	36.95	34.93	83	9	VEST	REST3	28	50
0	1	37.19	37.10	35.27	74	1	FAN	REST1	28	50
	2	37.18	37.13	35.26	74	1	FAN	REST1	28	50
	3	37.18	37.13	35.24	73	1	FAN	REST1	28	50
	4	37.18	37.11	35.22	76	1	FAN	REST1	28	50
	5	37.18	37.12	35.21	81	1	FAN	REST1	28	50
	6	37.18	37.13	35.20	73	1	FAN	REST1	28	50
	7	37.18	37.12	35.19	71	1	FAN	REST1	28	50
	8	37.18	37.13	35.18	76	1	FAN	REST1	28	50
	9	37.17	37.12	35.18	74	1	FAN	REST1	28	50
	10	37.17	37.12	35.18	73	1	FAN	REST1	28	50
	11	37.17	37.12	35.19	77	1	FAN	REST1	28	50
	12	37.18	37.10	35.19	72	1	FAN	REST1	28	50
	13	37.21	37.11	35.19	73	1	FAN	REST1	28	50
	14	37.22	37.11	35.19	74	1	FAN	REST1	28	50
	15	37.21	37.10	35.20	75	1	FAN	REST1	28	50
	16	37.21	37.11	35.21	79	1	FAN	REST1	28	50
	17	37.20	37.11	35.22	88	1	FAN	REST1	28	50
	18	37.18	37.10	35.22	73	1	FAN	REST1	28	50
	19	37.17	37.10	35.22	72	1	FAN	REST1	28	50
	20	37.17	37.09	35.23	82	1	FAN	REST1	28	50
	21	37.17	37.10	35.23	82	1	FAN	REST1	28	50
	22	37.16	37.11	35.23	80	1	FAN	REST1	28	50
	23	37.16	37.09	35.24	83	1	FAN	REST1	28	50
	24	37.17	37.09	35.25	78	1	FAN	REST1	28	50
	25	37.19	37.10	35.26	80	1	FAN	REST1	28	50
	26	37.20	37.08	35.28	80	1	FAN	REST1	28	50

min	number	Tre	Tes	Tsk-head	HR	ID	condition	period	Ta	RH
	27	37.21	37.08	35.29	79	1	FAN	REST1	28	50
	28	37.21	37.09	35.30	76	1	FAN	REST1	28	50
	29	37.20	37.10	35.33	77	1	FAN	REST1	28	50
	30	37.18	37.11	35.35	69	1	FAN	REST1	28	50
5	31	37.18	37.09	35.34	80	1	FAN	REST1	28	50
	32	37.18	37.07	35.33	82	1	FAN	REST1	28	50
	33	37.17	37.08	35.33	85	1	FAN	REST1	28	50
	34	37.16	37.10	35.33	88	1	FAN	REST1	28	50
	35	37.16	37.10	35.32	79	1	FAN	REST1	28	50
	36	37.16	37.08	35.30	79	1	FAN	REST1	28	50
	37	37.16	37.06	35.30	77	1	FAN	REST1	28	50
	38	37.16	37.09	35.30	78	1	FAN	REST1	28	50
	39	37.17	37.07	35.30	77	1	FAN	REST1	28	50
	40	37.17	37.06	35.29	79	1	FAN	REST1	28	50
	41	37.16	37.09	35.29	78	1	FAN	REST1	28	50
	42	37.15	37.10	35.29	77	1	FAN	REST1	28	50
	43	37.15	37.08	35.29	79	1	FAN	REST1	28	50
	44	37.15	37.08	35.28	79	1	FAN	REST1	28	50
	45	37.15	37.09	35.30	77	1	FAN	REST1	28	50
	46	37.16	37.09	35.31	78	1	FAN	REST1	28	50
	47	37.15	37.09	35.31	82	1	FAN	REST1	28	50
	48	37.14	37.08	35.31	81	1	FAN	REST1	28	50
	49	37.14	37.09	35.30	75	1	FAN	REST1	28	50
	50	37.13	37.10	35.29	73	1	FAN	REST1	28	50
	51	37.12	37.11	35.29	73	1	FAN	REST1	28	50
	52	37.12	37.09	35.29	76	1	FAN	REST1	28	50
	53	37.12	37.08	35.27	76	1	FAN	REST1	28	50
	54	37.11	37.10	35.28	77	1	FAN	REST1	28	50
	55	37.11	37.10	35.31	72	1	FAN	REST1	28	50
	56	37.11	37.10	35.31	76	1	FAN	REST1	28	50
	57	37.10	37.10	35.29	74	1	FAN	REST1	28	50
	58	37.10	37.07	35.29	74	1	FAN	REST1	28	50
	59	37.11	37.09	35.31	77	1	FAN	REST1	28	50
	60	37.11	37.10	35.31	77	1	FAN	REST1	28	50
10	61	37.11	37.10	35.30	79	1	FAN	REST1	28	50
	62	37.10	37.10	35.30	80	1	FAN	REST1	28	50
	63	37.10	37.09	35.32	74	1	FAN	REST1	28	50
	64	37.11	37.09	35.33	76	1	FAN	REST1	28	50
	65	37.11	37.08	35.32	83	1	FAN	REST1	28	50
	66	37.11	37.07	35.31	80	1	FAN	REST1	28	50
	67	37.10	37.08	35.31	79	1	FAN	REST1	28	50
	68	37.11	37.09	35.32	78	1	FAN	REST1	28	50
	69	37.11	37.10	35.32	78	1	FAN	REST1	28	50
	70	37.11	37.09	35.32	83	1	FAN	REST1	28	50
	71	37.11	37.09	35.34	86	1	FAN	REST1	28	50
	72	37.10	37.10	35.36	84	1	FAN	REST1	28	50

min	number	Tre	Tes	Tsk-head	HR	ID	condition	period	Ta	RH
	73	37.10	37.11	35.34	79	1	FAN	REST1	28	50
	74	37.09	37.10	35.34	75	1	FAN	REST1	28	50
	75	37.09	37.07	35.36	74	1	FAN	REST1	28	50
	76	37.09	37.06	35.35	79	1	FAN	REST1	28	50
	77	37.09	37.08	35.37	91	1	FAN	REST1	28	50
	78	37.09	37.09	35.39	85	1	FAN	REST1	28	50
	79	37.09	37.06	35.40	72	1	FAN	REST1	28	50
	80	37.10	37.05	35.42	75	1	FAN	REST1	28	50
	81	37.10	37.06	35.41	77	1	FAN	REST1	28	50
	82	37.09	37.07	35.40	77	1	FAN	REST1	28	50
	83	37.09	37.07	35.39	77	1	FAN	REST1	28	50
	84	37.08	37.08	35.39	78	1	FAN	REST1	28	50
	85	37.08	37.08	35.39	80	1	FAN	REST1	28	50
	86	37.08	37.07	35.37	77	1	FAN	REST1	28	50
	87	37.08	37.06	35.36	77	1	FAN	REST1	28	50
	88	37.10	37.06	35.36	75	1	FAN	REST1	28	50
	89	37.11	37.04	35.36	76	1	FAN	REST1	28	50
	90	37.10	37.05	35.37	75	1	FAN	REST1	28	50
15	91	37.10	37.09	35.40	75	1	FAN	REST1	28	50
	92	37.09	37.09	35.42	80	1	FAN	REST1	28	50
	93	37.08	37.08	35.43	75	1	FAN	REST1	28	50
	94	37.08	37.09	35.46	74	1	FAN	REST1	28	50
	95	37.08	37.09	35.49	76	1	FAN	REST1	28	50
	96	37.08	37.09	35.49	73	1	FAN	REST1	28	50
	97	37.08	37.09	35.48	74	1	FAN	REST1	28	50
	98	37.08	37.08	35.47	75	1	FAN	REST1	28	50
	99	37.08	37.10	35.47	81	1	FAN	REST1	28	50
	100	37.08	37.10	35.47	78	1	FAN	REST1	28	50
	101	37.08	37.08	35.46	77	1	FAN	REST1	28	50
	102	37.08	37.08	35.45	78	1	FAN	REST1	28	50
	103	37.08	37.08	35.44	72	1	FAN	REST1	40	50
	104	37.09	37.09	35.50	99	1	FAN	REST1	40	50
	105	37.10	37.09	35.72	113	1	FAN	REST1	40	50
	106	37.11	37.09	35.93	81	1	FAN	REST1	40	50
	107	37.12	37.09	36.02	77	1	FAN	REST1	40	50
	108	37.11	37.07	36.08	81	1	FAN	REST1	40	50
	109	37.10	37.08	36.12	85	1	FAN	REST1	40	50
	110	37.10	37.09	36.20	94	1	FAN	REST1	40	50
	111	37.10	37.09	36.25	90	1	FAN	REST1	40	50
	112	37.10	37.10	36.27	86	1	FAN	REST1	40	50
	113	37.10	37.05	36.32	83	1	FAN	REST1	40	50
	114	37.09	37.02	36.37	83	1	FAN	REST1	40	50
	115	37.09	37.05	36.41	79	1	FAN	REST1	40	50
	116	37.10	37.05	36.45	82	1	FAN	REST1	40	50
	117	37.10	37.00	36.49	86	1	FAN	REST1	40	50
	118	37.12	36.97	36.52	86	1	FAN	REST1	40	50

min	number	Tre	Tes	Tsk-head	HR	ID	condition	period	Ta	RH
	119	37.13	36.97	36.56	78	1	FAN	REST1	40	50
	120	37.13	37.00	36.59	85	1	FAN	REST1	40	50
20	121	37.12	36.99	36.62	83	1	FAN	REST1	40	50
	122	37.12	36.95	36.64	81	1	FAN	REST1	40	50
	123	37.13	36.96	36.66	80	1	FAN	REST1	40	50
	124	37.13	36.98	36.68	81	1	FAN	REST1	40	50
	125	37.13	36.98	36.69	86	1	FAN	REST1	40	50
	126	37.12	36.97	36.70	87	1	FAN	REST1	40	50
	127	37.11	36.96	36.71	84	1	FAN	REST1	40	50
	128	37.11	37.01	36.72	92	1	FAN	REST1	40	50
	129	37.12	37.05	36.73	91	1	FAN	REST1	40	50
	130	37.12	37.05	36.75	91	1	FAN	REST1	40	50
	131	37.11	37.02	36.77	84	1	FAN	REST1	40	50
	132	37.12	37.00	36.78	78	1	FAN	REST1	40	50
	133	37.14	37.03	36.80	82	1	FAN	REST1	40	50
	134	37.14	37.09	36.82	96	1	FAN	REST1	40	50
	135	37.14	37.10	36.81	108	1	FAN	REST1	40	50
	136	37.15	37.10	36.81	77	1	FAN	REST1	40	50
	137	37.15	37.11	36.83	88	1	FAN	REST1	40	50
	138	37.14	37.13	36.84	88	1	FAN	REST1	40	50
	139	37.13	37.14	36.85	92	1	FAN	EXERCISE1	40	50
	140	37.12	37.15	36.86	78	1	FAN	EXERCISE1	40	50
	141	37.12	37.15	36.87	95	1	FAN	EXERCISE1	40	50
	142	37.11	37.15	36.88	100	1	FAN	EXERCISE1	40	50
	143	37.11	37.15	36.88	105	1	FAN	EXERCISE1	40	50
	144	37.10	37.13	36.89	106	1	FAN	EXERCISE1	40	50
	145	37.11	37.14	36.90	107	1	FAN	EXERCISE1	40	50
	146	37.13	37.18	36.91	109	1	FAN	EXERCISE1	40	50
	147	37.13	37.19	36.92	112	1	FAN	EXERCISE1	40	50
	148	37.13	37.17	36.93	106	1	FAN	EXERCISE1	40	50
	149	37.13	37.15	36.94	108	1	FAN	EXERCISE1	40	50
	150	37.12	37.15	36.94	109	1	FAN	EXERCISE1	40	50
25	151	37.13	37.15	36.95	111	1	FAN	EXERCISE1	40	50
	152	37.14	37.16	36.96	107	1	FAN	EXERCISE1	40	50
	153	37.15	37.19	36.97	107	1	FAN	EXERCISE1	40	50
	154	37.14	37.20	36.97	106	1	FAN	EXERCISE1	40	50
	155	37.13	37.19	36.98	109	1	FAN	EXERCISE1	40	50
	156	37.13	37.17	36.98	110	1	FAN	EXERCISE1	40	50
	157	37.14	37.19	36.99	114	1	FAN	EXERCISE1	40	50
	158	37.13	37.21	36.99	114	1	FAN	EXERCISE1	40	50
	159	37.14	37.21	37.00	115	1	FAN	EXERCISE1	40	50
	160	37.15	37.22	37.01	113	1	FAN	EXERCISE1	40	50
	161	37.15	37.25	37.02	116	1	FAN	EXERCISE1	40	50
	162	37.15	37.26	37.03	116	1	FAN	EXERCISE1	40	50
	163	37.14	37.26	37.03	111	1	FAN	EXERCISE1	40	50
	164	37.15	37.26	37.03	112	1	FAN	EXERCISE1	40	50

min	number	Tre	Tes	Tsk-head	HR	ID	condition	period	Ta	RH
	165	37.16	37.25	37.05	111	1	FAN	EXERCISE1	40	50
	166	37.16	37.27	37.07	114	1	FAN	EXERCISE1	40	50
	167	37.15	37.30	37.07	112	1	FAN	EXERCISE1	40	50
	168	37.15	37.30	37.08	112	1	FAN	EXERCISE1	40	50
	169	37.16	37.31	37.09	114	1	FAN	EXERCISE1	40	50
	170	37.15	37.33	37.10	115	1	FAN	EXERCISE1	40	50
	171	37.15	37.33	37.11	115	1	FAN	EXERCISE1	40	50
	172	37.15	37.34	37.12	115	1	FAN	EXERCISE1	40	50
	173	37.16	37.37	37.14	115	1	FAN	EXERCISE1	40	50
	174	37.17	37.38	37.15	119	1	FAN	EXERCISE1	40	50
	175	37.17	37.37	37.15	118	1	FAN	EXERCISE1	40	50
	176	37.18	37.37	37.16	119	1	FAN	EXERCISE1	40	50
	177	37.18	37.40	37.18	118	1	FAN	EXERCISE1	40	50
	178	37.18	37.41	37.19	121	1	FAN	EXERCISE1	40	50
	179	37.18	37.40	37.21	119	1	FAN	EXERCISE1	40	50
	180	37.18	37.43	37.23	115	1	FAN	EXERCISE1	40	50
30	181	37.18	37.45	37.25	116	1	FAN	EXERCISE1	40	50
	182	37.18	37.46	37.28	118	1	FAN	EXERCISE1	40	50
	183	37.19	37.46	37.30	118	1	FAN	EXERCISE1	40	50
	184	37.20	37.47	37.32	116	1	FAN	EXERCISE1	40	50
	185	37.21	37.51	37.33	114	1	FAN	EXERCISE1	40	50
	186	37.21	37.54	37.34	116	1	FAN	EXERCISE1	40	50
	187	37.21	37.54	37.35	117	1	FAN	EXERCISE1	40	50
	188	37.21	37.53	37.35	118	1	FAN	EXERCISE1	40	50
	189	37.21	37.53	37.35	119	1	FAN	EXERCISE1	40	50
	190	37.21	37.54	37.37	117	1	FAN	EXERCISE1	40	50
	191	37.22	37.55	37.38	118	1	FAN	EXERCISE1	40	50
	192	37.22	37.55	37.38	120	1	FAN	EXERCISE1	40	50
	193	37.21	37.57	37.37	120	1	FAN	EXERCISE1	40	50
	194	37.21	37.57	37.37	121	1	FAN	EXERCISE1	40	50
	195	37.22	37.56	37.38	121	1	FAN	EXERCISE1	40	50
	196	37.22	37.58	37.38	120	1	FAN	EXERCISE1	40	50
	197	37.22	37.60	37.39	122	1	FAN	EXERCISE1	40	50
	198	37.22	37.57	37.40	117	1	FAN	EXERCISE1	40	50
	199	37.23	37.51	37.39	121	1	FAN	EXERCISE1	40	50
	200	37.22	37.50	37.39	124	1	FAN	EXERCISE1	40	50
	201	37.22	37.53	37.39	123	1	FAN	EXERCISE1	40	50
	202	37.23	37.55	37.40	123	1	FAN	EXERCISE1	40	50
	203	37.24	37.57	37.41	122	1	FAN	EXERCISE1	40	50
	204	37.25	37.59	37.41	123	1	FAN	EXERCISE1	40	50
	205	37.24	37.59	37.42	123	1	FAN	EXERCISE1	40	50
	206	37.24	37.61	37.44	127	1	FAN	EXERCISE1	40	50
	207	37.24	37.62	37.44	125	1	FAN	EXERCISE1	40	50
	208	37.24	37.63	37.44	126	1	FAN	EXERCISE1	40	50
	209	37.24	37.66	37.45	125	1	FAN	EXERCISE1	40	50
	210	37.25	37.66	37.46	117	1	FAN	EXERCISE1	40	50

min	number	Tre	Tes	Tsk-head	HR	ID	condition	period	Ta	RH
35	211	37.24	37.66	37.46	120	1	FAN	EXERCISE1	40	50
	212	37.24	37.68	37.47	128	1	FAN	EXERCISE1	40	50
	213	37.25	37.69	37.48	123	1	FAN	EXERCISE1	40	50
	214	37.25	37.69	37.49	122	1	FAN	EXERCISE1	40	50
	215	37.25	37.70	37.49	122	1	FAN	EXERCISE1	40	50
	216	37.26	37.69	37.49	125	1	FAN	EXERCISE1	40	50
	217	37.25	37.69	37.49	126	1	FAN	EXERCISE1	40	50
	218	37.25	37.72	37.50	128	1	FAN	EXERCISE1	40	50
	219	37.25	37.71	37.50	121	1	FAN	EXERCISE1	40	50
	220	37.25	37.72	37.51	123	1	FAN	EXERCISE1	40	50
	221	37.26	37.74	37.51	123	1	FAN	EXERCISE1	40	50
	222	37.26	37.73	37.51	126	1	FAN	EXERCISE1	40	50
	223	37.27	37.73	37.52	127	1	FAN	EXERCISE1	40	50
	224	37.28	37.71	37.51	126	1	FAN	EXERCISE1	40	50
	225	37.29	37.69	37.51	126	1	FAN	EXERCISE1	40	50
	226	37.29	37.70	37.51	128	1	FAN	EXERCISE1	40	50
	227	37.29	37.70	37.50	130	1	FAN	EXERCISE1	40	50
	228	37.29	37.69	37.50	128	1	FAN	EXERCISE1	40	50
	229	37.29	37.65	37.50	128	1	FAN	EXERCISE1	40	50
	230	37.30	37.66	37.50	124	1	FAN	EXERCISE1	40	50
	231	37.30	37.68	37.50	121	1	FAN	EXERCISE1	40	50
	232	37.30	37.70	37.50	125	1	FAN	EXERCISE1	40	50
	233	37.30	37.71	37.50	127	1	FAN	EXERCISE1	40	50
	234	37.30	37.71	37.51	126	1	FAN	EXERCISE1	40	50
	235	37.30	37.71	37.50	126	1	FAN	EXERCISE1	40	50
	236	37.30	37.70	37.51	127	1	FAN	EXERCISE1	40	50
	237	37.30	37.72	37.52	126	1	FAN	EXERCISE1	40	50
	238	37.30	37.74	37.52	127	1	FAN	EXERCISE1	40	50
	239	37.30	37.74	37.51	126	1	FAN	EXERCISE1	40	50
	240	37.31	37.76	37.51	127	1	FAN	EXERCISE1	40	50
40	241	37.31	37.78	37.52	129	1	FAN	EXERCISE1	40	50
	242	37.32	37.79	37.53	127	1	FAN	EXERCISE1	40	50
	243	37.32	37.75	37.54	128	1	FAN	EXERCISE1	40	50
	244	37.32	37.71	37.54	127	1	FAN	EXERCISE1	40	50
	245	37.31	37.71	37.53	130	1	FAN	EXERCISE1	40	50
	246	37.32	37.73	37.54	133	1	FAN	EXERCISE1	40	50
	247	37.33	37.78	37.54	131	1	FAN	EXERCISE1	40	50
	248	37.33	37.77	37.54	133	1	FAN	EXERCISE1	40	50
	249	37.33	37.77	37.54	131	1	FAN	EXERCISE1	40	50
	250	37.33	37.79	37.54	129	1	FAN	EXERCISE1	40	50
	251	37.33	37.81	37.55	128	1	FAN	EXERCISE1	40	50
	252	37.33	37.80	37.55	127	1	FAN	EXERCISE1	40	50
	253	37.34	37.79	37.55	130	1	FAN	EXERCISE1	40	50
	254	37.34	37.82	37.55	128	1	FAN	EXERCISE1	40	50
	255	37.35	37.85	37.55	132	1	FAN	EXERCISE1	40	50
	256	37.34	37.86	37.55	129	1	FAN	EXERCISE1	40	50

min	number	Tre	Tes	Tsk-head	HR	ID	condition	period	Ta	RH
	257	37.34	37.84	37.56	129	1	FAN	EXERCISE1	40	50
	258	37.35	37.82	37.56	130	1	FAN	EXERCISE1	40	50
	259	37.36	37.80	37.55	129	1	FAN	EXERCISE1	40	50
	260	37.37	37.80	37.56	130	1	FAN	EXERCISE1	40	50
	261	37.37	37.80	37.56	131	1	FAN	EXERCISE1	40	50
	262	37.37	37.80	37.56	131	1	FAN	EXERCISE1	40	50
	263	37.36	37.83	37.56	131	1	FAN	EXERCISE1	40	50
	264	37.37	37.84	37.55	133	1	FAN	EXERCISE1	40	50
	265	37.37	37.85	37.55	136	1	FAN	EXERCISE1	40	50
	266	37.38	37.84	37.55	136	1	FAN	EXERCISE1	40	50
	267	37.39	37.81	37.56	136	1	FAN	EXERCISE1	40	50
	268	37.39	37.82	37.56	134	1	FAN	EXERCISE1	40	50
	269	37.39	37.84	37.56	133	1	FAN	EXERCISE1	40	50
	270	37.40	37.83	37.56	133	1	FAN	EXERCISE1	40	50
45	271	37.41	37.79	37.56	132	1	FAN	EXERCISE1	40	50
	272	37.41	37.83	37.56	132	1	FAN	EXERCISE1	40	50
	273	37.42	37.88	37.56	130	1	FAN	EXERCISE1	40	50
	274	37.42	37.87	37.56	129	1	FAN	EXERCISE1	40	50
	275	37.42	37.87	37.55	129	1	FAN	EXERCISE1	40	50
	276	37.42	37.89	37.56	130	1	FAN	EXERCISE1	40	50
	277	37.43	37.88	37.57	131	1	FAN	EXERCISE1	40	50
	278	37.44	37.89	37.58	131	1	FAN	EXERCISE1	40	50
	279	37.44	37.90	37.58	132	1	FAN	EXERCISE1	40	50
	280	37.44	37.88	37.59	130	1	FAN	EXERCISE1	40	50
	281	37.44	37.89	37.58	131	1	FAN	EXERCISE1	40	50
	282	37.45	37.88	37.58	128	1	FAN	EXERCISE1	40	50
	283	37.45	37.84	37.59	130	1	FAN	EXERCISE1	40	50
	284	37.46	37.83	37.60	132	1	FAN	EXERCISE1	40	50
	285	37.46	37.87	37.59	133	1	FAN	EXERCISE1	40	50
	286	37.46	37.87	37.59	133	1	FAN	EXERCISE1	40	50
	287	37.46	37.86	37.59	134	1	FAN	EXERCISE1	40	50
	288	37.46	37.85	37.58	133	1	FAN	EXERCISE1	40	50
	289	37.46	37.84	37.58	132	1	FAN	EXERCISE1	40	50
	290	37.47	37.86	37.59	132	1	FAN	EXERCISE1	40	50
	291	37.48	37.83	37.59	133	1	FAN	EXERCISE1	40	50
	292	37.48	37.85	37.59	132	1	FAN	EXERCISE1	40	50
	293	37.48	37.89	37.59	133	1	FAN	EXERCISE1	40	50
	294	37.48	37.88	37.60	136	1	FAN	EXERCISE1	40	50
	295	37.48	37.89	37.61	133	1	FAN	EXERCISE1	40	50
	296	37.48	37.89	37.62	133	1	FAN	EXERCISE1	40	50
	297	37.48	37.91	37.63	133	1	FAN	EXERCISE1	40	50
	298	37.49	37.90	37.62	136	1	FAN	EXERCISE1	40	50
	299	37.49	37.91	37.62	136	1	FAN	EXERCISE1	40	50
	300	37.49	37.93	37.61	136	1	FAN	EXERCISE1	40	50
50	301	37.48	37.93	37.60	136	1	FAN	EXERCISE1	40	50
	302	37.48	37.97	37.61	138	1	FAN	EXERCISE1	40	50

min	number	Tre	Tes	Tsk-head	HR	ID	condition	period	Ta	RH
	303	37.49	37.97	37.62	136	1	FAN	EXERCISE1	40	50
	304	37.50	37.95	37.61	137	1	FAN	EXERCISE1	40	50
	305	37.49	37.97	37.61	133	1	FAN	EXERCISE1	40	50
	306	37.49	37.95	37.61	136	1	FAN	EXERCISE1	40	50
	307	37.49	37.89	37.61	135	1	FAN	EXERCISE1	40	50
	308	37.49	37.91	37.62	134	1	FAN	EXERCISE1	40	50
	309	37.49	37.94	37.63	136	1	FAN	EXERCISE1	40	50
	310	37.49	37.97	37.65	137	1	FAN	EXERCISE1	40	50
	311	37.50	37.99	37.65	135	1	FAN	EXERCISE1	40	50
	312	37.50	37.98	37.63	135	1	FAN	EXERCISE1	40	50
	313	37.50	37.98	37.64	136	1	FAN	EXERCISE1	40	50
	314	37.51	37.99	37.64	137	1	FAN	EXERCISE1	40	50
	315	37.51	38.00	37.64	135	1	FAN	EXERCISE1	40	50
	316	37.50	37.98	37.63	134	1	FAN	EXERCISE1	40	50
	317	37.50	37.98	37.62	134	1	FAN	EXERCISE1	40	50
	318	37.51	38.00	37.61	133	1	FAN	EXERCISE1	40	50
	319	37.52	38.00	37.61	135	1	FAN	EXERCISE1	40	50
	320	37.53	38.00	37.62	132	1	FAN	REST2	28	50
	321	37.52	37.99	37.59	137	1	FAN	REST2	28	50
	322	37.52	37.98	37.40	130	1	FAN	REST2	28	50
	323	37.51	37.99	37.18	125	1	FAN	REST2	28	50
	324	37.51	37.91	37.07	109	1	FAN	REST2	28	50
	325	37.51	37.91	36.98	113	1	FAN	REST2	28	50
	326	37.51	37.97	36.91	109	1	FAN	REST2	28	50
	327	37.50	37.93	36.85	113	1	FAN	REST2	28	50
	328	37.50	37.93	36.80	109	1	FAN	REST2	28	50
	329	37.50	37.91	36.77	80	1	FAN	REST2	28	50
	330	37.51	37.89	36.74	96	1	FAN	REST2	28	50
55	331	37.51	37.92	36.63	101	1	FAN	REST2	28	50
	332	37.52	37.93	36.45	109	1	FAN	REST2	28	50
	333	37.52	37.84	36.35	102	1	FAN	REST2	28	50
	334	37.53	37.82	36.32	105	1	FAN	REST2	28	50
	335	37.53	37.80	36.23	107	1	FAN	REST2	28	50
	336	37.53	37.78	36.12	110	1	FAN	REST2	28	50
	337	37.54	37.78	36.12	106	1	FAN	REST2	28	50
	338	37.55	33.74	36.11	113	1	FAN	REST2	28	50
	339	37.54	29.02	36.09	115	1	FAN	REST2	28	50
	340	37.54	30.22	36.11	115	1	FAN	REST2	28	50
	341	37.54	31.26	36.12	104	1	FAN	REST2	28	50
	342	37.53	29.95	36.15	113	1	FAN	REST2	28	50
	343	37.54	30.04	36.18	110	1	FAN	REST2	28	50
	344	37.54	30.66	36.22	111	1	FAN	REST2	28	50
	345	37.54	30.63	36.23	112	1	FAN	REST2	28	50
	346	37.54	30.50	36.20	102	1	FAN	REST2	28	50
	347	37.53	30.19	36.18	112	1	FAN	REST2	28	50
	348	37.53	29.93	36.19	110	1	FAN	REST2	28	50

min	number	Tre	Tes	Tsk-head	HR	ID	condition	period	Ta	RH
	349	37.54	30.07	36.20	110	1	FAN	REST2	28	50
	350	37.54	30.27	36.24	111	1	FAN	REST2	28	50
	351	37.54	30.48	36.26	99	1	FAN	REST2	28	50
	352	37.54	30.50	36.23	104	1	FAN	REST2	28	50
	353	37.55	30.55	36.22	103	1	FAN	REST2	28	50
	354	37.54	30.82	36.21	107	1	FAN	REST2	28	50
	355	37.54	30.81	36.20	104	1	FAN	REST2	28	50
	356	37.54	30.61	36.16	111	1	FAN	REST2	28	50
	357	37.54	30.58	36.12	88	1	FAN	REST2	28	50
	358	37.55	30.88	36.13	103	1	FAN	REST2	28	50
	359	37.55	30.90	36.12	113	1	FAN	REST2	28	50
	360	37.55	30.69	36.09	96	1	FAN	REST2	28	50
60	361	37.54	30.78	36.08	89	1	FAN	REST2	28	50
	362	37.54	30.81	36.07	105	1	FAN	REST2	28	50
	363	37.54	31.13	36.04	87	1	FAN	REST2	28	50
	364	37.54	31.38	36.03	101	1	FAN	REST2	28	50
	365	37.54	31.40	36.03	104	1	FAN	REST2	28	50
	366	37.54	31.52	36.02	89	1	FAN	REST2	28	50
	367	37.54	31.65	36.02	92	1	FAN	REST2	28	50
	368	37.55	31.88	35.99	100	1	FAN	REST2	28	50
	369	37.56	32.06	35.95	101	1	FAN	REST2	28	50
	370	37.56	32.16	35.95	93	1	FAN	REST2	28	50
	371	37.56	32.27	35.93	96	1	FAN	REST2	28	50
	372	37.55	32.29	35.92	93	1	FAN	REST2	28	50
	373	37.55	32.24	35.94	91	1	FAN	REST2	28	50
	374	37.55	32.38	35.95	91	1	FAN	REST2	28	50
	375	37.56	32.49	35.94	103	1	FAN	REST2	28	50
	376	37.56	32.41	35.91	88	1	FAN	REST2	28	50
	377	37.56	32.80	35.88	80	1	FAN	REST2	28	50
	378	37.57	32.93	35.87	90	1	FAN	REST2	28	50
	379	37.58	32.85	35.88	91	1	FAN	REST2	28	50
	380	37.59	33.13	35.89	97	1	FAN	REST2	28	50
	381	37.59	33.15	35.91	82	1	FAN	REST2	28	50
	382	37.59	33.36	35.92	93	1	FAN	REST2	28	50
	383	37.58	33.41	35.91	90	1	FAN	REST2	28	50
	384	37.58	33.27	35.91	83	1	FAN	REST2	28	50
	385	37.58	33.34	35.92	97	1	FAN	REST2	28	50
	386	37.58	33.30	35.92	105	1	FAN	REST2	28	50
	387	37.59	33.31	35.94	104	1	FAN	REST2	28	50
	388	37.59	33.40	35.94	84	1	FAN	REST2	28	50
	389	37.59	33.43	35.93	85	1	FAN	REST2	28	50
	390	37.59	33.35	35.93	86	1	FAN	REST2	28	50
65	391	37.59	33.35	35.92	85	1	FAN	REST2	28	50
	392	37.59	33.44	35.90	89	1	FAN	REST2	28	50
	393	37.59	33.47	35.90	89	1	FAN	REST2	28	50
	394	37.59	33.51	35.94	91	1	FAN	REST2	28	50

min	number	Tre	Tes	Tsk-head	HR	ID	condition	period	Ta	RH
	395	37.59	33.58	35.97	86	1	FAN	REST2	28	50
	396	37.58	33.67	35.95	92	1	FAN	REST2	28	50
	397	37.59	33.79	35.92	88	1	FAN	REST2	28	50
	398	37.60	33.83	35.90	82	1	FAN	REST2	28	50
	399	37.60	33.89	35.86	85	1	FAN	REST2	28	50
	400	37.60	34.01	35.87	87	1	FAN	REST2	28	50
	401	37.60	34.28	35.92	91	1	FAN	REST2	28	50
	402	37.60	34.31	35.91	90	1	FAN	REST2	28	50
	403	37.60	34.32	35.88	87	1	FAN	REST2	28	50
	404	37.60	34.36	35.88	85	1	FAN	REST2	28	50
	405	37.61	34.21	35.90	86	1	FAN	REST2	28	50
	406	37.61	34.21	35.93	86	1	FAN	REST2	28	50
	407	37.61	34.26	35.94	87	1	FAN	REST2	28	50
	408	37.61	34.37	35.95	86	1	FAN	REST2	28	50
	409	37.60	34.44	35.96	85	1	FAN	REST2	28	50
	410	37.59	34.45	35.97	83	1	FAN	REST2	28	50
	411	37.58	34.51	35.99	84	1	FAN	REST2	28	50
	412	37.58	34.58	36.00	84	1	FAN	REST2	28	50
	413	37.58	34.63	35.96	89	1	FAN	REST2	28	50
	414	37.56	34.66	35.93	90	1	FAN	REST2	28	50
	415	37.56	34.71	35.93	84	1	FAN	REST2	28	50
	416	37.57	34.78	35.94	86	1	FAN	REST2	28	50
	417	37.57	34.83	35.97	87	1	FAN	REST2	28	50
	418	37.58	34.82	35.98	89	1	FAN	REST2	28	50
	419	37.59	34.83	35.96	96	1	FAN	REST2	28	50
	420	37.59	35.05	35.91	87	1	FAN	REST2	28	50
70	421	37.60	35.13	35.84	86	1	FAN	REST2	28	50
	422	37.60	35.16	35.77	100	1	FAN	REST2	28	50
	423	37.60	35.36	35.75	100	1	FAN	REST2	28	50
	424	37.59	35.50	35.80	93	1	FAN	REST2	28	50
	425	37.59	35.60	35.84	90	1	FAN	REST2	28	50
	426	37.59	35.52	35.83	91	1	FAN	REST2	28	50
	427	37.59	35.38	35.80	90	1	FAN	REST2	28	50
	428	37.59	35.36	35.78	88	1	FAN	REST2	28	50
	429	37.59	35.42	35.79	90	1	FAN	REST2	28	50
	430	37.59	35.38	35.80	93	1	FAN	REST2	28	50
	431	37.58	35.36	35.82	83	1	FAN	REST2	28	50
	432	37.57	35.63	35.84	83	1	FAN	REST2	28	50
	433	37.57	35.75	35.84	93	1	FAN	REST2	28	50
	434	37.57	35.63	35.84	93	1	FAN	REST2	28	50
	435	37.56	35.70	35.85	86	1	FAN	REST2	28	50
	436	37.55	35.87	35.85	86	1	FAN	REST2	28	50
	437	37.55	36.10	35.84	85	1	FAN	REST2	28	50
	438	37.53	36.18	35.85	84	1	FAN	REST2	28	50
	439	37.52	35.93	35.85	91	1	FAN	REST2	40	50
	440	37.53	36.06	36.00	100	1	FAN	REST2	40	50

min	number	Tre	Tes	Tsk-head	HR	ID	condition	period	Ta	RH
	441	37.53	36.21	36.24	89	1	FAN	REST2	40	50
	442	37.54	35.96	36.38	83	1	FAN	REST2	40	50
	443	37.55	35.84	36.47	92	1	FAN	REST2	40	50
	444	37.54	35.85	36.56	93	1	FAN	REST2	40	50
	445	37.54	35.83	36.62	93	1	FAN	REST2	40	50
	446	37.54	35.79	36.67	95	1	FAN	REST2	40	50
	447	37.54	35.81	36.71	95	1	FAN	REST2	40	50
	448	37.55	35.83	36.74	93	1	FAN	REST2	40	50
	449	37.55	35.87	36.77	93	1	FAN	REST2	40	50
	450	37.53	36.40	36.79	100	1	FAN	REST2	40	50
75	451	37.53	36.71	36.80	92	1	FAN	REST2	40	50
	452	37.52	36.52	36.81	86	1	FAN	REST2	40	50
	453	37.52	36.23	36.82	80	1	FAN	REST2	40	50
	454	37.51	36.10	36.83	89	1	FAN	REST2	40	50
	455	37.51	36.57	36.84	91	1	FAN	REST2	40	50
	456	37.52	36.90	36.85	85	1	FAN	REST2	40	50
	457	37.53	36.96	36.86	88	1	FAN	REST2	40	50
	458	37.54	37.00	36.87	95	1	FAN	REST2	40	50
	459	37.53	37.02	36.87	106	1	FAN	REST2	40	50
	460	37.52	37.03	36.87	80	1	FAN	REST2	40	50
	461	37.52	37.03	36.87	99	1	FAN	REST2	40	50
	462	37.51	37.04	36.86	97	1	FAN	REST2	40	50
	463	37.51	37.06	36.88	87	1	FAN	EXERCISE2	40	50
	464	37.51	37.07	36.90	95	1	FAN	EXERCISE2	40	50
	465	37.51	37.05	36.91	100	1	FAN	EXERCISE2	40	50
	466	37.50	37.03	36.92	104	1	FAN	EXERCISE2	40	50
	467	37.50	37.03	36.93	105	1	FAN	EXERCISE2	40	50
	468	37.50	37.03	36.94	110	1	FAN	EXERCISE2	40	50
	469	37.50	37.01	36.97	114	1	FAN	EXERCISE2	40	50
	470	37.50	37.03	36.97	117	1	FAN	EXERCISE2	40	50
	471	37.50	37.02	36.98	112	1	FAN	EXERCISE2	40	50
	472	37.50	36.96	36.99	114	1	FAN	EXERCISE2	40	50
	473	37.50	37.01	37.00	117	1	FAN	EXERCISE2	40	50
	474	37.51	37.03	37.02	120	1	FAN	EXERCISE2	40	50
	475	37.51	37.01	37.03	119	1	FAN	EXERCISE2	40	50
	476	37.51	37.01	37.05	116	1	FAN	EXERCISE2	40	50
	477	37.50	36.96	37.07	118	1	FAN	EXERCISE2	40	50
	478	37.50	36.97	37.10	123	1	FAN	EXERCISE2	40	50
	479	37.50	36.98	37.13	121	1	FAN	EXERCISE2	40	50
	480	37.50	36.96	37.14	121	1	FAN	EXERCISE2	40	50
80	481	37.50	36.96	37.15	120	1	FAN	EXERCISE2	40	50
	482	37.51	36.99	37.18	120	1	FAN	EXERCISE2	40	50
	483	37.51	37.03	37.20	122	1	FAN	EXERCISE2	40	50
	484	37.51	37.06	37.20	121	1	FAN	EXERCISE2	40	50
	485	37.51	37.02	37.22	118	1	FAN	EXERCISE2	40	50
	486	37.51	37.03	37.25	120	1	FAN	EXERCISE2	40	50

min	number	Tre	Tes	Tsk-head	HR	ID	condition	period	Ta	RH
	487	37.51	37.08	37.26	122	1	FAN	EXERCISE2	40	50
	488	37.50	37.05	37.27	121	1	FAN	EXERCISE2	40	50
	489	37.50	37.03	37.28	123	1	FAN	EXERCISE2	40	50
	490	37.50	36.90	37.29	122	1	FAN	EXERCISE2	40	50
	491	37.50	36.95	37.29	122	1	FAN	EXERCISE2	40	50
	492	37.51	37.20	37.30	122	1	FAN	EXERCISE2	40	50
	493	37.52	37.28	37.31	125	1	FAN	EXERCISE2	40	50
	494	37.52	37.32	37.32	126	1	FAN	EXERCISE2	40	50
	495	37.53	37.34	37.33	125	1	FAN	EXERCISE2	40	50
	496	37.53	37.33	37.33	126	1	FAN	EXERCISE2	40	50
	497	37.53	37.33	37.33	126	1	FAN	EXERCISE2	40	50
	498	37.53	37.33	37.33	127	1	FAN	EXERCISE2	40	50
	499	37.52	37.34	37.33	124	1	FAN	EXERCISE2	40	50
	500	37.52	37.35	37.33	126	1	FAN	EXERCISE2	40	50
	501	37.53	37.37	37.34	128	1	FAN	EXERCISE2	40	50
	502	37.52	37.39	37.35	128	1	FAN	EXERCISE2	40	50
	503	37.52	37.38	37.35	132	1	FAN	EXERCISE2	40	50
	504	37.53	37.40	37.35	125	1	FAN	EXERCISE2	40	50
	505	37.53	37.42	37.36	125	1	FAN	EXERCISE2	40	50
	506	37.54	37.41	37.36	127	1	FAN	EXERCISE2	40	50
	507	37.53	37.42	37.36	129	1	FAN	EXERCISE2	40	50
	508	37.53	37.44	37.37	131	1	FAN	EXERCISE2	40	50
	509	37.55	37.44	37.39	132	1	FAN	EXERCISE2	40	50
	510	37.55	37.44	37.39	134	1	FAN	EXERCISE2	40	50
85	511	37.55	37.45	37.39	133	1	FAN	EXERCISE2	40	50
	512	37.56	37.49	37.41	132	1	FAN	EXERCISE2	40	50
	513	37.56	37.50	37.41	132	1	FAN	EXERCISE2	40	50
	514	37.55	37.48	37.41	130	1	FAN	EXERCISE2	40	50
	515	37.56	37.50	37.42	131	1	FAN	EXERCISE2	40	50
	516	37.56	37.49	37.41	131	1	FAN	EXERCISE2	40	50
	517	37.55	37.48	37.41	130	1	FAN	EXERCISE2	40	50
	518	37.56	37.51	37.41	130	1	FAN	EXERCISE2	40	50
	519	37.57	37.54	37.41	131	1	FAN	EXERCISE2	40	50
	520	37.57	37.54	37.41	130	1	FAN	EXERCISE2	40	50
	521	37.57	37.53	37.40	128	1	FAN	EXERCISE2	40	50
	522	37.57	37.52	37.41	130	1	FAN	EXERCISE2	40	50
	523	37.57	37.53	37.41	133	1	FAN	EXERCISE2	40	50
	524	37.57	37.51	37.41	132	1	FAN	EXERCISE2	40	50
	525	37.56	37.49	37.41	130	1	FAN	EXERCISE2	40	50
	526	37.56	37.50	37.41	131	1	FAN	EXERCISE2	40	50
	527	37.57	37.44	37.43	130	1	FAN	EXERCISE2	40	50
	528	37.57	37.30	37.43	128	1	FAN	EXERCISE2	40	50
	529	37.58	37.34	37.43	127	1	FAN	EXERCISE2	40	50
	530	37.58	37.47	37.42	131	1	FAN	EXERCISE2	40	50
	531	37.59	37.49	37.42	134	1	FAN	EXERCISE2	40	50
	532	37.59	37.49	37.42	131	1	FAN	EXERCISE2	40	50

min	number	Tre	Tes	Tsk-head	HR	ID	condition	period	Ta	RH
	533	37.59	37.49	37.42	131	1	FAN	EXERCISE2	40	50
	534	37.59	37.51	37.43	131	1	FAN	EXERCISE2	40	50
	535	37.59	37.55	37.45	131	1	FAN	EXERCISE2	40	50
	536	37.59	37.55	37.44	131	1	FAN	EXERCISE2	40	50
	537	37.60	37.54	37.44	133	1	FAN	EXERCISE2	40	50
	538	37.62	37.55	37.45	134	1	FAN	EXERCISE2	40	50
	539	37.63	37.57	37.46	136	1	FAN	EXERCISE2	40	50
	540	37.63	37.56	37.47	137	1	FAN	EXERCISE2	40	50
90	541	37.61	37.56	37.46	134	1	FAN	EXERCISE2	40	50
	542	37.60	37.59	37.46	134	1	FAN	EXERCISE2	40	50
	543	37.60	37.61	37.46	132	1	FAN	EXERCISE2	40	50
	544	37.61	37.61	37.47	134	1	FAN	EXERCISE2	40	50
	545	37.61	37.62	37.46	133	1	FAN	EXERCISE2	40	50
	546	37.62	37.61	37.45	134	1	FAN	EXERCISE2	40	50
	547	37.62	37.52	37.46	134	1	FAN	EXERCISE2	40	50
	548	37.62	37.51	37.45	134	1	FAN	EXERCISE2	40	50
	549	37.61	37.59	37.45	134	1	FAN	EXERCISE2	40	50
	550	37.61	37.60	37.45	135	1	FAN	EXERCISE2	40	50
	551	37.61	37.63	37.45	132	1	FAN	EXERCISE2	40	50
	552	37.61	37.66	37.42	135	1	FAN	EXERCISE2	40	50
	553	37.62	37.65	37.41	135	1	FAN	EXERCISE2	40	50
	554	37.61	37.64	37.43	135	1	FAN	EXERCISE2	40	50
	555	37.63	37.63	37.45	135	1	FAN	EXERCISE2	40	50
	556	37.65	37.64	37.46	136	1	FAN	EXERCISE2	40	50
	557	37.65	37.64	37.46	136	1	FAN	EXERCISE2	40	50
	558	37.65	37.66	37.48	136	1	FAN	EXERCISE2	40	50
	559	37.65	37.68	37.48	136	1	FAN	EXERCISE2	40	50
	560	37.65	37.68	37.47	135	1	FAN	EXERCISE2	40	50
	561	37.65	37.68	37.48	134	1	FAN	EXERCISE2	40	50
	562	37.64	37.68	37.47	135	1	FAN	EXERCISE2	40	50
	563	37.65	37.70	37.48	135	1	FAN	EXERCISE2	40	50
	564	37.65	37.71	37.49	136	1	FAN	EXERCISE2	40	50
	565	37.64	37.72	37.50	134	1	FAN	EXERCISE2	40	50
	566	37.65	37.72	37.50	136	1	FAN	EXERCISE2	40	50
	567	37.66	37.73	37.49	135	1	FAN	EXERCISE2	40	50
	568	37.66	37.73	37.48	136	1	FAN	EXERCISE2	40	50
	569	37.66	37.71	37.48	134	1	FAN	EXERCISE2	40	50
	570	37.66	37.73	37.47	137	1	FAN	EXERCISE2	40	50
95	571	37.67	37.68	37.45	136	1	FAN	EXERCISE2	40	50
	572	37.67	37.64	37.45	134	1	FAN	EXERCISE2	40	50
	573	37.67	37.67	37.45	135	1	FAN	EXERCISE2	40	50
	574	37.67	37.70	37.46	137	1	FAN	EXERCISE2	40	50
	575	37.67	37.70	37.46	137	1	FAN	EXERCISE2	40	50
	576	37.67	37.70	37.44	137	1	FAN	EXERCISE2	40	50
	577	37.67	37.71	37.44	137	1	FAN	EXERCISE2	40	50
	578	37.68	37.73	37.44	137	1	FAN	EXERCISE2	40	50

min	number	Tre	Tes	Tsk-head	HR	ID	condition	period	Ta	RH
	579	37.69	37.75	37.44	137	1	FAN	EXERCISE2	40	50
	580	37.70	37.73	37.45	137	1	FAN	EXERCISE2	40	50
	581	37.71	37.73	37.45	137	1	FAN	EXERCISE2	40	50
	582	37.70	37.75	37.45	138	1	FAN	EXERCISE2	40	50
	583	37.69	37.75	37.46	137	1	FAN	EXERCISE2	40	50
	584	37.69	37.75	37.47	136	1	FAN	EXERCISE2	40	50
	585	37.69	37.75	37.46	137	1	FAN	EXERCISE2	40	50
	586	37.69	37.75	37.46	138	1	FAN	EXERCISE2	40	50
	587	37.70	37.76	37.46	139	1	FAN	EXERCISE2	40	50
	588	37.71	37.75	37.46	139	1	FAN	EXERCISE2	40	50
	589	37.71	37.73	37.47	140	1	FAN	EXERCISE2	40	50
	590	37.71	37.74	37.46	140	1	FAN	EXERCISE2	40	50
	591	37.71	37.73	37.44	142	1	FAN	EXERCISE2	40	50
	592	37.71	37.72	37.44	140	1	FAN	EXERCISE2	40	50
	593	37.72	37.72	37.44	142	1	FAN	EXERCISE2	40	50
	594	37.72	37.74	37.45	143	1	FAN	EXERCISE2	40	50
	595	37.72	37.74	37.46	142	1	FAN	EXERCISE2	40	50
	596	37.73	37.73	37.46	137	1	FAN	EXERCISE2	40	50
	597	37.74	37.73	37.46	137	1	FAN	EXERCISE2	40	50
	598	37.73	37.73	37.45	138	1	FAN	EXERCISE2	40	50
	599	37.73	37.72	37.44	140	1	FAN	EXERCISE2	40	50
	600	37.73	37.71	37.44	137	1	FAN	EXERCISE2	40	50
100	601	37.74	37.75	37.44	136	1	FAN	EXERCISE2	40	50
	602	37.74	37.79	37.42	135	1	FAN	EXERCISE2	40	50
	603	37.73	37.77	37.40	137	1	FAN	EXERCISE2	40	50
	604	37.73	37.78	37.39	140	1	FAN	EXERCISE2	40	50
	605	37.75	37.79	37.39	140	1	FAN	EXERCISE2	40	50
	606	37.75	37.76	37.38	140	1	FAN	EXERCISE2	40	50
	607	37.75	37.78	37.37	143	1	FAN	EXERCISE2	40	50
	608	37.76	37.72	37.38	139	1	FAN	EXERCISE2	40	50
	609	37.76	37.67	37.38	138	1	FAN	EXERCISE2	40	50
	610	37.75	37.72	37.39	140	1	FAN	EXERCISE2	40	50
	611	37.76	37.74	37.40	139	1	FAN	EXERCISE2	40	50
	612	37.76	37.73	37.39	140	1	FAN	EXERCISE2	40	50
	613	37.77	37.74	37.39	137	1	FAN	EXERCISE2	40	50
	614	37.79	37.74	37.41	141	1	FAN	EXERCISE2	40	50
	615	37.78	37.73	37.43	139	1	FAN	EXERCISE2	40	50
	616	37.78	37.75	37.44	140	1	FAN	EXERCISE2	40	50
	617	37.78	37.76	37.45	140	1	FAN	EXERCISE2	40	50
	618	37.77	37.75	37.45	140	1	FAN	EXERCISE2	40	50
	619	37.77	37.77	37.45	140	1	FAN	EXERCISE2	40	50
	620	37.78	37.79	37.47	140	1	FAN	EXERCISE2	40	50
	621	37.77	37.79	37.47	142	1	FAN	EXERCISE2	40	50
	622	37.77	37.79	37.47	142	1	FAN	EXERCISE2	40	50
	623	37.77	37.80	37.45	142	1	FAN	EXERCISE2	40	50
	624	37.78	37.80	37.44	142	1	FAN	EXERCISE2	40	50

min	number	Tre	Tes	Tsk-head	HR	ID	condition	period	Ta	RH
	625	37.78	37.80	37.46	140	1	FAN	EXERCISE2	40	50
	626	37.78	37.83	37.48	140	1	FAN	EXERCISE2	40	50
	627	37.78	37.83	37.48	141	1	FAN	EXERCISE2	40	50
	628	37.78	37.82	37.48	143	1	FAN	EXERCISE2	40	50
	629	37.79	37.81	37.48	143	1	FAN	EXERCISE2	40	50
	630	37.79	37.81	37.49	143	1	FAN	EXERCISE2	40	50
105	631	37.79	37.82	37.50	143	1	FAN	EXERCISE2	40	50
	632	37.80	37.83	37.51	143	1	FAN	EXERCISE2	40	50
	633	37.81	37.86	37.50	143	1	FAN	EXERCISE2	40	50
	634	37.81	37.77	37.50	144	1	FAN	EXERCISE2	40	50
	635	37.81	37.73	37.51	140	1	FAN	EXERCISE2	40	50
	636	37.81	37.78	37.53	139	1	FAN	EXERCISE2	40	50
	637	37.81	37.81	37.52	142	1	FAN	EXERCISE2	40	50
	638	37.81	37.82	37.52	144	1	FAN	EXERCISE2	40	50
	639	37.81	37.81	37.51	145	1	FAN	EXERCISE2	40	50
	640	37.82	37.82	37.50	143	1	FAN	EXERCISE2	40	50
	641	37.82	37.83	37.49	143	1	FAN	EXERCISE2	40	50
	642	37.82	37.87	37.48	144	1	FAN	EXERCISE2	40	50
	643	37.82	37.88	37.48	144	1	FAN	EXERCISE2	40	50
	644	37.82	37.86	37.48	140	1	FAN	REST3	28	50
	645	37.82	37.88	37.29	144	1	FAN	REST3	28	50
	646	37.83	37.93	36.97	131	1	FAN	REST3	28	50
	647	37.83	37.92	36.81	130	1	FAN	REST3	28	50
	648	37.82	37.92	36.78	123	1	FAN	REST3	28	50
	649	37.82	37.91	36.75	113	1	FAN	REST3	28	50
	650	37.83	37.90	36.73	116	1	FAN	REST3	28	50
	651	37.85	37.90	36.69	115	1	FAN	REST3	28	50
	652	37.84	37.87	36.65	115	1	FAN	REST3	28	50
	653	37.83	37.88	36.62	114	1	FAN	REST3	28	50
	654	37.82	37.87	36.55	115	1	FAN	REST3	28	50
	655	37.81	37.84	36.46	112	1	FAN	REST3	28	50
	656	37.80	37.83	36.39	110	1	FAN	REST3	28	50
	657	37.82	37.80	36.36	110	1	FAN	REST3	28	50
	658	37.84	37.79	36.38	111	1	FAN	REST3	28	50
	659	37.84	37.80	36.44	110	1	FAN	REST3	28	50
	660	37.83	37.78	36.41	107	1	FAN	REST3	28	50
110	661	37.82	37.73	36.37	108	1	FAN	REST3	28	50
	662	37.82	37.73	36.38	107	1	FAN	REST3	28	50
	663	37.81	37.72	36.36	107	1	FAN	REST3	28	50
	664	37.80	37.68	36.36	106	1	FAN	REST3	28	50
	665	37.80	37.68	36.37	106	1	FAN	REST3	28	50
	666	37.80	37.66	36.38	103	1	FAN	REST3	28	50
	667	37.80	37.64	36.33	102	1	FAN	REST3	28	50
	668	37.80	37.63	36.27	103	1	FAN	REST3	28	50
	669	37.80	37.62	36.27	102	1	FAN	REST3	28	50
	670	37.79	37.58	36.28	101	1	FAN	REST3	28	50

min	number	Tre	Tes	Tsk-head	HR	ID	condition	period	Ta	RH
	671	37.78	37.56	36.24	102	1	FAN	REST3	28	50
	672	37.78	37.55	36.22	100	1	FAN	REST3	28	50
	673	37.77	37.55	36.27	102	1	FAN	REST3	28	50
	674	37.77	37.54	36.28	102	1	FAN	REST3	28	50
	675	37.76	37.52	36.28	100	1	FAN	REST3	28	50
	676	37.76	37.50	36.28	102	1	FAN	REST3	28	50
	677	37.77	37.48	36.30	104	1	FAN	REST3	28	50
	678	37.77	37.47	36.30	100	1	FAN	REST3	28	50
	679	37.77	37.46	36.30	99	1	FAN	REST3	28	50
	680	37.76	37.45	36.30	98	1	FAN	REST3	28	50
	681	37.76	37.45	36.24	99	1	FAN	REST3	28	50
	682	37.76	37.45	36.13	101	1	FAN	REST3	28	50
	683	37.76	37.43	36.04	105	1	FAN	REST3	28	50
	684	37.76	37.43	35.98	96	1	FAN	REST3	28	50
	685	37.75	37.43	35.93	105	1	FAN	REST3	28	50
	686	37.75	37.41	35.90	103	1	FAN	REST3	28	50
	687	37.75	37.42	35.92	98	1	FAN	REST3	28	50
	688	37.77	37.42	35.92	97	1	FAN	REST3	28	50
	689	37.78	37.41	35.91	97	1	FAN	REST3	28	50
	690	37.77	37.42	35.89	96	1	FAN	REST3	28	50
115	691	37.76	37.43	35.90	96	1	FAN	REST3	28	50
	692	37.76	37.43	35.92	96	1	FAN	REST3	28	50
	693	37.75	37.41	35.90	96	1	FAN	REST3	28	50
	694	37.75	37.40	35.87	96	1	FAN	REST3	28	50
	695	37.74	37.40	35.85	96	1	FAN	REST3	28	50
	696	37.74	37.40	35.80	98	1	FAN	REST3	28	50
	697	37.75	37.39	35.76	95	1	FAN	REST3	28	50
	698	37.78	37.37	35.75	95	1	FAN	REST3	28	50
	699	37.79	37.37	35.73	96	1	FAN	REST3	28	50
	700	37.78	37.38	35.70	100	1	FAN	REST3	28	50
	701	37.75	37.39	35.69	98	1	FAN	REST3	28	50
	702	37.73	37.38	35.70	101	1	FAN	REST3	28	50
	703	37.71	37.36	35.70	99	1	FAN	REST3	28	50
	704	37.71	37.34	35.72	98	1	FAN	REST3	28	50
	705	37.72	37.33	35.72	94	1	FAN	REST3	28	50
	706	37.72	37.34	35.71	95	1	FAN	REST3	28	50
	707	37.75	37.33	35.72	93	1	FAN	REST3	28	50
	708	37.77	37.33	35.74	96	1	FAN	REST3	28	50
	709	37.77	37.33	35.75	94	1	FAN	REST3	28	50
0	1	36.86	36.64	35.21	80	2	FAN	REST1	28	50
	2	36.87	36.65	35.22	84	2	FAN	REST1	28	50
	3	36.87	36.67	35.22	84	2	FAN	REST1	28	50
	4	36.86	36.69	35.22	83	2	FAN	REST1	28	50
	5	36.86	36.68	35.23	86	2	FAN	REST1	28	50
	6	36.86	36.67	35.23	83	2	FAN	REST1	28	50
	7	36.86	36.67	35.23	88	2	FAN	REST1	28	50

min	number	Tre	Tes	Tsk-head	HR	ID	condition	period	Ta	RH
	8	36.86	36.66	35.22	89	2	FAN	REST1	28	50
	9	36.85	36.64	35.23	81	2	FAN	REST1	28	50
	10	36.86	36.65	35.24	81	2	FAN	REST1	28	50
	11	36.87	36.68	35.24	82	2	FAN	REST1	28	50
	12	36.86	36.67	35.25	80	2	FAN	REST1	28	50
	13	36.85	36.66	35.25	80	2	FAN	REST1	28	50
	14	36.85	36.67	35.26	82	2	FAN	REST1	28	50
	15	36.85	36.68	35.24	82	2	FAN	REST1	28	50
	16	36.84	36.67	35.23	80	2	FAN	REST1	28	50
	17	36.84	36.67	35.22	84	2	FAN	REST1	28	50
	18	36.84	36.69	35.22	82	2	FAN	REST1	28	50
	19	36.84	36.70	35.22	81	2	FAN	REST1	28	50
	20	36.84	36.70		81	2	FAN	REST1	28	50
	21	36.83	36.70		79	2	FAN	REST1	28	50
	22	36.83	36.69		83	2	FAN	REST1	28	50
	23	36.83	36.70	34.45	88	2	FAN	REST1	28	50
	24	36.83	36.70	34.98	87	2	FAN	REST1	28	50
	25	36.82	36.69	35.13	81	2	FAN	REST1	28	50
	26	36.82	36.69	35.19	89	2	FAN	REST1	28	50
	27	36.83	36.69	35.25	86	2	FAN	REST1	28	50
	28	36.83	36.69	35.31	84	2	FAN	REST1	28	50
	29	36.83	36.70	35.36	83	2	FAN	REST1	28	50
	30	36.83	36.70	35.38	80	2	FAN	REST1	28	50
5	31	36.82	36.68	35.40	82	2	FAN	REST1	28	50
	32	36.81	36.67	35.42	83	2	FAN	REST1	28	50
	33	36.81	36.68	35.42	82	2	FAN	REST1	28	50
	34	36.82	36.67	35.45	79	2	FAN	REST1	28	50
	35	36.82	36.66	35.46	74	2	FAN	REST1	28	50
	36	36.81	36.68	35.46	79	2	FAN	REST1	28	50
	37	36.81	36.70	35.46	77	2	FAN	REST1	28	50
	38	36.81	36.71	35.44	79	2	FAN	REST1	28	50
	39	36.81	36.70	35.43	78	2	FAN	REST1	28	50
	40	36.81	36.69	35.41	80	2	FAN	REST1	28	50
	41	36.81	36.70	35.41	81	2	FAN	REST1	28	50
	42	36.81	36.71	35.44	78	2	FAN	REST1	28	50
	43	36.81	36.69	35.46	80	2	FAN	REST1	28	50
	44	36.81	36.67	35.46	73	2	FAN	REST1	28	50
	45	36.80	36.66	35.45	73	2	FAN	REST1	28	50
	46	36.81	36.67	35.46	76	2	FAN	REST1	28	50
	47	36.81	36.66	35.44	74	2	FAN	REST1	28	50
	48	36.80	36.65	35.43	75	2	FAN	REST1	28	50
	49	36.79	36.67	35.43	80	2	FAN	REST1	28	50
	50	36.78	36.68	35.44	77	2	FAN	REST1	28	50
	51	36.78	36.68	35.46	77	2	FAN	REST1	28	50
	52	36.78	36.66	35.47	75	2	FAN	REST1	28	50
	53	36.77	36.66	35.47	76	2	FAN	REST1	28	50

min	number	Tre	Tes	Tsk-head	HR	ID	condition	period	Ta	RH
	54	36.77	36.67	35.48	76	2	FAN	REST1	28	50
	55	36.77	36.67	35.47	75	2	FAN	REST1	28	50
	56	36.78	36.65	35.46	75	2	FAN	REST1	28	50
	57	36.77	36.63	35.45	80	2	FAN	REST1	28	50
	58	36.78	36.66	35.44	84	2	FAN	REST1	28	50
	59	36.79	36.66	35.45	74	2	FAN	REST1	28	50
	60	36.79	36.66	35.45	75	2	FAN	REST1	28	50
10	61	36.79	36.67	35.44	76	2	FAN	REST1	28	50
	62	36.79	36.68	35.44	76	2	FAN	REST1	28	50
	63	36.79	36.69	35.46	76	2	FAN	REST1	28	50
	64	36.79	36.67	35.47	75	2	FAN	REST1	28	50
	65	36.79	36.67	35.47	72	2	FAN	REST1	28	50
	66	36.79	36.66	35.47	73	2	FAN	REST1	28	50
	67	36.79	36.66	35.47	77	2	FAN	REST1	28	50
	68	36.78	36.68	35.47	77	2	FAN	REST1	28	50
	69	36.78	36.70	35.45	76	2	FAN	REST1	28	50
	70	36.78	36.70	35.44	76	2	FAN	REST1	28	50
	71	36.78	36.70	35.44	77	2	FAN	REST1	28	50
	72	36.77	36.69	35.45	75	2	FAN	REST1	28	50
	73	36.76	36.66	35.44	74	2	FAN	REST1	28	50
	74	36.77	36.68	35.45	76	2	FAN	REST1	28	50
	75	36.78	36.70	35.47	80	2	FAN	REST1	28	50
	76	36.77	36.70	35.47	86	2	FAN	REST1	28	50
	77	36.77	36.68	35.49	79	2	FAN	REST1	28	50
	78	36.78	36.69	35.49	76	2	FAN	REST1	28	50
	79	36.78	36.71	35.48	76	2	FAN	REST1	28	50
	80	36.77	36.68	35.45	76	2	FAN	REST1	28	50
	81	36.77	36.69	35.43	77	2	FAN	REST1	28	50
	82	36.77	36.70	35.41	77	2	FAN	REST1	28	50
	83	36.78	36.69	35.41	80	2	FAN	REST1	28	50
	84	36.77	36.66	35.42	84	2	FAN	REST1	28	50
	85	36.77	36.66	35.44	76	2	FAN	REST1	28	50
	86	36.77	36.69	35.44	73	2	FAN	REST1	28	50
	87	36.76	36.70	35.44	74	2	FAN	REST1	28	50
	88	36.76	36.69	35.42	75	2	FAN	REST1	28	50
	89	36.77	36.69	35.42	76	2	FAN	REST1	28	50
	90	36.77	36.70	35.44	79	2	FAN	REST1	28	50
15	91	36.77	36.71	35.44	78	2	FAN	REST1	28	50
	92	36.77	36.69	35.44	77	2	FAN	REST1	28	50
	93	36.77	36.69	35.45	76	2	FAN	REST1	28	50
	94	36.78	36.68	35.44	83	2	FAN	REST1	28	50
	95	36.77	36.66	35.43	77	2	FAN	REST1	28	50
	96	36.76	36.66	35.42	77	2	FAN	REST1	28	50
	97	36.76	36.65	35.42	74	2	FAN	REST1	28	50
	98	36.77	36.66	35.41	76	2	FAN	REST1	28	50
	99	36.77	36.67	35.41	76	2	FAN	REST1	28	50

min	number	Tre	Tes	Tsk-head	HR	ID	condition	period	Ta	RH
	100	36.76	36.67	35.41	88	2	FAN	REST1	28	50
	101	36.76	36.67	35.43	102	2	FAN	REST1	28	50
	102	36.76	36.66	35.44	85	2	FAN	REST1	28	50
	103	36.76	36.65	35.45	81	2	FAN	REST1	40	50
	104	36.76	36.65	35.57	98	2	FAN	REST1	40	50
	105	36.76	36.64	35.75	91	2	FAN	REST1	40	50
	106	36.76	36.63	35.87	89	2	FAN	REST1	40	50
	107	36.76	36.64	35.94	81	2	FAN	REST1	40	50
	108	36.76	36.66	36.00	89	2	FAN	REST1	40	50
	109	36.76	36.67	36.06	81	2	FAN	REST1	40	50
	110	36.76	36.67	36.10	87	2	FAN	REST1	40	50
	111	36.76	36.67	36.16	99	2	FAN	REST1	40	50
	112	36.76	36.62	36.19	87	2	FAN	REST1	40	50
	113	36.74	36.59	36.20	86	2	FAN	REST1	40	50
	114	36.73	36.61	36.22	105	2	FAN	REST1	40	50
	115	36.73	36.64	36.25	86	2	FAN	REST1	40	50
	116	36.73	36.65	36.27	88	2	FAN	REST1	40	50
	117	36.73	36.65	36.28	86	2	FAN	REST1	40	50
	118	36.73	36.66	36.30	84	2	FAN	REST1	40	50
	119	36.73	36.65	36.32	84	2	FAN	REST1	40	50
	120	36.74	36.66	36.33	92	2	FAN	REST1	40	50
20	121	36.73	36.67	36.34	88	2	FAN	REST1	40	50
	122	36.74	36.67	36.36	88	2	FAN	REST1	40	50
	123	36.74	36.68	36.36	88	2	FAN	REST1	40	50
	124	36.74	36.66	36.37	95	2	FAN	REST1	40	50
	125	36.74	36.64	36.39	86	2	FAN	REST1	40	50
	126	36.75	36.66	36.40	87	2	FAN	REST1	40	50
	127	36.75	36.68	36.41	89	2	FAN	REST1	40	50
	128	36.75	36.68	36.42	92	2	FAN	REST1	40	50
	129	36.75	36.68	36.44	94	2	FAN	REST1	40	50
	130	36.75	36.66	36.45	89	2	FAN	REST1	40	50
	131	36.75	36.66	36.46	79	2	FAN	REST1	40	50
	132	36.75	36.68	36.47	79	2	FAN	REST1	40	50
	133	36.75	36.69	36.48	87	2	FAN	REST1	40	50
	134	36.76	36.69	36.48	101	2	FAN	REST1	40	50
	135	36.76	36.67	36.49	91	2	FAN	REST1	40	50
	136	36.77	36.68	36.51	95	2	FAN	REST1	40	50
	137	36.78	36.72	36.52	92	2	FAN	REST1	40	50
	138	36.78	36.72	36.52	91	2	FAN	REST1	40	50
	139	36.78	36.71	36.53	87	2	FAN	EXERCISE1	40	50
	140	36.78	36.70	36.54	92	2	FAN	EXERCISE1	40	50
	141	36.78	36.68	36.56	99	2	FAN	EXERCISE1	40	50
	142	36.78	36.65	36.57	107	2	FAN	EXERCISE1	40	50
	143	36.78	36.66	36.57	108	2	FAN	EXERCISE1	40	50
	144	36.77	36.69	36.60	109	2	FAN	EXERCISE1	40	50
	145	36.77	36.69	36.62	107	2	FAN	EXERCISE1	40	50

min	number	Tre	Tes	Tsk-head	HR	ID	condition	period	Ta	RH
	146	36.77	36.69	36.60	110	2	FAN	EXERCISE1	40	50
	147	36.77	36.68	36.59	109	2	FAN	EXERCISE1	40	50
	148	36.77	36.70	36.61	110	2	FAN	EXERCISE1	40	50
	149	36.79	36.71	36.62	112	2	FAN	EXERCISE1	40	50
	150	36.79	36.70	36.63	112	2	FAN	EXERCISE1	40	50
25	151	36.78	36.72	36.64	110	2	FAN	EXERCISE1	40	50
	152	36.79	36.70	36.64	111	2	FAN	EXERCISE1	40	50
	153	36.79	36.67	36.65	112	2	FAN	EXERCISE1	40	50
	154	36.78	36.68	36.65	114	2	FAN	EXERCISE1	40	50
	155	36.79	36.70	36.65	113	2	FAN	EXERCISE1	40	50
	156	36.80	36.71	36.67	114	2	FAN	EXERCISE1	40	50
	157	36.80	36.72	36.69	112	2	FAN	EXERCISE1	40	50
	158	36.80	36.72	36.68	110	2	FAN	EXERCISE1	40	50
	159	36.80	36.74	36.69	113	2	FAN	EXERCISE1	40	50
	160	36.80	36.74	36.70	113	2	FAN	EXERCISE1	40	50
	161	36.80	36.75	36.70	113	2	FAN	EXERCISE1	40	50
	162	36.80	36.75	36.70	114	2	FAN	EXERCISE1	40	50
	163	36.80	36.74	36.70	114	2	FAN	EXERCISE1	40	50
	164	36.80	36.76	36.70	113	2	FAN	EXERCISE1	40	50
	165	36.81	36.78	36.70	115	2	FAN	EXERCISE1	40	50
	166	36.82	36.77	36.71	116	2	FAN	EXERCISE1	40	50
	167	36.82	36.75	36.72	112	2	FAN	EXERCISE1	40	50
	168	36.82	36.77	36.73	110	2	FAN	EXERCISE1	40	50
	169	36.83	36.81	36.74	112	2	FAN	EXERCISE1	40	50
	170	36.83	36.82	36.75	113	2	FAN	EXERCISE1	40	50
	171	36.82	36.81	36.75	115	2	FAN	EXERCISE1	40	50
	172	36.81	36.82	36.74	115	2	FAN	EXERCISE1	40	50
	173	36.81	36.84	36.75	114	2	FAN	EXERCISE1	40	50
	174	36.82	36.85	36.76	111	2	FAN	EXERCISE1	40	50
	175	36.82	36.85	36.77	113	2	FAN	EXERCISE1	40	50
	176	36.81	36.84	36.77	113	2	FAN	EXERCISE1	40	50
	177	36.81	36.88	36.77	109	2	FAN	EXERCISE1	40	50
	178	36.82	36.90	36.77	114	2	FAN	EXERCISE1	40	50
	179	36.81	36.90	36.77	117	2	FAN	EXERCISE1	40	50
	180	36.80	36.88	36.78	117	2	FAN	EXERCISE1	40	50
30	181	36.81	36.89	36.79	119	2	FAN	EXERCISE1	40	50
	182	36.82	36.89	36.79	118	2	FAN	EXERCISE1	40	50
	183	36.82	36.87	36.81	121	2	FAN	EXERCISE1	40	50
	184	36.82	36.87	36.82	120	2	FAN	EXERCISE1	40	50
	185	36.81	36.88	36.81	115	2	FAN	EXERCISE1	40	50
	186	36.82	36.89	36.82	117	2	FAN	EXERCISE1	40	50
	187	36.84	36.87	36.82	117	2	FAN	EXERCISE1	40	50
	188	36.84	36.87	36.82	115	2	FAN	EXERCISE1	40	50
	189	36.83	36.90	36.83	118	2	FAN	EXERCISE1	40	50
	190	36.83	36.92	36.84	118	2	FAN	EXERCISE1	40	50
	191	36.84	36.89	36.84	118	2	FAN	EXERCISE1	40	50

min	number	Tre	Tes	Tsk-head	HR	ID	condition	period	Ta	RH
	192	36.86	36.88	36.84	123	2	FAN	EXERCISE1	40	50
	193	36.86	36.91	36.85	117	2	FAN	EXERCISE1	40	50
	194	36.86	36.91	36.84	118	2	FAN	EXERCISE1	40	50
	195	36.86	36.89	36.84	122	2	FAN	EXERCISE1	40	50
	196	36.86	36.92	36.84	118	2	FAN	EXERCISE1	40	50
	197	36.87	36.94	36.85	117	2	FAN	EXERCISE1	40	50
	198	36.87	36.93	36.85	120	2	FAN	EXERCISE1	40	50
	199	36.87	36.96	36.85	122	2	FAN	EXERCISE1	40	50
	200	36.87	36.97	36.86	123	2	FAN	EXERCISE1	40	50
	201	36.87	36.96	36.86	121	2	FAN	EXERCISE1	40	50
	202	36.87	36.99	36.85	115	2	FAN	EXERCISE1	40	50
	203	36.87	37.00	36.84	118	2	FAN	EXERCISE1	40	50
	204	36.88	37.01	36.84	122	2	FAN	EXERCISE1	40	50
	205	36.88	37.02	36.85	120	2	FAN	EXERCISE1	40	50
	206	36.88	37.02	36.84	121	2	FAN	EXERCISE1	40	50
	207	36.89	36.98	36.83	126	2	FAN	EXERCISE1	40	50
	208	36.89	36.96	36.83	123	2	FAN	EXERCISE1	40	50
	209	36.89	37.02	36.83	122	2	FAN	EXERCISE1	40	50
	210	36.88	37.02	36.83	124	2	FAN	EXERCISE1	40	50
35	211	36.87	37.01	36.83	125	2	FAN	EXERCISE1	40	50
	212	36.87	37.02	36.84	124	2	FAN	EXERCISE1	40	50
	213	36.88	37.03	36.84	122	2	FAN	EXERCISE1	40	50
	214	36.88	37.02	36.85	121	2	FAN	EXERCISE1	40	50
	215	36.87	37.03	36.84	121	2	FAN	EXERCISE1	40	50
	216	36.88	37.03	36.83	120	2	FAN	EXERCISE1	40	50
	217	36.90	37.04	36.83	119	2	FAN	EXERCISE1	40	50
	218	36.91	37.06	36.84	122	2	FAN	EXERCISE1	40	50
	219	36.91	37.08	36.84	121	2	FAN	EXERCISE1	40	50
	220	36.91	37.09	36.83	121	2	FAN	EXERCISE1	40	50
	221	36.92	37.10	36.83	123	2	FAN	EXERCISE1	40	50
	222	36.92	37.10	36.83	124	2	FAN	EXERCISE1	40	50
	223	36.91	37.08	36.83	124	2	FAN	EXERCISE1	40	50
	224	36.91	37.10	36.83	125	2	FAN	EXERCISE1	40	50
	225	36.92	37.13	36.83	125	2	FAN	EXERCISE1	40	50
	226	36.92	37.11	36.83	126	2	FAN	EXERCISE1	40	50
	227	36.93	37.10	36.83	124	2	FAN	EXERCISE1	40	50
	228	36.92	37.09	36.84	123	2	FAN	EXERCISE1	40	50
	229	36.92	37.09	36.84	122	2	FAN	EXERCISE1	40	50
	230	36.92	37.13	36.86	120	2	FAN	EXERCISE1	40	50
	231	36.92	37.14	36.86	122	2	FAN	EXERCISE1	40	50
	232	36.92	37.13	36.87	124	2	FAN	EXERCISE1	40	50
	233	36.93	37.11	36.88	123	2	FAN	EXERCISE1	40	50
	234	36.93	37.11	36.89	123	2	FAN	EXERCISE1	40	50
	235	36.92	37.14	36.90	124	2	FAN	EXERCISE1	40	50
	236	36.92	37.14	36.90	125	2	FAN	EXERCISE1	40	50
	237	36.92	37.13	36.91	125	2	FAN	EXERCISE1	40	50

min	number	Tre	Tes	Tsk-head	HR	ID	condition	period	Ta	RH
	238	36.93	37.13	36.92	125	2	FAN	EXERCISE1	40	50
	239	36.94	37.12	36.91	117	2	FAN	EXERCISE1	40	50
	240	36.94	37.11	36.90	120	2	FAN	EXERCISE1	40	50
40	241	36.94	37.15	36.90	120	2	FAN	EXERCISE1	40	50
	242	36.93	37.14	36.88	125	2	FAN	EXERCISE1	40	50
	243	36.93	37.13	36.87	126	2	FAN	EXERCISE1	40	50
	244	36.93	37.14	36.87	127	2	FAN	EXERCISE1	40	50
	245	36.93	37.16	36.88	127	2	FAN	EXERCISE1	40	50
	246	36.93	37.17	36.89	126	2	FAN	EXERCISE1	40	50
	247	36.94	37.18	36.90	124	2	FAN	EXERCISE1	40	50
	248	36.94	37.20	36.89	121	2	FAN	EXERCISE1	40	50
	249	36.93	37.20	36.89	121	2	FAN	EXERCISE1	40	50
	250	36.94	37.18	36.90	127	2	FAN	EXERCISE1	40	50
	251	36.94	37.09	36.93	125	2	FAN	EXERCISE1	40	50
	252	36.94	37.10	36.93	123	2	FAN	EXERCISE1	40	50
	253	36.94	37.14	36.92	119	2	FAN	EXERCISE1	40	50
	254	36.94	37.13	36.93	119	2	FAN	EXERCISE1	40	50
	255	36.94	37.15	36.93	122	2	FAN	EXERCISE1	40	50
	256	36.94	37.15	36.92	126	2	FAN	EXERCISE1	40	50
	257	36.96	37.15	36.92	128	2	FAN	EXERCISE1	40	50
	258	36.97	37.19	36.94	126	2	FAN	EXERCISE1	40	50
	259	36.98	37.14	36.95	129	2	FAN	EXERCISE1	40	50
	260	36.97	37.13	36.93	127	2	FAN	EXERCISE1	40	50
	261	36.97	37.19	36.93	128	2	FAN	EXERCISE1	40	50
	262	36.97	37.21	36.94	125	2	FAN	EXERCISE1	40	50
	263	36.98	37.23	36.94	126	2	FAN	EXERCISE1	40	50
	264	36.98	37.22	36.94	124	2	FAN	EXERCISE1	40	50
	265	36.97	37.22	36.93	130	2	FAN	EXERCISE1	40	50
	266	36.97	37.20	36.93	126	2	FAN	EXERCISE1	40	50
	267	36.97	37.18	36.93	126	2	FAN	EXERCISE1	40	50
	268	36.98	37.19	36.93	127	2	FAN	EXERCISE1	40	50
	269	36.98	37.19	36.93	131	2	FAN	EXERCISE1	40	50
	270	36.99	37.19	36.93	134	2	FAN	EXERCISE1	40	50
45	271	36.99	37.22	36.93	133	2	FAN	EXERCISE1	40	50
	272	36.98	37.23	36.93	130	2	FAN	EXERCISE1	40	50
	273	36.98	37.21	36.93	131	2	FAN	EXERCISE1	40	50
	274	36.99	37.21	36.93	131	2	FAN	EXERCISE1	40	50
	275	37.00	37.25	36.94	131	2	FAN	EXERCISE1	40	50
	276	36.99	37.24	36.92	128	2	FAN	EXERCISE1	40	50
	277	36.99	37.24	36.92	129	2	FAN	EXERCISE1	40	50
	278	37.00	37.25	36.94	128	2	FAN	EXERCISE1	40	50
	279	37.00	37.25	36.94	125	2	FAN	EXERCISE1	40	50
	280	37.00	37.24	36.94	126	2	FAN	EXERCISE1	40	50
	281	37.00	37.25	36.94	126	2	FAN	EXERCISE1	40	50
	282	37.00	37.26	36.95	125	2	FAN	EXERCISE1	40	50
	283	37.00	37.27	36.96	126	2	FAN	EXERCISE1	40	50

min	number	Tre	Tes	Tsk-head	HR	ID	condition	period	Ta	RH
	284	37.00	37.27	36.96	129	2	FAN	EXERCISE1	40	50
	285	37.01	37.27	36.95	128	2	FAN	EXERCISE1	40	50
	286	37.02	37.29	36.95	131	2	FAN	EXERCISE1	40	50
	287	37.02	37.26	36.95	132	2	FAN	EXERCISE1	40	50
	288	37.03	37.28	36.97	130	2	FAN	EXERCISE1	40	50
	289	37.03	37.29	36.97	131	2	FAN	EXERCISE1	40	50
	290	37.03	37.27	36.97	131	2	FAN	EXERCISE1	40	50
	291	37.04	37.30	36.97	132	2	FAN	EXERCISE1	40	50
	292	37.03	37.15	36.97	136	2	FAN	EXERCISE1	40	50
	293	37.03	37.12	36.98	131	2	FAN	EXERCISE1	40	50
	294	37.03	37.24	36.98	128	2	FAN	EXERCISE1	40	50
	295	37.02	37.26	36.96	128	2	FAN	EXERCISE1	40	50
	296	37.03	37.28	36.97	129	2	FAN	EXERCISE1	40	50
	297	37.04	37.27	36.97	130	2	FAN	EXERCISE1	40	50
	298	37.04	37.23	36.97	131	2	FAN	EXERCISE1	40	50
	299	37.04	37.22	36.96	128	2	FAN	EXERCISE1	40	50
	300	37.04	37.29	36.96	128	2	FAN	EXERCISE1	40	50
50	301	37.05	37.31	36.97	128	2	FAN	EXERCISE1	40	50
	302	37.05	37.32	36.97	128	2	FAN	EXERCISE1	40	50
	303	37.05	37.35	36.96	129	2	FAN	EXERCISE1	40	50
	304	37.06	37.34	36.96	124	2	FAN	EXERCISE1	40	50
	305	37.07	37.34	36.97	128	2	FAN	EXERCISE1	40	50
	306	37.06	37.34	36.97	129	2	FAN	EXERCISE1	40	50
	307	37.06	37.34	36.97	130	2	FAN	EXERCISE1	40	50
	308	37.07	37.35	36.98	134	2	FAN	EXERCISE1	40	50
	309	37.07	37.35	36.98	134	2	FAN	EXERCISE1	40	50
	310	37.07	37.35	36.97	134	2	FAN	EXERCISE1	40	50
	311	37.07	37.36	36.96	134	2	FAN	EXERCISE1	40	50
	312	37.07	37.34	36.97	132	2	FAN	EXERCISE1	40	50
	313	37.08	37.33	36.98	129	2	FAN	EXERCISE1	40	50
	314	37.07	37.33	36.98	133	2	FAN	EXERCISE1	40	50
	315	37.07	37.30	36.98	136	2	FAN	EXERCISE1	40	50
	316	37.07	37.26	36.99	134	2	FAN	EXERCISE1	40	50
	317	37.07	37.27	36.99	131	2	FAN	EXERCISE1	40	50
	318	37.07	37.29	36.99	133	2	FAN	EXERCISE1	40	50
	319	37.07	37.27	36.99	131	2	FAN	EXERCISE1	40	50
	320	37.08	37.25	37.01	130	2	FAN	REST2	28	50
	321	37.09	37.23	37.01	128	2	FAN	REST2	28	50
	322	37.09	37.26	36.87	129	2	FAN	REST2	28	50
	323	37.08	37.31	36.65	117	2	FAN	REST2	28	50
	324	37.07	37.36	36.47	121	2	FAN	REST2	28	50
	325	37.08	37.40	36.31	114	2	FAN	REST2	28	50
	326	37.08	37.29	36.21	110	2	FAN	REST2	28	50
	327	37.08	37.23	36.11	95	2	FAN	REST2	28	50
	328	37.09	37.28	36.03	112	2	FAN	REST2	28	50
	329	37.10	37.36	35.96	109	2	FAN	REST2	28	50

min	number	Tre	Tes	Tsk-head	HR	ID	condition	period	Ta	RH
	330	37.10	37.37	35.91	106	2	FAN	REST2	28	50
55	331	37.10	37.36	35.87	98	2	FAN	REST2	28	50
	332	37.10	37.34	35.87	101	2	FAN	REST2	28	50
	333	37.09	37.21	35.84	106	2	FAN	REST2	28	50
	334	37.09	37.19	35.79	103	2	FAN	REST2	28	50
	335	37.09	37.16	35.76	104	2	FAN	REST2	28	50
	336	37.09	37.14	35.74	104	2	FAN	REST2	28	50
	337	37.09	37.20	35.73	98	2	FAN	REST2	28	50
	338	37.10	37.23	35.67	103	2	FAN	REST2	28	50
	339	37.10	31.50	35.62	119	2	FAN	REST2	28	50
	340	37.10	27.80	35.57	113	2	FAN	REST2	28	50
	341	37.10	30.77	35.50	108	2	FAN	REST2	28	50
	342	37.10	31.51	35.51	102	2	FAN	REST2	28	50
	343	37.10	31.53	35.56	103	2	FAN	REST2	28	50
	344	37.10	31.23	35.56	102	2	FAN	REST2	28	50
	345	37.10	32.21	35.56	100	2	FAN	REST2	28	50
	346	37.09	32.24	35.52	102	2	FAN	REST2	28	50
	347	37.08	31.15	35.51	96	2	FAN	REST2	28	50
	348	37.08	31.85	35.51	98	2	FAN	REST2	28	50
	349	37.08	33.60	35.50	103	2	FAN	REST2	28	50
	350	37.08	33.43	35.47	100	2	FAN	REST2	28	50
	351	37.08	32.74	35.44	100	2	FAN	REST2	28	50
	352	37.08	31.09	35.42	104	2	FAN	REST2	28	50
	353	37.08	29.50	35.41	104	2	FAN	REST2	28	50
	354	37.08	30.44	35.46	91	2	FAN	REST2	28	50
	355	37.08	30.65	35.52	89	2	FAN	REST2	28	50
	356	37.08	30.90	35.59	97	2	FAN	REST2	28	50
	357	37.08	31.28	35.62	88	2	FAN	REST2	28	50
	358	37.08	31.65	35.59	93	2	FAN	REST2	28	50
	359	37.08	29.93	35.53	96	2	FAN	REST2	28	50
	360	37.07	29.15	35.49	91	2	FAN	REST2	28	50
60	361	37.07	30.20	35.48	92	2	FAN	REST2	28	50
	362	37.08	30.15	35.50	84	2	FAN	REST2	28	50
	363	37.08	30.40	35.48	85	2	FAN	REST2	28	50
	364	37.08	31.94	35.46	93	2	FAN	REST2	28	50
	365	37.08	29.60	35.42	103	2	FAN	REST2	28	50
	366	37.09	27.90	35.39	79	2	FAN	REST2	28	50
	367	37.09	29.74	35.42	89	2	FAN	REST2	28	50
	368	37.09	30.12	35.44	86	2	FAN	REST2	28	50
	369	37.09	30.69	35.45	92	2	FAN	REST2	28	50
	370	37.09	28.47	35.40	99	2	FAN	REST2	28	50
	371	37.08	27.37	35.36	92	2	FAN	REST2	28	50
	372	37.07	29.10	35.38	90	2	FAN	REST2	28	50
	373	37.07	29.75	35.39	93	2	FAN	REST2	28	50
	374	37.07	30.60	35.40	90	2	FAN	REST2	28	50
	375	37.06	28.18	35.40	97	2	FAN	REST2	28	50

min	number	Tre	Tes	Tsk-head	HR	ID	condition	period	Ta	RH
	376	37.05	27.18	35.37	85	2	FAN	REST2	28	50
	377	37.05	28.88	35.31	100	2	FAN	REST2	28	50
	378	37.04	28.98	35.25	98	2	FAN	REST2	28	50
	379	37.04	29.70	35.20	93	2	FAN	REST2	28	50
	380	37.05	29.86	35.20	101	2	FAN	REST2	28	50
	381	37.05	29.77	35.24	85	2	FAN	REST2	28	50
	382	37.05	29.94	35.29	83	2	FAN	REST2	28	50
	383	37.05	30.13	35.32	92	2	FAN	REST2	28	50
	384	37.05	31.05	35.34	86	2	FAN	REST2	28	50
	385	37.06	31.27	35.35	74	2	FAN	REST2	28	50
	386	37.05	30.45	35.35	90	2	FAN	REST2	28	50
	387	37.05	30.48	35.34	86	2	FAN	REST2	28	50
	388	37.04	30.54	35.34	91	2	FAN	REST2	28	50
	389	37.03	30.42	35.35	85	2	FAN	REST2	28	50
	390	37.04	30.46	35.35	84	2	FAN	REST2	28	50
65	391	37.03	30.58	35.31	85	2	FAN	REST2	28	50
	392	37.03	30.71	35.28	87	2	FAN	REST2	28	50
	393	37.04	30.85	35.28	84	2	FAN	REST2	28	50
	394	37.04	30.87	35.29	83	2	FAN	REST2	28	50
	395	37.03	30.97	35.27	94	2	FAN	REST2	28	50
	396	37.02	31.50	35.19	91	2	FAN	REST2	28	50
	397	37.02	31.59	35.17	92	2	FAN	REST2	28	50
	398	37.02	31.32	35.20	93	2	FAN	REST2	28	50
	399	37.01	31.37	35.24	91	2	FAN	REST2	28	50
	400	37.02	31.32	35.28	85	2	FAN	REST2	28	50
	401	37.02	31.27	35.29	79	2	FAN	REST2	28	50
	402	37.01	31.45	35.32	79	2	FAN	REST2	28	50
	403	37.01	31.56	35.36	79	2	FAN	REST2	28	50
	404	37.00	31.63	35.38	81	2	FAN	REST2	28	50
	405	37.01	31.67	35.39	82	2	FAN	REST2	28	50
	406	37.02	31.71	35.40	85	2	FAN	REST2	28	50
	407	37.02	31.77	35.43	81	2	FAN	REST2	28	50
	408	37.01	31.85	35.44	79	2	FAN	REST2	28	50
	409	37.00	32.00	35.47	82	2	FAN	REST2	28	50
	410	37.01	32.11	35.50	84	2	FAN	REST2	28	50
	411	37.00	32.18	35.51	79	2	FAN	REST2	28	50
	412	37.00	32.26	35.53	78	2	FAN	REST2	28	50
	413	37.00	32.32	35.55	78	2	FAN	REST2	28	50
	414	37.00	32.41	35.57	78	2	FAN	REST2	28	50
	415	37.00	32.49	35.60	79	2	FAN	REST2	28	50
	416	37.00	32.57	35.61	83	2	FAN	REST2	28	50
	417	37.00	32.63	35.55	96	2	FAN	REST2	28	50
	418	36.99	32.70	35.48	90	2	FAN	REST2	28	50
	419	36.99	32.78	35.45	86	2	FAN	REST2	28	50
	420	36.99	32.86	35.36	89	2	FAN	REST2	28	50
70	421	36.99	33.02	35.28	99	2	FAN	REST2	28	50

min	number	Tre	Tes	Tsk-head	HR	ID	condition	period	Ta	RH
	422	36.99	33.12	35.28	96	2	FAN	REST2	28	50
	423	36.98	33.33	35.27	87	2	FAN	REST2	28	50
	424	36.97	33.47	35.24	93	2	FAN	REST2	28	50
	425	36.98	33.62	35.21	80	2	FAN	REST2	28	50
	426	36.98	33.91	35.23	85	2	FAN	REST2	28	50
	427	36.98	33.92	35.27	78	2	FAN	REST2	28	50
	428	36.98	33.97	35.29	78	2	FAN	REST2	28	50
	429	36.98	34.27	35.30	82	2	FAN	REST2	28	50
	430	36.99	34.40	35.31	89	2	FAN	REST2	28	50
	431	36.99	34.55	35.33	84	2	FAN	REST2	28	50
	432	36.98	35.27	35.34	79	2	FAN	REST2	28	50
	433	36.99	35.97	35.35	86	2	FAN	REST2	28	50
	434	37.00	36.14	35.31	83	2	FAN	REST2	28	50
	435	37.00	36.05	35.27	87	2	FAN	REST2	28	50
	436	37.01	35.84	35.30	83	2	FAN	REST2	28	50
	437	37.00	35.95	35.34	80	2	FAN	REST2	28	50
	438	36.99	36.38	35.36	79	2	FAN	REST2	28	50
	439	36.98	36.57	35.37	79	2	FAN	REST2	40	50
	440	36.98	36.57	35.38	113	2	FAN	REST2	40	50
	441	36.98	36.56	35.56	100	2	FAN	REST2	40	50
	442	36.98	36.59	35.79	93	2	FAN	REST2	40	50
	443	36.99	36.61	35.89	93	2	FAN	REST2	40	50
	444	36.98	36.59	35.96	83	2	FAN	REST2	40	50
	445	36.97	36.58	36.03	80	2	FAN	REST2	40	50
	446	36.98	36.62	36.08	76	2	FAN	REST2	40	50
	447	36.98	36.62	36.13	80	2	FAN	REST2	40	50
	448	36.97	36.61	36.17	83	2	FAN	REST2	40	50
	449	36.97	36.64	36.20	87	2	FAN	REST2	40	50
	450	36.97	36.65	36.20	86	2	FAN	REST2	40	50
75	451	36.97	36.63	36.20	80	2	FAN	REST2	40	50
	452	36.97	36.63	36.21	88	2	FAN	REST2	40	50
	453	36.97	36.65	36.22	81	2	FAN	REST2	40	50
	454	36.97	36.62	36.23	80	2	FAN	REST2	40	50
	455	36.96	36.64	36.25	85	2	FAN	REST2	40	50
	456	36.96	36.66	36.28	90	2	FAN	REST2	40	50
	457	36.97	36.68	36.30	91	2	FAN	REST2	40	50
	458	36.96	36.69	36.30	107	2	FAN	REST2	40	50
	459	36.96	36.67	36.32	98	2	FAN	REST2	40	50
	460	36.96	36.66	36.34	103	2	FAN	REST2	40	50
	461	36.96	36.64	36.36	98	2	FAN	REST2	40	50
	462	36.96	36.65	36.36	98	2	FAN	REST2	40	50
	463	36.96	36.68	36.36	88	2	FAN	EXERCISE2	40	50
	464	36.96	36.70	36.38	83	2	FAN	EXERCISE2	40	50
	465	36.97	36.59	36.40	105	2	FAN	EXERCISE2	40	50
	466	36.98	36.55	36.40	107	2	FAN	EXERCISE2	40	50
	467	36.98	36.07	36.41	111	2	FAN	EXERCISE2	40	50

min	number	Tre	Tes	Tsk-head	HR	ID	condition	period	Ta	RH
	468	36.99	35.82	36.42	107	2	FAN	EXERCISE2	40	50
	469	37.00	36.24	36.44	110	2	FAN	EXERCISE2	40	50
	470	37.00	36.41	36.45	113	2	FAN	EXERCISE2	40	50
	471	36.99	36.47	36.47	115	2	FAN	EXERCISE2	40	50
	472	36.98	36.51	36.48	113	2	FAN	EXERCISE2	40	50
	473	36.98	36.55	36.49	108	2	FAN	EXERCISE2	40	50
	474	36.99		36.51	113	2	FAN	EXERCISE2	40	50
	475	36.99		36.54	112	2	FAN	EXERCISE2	40	50
	476	37.00		36.56	114	2	FAN	EXERCISE2	40	50
	477	37.00		36.56	112	2	FAN	EXERCISE2	40	50
	478	37.00		36.58	114	2	FAN	EXERCISE2	40	50
	479	37.01		36.61	113	2	FAN	EXERCISE2	40	50
	480	37.01	36.77	36.63	115	2	FAN	EXERCISE2	40	50
80	481	37.01	36.78	36.63	116	2	FAN	EXERCISE2	40	50
	482	37.00	36.65	36.62	115	2	FAN	EXERCISE2	40	50
	483	37.00	36.09	36.63	123	2	FAN	EXERCISE2	40	50
	484	37.00	36.01	36.65	117	2	FAN	EXERCISE2	40	50
	485	37.02	36.42	36.66	103	2	FAN	EXERCISE2	40	50
	486	37.02	36.52	36.66	115	2	FAN	EXERCISE2	40	50
	487	37.01	36.56	36.64	100	2	FAN	EXERCISE2	40	50
	488	37.01	36.62	36.65	118	2	FAN	EXERCISE2	40	50
	489	37.02	36.70	36.68	115	2	FAN	EXERCISE2	40	50
	490	37.03	36.76	36.69	119	2	FAN	EXERCISE2	40	50
	491	37.04	36.79	36.71	119	2	FAN	EXERCISE2	40	50
	492	37.04	36.80	36.72	118	2	FAN	EXERCISE2	40	50
	493	37.04	36.82	36.73	117	2	FAN	EXERCISE2	40	50
	494	37.04	36.86	36.75	119	2	FAN	EXERCISE2	40	50
	495	37.04	36.87	36.77	117	2	FAN	EXERCISE2	40	50
	496	37.04	36.90	36.78	116	2	FAN	EXERCISE2	40	50
	497	37.04	36.94	36.79	116	2	FAN	EXERCISE2	40	50
	498	37.05	36.94	36.80	117	2	FAN	EXERCISE2	40	50
	499	37.05	36.95	36.80	120	2	FAN	EXERCISE2	40	50
	500	37.05	36.97	36.80	117	2	FAN	EXERCISE2	40	50
	501	37.06	37.00	36.79	120	2	FAN	EXERCISE2	40	50
	502	37.07	37.02	36.79	125	2	FAN	EXERCISE2	40	50
	503	37.07	37.03	36.80	123	2	FAN	EXERCISE2	40	50
	504	37.07	37.06	36.81	122	2	FAN	EXERCISE2	40	50
	505	37.07	37.07	36.81	120	2	FAN	EXERCISE2	40	50
	506	37.07	37.08	36.81	118	2	FAN	EXERCISE2	40	50
	507	37.07	37.09	36.82	122	2	FAN	EXERCISE2	40	50
	508	37.08	37.00	36.83	123	2	FAN	EXERCISE2	40	50
	509	37.08	36.73	36.83	128	2	FAN	EXERCISE2	40	50
	510	37.09	36.66	36.84	121	2	FAN	EXERCISE2	40	50
85	511	37.09	36.81	36.84	121	2	FAN	EXERCISE2	40	50
	512	37.10	36.89	36.84	122	2	FAN	EXERCISE2	40	50
	513	37.10	36.93	36.84	122	2	FAN	EXERCISE2	40	50

min	number	Tre	Tes	Tsk-head	HR	ID	condition	period	Ta	RH
	514	37.10	36.95	36.83	119	2	FAN	EXERCISE2	40	50
	515	37.09	36.98	36.83	120	2	FAN	EXERCISE2	40	50
	516	37.10	37.01	36.84	119	2	FAN	EXERCISE2	40	50
	517	37.11	37.04	36.84	125	2	FAN	EXERCISE2	40	50
	518	37.10	37.04	36.84	124	2	FAN	EXERCISE2	40	50
	519	37.11	36.88	36.84	128	2	FAN	EXERCISE2	40	50
	520	37.12	36.88	36.84	125	2	FAN	EXERCISE2	40	50
	521	37.11	37.04	36.86	126	2	FAN	EXERCISE2	40	50
	522	37.12	37.06	36.84	121	2	FAN	EXERCISE2	40	50
	523	37.12	37.10	36.79	124	2	FAN	EXERCISE2	40	50
	524	37.12	37.12	36.76	124	2	FAN	EXERCISE2	40	50
	525	37.13	37.13	36.75	123	2	FAN	EXERCISE2	40	50
	526	37.14	37.17	36.75	124	2	FAN	EXERCISE2	40	50
	527	37.14	37.07	36.74	124	2	FAN	EXERCISE2	40	50
	528	37.14	36.80	36.74	127	2	FAN	EXERCISE2	40	50
	529	37.14	36.78	36.75	121	2	FAN	EXERCISE2	40	50
	530	37.14	36.91	36.76	117	2	FAN	EXERCISE2	40	50
	531	37.15	36.95	36.77	124	2	FAN	EXERCISE2	40	50
	532	37.15	37.00	36.78	126	2	FAN	EXERCISE2	40	50
	533	37.15	37.04	36.78	128	2	FAN	EXERCISE2	40	50
	534	37.15	37.06	36.78	127	2	FAN	EXERCISE2	40	50
	535	37.16	37.09	36.78	130	2	FAN	EXERCISE2	40	50
	536	37.16	37.11	36.77	129	2	FAN	EXERCISE2	40	50
	537	37.16	37.11	36.79	129	2	FAN	EXERCISE2	40	50
	538	37.17	37.09	36.80	128	2	FAN	EXERCISE2	40	50
	539	37.16	37.11	36.78	128	2	FAN	EXERCISE2	40	50
	540	37.16	37.14	36.75	129	2	FAN	EXERCISE2	40	50
90	541	37.16	37.15	36.74	128	2	FAN	EXERCISE2	40	50
	542	37.17	37.11	36.74	135	2	FAN	EXERCISE2	40	50
	543	37.17	37.12	36.75	129	2	FAN	EXERCISE2	40	50
	544	37.17	37.20	36.78	129	2	FAN	EXERCISE2	40	50
	545	37.17	37.21	36.78	128	2	FAN	EXERCISE2	40	50
	546	37.17	37.20	36.77	127	2	FAN	EXERCISE2	40	50
	547	37.17	37.23	36.77	127	2	FAN	EXERCISE2	40	50
	548	37.17	37.23	36.78	124	2	FAN	EXERCISE2	40	50
	549	37.17	37.23	36.79	125	2	FAN	EXERCISE2	40	50
	550	37.18	37.21	36.80	130	2	FAN	EXERCISE2	40	50
	551	37.19	37.21	36.81	124	2	FAN	EXERCISE2	40	50
	552	37.18	37.27	36.79	126	2	FAN	EXERCISE2	40	50
	553	37.18	37.29	36.76	127	2	FAN	EXERCISE2	40	50
	554	37.17	37.28	36.75	128	2	FAN	EXERCISE2	40	50
	555	37.17	37.28	36.77	129	2	FAN	EXERCISE2	40	50
	556	37.17	37.21	36.77	130	2	FAN	EXERCISE2	40	50
	557	37.17	37.18	36.78	125	2	FAN	EXERCISE2	40	50
	558	37.18	37.23	36.80	125	2	FAN	EXERCISE2	40	50
	559	37.19	37.27	36.83	126	2	FAN	EXERCISE2	40	50

min	number	Tre	Tes	Tsk-head	HR	ID	condition	period	Ta	RH
	560	37.19	37.29	36.85	126	2	FAN	EXERCISE2	40	50
	561	37.19	37.31	36.86	128	2	FAN	EXERCISE2	40	50
	562	37.20	37.33	36.88	129	2	FAN	EXERCISE2	40	50
	563	37.21	37.32	36.88	126	2	FAN	EXERCISE2	40	50
	564	37.22	37.28	36.88	131	2	FAN	EXERCISE2	40	50
	565	37.22	37.11	36.90	130	2	FAN	EXERCISE2	40	50
	566	37.22	37.00	36.90	134	2	FAN	EXERCISE2	40	50
	567	37.22	37.12	36.91	131	2	FAN	EXERCISE2	40	50
	568	37.22	37.21	36.91	132	2	FAN	EXERCISE2	40	50
	569	37.22	37.24	36.90	130	2	FAN	EXERCISE2	40	50
	570	37.22	37.26	36.88	129	2	FAN	EXERCISE2	40	50
95	571	37.22	37.29	36.86	135	2	FAN	EXERCISE2	40	50
	572	37.22	37.26	36.83	134	2	FAN	EXERCISE2	40	50
	573	37.22	37.22	36.81	131	2	FAN	EXERCISE2	40	50
	574	37.22	37.26	36.81	130	2	FAN	EXERCISE2	40	50
	575	37.22	37.29	36.81	130	2	FAN	EXERCISE2	40	50
	576	37.22	37.30	36.80	131	2	FAN	EXERCISE2	40	50
	577	37.23	37.33	36.79	131	2	FAN	EXERCISE2	40	50
	578	37.23	37.35	36.75	130	2	FAN	EXERCISE2	40	50
	579	37.21	37.36	36.71	133	2	FAN	EXERCISE2	40	50
	580	37.21	37.39	36.65	132	2	FAN	EXERCISE2	40	50
	581	37.22	37.37	36.49	129	2	FAN	EXERCISE2	40	50
	582	37.22	37.37	36.31	131	2	FAN	EXERCISE2	40	50
	583	37.23	37.39	36.24	131	2	FAN	EXERCISE2	40	50
	584	37.24	37.42	36.22	131	2	FAN	EXERCISE2	40	50
	585	37.24	37.42	35.92	122	2	FAN	EXERCISE2	40	50
	586	37.24	37.42	35.80	129	2	FAN	EXERCISE2	40	50
	587	37.24	37.40	35.97	131	2	FAN	EXERCISE2	40	50
	588	37.25	37.37	35.93	131	2	FAN	EXERCISE2	40	50
	589	37.25	37.42	36.11	131	2	FAN	EXERCISE2	40	50
	590	37.25	37.45	36.58	134	2	FAN	EXERCISE2	40	50
	591	37.25	37.42	36.89	131	2	FAN	EXERCISE2	40	50
	592	37.25	37.42	37.03	130	2	FAN	EXERCISE2	40	50
	593	37.25	37.46	37.10	129	2	FAN	EXERCISE2	40	50
	594	37.25	37.40	37.16	133	2	FAN	EXERCISE2	40	50
	595	37.25	37.35	37.20	132	2	FAN	EXERCISE2	40	50
	596	37.25	37.38	37.22	134	2	FAN	EXERCISE2	40	50
	597	37.26	37.34	37.24	134	2	FAN	EXERCISE2	40	50
	598	37.25	37.30	37.25	132	2	FAN	EXERCISE2	40	50
	599	37.25	37.31	37.25	130	2	FAN	EXERCISE2	40	50
	600	37.26	37.31	37.25	129	2	FAN	EXERCISE2	40	50
100	601	37.26	37.34	37.25	129	2	FAN	EXERCISE2	40	50
	602	37.26	37.35	37.26	130	2	FAN	EXERCISE2	40	50
	603	37.27	37.37	37.27	128	2	FAN	EXERCISE2	40	50
	604	37.28	37.41	37.27	131	2	FAN	EXERCISE2	40	50
	605	37.27	37.42	37.28	132	2	FAN	EXERCISE2	40	50

min	number	Tre	Tes	Tsk-head	HR	ID	condition	period	Ta	RH
	606	37.27	37.41	37.29	132	2	FAN	EXERCISE2	40	50
	607	37.27	37.40	37.30	127	2	FAN	EXERCISE2	40	50
	608	37.27	37.41	37.30	129	2	FAN	EXERCISE2	40	50
	609	37.28	37.42	37.29	131	2	FAN	EXERCISE2	40	50
	610	37.29	37.42	37.26	135	2	FAN	EXERCISE2	40	50
	611	37.30	37.36	37.23	136	2	FAN	EXERCISE2	40	50
	612	37.30	37.32	37.22	135	2	FAN	EXERCISE2	40	50
	613	37.29	37.35	37.19	131	2	FAN	EXERCISE2	40	50
	614	37.29	37.37	37.15	132	2	FAN	EXERCISE2	40	50
	615	37.30	37.38	37.10	129	2	FAN	EXERCISE2	40	50
	616	37.31	37.37	37.08	130	2	FAN	EXERCISE2	40	50
	617	37.31	37.39	37.11	132	2	FAN	EXERCISE2	40	50
	618	37.31	37.39	37.09	135	2	FAN	EXERCISE2	40	50
	619	37.31	37.40	37.04	135	2	FAN	EXERCISE2	40	50
	620	37.31	37.42	37.00	134	2	FAN	EXERCISE2	40	50
	621	37.32	37.43	36.99	133	2	FAN	EXERCISE2	40	50
	622	37.32	37.42	37.02	134	2	FAN	EXERCISE2	40	50
	623	37.32	37.43	37.07	134	2	FAN	EXERCISE2	40	50
	624	37.32	37.44	37.04	134	2	FAN	EXERCISE2	40	50
	625	37.33	37.45	36.96	134	2	FAN	EXERCISE2	40	50
	626	37.32	37.44	36.98	135	2	FAN	EXERCISE2	40	50
	627	37.32	37.44	37.06	135	2	FAN	EXERCISE2	40	50
	628	37.33	37.47	37.13	137	2	FAN	EXERCISE2	40	50
	629	37.33	37.46	37.17	138	2	FAN	EXERCISE2	40	50
	630	37.33	37.47	37.16	139	2	FAN	EXERCISE2	40	50
105	631	37.34	37.49	37.12	136	2	FAN	EXERCISE2	40	50
	632	37.34	37.50	37.10	135	2	FAN	EXERCISE2	40	50
	633	37.34	37.29	37.04	140	2	FAN	EXERCISE2	40	50
	634	37.35	37.24	36.94	136	2	FAN	EXERCISE2	40	50
	635	37.35	37.41	36.89	135	2	FAN	EXERCISE2	40	50
	636	37.35	37.44	36.93	134	2	FAN	EXERCISE2	40	50
	637	37.36	37.47	36.97	137	2	FAN	EXERCISE2	40	50
	638	37.36	37.49	36.91	136	2	FAN	EXERCISE2	40	50
	639	37.36	37.49	36.80	135	2	FAN	EXERCISE2	40	50
	640	37.37	37.47	36.74	135	2	FAN	EXERCISE2	40	50
	641	37.38	37.34	36.72	138	2	FAN	EXERCISE2	40	50
	642	37.39	37.35	36.86	136	2	FAN	EXERCISE2	40	50
	643	37.39	37.50	37.08	135	2	FAN	EXERCISE2	40	50
	644	37.38	37.48	37.18	133	2	FAN	REST3	28	50
	645	37.37	37.47	37.13	137	2	FAN	REST3	28	50
	646	37.38	37.50	36.97	130	2	FAN	REST3	28	50
	647	37.39	37.56	36.79	122	2	FAN	REST3	28	50
	648	37.40	37.61	36.65	119	2	FAN	REST3	28	50
	649	37.39	37.61	36.63	111	2	FAN	REST3	28	50
	650	37.38	37.62	36.65	109	2	FAN	REST3	28	50
	651	37.38	37.61	36.46	116	2	FAN	REST3	28	50

min	number	Tre	Tes	Tsk-head	HR	ID	condition	period	Ta	RH
	652	37.39	37.60	36.25	110	2	FAN	REST3	28	50
	653	37.39	37.62	36.21	111	2	FAN	REST3	28	50
	654	37.39	37.63	36.25	107	2	FAN	REST3	28	50
	655	37.39	37.63	36.32	102	2	FAN	REST3	28	50
	656	37.40	37.62	36.31	102	2	FAN	REST3	28	50
	657	37.40	37.60	36.23	103	2	FAN	REST3	28	50
	658	37.40	37.58	36.15	101	2	FAN	REST3	28	50
	659	37.40	37.58	36.10	102	2	FAN	REST3	28	50
	660	37.41	37.58	36.04	101	2	FAN	REST3	28	50
110	661	37.41	37.58	36.01	100	2	FAN	REST3	28	50
	662	37.41	37.55	36.01	108	2	FAN	REST3	28	50
	663	37.41	37.52	36.01	107	2	FAN	REST3	28	50
	664	37.41	37.54	36.02	104	2	FAN	REST3	28	50
	665	37.40	37.54	36.06	95	2	FAN	REST3	28	50
	666	37.40	37.50	36.10	99	2	FAN	REST3	28	50
	667	37.40	37.50	36.13	99	2	FAN	REST3	28	50
	668	37.39	37.32	36.16	105	2	FAN	REST3	28	50
	669	37.38	37.25	36.18	103	2	FAN	REST3	28	50
	670	37.38	37.33	36.17	99	2	FAN	REST3	28	50
	671	37.38	37.36	36.15	96	2	FAN	REST3	28	50
	672	37.38	37.42	36.15	96	2	FAN	REST3	28	50
	673	37.38	37.41	36.14	97	2	FAN	REST3	28	50
	674	37.38	37.36	36.10	99	2	FAN	REST3	28	50
	675	37.38	37.34	36.06	98	2	FAN	REST3	28	50
	676	37.38	37.38	36.02	96	2	FAN	REST3	28	50
	677	37.38	37.35	35.98	94	2	FAN	REST3	28	50
	678	37.38	37.34	35.93	98	2	FAN	REST3	28	50
	679	37.37	37.31	35.90	99	2	FAN	REST3	28	50
	680	37.37	37.31	35.88	96	2	FAN	REST3	28	50
	681	37.37	37.33	35.87	104	2	FAN	REST3	28	50
	682	37.36	37.26	35.87	99	2	FAN	REST3	28	50
	683	37.36	37.25	35.85	102	2	FAN	REST3	28	50
	684	37.37	37.29	35.80	97	2	FAN	REST3	28	50
	685	37.37	37.31	35.80	88	2	FAN	REST3	28	50
	686	37.37	37.18	35.77	99	2	FAN	REST3	28	50
	687	37.37	37.11	35.58	101	2	FAN	REST3	28	50
	688	37.37	37.22	35.12	98	2	FAN	REST3	28	50
	689	37.37	37.20	34.25	92	2	FAN	REST3	28	50
	690	37.37	37.12	34.02	100	2	FAN	REST3	28	50
115	691	37.36	37.18		96	2	FAN	REST3	28	50
	692	37.36	37.25		97	2	FAN	REST3	28	50
	693	37.37	37.24		99	2	FAN	REST3	28	50
	694	37.36	37.13		100	2	FAN	REST3	28	50
	695	37.36	37.10		99	2	FAN	REST3	28	50
	696	37.36	37.14		100	2	FAN	REST3	28	50
	697	37.36	37.14		95	2	FAN	REST3	28	50

min	number	Tre	Tes	Tsk-head	HR	ID	condition	period	Ta	RH
	698	37.35	37.19		90	2	FAN	REST3	28	50
	699	37.35	37.23	33.52	94	2	FAN	REST3	28	50
	700	37.35	37.26	34.07	95	2	FAN	REST3	28	50
	701	37.36	37.26	34.35	96	2	FAN	REST3	28	50
	702	37.37	37.26	34.52	96	2	FAN	REST3	28	50
	703	37.38	37.28	34.67	96	2	FAN	REST3	28	50
	704	37.38	37.27	34.81	94	2	FAN	REST3	28	50
	705	37.38	37.09	34.90	106	2	FAN	REST3	28	50
	706	37.39	37.08	34.98	97	2	FAN	REST3	28	50
	707	37.39	37.26	35.05	94	2	FAN	REST3	28	50
	708	37.42	37.29	35.08	93	2	FAN	REST3	28	50
	709	37.46	37.29	35.12	90	2	FAN	REST3	28	50
0	1	36.72	36.87	35.04	87	3	FAN	REST1	28	50
	2	36.72	36.84	35.06	95	3	FAN	REST1	28	50
	3	36.73	36.84	35.09	87	3	FAN	REST1	28	50
	4	36.73	36.84	35.12	85	3	FAN	REST1	28	50
	5	36.73	36.86	35.14	83	3	FAN	REST1	28	50
	6	36.73	36.87	35.17	78	3	FAN	REST1	28	50
	7	36.74	36.89	35.18	92	3	FAN	REST1	28	50
	8	36.73	36.93	35.20	89	3	FAN	REST1	28	50
	9	36.72	36.93	35.23	92	3	FAN	REST1	28	50
	10	36.72	36.91	35.25	82	3	FAN	REST1	28	50
	11	36.72	36.90	35.27	79	3	FAN	REST1	28	50
	12	36.72	36.90	35.29	88	3	FAN	REST1	28	50
	13	36.73	36.91	35.31	91	3	FAN	REST1	28	50
	14	36.73	36.90	35.33	90	3	FAN	REST1	28	50
	15	36.73	36.89	35.34	91	3	FAN	REST1	28	50
	16	36.73	36.90	35.36	91	3	FAN	REST1	28	50
	17	36.74	36.90	35.36	95	3	FAN	REST1	28	50
	18	36.74	36.90	35.37	87	3	FAN	REST1	28	50
	19	36.74	36.90	35.39	82	3	FAN	REST1	28	50
	20	36.73	36.89	35.40	82	3	FAN	REST1	28	50
	21	36.73	36.89	35.40	84	3	FAN	REST1	28	50
	22	36.73	36.89	35.39	86	3	FAN	REST1	28	50
	23	36.74	36.90	35.42	82	3	FAN	REST1	28	50
	24	36.74	36.91	35.46	80	3	FAN	REST1	28	50
	25	36.73	36.90	35.46	85	3	FAN	REST1	28	50
	26	36.73	36.90	35.44	82	3	FAN	REST1	28	50
	27	36.73	36.91	35.45	84	3	FAN	REST1	28	50
	28	36.73	36.91	35.48	85	3	FAN	REST1	28	50
	29	36.73	36.92	35.49	87	3	FAN	REST1	28	50
	30	36.73	36.92	35.49	79	3	FAN	REST1	28	50
5	31	36.72	36.91	35.50	86	3	FAN	REST1	28	50
	32	36.72	36.91	35.51	82	3	FAN	REST1	28	50
	33	36.73	36.92	35.51	82	3	FAN	REST1	28	50
	34	36.73	36.92	35.51	87	3	FAN	REST1	28	50

min	number	Tre	Tes	Tsk-head	HR	ID	condition	period	Ta	RH
	35	36.73	36.92	35.53	78	3	FAN	REST1	28	50
	36	36.73	36.93	35.55	81	3	FAN	REST1	28	50
	37	36.73	36.94	35.56	80	3	FAN	REST1	28	50
	38	36.72	36.94	35.57	79	3	FAN	REST1	28	50
	39	36.71	36.94	35.56	82	3	FAN	REST1	28	50
	40	36.71	36.95	35.55	79	3	FAN	REST1	28	50
	41	36.72	36.96	35.58	79	3	FAN	REST1	28	50
	42	36.72	37.00	35.62	86	3	FAN	REST1	28	50
	43	36.72	37.00	35.62	82	3	FAN	REST1	28	50
	44	36.72	36.96	35.59	82	3	FAN	REST1	28	50
	45	36.72	36.97	35.60	80	3	FAN	REST1	28	50
	46	36.72	36.97	35.62	89	3	FAN	REST1	28	50
	47	36.72	36.97	35.61	86	3	FAN	REST1	28	50
	48	36.71	36.97	35.61	81	3	FAN	REST1	28	50
	49	36.71	36.97	35.63	82	3	FAN	REST1	28	50
	50	36.71	36.96	35.62	86	3	FAN	REST1	28	50
	51	36.72	36.96	35.61	84	3	FAN	REST1	28	50
	52	36.72	36.97	35.63	86	3	FAN	REST1	28	50
	53	36.72	36.96	35.65	86	3	FAN	REST1	28	50
	54	36.71	36.96	35.64	89	3	FAN	REST1	28	50
	55	36.70	36.96	35.64	86	3	FAN	REST1	28	50
	56	36.70	36.95	35.66	90	3	FAN	REST1	28	50
	57	36.70	36.97	35.66	80	3	FAN	REST1	28	50
	58	36.71	36.97	35.65	86	3	FAN	REST1	28	50
	59	36.71	36.95	35.67	84	3	FAN	REST1	28	50
	60	36.70	36.95	35.66	83	3	FAN	REST1	28	50
10	61	36.69	36.95	35.66	84	3	FAN	REST1	28	50
	62	36.69	36.95	35.68	84	3	FAN	REST1	28	50
	63	36.70	36.95	35.67	79	3	FAN	REST1	28	50
	64	36.70	36.95	35.68	85	3	FAN	REST1	28	50
	65	36.70	36.96	35.69	85	3	FAN	REST1	28	50
	66	36.69	36.95	35.69	81	3	FAN	REST1	28	50
	67	36.69	36.95	35.69	90	3	FAN	REST1	28	50
	68	36.70	36.95	35.68	84	3	FAN	REST1	28	50
	69	36.70	36.95	35.68	81	3	FAN	REST1	28	50
	70	36.70	36.95	35.70	80	3	FAN	REST1	28	50
	71	36.69	36.94	35.69	78	3	FAN	REST1	28	50
	72	36.69	36.94	35.69	83	3	FAN	REST1	28	50
	73	36.69	36.94	35.70	79	3	FAN	REST1	28	50
	74	36.69	36.94	35.71	84	3	FAN	REST1	28	50
	75	36.69	36.94	35.72	80	3	FAN	REST1	28	50
	76	36.69	36.94	35.70	75	3	FAN	REST1	28	50
	77	36.69	36.94	35.72	77	3	FAN	REST1	28	50
	78	36.69	36.93	35.72	77	3	FAN	REST1	28	50
	79	36.69	36.94	35.72	86	3	FAN	REST1	28	50
	80	36.69	36.94	35.75	79	3	FAN	REST1	28	50

min	number	Tre	Tes	Tsk-head	HR	ID	condition	period	Ta	RH
	81	36.69	36.94	35.75	83	3	FAN	REST1	28	50
	82	36.69	36.94	35.75	85	3	FAN	REST1	28	50
	83	36.69	36.95	35.73	78	3	FAN	REST1	28	50
	84	36.69	36.95	35.74	79	3	FAN	REST1	28	50
	85	36.69	36.94	35.76	84	3	FAN	REST1	28	50
	86	36.69	36.94	35.77	85	3	FAN	REST1	28	50
	87	36.70	36.94	35.77	84	3	FAN	REST1	28	50
	88	36.70	36.95	35.75	81	3	FAN	REST1	28	50
	89	36.70	36.95	35.75	81	3	FAN	REST1	28	50
	90	36.70	36.94	35.77	84	3	FAN	REST1	28	50
15	91	36.70	36.93	35.76	87	3	FAN	REST1	28	50
	92	36.71	36.93	35.76	81	3	FAN	REST1	28	50
	93	36.72	36.93	35.75	77	3	FAN	REST1	28	50
	94	36.72	36.94	35.75	94	3	FAN	REST1	28	50
	95	36.70	36.95	35.78	85	3	FAN	REST1	28	50
	96	36.69	36.95	35.77	90	3	FAN	REST1	28	50
	97	36.68	36.95	35.75	92	3	FAN	REST1	28	50
	98	36.67	36.95	35.77	92	3	FAN	REST1	28	50
	99	36.67	36.97	35.76	95	3	FAN	REST1	28	50
	100	36.67	36.97	35.75	77	3	FAN	REST1	28	50
	101	36.65	36.96	35.77	84	3	FAN	REST1	28	50
	102	36.65	36.96	35.74	89	3	FAN	REST1	28	50
	103	36.65	36.96	35.75	90	3	FAN	REST1	40	50
	104	36.63	36.96	35.77	93	3	FAN	REST1	40	50
	105	36.63	36.95	35.76	93	3	FAN	REST1	40	50
	106	36.63	36.91	35.79	93	3	FAN	REST1	40	50
	107	36.64	36.91	35.86	98	3	FAN	REST1	40	50
	108	36.64	36.92	35.93	88	3	FAN	REST1	40	50
	109	36.64	36.92	35.99	89	3	FAN	REST1	40	50
	110	36.63	36.87	36.03	92	3	FAN	REST1	40	50
	111	36.64	36.87	36.08	100	3	FAN	REST1	40	50
	112	36.65	36.95	36.16	87	3	FAN	REST1	40	50
	113	36.64	36.95	36.20	99	3	FAN	REST1	40	50
	114	36.62	36.93	36.24	99	3	FAN	REST1	40	50
	115	36.61	36.93	36.30	92	3	FAN	REST1	40	50
	116	36.63	36.94	36.36	94	3	FAN	REST1	40	50
	117	36.63	36.95	36.41	94	3	FAN	REST1	40	50
	118	36.63	36.95	36.45	91	3	FAN	REST1	40	50
	119	36.63	36.95	36.50	93	3	FAN	REST1	40	50
	120	36.62	36.94	36.54	99	3	FAN	REST1	40	50
20	121	36.63	36.94	36.58	91	3	FAN	REST1	40	50
	122	36.62	36.94	36.62	85	3	FAN	REST1	40	50
	123	36.62	36.95	36.67	87	3	FAN	REST1	40	50
	124	36.63	36.96	36.72	90	3	FAN	REST1	40	50
	125	36.62	36.95	36.75	97	3	FAN	REST1	40	50
	126	36.62	36.95	36.78	92	3	FAN	REST1	40	50

min	number	Tre	Tes	Tsk-head	HR	ID	condition	period	Ta	RH
	127	36.63	36.96	36.81	94	3	FAN	REST1	40	50
	128	36.65	36.96	36.84	95	3	FAN	REST1	40	50
	129	36.65	36.97	36.87	91	3	FAN	REST1	40	50
	130	36.64	36.97	36.88	88	3	FAN	REST1	40	50
	131	36.64	36.97	36.89	101	3	FAN	REST1	40	50
	132	36.64	36.95	36.91	95	3	FAN	REST1	40	50
	133	36.64	36.93	36.94	111	3	FAN	REST1	40	50
	134	36.64	36.94	36.95	101	3	FAN	REST1	40	50
	135	36.65	36.95	36.96	105	3	FAN	REST1	40	50
	136	36.66	36.95	36.98	99	3	FAN	REST1	40	50
	137	36.66	36.96	36.99	96	3	FAN	REST1	40	50
	138	36.66	36.98	37.00	96	3	FAN	REST1	40	50
	139	36.66	36.97	37.02	104	3	FAN	EXERCISE1	40	50
	140	36.67	36.98	37.04	106	3	FAN	EXERCISE1	40	50
	141	36.67	37.00	37.05	97	3	FAN	EXERCISE1	40	50
	142	36.66	36.97	37.06	103	3	FAN	EXERCISE1	40	50
	143	36.66	36.96	37.07	106	3	FAN	EXERCISE1	40	50
	144	36.66	36.99	37.09	111	3	FAN	EXERCISE1	40	50
	145	36.67	37.00	37.10	112	3	FAN	EXERCISE1	40	50
	146	36.66	37.00	37.11	110	3	FAN	EXERCISE1	40	50
	147	36.66	37.01	37.12	113	3	FAN	EXERCISE1	40	50
	148	36.66	37.00	37.13	113	3	FAN	EXERCISE1	40	50
	149	36.66	36.97	37.14	115	3	FAN	EXERCISE1	40	50
	150	36.67	36.93	37.16	114	3	FAN	EXERCISE1	40	50
25	151	36.67	36.95	37.16	116	3	FAN	EXERCISE1	40	50
	152	36.67	36.98	37.17	116	3	FAN	EXERCISE1	40	50
	153	36.67	36.99	37.18	115	3	FAN	EXERCISE1	40	50
	154	36.67	37.00	37.19	117	3	FAN	EXERCISE1	40	50
	155	36.67	37.01	37.19	116	3	FAN	EXERCISE1	40	50
	156	36.67	37.02	37.19	114	3	FAN	EXERCISE1	40	50
	157	36.67	37.03	37.20	116	3	FAN	EXERCISE1	40	50
	158	36.67	37.03	37.21	116	3	FAN	EXERCISE1	40	50
	159	36.67	37.03	37.22	121	3	FAN	EXERCISE1	40	50
	160	36.67	37.04	37.23	119	3	FAN	EXERCISE1	40	50
	161	36.67	37.04	37.23	108	3	FAN	EXERCISE1	40	50
	162	36.67	37.05	37.23	120	3	FAN	EXERCISE1	40	50
	163	36.67	37.06	37.24	119	3	FAN	EXERCISE1	40	50
	164	36.67	37.06	37.24	120	3	FAN	EXERCISE1	40	50
	165	36.68	37.07	37.25	122	3	FAN	EXERCISE1	40	50
	166	36.68	37.09	37.25	126	3	FAN	EXERCISE1	40	50
	167	36.68	37.09	37.26	123	3	FAN	EXERCISE1	40	50
	168	36.68	37.11	37.27	124	3	FAN	EXERCISE1	40	50
	169	36.67	37.13	37.27	123	3	FAN	EXERCISE1	40	50
	170	36.68	37.12	37.28	127	3	FAN	EXERCISE1	40	50
	171	36.69	37.12	37.28	126	3	FAN	EXERCISE1	40	50
	172	36.69	37.14	37.28	125	3	FAN	EXERCISE1	40	50

min	number	Tre	Tes	Tsk-head	HR	ID	condition	period	Ta	RH
	173	36.69	37.14	37.29	128	3	FAN	EXERCISE1	40	50
	174	36.69	37.16	37.29	130	3	FAN	EXERCISE1	40	50
	175	36.69	37.16	37.30	128	3	FAN	EXERCISE1	40	50
	176	36.69	37.16	37.30	130	3	FAN	EXERCISE1	40	50
	177	36.70	37.17	37.31	128	3	FAN	EXERCISE1	40	50
	178	36.70	37.17	37.31	133	3	FAN	EXERCISE1	40	50
	179	36.70	37.20	37.31	128	3	FAN	EXERCISE1	40	50
	180	36.70	37.22	37.32	128	3	FAN	EXERCISE1	40	50
30	181	36.71	37.23	37.33	138	3	FAN	EXERCISE1	40	50
	182	36.72	37.23	37.34	137	3	FAN	EXERCISE1	40	50
	183	36.72	37.22	37.34	139	3	FAN	EXERCISE1	40	50
	184	36.74	37.24	37.34	138	3	FAN	EXERCISE1	40	50
	185	36.74	37.27	37.34	133	3	FAN	EXERCISE1	40	50
	186	36.74	37.27	37.34	132	3	FAN	EXERCISE1	40	50
	187	36.75	37.25	37.34	133	3	FAN	EXERCISE1	40	50
	188	36.75	37.28	37.35	128	3	FAN	EXERCISE1	40	50
	189	36.76	37.29	37.36	129	3	FAN	EXERCISE1	40	50
	190	36.76	37.28	37.36	130	3	FAN	EXERCISE1	40	50
	191	36.76	37.29	37.36	129	3	FAN	EXERCISE1	40	50
	192	36.77	37.30	37.37	130	3	FAN	EXERCISE1	40	50
	193	36.77	37.31	37.37	131	3	FAN	EXERCISE1	40	50
	194	36.77	37.33	37.36	133	3	FAN	EXERCISE1	40	50
	195	36.78	37.35	37.37	133	3	FAN	EXERCISE1	40	50
	196	36.78	37.35	37.37	140	3	FAN	EXERCISE1	40	50
	197	36.78	37.36	37.37	141	3	FAN	EXERCISE1	40	50
	198	36.79	37.39	37.38	143	3	FAN	EXERCISE1	40	50
	199	36.79	37.40	37.39	137	3	FAN	EXERCISE1	40	50
	200	36.78	37.38	37.39	135	3	FAN	EXERCISE1	40	50
	201	36.79	37.39	37.40	140	3	FAN	EXERCISE1	40	50
	202	36.80	37.40	37.40	139	3	FAN	EXERCISE1	40	50
	203	36.80	37.40	37.40	134	3	FAN	EXERCISE1	40	50
	204	36.80	37.38	37.40	135	3	FAN	EXERCISE1	40	50
	205	36.80	37.40	37.40	134	3	FAN	EXERCISE1	40	50
	206	36.80	37.41	37.41	135	3	FAN	EXERCISE1	40	50
	207	36.80	37.41	37.42	135	3	FAN	EXERCISE1	40	50
	208	36.80	37.43	37.42	136	3	FAN	EXERCISE1	40	50
	209	36.81	37.46	37.43	136	3	FAN	EXERCISE1	40	50
	210	36.80	37.49	37.43	139	3	FAN	EXERCISE1	40	50
35	211	36.80	37.48	37.43	138	3	FAN	EXERCISE1	40	50
	212	36.81	37.47	37.43	134	3	FAN	EXERCISE1	40	50
	213	36.82	37.49	37.44	135	3	FAN	EXERCISE1	40	50
	214	36.82	37.50	37.44	133	3	FAN	EXERCISE1	40	50
	215	36.82	37.51	37.44	136	3	FAN	EXERCISE1	40	50
	216	36.83	37.52	37.45	137	3	FAN	EXERCISE1	40	50
	217	36.83	37.52	37.45	133	3	FAN	EXERCISE1	40	50
	218	36.84	37.55	37.45	134	3	FAN	EXERCISE1	40	50

min	number	Tre	Tes	Tsk-head	HR	ID	condition	period	Ta	RH
	219	36.85	37.56	37.44	136	3	FAN	EXERCISE1	40	50
	220	36.85	37.56	37.45	140	3	FAN	EXERCISE1	40	50
	221	36.85	37.55	37.46	135	3	FAN	EXERCISE1	40	50
	222	36.85	37.54	37.45	129	3	FAN	EXERCISE1	40	50
	223	36.86	37.57	37.46	130	3	FAN	EXERCISE1	40	50
	224	36.87	37.58	37.47	132	3	FAN	EXERCISE1	40	50
	225	36.87	37.58	37.47	128	3	FAN	EXERCISE1	40	50
	226	36.87	37.57	37.47	132	3	FAN	EXERCISE1	40	50
	227	36.87	37.56	37.47	134	3	FAN	EXERCISE1	40	50
	228	36.88	37.59	37.47	137	3	FAN	EXERCISE1	40	50
	229	36.88	37.59	37.47	136	3	FAN	EXERCISE1	40	50
	230	36.88	37.58	37.46	135	3	FAN	EXERCISE1	40	50
	231	36.87	37.59	37.47	131	3	FAN	EXERCISE1	40	50
	232	36.87	37.60	37.47	137	3	FAN	EXERCISE1	40	50
	233	36.87	37.59	37.48	137	3	FAN	EXERCISE1	40	50
	234	36.87	37.59	37.48	137	3	FAN	EXERCISE1	40	50
	235	36.87	37.58	37.48	140	3	FAN	EXERCISE1	40	50
	236	36.87	37.58	37.48	136	3	FAN	EXERCISE1	40	50
	237	36.88	37.59	37.47	140	3	FAN	EXERCISE1	40	50
	238	36.88	37.60	37.47	136	3	FAN	EXERCISE1	40	50
	239	36.90	37.61	37.47	134	3	FAN	EXERCISE1	40	50
	240	36.91	37.62	37.47	135	3	FAN	EXERCISE1	40	50
40	241	36.91	37.62	37.47	137	3	FAN	EXERCISE1	40	50
	242	36.90	37.63	37.46	135	3	FAN	EXERCISE1	40	50
	243	36.90	37.65	37.46	132	3	FAN	EXERCISE1	40	50
	244	36.91	37.64	37.47	136	3	FAN	EXERCISE1	40	50
	245	36.92	37.65	37.48	135	3	FAN	EXERCISE1	40	50
	246	36.92	37.66	37.48	136	3	FAN	EXERCISE1	40	50
	247	36.92	37.66	37.48	136	3	FAN	EXERCISE1	40	50
	248	36.92	37.66	37.49	134	3	FAN	EXERCISE1	40	50
	249	36.92	37.66	37.49	138	3	FAN	EXERCISE1	40	50
	250	36.92	37.66	37.49	138	3	FAN	EXERCISE1	40	50
	251	36.92	37.65	37.48	155	3	FAN	EXERCISE1	40	50
	252	36.92	37.65	37.48	139	3	FAN	EXERCISE1	40	50
	253	36.91	37.65	37.49	149	3	FAN	EXERCISE1	40	50
	254	36.91	37.66	37.49	137	3	FAN	EXERCISE1	40	50
	255	36.91	37.68	37.50	139	3	FAN	EXERCISE1	40	50
	256	36.92	37.68	37.50	137	3	FAN	EXERCISE1	40	50
	257	36.92	37.69	37.50	142	3	FAN	EXERCISE1	40	50
	258	36.93	37.69	37.50	141	3	FAN	EXERCISE1	40	50
	259	36.92	37.67	37.50	139	3	FAN	EXERCISE1	40	50
	260	36.93	37.68	37.50	142	3	FAN	EXERCISE1	40	50
	261	36.94	37.71	37.50	141	3	FAN	EXERCISE1	40	50
	262	36.94	37.70	37.51	140	3	FAN	EXERCISE1	40	50
	263	36.95	37.71	37.51	135	3	FAN	EXERCISE1	40	50
	264	36.96	37.72	37.51	138	3	FAN	EXERCISE1	40	50

min	number	Tre	Tes	Tsk-head	HR	ID	condition	period	Ta	RH
	265	36.96	37.71	37.51	140	3	FAN	EXERCISE1	40	50
	266	36.95	37.70	37.51	137	3	FAN	EXERCISE1	40	50
	267	36.95	37.72	37.52	142	3	FAN	EXERCISE1	40	50
	268	36.95	37.74	37.52	142	3	FAN	EXERCISE1	40	50
	269	36.96	37.73	37.52	138	3	FAN	EXERCISE1	40	50
	270	36.97	37.72	37.52	152	3	FAN	EXERCISE1	40	50
45	271	36.96	37.71	37.52	139	3	FAN	EXERCISE1	40	50
	272	36.97	37.72	37.52	138	3	FAN	EXERCISE1	40	50
	273	36.99	37.72	37.52	173	3	FAN	EXERCISE1	40	50
	274	37.00	37.71	37.52	156	3	FAN	EXERCISE1	40	50
	275	37.00	37.73	37.51	137	3	FAN	EXERCISE1	40	50
	276	37.01	37.75	37.52	138	3	FAN	EXERCISE1	40	50
	277	37.01	37.76	37.51	157	3	FAN	EXERCISE1	40	50
	278	37.01	37.77	37.51	138	3	FAN	EXERCISE1	40	50
	279	37.01	37.75	37.51	159	3	FAN	EXERCISE1	40	50
	280	37.02	37.75	37.52	140	3	FAN	EXERCISE1	40	50
	281	37.02	37.74	37.52	141	3	FAN	EXERCISE1	40	50
	282	37.01	37.74	37.51	140	3	FAN	EXERCISE1	40	50
	283	37.01	37.77	37.51	141	3	FAN	EXERCISE1	40	50
	284	37.02	37.78	37.52	142	3	FAN	EXERCISE1	40	50
	285	37.03	37.79	37.53	140	3	FAN	EXERCISE1	40	50
	286	37.04	37.81	37.53	143	3	FAN	EXERCISE1	40	50
	287	37.04	37.81	37.52	143	3	FAN	EXERCISE1	40	50
	288	37.04	37.80	37.52	142	3	FAN	EXERCISE1	40	50
	289	37.04	37.78	37.52	145	3	FAN	EXERCISE1	40	50
	290	37.04	37.77	37.52	179	3	FAN	EXERCISE1	40	50
	291	37.05	37.81	37.53	144	3	FAN	EXERCISE1	40	50
	292	37.05	37.83	37.53	166	3	FAN	EXERCISE1	40	50
	293	37.05	37.83	37.53	162	3	FAN	EXERCISE1	40	50
	294	37.05	37.82	37.54	173	3	FAN	EXERCISE1	40	50
	295	37.06	37.76	37.53	166	3	FAN	EXERCISE1	40	50
	296	37.07	37.76	37.53	146	3	FAN	EXERCISE1	40	50
	297	37.07	37.83	37.54	147	3	FAN	EXERCISE1	40	50
	298	37.07	37.83	37.54	148	3	FAN	EXERCISE1	40	50
	299	37.06	37.81	37.53	145	3	FAN	EXERCISE1	40	50
	300	37.07	37.80	37.54	177	3	FAN	EXERCISE1	40	50
50	301	37.07	37.78	37.54	167	3	FAN	EXERCISE1	40	50
	302	37.07	37.82	37.55	145	3	FAN	EXERCISE1	40	50
	303	37.08	37.82	37.55	141	3	FAN	EXERCISE1	40	50
	304	37.08	37.81	37.55	145	3	FAN	EXERCISE1	40	50
	305	37.09	37.84	37.56	177	3	FAN	EXERCISE1	40	50
	306	37.09	37.85	37.56	130	3	FAN	EXERCISE1	40	50
	307	37.10	37.85	37.55	145	3	FAN	EXERCISE1	40	50
	308	37.12	37.86	37.56	166	3	FAN	EXERCISE1	40	50
	309	37.12	37.86	37.57	162	3	FAN	EXERCISE1	40	50
	310	37.11	37.86	37.56	142	3	FAN	EXERCISE1	40	50

min	number	Tre	Tes	Tsk-head	HR	ID	condition	period	Ta	RH
	311	37.10	37.86	37.55	142	3	FAN	EXERCISE1	40	50
	312	37.11	37.85	37.56	143	3	FAN	EXERCISE1	40	50
	313	37.12	37.87	37.57	145	3	FAN	EXERCISE1	40	50
	314	37.12	37.88	37.57	145	3	FAN	EXERCISE1	40	50
	315	37.13	37.88	37.56	147	3	FAN	EXERCISE1	40	50
	316	37.13	37.87	37.56	144	3	FAN	EXERCISE1	40	50
	317	37.14	37.89	37.56	144	3	FAN	EXERCISE1	40	50
	318	37.14	37.90	37.57	147	3	FAN	EXERCISE1	40	50
	319	37.14	37.91	37.57	146	3	FAN	EXERCISE1	40	50
	320	37.14	37.90	37.55	229	3	FAN	REST2	28	50
	321	37.13	37.90	37.55	209	3	FAN	REST2	28	50
	322	37.13	37.93	37.54	162	3	FAN	REST2	28	50
	323	37.14	37.93	37.48	137	3	FAN	REST2	28	50
	324	37.16	37.95	37.41	129	3	FAN	REST2	28	50
	325	37.17	37.99	36.99	142	3	FAN	REST2	28	50
	326	37.17	37.95	36.24	136	3	FAN	REST2	28	50
	327	37.19	37.93	35.75	122	3	FAN	REST2	28	50
	328	37.20	37.95	35.61	121	3	FAN	REST2	28	50
	329	37.21	37.97	35.53	119	3	FAN	REST2	28	50
	330	37.21	37.98	35.35	115	3	FAN	REST2	28	50
55	331	37.21	37.96	35.24	113	3	FAN	REST2	28	50
	332	37.22	37.96	35.29	107	3	FAN	REST2	28	50
	333	37.23	37.95	35.53	119	3	FAN	REST2	28	50
	334	37.23	37.91	35.85	114	3	FAN	REST2	28	50
	335	37.24	37.87	35.99	112	3	FAN	REST2	28	50
	336	37.25	37.86	36.03	113	3	FAN	REST2	28	50
	337	37.26	37.87	36.06	114	3	FAN	REST2	28	50
	338	37.26	37.79	36.13	113	3	FAN	REST2	28	50
	339	37.26	32.46	36.19	116	3	FAN	REST2	28	50
	340	37.26	27.72	36.18	109	3	FAN	REST2	28	50
	341	37.27	29.01	36.18	106	3	FAN	REST2	28	50
	342	37.29	29.91	36.20	106	3	FAN	REST2	28	50
	343	37.29	29.93	36.21	112	3	FAN	REST2	28	50
	344	37.29	29.96	36.23	106	3	FAN	REST2	28	50
	345	37.30	30.00	36.22	112	3	FAN	REST2	28	50
	346	37.29	30.14	36.20	110	3	FAN	REST2	28	50
	347	37.29	29.04	36.19	115	3	FAN	REST2	28	50
	348	37.29	28.66	36.18	113	3	FAN	REST2	28	50
	349	37.30	28.96	36.19	113	3	FAN	REST2	28	50
	350	37.31	27.59	36.22	115	3	FAN	REST2	28	50
	351	37.32	27.79	36.22	112	3	FAN	REST2	28	50
	352	37.33	28.25	36.22	109	3	FAN	REST2	28	50
	353	37.34	28.13	36.22	103	3	FAN	REST2	28	50
	354	37.35	28.59	36.18	106	3	FAN	REST2	28	50
	355	37.35	28.90	36.15	106	3	FAN	REST2	28	50
	356	37.35	28.96	36.12	106	3	FAN	REST2	28	50

min	number	Tre	Tes	Tsk-head	HR	ID	condition	period	Ta	RH
	357	37.34	28.75	36.06	108	3	FAN	REST2	28	50
	358	37.33	28.83	36.02	105	3	FAN	REST2	28	50
	359	37.32	29.05	35.99	111	3	FAN	REST2	28	50
	360	37.30	29.03	35.96	103	3	FAN	REST2	28	50
60	361	37.29	28.97	35.90	98	3	FAN	REST2	28	50
	362	37.30	29.16	35.87	101	3	FAN	REST2	28	50
	363	37.30	29.43	35.86	102	3	FAN	REST2	28	50
	364	37.29	29.55	35.81	103	3	FAN	REST2	28	50
	365	37.29	29.67	35.78	101	3	FAN	REST2	28	50
	366	37.29	29.74	35.79	103	3	FAN	REST2	28	50
	367	37.29	29.62	35.82	95	3	FAN	REST2	28	50
	368	37.30	29.76	35.82	99	3	FAN	REST2	28	50
	369	37.29	29.99	35.81	102	3	FAN	REST2	28	50
	370	37.27	30.13	35.82	104	3	FAN	REST2	28	50
	371	37.27	29.40	35.83	107	3	FAN	REST2	28	50
	372	37.27	28.87	35.82	104	3	FAN	REST2	28	50
	373	37.27	29.57	35.80	100	3	FAN	REST2	28	50
	374	37.27	29.81	35.80	95	3	FAN	REST2	28	50
	375	37.27	29.89	35.81	95	3	FAN	REST2	28	50
	376	37.26	30.35	35.81	96	3	FAN	REST2	28	50
	377	37.26	30.66	35.82	90	3	FAN	REST2	28	50
	378	37.26	30.74	35.83	97	3	FAN	REST2	28	50
	379	37.26	30.76	35.83	97	3	FAN	REST2	28	50
	380	37.26	30.87	35.84	91	3	FAN	REST2	28	50
	381	37.26	30.98	35.85	87	3	FAN	REST2	28	50
	382	37.26	31.03	35.86	87	3	FAN	REST2	28	50
	383	37.25	31.08	35.85	87	3	FAN	REST2	28	50
	384	37.25	31.11	35.85	85	3	FAN	REST2	28	50
	385	37.25	31.19	35.86	85	3	FAN	REST2	28	50
	386	37.25	31.26	35.90	88	3	FAN	REST2	28	50
	387	37.26	31.28	35.93	89	3	FAN	REST2	28	50
	388	37.26	31.36	35.95	89	3	FAN	REST2	28	50
	389	37.25	31.42	35.95	91	3	FAN	REST2	28	50
	390	37.25	31.56	35.96	88	3	FAN	REST2	28	50
65	391	37.25	31.72	35.99	85	3	FAN	REST2	28	50
	392	37.26	31.94	35.97	92	3	FAN	REST2	28	50
	393	37.27	32.13	35.95	90	3	FAN	REST2	28	50
	394	37.27	32.15	35.94	80	3	FAN	REST2	28	50
	395	37.27	32.26	35.92	83	3	FAN	REST2	28	50
	396	37.26	32.45	35.90	89	3	FAN	REST2	28	50
	397	37.25	32.76	35.93	89	3	FAN	REST2	28	50
	398	37.25	33.11	35.94	99	3	FAN	REST2	28	50
	399	37.26	33.17	35.94	92	3	FAN	REST2	28	50
	400	37.25	33.10	35.94	83	3	FAN	REST2	28	50
	401	37.26	33.15	35.95	85	3	FAN	REST2	28	50
	402	37.26	33.13	35.97	93	3	FAN	REST2	28	50

min	number	Tre	Tes	Tsk-head	HR	ID	condition	period	Ta	RH
	403	37.26	33.11	35.95	87	3	FAN	REST2	28	50
	404	37.27	33.19	35.96	87	3	FAN	REST2	28	50
	405	37.27	33.25	35.97	85	3	FAN	REST2	28	50
	406	37.27	33.36	35.96	85	3	FAN	REST2	28	50
	407	37.27	33.53	35.93	87	3	FAN	REST2	28	50
	408	37.27	33.64	35.88	92	3	FAN	REST2	28	50
	409	37.28	33.68	35.85	89	3	FAN	REST2	28	50
	410	37.27	33.86	35.84	90	3	FAN	REST2	28	50
	411	37.27	33.86	35.84	95	3	FAN	REST2	28	50
	412	37.27	33.80	35.86	87	3	FAN	REST2	28	50
	413	37.28	33.92	35.88	89	3	FAN	REST2	28	50
	414	37.29	33.98	35.92	92	3	FAN	REST2	28	50
	415	37.29	34.04	35.92	96	3	FAN	REST2	28	50
	416	37.30	33.98	35.92	92	3	FAN	REST2	28	50
	417	37.31	34.09	35.94	119	3	FAN	REST2	28	50
	418	37.33	34.29	35.97	99	3	FAN	REST2	28	50
	419	37.34	34.31	35.99	101	3	FAN	REST2	28	50
	420	37.35	34.38	36.01	140	3	FAN	REST2	28	50
70	421	37.35	34.44	36.04	118	3	FAN	REST2	28	50
	422	37.36	34.51	36.05	114	3	FAN	REST2	28	50
	423	37.36	34.58	36.05	102	3	FAN	REST2	28	50
	424	37.36	34.64	36.04	94	3	FAN	REST2	28	50
	425	37.35	34.67	36.01	93	3	FAN	REST2	28	50
	426	37.35	34.67	36.01	100	3	FAN	REST2	28	50
	427	37.35	34.74	35.98	99	3	FAN	REST2	28	50
	428	37.36	34.83	35.86	95	3	FAN	REST2	28	50
	429	37.37	34.89	35.79	96	3	FAN	REST2	28	50
	430	37.36	34.94	35.80	99	3	FAN	REST2	28	50
	431	37.35	34.98	35.77	100	3	FAN	REST2	28	50
	432	37.35	35.04	35.76	96	3	FAN	REST2	28	50
	433	37.36	35.11	35.80	91	3	FAN	REST2	28	50
	434	37.36	35.04	35.79	95	3	FAN	REST2	28	50
	435	37.37	35.04	35.75	102	3	FAN	REST2	28	50
	436	37.37	35.19	35.73	91	3	FAN	REST2	28	50
	437	37.38	35.25	35.72	93	3	FAN	REST2	28	50
	438	37.38	35.26	35.72	92	3	FAN	REST2	28	50
	439	37.39	35.30	35.71	86	3	FAN	REST2	40	50
	440	37.40	35.30	35.69	102	3	FAN	REST2	40	50
	441	37.38	35.34	35.80	107	3	FAN	REST2	40	50
	442	37.37	35.37	36.00	103	3	FAN	REST2	40	50
	443	37.38	35.32	36.16	104	3	FAN	REST2	40	50
	444	37.39	35.35	36.30	102	3	FAN	REST2	40	50
	445	37.39	35.38	36.42	105	3	FAN	REST2	40	50
	446	37.39	35.42	36.50	104	3	FAN	REST2	40	50
	447	37.39	35.45	36.55	107	3	FAN	REST2	40	50
	448	37.40	35.48	36.59	93	3	FAN	REST2	40	50

min	number	Tre	Tes	Tsk-head	HR	ID	condition	period	Ta	RH
	449	37.40	35.52	36.62	105	3	FAN	REST2	40	50
	450	37.40	35.53	36.65	101	3	FAN	REST2	40	50
75	451	37.39	35.56	36.68	97	3	FAN	REST2	40	50
	452	37.40	35.60	36.71	103	3	FAN	REST2	40	50
	453	37.40	35.64	36.74	106	3	FAN	REST2	40	50
	454	37.40	35.68	36.75	102	3	FAN	REST2	40	50
	455	37.39	35.67	36.78	103	3	FAN	REST2	40	50
	456	37.38	35.66	36.81	103	3	FAN	REST2	40	50
	457	37.38	35.66	36.83	98	3	FAN	REST2	40	50
	458	37.38	35.74	36.88	109	3	FAN	REST2	40	50
	459	37.38	35.82	36.91	103	3	FAN	REST2	40	50
	460	37.38	35.83	36.94	108	3	FAN	REST2	40	50
	461	37.39	35.85	36.95	109	3	FAN	REST2	40	50
	462	37.39	35.87	36.97	110	3	FAN	REST2	40	50
	463	37.39	35.90	36.99	110	3	FAN	EXERCISE2	40	50
	464	37.39	35.94	37.00	103	3	FAN	EXERCISE2	40	50
	465	37.39	35.96	37.00	107	3	FAN	EXERCISE2	40	50
	466	37.38	35.98	37.01	111	3	FAN	EXERCISE2	40	50
	467	37.37	36.00	37.03	114	3	FAN	EXERCISE2	40	50
	468	37.38	36.01	37.05	112	3	FAN	EXERCISE2	40	50
	469	37.38	36.04	37.06	113	3	FAN	EXERCISE2	40	50
	470	37.37	36.08	37.07	119	3	FAN	EXERCISE2	40	50
	471	37.37	36.10	37.08	120	3	FAN	EXERCISE2	40	50
	472	37.36	36.10	37.09	122	3	FAN	EXERCISE2	40	50
	473	37.37	36.12	37.09	123	3	FAN	EXERCISE2	40	50
	474	37.38	36.15	37.10	122	3	FAN	EXERCISE2	40	50
	475	37.38	36.18	37.12	123	3	FAN	EXERCISE2	40	50
	476	37.39	36.21	37.14	122	3	FAN	EXERCISE2	40	50
	477	37.39	36.24	37.16	121	3	FAN	EXERCISE2	40	50
	478	37.39	36.24	37.17	122	3	FAN	EXERCISE2	40	50
	479	37.39	36.26	37.19	128	3	FAN	EXERCISE2	40	50
	480	37.40	36.29	37.21	126	3	FAN	EXERCISE2	40	50
80	481	37.39	36.31	37.22	127	3	FAN	EXERCISE2	40	50
	482	37.39	36.32	37.24	128	3	FAN	EXERCISE2	40	50
	483	37.39	36.34	37.25	132	3	FAN	EXERCISE2	40	50
	484	37.39	36.36	37.26	128	3	FAN	EXERCISE2	40	50
	485	37.40	36.39	37.27	131	3	FAN	EXERCISE2	40	50
	486	37.40	36.40	37.29	128	3	FAN	EXERCISE2	40	50
	487	37.40	36.42	37.31	130	3	FAN	EXERCISE2	40	50
	488	37.40	36.45	37.32	128	3	FAN	EXERCISE2	40	50
	489	37.40	36.47	37.33	128	3	FAN	EXERCISE2	40	50
	490	37.41	36.49	37.34	132	3	FAN	EXERCISE2	40	50
	491	37.41	36.51	37.35	129	3	FAN	EXERCISE2	40	50
	492	37.41	36.52	37.36	130	3	FAN	EXERCISE2	40	50
	493	37.41	36.54	37.36	131	3	FAN	EXERCISE2	40	50
	494	37.41	36.56	37.37	130	3	FAN	EXERCISE2	40	50

min	number	Tre	Tes	Tsk-head	HR	ID	condition	period	Ta	RH
	495	37.42	36.59	37.38	128	3	FAN	EXERCISE2	40	50
	496	37.43	36.61	37.39	131	3	FAN	EXERCISE2	40	50
	497	37.45	36.62	37.40	131	3	FAN	EXERCISE2	40	50
	498	37.45	36.63	37.40	134	3	FAN	EXERCISE2	40	50
	499	37.45	36.66	37.40	134	3	FAN	EXERCISE2	40	50
	500	37.45	36.68	37.41	137	3	FAN	EXERCISE2	40	50
	501	37.45	36.70	37.42	137	3	FAN	EXERCISE2	40	50
	502	37.44	36.71	37.42	137	3	FAN	EXERCISE2	40	50
	503	37.44	36.73	37.41	138	3	FAN	EXERCISE2	40	50
	504	37.43	36.74	37.41	123	3	FAN	EXERCISE2	40	50
	505	37.42	36.75	37.43	134	3	FAN	EXERCISE2	40	50
	506	37.43	36.76	37.43	137	3	FAN	EXERCISE2	40	50
	507	37.43	36.78	37.42	136	3	FAN	EXERCISE2	40	50
	508	37.44	36.80	37.42	136	3	FAN	EXERCISE2	40	50
	509	37.44	36.81	37.42	132	3	FAN	EXERCISE2	40	50
	510	37.45	36.82	37.42	131	3	FAN	EXERCISE2	40	50
85	511	37.45	36.83	37.42	134	3	FAN	EXERCISE2	40	50
	512	37.44	36.83	37.42	133	3	FAN	EXERCISE2	40	50
	513	37.44	36.85	37.41	133	3	FAN	EXERCISE2	40	50
	514	37.44	36.87	37.42	134	3	FAN	EXERCISE2	40	50
	515	37.44	36.88	37.43	132	3	FAN	EXERCISE2	40	50
	516	37.44	36.90	37.44	131	3	FAN	EXERCISE2	40	50
	517	37.44	36.90	37.43	133	3	FAN	EXERCISE2	40	50
	518	37.43	36.91	37.43	134	3	FAN	EXERCISE2	40	50
	519	37.43	36.93	37.43	136	3	FAN	EXERCISE2	40	50
	520	37.43	36.93	37.43	134	3	FAN	EXERCISE2	40	50
	521	37.43	36.95	37.42	157	3	FAN	EXERCISE2	40	50
	522	37.43	36.97	37.41	137	3	FAN	EXERCISE2	40	50
	523	37.43	36.98	37.42	137	3	FAN	EXERCISE2	40	50
	524	37.43	36.98	37.43	137	3	FAN	EXERCISE2	40	50
	525	37.44	37.00	37.44	134	3	FAN	EXERCISE2	40	50
	526	37.44	37.02	37.45	132	3	FAN	EXERCISE2	40	50
	527	37.44	37.03	37.44	134	3	FAN	EXERCISE2	40	50
	528	37.44	37.04	37.44	135	3	FAN	EXERCISE2	40	50
	529	37.44	37.05	37.45	158	3	FAN	EXERCISE2	40	50
	530	37.44	37.06	37.45	143	3	FAN	EXERCISE2	40	50
	531	37.44	37.08	37.44	141	3	FAN	EXERCISE2	40	50
	532	37.45	37.09	37.44	142	3	FAN	EXERCISE2	40	50
	533	37.45	37.10	37.44	143	3	FAN	EXERCISE2	40	50
	534	37.45	37.10	37.45	142	3	FAN	EXERCISE2	40	50
	535	37.45	37.10	37.44	138	3	FAN	EXERCISE2	40	50
	536	37.46	37.11	37.44	139	3	FAN	EXERCISE2	40	50
	537	37.46	37.14	37.46	135	3	FAN	EXERCISE2	40	50
	538	37.47	37.15	37.45	140	3	FAN	EXERCISE2	40	50
	539	37.47	37.16	37.44	140	3	FAN	EXERCISE2	40	50
	540	37.48	37.17	37.45	136	3	FAN	EXERCISE2	40	50

min	number	Tre	Tes	Tsk-head	HR	ID	condition	period	Ta	RH
90	541	37.48	37.18	37.47	139	3	FAN	EXERCISE2	40	50
	542	37.49	37.19	37.48	139	3	FAN	EXERCISE2	40	50
	543	37.49	37.19	37.48	140	3	FAN	EXERCISE2	40	50
	544	37.49	37.22	37.49	137	3	FAN	EXERCISE2	40	50
	545	37.48	37.22	37.48	139	3	FAN	EXERCISE2	40	50
	546	37.48	37.22	37.47	140	3	FAN	EXERCISE2	40	50
	547	37.48	37.22	37.48	139	3	FAN	EXERCISE2	40	50
	548	37.48	37.23	37.48	140	3	FAN	EXERCISE2	40	50
	549	37.49	37.25	37.48	141	3	FAN	EXERCISE2	40	50
	550	37.49	37.26	37.48	140	3	FAN	EXERCISE2	40	50
	551	37.50	37.25	37.49	140	3	FAN	EXERCISE2	40	50
	552	37.50	37.25	37.50	140	3	FAN	EXERCISE2	40	50
	553	37.50	37.25	37.50	143	3	FAN	EXERCISE2	40	50
	554	37.50	37.26	37.49	143	3	FAN	EXERCISE2	40	50
	555	37.51	37.27	37.53	143	3	FAN	EXERCISE2	40	50
	556	37.50	37.27	37.53	143	3	FAN	EXERCISE2	40	50
	557	37.50	37.28	37.49	143	3	FAN	EXERCISE2	40	50
	558	37.50	37.31	37.48	143	3	FAN	EXERCISE2	40	50
	559	37.50	37.34	37.48	140	3	FAN	EXERCISE2	40	50
	560	37.49	37.35	37.49	141	3	FAN	EXERCISE2	40	50
	561	37.49	37.36	37.50	141	3	FAN	EXERCISE2	40	50
	562	37.50	37.38	37.50	141	3	FAN	EXERCISE2	40	50
	563	37.51	37.38	37.51	124	3	FAN	EXERCISE2	40	50
	564	37.52	37.37	37.52	138	3	FAN	EXERCISE2	40	50
	565	37.52	37.36	37.51	143	3	FAN	EXERCISE2	40	50
	566	37.52	37.37	37.50	142	3	FAN	EXERCISE2	40	50
	567	37.52	37.38	37.51	144	3	FAN	EXERCISE2	40	50
	568	37.52	37.37	37.52	139	3	FAN	EXERCISE2	40	50
	569	37.53	37.36	37.51	139	3	FAN	EXERCISE2	40	50
	570	37.53	37.38	37.51	138	3	FAN	EXERCISE2	40	50
95	571	37.54	37.39	37.51	140	3	FAN	EXERCISE2	40	50
	572	37.53	37.38	37.51	140	3	FAN	EXERCISE2	40	50
	573	37.52	37.39	37.52	140	3	FAN	EXERCISE2	40	50
	574	37.51	37.41	37.51	140	3	FAN	EXERCISE2	40	50
	575	37.51	37.41	37.50	143	3	FAN	EXERCISE2	40	50
	576	37.52	37.39	37.50	144	3	FAN	EXERCISE2	40	50
	577	37.52	37.41	37.49	143	3	FAN	EXERCISE2	40	50
	578	37.53	37.43	37.48	142	3	FAN	EXERCISE2	40	50
	579	37.53	37.44	37.49	143	3	FAN	EXERCISE2	40	50
	580	37.53	37.46	37.49	146	3	FAN	EXERCISE2	40	50
	581	37.54	37.46	37.50	143	3	FAN	EXERCISE2	40	50
	582	37.54	37.48	37.51	140	3	FAN	EXERCISE2	40	50
	583	37.54	37.48	37.50	143	3	FAN	EXERCISE2	40	50
	584	37.54	37.48	37.49	147	3	FAN	EXERCISE2	40	50
	585	37.54	37.50	37.48	144	3	FAN	EXERCISE2	40	50
	586	37.54	37.51	37.49	147	3	FAN	EXERCISE2	40	50

min	number	Tre	Tes	Tsk-head	HR	ID	condition	period	Ta	RH
	587	37.54	37.52	37.49	139	3	FAN	EXERCISE2	40	50
	588	37.53	37.52	37.49	143	3	FAN	EXERCISE2	40	50
	589	37.53	37.51	37.49	143	3	FAN	EXERCISE2	40	50
	590	37.54	37.51	37.50	139	3	FAN	EXERCISE2	40	50
	591	37.55	37.52	37.50	137	3	FAN	EXERCISE2	40	50
	592	37.55	37.53	37.48	140	3	FAN	EXERCISE2	40	50
	593	37.55	37.54	37.47	139	3	FAN	EXERCISE2	40	50
	594	37.55	37.57	37.46	139	3	FAN	EXERCISE2	40	50
	595	37.55	37.58	37.47	142	3	FAN	EXERCISE2	40	50
	596	37.55	37.56	37.48	145	3	FAN	EXERCISE2	40	50
	597	37.55	37.56	37.46	149	3	FAN	EXERCISE2	40	50
	598	37.56	37.57	37.47	146	3	FAN	EXERCISE2	40	50
	599	37.57	37.58	37.48	147	3	FAN	EXERCISE2	40	50
	600	37.57	37.61	37.48	146	3	FAN	EXERCISE2	40	50
100	601	37.58	37.63	37.49	143	3	FAN	EXERCISE2	40	50
	602	37.58	37.62	37.49	145	3	FAN	EXERCISE2	40	50
	603	37.57	37.60	37.48	146	3	FAN	EXERCISE2	40	50
	604	37.56	37.61	37.49	144	3	FAN	EXERCISE2	40	50
	605	37.57	37.61	37.49	143	3	FAN	EXERCISE2	40	50
	606	37.58	37.61	37.48	143	3	FAN	EXERCISE2	40	50
	607	37.59	37.62	37.49	140	3	FAN	EXERCISE2	40	50
	608	37.59	37.62	37.51	142	3	FAN	EXERCISE2	40	50
	609	37.59	37.62	37.50	141	3	FAN	EXERCISE2	40	50
	610	37.59	37.63	37.49	141	3	FAN	EXERCISE2	40	50
	611	37.58	37.64	37.49	143	3	FAN	EXERCISE2	40	50
	612	37.60	37.65	37.49	141	3	FAN	EXERCISE2	40	50
	613	37.61	37.67	37.50	146	3	FAN	EXERCISE2	40	50
	614	37.61	37.66	37.50	149	3	FAN	EXERCISE2	40	50
	615	37.61	37.65	37.51	146	3	FAN	EXERCISE2	40	50
	616	37.62	37.64	37.51	143	3	FAN	EXERCISE2	40	50
	617	37.62	37.65	37.51	145	3	FAN	EXERCISE2	40	50
	618	37.61	37.65	37.51	146	3	FAN	EXERCISE2	40	50
	619	37.61	37.68	37.53	148	3	FAN	EXERCISE2	40	50
	620	37.62	37.68	37.54	146	3	FAN	EXERCISE2	40	50
	621	37.62	37.67	37.54	146	3	FAN	EXERCISE2	40	50
	622	37.62	37.69	37.53	149	3	FAN	EXERCISE2	40	50
	623	37.63	37.72	37.54	151	3	FAN	EXERCISE2	40	50
	624	37.64	37.72	37.55	151	3	FAN	EXERCISE2	40	50
	625	37.64	37.72	37.55	153	3	FAN	EXERCISE2	40	50
	626	37.64	37.73	37.56	155	3	FAN	EXERCISE2	40	50
	627	37.64	37.72	37.55	153	3	FAN	EXERCISE2	40	50
	628	37.65	37.72	37.55	154	3	FAN	EXERCISE2	40	50
	629	37.66	37.74	37.55	153	3	FAN	EXERCISE2	40	50
	630	37.66	37.76	37.56	152	3	FAN	EXERCISE2	40	50
105	631	37.66	37.77	37.55	153	3	FAN	EXERCISE2	40	50
	632	37.67	37.77	37.54	153	3	FAN	EXERCISE2	40	50

min	number	Tre	Tes	Tsk-head	HR	ID	condition	period	Ta	RH
	633	37.68	37.77	37.54	153	3	FAN	EXERCISE2	40	50
	634	37.68	37.76	37.56	153	3	FAN	EXERCISE2	40	50
	635	37.67	37.77	37.57	152	3	FAN	EXERCISE2	40	50
	636	37.67	37.79	37.57	153	3	FAN	EXERCISE2	40	50
	637	37.67	37.79	37.57	150	3	FAN	EXERCISE2	40	50
	638	37.67	37.79	37.57	151	3	FAN	EXERCISE2	40	50
	639	37.68	37.79	37.57	147	3	FAN	EXERCISE2	40	50
	640	37.68	37.78	37.56	146	3	FAN	EXERCISE2	40	50
	641	37.68	37.79	37.56	144	3	FAN	EXERCISE2	40	50
	642	37.69	37.80	37.56	148	3	FAN	EXERCISE2	40	50
	643	37.68	37.82	37.56	147	3	FAN	EXERCISE2	40	50
	644	37.68	37.82	37.56	150	3	FAN	REST3	28	50
	645	37.68	37.82	37.56	150	3	FAN	REST3	28	50
	646	37.68	37.85	37.56	143	3	FAN	REST3	28	50
	647	37.67	37.85	37.57	145	3	FAN	REST3	28	50
	648	37.63	37.82	37.47	141	3	FAN	REST3	28	50
	649	37.63	37.86	37.34	139	3	FAN	REST3	28	50
	650	37.65	37.89	37.24	139	3	FAN	REST3	28	50
	651	37.64	37.88	37.14	132	3	FAN	REST3	28	50
	652	37.64	37.88	37.05	124	3	FAN	REST3	28	50
	653	37.65	37.90	36.97	126	3	FAN	REST3	28	50
	654	37.65	37.91	36.89	134	3	FAN	REST3	28	50
	655	37.65	37.90	36.82	133	3	FAN	REST3	28	50
	656	37.65	37.90	36.75	109	3	FAN	REST3	28	50
	657	37.65	37.90	36.68	112	3	FAN	REST3	28	50
	658	37.64	37.89	36.61	110	3	FAN	REST3	28	50
	659	37.62	37.89	36.54	112	3	FAN	REST3	28	50
	660	37.62	37.91	36.48	111	3	FAN	REST3	28	50
110	661	37.63	37.91	36.44	113	3	FAN	REST3	28	50
	662	37.65	37.90	36.42	107	3	FAN	REST3	28	50
	663	37.65	37.90	36.43	107	3	FAN	REST3	28	50
	664	37.65	37.90	36.41	106	3	FAN	REST3	28	50
	665	37.65	37.89	36.33	107	3	FAN	REST3	28	50
	666	37.65	37.90	36.25	104	3	FAN	REST3	28	50
	667	37.66	37.90	36.21	101	3	FAN	REST3	28	50
	668	37.65	37.88	36.17	95	3	FAN	REST3	28	50
	669	37.64	37.84	36.15	105	3	FAN	REST3	28	50
	670	37.63	37.81	36.12	102	3	FAN	REST3	28	50
	671	37.62	37.79	36.08	103	3	FAN	REST3	28	50
	672	37.63	37.78	36.09	112	3	FAN	REST3	28	50
	673	37.63	37.77	36.13	106	3	FAN	REST3	28	50
	674	37.61	37.75	36.18	107	3	FAN	REST3	28	50
	675	37.60	37.74	36.20	103	3	FAN	REST3	28	50
	676	37.59	37.75	36.19	103	3	FAN	REST3	28	50
	677	37.60	37.75	36.21	102	3	FAN	REST3	28	50
	678	37.60	37.74	36.24	98	3	FAN	REST3	28	50

min	number	Tre	Tes	Tsk-head	HR	ID	condition	period	Ta	RH
	679	37.60	37.74	36.25	99	3	FAN	REST3	28	50
	680	37.59	37.74	36.25	102	3	FAN	REST3	28	50
	681	37.59	37.73	36.24	101	3	FAN	REST3	28	50
	682	37.59	37.72	36.21	107	3	FAN	REST3	28	50
	683	37.59	37.72	36.21	107	3	FAN	REST3	28	50
	684	37.59	37.71	36.17	112	3	FAN	REST3	28	50
	685	37.59	37.72	36.08	108	3	FAN	REST3	28	50
	686	37.60	37.72	36.06	106	3	FAN	REST3	28	50
	687	37.63	37.72	36.04	99	3	FAN	REST3	28	50
	688	37.64	37.72	35.95	104	3	FAN	REST3	28	50
	689	37.62	37.71	35.90	101	3	FAN	REST3	28	50
	690	37.61	37.72	35.92	104	3	FAN	REST3	28	50
115	691	37.61	37.72	35.94	101	3	FAN	REST3	28	50
	692	37.60	37.72	35.93	102	3	FAN	REST3	28	50
	693	37.61	37.71	35.92	106	3	FAN	REST3	28	50
	694	37.61	37.72	35.89	102	3	FAN	REST3	28	50
	695	37.61	37.72	35.89	102	3	FAN	REST3	28	50
	696	37.61	37.70	35.92	101	3	FAN	REST3	28	50
	697	37.60	37.68	35.92	101	3	FAN	REST3	28	50
	698	37.60	37.66	35.95	102	3	FAN	REST3	28	50
	699	37.59	37.65	35.99	107	3	FAN	REST3	28	50
	700	37.58	37.66	36.00	101	3	FAN	REST3	28	50
	701	37.58	37.66	36.00	78	3	FAN	REST3	28	50
	702	37.57	37.65	36.03	98	3	FAN	REST3	28	50
	703	37.56	37.65	36.03	102	3	FAN	REST3	28	50
	704	37.56	37.65	36.01	96	3	FAN	REST3	28	50
	705	37.57	37.72	35.99	105	3	FAN	REST3	28	50
	706	37.57	37.76	36.00	93	3	FAN	REST3	28	50
	707	37.57	37.72	35.98	105	3	FAN	REST3	28	50
	708	37.56	37.68	35.94	108	3	FAN	REST3	28	50
	709	37.56	37.67	35.96	106	3	FAN	REST3	28	50
0	1	36.51	36.44	35.04	63	4	FAN	REST1	28	50
	2	36.50	36.42	35.03	64	4	FAN	REST1	28	50
	3	36.50	36.39	35.01	86	4	FAN	REST1	28	50
	4	36.50	36.37	35.01	74	4	FAN	REST1	28	50
	5	36.50	36.40	35.02	71	4	FAN	REST1	28	50
	6	36.50	36.39	35.02	68	4	FAN	REST1	28	50
	7	36.50	36.38	35.03	73	4	FAN	REST1	28	50
	8	36.49	36.40	35.05	78	4	FAN	REST1	28	50
	9	36.49	36.40	35.07	81	4	FAN	REST1	28	50
	10	36.49	36.42	35.09	68	4	FAN	REST1	28	50
	11	36.49	36.42	35.10	70	4	FAN	REST1	28	50
	12	36.50	36.43	35.11	68	4	FAN	REST1	28	50
	13	36.50	36.43	35.12	84	4	FAN	REST1	28	50
	14	36.50	36.41	35.13	76	4	FAN	REST1	28	50
	15	36.48	36.42	35.15	69	4	FAN	REST1	28	50

min	number	Tre	Tes	Tsk-head	HR	ID	condition	period	Ta	RH
	16	36.48	36.46	35.14	70	4	FAN	REST1	28	50
	17	36.48	36.46	35.13	84	4	FAN	REST1	28	50
	18	36.48	36.43	35.12	68	4	FAN	REST1	28	50
	19	36.49	36.43	35.11	67	4	FAN	REST1	28	50
	20	36.49	36.45	35.10	76	4	FAN	REST1	28	50
	21	36.49	36.45	35.10	72	4	FAN	REST1	28	50
	22	36.48	36.46	35.10	70	4	FAN	REST1	28	50
	23	36.48	36.47	35.10	67	4	FAN	REST1	28	50
	24	36.48	36.45	35.09	67	4	FAN	REST1	28	50
	25	36.47	36.44	35.09	68	4	FAN	REST1	28	50
	26	36.47	36.45	35.09	68	4	FAN	REST1	28	50
	27	36.48	36.45	35.09	67	4	FAN	REST1	28	50
	28	36.48	36.45	35.10	70	4	FAN	REST1	28	50
	29	36.47	36.45	35.09	67	4	FAN	REST1	28	50
	30	36.47	36.46	35.09	66	4	FAN	REST1	28	50
5	31	36.46	36.45	35.08	67	4	FAN	REST1	28	50
	32	36.46	36.44	35.08	67	4	FAN	REST1	28	50
	33	36.47	36.45	35.09	67	4	FAN	REST1	28	50
	34	36.47	36.43	35.07	68	4	FAN	REST1	28	50
	35	36.46	36.42	35.05	67	4	FAN	REST1	28	50
	36	36.45	36.42	35.05	66	4	FAN	REST1	28	50
	37	36.44	36.42	35.05	67	4	FAN	REST1	28	50
	38	36.45	36.44	35.05	67	4	FAN	REST1	28	50
	39	36.45	36.43	35.04	68	4	FAN	REST1	28	50
	40	36.45	36.43	35.04	65	4	FAN	REST1	28	50
	41	36.44	36.44	35.04	73	4	FAN	REST1	28	50
	42	36.44	36.42	35.04	69	4	FAN	REST1	28	50
	43	36.45	36.41	35.03	67	4	FAN	REST1	28	50
	44	36.45	36.42	35.03	67	4	FAN	REST1	28	50
	45	36.45	36.42	35.02	66	4	FAN	REST1	28	50
	46	36.44	36.40	35.02	68	4	FAN	REST1	28	50
	47	36.42	36.40	35.02	70	4	FAN	REST1	28	50
	48	36.42	36.38	35.02	69	4	FAN	REST1	28	50
	49	36.42	36.36	35.01	66	4	FAN	REST1	28	50
	50	36.43	36.36	35.01	73	4	FAN	REST1	28	50
	51	36.43	36.37	35.02	66	4	FAN	REST1	28	50
	52	36.42	36.39	35.01	66	4	FAN	REST1	28	50
	53	36.42	36.38	35.01	67	4	FAN	REST1	28	50
	54	36.42	36.37	35.00	66	4	FAN	REST1	28	50
	55	36.42	36.38	35.00	66	4	FAN	REST1	28	50
	56	36.43	36.40	35.02	64	4	FAN	REST1	28	50
	57	36.42	36.39	35.02	67	4	FAN	REST1	28	50
	58	36.41	36.39	35.03	69	4	FAN	REST1	28	50
	59	36.41	36.39	35.04	69	4	FAN	REST1	28	50
	60	36.40	36.38	35.04	67	4	FAN	REST1	28	50
10	61	36.40	36.37	35.04	68	4	FAN	REST1	28	50

min	number	Tre	Tes	Tsk-head	HR	ID	condition	period	Ta	RH
	62	36.40	36.38	35.05	67	4	FAN	REST1	28	50
	63	36.41	36.38	35.05	66	4	FAN	REST1	28	50
	64	36.41	36.39	35.05	66	4	FAN	REST1	28	50
	65	36.42	36.40	35.05	64	4	FAN	REST1	28	50
	66	36.42	36.38	35.05	64	4	FAN	REST1	28	50
	67	36.41	36.39	35.05	66	4	FAN	REST1	28	50
	68	36.41	36.41	35.05	66	4	FAN	REST1	28	50
	69	36.42	36.42	35.05	66	4	FAN	REST1	28	50
	70	36.43	36.40	35.05	65	4	FAN	REST1	28	50
	71	36.42	36.39	35.05	71	4	FAN	REST1	28	50
	72	36.41	36.41	35.05	71	4	FAN	REST1	28	50
	73	36.42	36.42	35.05	70	4	FAN	REST1	28	50
	74	36.41	36.40	35.05	67	4	FAN	REST1	28	50
	75	36.40	36.41	35.06	66	4	FAN	REST1	28	50
	76	36.40	36.41	35.06	68	4	FAN	REST1	28	50
	77	36.40	36.40	35.05	64	4	FAN	REST1	28	50
	78	36.39	36.42	35.05	66	4	FAN	REST1	28	50
	79	36.38	36.41	35.05	65	4	FAN	REST1	28	50
	80	36.39	36.41	35.05	67	4	FAN	REST1	28	50
	81	36.39	36.40	35.05	69	4	FAN	REST1	28	50
	82	36.38	36.40	35.05	68	4	FAN	REST1	28	50
	83	36.38	36.41	35.05	69	4	FAN	REST1	28	50
	84	36.39	36.41	35.06	68	4	FAN	REST1	28	50
	85	36.40	36.43	35.06	68	4	FAN	REST1	28	50
	86	36.41	36.42	35.06	68	4	FAN	REST1	28	50
	87	36.41	36.42	35.06	70	4	FAN	REST1	28	50
	88	36.41	36.44	35.07	71	4	FAN	REST1	28	50
	89	36.42	36.45	35.07	67	4	FAN	REST1	28	50
	90	36.42	36.45	35.07	69	4	FAN	REST1	28	50
15	91	36.41	36.43	35.07	69	4	FAN	REST1	28	50
	92	36.39	36.43	35.08	71	4	FAN	REST1	28	50
	93	36.39	36.44	35.08	71	4	FAN	REST1	28	50
	94	36.40	36.43	35.07	69	4	FAN	REST1	28	50
	95	36.40	36.43	35.07	71	4	FAN	REST1	28	50
	96	36.40	36.46	35.08	78	4	FAN	REST1	28	50
	97	36.40	36.44	35.09	72	4	FAN	REST1	28	50
	98	36.40	36.41	35.09	69	4	FAN	REST1	28	50
	99	36.40	36.40	35.09	75	4	FAN	REST1	28	50
	100	36.41	36.41	35.08	76	4	FAN	REST1	28	50
	101	36.43	36.43	35.08	77	4	FAN	REST1	28	50
	102	36.44	36.42	35.08	84	4	FAN	REST1	28	50
	103	36.43	36.42	35.04	77	4	FAN	REST1	40	50
	104	36.44	36.44		84	4	FAN	REST1	40	50
	105	36.46	36.44		90	4	FAN	REST1	40	50
	106	36.47	36.42		76	4	FAN	REST1	40	50
	107	36.48	36.43		77	4	FAN	REST1	40	50

min	number	Tre	Tes	Tsk-head	HR	ID	condition	period	Ta	RH
	108	36.49	36.43		75	4	FAN	REST1	40	50
	109	36.49	36.41		74	4	FAN	REST1	40	50
	110	36.49	36.43		79	4	FAN	REST1	40	50
	111	36.48	36.42		78	4	FAN	REST1	40	50
	112	36.47	36.41		76	4	FAN	REST1	40	50
	113	36.47	36.42		76	4	FAN	REST1	40	50
	114	36.47	36.39		77	4	FAN	REST1	40	50
	115	36.46	36.36		78	4	FAN	REST1	40	50
	116	36.46	36.36		81	4	FAN	REST1	40	50
	117	36.46	36.38		76	4	FAN	REST1	40	50
	118	36.47	36.39		75	4	FAN	REST1	40	50
	119	36.47	36.41	37.19	74	4	FAN	REST1	40	50
	120	36.47	36.41	37.21	77	4	FAN	REST1	40	50
20	121	36.47	36.40	37.69	91	4	FAN	REST1	40	50
	122	36.47	36.40	36.74	78	4	FAN	REST1	40	50
	123	36.47	36.41	35.97	87	4	FAN	REST1	40	50
	124	36.48	36.42	35.97	90	4	FAN	REST1	40	50
	125	36.49	36.40	36.01	79	4	FAN	REST1	40	50
	126	36.48	36.40	36.06	80	4	FAN	REST1	40	50
	127	36.48	36.41	36.09	82	4	FAN	REST1	40	50
	128	36.49	36.42	36.10	84	4	FAN	REST1	40	50
	129	36.49	36.37	36.10	89	4	FAN	REST1	40	50
	130	36.49	36.34	36.10	81	4	FAN	REST1	40	50
	131	36.49	36.36	36.10	79	4	FAN	REST1	40	50
	132	36.49	36.35	36.11	79	4	FAN	REST1	40	50
	133	36.49	36.37	36.14	75	4	FAN	REST1	40	50
	134	36.49	36.38	36.16	74	4	FAN	REST1	40	50
	135	36.50	36.37	36.17	76	4	FAN	REST1	40	50
	136	36.50	36.36	36.18	77	4	FAN	REST1	40	50
	137	36.50	36.36	36.19	77	4	FAN	REST1	40	50
	138	36.50	36.37	36.19	77	4	FAN	REST1	40	50
	139	36.50	36.39	36.19	78	4	FAN	EXERCISE1	40	50
	140	36.50	36.38	36.19	83	4	FAN	EXERCISE1	40	50
	141	36.50	36.40	36.19	88	4	FAN	EXERCISE1	40	50
	142	36.50	36.44	36.20	91	4	FAN	EXERCISE1	40	50
	143	36.50	36.43	36.21	94	4	FAN	EXERCISE1	40	50
	144	36.49	36.40	36.20	102	4	FAN	EXERCISE1	40	50
	145	36.51	36.41	36.22	102	4	FAN	EXERCISE1	40	50
	146	36.52	36.43	36.24	99	4	FAN	EXERCISE1	40	50
	147	36.51	36.42	36.24	104	4	FAN	EXERCISE1	40	50
	148	36.51	36.41	36.25	106	4	FAN	EXERCISE1	40	50
	149	36.52	36.41	36.27	106	4	FAN	EXERCISE1	40	50
	150	36.53	36.42	36.28	108	4	FAN	EXERCISE1	40	50
25	151	36.52	36.42	36.29	102	4	FAN	EXERCISE1	40	50
	152	36.53	36.44	36.29	102	4	FAN	EXERCISE1	40	50
	153	36.53	36.45	36.30	104	4	FAN	EXERCISE1	40	50

min	number	Tre	Tes	Tsk-head	HR	ID	condition	period	Ta	RH
	154	36.52	36.44	36.30	103	4	FAN	EXERCISE1	40	50
	155	36.53	36.47	36.32	106	4	FAN	EXERCISE1	40	50
	156	36.53	36.47	36.32	107	4	FAN	EXERCISE1	40	50
	157	36.53	36.47	36.33	106	4	FAN	EXERCISE1	40	50
	158	36.53	36.50	36.34	105	4	FAN	EXERCISE1	40	50
	159	36.53	36.49	36.35	105	4	FAN	EXERCISE1	40	50
	160	36.53	36.49	36.36	105	4	FAN	EXERCISE1	40	50
	161	36.54	36.53	36.38	104	4	FAN	EXERCISE1	40	50
	162	36.54	36.53	36.38	105	4	FAN	EXERCISE1	40	50
	163	36.55	36.53	36.39	108	4	FAN	EXERCISE1	40	50
	164	36.56	36.55	36.41	104	4	FAN	EXERCISE1	40	50
	165	36.56	36.56	36.42	106	4	FAN	EXERCISE1	40	50
	166	36.57	36.57	36.43	108	4	FAN	EXERCISE1	40	50
	167	36.57	36.60	36.45	111	4	FAN	EXERCISE1	40	50
	168	36.57	36.62	36.46	110	4	FAN	EXERCISE1	40	50
	169	36.58	36.64	36.48	109	4	FAN	EXERCISE1	40	50
	170	36.59	36.65	36.50	105	4	FAN	EXERCISE1	40	50
	171	36.60	36.66	36.50	108	4	FAN	EXERCISE1	40	50
	172	36.61	36.68	36.51	108	4	FAN	EXERCISE1	40	50
	173	36.61	36.69	36.52	112	4	FAN	EXERCISE1	40	50
	174	36.62	36.69	36.52	110	4	FAN	EXERCISE1	40	50
	175	36.63	36.68	36.52	112	4	FAN	EXERCISE1	40	50
	176	36.64	36.69	36.54	111	4	FAN	EXERCISE1	40	50
	177	36.64	36.70	36.55	111	4	FAN	EXERCISE1	40	50
	178	36.65	36.72	36.56	110	4	FAN	EXERCISE1	40	50
	179	36.66	36.74	36.57	113	4	FAN	EXERCISE1	40	50
	180	36.67	36.74	36.58	111	4	FAN	EXERCISE1	40	50
30	181	36.67	36.76	36.59	110	4	FAN	EXERCISE1	40	50
	182	36.68	36.80	36.60	110	4	FAN	EXERCISE1	40	50
	183	36.69	36.79	36.60	111	4	FAN	EXERCISE1	40	50
	184	36.69	36.79	36.61	111	4	FAN	EXERCISE1	40	50
	185	36.70	36.80	36.63	110	4	FAN	EXERCISE1	40	50
	186	36.70	36.80	36.64	112	4	FAN	EXERCISE1	40	50
	187	36.70	36.81	36.64	114	4	FAN	EXERCISE1	40	50
	188	36.71	36.83	36.66	114	4	FAN	EXERCISE1	40	50
	189	36.72	36.85	36.68	116	4	FAN	EXERCISE1	40	50
	190	36.73	36.85	36.69	116	4	FAN	EXERCISE1	40	50
	191	36.73	36.86	36.71	116	4	FAN	EXERCISE1	40	50
	192	36.73	36.86	36.73	116	4	FAN	EXERCISE1	40	50
	193	36.74	36.87	36.73	119	4	FAN	EXERCISE1	40	50
	194	36.75	36.86	36.73	120	4	FAN	EXERCISE1	40	50
	195	36.75	36.84	36.73	117	4	FAN	EXERCISE1	40	50
	196	36.75	36.85	36.75	113	4	FAN	EXERCISE1	40	50
	197	36.76	36.88	36.74	111	4	FAN	EXERCISE1	40	50
	198	36.75	36.91	36.73	112	4	FAN	EXERCISE1	40	50
	199	36.75	36.90	36.74	113	4	FAN	EXERCISE1	40	50

min	number	Tre	Tes	Tsk-head	HR	ID	condition	period	Ta	RH
	200	36.76	36.89	36.75	114	4	FAN	EXERCISE1	40	50
	201	36.77	36.92	36.76	115	4	FAN	EXERCISE1	40	50
	202	36.78	36.92	36.76	116	4	FAN	EXERCISE1	40	50
	203	36.79	36.91	36.76	117	4	FAN	EXERCISE1	40	50
	204	36.78	36.92	36.76	119	4	FAN	EXERCISE1	40	50
	205	36.78	36.93	36.77	120	4	FAN	EXERCISE1	40	50
	206	36.79	36.89	36.78	118	4	FAN	EXERCISE1	40	50
	207	36.79	36.89	36.80	116	4	FAN	EXERCISE1	40	50
	208	36.81	36.93	36.81	116	4	FAN	EXERCISE1	40	50
	209	36.82	36.95	36.81	113	4	FAN	EXERCISE1	40	50
	210	36.81	36.95	36.81	114	4	FAN	EXERCISE1	40	50
35	211	36.80	36.96	36.82	116	4	FAN	EXERCISE1	40	50
	212	36.82	36.96	36.83	117	4	FAN	EXERCISE1	40	50
	213	36.82	36.97	36.84	117	4	FAN	EXERCISE1	40	50
	214	36.82	36.97	36.83	116	4	FAN	EXERCISE1	40	50
	215	36.83	36.96	36.82	117	4	FAN	EXERCISE1	40	50
	216	36.84	36.98	36.83	115	4	FAN	EXERCISE1	40	50
	217	36.84	36.99	36.84	116	4	FAN	EXERCISE1	40	50
	218	36.84	36.98	36.84	117	4	FAN	EXERCISE1	40	50
	219	36.85	36.98	36.84	116	4	FAN	EXERCISE1	40	50
	220	36.85	36.97	36.85	116	4	FAN	EXERCISE1	40	50
	221	36.86	36.98	36.86	120	4	FAN	EXERCISE1	40	50
	222	36.86	37.00	36.86	121	4	FAN	EXERCISE1	40	50
	223	36.86	37.02	36.86	121	4	FAN	EXERCISE1	40	50
	224	36.86	37.02	36.87	121	4	FAN	EXERCISE1	40	50
	225	36.87	37.01	36.87	121	4	FAN	EXERCISE1	40	50
	226	36.88	37.01	36.87	119	4	FAN	EXERCISE1	40	50
	227	36.88	37.01	36.87	121	4	FAN	EXERCISE1	40	50
	228	36.87	37.01	36.88	119	4	FAN	EXERCISE1	40	50
	229	36.87	37.03	36.89	119	4	FAN	EXERCISE1	40	50
	230	36.88	37.05	36.90	119	4	FAN	EXERCISE1	40	50
	231	36.89	37.05	36.91	119	4	FAN	EXERCISE1	40	50
	232	36.89	37.05	36.92	119	4	FAN	EXERCISE1	40	50
	233	36.90	37.05	36.92	117	4	FAN	EXERCISE1	40	50
	234	36.91	37.07	36.93	117	4	FAN	EXERCISE1	40	50
	235	36.91	37.08	36.94	118	4	FAN	EXERCISE1	40	50
	236	36.92	37.08	36.94	119	4	FAN	EXERCISE1	40	50
	237	36.92	37.07	36.94	119	4	FAN	EXERCISE1	40	50
	238	36.91	37.06	36.94	119	4	FAN	EXERCISE1	40	50
	239	36.91	37.07	36.94	118	4	FAN	EXERCISE1	40	50
	240	36.92	37.10	36.95	119	4	FAN	EXERCISE1	40	50
40	241	36.94	37.10	36.94	119	4	FAN	EXERCISE1	40	50
	242	36.95	37.08	36.94	119	4	FAN	EXERCISE1	40	50
	243	36.96	37.10	36.95	120	4	FAN	EXERCISE1	40	50
	244	36.97	37.10	36.94	121	4	FAN	EXERCISE1	40	50
	245	36.97	37.09	36.94	124	4	FAN	EXERCISE1	40	50

min	number	Tre	Tes	Tsk-head	HR	ID	condition	period	Ta	RH
	246	36.97	37.10	36.95	120	4	FAN	EXERCISE1	40	50
	247	36.97	37.10	36.96	121	4	FAN	EXERCISE1	40	50
	248	36.97	37.11	36.97	121	4	FAN	EXERCISE1	40	50
	249	36.97	37.13	36.98	121	4	FAN	EXERCISE1	40	50
	250	36.99	37.13	36.98	122	4	FAN	EXERCISE1	40	50
	251	37.00	37.12	36.98	123	4	FAN	EXERCISE1	40	50
	252	37.00	37.14	36.99	124	4	FAN	EXERCISE1	40	50
	253	37.00	37.15	36.99	121	4	FAN	EXERCISE1	40	50
	254	37.00	37.16	36.99	120	4	FAN	EXERCISE1	40	50
	255	37.00	37.16	36.98	121	4	FAN	EXERCISE1	40	50
	256	37.00	37.16	36.97	117	4	FAN	EXERCISE1	40	50
	257	37.02	37.19	36.98	121	4	FAN	EXERCISE1	40	50
	258	37.03	37.25	36.99	118	4	FAN	EXERCISE1	40	50
	259	37.03	37.19	36.99	124	4	FAN	EXERCISE1	40	50
	260	37.02	37.12	36.99	121	4	FAN	EXERCISE1	40	50
	261	37.03	37.15	37.00	121	4	FAN	EXERCISE1	40	50
	262	37.05	37.16	37.01	122	4	FAN	EXERCISE1	40	50
	263	37.05	37.17	37.01	119	4	FAN	EXERCISE1	40	50
	264	37.05	37.18	37.01	120	4	FAN	EXERCISE1	40	50
	265	37.06	37.19	37.03	121	4	FAN	EXERCISE1	40	50
	266	37.06	37.19	37.03	123	4	FAN	EXERCISE1	40	50
	267	37.06	37.18	37.03	121	4	FAN	EXERCISE1	40	50
	268	37.08	37.18	37.02	125	4	FAN	EXERCISE1	40	50
	269	37.09	37.20	37.03	126	4	FAN	EXERCISE1	40	50
	270	37.09	37.22	37.05	128	4	FAN	EXERCISE1	40	50
45	271	37.09	37.20	37.04	128	4	FAN	EXERCISE1	40	50
	272	37.09	37.19	37.05	129	4	FAN	EXERCISE1	40	50
	273	37.10	37.19	37.07	131	4	FAN	EXERCISE1	40	50
	274	37.09	37.19	37.05	129	4	FAN	EXERCISE1	40	50
	275	37.09	37.20	37.04	129	4	FAN	EXERCISE1	40	50
	276	37.10	37.20	37.04	125	4	FAN	EXERCISE1	40	50
	277	37.11	37.21	37.04	124	4	FAN	EXERCISE1	40	50
	278	37.12	37.20	37.04	127	4	FAN	EXERCISE1	40	50
	279	37.13	37.22	37.04	130	4	FAN	EXERCISE1	40	50
	280	37.14	37.21	37.04	127	4	FAN	EXERCISE1	40	50
	281	37.15	37.20	37.03	125	4	FAN	EXERCISE1	40	50
	282	37.14	37.22	37.03	126	4	FAN	EXERCISE1	40	50
	283	37.14	37.21	37.04	128	4	FAN	EXERCISE1	40	50
	284	37.15	37.21	37.06	129	4	FAN	EXERCISE1	40	50
	285	37.15	37.22	37.06	130	4	FAN	EXERCISE1	40	50
	286	37.16	37.25	37.06	131	4	FAN	EXERCISE1	40	50
	287	37.17	37.27	37.08	130	4	FAN	EXERCISE1	40	50
	288	37.17	37.28	37.08	128	4	FAN	EXERCISE1	40	50
	289	37.17	37.29	37.07	130	4	FAN	EXERCISE1	40	50
	290	37.17	37.29	37.08	126	4	FAN	EXERCISE1	40	50
	291	37.18	37.27	37.09	125	4	FAN	EXERCISE1	40	50

min	number	Tre	Tes	Tsk-head	HR	ID	condition	period	Ta	RH
	292	37.19	37.27	37.09	126	4	FAN	EXERCISE1	40	50
	293	37.19	37.28	37.08	125	4	FAN	EXERCISE1	40	50
	294	37.19	37.27	37.08	127	4	FAN	EXERCISE1	40	50
	295	37.19	37.27	37.09	129	4	FAN	EXERCISE1	40	50
	296	37.20	37.30	37.09	130	4	FAN	EXERCISE1	40	50
	297	37.22	37.30	37.10	129	4	FAN	EXERCISE1	40	50
	298	37.23	37.30	37.11	128	4	FAN	EXERCISE1	40	50
	299	37.23	37.30	37.11	131	4	FAN	EXERCISE1	40	50
	300	37.23	37.31	37.12	132	4	FAN	EXERCISE1	40	50
50	301	37.23	37.31	37.13	130	4	FAN	EXERCISE1	40	50
	302	37.24	37.32	37.14	129	4	FAN	EXERCISE1	40	50
	303	37.25	37.33	37.15	129	4	FAN	EXERCISE1	40	50
	304	37.25	37.34	37.16	127	4	FAN	EXERCISE1	40	50
	305	37.26	37.35	37.16	129	4	FAN	EXERCISE1	40	50
	306	37.26	37.32	37.15	128	4	FAN	EXERCISE1	40	50
	307	37.26	37.35	37.15	126	4	FAN	EXERCISE1	40	50
	308	37.27	37.36	37.16	127	4	FAN	EXERCISE1	40	50
	309	37.27	37.36	37.17	127	4	FAN	EXERCISE1	40	50
	310	37.28	37.37	37.18	128	4	FAN	EXERCISE1	40	50
	311	37.28	37.38	37.18	131	4	FAN	EXERCISE1	40	50
	312	37.29	37.38	37.17	132	4	FAN	EXERCISE1	40	50
	313	37.29	37.38	37.18	130	4	FAN	EXERCISE1	40	50
	314	37.29	37.38	37.18	129	4	FAN	EXERCISE1	40	50
	315	37.30	37.41	37.18	129	4	FAN	EXERCISE1	40	50
	316	37.30	37.42	37.17	128	4	FAN	EXERCISE1	40	50
	317	37.30	37.35	37.19	131	4	FAN	EXERCISE1	40	50
	318	37.31	37.34	37.20	130	4	FAN	EXERCISE1	40	50
	319	37.31	37.38	37.19	131	4	FAN	EXERCISE1	40	50
	320	37.32	37.43	37.19	126	4	FAN	REST2	28	50
	321	37.32	37.44	37.14	124	4	FAN	REST2	28	50
	322	37.33	37.43	36.96	115	4	FAN	REST2	28	50
	323	37.34	37.45	36.36	111	4	FAN	REST2	28	50
	324	37.33	37.46		111	4	FAN	REST2	28	50
	325	37.33	37.49		111	4	FAN	REST2	28	50
	326	37.33	37.51		106	4	FAN	REST2	28	50
	327	37.32	37.53		104	4	FAN	REST2	28	50
	328	37.32	37.56		104	4	FAN	REST2	28	50
	329	37.32	37.40	34.51	109	4	FAN	REST2	28	50
	330	37.31	37.32	35.50	103	4	FAN	REST2	28	50
55	331	37.30	37.42	35.79	101	4	FAN	REST2	28	50
	332	37.30	37.47	35.92	101	4	FAN	REST2	28	50
	333	37.29	37.50	36.02	98	4	FAN	REST2	28	50
	334	37.29	37.51	36.13	95	4	FAN	REST2	28	50
	335	37.28	37.49	36.18	97	4	FAN	REST2	28	50
	336	37.27	37.40	36.19	101	4	FAN	REST2	28	50
	337	37.26	37.35	36.18	95	4	FAN	REST2	28	50

min	number	Tre	Tes	Tsk-head	HR	ID	condition	period	Ta	RH
	338	37.26	37.34	36.15	92	4	FAN	REST2	28	50
	339	37.25	32.24	36.11	93	4	FAN	REST2	28	50
	340	37.23	29.46	36.10	102	4	FAN	REST2	28	50
	341	37.22	32.57	36.09	98	4	FAN	REST2	28	50
	342	37.21	33.58	36.09	97	4	FAN	REST2	28	50
	343	37.20	33.99	36.09	92	4	FAN	REST2	28	50
	344	37.19	34.33	36.10	91	4	FAN	REST2	28	50
	345	37.18	34.75	36.14	96	4	FAN	REST2	28	50
	346	37.17	31.43	36.11	101	4	FAN	REST2	28	50
	347	37.17	27.02	36.08	99	4	FAN	REST2	28	50
	348	37.16	26.05	36.07	100	4	FAN	REST2	28	50
	349	37.14	26.13	36.04	96	4	FAN	REST2	28	50
	350	37.13	26.38	36.01	97	4	FAN	REST2	28	50
	351	37.13	26.34	36.01	97	4	FAN	REST2	28	50
	352	37.13	27.62	35.99	97	4	FAN	REST2	28	50
	353	37.12	28.30	35.95	98	4	FAN	REST2	28	50
	354	37.10	27.32	35.89	97	4	FAN	REST2	28	50
	355	37.10	27.28	35.85	92	4	FAN	REST2	28	50
	356	37.09	28.91	35.83	94	4	FAN	REST2	28	50
	357	37.08	30.30	35.81	89	4	FAN	REST2	28	50
	358	37.06	28.64	35.80	88	4	FAN	REST2	28	50
	359	37.05	27.10	35.79	92	4	FAN	REST2	28	50
	360	37.04	28.95	35.76	90	4	FAN	REST2	28	50
60	361	37.03	30.60	35.72	92	4	FAN	REST2	28	50
	362	37.03	28.99	35.72	94	4	FAN	REST2	28	50
	363	37.03	29.06	35.68	93	4	FAN	REST2	28	50
	364	37.04	30.94	35.61	85	4	FAN	REST2	28	50
	365	37.03	31.48	35.57	83	4	FAN	REST2	28	50
	366	37.04	31.86	35.58	85	4	FAN	REST2	28	50
	367	37.05	32.10	35.58	85	4	FAN	REST2	28	50
	368	37.08	32.72	35.59	98	4	FAN	REST2	28	50
	369	37.10	33.35	35.58	90	4	FAN	REST2	28	50
	370	37.11	33.72	35.56	83	4	FAN	REST2	28	50
	371	37.17	34.01	35.56	88	4	FAN	REST2	28	50
	372	37.24	34.28	35.56	88	4	FAN	REST2	28	50
	373	37.26	34.65	35.56	105	4	FAN	REST2	28	50
	374	37.26	34.91	35.52	81	4	FAN	REST2	28	50
	375	37.27	35.00	35.50	80	4	FAN	REST2	28	50
	376	37.27	35.08	35.46	81	4	FAN	REST2	28	50
	377	37.26	35.21	35.42	86	4	FAN	REST2	28	50
	378	37.26	35.33	35.44	81	4	FAN	REST2	28	50
	379	37.26	35.40	35.44	82	4	FAN	REST2	28	50
	380	37.25	35.49	35.42	80	4	FAN	REST2	28	50
	381	37.24	35.57	35.42	83	4	FAN	REST2	28	50
	382	37.24	35.67	35.42	84	4	FAN	REST2	28	50
	383	37.22	35.74	35.39	80	4	FAN	REST2	28	50

min	number	Tre	Tes	Tsk-head	HR	ID	condition	period	Ta	RH
	384	37.19	35.80	35.37	79	4	FAN	REST2	28	50
	385	37.17	35.87	35.36	76	4	FAN	REST2	28	50
	386	37.16	35.93	35.35	76	4	FAN	REST2	28	50
	387	37.16	35.98	35.33	79	4	FAN	REST2	28	50
	388	37.15	36.03	35.29	84	4	FAN	REST2	28	50
	389	37.14	36.08	35.26	81	4	FAN	REST2	28	50
	390	37.14	36.12	35.24	83	4	FAN	REST2	28	50
65	391	37.14	36.14	35.22	77	4	FAN	REST2	28	50
	392	37.13	36.16	35.20	78	4	FAN	REST2	28	50
	393	37.13	36.20	35.19	80	4	FAN	REST2	28	50
	394	37.13	36.22	35.20	84	4	FAN	REST2	28	50
	395	37.13	36.24	35.21	81	4	FAN	REST2	28	50
	396	37.12	36.26	35.21	81	4	FAN	REST2	28	50
	397	37.11	36.30	35.20	84	4	FAN	REST2	28	50
	398	37.10	36.33	35.21	84	4	FAN	REST2	28	50
	399	37.08	36.33	35.21	80	4	FAN	REST2	28	50
	400	37.06	36.33	35.20	79	4	FAN	REST2	28	50
	401	37.06	36.36	35.21	79	4	FAN	REST2	28	50
	402	37.05	36.38	35.24	79	4	FAN	REST2	28	50
	403	37.04	36.40	35.24	78	4	FAN	REST2	28	50
	404	37.03	36.42	35.21	81	4	FAN	REST2	28	50
	405	37.02	36.42	35.19	79	4	FAN	REST2	28	50
	406	37.01	36.44	35.19	79	4	FAN	REST2	28	50
	407	37.01	36.46	35.22	79	4	FAN	REST2	28	50
	408	37.01	36.46	35.20	84	4	FAN	REST2	28	50
	409	37.00	36.49	35.19	80	4	FAN	REST2	28	50
	410	37.00	36.50	35.18	77	4	FAN	REST2	28	50
	411	36.99	36.50	35.16	79	4	FAN	REST2	28	50
	412	36.98	36.51	35.16	77	4	FAN	REST2	28	50
	413	36.98	35.99	35.19	78	4	FAN	REST2	28	50
	414	36.99	35.50	35.19	80	4	FAN	REST2	28	50
	415	36.98	35.81	35.18	78	4	FAN	REST2	28	50
	416	36.98	36.14	35.17	79	4	FAN	REST2	28	50
	417	36.97	36.24	35.15	78	4	FAN	REST2	28	50
	418	36.96	36.32	35.14	82	4	FAN	REST2	28	50
	419	36.96	36.36	35.16	78	4	FAN	REST2	28	50
	420	36.96	36.40	35.16	81	4	FAN	REST2	28	50
70	421	36.97	36.43	35.15	80	4	FAN	REST2	28	50
	422	36.97	36.47	35.14	86	4	FAN	REST2	28	50
	423	36.96	36.50	35.13	83	4	FAN	REST2	28	50
	424	36.94	36.51	35.10	79	4	FAN	REST2	28	50
	425	36.94	36.55	35.08	78	4	FAN	REST2	28	50
	426	36.93	36.57	35.07	78	4	FAN	REST2	28	50
	427	36.92	36.59	35.09	82	4	FAN	REST2	28	50
	428	36.92	36.59	35.14	79	4	FAN	REST2	28	50
	429	36.91	36.58	35.14	79	4	FAN	REST2	28	50

min	number	Tre	Tes	Tsk-head	HR	ID	condition	period	Ta	RH
	430	36.90	36.60	35.14	81	4	FAN	REST2	28	50
	431	36.92	36.63	35.11	85	4	FAN	REST2	28	50
	432	36.93	36.65	35.05	82	4	FAN	REST2	28	50
	433	36.92	36.67	35.04	77	4	FAN	REST2	28	50
	434	36.91	36.66	35.05	78	4	FAN	REST2	28	50
	435	36.91	36.66	35.05	80	4	FAN	REST2	28	50
	436	36.92	36.69	35.05	80	4	FAN	REST2	28	50
	437	36.91	36.69	35.03	86	4	FAN	REST2	28	50
	438	36.90	36.66	35.02	82	4	FAN	REST2	28	50
	439	36.90	36.66	35.01	85	4	FAN	REST2	40	50
	440	36.91	36.67	35.10	89	4	FAN	REST2	40	50
	441	36.92	36.67	35.27	89	4	FAN	REST2	40	50
	442	36.93	36.66	35.39	95	4	FAN	REST2	40	50
	443	36.93	36.67	35.46	85	4	FAN	REST2	40	50
	444	36.94	36.67	35.52	84	4	FAN	REST2	40	50
	445	36.94	36.65	35.60	83	4	FAN	REST2	40	50
	446	36.94	36.64	35.66	86	4	FAN	REST2	40	50
	447	36.94	36.63	35.69	85	4	FAN	REST2	40	50
	448	36.95	36.64	35.74	86	4	FAN	REST2	40	50
	449	36.96	36.64	35.78	79	4	FAN	REST2	40	50
	450	36.95	36.64	35.81	83	4	FAN	REST2	40	50
75	451	36.95	36.68	35.84	85	4	FAN	REST2	40	50
	452	36.96	36.70	35.87	86	4	FAN	REST2	40	50
	453	36.96	36.67	35.89	80	4	FAN	REST2	40	50
	454	36.95	36.65	35.92	77	4	FAN	REST2	40	50
	455	36.94	36.66	35.93	79	4	FAN	REST2	40	50
	456	36.95	36.67	35.96	79	4	FAN	REST2	40	50
	457	36.97	36.69	35.99	89	4	FAN	REST2	40	50
	458	36.98	36.71	36.02	95	4	FAN	REST2	40	50
	459	36.97	36.69	36.04	92	4	FAN	REST2	40	50
	460	36.96	36.67	36.03	91	4	FAN	REST2	40	50
	461	36.97	36.68	36.05	90	4	FAN	REST2	40	50
	462	37.00	36.67	36.09	89	4	FAN	REST2	40	50
	463	37.02	36.66	36.11	91	4	FAN	EXERCISE2	40	50
	464	37.04	36.66	36.13	89	4	FAN	EXERCISE2	40	50
	465	37.05	36.63	36.16	97	4	FAN	EXERCISE2	40	50
	466	37.05	36.63	36.17	96	4	FAN	EXERCISE2	40	50
	467	37.05	36.57	36.19	100	4	FAN	EXERCISE2	40	50
	468	37.03	36.41	36.20	103	4	FAN	EXERCISE2	40	50
	469	37.04	36.48	36.22	103	4	FAN	EXERCISE2	40	50
	470	37.05	36.63	36.25	105	4	FAN	EXERCISE2	40	50
	471	37.05	36.66	36.27	106	4	FAN	EXERCISE2	40	50
	472	37.05	36.69	36.29	107	4	FAN	EXERCISE2	40	50
	473	37.05	36.70	36.33	110	4	FAN	EXERCISE2	40	50
	474	37.05	36.69	36.35	108	4	FAN	EXERCISE2	40	50
	475	37.06	36.71	36.37	109	4	FAN	EXERCISE2	40	50

min	number	Tre	Tes	Tsk-head	HR	ID	condition	period	Ta	RH
	476	37.06	36.74	36.40	110	4	FAN	EXERCISE2	40	50
	477	37.06	36.76	36.44	110	4	FAN	EXERCISE2	40	50
	478	37.06	36.76	36.47	114	4	FAN	EXERCISE2	40	50
	479	37.05	36.77	36.48	110	4	FAN	EXERCISE2	40	50
	480	37.05	36.78	36.50	111	4	FAN	EXERCISE2	40	50
80	481	37.04	36.78	36.54	113	4	FAN	EXERCISE2	40	50
	482	37.05	36.80	36.56	111	4	FAN	EXERCISE2	40	50
	483	37.05	36.82	36.58	112	4	FAN	EXERCISE2	40	50
	484	37.05	36.82	36.59	113	4	FAN	EXERCISE2	40	50
	485	37.05	36.84	36.60	111	4	FAN	EXERCISE2	40	50
	486	37.05	36.85	36.63	113	4	FAN	EXERCISE2	40	50
	487	37.05	36.86	36.65	115	4	FAN	EXERCISE2	40	50
	488	37.07	36.88	36.66	114	4	FAN	EXERCISE2	40	50
	489	37.08	36.91	36.67	111	4	FAN	EXERCISE2	40	50
	490	37.09	36.93	36.68	112	4	FAN	EXERCISE2	40	50
	491	37.09	36.93	36.69	115	4	FAN	EXERCISE2	40	50
	492	37.09	36.94	36.70	116	4	FAN	EXERCISE2	40	50
	493	37.09	36.95	36.71	117	4	FAN	EXERCISE2	40	50
	494	37.09	36.94	36.73	118	4	FAN	EXERCISE2	40	50
	495	37.11	36.93	36.76	120	4	FAN	EXERCISE2	40	50
	496	37.13	36.94	36.78	121	4	FAN	EXERCISE2	40	50
	497	37.14	36.97	36.77	121	4	FAN	EXERCISE2	40	50
	498	37.16	36.98	36.80	120	4	FAN	EXERCISE2	40	50
	499	37.16	36.98	36.82	120	4	FAN	EXERCISE2	40	50
	500	37.16	36.99	36.82	118	4	FAN	EXERCISE2	40	50
	501	37.17	36.97	36.84	122	4	FAN	EXERCISE2	40	50
	502	37.17	36.97	36.86	121	4	FAN	EXERCISE2	40	50
	503	37.17	37.00	36.87	119	4	FAN	EXERCISE2	40	50
	504	37.17	37.03	36.87	120	4	FAN	EXERCISE2	40	50
	505	37.18	37.03	36.88	120	4	FAN	EXERCISE2	40	50
	506	37.18	37.02	36.90	116	4	FAN	EXERCISE2	40	50
	507	37.19	37.03	36.90	116	4	FAN	EXERCISE2	40	50
	508	37.18	37.04	36.89	118	4	FAN	EXERCISE2	40	50
	509	37.18	37.04	36.89	121	4	FAN	EXERCISE2	40	50
	510	37.20	37.04	36.90	122	4	FAN	EXERCISE2	40	50
85	511	37.21	37.05	36.91	118	4	FAN	EXERCISE2	40	50
	512	37.22	37.06	36.91	119	4	FAN	EXERCISE2	40	50
	513	37.23	37.05	36.90	121	4	FAN	EXERCISE2	40	50
	514	37.23	37.07	36.89	123	4	FAN	EXERCISE2	40	50
	515	37.23	37.08	36.90	126	4	FAN	EXERCISE2	40	50
	516	37.23	37.06	36.90	124	4	FAN	EXERCISE2	40	50
	517	37.23	37.06	36.90	122	4	FAN	EXERCISE2	40	50
	518	37.23	37.07	36.92	122	4	FAN	EXERCISE2	40	50
	519	37.24	37.08	36.93	123	4	FAN	EXERCISE2	40	50
	520	37.25	37.09	36.94	122	4	FAN	EXERCISE2	40	50
	521	37.25	37.08	36.95	123	4	FAN	EXERCISE2	40	50

min	number	Tre	Tes	Tsk-head	HR	ID	condition	period	Ta	RH
	522	37.25	37.10	36.94	121	4	FAN	EXERCISE2	40	50
	523	37.25	37.11	36.93	120	4	FAN	EXERCISE2	40	50
	524	37.26	37.11	36.94	122	4	FAN	EXERCISE2	40	50
	525	37.27	37.12	36.94	124	4	FAN	EXERCISE2	40	50
	526	37.28	37.13	36.94	123	4	FAN	EXERCISE2	40	50
	527	37.28	37.13	36.94	123	4	FAN	EXERCISE2	40	50
	528	37.28	37.13	36.95	123	4	FAN	EXERCISE2	40	50
	529	37.28	37.14	36.95	124	4	FAN	EXERCISE2	40	50
	530	37.27	37.14	36.96	124	4	FAN	EXERCISE2	40	50
	531	37.28	37.16	36.97	125	4	FAN	EXERCISE2	40	50
	532	37.29	37.15	36.97	126	4	FAN	EXERCISE2	40	50
	533	37.30	37.14	36.97	124	4	FAN	EXERCISE2	40	50
	534	37.31	37.12	36.98	124	4	FAN	EXERCISE2	40	50
	535	37.32	37.12	36.99	122	4	FAN	EXERCISE2	40	50
	536	37.32	37.15	37.00	123	4	FAN	EXERCISE2	40	50
	537	37.32	37.16	37.00	125	4	FAN	EXERCISE2	40	50
	538	37.32	37.16	37.00	124	4	FAN	EXERCISE2	40	50
	539	37.33	37.16	37.01	121	4	FAN	EXERCISE2	40	50
	540	37.33	37.17	37.01	125	4	FAN	EXERCISE2	40	50
90	541	37.34	37.18	37.02	127	4	FAN	EXERCISE2	40	50
	542	37.35	37.18	37.03	125	4	FAN	EXERCISE2	40	50
	543	37.35	37.20	37.03	125	4	FAN	EXERCISE2	40	50
	544	37.36	37.24	37.04	128	4	FAN	EXERCISE2	40	50
	545	37.36	37.23	37.03	126	4	FAN	EXERCISE2	40	50
	546	37.36	37.23	37.03	124	4	FAN	EXERCISE2	40	50
	547	37.37	37.23	37.04	126	4	FAN	EXERCISE2	40	50
	548	37.38	37.23	37.04	126	4	FAN	EXERCISE2	40	50
	549	37.38	37.25	37.05	126	4	FAN	EXERCISE2	40	50
	550	37.38	37.25	37.05	128	4	FAN	EXERCISE2	40	50
	551	37.38	37.25	37.05	127	4	FAN	EXERCISE2	40	50
	552	37.38	37.25	37.05	127	4	FAN	EXERCISE2	40	50
	553	37.40	37.25	37.06	128	4	FAN	EXERCISE2	40	50
	554	37.41	37.25	37.06	127	4	FAN	EXERCISE2	40	50
	555	37.41	37.25	37.05	124	4	FAN	EXERCISE2	40	50
	556	37.41	37.27	37.05	124	4	FAN	EXERCISE2	40	50
	557	37.41	37.27	37.06	127	4	FAN	EXERCISE2	40	50
	558	37.42	37.27	37.06	130	4	FAN	EXERCISE2	40	50
	559	37.42	37.28	37.05	128	4	FAN	EXERCISE2	40	50
	560	37.42	37.29	37.05	128	4	FAN	EXERCISE2	40	50
	561	37.42	37.25	37.06	132	4	FAN	EXERCISE2	40	50
	562	37.43	37.22	37.05	129	4	FAN	EXERCISE2	40	50
	563	37.44	37.29	37.05	127	4	FAN	EXERCISE2	40	50
	564	37.44	37.30	37.05	130	4	FAN	EXERCISE2	40	50
	565	37.45	37.29	37.04	127	4	FAN	EXERCISE2	40	50
	566	37.46	37.28	37.04	128	4	FAN	EXERCISE2	40	50
	567	37.46	37.31	37.03	131	4	FAN	EXERCISE2	40	50

min	number	Tre	Tes	Tsk-head	HR	ID	condition	period	Ta	RH
	568	37.46	37.32	37.01	129	4	FAN	EXERCISE2	40	50
	569	37.47	37.31	36.96	132	4	FAN	EXERCISE2	40	50
	570	37.48	37.33	36.90	128	4	FAN	EXERCISE2	40	50
95	571	37.47	37.32	36.87	127	4	FAN	EXERCISE2	40	50
	572	37.47	37.33	36.86	127	4	FAN	EXERCISE2	40	50
	573	37.48	37.36	36.86	126	4	FAN	EXERCISE2	40	50
	574	37.49	37.36	36.85	126	4	FAN	EXERCISE2	40	50
	575	37.48	37.33	36.84	127	4	FAN	EXERCISE2	40	50
	576	37.48	37.34	36.83	124	4	FAN	EXERCISE2	40	50
	577	37.49	37.35	36.82	125	4	FAN	EXERCISE2	40	50
	578	37.50	37.33	36.81	127	4	FAN	EXERCISE2	40	50
	579	37.51	37.31	36.80	129	4	FAN	EXERCISE2	40	50
	580	37.50	37.30	36.80	132	4	FAN	EXERCISE2	40	50
	581	37.50	37.31	36.80	130	4	FAN	EXERCISE2	40	50
	582	37.51	37.37	36.80	128	4	FAN	EXERCISE2	40	50
	583	37.51	37.41	36.79	126	4	FAN	EXERCISE2	40	50
	584	37.52	37.42	36.74	126	4	FAN	EXERCISE2	40	50
	585	37.52	37.42	36.70	126	4	FAN	EXERCISE2	40	50
	586	37.51	37.40	36.71	126	4	FAN	EXERCISE2	40	50
	587	37.52	37.38	36.72	127	4	FAN	EXERCISE2	40	50
	588	37.52	37.36	36.71	131	4	FAN	EXERCISE2	40	50
	589	37.52	37.35	36.71	128	4	FAN	EXERCISE2	40	50
	590	37.51	37.37	36.68	129	4	FAN	EXERCISE2	40	50
	591	37.52	37.40	36.65	130	4	FAN	EXERCISE2	40	50
	592	37.52	37.39	36.63	129	4	FAN	EXERCISE2	40	50
	593	37.53	37.39	36.63	129	4	FAN	EXERCISE2	40	50
	594	37.54	37.39	36.66	129	4	FAN	EXERCISE2	40	50
	595	37.55	37.39	36.68	134	4	FAN	EXERCISE2	40	50
	596	37.56	37.40	36.69	132	4	FAN	EXERCISE2	40	50
	597	37.56	37.39	36.69	130	4	FAN	EXERCISE2	40	50
	598	37.55	37.39	36.69	132	4	FAN	EXERCISE2	40	50
	599	37.55	37.39	36.71	131	4	FAN	EXERCISE2	40	50
	600	37.56	37.36	36.65	134	4	FAN	EXERCISE2	40	50
100	601	37.57	37.37	36.50	132	4	FAN	EXERCISE2	40	50
	602	37.58	37.41	36.43	131	4	FAN	EXERCISE2	40	50
	603	37.59	37.41	36.46	132	4	FAN	EXERCISE2	40	50
	604	37.58	37.41	36.48	132	4	FAN	EXERCISE2	40	50
	605	37.58	37.43	36.50	130	4	FAN	EXERCISE2	40	50
	606	37.59	37.45	36.55	131	4	FAN	EXERCISE2	40	50
	607	37.58	37.44	36.59	129	4	FAN	EXERCISE2	40	50
	608	37.58	37.42	36.62	131	4	FAN	EXERCISE2	40	50
	609	37.58	37.44	36.63	132	4	FAN	EXERCISE2	40	50
	610	37.59	37.45	36.64	132	4	FAN	EXERCISE2	40	50
	611	37.60	37.44	36.62	132	4	FAN	EXERCISE2	40	50
	612	37.59	37.43	36.60	131	4	FAN	EXERCISE2	40	50
	613	37.59	37.45	36.60	131	4	FAN	EXERCISE2	40	50

min	number	Tre	Tes	Tsk-head	HR	ID	condition	period	Ta	RH
	614	37.60	37.46	36.61	133	4	FAN	EXERCISE2	40	50
	615	37.59	37.45	36.58	132	4	FAN	EXERCISE2	40	50
	616	37.59	37.44	36.56	132	4	FAN	EXERCISE2	40	50
	617	37.60	37.45	36.58	133	4	FAN	EXERCISE2	40	50
	618	37.60	37.47	36.61	133	4	FAN	EXERCISE2	40	50
	619	37.60	37.47	36.62	133	4	FAN	EXERCISE2	40	50
	620	37.60	37.47	36.65	134	4	FAN	EXERCISE2	40	50
	621	37.61	37.47	36.69	135	4	FAN	EXERCISE2	40	50
	622	37.61	37.47	36.71	134	4	FAN	EXERCISE2	40	50
	623	37.61	37.47	36.72	136	4	FAN	EXERCISE2	40	50
	624	37.61	37.47	36.72	136	4	FAN	EXERCISE2	40	50
	625	37.62	37.48	36.74	136	4	FAN	EXERCISE2	40	50
	626	37.63	37.51	36.75	139	4	FAN	EXERCISE2	40	50
	627	37.63	37.52	36.70	136	4	FAN	EXERCISE2	40	50
	628	37.64	37.52	36.63	136	4	FAN	EXERCISE2	40	50
	629	37.63	37.50	36.60	135	4	FAN	EXERCISE2	40	50
	630	37.64	37.50	36.62	136	4	FAN	EXERCISE2	40	50
105	631	37.64	37.51	36.63	138	4	FAN	EXERCISE2	40	50
	632	37.64	37.53	36.67	138	4	FAN	EXERCISE2	40	50
	633	37.65	37.54	36.72	134	4	FAN	EXERCISE2	40	50
	634	37.65	37.53	36.75	133	4	FAN	EXERCISE2	40	50
	635	37.65	37.52	36.76	135	4	FAN	EXERCISE2	40	50
	636	37.65	37.52	36.77	136	4	FAN	EXERCISE2	40	50
	637	37.65	37.53	36.77	134	4	FAN	EXERCISE2	40	50
	638	37.66	37.54	36.77	133	4	FAN	EXERCISE2	40	50
	639	37.65	37.54	36.75	133	4	FAN	EXERCISE2	40	50
	640	37.66	37.54	36.73	133	4	FAN	EXERCISE2	40	50
	641	37.67	37.53	36.72	132	4	FAN	EXERCISE2	40	50
	642	37.67	37.52	36.68	131	4	FAN	EXERCISE2	40	50
	643	37.67	37.52	36.66	133	4	FAN	EXERCISE2	40	50
	644	37.67	37.58	36.69	129	4	FAN	REST3	28	50
	645	37.67	37.63	36.50	130	4	FAN	REST3	28	50
	646	37.67	37.65	36.08	122	4	FAN	REST3	28	50
	647	37.68	37.68	35.89	121	4	FAN	REST3	28	50
	648	37.68	37.70	35.93	117	4	FAN	REST3	28	50
	649	37.68	37.73	35.90	117	4	FAN	REST3	28	50
	650	37.68	37.69	35.84	113	4	FAN	REST3	28	50
	651	37.69	37.67	35.81	110	4	FAN	REST3	28	50
	652	37.69	37.70	35.81	113	4	FAN	REST3	28	50
	653	37.69	37.70	35.75	117	4	FAN	REST3	28	50
	654	37.68	37.71	35.64	104	4	FAN	REST3	28	50
	655	37.69	37.71	35.62	102	4	FAN	REST3	28	50
	656	37.68	37.68	35.60	102	4	FAN	REST3	28	50
	657	37.67	37.56	35.52	104	4	FAN	REST3	28	50
	658	37.66	37.49	35.43	102	4	FAN	REST3	28	50
	659	37.64	37.54	35.45	99	4	FAN	REST3	28	50

min	number	Tre	Tes	Tsk-head	HR	ID	condition	period	Ta	RH
	660	37.64	37.55	35.42	99	4	FAN	REST3	28	50
110	661	37.62	37.54	35.28	99	4	FAN	REST3	28	50
	662	37.60	37.52	35.22	100	4	FAN	REST3	28	50
	663	37.58	37.51	35.18	98	4	FAN	REST3	28	50
	664	37.56	37.49	35.15	100	4	FAN	REST3	28	50
	665	37.55	37.48	35.22	99	4	FAN	REST3	28	50
	666	37.54	37.47	35.30	94	4	FAN	REST3	28	50
	667	37.52	37.43	35.30	96	4	FAN	REST3	28	50
	668	37.48	37.42	35.27	95	4	FAN	REST3	28	50
	669	37.45	37.40	35.23	92	4	FAN	REST3	28	50
	670	37.46	37.35	35.21	92	4	FAN	REST3	28	50
	671	37.48	37.36	35.22	92	4	FAN	REST3	28	50
	672	37.47	37.35	35.20	95	4	FAN	REST3	28	50
	673	37.47	37.31	35.16	93	4	FAN	REST3	28	50
	674	37.47	37.29	35.11	93	4	FAN	REST3	28	50
	675	37.47	37.29	35.05	91	4	FAN	REST3	28	50
	676	37.46	37.26	35.02	92	4	FAN	REST3	28	50
	677	37.44	37.24	34.98	91	4	FAN	REST3	28	50
	678	37.42	37.22	34.93	92	4	FAN	REST3	28	50
	679	37.40	37.21	34.91	91	4	FAN	REST3	28	50
	680	37.37	37.17	34.86	99	4	FAN	REST3	28	50
	681	37.35	37.14	34.74	98	4	FAN	REST3	28	50
	682	37.36	37.08	34.43	99	4	FAN	REST3	28	50
	683	37.36	37.05	34.19	96	4	FAN	REST3	28	50
	684	37.35	37.13	34.16	102	4	FAN	REST3	28	50
	685	37.33	37.07	34.07	92	4	FAN	REST3	28	50
	686	37.32	37.02	34.00	88	4	FAN	REST3	28	50
	687	37.33	37.07	33.89	90	4	FAN	REST3	28	50
	688	37.34	37.10	33.75	90	4	FAN	REST3	28	50
	689	37.35	37.12	33.77	97	4	FAN	REST3	28	50
	690	37.34	37.11	33.72	89	4	FAN	REST3	28	50
115	691	37.34	37.11	33.56	92	4	FAN	REST3	28	50
	692	37.35	37.11	33.36	91	4	FAN	REST3	28	50
	693	37.36	37.11	33.35	94	4	FAN	REST3	28	50
	694	37.37	37.12	33.47	95	4	FAN	REST3	28	50
	695	37.37	37.11	33.49	95	4	FAN	REST3	28	50
	696	37.36	37.11	33.53	106	4	FAN	REST3	28	50
	697	37.35	37.12	33.49	97	4	FAN	REST3	28	50
	698	37.34	37.12	33.45	89	4	FAN	REST3	28	50
	699	37.34	37.14	33.48	100	4	FAN	REST3	28	50
	700	37.33	37.10	33.47	101	4	FAN	REST3	28	50
	701	37.33	37.06	33.51	91	4	FAN	REST3	28	50
	702	37.34	37.10	33.64	102	4	FAN	REST3	28	50
	703	37.35	37.13	33.81	92	4	FAN	REST3	28	50
	704	37.36	37.13	34.05	89	4	FAN	REST3	28	50
	705	37.37	37.12	34.30	90	4	FAN	REST3	28	50

min	number	Tre	Tes	Tsk-head	HR	ID	condition	period	Ta	RH
	706	37.38	37.14	34.50	91	4	FAN	REST3	28	50
	707	37.38	37.16	34.63	95	4	FAN	REST3	28	50
	708	37.39	37.18	34.71	95	4	FAN	REST3	28	50
	709	37.39	37.17	34.77	91	4	FAN	REST3	28	50
0	1	36.96	36.87	35.51	80	5	FAN	REST1	28	50
	2	36.97	36.86	35.50	80	5	FAN	REST1	28	50
	3	36.96	36.87	35.49	79	5	FAN	REST1	28	50
	4	36.96	36.88	35.48	80	5	FAN	REST1	28	50
	5	36.97	36.88	35.48	88	5	FAN	REST1	28	50
	6	36.98	36.89	35.48	84	5	FAN	REST1	28	50
	7	36.97	36.87	35.47	82	5	FAN	REST1	28	50
	8	36.96	36.87	35.47	89	5	FAN	REST1	28	50
	9	36.96	36.87	35.48	92	5	FAN	REST1	28	50
	10	36.97	36.87	35.48	98	5	FAN	REST1	28	50
	11	36.97	36.87	35.47	89	5	FAN	REST1	28	50
	12	36.97	36.87	35.47	87	5	FAN	REST1	28	50
	13	36.97	36.87	35.48	89	5	FAN	REST1	28	50
	14	36.97	36.88	35.49	86	5	FAN	REST1	28	50
	15	36.97	36.90	35.49	88	5	FAN	REST1	28	50
	16	36.97	36.89	35.47	87	5	FAN	REST1	28	50
	17	36.97	36.89	35.47	97	5	FAN	REST1	28	50
	18	36.97	36.87	35.47	89	5	FAN	REST1	28	50
	19	36.98	36.86	35.47	86	5	FAN	REST1	28	50
	20	36.98	36.87	35.47	86	5	FAN	REST1	28	50
	21	36.99	36.87	35.47	91	5	FAN	REST1	28	50
	22	36.99	36.89	35.46	87	5	FAN	REST1	28	50
	23	36.98	36.88	35.44	84	5	FAN	REST1	28	50
	24	36.98	36.88	35.44	87	5	FAN	REST1	28	50
	25	36.97	36.90	35.46	86	5	FAN	REST1	28	50
	26	36.98	36.89	35.46	87	5	FAN	REST1	28	50
	27	36.98	36.88	35.45	88	5	FAN	REST1	28	50
	28	36.98	36.89	35.47	92	5	FAN	REST1	28	50
	29	36.98	36.89	35.48	92	5	FAN	REST1	28	50
	30	36.98	36.88	35.46	93	5	FAN	REST1	28	50
5	31	36.98	36.88	35.46	92	5	FAN	REST1	28	50
	32	36.97	36.89	35.47	92	5	FAN	REST1	28	50
	33	36.98	36.89	35.45	100	5	FAN	REST1	28	50
	34	36.98	36.87	35.45	93	5	FAN	REST1	28	50
	35	36.98	36.87	35.47	88	5	FAN	REST1	28	50
	36	36.97	36.86	35.48	82	5	FAN	REST1	28	50
	37	36.97	36.86	35.48	83	5	FAN	REST1	28	50
	38	36.96	36.85	35.48	80	5	FAN	REST1	28	50
	39	36.96	36.85	35.48	82	5	FAN	REST1	28	50
	40	36.97	36.85	35.47	90	5	FAN	REST1	28	50
	41	36.96	36.85	35.46	83	5	FAN	REST1	28	50
	42	36.96	36.86	35.47	83	5	FAN	REST1	28	50

min	number	Tre	Tes	Tsk-head	HR	ID	condition	period	Ta	RH
	43	36.96	36.87	35.47	91	5	FAN	REST1	28	50
	44	36.96	36.86	35.46	86	5	FAN	REST1	28	50
	45	36.96	36.85	35.46	80	5	FAN	REST1	28	50
	46	36.96	36.86	35.47	78	5	FAN	REST1	28	50
	47	36.97	36.86	35.48	78	5	FAN	REST1	28	50
	48	36.96	36.83	35.47	76	5	FAN	REST1	28	50
	49	36.95	36.82	35.46	76	5	FAN	REST1	28	50
	50	36.95	36.84	35.47	80	5	FAN	REST1	28	50
	51	36.95	36.82	35.47	76	5	FAN	REST1	28	50
	52	36.95	36.81	35.46	74	5	FAN	REST1	28	50
	53	36.95	36.82	35.46	78	5	FAN	REST1	28	50
	54	36.95	36.82	35.47	77	5	FAN	REST1	28	50
	55	36.95	36.81	35.47	91	5	FAN	REST1	28	50
	56	36.95	36.81	35.48	90	5	FAN	REST1	28	50
	57	36.95	36.82	35.48	85	5	FAN	REST1	28	50
	58	36.95	36.81	35.46	81	5	FAN	REST1	28	50
	59	36.96	36.81	35.46	80	5	FAN	REST1	28	50
	60	36.96	36.82	35.47	90	5	FAN	REST1	28	50
10	61	36.96	36.82	35.48	85	5	FAN	REST1	28	50
	62	36.97	36.81	35.47	85	5	FAN	REST1	28	50
	63	36.97	36.81	35.47	90	5	FAN	REST1	28	50
	64	36.97	36.82	35.47	84	5	FAN	REST1	28	50
	65	36.97	36.80	35.46	87	5	FAN	REST1	28	50
	66	36.97	36.80	35.47	93	5	FAN	REST1	28	50
	67	36.97	36.83	35.49	86	5	FAN	REST1	28	50
	68	36.97	36.82	35.48	85	5	FAN	REST1	28	50
	69	36.97	36.81	35.47	81	5	FAN	REST1	28	50
	70	36.97	36.80	35.47	88	5	FAN	REST1	28	50
	71	36.96	36.81	35.46	84	5	FAN	REST1	28	50
	72	36.96	36.83	35.47	85	5	FAN	REST1	28	50
	73	36.96	36.83	35.47	86	5	FAN	REST1	28	50
	74	36.96	36.83	35.46	81	5	FAN	REST1	28	50
	75	36.97	36.85	35.47	83	5	FAN	REST1	28	50
	76	36.96	36.85	35.46	82	5	FAN	REST1	28	50
	77	36.95	36.85	35.45	84	5	FAN	REST1	28	50
	78	36.95	36.85	35.45	85	5	FAN	REST1	28	50
	79	36.96	36.85	35.46	84	5	FAN	REST1	28	50
	80	36.97	36.86	35.46	93	5	FAN	REST1	28	50
	81	36.97	36.87	35.47	85	5	FAN	REST1	28	50
	82	36.97	36.87	35.48	90	5	FAN	REST1	28	50
	83	36.95	36.88	35.47	83	5	FAN	REST1	28	50
	84	36.95	36.88	35.47	80	5	FAN	REST1	28	50
	85	36.95	36.88	35.47	81	5	FAN	REST1	28	50
	86	36.96	36.88	35.48	90	5	FAN	REST1	28	50
	87	36.96	36.88	35.49	90	5	FAN	REST1	28	50
	88	36.96	36.88	35.48	85	5	FAN	REST1	28	50

min	number	Tre	Tes	Tsk-head	HR	ID	condition	period	Ta	RH
	89	36.96	36.89	35.48	83	5	FAN	REST1	28	50
	90	36.97	36.89	35.48	78	5	FAN	REST1	28	50
15	91	36.96	36.88	35.47	83	5	FAN	REST1	28	50
	92	36.96	36.88	35.48	83	5	FAN	REST1	28	50
	93	36.95	36.89	35.49	81	5	FAN	REST1	28	50
	94	36.95	36.90	35.48	93	5	FAN	REST1	28	50
	95	36.95	36.92	35.48	99	5	FAN	REST1	28	50
	96	36.95	36.91	35.49	92	5	FAN	REST1	28	50
	97	36.94	36.90	35.51	92	5	FAN	REST1	28	50
	98	36.95	36.90	35.52	94	5	FAN	REST1	28	50
	99	36.94	36.88	35.51	94	5	FAN	REST1	28	50
	100	36.94	36.87	35.53	94	5	FAN	REST1	28	50
	101	36.94	36.85	35.55	94	5	FAN	REST1	28	50
	102	36.94	36.86	35.57	91	5	FAN	REST1	28	50
	103	36.94	36.86	35.62	96	5	FAN	REST1	40	50
	104	36.95	36.87	35.85	112	5	FAN	REST1	40	50
	105	36.96	36.88	36.11	94	5	FAN	REST1	40	50
	106	36.95	36.87	36.22	92	5	FAN	REST1	40	50
	107	36.94	36.87	36.28	93	5	FAN	REST1	40	50
	108	36.94	36.85	36.34	89	5	FAN	REST1	40	50
	109	36.95	36.85	36.40	90	5	FAN	REST1	40	50
	110	36.96	36.84	36.47	109	5	FAN	REST1	40	50
	111	36.96	36.83	36.52	101	5	FAN	REST1	40	50
	112	36.96	36.84	36.55	96	5	FAN	REST1	40	50
	113	36.96	36.84	36.58	96	5	FAN	REST1	40	50
	114	36.96	36.86	36.61	93	5	FAN	REST1	40	50
	115	36.96	36.85	36.64	91	5	FAN	REST1	40	50
	116	36.95	36.83	36.66	95	5	FAN	REST1	40	50
	117	36.95	36.83	36.68	94	5	FAN	REST1	40	50
	118	36.96	36.84	36.70	98	5	FAN	REST1	40	50
	119	36.95	36.83	36.71	94	5	FAN	REST1	40	50
	120	36.96	36.84	36.72	107	5	FAN	REST1	40	50
20	121	36.96	36.84	36.74	94	5	FAN	REST1	40	50
	122	36.96	36.83	36.76	93	5	FAN	REST1	40	50
	123	36.96	36.85	36.77	97	5	FAN	REST1	40	50
	124	36.96	36.86	36.77	93	5	FAN	REST1	40	50
	125	36.95	36.84	36.78	90	5	FAN	REST1	40	50
	126	36.96	36.85	36.79	92	5	FAN	REST1	40	50
	127	36.97	36.86	36.81	94	5	FAN	REST1	40	50
	128	36.96	36.84	36.82	95	5	FAN	REST1	40	50
	129	36.96	36.86	36.84	93	5	FAN	REST1	40	50
	130	36.95	36.85	36.86	91	5	FAN	REST1	40	50
	131	36.95	36.84	36.87	91	5	FAN	REST1	40	50
	132	36.95	36.86	36.88	89	5	FAN	REST1	40	50
	133	36.96	36.86	36.87	94	5	FAN	REST1	40	50
	134	36.97	36.85	36.88	108	5	FAN	REST1	40	50

min	number	Tre	Tes	Tsk-head	HR	ID	condition	period	Ta	RH
	135	37.04	36.86	36.90	114	5	FAN	REST1	40	50
	136	37.03	36.86	36.90	103	5	FAN	REST1	40	50
	137	36.95	36.85	36.90	104	5	FAN	REST1	40	50
	138	36.95	36.86	36.90	101	5	FAN	REST1	40	50
	139	36.95	36.89	36.91	109	5	FAN	EXERCISE1	40	50
	140	36.96	36.90	36.93	116	5	FAN	EXERCISE1	40	50
	141	36.96	36.87	36.93	113	5	FAN	EXERCISE1	40	50
	142	36.96	36.83	36.92	117	5	FAN	EXERCISE1	40	50
	143	36.96	36.83	36.92	119	5	FAN	EXERCISE1	40	50
	144	36.95	36.85	36.92	121	5	FAN	EXERCISE1	40	50
	145	36.95	36.85	36.93	116	5	FAN	EXERCISE1	40	50
	146	36.95	36.86	36.93	117	5	FAN	EXERCISE1	40	50
	147	36.95	36.86	36.93	122	5	FAN	EXERCISE1	40	50
	148	36.95	36.84	36.93	121	5	FAN	EXERCISE1	40	50
	149	36.95	36.83	36.94	119	5	FAN	EXERCISE1	40	50
	150	36.95	36.83	36.94	114	5	FAN	EXERCISE1	40	50
25	151	36.95	36.83	36.95	118	5	FAN	EXERCISE1	40	50
	152	36.96	36.84	36.95	118	5	FAN	EXERCISE1	40	50
	153	36.96	36.87	36.95	115	5	FAN	EXERCISE1	40	50
	154	36.95	36.87	36.97	121	5	FAN	EXERCISE1	40	50
	155	36.95	36.85	36.98	123	5	FAN	EXERCISE1	40	50
	156	36.95	36.85	36.98	118	5	FAN	EXERCISE1	40	50
	157	36.95	36.86	36.99	120	5	FAN	EXERCISE1	40	50
	158	36.96	36.88	37.01	123	5	FAN	EXERCISE1	40	50
	159	36.96	36.90	37.03	123	5	FAN	EXERCISE1	40	50
	160	36.96	36.89	37.03	122	5	FAN	EXERCISE1	40	50
	161	36.96	36.88	37.03	125	5	FAN	EXERCISE1	40	50
	162	36.97	36.91	37.02	123	5	FAN	EXERCISE1	40	50
	163	36.97	36.91	37.02	125	5	FAN	EXERCISE1	40	50
	164	36.96	36.92	37.03	125	5	FAN	EXERCISE1	40	50
	165	36.96	36.93	37.05	123	5	FAN	EXERCISE1	40	50
	166	36.97	36.94	37.05	120	5	FAN	EXERCISE1	40	50
	167	36.97	36.95	37.07	122	5	FAN	EXERCISE1	40	50
	168	36.96	36.95	37.07	122	5	FAN	EXERCISE1	40	50
	169	36.97	36.96	37.07	121	5	FAN	EXERCISE1	40	50
	170	36.97	36.96	37.06	121	5	FAN	EXERCISE1	40	50
	171	36.96	36.98	37.05	122	5	FAN	EXERCISE1	40	50
	172	36.96	36.99	37.05	123	5	FAN	EXERCISE1	40	50
	173	36.96	36.99	37.05	124	5	FAN	EXERCISE1	40	50
	174	36.96	37.00	37.05	125	5	FAN	EXERCISE1	40	50
	175	36.96	37.02	37.05	125	5	FAN	EXERCISE1	40	50
	176	36.96	37.04	37.06	126	5	FAN	EXERCISE1	40	50
	177	36.96	37.04	37.07	123	5	FAN	EXERCISE1	40	50
	178	36.96	37.03	37.07	126	5	FAN	EXERCISE1	40	50
	179	36.97	37.06	37.09	123	5	FAN	EXERCISE1	40	50
	180	36.97	37.06	37.10	117	5	FAN	EXERCISE1	40	50

min	number	Tre	Tes	Tsk-head	HR	ID	condition	period	Ta	RH
30	181	36.96	37.05	37.07	123	5	FAN	EXERCISE1	40	50
	182	36.97	37.07	37.06	122	5	FAN	EXERCISE1	40	50
	183	36.97	37.06	37.08	119	5	FAN	EXERCISE1	40	50
	184	36.97	37.04	37.09	122	5	FAN	EXERCISE1	40	50
	185	36.99	37.09	37.09	122	5	FAN	EXERCISE1	40	50
	186	36.99	37.10	37.08	122	5	FAN	EXERCISE1	40	50
	187	36.99	37.11	37.08	121	5	FAN	EXERCISE1	40	50
	188	37.00	37.14	37.10	122	5	FAN	EXERCISE1	40	50
	189	37.00	37.14	37.10	124	5	FAN	EXERCISE1	40	50
	190	37.01	37.13	37.08	125	5	FAN	EXERCISE1	40	50
	191	37.01	37.14	37.09	126	5	FAN	EXERCISE1	40	50
	192	37.00	37.16	37.11	125	5	FAN	EXERCISE1	40	50
	193	37.01	37.12	37.10	129	5	FAN	EXERCISE1	40	50
	194	37.01	37.10	37.10	126	5	FAN	EXERCISE1	40	50
	195	37.01	37.16	37.11	128	5	FAN	EXERCISE1	40	50
	196	37.02	37.20	37.12	125	5	FAN	EXERCISE1	40	50
	197	37.02	37.19	37.12	125	5	FAN	EXERCISE1	40	50
	198	37.02	37.19	37.13	126	5	FAN	EXERCISE1	40	50
	199	37.02	37.20	37.14	125	5	FAN	EXERCISE1	40	50
	200	37.03	37.21	37.11	122	5	FAN	EXERCISE1	40	50
	201	37.03	37.21	37.10	126	5	FAN	EXERCISE1	40	50
	202	37.04	37.23	37.12	127	5	FAN	EXERCISE1	40	50
	203	37.04	37.25	37.12	122	5	FAN	EXERCISE1	40	50
	204	37.04	37.24	37.12	123	5	FAN	EXERCISE1	40	50
	205	37.04	37.24	37.14	124	5	FAN	EXERCISE1	40	50
	206	37.05	37.25	37.14	124	5	FAN	EXERCISE1	40	50
	207	37.05	37.27	37.13	125	5	FAN	EXERCISE1	40	50
	208	37.05	37.28	37.13	126	5	FAN	EXERCISE1	40	50
	209	37.05	37.27	37.12	128	5	FAN	EXERCISE1	40	50
	210	37.06	37.27	37.11	128	5	FAN	EXERCISE1	40	50
35	211	37.07	37.29	37.13	128	5	FAN	EXERCISE1	40	50
	212	37.07	37.30	37.12	131	5	FAN	EXERCISE1	40	50
	213	37.07	37.29	37.11	133	5	FAN	EXERCISE1	40	50
	214	37.07	37.28	37.13	131	5	FAN	EXERCISE1	40	50
	215	37.07	37.27	37.14	127	5	FAN	EXERCISE1	40	50
	216	37.08	37.28	37.14	127	5	FAN	EXERCISE1	40	50
	217	37.09	37.28	37.14	126	5	FAN	EXERCISE1	40	50
	218	37.09	37.29	37.14	126	5	FAN	EXERCISE1	40	50
	219	37.09	37.30	37.14	126	5	FAN	EXERCISE1	40	50
	220	37.10	37.30	37.13	127	5	FAN	EXERCISE1	40	50
	221	37.10	37.31	37.13	129	5	FAN	EXERCISE1	40	50
	222	37.10	37.32	37.13	126	5	FAN	EXERCISE1	40	50
	223	37.10	37.33	37.14	126	5	FAN	EXERCISE1	40	50
	224	37.11	37.32	37.14	129	5	FAN	EXERCISE1	40	50
	225	37.11	37.34	37.13	131	5	FAN	EXERCISE1	40	50
	226	37.12	37.34	37.13	131	5	FAN	EXERCISE1	40	50

min	number	Tre	Tes	Tsk-head	HR	ID	condition	period	Ta	RH
	227	37.12	37.33	37.14	131	5	FAN	EXERCISE1	40	50
	228	37.11	37.35	37.14	131	5	FAN	EXERCISE1	40	50
	229	37.11	37.34	37.13	131	5	FAN	EXERCISE1	40	50
	230	37.13	37.35	37.13	134	5	FAN	EXERCISE1	40	50
	231	37.14	37.35	37.13	133	5	FAN	EXERCISE1	40	50
	232	37.13	37.36	37.13	131	5	FAN	EXERCISE1	40	50
	233	37.13	37.37	37.15	132	5	FAN	EXERCISE1	40	50
	234	37.14	37.38	37.17	131	5	FAN	EXERCISE1	40	50
	235	37.14	37.39	37.18	131	5	FAN	EXERCISE1	40	50
	236	37.15	37.22	37.18	134	5	FAN	EXERCISE1	40	50
	237	37.15	37.15	37.17	134	5	FAN	EXERCISE1	40	50
	238	37.15	37.27	37.17	133	5	FAN	EXERCISE1	40	50
	239	37.15	37.28	37.17	130	5	FAN	EXERCISE1	40	50
	240	37.15	37.30	37.18	128	5	FAN	EXERCISE1	40	50
40	241	37.15	37.31	37.18	131	5	FAN	EXERCISE1	40	50
	242	37.16	37.32	37.20	132	5	FAN	EXERCISE1	40	50
	243	37.17	37.34	37.22	134	5	FAN	EXERCISE1	40	50
	244	37.17	37.35	37.23	132	5	FAN	EXERCISE1	40	50
	245	37.18	37.36	37.24	131	5	FAN	EXERCISE1	40	50
	246	37.18	37.37	37.23	131	5	FAN	EXERCISE1	40	50
	247	37.19	37.37	37.22	133	5	FAN	EXERCISE1	40	50
	248	37.19	37.37	37.23	134	5	FAN	EXERCISE1	40	50
	249	37.19	37.38	37.22	137	5	FAN	EXERCISE1	40	50
	250	37.20	37.39	37.22	138	5	FAN	EXERCISE1	40	50
	251	37.20	37.41	37.21	139	5	FAN	EXERCISE1	40	50
	252	37.21	37.37	37.20	140	5	FAN	EXERCISE1	40	50
	253	37.20	37.34	37.20	136	5	FAN	EXERCISE1	40	50
	254	37.20	37.36	37.21	137	5	FAN	EXERCISE1	40	50
	255	37.21	37.37	37.22	134	5	FAN	EXERCISE1	40	50
	256	37.22	37.37	37.22	133	5	FAN	EXERCISE1	40	50
	257	37.22	37.37	37.20	128	5	FAN	EXERCISE1	40	50
	258	37.22	37.38	37.20	132	5	FAN	EXERCISE1	40	50
	259	37.23	37.39	37.19	130	5	FAN	EXERCISE1	40	50
	260	37.24	37.41	37.19	132	5	FAN	EXERCISE1	40	50
	261	37.24	37.43	37.20	134	5	FAN	EXERCISE1	40	50
	262	37.24	37.44	37.20	136	5	FAN	EXERCISE1	40	50
	263	37.24	37.43	37.20	138	5	FAN	EXERCISE1	40	50
	264	37.24	37.42	37.19	137	5	FAN	EXERCISE1	40	50
	265	37.24	37.43	37.19	137	5	FAN	EXERCISE1	40	50
	266	37.25	37.45	37.21	137	5	FAN	EXERCISE1	40	50
	267	37.25	37.45	37.22	137	5	FAN	EXERCISE1	40	50
	268	37.25	37.44	37.23	139	5	FAN	EXERCISE1	40	50
	269	37.25	37.46	37.25	140	5	FAN	EXERCISE1	40	50
	270	37.26	37.46	37.26	142	5	FAN	EXERCISE1	40	50
45	271	37.26	37.45	37.26	143	5	FAN	EXERCISE1	40	50
	272	37.26	37.46	37.26	140	5	FAN	EXERCISE1	40	50

min	number	Tre	Tes	Tsk-head	HR	ID	condition	period	Ta	RH
	273	37.27	37.47	37.27	143	5	FAN	EXERCISE1	40	50
	274	37.27	37.49	37.29	146	5	FAN	EXERCISE1	40	50
	275	37.28	37.49	37.30	146	5	FAN	EXERCISE1	40	50
	276	37.28	37.39	37.29	144	5	FAN	EXERCISE1	40	50
	277	37.28	37.38	37.30	140	5	FAN	EXERCISE1	40	50
	278	37.28	37.47	37.29	142	5	FAN	EXERCISE1	40	50
	279	37.28	37.48	37.28	145	5	FAN	EXERCISE1	40	50
	280	37.28	37.49	37.28	143	5	FAN	EXERCISE1	40	50
	281	37.28	37.43	37.28	143	5	FAN	EXERCISE1	40	50
	282	37.29	37.42	37.29	137	5	FAN	EXERCISE1	40	50
	283	37.30	37.48	37.29	137	5	FAN	EXERCISE1	40	50
	284	37.30	37.50	37.27	137	5	FAN	EXERCISE1	40	50
	285	37.30	37.51	37.25	137	5	FAN	EXERCISE1	40	50
	286	37.30	37.52	37.25	141	5	FAN	EXERCISE1	40	50
	287	37.30	37.52	37.25	141	5	FAN	EXERCISE1	40	50
	288	37.31	37.51	37.24	140	5	FAN	EXERCISE1	40	50
	289	37.32	37.49	37.24	138	5	FAN	EXERCISE1	40	50
	290	37.32	37.50	37.24	140	5	FAN	EXERCISE1	40	50
	291	37.32	37.52	37.23	144	5	FAN	EXERCISE1	40	50
	292	37.32	37.53	37.23	145	5	FAN	EXERCISE1	40	50
	293	37.32	37.51	37.23	146	5	FAN	EXERCISE1	40	50
	294	37.33	37.51	37.24	143	5	FAN	EXERCISE1	40	50
	295	37.33	37.52	37.24	144	5	FAN	EXERCISE1	40	50
	296	37.32	37.52	37.23	143	5	FAN	EXERCISE1	40	50
	297	37.32	37.53	37.24	144	5	FAN	EXERCISE1	40	50
	298	37.32	37.53	37.23	143	5	FAN	EXERCISE1	40	50
	299	37.33	37.48	37.22	147	5	FAN	EXERCISE1	40	50
	300	37.33	37.48	37.23	143	5	FAN	EXERCISE1	40	50
50	301	37.33	37.54	37.22	143	5	FAN	EXERCISE1	40	50
	302	37.33	37.54	37.21	142	5	FAN	EXERCISE1	40	50
	303	37.34	37.54	37.22	143	5	FAN	EXERCISE1	40	50
	304	37.34	37.56	37.25	141	5	FAN	EXERCISE1	40	50
	305	37.34	37.57	37.25	143	5	FAN	EXERCISE1	40	50
	306	37.34	37.52	37.23	144	5	FAN	EXERCISE1	40	50
	307	37.35	37.54	37.24	143	5	FAN	EXERCISE1	40	50
	308	37.36	37.57	37.26	143	5	FAN	EXERCISE1	40	50
	309	37.35	37.57	37.25	143	5	FAN	EXERCISE1	40	50
	310	37.36	37.58	37.26	143	5	FAN	EXERCISE1	40	50
	311	37.37	37.57	37.26	143	5	FAN	EXERCISE1	40	50
	312	37.37	37.56	37.25	145	5	FAN	EXERCISE1	40	50
	313	37.37	37.57	37.25	145	5	FAN	EXERCISE1	40	50
	314	37.38	37.58	37.25	146	5	FAN	EXERCISE1	40	50
	315	37.38	37.58	37.26	146	5	FAN	EXERCISE1	40	50
	316	37.39	37.59	37.27	140	5	FAN	EXERCISE1	40	50
	317	37.40	37.60	37.27	137	5	FAN	EXERCISE1	40	50
	318	37.40	37.59	37.27	135	5	FAN	EXERCISE1	40	50

min	number	Tre	Tes	Tsk-head	HR	ID	condition	period	Ta	RH
	319	37.40	37.59	37.28	138	5	FAN	EXERCISE1	40	50
	320	37.39	37.58	37.28	137	5	FAN	REST2	28	50
	321	37.39	37.58	37.27	138	5	FAN	REST2	28	50
	322	37.39	37.56	37.16	140	5	FAN	REST2	28	50
	323	37.39	37.52	36.96	127	5	FAN	REST2	28	50
	324	37.39	37.53	36.73	117	5	FAN	REST2	28	50
	325	37.39	37.57	36.44	123	5	FAN	REST2	28	50
	326	37.39	37.60	36.28	120	5	FAN	REST2	28	50
	327	37.39	37.45	36.30	119	5	FAN	REST2	28	50
	328	37.39	37.41	36.28	113	5	FAN	REST2	28	50
	329	37.40	37.37	36.24	116	5	FAN	REST2	28	50
	330	37.39	37.35	36.20	114	5	FAN	REST2	28	50
55	331	37.40	37.52	36.17	112	5	FAN	REST2	28	50
	332	37.41	37.56	35.96	111	5	FAN	REST2	28	50
	333	37.41	37.58	35.80	108	5	FAN	REST2	28	50
	334	37.41	37.57	35.86	108	5	FAN	REST2	28	50
	335	37.42	37.56	35.87	105	5	FAN	REST2	28	50
	336	37.43	37.55	35.89	105	5	FAN	REST2	28	50
	337	37.42	37.55	35.90	103	5	FAN	REST2	28	50
	338	37.42	32.34	35.76	106	5	FAN	REST2	28	50
	339	37.43	27.08	35.73	120	5	FAN	REST2	28	50
	340	37.43	25.97	35.83	128	5	FAN	REST2	28	50
	341	37.43	24.98	35.82	124	5	FAN	REST2	28	50
	342	37.44	24.94	35.82	121	5	FAN	REST2	28	50
	343	37.43	26.25	35.84	116	5	FAN	REST2	28	50
	344	37.43	28.96	35.82	115	5	FAN	REST2	28	50
	345	37.43	30.49	35.78	107	5	FAN	REST2	28	50
	346	37.43	31.12	35.74	106	5	FAN	REST2	28	50
	347	37.43	31.93	35.71	107	5	FAN	REST2	28	50
	348	37.43	32.89	35.70	111	5	FAN	REST2	28	50
	349	37.42	32.51	35.65	109	5	FAN	REST2	28	50
	350	37.42	32.07	35.60	111	5	FAN	REST2	28	50
	351	37.43	33.65	35.62	104	5	FAN	REST2	28	50
	352	37.44	34.96	35.64	110	5	FAN	REST2	28	50
	353	37.43	35.36	35.68	106	5	FAN	REST2	28	50
	354	37.43	35.64	35.70	105	5	FAN	REST2	28	50
	355	37.44	35.79	35.70	109	5	FAN	REST2	28	50
	356	37.43	35.91	35.69	106	5	FAN	REST2	28	50
	357	37.42	36.05	35.66	106	5	FAN	REST2	28	50
	358	37.42	36.16	35.62	104	5	FAN	REST2	28	50
	359	37.43	36.24	35.61	102	5	FAN	REST2	28	50
	360	37.43	36.33	35.62	100	5	FAN	REST2	28	50
60	361	37.43	36.40	35.62	101	5	FAN	REST2	28	50
	362	37.44	36.46	35.59	97	5	FAN	REST2	28	50
	363	37.43	36.51	35.57	98	5	FAN	REST2	28	50
	364	37.43	36.54	35.58	103	5	FAN	REST2	28	50

min	number	Tre	Tes	Tsk-head	HR	ID	condition	period	Ta	RH
	365	37.43	36.58	35.58	104	5	FAN	REST2	28	50
	366	37.43	36.61	35.54	108	5	FAN	REST2	28	50
	367	37.44	36.65	35.50	99	5	FAN	REST2	28	50
	368	37.43	36.67	35.49	95	5	FAN	REST2	28	50
	369	37.43	36.68	35.47	98	5	FAN	REST2	28	50
	370	37.43	36.69	35.46	96	5	FAN	REST2	28	50
	371	37.42	36.71	35.42	99	5	FAN	REST2	28	50
	372	37.41	36.72	35.39	100	5	FAN	REST2	28	50
	373	37.42	36.73	35.41	96	5	FAN	REST2	28	50
	374	37.42	36.76	35.42	92	5	FAN	REST2	28	50
	375	37.42	36.76	35.41	96	5	FAN	REST2	28	50
	376	37.42	36.77	35.39	95	5	FAN	REST2	28	50
	377	37.42	36.78	35.36	100	5	FAN	REST2	28	50
	378	37.42	36.78	35.35	98	5	FAN	REST2	28	50
	379	37.42	36.79	35.33	93	5	FAN	REST2	28	50
	380	37.41	36.78	35.32	103	5	FAN	REST2	28	50
	381	37.41	36.79	35.31	92	5	FAN	REST2	28	50
	382	37.41	36.81	35.29	93	5	FAN	REST2	28	50
	383	37.42	36.81	35.27	97	5	FAN	REST2	28	50
	384	37.43	36.80	35.27	101	5	FAN	REST2	28	50
	385	37.42	36.78	35.21	103	5	FAN	REST2	28	50
	386	37.41	36.78	35.09	92	5	FAN	REST2	28	50
	387	37.40	36.80	35.01	92	5	FAN	REST2	28	50
	388	37.41	36.81	34.97	91	5	FAN	REST2	28	50
	389	37.41	36.83	34.96	92	5	FAN	REST2	28	50
	390	37.41	36.85	34.98	94	5	FAN	REST2	28	50
65	391	37.41	36.86	35.03	96	5	FAN	REST2	28	50
	392	37.40	36.83	35.09	96	5	FAN	REST2	28	50
	393	37.39	36.85	35.11	95	5	FAN	REST2	28	50
	394	37.40	36.88	35.13	93	5	FAN	REST2	28	50
	395	37.40	36.87	35.16	93	5	FAN	REST2	28	50
	396	37.39	36.89	35.20	92	5	FAN	REST2	28	50
	397	37.39	36.90	35.23	89	5	FAN	REST2	28	50
	398	37.39	36.89	35.24	89	5	FAN	REST2	28	50
	399	37.39	36.89	35.27	90	5	FAN	REST2	28	50
	400	37.38	36.89	35.27	87	5	FAN	REST2	28	50
	401	37.38	36.91	35.28	96	5	FAN	REST2	28	50
	402	37.39	36.92	35.31	94	5	FAN	REST2	28	50
	403	37.39	36.92	35.32	89	5	FAN	REST2	28	50
	404	37.39	36.94	35.32	91	5	FAN	REST2	28	50
	405	37.39	36.95	35.33	99	5	FAN	REST2	28	50
	406	37.39	36.97	35.37	100	5	FAN	REST2	28	50
	407	37.38	36.97	35.42	96	5	FAN	REST2	28	50
	408	37.38	36.96	35.46	91	5	FAN	REST2	28	50
	409	37.38	36.97	35.47	95	5	FAN	REST2	28	50
	410	37.37	36.96	35.48	98	5	FAN	REST2	28	50

min	number	Tre	Tes	Tsk-head	HR	ID	condition	period	Ta	RH
	411	37.37	36.97	35.51	94	5	FAN	REST2	28	50
	412	37.37	36.98	35.50	91	5	FAN	REST2	28	50
	413	37.36	36.97	35.50	92	5	FAN	REST2	28	50
	414	37.36	36.98	35.52	91	5	FAN	REST2	28	50
	415	37.36	37.00	35.56	89	5	FAN	REST2	28	50
	416	37.36	37.00	35.57	92	5	FAN	REST2	28	50
	417	37.36	37.00	35.55	92	5	FAN	REST2	28	50
	418	37.36	37.00	35.55	92	5	FAN	REST2	28	50
	419	37.36	37.00	35.55	96	5	FAN	REST2	28	50
	420	37.36	37.00	35.54	99	5	FAN	REST2	28	50
70	421	37.35	37.00	35.55	103	5	FAN	REST2	28	50
	422	37.34	36.60	35.56	101	5	FAN	REST2	28	50
	423	37.34	36.16	35.54	102	5	FAN	REST2	28	50
	424	37.35	36.55	35.53	92	5	FAN	REST2	28	50
	425	37.34	36.98	35.56	90	5	FAN	REST2	28	50
	426	37.34	37.00	35.58	93	5	FAN	REST2	28	50
	427	37.35	37.03	35.59	90	5	FAN	REST2	28	50
	428	37.35	37.03	35.59	90	5	FAN	REST2	28	50
	429	37.35	37.03	35.59	90	5	FAN	REST2	28	50
	430	37.35	37.02	35.59	93	5	FAN	REST2	28	50
	431	37.36	37.03	35.60	94	5	FAN	REST2	28	50
	432	37.36	37.06	35.59	90	5	FAN	REST2	28	50
	433	37.36	37.08	35.58	90	5	FAN	REST2	28	50
	434	37.36	37.08	35.57	90	5	FAN	REST2	28	50
	435	37.36	37.07	35.57	87	5	FAN	REST2	28	50
	436	37.36	37.09	35.57	90	5	FAN	REST2	28	50
	437	37.36	37.10	35.56	89	5	FAN	REST2	28	50
	438	37.36	37.10	35.55	93	5	FAN	REST2	28	50
	439	37.36	37.11	35.59	93	5	FAN	REST2	40	50
	440	37.36	37.11	35.77	106	5	FAN	REST2	40	50
	441	37.36	37.13	35.99	113	5	FAN	REST2	40	50
	442	37.36	37.12	36.14	104	5	FAN	REST2	40	50
	443	37.36	37.11	36.24	104	5	FAN	REST2	40	50
	444	37.36	37.10	36.33	101	5	FAN	REST2	40	50
	445	37.37	37.10	36.40	104	5	FAN	REST2	40	50
	446	37.37	37.11	36.46	101	5	FAN	REST2	40	50
	447	37.37	37.13	36.53	98	5	FAN	REST2	40	50
	448	37.38	37.13	36.58	95	5	FAN	REST2	40	50
	449	37.38	37.11	36.59	95	5	FAN	REST2	40	50
	450	37.38	37.12	36.61	92	5	FAN	REST2	40	50
75	451	37.38	37.12	36.63	97	5	FAN	REST2	40	50
	452	37.38	37.11	36.64	105	5	FAN	REST2	40	50
	453	37.38	37.12	36.66	92	5	FAN	REST2	40	50
	454	37.37	37.11	36.68	101	5	FAN	REST2	40	50
	455	37.36	37.11	36.69	102	5	FAN	REST2	40	50
	456	37.36	37.12	36.71	102	5	FAN	REST2	40	50

min	number	Tre	Tes	Tsk-head	HR	ID	condition	period	Ta	RH
	457	37.37	37.13	36.71	108	5	FAN	REST2	40	50
	458	37.36	37.14	36.71	121	5	FAN	REST2	40	50
	459	37.36	37.13	36.71	124	5	FAN	REST2	40	50
	460	37.36	36.97	36.72	119	5	FAN	REST2	40	50
	461	37.36	36.61	36.75	113	5	FAN	REST2	40	50
	462	37.36	36.75	36.76	106	5	FAN	REST2	40	50
	463	37.36	37.09	36.78	110	5	FAN	EXERCISE2	40	50
	464	37.36	37.10	36.79	119	5	FAN	EXERCISE2	40	50
	465	37.36	37.11	36.80	121	5	FAN	EXERCISE2	40	50
	466	37.35	37.11	36.81	123	5	FAN	EXERCISE2	40	50
	467	37.35	37.12	36.82	124	5	FAN	EXERCISE2	40	50
	468	37.36	37.14	36.84	122	5	FAN	EXERCISE2	40	50
	469	37.36	37.15	36.85	118	5	FAN	EXERCISE2	40	50
	470	37.35	37.14	36.83	122	5	FAN	EXERCISE2	40	50
	471	37.35	37.15	36.84	129	5	FAN	EXERCISE2	40	50
	472	37.36	37.16	36.86	134	5	FAN	EXERCISE2	40	50
	473	37.36	37.17	36.87	136	5	FAN	EXERCISE2	40	50
	474	37.35	37.18	36.87	134	5	FAN	EXERCISE2	40	50
	475	37.36	37.17	36.89	128	5	FAN	EXERCISE2	40	50
	476	37.36	37.19	36.91	127	5	FAN	EXERCISE2	40	50
	477	37.36	37.22	36.93	123	5	FAN	EXERCISE2	40	50
	478	37.35	37.23	36.94	124	5	FAN	EXERCISE2	40	50
	479	37.35	37.22	36.95	124	5	FAN	EXERCISE2	40	50
	480	37.36	37.22	36.96	125	5	FAN	EXERCISE2	40	50
80	481	37.36	37.23	36.98	128	5	FAN	EXERCISE2	40	50
	482	37.36	37.24	36.97	127	5	FAN	EXERCISE2	40	50
	483	37.36	37.25	36.98	126	5	FAN	EXERCISE2	40	50
	484	37.37	37.27	37.00	125	5	FAN	EXERCISE2	40	50
	485	37.37	37.30	37.01	126	5	FAN	EXERCISE2	40	50
	486	37.36	37.30	37.02	126	5	FAN	EXERCISE2	40	50
	487	37.36	37.30	37.05	126	5	FAN	EXERCISE2	40	50
	488	37.36	37.30	37.06	128	5	FAN	EXERCISE2	40	50
	489	37.37	37.31	37.07	129	5	FAN	EXERCISE2	40	50
	490	37.37	37.27	37.08	126	5	FAN	EXERCISE2	40	50
	491	37.38	37.27	37.10	125	5	FAN	EXERCISE2	40	50
	492	37.38	37.32	37.15	128	5	FAN	EXERCISE2	40	50
	493	37.38	37.34	37.16	131	5	FAN	EXERCISE2	40	50
	494	37.38	37.35	37.14	125	5	FAN	EXERCISE2	40	50
	495	37.38	37.36	37.15	124	5	FAN	EXERCISE2	40	50
	496	37.39	37.37	37.17	123	5	FAN	EXERCISE2	40	50
	497	37.39	37.38	37.16	129	5	FAN	EXERCISE2	40	50
	498	37.39	37.39	37.13	131	5	FAN	EXERCISE2	40	50
	499	37.39	37.36	37.14	131	5	FAN	EXERCISE2	40	50
	500	37.40	37.28	37.15	136	5	FAN	EXERCISE2	40	50
	501	37.40	37.29	37.16	134	5	FAN	EXERCISE2	40	50
	502	37.39	37.34	37.16	134	5	FAN	EXERCISE2	40	50

min	number	Tre	Tes	Tsk-head	HR	ID	condition	period	Ta	RH
	503	37.40	37.36	37.15	135	5	FAN	EXERCISE2	40	50
	504	37.40	37.39	37.16	134	5	FAN	EXERCISE2	40	50
	505	37.40	37.41	37.17	136	5	FAN	EXERCISE2	40	50
	506	37.40	37.42	37.17	137	5	FAN	EXERCISE2	40	50
	507	37.41	37.43	37.16	143	5	FAN	EXERCISE2	40	50
	508	37.42	37.44	37.14	137	5	FAN	EXERCISE2	40	50
	509	37.42	37.44	37.14	136	5	FAN	EXERCISE2	40	50
	510	37.43	37.45	37.15	139	5	FAN	EXERCISE2	40	50
85	511	37.42	37.46	37.16	141	5	FAN	EXERCISE2	40	50
	512	37.42	37.47	37.17	137	5	FAN	EXERCISE2	40	50
	513	37.43	37.47	37.17	140	5	FAN	EXERCISE2	40	50
	514	37.43	37.48	37.17	141	5	FAN	EXERCISE2	40	50
	515	37.44	37.50	37.18	141	5	FAN	EXERCISE2	40	50
	516	37.45	37.49	37.18	140	5	FAN	EXERCISE2	40	50
	517	37.44	37.51	37.20	140	5	FAN	EXERCISE2	40	50
	518	37.44	37.52	37.21	138	5	FAN	EXERCISE2	40	50
	519	37.45	37.52	37.22	140	5	FAN	EXERCISE2	40	50
	520	37.45	37.53	37.22	140	5	FAN	EXERCISE2	40	50
	521	37.46	37.49	37.22	143	5	FAN	EXERCISE2	40	50
	522	37.46	37.47	37.23	143	5	FAN	EXERCISE2	40	50
	523	37.46	37.52	37.25	140	5	FAN	EXERCISE2	40	50
	524	37.46	37.56	37.26	137	5	FAN	EXERCISE2	40	50
	525	37.46	37.56	37.25	137	5	FAN	EXERCISE2	40	50
	526	37.46	37.55	37.24	140	5	FAN	EXERCISE2	40	50
	527	37.47	37.36	37.26	142	5	FAN	EXERCISE2	40	50
	528	37.47	37.34	37.27	141	5	FAN	EXERCISE2	40	50
	529	37.48	37.52	37.28	141	5	FAN	EXERCISE2	40	50
	530	37.48	37.54	37.30	142	5	FAN	EXERCISE2	40	50
	531	37.48	37.55	37.30	143	5	FAN	EXERCISE2	40	50
	532	37.48	37.55	37.30	146	5	FAN	EXERCISE2	40	50
	533	37.49	37.58	37.30	144	5	FAN	EXERCISE2	40	50
	534	37.49	37.60	37.32	145	5	FAN	EXERCISE2	40	50
	535	37.49	37.62	37.32	144	5	FAN	EXERCISE2	40	50
	536	37.50	37.62	37.32	146	5	FAN	EXERCISE2	40	50
	537	37.50	37.63	37.32	147	5	FAN	EXERCISE2	40	50
	538	37.50	37.64	37.32	150	5	FAN	EXERCISE2	40	50
	539	37.50	37.64	37.33	150	5	FAN	EXERCISE2	40	50
	540	37.51	37.64	37.33	149	5	FAN	EXERCISE2	40	50
90	541	37.51	37.63	37.30	149	5	FAN	EXERCISE2	40	50
	542	37.51	37.64	37.30	150	5	FAN	EXERCISE2	40	50
	543	37.52	37.66	37.32	148	5	FAN	EXERCISE2	40	50
	544	37.52	37.66	37.34	148	5	FAN	EXERCISE2	40	50
	545	37.53	37.65	37.35	147	5	FAN	EXERCISE2	40	50
	546	37.53	37.66	37.35	148	5	FAN	EXERCISE2	40	50
	547	37.53	37.68	37.35	147	5	FAN	EXERCISE2	40	50
	548	37.53	37.69	37.35	146	5	FAN	EXERCISE2	40	50

min	number	Tre	Tes	Tsk-head	HR	ID	condition	period	Ta	RH
	549	37.53	37.67	37.35	144	5	FAN	EXERCISE2	40	50
	550	37.53	37.68	37.36	147	5	FAN	EXERCISE2	40	50
	551	37.54	37.69	37.38	148	5	FAN	EXERCISE2	40	50
	552	37.54	37.68	37.39	151	5	FAN	EXERCISE2	40	50
	553	37.54	37.69	37.40	153	5	FAN	EXERCISE2	40	50
	554	37.54	37.70	37.41	146	5	FAN	EXERCISE2	40	50
	555	37.55	37.71	37.39	140	5	FAN	EXERCISE2	40	50
	556	37.55	37.71	37.38	139	5	FAN	EXERCISE2	40	50
	557	37.55	37.70	37.37	143	5	FAN	EXERCISE2	40	50
	558	37.55	37.70	37.38	146	5	FAN	EXERCISE2	40	50
	559	37.55	37.70	37.39	146	5	FAN	EXERCISE2	40	50
	560	37.55	37.63	37.40	150	5	FAN	EXERCISE2	40	50
	561	37.55	37.63	37.40	146	5	FAN	EXERCISE2	40	50
	562	37.56	37.68	37.40	146	5	FAN	EXERCISE2	40	50
	563	37.57	37.70	37.41	146	5	FAN	EXERCISE2	40	50
	564	37.57	37.72	37.42	146	5	FAN	EXERCISE2	40	50
	565	37.57	37.71	37.42	147	5	FAN	EXERCISE2	40	50
	566	37.57	37.72	37.42	148	5	FAN	EXERCISE2	40	50
	567	37.57	37.70	37.41	146	5	FAN	EXERCISE2	40	50
	568	37.56	37.70	37.40	143	5	FAN	EXERCISE2	40	50
	569	37.56	37.73	37.40	143	5	FAN	EXERCISE2	40	50
	570	37.57	37.74	37.42	146	5	FAN	EXERCISE2	40	50
95	571	37.58	37.53	37.42	150	5	FAN	EXERCISE2	40	50
	572	37.58	37.49	37.41	148	5	FAN	EXERCISE2	40	50
	573	37.58	37.66	37.41	146	5	FAN	EXERCISE2	40	50
	574	37.59	37.67	37.41	146	5	FAN	EXERCISE2	40	50
	575	37.59	37.67	37.40	144	5	FAN	EXERCISE2	40	50
	576	37.59	37.67	37.40	146	5	FAN	EXERCISE2	40	50
	577	37.60	37.68	37.41	145	5	FAN	EXERCISE2	40	50
	578	37.60	37.70	37.42	143	5	FAN	EXERCISE2	40	50
	579	37.60	37.71	37.43	145	5	FAN	EXERCISE2	40	50
	580	37.60	37.70	37.43	141	5	FAN	EXERCISE2	40	50
	581	37.60	37.71	37.42	141	5	FAN	EXERCISE2	40	50
	582	37.61	37.73	37.43	146	5	FAN	EXERCISE2	40	50
	583	37.61	37.72	37.44	146	5	FAN	EXERCISE2	40	50
	584	37.61	37.71	37.45	144	5	FAN	EXERCISE2	40	50
	585	37.61	37.72	37.44	143	5	FAN	EXERCISE2	40	50
	586	37.62	37.74	37.43	143	5	FAN	EXERCISE2	40	50
	587	37.62	37.75	37.44	143	5	FAN	EXERCISE2	40	50
	588	37.62	37.75	37.44	146	5	FAN	EXERCISE2	40	50
	589	37.62	37.75	37.43	150	5	FAN	EXERCISE2	40	50
	590	37.63	37.73	37.44	147	5	FAN	EXERCISE2	40	50
	591	37.64	37.72	37.44	146	5	FAN	EXERCISE2	40	50
	592	37.65	37.73	37.44	143	5	FAN	EXERCISE2	40	50
	593	37.65	37.74	37.45	143	5	FAN	EXERCISE2	40	50
	594	37.65	37.74	37.44	145	5	FAN	EXERCISE2	40	50

min	number	Tre	Tes	Tsk-head	HR	ID	condition	period	Ta	RH
	595	37.64	37.74	37.44	144	5	FAN	EXERCISE2	40	50
	596	37.65	37.75	37.46	145	5	FAN	EXERCISE2	40	50
	597	37.67	37.76	37.47	145	5	FAN	EXERCISE2	40	50
	598	37.66	37.76	37.43	146	5	FAN	EXERCISE2	40	50
	599	37.66	37.77	37.44	146	5	FAN	EXERCISE2	40	50
	600	37.66	37.76	37.47	144	5	FAN	EXERCISE2	40	50
100	601	37.66	37.77	37.47	143	5	FAN	EXERCISE2	40	50
	602	37.67	37.79	37.48	143	5	FAN	EXERCISE2	40	50
	603	37.68	37.80	37.49	143	5	FAN	EXERCISE2	40	50
	604	37.68	37.81	37.52	145	5	FAN	EXERCISE2	40	50
	605	37.68	37.80	37.51	144	5	FAN	EXERCISE2	40	50
	606	37.68	37.78	37.49	143	5	FAN	EXERCISE2	40	50
	607	37.69	37.78	37.51	146	5	FAN	EXERCISE2	40	50
	608	37.69	37.78	37.50	146	5	FAN	EXERCISE2	40	50
	609	37.70	37.79	37.48	147	5	FAN	EXERCISE2	40	50
	610	37.70	37.75	37.48	146	5	FAN	EXERCISE2	40	50
	611	37.70	37.73	37.49	143	5	FAN	EXERCISE2	40	50
	612	37.70	37.65	37.50	143	5	FAN	EXERCISE2	40	50
	613	37.70	37.61	37.50	148	5	FAN	EXERCISE2	40	50
	614	37.69	37.70	37.49	145	5	FAN	EXERCISE2	40	50
	615	37.70	37.71	37.49	147	5	FAN	EXERCISE2	40	50
	616	37.70	37.71	37.49	146	5	FAN	EXERCISE2	40	50
	617	37.71	37.73	37.50	152	5	FAN	EXERCISE2	40	50
	618	37.71	37.74	37.51	153	5	FAN	EXERCISE2	40	50
	619	37.70	37.76	37.51	149	5	FAN	EXERCISE2	40	50
	620	37.71	37.77	37.51	150	5	FAN	EXERCISE2	40	50
	621	37.72	37.77	37.52	151	5	FAN	EXERCISE2	40	50
	622	37.72	37.78	37.52	153	5	FAN	EXERCISE2	40	50
	623	37.72	37.79	37.53	154	5	FAN	EXERCISE2	40	50
	624	37.73	37.79	37.53	157	5	FAN	EXERCISE2	40	50
	625	37.74	37.80	37.54	157	5	FAN	EXERCISE2	40	50
	626	37.74	37.81	37.55	153	5	FAN	EXERCISE2	40	50
	627	37.74	37.83	37.56	153	5	FAN	EXERCISE2	40	50
	628	37.75	37.83	37.61	153	5	FAN	EXERCISE2	40	50
	629	37.75	37.82	37.61	153	5	FAN	EXERCISE2	40	50
	630	37.75	37.82	37.56	156	5	FAN	EXERCISE2	40	50
105	631	37.75	37.84	37.58	153	5	FAN	EXERCISE2	40	50
	632	37.75	37.83	37.57	153	5	FAN	EXERCISE2	40	50
	633	37.75	37.83	37.54	153	5	FAN	EXERCISE2	40	50
	634	37.75	37.85	37.56	153	5	FAN	EXERCISE2	40	50
	635	37.76	37.85	37.56	153	5	FAN	EXERCISE2	40	50
	636	37.76	37.85	37.56	149	5	FAN	EXERCISE2	40	50
	637	37.76	37.65	37.56	147	5	FAN	EXERCISE2	40	50
	638	37.77	37.60	37.55	150	5	FAN	EXERCISE2	40	50
	639	37.78	37.78	37.55	150	5	FAN	EXERCISE2	40	50
	640	37.78	37.81	37.55	154	5	FAN	EXERCISE2	40	50

min	number	Tre	Tes	Tsk-head	HR	ID	condition	period	Ta	RH
	641	37.78	37.81	37.54	155	5	FAN	EXERCISE2	40	50
	642	37.77	37.81	37.55	151	5	FAN	EXERCISE2	40	50
	643	37.77	37.82	37.57	146	5	FAN	EXERCISE2	40	50
	644	37.77	37.82	37.56	147	5	FAN	REST3	28	50
	645	37.78	37.85	37.40	150	5	FAN	REST3	28	50
	646	37.79	37.90	37.14	141	5	FAN	REST3	28	50
	647	37.78	37.91	36.95	139	5	FAN	REST3	28	50
	648	37.79	37.89	36.85	131	5	FAN	REST3	28	50
	649	37.80	37.88	36.74	125	5	FAN	REST3	28	50
	650	37.80	37.89	36.71	127	5	FAN	REST3	28	50
	651	37.80	37.88	36.73	127	5	FAN	REST3	28	50
	652	37.80	37.88	36.72	127	5	FAN	REST3	28	50
	653	37.80	37.90	36.69	126	5	FAN	REST3	28	50
	654	37.80	37.89	36.65	119	5	FAN	REST3	28	50
	655	37.80	37.89	36.60	121	5	FAN	REST3	28	50
	656	37.80	37.88	36.55	119	5	FAN	REST3	28	50
	657	37.81	37.86	36.51	118	5	FAN	REST3	28	50
	658	37.80	37.86	36.46	118	5	FAN	REST3	28	50
	659	37.81	37.84	36.43	118	5	FAN	REST3	28	50
	660	37.82	37.79	36.39	126	5	FAN	REST3	28	50
110	661	37.82	37.77	36.33	115	5	FAN	REST3	28	50
	662	37.81	37.79	36.29	113	5	FAN	REST3	28	50
	663	37.81	37.77	36.27	112	5	FAN	REST3	28	50
	664	37.82	37.78	36.23	113	5	FAN	REST3	28	50
	665	37.83	37.80	36.18	113	5	FAN	REST3	28	50
	666	37.84	37.79	36.18	112	5	FAN	REST3	28	50
	667	37.84	37.79	36.16	110	5	FAN	REST3	28	50
	668	37.84	37.77	36.09	110	5	FAN	REST3	28	50
	669	37.84	37.75	36.06	111	5	FAN	REST3	28	50
	670	37.84	37.74	36.05	106	5	FAN	REST3	28	50
	671	37.84	37.74	36.04	107	5	FAN	REST3	28	50
	672	37.84	37.74	36.01	104	5	FAN	REST3	28	50
	673	37.83	37.73	35.98	108	5	FAN	REST3	28	50
	674	37.84	37.71	35.95	107	5	FAN	REST3	28	50
	675	37.84	37.71	35.93	110	5	FAN	REST3	28	50
	676	37.85	37.71	35.92	110	5	FAN	REST3	28	50
	677	37.84	37.71	35.90	111	5	FAN	REST3	28	50
	678	37.84	37.63	35.89	117	5	FAN	REST3	28	50
	679	37.83	37.59	35.88	112	5	FAN	REST3	28	50
	680	37.83	37.64	35.86	109	5	FAN	REST3	28	50
	681	37.82	37.67	35.85	107	5	FAN	REST3	28	50
	682	37.82	37.68	35.84	110	5	FAN	REST3	28	50
	683	37.83	37.67	35.81	110	5	FAN	REST3	28	50
	684	37.83	37.65	35.80	116	5	FAN	REST3	28	50
	685	37.84	37.65	35.75	121	5	FAN	REST3	28	50
	686	37.83	37.60	35.69	114	5	FAN	REST3	28	50

min	number	Tre	Tes	Tsk-head	HR	ID	condition	period	Ta	RH
	687	37.83	37.60	35.67	107	5	FAN	REST3	28	50
	688	37.84	37.64	35.66	105	5	FAN	REST3	28	50
	689	37.84	37.64	35.65	109	5	FAN	REST3	28	50
	690	37.84	37.65	35.58	108	5	FAN	REST3	28	50
115	691	37.84	37.67	35.55	103	5	FAN	REST3	28	50
	692	37.85	37.67	35.57	101	5	FAN	REST3	28	50
	693	37.84	37.63	35.54	100	5	FAN	REST3	28	50
	694	37.82	37.64	35.49	109	5	FAN	REST3	28	50
	695	37.83	37.64	35.48	109	5	FAN	REST3	28	50
	696	37.84	37.62	35.47	108	5	FAN	REST3	28	50
	697	37.83	37.62	35.42	107	5	FAN	REST3	28	50
	698	37.83	37.64	35.36	105	5	FAN	REST3	28	50
	699	37.83	37.65	35.34	108	5	FAN	REST3	28	50
	700	37.83	37.65	35.32	103	5	FAN	REST3	28	50
	701	37.83	37.64	35.32	99	5	FAN	REST3	28	50
	702	37.84	37.63	35.35	102	5	FAN	REST3	28	50
	703	37.84	37.63	35.37	97	5	FAN	REST3	28	50
	704	37.84	37.63	35.39	105	5	FAN	REST3	28	50
	705	37.84	37.63	35.42	112	5	FAN	REST3	28	50
	706	37.84	37.64	35.42	98	5	FAN	REST3	28	50
	707	37.84	37.64	35.38	105	5	FAN	REST3	28	50
	708	37.85	37.65	35.36	96	5	FAN	REST3	28	50
	709	37.85	37.66	35.38	103	5	FAN	REST3	28	50
0	1	36.76	36.76	35.33	79	6	FAN	REST1	28	50
	2	36.76	36.76	35.32	81	6	FAN	REST1	28	50
	3	36.77	36.77	35.32	77	6	FAN	REST1	28	50
	4	36.78	36.78	35.31	79	6	FAN	REST1	28	50
	5	36.78	36.79	35.28	76	6	FAN	REST1	28	50
	6	36.78	36.77	35.29	76	6	FAN	REST1	28	50
	7	36.77	36.77	35.26	83	6	FAN	REST1	28	50
	8	36.77	36.78	35.24	85	6	FAN	REST1	28	50
	9	36.78	36.79	35.23	70	6	FAN	REST1	28	50
	10	36.78	36.78	35.24	76	6	FAN	REST1	28	50
	11	36.78	36.79	35.23	79	6	FAN	REST1	28	50
	12	36.79	36.80	35.24	77	6	FAN	REST1	28	50
	13	36.80	36.79	35.25	79	6	FAN	REST1	28	50
	14	36.79	36.80	35.27	78	6	FAN	REST1	28	50
	15	36.79	36.79	35.31	77	6	FAN	REST1	28	50
	16	36.79	36.79	35.31	83	6	FAN	REST1	28	50
	17	36.79	36.80	35.29	75	6	FAN	REST1	28	50
	18	36.79	36.81	35.31	78	6	FAN	REST1	28	50
	19	36.80	36.79	35.35	79	6	FAN	REST1	28	50
	20	36.80	36.78	35.36	80	6	FAN	REST1	28	50
	21	36.80	36.78	35.35	77	6	FAN	REST1	28	50
	22	36.80	36.80	35.34	78	6	FAN	REST1	28	50
	23	36.81	36.82	35.36	76	6	FAN	REST1	28	50

min	number	Tre	Tes	Tsk-head	HR	ID	condition	period	Ta	RH
	24	36.81	36.82	35.38	80	6	FAN	REST1	28	50
	25	36.81	36.81	35.40	79	6	FAN	REST1	28	50
	26	36.81	36.80	35.41	78	6	FAN	REST1	28	50
	27	36.81	36.80	35.40	83	6	FAN	REST1	28	50
	28	36.81	36.80	35.39	82	6	FAN	REST1	28	50
	29	36.81	36.80	35.40	82	6	FAN	REST1	28	50
	30	36.82	36.82	35.39	82	6	FAN	REST1	28	50
5	31	36.83	36.83	35.38	82	6	FAN	REST1	28	50
	32	36.83	36.83	35.37	81	6	FAN	REST1	28	50
	33	36.82	36.81	35.36	82	6	FAN	REST1	28	50
	34	36.82	36.82	35.36	81	6	FAN	REST1	28	50
	35	36.82	36.82	35.34	81	6	FAN	REST1	28	50
	36	36.82	36.80	35.33	81	6	FAN	REST1	28	50
	37	36.81	36.81	35.32	76	6	FAN	REST1	28	50
	38	36.81	36.80	35.33	79	6	FAN	REST1	28	50
	39	36.81	36.79	35.36	77	6	FAN	REST1	28	50
	40	36.81	36.79	35.36	81	6	FAN	REST1	28	50
	41	36.83	36.76	35.35	83	6	FAN	REST1	28	50
	42	36.83	36.73	35.34	77	6	FAN	REST1	28	50
	43	36.82	36.76	35.33	79	6	FAN	REST1	28	50
	44	36.83	36.77	35.32	81	6	FAN	REST1	28	50
	45	36.83	36.79	35.29	84	6	FAN	REST1	28	50
	46	36.84	36.81	35.31	86	6	FAN	REST1	28	50
	47	36.84	36.79	35.31	82	6	FAN	REST1	28	50
	48	36.84	36.79	35.31	75	6	FAN	REST1	28	50
	49	36.85	36.81	35.33	77	6	FAN	REST1	28	50
	50	36.84	36.81	35.33	77	6	FAN	REST1	28	50
	51	36.84	36.80	35.32	78	6	FAN	REST1	28	50
	52	36.84	36.81	35.30	81	6	FAN	REST1	28	50
	53	36.84	36.81	35.31	86	6	FAN	REST1	28	50
	54	36.85	36.80	35.34	77	6	FAN	REST1	28	50
	55	36.85	36.80	35.36	74	6	FAN	REST1	28	50
	56	36.84	36.81	35.35	80	6	FAN	REST1	28	50
	57	36.85	36.82	35.34	79	6	FAN	REST1	28	50
	58	36.85	36.80	35.34	76	6	FAN	REST1	28	50
	59	36.86	36.81	35.34	79	6	FAN	REST1	28	50
	60	36.86	36.84	35.32	84	6	FAN	REST1	28	50
10	61	36.85	36.77	35.30	85	6	FAN	REST1	28	50
	62	36.85	36.75	35.29	81	6	FAN	REST1	28	50
	63	36.85	36.79	35.28	79	6	FAN	REST1	28	50
	64	36.85	36.79	35.26	79	6	FAN	REST1	28	50
	65	36.85	36.80	35.26	80	6	FAN	REST1	28	50
	66	36.86	36.80	35.25	74	6	FAN	REST1	28	50
	67	36.86	36.79	35.26	86	6	FAN	REST1	28	50
	68	36.85	36.81	35.28	80	6	FAN	REST1	28	50
	69	36.85	36.86	35.29	80	6	FAN	REST1	28	50

min	number	Tre	Tes	Tsk-head	HR	ID	condition	period	Ta	RH
	70	36.85	36.87	35.28	80	6	FAN	REST1	28	50
	71	36.85	36.83	35.29	77	6	FAN	REST1	28	50
	72	36.85	36.79	35.29	79	6	FAN	REST1	28	50
	73	36.85	36.79	35.28	80	6	FAN	REST1	28	50
	74	36.86	36.81	35.27	83	6	FAN	REST1	28	50
	75	36.86	36.82	35.26	82	6	FAN	REST1	28	50
	76	36.86	36.78	35.24	80	6	FAN	REST1	28	50
	77	36.86	36.76	35.22	79	6	FAN	REST1	28	50
	78	36.86	36.79	35.21	79	6	FAN	REST1	28	50
	79	36.86	36.78	35.22	83	6	FAN	REST1	28	50
	80	36.87	36.76	35.23	81	6	FAN	REST1	28	50
	81	36.86	36.77	35.25	82	6	FAN	REST1	28	50
	82	36.86	36.79	35.25	83	6	FAN	REST1	28	50
	83	36.86	36.79	35.24	83	6	FAN	REST1	28	50
	84	36.86	36.77	35.25	82	6	FAN	REST1	28	50
	85	36.86	36.80	35.24	86	6	FAN	REST1	28	50
	86	36.86	36.82	35.22	84	6	FAN	REST1	28	50
	87	36.86	36.80	35.21	83	6	FAN	REST1	28	50
	88	36.87	36.70	35.21	87	6	FAN	REST1	28	50
	89	36.87	36.70	35.21	99	6	FAN	REST1	28	50
	90	36.86	36.78	35.22	82	6	FAN	REST1	28	50
15	91	36.86	36.77	35.22	81	6	FAN	REST1	28	50
	92	36.87	36.78	35.22	84	6	FAN	REST1	28	50
	93	36.86	36.79	35.22	88	6	FAN	REST1	28	50
	94	36.85	36.79	35.21	75	6	FAN	REST1	28	50
	95	36.86	36.76	35.21	82	6	FAN	REST1	28	50
	96	36.85	36.74	35.20	71	6	FAN	REST1	28	50
	97	36.85	36.74	35.20	82	6	FAN	REST1	28	50
	98	36.84	36.68	35.23	84	6	FAN	REST1	28	50
	99	36.84	36.65	35.24	86	6	FAN	REST1	28	50
	100	36.84	36.69	35.23	78	6	FAN	REST1	28	50
	101	36.83	36.71	35.21	80	6	FAN	REST1	28	50
	102	36.84	36.74	35.21	80	6	FAN	REST1	28	50
	103	36.85	36.75	35.21	84	6	FAN	REST1	40	50
	104	36.84	36.75	35.24	91	6	FAN	REST1	40	50
	105	36.83	36.74	35.31	104	6	FAN	REST1	40	50
	106	36.81	36.72	35.42	84	6	FAN	REST1	40	50
	107	36.79	36.70	35.49	97	6	FAN	REST1	40	50
	108	36.77	36.70	35.56	90	6	FAN	REST1	40	50
	109	36.77	36.72	35.61	88	6	FAN	REST1	40	50
	110	36.76	36.70	35.63	82	6	FAN	REST1	40	50
	111	36.75	36.68	35.68	90	6	FAN	REST1	40	50
	112	36.74	36.69	35.72	79	6	FAN	REST1	40	50
	113	36.72	36.62	35.73	84	6	FAN	REST1	40	50
	114	36.71	36.56	35.75	85	6	FAN	REST1	40	50
	115	36.70	36.60	35.78	96	6	FAN	REST1	40	50

min	number	Tre	Tes	Tsk-head	HR	ID	condition	period	Ta	RH
	116	36.70	36.63	35.79	96	6	FAN	REST1	40	50
	117	36.70	36.62	35.80	62	6	FAN	REST1	40	50
	118	36.71	36.61	35.83	78	6	FAN	REST1	40	50
	119	36.72	36.62	35.85	76	6	FAN	REST1	40	50
	120	36.72	36.60	35.88	78	6	FAN	REST1	40	50
20	121	36.73	36.61	35.92	73	6	FAN	REST1	40	50
	122	36.73	36.62	35.96	77	6	FAN	REST1	40	50
	123	36.73	36.62	36.00	83	6	FAN	REST1	40	50
	124	36.74	36.63	36.02	79	6	FAN	REST1	40	50
	125	36.75	36.62	36.03	81	6	FAN	REST1	40	50
	126	36.75	36.61	36.03	79	6	FAN	REST1	40	50
	127	36.75	36.61	36.04	78	6	FAN	REST1	40	50
	128	36.75	36.63	36.06	83	6	FAN	REST1	40	50
	129	36.76	36.62	36.08	90	6	FAN	REST1	40	50
	130	36.76	36.58	36.09	71	6	FAN	REST1	40	50
	131	36.76	36.58	36.09	76	6	FAN	REST1	40	50
	132	36.76	36.59	36.09	83	6	FAN	REST1	40	50
	133	36.76	36.60	36.11	85	6	FAN	REST1	40	50
	134	36.76	36.61	36.11	87	6	FAN	REST1	40	50
	135	36.76	36.61	36.10	100	6	FAN	REST1	40	50
	136	36.76	36.61	36.11	88	6	FAN	REST1	40	50
	137	36.75	36.62	36.12	88	6	FAN	REST1	40	50
	138	36.75	36.62	36.13	89	6	FAN	REST1	40	50
	139	36.76	36.62	36.14	86	6	FAN	EXERCISE1	40	50
	140	36.76	36.61	36.14	85	6	FAN	EXERCISE1	40	50
	141	36.75	36.61	36.15	90	6	FAN	EXERCISE1	40	50
	142	36.73	36.62	36.16	92	6	FAN	EXERCISE1	40	50
	143	36.71	36.61	36.16	99	6	FAN	EXERCISE1	40	50
	144	36.70	36.63	36.16	101	6	FAN	EXERCISE1	40	50
	145	36.68	36.65	36.16	102	6	FAN	EXERCISE1	40	50
	146	36.67	36.63	36.16	104	6	FAN	EXERCISE1	40	50
	147	36.65	36.59	36.16	103	6	FAN	EXERCISE1	40	50
	148	36.64	36.57	36.15	106	6	FAN	EXERCISE1	40	50
	149	36.63	36.56	36.14	105	6	FAN	EXERCISE1	40	50
	150	36.64	36.46	36.15	108	6	FAN	EXERCISE1	40	50
25	151	36.65	36.43	36.16	107	6	FAN	EXERCISE1	40	50
	152	36.64	36.47	36.15	110	6	FAN	EXERCISE1	40	50
	153	36.64	36.48	36.15	112	6	FAN	EXERCISE1	40	50
	154	36.64	36.50	36.17	110	6	FAN	EXERCISE1	40	50
	155	36.64	36.49	36.13	111	6	FAN	EXERCISE1	40	50
	156	36.64	36.49	36.08	109	6	FAN	EXERCISE1	40	50
	157	36.63	36.50	36.08	110	6	FAN	EXERCISE1	40	50
	158	36.62	36.49	36.10	110	6	FAN	EXERCISE1	40	50
	159	36.62	36.45	36.12	107	6	FAN	EXERCISE1	40	50
	160	36.63	36.42	36.13	105	6	FAN	EXERCISE1	40	50
	161	36.63	36.44	36.12	110	6	FAN	EXERCISE1	40	50

min	number	Tre	Tes	Tsk-head	HR	ID	condition	period	Ta	RH
	162	36.62	36.47	36.12	114	6	FAN	EXERCISE1	40	50
	163	36.61	36.50	36.14	112	6	FAN	EXERCISE1	40	50
	164	36.61	36.51	36.12	113	6	FAN	EXERCISE1	40	50
	165	36.62	36.52	36.11	109	6	FAN	EXERCISE1	40	50
	166	36.62	36.55	36.13	108	6	FAN	EXERCISE1	40	50
	167	36.62	36.57	36.14	112	6	FAN	EXERCISE1	40	50
	168	36.62	36.57	36.13	115	6	FAN	EXERCISE1	40	50
	169	36.62	36.57	36.14	115	6	FAN	EXERCISE1	40	50
	170	36.62	36.58	36.15	112	6	FAN	EXERCISE1	40	50
	171	36.62	36.59	36.16	111	6	FAN	EXERCISE1	40	50
	172	36.62	36.61	36.18	112	6	FAN	EXERCISE1	40	50
	173	36.62	36.64	36.20	111	6	FAN	EXERCISE1	40	50
	174	36.63	36.67	36.22	115	6	FAN	EXERCISE1	40	50
	175	36.63	36.59	36.24	120	6	FAN	EXERCISE1	40	50
	176	36.63	36.56	36.25	116	6	FAN	EXERCISE1	40	50
	177	36.64	36.66	36.30	117	6	FAN	EXERCISE1	40	50
	178	36.65	36.68	36.33	114	6	FAN	EXERCISE1	40	50
	179	36.65	36.69	36.35	115	6	FAN	EXERCISE1	40	50
	180	36.66	36.72	36.38	115	6	FAN	EXERCISE1	40	50
30	181	36.66	36.74	36.41	117	6	FAN	EXERCISE1	40	50
	182	36.66	36.74	36.42	120	6	FAN	EXERCISE1	40	50
	183	36.66	36.74	36.44	119	6	FAN	EXERCISE1	40	50
	184	36.65	36.75	36.45	116	6	FAN	EXERCISE1	40	50
	185	36.65	36.77	36.45	116	6	FAN	EXERCISE1	40	50
	186	36.66	36.77	36.45	118	6	FAN	EXERCISE1	40	50
	187	36.66	36.78	36.47	117	6	FAN	EXERCISE1	40	50
	188	36.66	36.78	36.48	119	6	FAN	EXERCISE1	40	50
	189	36.67	36.79	36.48	121	6	FAN	EXERCISE1	40	50
	190	36.67	36.81	36.48	123	6	FAN	EXERCISE1	40	50
	191	36.67	36.80	36.49	122	6	FAN	EXERCISE1	40	50
	192	36.68	36.80	36.50	122	6	FAN	EXERCISE1	40	50
	193	36.69	36.82	36.51	124	6	FAN	EXERCISE1	40	50
	194	36.69	36.81	36.52	124	6	FAN	EXERCISE1	40	50
	195	36.68	36.81	36.53	123	6	FAN	EXERCISE1	40	50
	196	36.68	36.82	36.52	123	6	FAN	EXERCISE1	40	50
	197	36.68	36.81	36.51	125	6	FAN	EXERCISE1	40	50
	198	36.69	36.81	36.52	124	6	FAN	EXERCISE1	40	50
	199	36.70	36.82	36.54	126	6	FAN	EXERCISE1	40	50
	200	36.69	36.81	36.55	124	6	FAN	EXERCISE1	40	50
	201	36.69	36.82	36.55	122	6	FAN	EXERCISE1	40	50
	202	36.69	36.80	36.56	126	6	FAN	EXERCISE1	40	50
	203	36.69	36.80	36.57	126	6	FAN	EXERCISE1	40	50
	204	36.69	36.83	36.55	130	6	FAN	EXERCISE1	40	50
	205	36.69	36.80	36.53	133	6	FAN	EXERCISE1	40	50
	206	36.69	36.79	36.53	133	6	FAN	EXERCISE1	40	50
	207	36.69	36.82	36.53	129	6	FAN	EXERCISE1	40	50

min	number	Tre	Tes	Tsk-head	HR	ID	condition	period	Ta	RH
	208	36.70	36.85	36.55	128	6	FAN	EXERCISE1	40	50
	209	36.70	36.86	36.56	126	6	FAN	EXERCISE1	40	50
	210	36.70	36.86	36.55	125	6	FAN	EXERCISE1	40	50
35	211	36.70	36.86	36.56	128	6	FAN	EXERCISE1	40	50
	212	36.71	36.84	36.55	130	6	FAN	EXERCISE1	40	50
	213	36.72	36.83	36.55	125	6	FAN	EXERCISE1	40	50
	214	36.72	36.84	36.57	125	6	FAN	EXERCISE1	40	50
	215	36.72	36.87	36.57	128	6	FAN	EXERCISE1	40	50
	216	36.73	36.89	36.59	133	6	FAN	EXERCISE1	40	50
	217	36.73	36.88	36.61	132	6	FAN	EXERCISE1	40	50
	218	36.74	36.88	36.61	130	6	FAN	EXERCISE1	40	50
	219	36.75	36.89	36.62	128	6	FAN	EXERCISE1	40	50
	220	36.75	36.90	36.62	128	6	FAN	EXERCISE1	40	50
	221	36.75	36.93	36.62	126	6	FAN	EXERCISE1	40	50
	222	36.75	36.94	36.63	128	6	FAN	EXERCISE1	40	50
	223	36.76	36.90	36.64	126	6	FAN	EXERCISE1	40	50
	224	36.76	36.89	36.65	123	6	FAN	EXERCISE1	40	50
	225	36.76	36.88	36.65	126	6	FAN	EXERCISE1	40	50
	226	36.76	36.90	36.65	127	6	FAN	EXERCISE1	40	50
	227	36.76	36.94	36.65	133	6	FAN	EXERCISE1	40	50
	228	36.76	36.97	36.66	130	6	FAN	EXERCISE1	40	50
	229	36.78	37.04	36.66	126	6	FAN	EXERCISE1	40	50
	230	36.79	37.01	36.66	128	6	FAN	EXERCISE1	40	50
	231	36.79	36.97	36.67	130	6	FAN	EXERCISE1	40	50
	232	36.78	36.99	36.68	131	6	FAN	EXERCISE1	40	50
	233	36.79	36.93	36.69	133	6	FAN	EXERCISE1	40	50
	234	36.79	36.90	36.69	130	6	FAN	EXERCISE1	40	50
	235	36.79	36.95	36.68	128	6	FAN	EXERCISE1	40	50
	236	36.80	36.97	36.68	131	6	FAN	EXERCISE1	40	50
	237	36.81	36.98	36.69	133	6	FAN	EXERCISE1	40	50
	238	36.81	36.99	36.70	132	6	FAN	EXERCISE1	40	50
	239	36.80	37.00	36.70	132	6	FAN	EXERCISE1	40	50
	240	36.80	37.02	36.70	131	6	FAN	EXERCISE1	40	50
40	241	36.81	37.02	36.69	134	6	FAN	EXERCISE1	40	50
	242	36.81	37.01	36.69	136	6	FAN	EXERCISE1	40	50
	243	36.82	37.03	36.71	135	6	FAN	EXERCISE1	40	50
	244	36.82	36.95	36.72	133	6	FAN	EXERCISE1	40	50
	245	36.82	36.92	36.71	134	6	FAN	EXERCISE1	40	50
	246	36.83	36.98	36.73	136	6	FAN	EXERCISE1	40	50
	247	36.83	36.96	36.73	134	6	FAN	EXERCISE1	40	50
	248	36.83	36.97	36.71	128	6	FAN	EXERCISE1	40	50
	249	36.84	37.01	36.70	130	6	FAN	EXERCISE1	40	50
	250	36.84	37.03	36.70	133	6	FAN	EXERCISE1	40	50
	251	36.85	37.04	36.71	135	6	FAN	EXERCISE1	40	50
	252	36.85	37.04	36.70	133	6	FAN	EXERCISE1	40	50
	253	36.87	37.05	36.72	133	6	FAN	EXERCISE1	40	50

min	number	Tre	Tes	Tsk-head	HR	ID	condition	period	Ta	RH
	254	36.88	37.02	36.75	136	6	FAN	EXERCISE1	40	50
	255	36.88	37.02	36.74	134	6	FAN	EXERCISE1	40	50
	256	36.88	37.05	36.75	133	6	FAN	EXERCISE1	40	50
	257	36.88	37.05	36.77	136	6	FAN	EXERCISE1	40	50
	258	36.88	37.05	36.78	137	6	FAN	EXERCISE1	40	50
	259	36.89	37.07	36.79	137	6	FAN	EXERCISE1	40	50
	260	36.89	37.09	36.81	136	6	FAN	EXERCISE1	40	50
	261	36.89	37.10	36.82	136	6	FAN	EXERCISE1	40	50
	262	36.89	37.10	36.82	136	6	FAN	EXERCISE1	40	50
	263	36.90	37.11	36.83	134	6	FAN	EXERCISE1	40	50
	264	36.92	37.12	36.85	134	6	FAN	EXERCISE1	40	50
	265	36.92	37.15	36.86	134	6	FAN	EXERCISE1	40	50
	266	36.92	37.16	36.86	136	6	FAN	EXERCISE1	40	50
	267	36.92	37.09	36.85	139	6	FAN	EXERCISE1	40	50
	268	36.92	37.06	36.84	136	6	FAN	EXERCISE1	40	50
	269	36.92	37.12	36.85	137	6	FAN	EXERCISE1	40	50
	270	36.93	37.14	36.87	136	6	FAN	EXERCISE1	40	50
45	271	36.94	37.16	36.87	139	6	FAN	EXERCISE1	40	50
	272	36.95	37.18	36.85	142	6	FAN	EXERCISE1	40	50
	273	36.94	37.13	36.83	141	6	FAN	EXERCISE1	40	50
	274	36.93	37.10	36.82	136	6	FAN	EXERCISE1	40	50
	275	36.94	37.12	36.81	138	6	FAN	EXERCISE1	40	50
	276	36.95	37.13	36.82	139	6	FAN	EXERCISE1	40	50
	277	36.96	37.13	36.84	141	6	FAN	EXERCISE1	40	50
	278	36.96	37.12	36.86	136	6	FAN	EXERCISE1	40	50
	279	36.96	37.16	36.86	136	6	FAN	EXERCISE1	40	50
	280	36.96	37.19	36.86	137	6	FAN	EXERCISE1	40	50
	281	36.95	37.18	36.86	139	6	FAN	EXERCISE1	40	50
	282	36.95	37.18	36.86	140	6	FAN	EXERCISE1	40	50
	283	36.95	37.20	36.87	140	6	FAN	EXERCISE1	40	50
	284	36.96	37.20	36.88	143	6	FAN	EXERCISE1	40	50
	285	36.97	37.20	36.89	142	6	FAN	EXERCISE1	40	50
	286	36.97	37.18	36.90	142	6	FAN	EXERCISE1	40	50
	287	36.99	37.18	36.91	140	6	FAN	EXERCISE1	40	50
	288	37.00	37.18	36.91	143	6	FAN	EXERCISE1	40	50
	289	37.00	37.19	36.91	141	6	FAN	EXERCISE1	40	50
	290	37.01	37.20	36.91	140	6	FAN	EXERCISE1	40	50
	291	37.01	37.21	36.91	139	6	FAN	EXERCISE1	40	50
	292	37.02	37.22	36.93	139	6	FAN	EXERCISE1	40	50
	293	37.03	37.22	36.91	139	6	FAN	EXERCISE1	40	50
	294	37.03	37.21	36.88	141	6	FAN	EXERCISE1	40	50
	295	37.03	37.19	36.89	141	6	FAN	EXERCISE1	40	50
	296	37.03	37.20	36.89	139	6	FAN	EXERCISE1	40	50
	297	37.03	37.23	36.89	141	6	FAN	EXERCISE1	40	50
	298	37.03	37.25	36.91	142	6	FAN	EXERCISE1	40	50
	299	37.04	37.27	36.91	142	6	FAN	EXERCISE1	40	50

min	number	Tre	Tes	Tsk-head	HR	ID	condition	period	Ta	RH
	300	37.04	37.25	36.90	143	6	FAN	EXERCISE1	40	50
50	301	37.06	37.24	36.91	141	6	FAN	EXERCISE1	40	50
	302	37.07	37.27	36.90	143	6	FAN	EXERCISE1	40	50
	303	37.08	37.26	36.89	142	6	FAN	EXERCISE1	40	50
	304	37.08	37.26	36.89	141	6	FAN	EXERCISE1	40	50
	305	37.08	37.27	36.88	143	6	FAN	EXERCISE1	40	50
	306	37.09	37.28	36.89	146	6	FAN	EXERCISE1	40	50
	307	37.09	37.30	36.91	146	6	FAN	EXERCISE1	40	50
	308	37.09	37.30	36.92	144	6	FAN	EXERCISE1	40	50
	309	37.10	37.30	36.94	143	6	FAN	EXERCISE1	40	50
	310	37.10	37.31	36.94	141	6	FAN	EXERCISE1	40	50
	311	37.10	37.32	36.94	141	6	FAN	EXERCISE1	40	50
	312	37.11	37.31	36.92	143	6	FAN	EXERCISE1	40	50
	313	37.12	37.29	36.92	145	6	FAN	EXERCISE1	40	50
	314	37.12	37.29	36.94	143	6	FAN	EXERCISE1	40	50
	315	37.13	37.31	36.96	144	6	FAN	EXERCISE1	40	50
	316	37.13	37.32	36.98	146	6	FAN	EXERCISE1	40	50
	317	37.14	37.33	36.99	147	6	FAN	EXERCISE1	40	50
	318	37.14	37.35	37.00	146	6	FAN	EXERCISE1	40	50
	319	37.14	37.35	37.00	147	6	FAN	EXERCISE1	40	50
	320	37.15	37.36	37.00	143	6	FAN	REST2	28	50
	321	37.15	37.37	36.97	140	6	FAN	REST2	28	50
	322	37.15	37.37	36.91	140	6	FAN	REST2	28	50
	323	37.15	37.38	36.86	139	6	FAN	REST2	28	50
	324	37.16	37.42	36.80	130	6	FAN	REST2	28	50
	325	37.16	37.45	36.56	118	6	FAN	REST2	28	50
	326	37.16	37.45	36.30	120	6	FAN	REST2	28	50
	327	37.16	37.46	36.28	115	6	FAN	REST2	28	50
	328	37.17	37.42	36.31	113	6	FAN	REST2	28	50
	329	37.17	37.37	36.31	114	6	FAN	REST2	28	50
	330	37.18	37.40	36.30	111	6	FAN	REST2	28	50
55	331	37.18	37.42	36.21	106	6	FAN	REST2	28	50
	332	37.18	37.42	36.15	104	6	FAN	REST2	28	50
	333	37.18	37.42	36.13	103	6	FAN	REST2	28	50
	334	37.17	37.37	36.10	103	6	FAN	REST2	28	50
	335	37.18	37.35	36.10	103	6	FAN	REST2	28	50
	336	37.18	37.39	36.06	106	6	FAN	REST2	28	50
	337	37.18	37.38	36.03	102	6	FAN	REST2	28	50
	338	37.18	34.26	36.01	105	6	FAN	REST2	28	50
	339	37.18	32.97	36.01	108	6	FAN	REST2	28	50
	340	37.18	35.10	36.02	97	6	FAN	REST2	28	50
	341	37.19	35.51	36.01	103	6	FAN	REST2	28	50
	342	37.20	35.66	35.99	100	6	FAN	REST2	28	50
	343	37.20	35.84	35.96	98	6	FAN	REST2	28	50
	344	37.20	36.05	35.97	98	6	FAN	REST2	28	50
	345	37.20	31.29	35.96	100	6	FAN	REST2	28	50

min	number	Tre	Tes	Tsk-head	HR	ID	condition	period	Ta	RH
	346	37.20	27.42	35.95	86	6	FAN	REST2	28	50
	347	37.21	27.55	35.95	96	6	FAN	REST2	28	50
	348	37.21	27.91	35.95	91	6	FAN	REST2	28	50
	349	37.21	29.84	35.98	95	6	FAN	REST2	28	50
	350	37.20	31.16	35.98	97	6	FAN	REST2	28	50
	351	37.20	31.88	35.96	92	6	FAN	REST2	28	50
	352	37.21	32.20	35.96	93	6	FAN	REST2	28	50
	353	37.20	32.56	35.95	95	6	FAN	REST2	28	50
	354	37.20	32.95	35.95	94	6	FAN	REST2	28	50
	355	37.20	33.29	35.94	95	6	FAN	REST2	28	50
	356	37.20	33.68	35.91	96	6	FAN	REST2	28	50
	357	37.20	34.25	35.89	95	6	FAN	REST2	28	50
	358	37.20	34.65	35.80	88	6	FAN	REST2	28	50
	359	37.20	34.85	35.74	91	6	FAN	REST2	28	50
	360	37.20	35.01	35.74	90	6	FAN	REST2	28	50
60	361	37.20	35.17	35.70	91	6	FAN	REST2	28	50
	362	37.20	30.46	35.60	97	6	FAN	REST2	28	50
	363	37.21	27.07	35.49	79	6	FAN	REST2	28	50
	364	37.21	29.77	35.43	92	6	FAN	REST2	28	50
	365	37.21	28.90	35.36	97	6	FAN	REST2	28	50
	366	37.20	27.35	35.31	82	6	FAN	REST2	28	50
	367	37.20	29.33	35.24	83	6	FAN	REST2	28	50
	368	37.21	31.08	35.15	89	6	FAN	REST2	28	50
	369	37.21	31.79	35.08	83	6	FAN	REST2	28	50
	370	37.22	32.41	35.05	85	6	FAN	REST2	28	50
	371	37.22	30.01	34.99	87	6	FAN	REST2	28	50
	372	37.21	29.16	34.92	80	6	FAN	REST2	28	50
	373	37.21	32.17	34.88	86	6	FAN	REST2	28	50
	374	37.22	33.56	34.87	86	6	FAN	REST2	28	50
	375	37.21	30.15	34.82	91	6	FAN	REST2	28	50
	376	37.21	28.72	34.72	80	6	FAN	REST2	28	50
	377	37.21	31.55	34.67	78	6	FAN	REST2	28	50
	378	37.21	29.69	34.63	80	6	FAN	REST2	28	50
	379	37.21	28.76	34.57	81	6	FAN	REST2	28	50
	380	37.21	30.69	34.51	89	6	FAN	REST2	28	50
	381	37.21	31.69	34.42	78	6	FAN	REST2	28	50
	382	37.21	32.50	34.32	78	6	FAN	REST2	28	50
	383	37.21	32.86	34.29	81	6	FAN	REST2	28	50
	384	37.21	33.25	34.24	82	6	FAN	REST2	28	50
	385	37.21	29.95	34.21	87	6	FAN	REST2	28	50
	386	37.21	26.88	34.25	85	6	FAN	REST2	28	50
	387	37.21	28.69	34.33	91	6	FAN	REST2	28	50
	388	37.21	30.34	34.36	79	6	FAN	REST2	28	50
	389	37.21	31.00	34.32	77	6	FAN	REST2	28	50
	390	37.21	31.60	34.35	80	6	FAN	REST2	28	50
65	391	37.22	32.12	34.42	73	6	FAN	REST2	28	50

min	number	Tre	Tes	Tsk-head	HR	ID	condition	period	Ta	RH
	392	37.21	32.57	34.46	74	6	FAN	REST2	28	50
	393	37.21	32.95	34.49	77	6	FAN	REST2	28	50
	394	37.21	33.28	34.52	78	6	FAN	REST2	28	50
	395	37.22	33.51	34.55	78	6	FAN	REST2	28	50
	396	37.21	33.74	34.57	78	6	FAN	REST2	28	50
	397	37.21	33.96	34.60	78	6	FAN	REST2	28	50
	398	37.21	34.25	34.63	80	6	FAN	REST2	28	50
	399	37.21	34.55	34.64	80	6	FAN	REST2	28	50
	400	37.20	34.75	34.67	80	6	FAN	REST2	28	50
	401	37.21	34.94	34.68	79	6	FAN	REST2	28	50
	402	37.22	35.21	34.70	79	6	FAN	REST2	28	50
	403	37.21	35.44	34.70	80	6	FAN	REST2	28	50
	404	37.20	35.57	34.68	77	6	FAN	REST2	28	50
	405	37.20	35.68	34.67	76	6	FAN	REST2	28	50
	406	37.21	35.80	34.68	74	6	FAN	REST2	28	50
	407	37.22	35.89	34.73	69	6	FAN	REST2	28	50
	408	37.21	36.13	34.75	79	6	FAN	REST2	28	50
	409	37.21	36.26	34.73	88	6	FAN	REST2	28	50
	410	37.22	36.26	34.72	72	6	FAN	REST2	28	50
	411	37.21	36.37	34.71	78	6	FAN	REST2	28	50
	412	37.21	36.42	34.70	77	6	FAN	REST2	28	50
	413	37.21	36.46	34.73	77	6	FAN	REST2	28	50
	414	37.21	36.51	34.74	76	6	FAN	REST2	28	50
	415	37.20	36.51	34.56	80	6	FAN	REST2	28	50
	416	37.20	36.48	34.51	80	6	FAN	REST2	28	50
	417	37.21	36.59	34.67	81	6	FAN	REST2	28	50
	418	37.21	36.68	34.68	74	6	FAN	REST2	28	50
	419	37.21	36.70	34.64	75	6	FAN	REST2	28	50
	420	37.21	36.72	34.65	78	6	FAN	REST2	28	50
70	421	37.21	36.74	34.66	80	6	FAN	REST2	28	50
	422	37.21	36.78	34.65	78	6	FAN	REST2	28	50
	423	37.20	36.78	34.69	72	6	FAN	REST2	28	50
	424	37.20	36.79	34.72	76	6	FAN	REST2	28	50
	425	37.20	36.81	34.72	78	6	FAN	REST2	28	50
	426	37.20	36.82	34.70	79	6	FAN	REST2	28	50
	427	37.21	36.80	34.69	80	6	FAN	REST2	28	50
	428	37.20	36.80	34.68	82	6	FAN	REST2	28	50
	429	37.20	36.86	34.66	78	6	FAN	REST2	28	50
	430	37.20	36.88	34.65	74	6	FAN	REST2	28	50
	431	37.20	36.90	34.68	75	6	FAN	REST2	28	50
	432	37.19	36.92	34.69	76	6	FAN	REST2	28	50
	433	37.19	36.92	34.66	77	6	FAN	REST2	28	50
	434	37.20	36.84	34.66	78	6	FAN	REST2	28	50
	435	37.20	36.83	34.67	77	6	FAN	REST2	28	50
	436	37.19	36.91	34.68	73	6	FAN	REST2	28	50
	437	37.18	36.92	34.69	73	6	FAN	REST2	28	50

min	number	Tre	Tes	Tsk-head	HR	ID	condition	period	Ta	RH
	438	37.19	36.95	34.65	76	6	FAN	REST2	28	50
	439	37.19	36.98	34.66	75	6	FAN	REST2	40	50
	440	37.19	36.98	34.70	87	6	FAN	REST2	40	50
	441	37.20	36.99	34.78	90	6	FAN	REST2	40	50
	442	37.20	36.98	34.94	96	6	FAN	REST2	40	50
	443	37.20	36.97	35.11	92	6	FAN	REST2	40	50
	444	37.21	36.97	35.24	95	6	FAN	REST2	40	50
	445	37.23	36.94	35.33	85	6	FAN	REST2	40	50
	446	37.24	36.92	35.44	82	6	FAN	REST2	40	50
	447	37.25	36.93	35.52	78	6	FAN	REST2	40	50
	448	37.25	36.93	35.56	79	6	FAN	REST2	40	50
	449	37.24	36.92	35.58	81	6	FAN	REST2	40	50
	450	37.24	36.92	35.62	83	6	FAN	REST2	40	50
75	451	37.25	36.92	35.65	81	6	FAN	REST2	40	50
	452	37.25	36.92	35.67	85	6	FAN	REST2	40	50
	453	37.26	36.92	35.70	80	6	FAN	REST2	40	50
	454	37.26	36.90	35.70	85	6	FAN	REST2	40	50
	455	37.26	36.88	35.71	82	6	FAN	REST2	40	50
	456	37.26	36.90	35.74	84	6	FAN	REST2	40	50
	457	37.27	36.90	35.78	82	6	FAN	REST2	40	50
	458	37.28	36.89	35.77	92	6	FAN	REST2	40	50
	459	37.27	36.89	35.77	106	6	FAN	REST2	40	50
	460	37.26	36.90	35.79	89	6	FAN	REST2	40	50
	461	37.26	36.91	35.79	93	6	FAN	REST2	40	50
	462	37.25	36.90	35.80	88	6	FAN	REST2	40	50
	463	37.25	36.89	35.83	87	6	FAN	EXERCISE2	40	50
	464	37.25	36.89	35.83	86	6	FAN	EXERCISE2	40	50
	465	37.24	36.68	35.83	94	6	FAN	EXERCISE2	40	50
	466	37.23	36.49	35.81	100	6	FAN	EXERCISE2	40	50
	467	37.22	36.62	35.80	102	6	FAN	EXERCISE2	40	50
	468	37.21	36.75	35.81	106	6	FAN	EXERCISE2	40	50
	469	37.20	36.77	35.81	105	6	FAN	EXERCISE2	40	50
	470	37.20	36.79	35.82	109	6	FAN	EXERCISE2	40	50
	471	37.20	36.82	35.84	111	6	FAN	EXERCISE2	40	50
	472	37.19	36.83	35.84	112	6	FAN	EXERCISE2	40	50
	473	37.19	36.82	35.86	110	6	FAN	EXERCISE2	40	50
	474	37.19	36.75	35.91	109	6	FAN	EXERCISE2	40	50
	475	37.19	36.75	35.95	107	6	FAN	EXERCISE2	40	50
	476	37.18	36.83	35.99	109	6	FAN	EXERCISE2	40	50
	477	37.18	36.85	36.03	105	6	FAN	EXERCISE2	40	50
	478	37.18	36.86	36.07	111	6	FAN	EXERCISE2	40	50
	479	37.18	36.87	36.11	114	6	FAN	EXERCISE2	40	50
	480	37.18	36.88	36.11	113	6	FAN	EXERCISE2	40	50
80	481	37.18	36.88	36.13	129	6	FAN	EXERCISE2	40	50
	482	37.18	36.88	36.18	115	6	FAN	EXERCISE2	40	50
	483	37.18	36.88	36.24	113	6	FAN	EXERCISE2	40	50

min	number	Tre	Tes	Tsk-head	HR	ID	condition	period	Ta	RH
	484	37.18	36.89	36.28	112	6	FAN	EXERCISE2	40	50
	485	37.17	36.90	36.30	114	6	FAN	EXERCISE2	40	50
	486	37.18	36.91	36.30	117	6	FAN	EXERCISE2	40	50
	487	37.18	36.91	36.32	118	6	FAN	EXERCISE2	40	50
	488	37.17	36.93	36.34	115	6	FAN	EXERCISE2	40	50
	489	37.16	36.94	36.36	115	6	FAN	EXERCISE2	40	50
	490	37.16	36.94	36.36	117	6	FAN	EXERCISE2	40	50
	491	37.17	36.94	36.35	119	6	FAN	EXERCISE2	40	50
	492	37.17	36.97	36.38	119	6	FAN	EXERCISE2	40	50
	493	37.17	36.94	36.41	120	6	FAN	EXERCISE2	40	50
	494	37.17	36.92	36.43	120	6	FAN	EXERCISE2	40	50
	495	37.18	36.94	36.43	119	6	FAN	EXERCISE2	40	50
	496	37.18	36.95	36.43	118	6	FAN	EXERCISE2	40	50
	497	37.18	36.98	36.46	119	6	FAN	EXERCISE2	40	50
	498	37.19	36.98	36.49	119	6	FAN	EXERCISE2	40	50
	499	37.19	36.98	36.48	119	6	FAN	EXERCISE2	40	50
	500	37.19	36.98	36.48	122	6	FAN	EXERCISE2	40	50
	501	37.19	36.91	36.52	125	6	FAN	EXERCISE2	40	50
	502	37.19	36.91	36.55	122	6	FAN	EXERCISE2	40	50
	503	37.19	36.98	36.57	120	6	FAN	EXERCISE2	40	50
	504	37.19	36.99	36.59	120	6	FAN	EXERCISE2	40	50
	505	37.18	36.98	36.59	126	6	FAN	EXERCISE2	40	50
	506	37.18	36.99	36.59	123	6	FAN	EXERCISE2	40	50
	507	37.19	37.01	36.59	123	6	FAN	EXERCISE2	40	50
	508	37.19	37.03	36.57	124	6	FAN	EXERCISE2	40	50
	509	37.19	37.01	36.57	122	6	FAN	EXERCISE2	40	50
	510	37.18	37.00	36.56	122	6	FAN	EXERCISE2	40	50
85	511	37.18	37.01	36.54	125	6	FAN	EXERCISE2	40	50
	512	37.19	37.04	36.54	127	6	FAN	EXERCISE2	40	50
	513	37.19	37.05	36.56	123	6	FAN	EXERCISE2	40	50
	514	37.19	37.03	36.58	123	6	FAN	EXERCISE2	40	50
	515	37.19	37.02	36.59	126	6	FAN	EXERCISE2	40	50
	516	37.18	37.04	36.60	127	6	FAN	EXERCISE2	40	50
	517	37.19	37.02	36.59	125	6	FAN	EXERCISE2	40	50
	518	37.19	36.99	36.58	122	6	FAN	EXERCISE2	40	50
	519	37.20	37.02	36.58	119	6	FAN	EXERCISE2	40	50
	520	37.19	37.04	36.56	119	6	FAN	EXERCISE2	40	50
	521	37.19	37.04	36.55	123	6	FAN	EXERCISE2	40	50
	522	37.20	37.03	36.57	126	6	FAN	EXERCISE2	40	50
	523	37.20	37.03	36.57	127	6	FAN	EXERCISE2	40	50
	524	37.20	37.06	36.56	126	6	FAN	EXERCISE2	40	50
	525	37.21	37.08	36.58	125	6	FAN	EXERCISE2	40	50
	526	37.21	37.09	36.59	126	6	FAN	EXERCISE2	40	50
	527	37.21	37.08	36.60	128	6	FAN	EXERCISE2	40	50
	528	37.20	37.07	36.61	126	6	FAN	EXERCISE2	40	50
	529	37.20	37.07	36.61	125	6	FAN	EXERCISE2	40	50

min	number	Tre	Tes	Tsk-head	HR	ID	condition	period	Ta	RH
	530	37.21	37.03	36.62	128	6	FAN	EXERCISE2	40	50
	531	37.21	37.02	36.64	124	6	FAN	EXERCISE2	40	50
	532	37.21	37.07	36.65	123	6	FAN	EXERCISE2	40	50
	533	37.21	37.09	36.62	126	6	FAN	EXERCISE2	40	50
	534	37.22	37.09	36.62	129	6	FAN	EXERCISE2	40	50
	535	37.22	37.10	36.66	130	6	FAN	EXERCISE2	40	50
	536	37.23	37.10	36.67	130	6	FAN	EXERCISE2	40	50
	537	37.23	37.11	36.67	131	6	FAN	EXERCISE2	40	50
	538	37.23	37.13	36.66	129	6	FAN	EXERCISE2	40	50
	539	37.23	37.09	36.66	130	6	FAN	EXERCISE2	40	50
	540	37.23	37.08	36.67	126	6	FAN	EXERCISE2	40	50
90	541	37.22	37.10	36.68	125	6	FAN	EXERCISE2	40	50
	542	37.23	37.10	36.64	127	6	FAN	EXERCISE2	40	50
	543	37.24	37.12	36.66	126	6	FAN	EXERCISE2	40	50
	544	37.24	37.13	36.70	129	6	FAN	EXERCISE2	40	50
	545	37.23	37.12	36.70	131	6	FAN	EXERCISE2	40	50
	546	37.24	37.14	36.71	133	6	FAN	EXERCISE2	40	50
	547	37.24	37.15	36.71	135	6	FAN	EXERCISE2	40	50
	548	37.24	37.13	36.72	130	6	FAN	EXERCISE2	40	50
	549	37.24	37.13	36.72	129	6	FAN	EXERCISE2	40	50
	550	37.25	37.14	36.72	129	6	FAN	EXERCISE2	40	50
	551	37.26	37.16	36.74	133	6	FAN	EXERCISE2	40	50
	552	37.26	37.14	36.75	137	6	FAN	EXERCISE2	40	50
	553	37.27	37.12	36.76	136	6	FAN	EXERCISE2	40	50
	554	37.26	37.13	36.75	137	6	FAN	EXERCISE2	40	50
	555	37.26	37.14	36.73	136	6	FAN	EXERCISE2	40	50
	556	37.26	37.16	36.72	137	6	FAN	EXERCISE2	40	50
	557	37.26	37.02	36.72	137	6	FAN	EXERCISE2	40	50
	558	37.27	36.98	36.73	134	6	FAN	EXERCISE2	40	50
	559	37.27	37.08	36.73	135	6	FAN	EXERCISE2	40	50
	560	37.27	37.11	36.72	134	6	FAN	EXERCISE2	40	50
	561	37.27	37.14	36.71	135	6	FAN	EXERCISE2	40	50
	562	37.27	37.15	36.71	136	6	FAN	EXERCISE2	40	50
	563	37.28	37.15	36.71	136	6	FAN	EXERCISE2	40	50
	564	37.29	37.16	36.73	134	6	FAN	EXERCISE2	40	50
	565	37.29	37.18	36.74	135	6	FAN	EXERCISE2	40	50
	566	37.30	37.19	36.74	137	6	FAN	EXERCISE2	40	50
	567	37.30	37.21	36.75	136	6	FAN	EXERCISE2	40	50
	568	37.31	37.21	36.76	135	6	FAN	EXERCISE2	40	50
	569	37.31	37.19	36.78	135	6	FAN	EXERCISE2	40	50
	570	37.31	37.19	36.79	137	6	FAN	EXERCISE2	40	50
95	571	37.31	37.21	36.79	143	6	FAN	EXERCISE2	40	50
	572	37.31	37.21	36.80	143	6	FAN	EXERCISE2	40	50
	573	37.31	37.16	36.81	142	6	FAN	EXERCISE2	40	50
	574	37.31	37.13	36.81	139	6	FAN	EXERCISE2	40	50
	575	37.31	37.16	36.81	139	6	FAN	EXERCISE2	40	50

min	number	Tre	Tes	Tsk-head	HR	ID	condition	period	Ta	RH
	576	37.30	37.17	36.81	135	6	FAN	EXERCISE2	40	50
	577	37.30	37.19	36.83	134	6	FAN	EXERCISE2	40	50
	578	37.30	37.19	36.82	139	6	FAN	EXERCISE2	40	50
	579	37.30	37.19	36.81	140	6	FAN	EXERCISE2	40	50
	580	37.30	37.21	36.80	142	6	FAN	EXERCISE2	40	50
	581	37.30	37.22	36.75	143	6	FAN	EXERCISE2	40	50
	582	37.30	37.22	36.73	143	6	FAN	EXERCISE2	40	50
	583	37.31	37.21	36.75	140	6	FAN	EXERCISE2	40	50
	584	37.31	37.21	36.78	143	6	FAN	EXERCISE2	40	50
	585	37.32	37.24	36.80	143	6	FAN	EXERCISE2	40	50
	586	37.33	37.12	36.81	143	6	FAN	EXERCISE2	40	50
	587	37.33	37.09	36.81	140	6	FAN	EXERCISE2	40	50
	588	37.33	37.19	36.81	140	6	FAN	EXERCISE2	40	50
	589	37.33	37.22	36.81	140	6	FAN	EXERCISE2	40	50
	590	37.34	37.25	36.82	140	6	FAN	EXERCISE2	40	50
	591	37.35	37.18	36.81	143	6	FAN	EXERCISE2	40	50
	592	37.35	37.17	36.80	140	6	FAN	EXERCISE2	40	50
	593	37.35	37.24	36.81	141	6	FAN	EXERCISE2	40	50
	594	37.35	37.26	36.82	140	6	FAN	EXERCISE2	40	50
	595	37.36	37.27	36.82	140	6	FAN	EXERCISE2	40	50
	596	37.36	37.29	36.81	140	6	FAN	EXERCISE2	40	50
	597	37.37	37.30	36.81	140	6	FAN	EXERCISE2	40	50
	598	37.37	37.30	36.83	143	6	FAN	EXERCISE2	40	50
	599	37.37	37.29	36.84	146	6	FAN	EXERCISE2	40	50
	600	37.37	37.27	36.84	146	6	FAN	EXERCISE2	40	50
100	601	37.38	37.29	36.83	143	6	FAN	EXERCISE2	40	50
	602	37.38	37.30	36.85	140	6	FAN	EXERCISE2	40	50
	603	37.38	37.31	36.87	141	6	FAN	EXERCISE2	40	50
	604	37.39	37.31	36.87	143	6	FAN	EXERCISE2	40	50
	605	37.40	37.31	36.84	147	6	FAN	EXERCISE2	40	50
	606	37.40	37.29	36.84	150	6	FAN	EXERCISE2	40	50
	607	37.40	37.27	36.88	148	6	FAN	EXERCISE2	40	50
	608	37.41	37.29	36.90	146	6	FAN	EXERCISE2	40	50
	609	37.42	37.33	36.90	146	6	FAN	EXERCISE2	40	50
	610	37.43	37.36	36.89	144	6	FAN	EXERCISE2	40	50
	611	37.43	37.35	36.89	143	6	FAN	EXERCISE2	40	50
	612	37.43	37.34	36.89	143	6	FAN	EXERCISE2	40	50
	613	37.44	37.36	36.89	143	6	FAN	EXERCISE2	40	50
	614	37.46	37.38	36.91	144	6	FAN	EXERCISE2	40	50
	615	37.46	37.39	36.88	146	6	FAN	EXERCISE2	40	50
	616	37.46	37.38	36.88	146	6	FAN	EXERCISE2	40	50
	617	37.46	37.39	36.89	146	6	FAN	EXERCISE2	40	50
	618	37.47	37.41	36.88	144	6	FAN	EXERCISE2	40	50
	619	37.47	37.40	36.90	144	6	FAN	EXERCISE2	40	50
	620	37.47	37.35	36.90	147	6	FAN	EXERCISE2	40	50
	621	37.48	37.33	36.92	148	6	FAN	EXERCISE2	40	50

min	number	Tre	Tes	Tsk-head	HR	ID	condition	period	Ta	RH
	622	37.49	37.37	36.96	146	6	FAN	EXERCISE2	40	50
	623	37.49	37.38	36.98	146	6	FAN	EXERCISE2	40	50
	624	37.49	37.39	36.98	143	6	FAN	EXERCISE2	40	50
	625	37.49	37.40	36.97	144	6	FAN	EXERCISE2	40	50
	626	37.49	37.41	36.95	146	6	FAN	EXERCISE2	40	50
	627	37.49	37.42	36.94	146	6	FAN	EXERCISE2	40	50
	628	37.49	37.42	36.94	146	6	FAN	EXERCISE2	40	50
	629	37.50	37.42	36.97	144	6	FAN	EXERCISE2	40	50
	630	37.51	37.43	36.99	143	6	FAN	EXERCISE2	40	50
105	631	37.51	37.32	36.97	146	6	FAN	EXERCISE2	40	50
	632	37.52	37.30	36.98	150	6	FAN	EXERCISE2	40	50
	633	37.52	37.40	36.98	148	6	FAN	EXERCISE2	40	50
	634	37.51	37.42	36.98	146	6	FAN	EXERCISE2	40	50
	635	37.52	37.43	37.00	146	6	FAN	EXERCISE2	40	50
	636	37.54	37.46	36.99	145	6	FAN	EXERCISE2	40	50
	637	37.54	37.46	36.96	146	6	FAN	EXERCISE2	40	50
	638	37.53	37.45	36.98	147	6	FAN	EXERCISE2	40	50
	639	37.54	37.45	37.01	147	6	FAN	EXERCISE2	40	50
	640	37.55	37.45	37.01	148	6	FAN	EXERCISE2	40	50
	641	37.54	37.46	37.01	149	6	FAN	EXERCISE2	40	50
	642	37.55	37.47	37.02	147	6	FAN	EXERCISE2	40	50
	643	37.56	37.50	37.03	147	6	FAN	EXERCISE2	40	50
	644	37.56	37.53	37.03	145	6	FAN	REST3	28	50
	645	37.57	37.51	37.01	143	6	FAN	REST3	28	50
	646	37.58	37.52	36.94	150	6	FAN	REST3	28	50
	647	37.58	37.57	36.80	143	6	FAN	REST3	28	50
	648	37.59	37.54	36.64	128	6	FAN	REST3	28	50
	649	37.60	37.52	36.52	119	6	FAN	REST3	28	50
	650	37.60	37.59	36.48	116	6	FAN	REST3	28	50
	651	37.59	37.61	36.49	116	6	FAN	REST3	28	50
	652	37.59	37.61	36.51	115	6	FAN	REST3	28	50
	653	37.60	37.55	36.53	117	6	FAN	REST3	28	50
	654	37.59	37.54	36.53	111	6	FAN	REST3	28	50
	655	37.59	37.59	36.53	112	6	FAN	REST3	28	50
	656	37.59	37.60	36.50	114	6	FAN	REST3	28	50
	657	37.59	37.62	36.46	109	6	FAN	REST3	28	50
	658	37.59	37.63	36.41	111	6	FAN	REST3	28	50
	659	37.59	37.63	36.37	108	6	FAN	REST3	28	50
	660	37.58	37.62	36.35	105	6	FAN	REST3	28	50
110	661	37.58	37.61	36.34	105	6	FAN	REST3	28	50
	662	37.59	37.62	36.32	103	6	FAN	REST3	28	50
	663	37.59	37.62	36.32	104	6	FAN	REST3	28	50
	664	37.59	37.61	36.33	104	6	FAN	REST3	28	50
	665	37.59	37.61	36.31	106	6	FAN	REST3	28	50
	666	37.59	37.62	36.29	106	6	FAN	REST3	28	50
	667	37.60	37.63	36.28	107	6	FAN	REST3	28	50

min	number	Tre	Tes	Tsk-head	HR	ID	condition	period	Ta	RH
	668	37.60	37.61	36.25	102	6	FAN	REST3	28	50
	669	37.60	37.60	36.24	101	6	FAN	REST3	28	50
	670	37.60	37.61	36.25	105	6	FAN	REST3	28	50
	671	37.60	37.51	36.25	104	6	FAN	REST3	28	50
	672	37.61	37.48	36.24	100	6	FAN	REST3	28	50
	673	37.61	37.53	36.22	102	6	FAN	REST3	28	50
	674	37.61	37.52	36.19	100	6	FAN	REST3	28	50
	675	37.61	37.53	36.17	101	6	FAN	REST3	28	50
	676	37.61	37.54	36.14	97	6	FAN	REST3	28	50
	677	37.61	37.54	36.09	98	6	FAN	REST3	28	50
	678	37.61	37.53	36.04	95	6	FAN	REST3	28	50
	679	37.61	37.52	35.98	100	6	FAN	REST3	28	50
	680	37.62	37.52	35.92	101	6	FAN	REST3	28	50
	681	37.62	37.51	35.85	96	6	FAN	REST3	28	50
	682	37.62	37.51	35.68	97	6	FAN	REST3	28	50
	683	37.62	37.51	35.52	95	6	FAN	REST3	28	50
	684	37.63	37.50	35.21	95	6	FAN	REST3	28	50
	685	37.63	37.50	34.98	94	6	FAN	REST3	28	50
	686	37.63	37.53	34.98	97	6	FAN	REST3	28	50
	687	37.64	37.58	35.04	95	6	FAN	REST3	28	50
	688	37.63	37.60	35.14	96	6	FAN	REST3	28	50
	689	37.63	37.60	35.16	97	6	FAN	REST3	28	50
	690	37.63	37.59	35.19	96	6	FAN	REST3	28	50
115	691	37.64	37.58	35.19	97	6	FAN	REST3	28	50
	692	37.64	37.56	35.17	99	6	FAN	REST3	28	50
	693	37.64	37.56	35.14	97	6	FAN	REST3	28	50
	694	37.64	37.59	35.09	93	6	FAN	REST3	28	50
	695	37.65	37.61	34.82	95	6	FAN	REST3	28	50
	696	37.66	37.60	34.63	97	6	FAN	REST3	28	50
	697	37.66	37.58	34.67	99	6	FAN	REST3	28	50
	698	37.66	37.56	34.64	99	6	FAN	REST3	28	50
	699	37.66	37.57	34.70	97	6	FAN	REST3	28	50
	700	37.67	37.59	34.81	95	6	FAN	REST3	28	50
	701	37.68	37.59	34.85	99	6	FAN	REST3	28	50
	702	37.69	37.57	34.88	99	6	FAN	REST3	28	50
	703	37.69	37.57	34.91	99	6	FAN	REST3	28	50
	704	37.70	37.57	34.95	98	6	FAN	REST3	28	50
	705	37.70	37.57	34.98	93	6	FAN	REST3	28	50
	706	37.70	37.57	34.98	93	6	FAN	REST3	28	50
	707	37.70	37.55	34.99	90	6	FAN	REST3	28	50
	708	37.70	37.54	34.99	91	6	FAN	REST3	28	50
	709	37.70	37.47	34.99	92	6	FAN	REST3	28	50
0	1	37.05	36.89	35.34	83	7	FAN	REST1	28	50
	2	37.06	36.92	35.35	80	7	FAN	REST1	28	50
	3	37.08	36.93	35.36	79	7	FAN	REST1	28	50
	4	37.08	36.91	35.36	76	7	FAN	REST1	28	50

min	number	Tre	Tes	Tsk-head	HR	ID	condition	period	Ta	RH
	5	37.08	36.92	35.34	81	7	FAN	REST1	28	50
	6	37.09	36.92	35.32	83	7	FAN	REST1	28	50
	7	37.09	36.90	35.31	82	7	FAN	REST1	28	50
	8	37.08	36.90	35.31	78	7	FAN	REST1	28	50
	9	37.07	36.90	35.32	79	7	FAN	REST1	28	50
	10	37.07	36.91	35.33	80	7	FAN	REST1	28	50
	11	37.06	36.91	35.33	81	7	FAN	REST1	28	50
	12	37.06	36.90	35.33	83	7	FAN	REST1	28	50
	13	37.06	36.89	35.34	81	7	FAN	REST1	28	50
	14	37.06	36.90	35.35	80	7	FAN	REST1	28	50
	15	37.06	36.90	35.34	79	7	FAN	REST1	28	50
	16	37.06	36.88	35.33	77	7	FAN	REST1	28	50
	17	37.06	36.87	35.33	80	7	FAN	REST1	28	50
	18	37.05	36.88	35.33	80	7	FAN	REST1	28	50
	19	37.05	36.89	35.34	83	7	FAN	REST1	28	50
	20	37.05	36.88	35.33	80	7	FAN	REST1	28	50
	21	37.05	36.87	35.30	77	7	FAN	REST1	28	50
	22	37.05	36.89	35.30	76	7	FAN	REST1	28	50
	23	37.07	36.90	35.31	82	7	FAN	REST1	28	50
	24	37.10	36.88	35.31	77	7	FAN	REST1	28	50
	25	37.13	36.86	35.32	79	7	FAN	REST1	28	50
	26	37.15	36.85	35.31	80	7	FAN	REST1	28	50
	27	37.15	36.84	35.30	79	7	FAN	REST1	28	50
	28	37.15	36.84	35.30	79	7	FAN	REST1	28	50
	29	37.15	36.85	35.31	81	7	FAN	REST1	28	50
	30	37.15	36.87	35.32	81	7	FAN	REST1	28	50
5	31	37.15	36.88	35.32	78	7	FAN	REST1	28	50
	32	37.15	36.87	35.31	76	7	FAN	REST1	28	50
	33	37.15	36.86	35.30	76	7	FAN	REST1	28	50
	34	37.15	36.86	35.31	79	7	FAN	REST1	28	50
	35	37.15	36.86	35.31	81	7	FAN	REST1	28	50
	36	37.15	36.83	35.31	81	7	FAN	REST1	28	50
	37	37.14	36.81	35.32	79	7	FAN	REST1	28	50
	38	37.14	36.82	35.32	75	7	FAN	REST1	28	50
	39	37.14	36.84	35.31	78	7	FAN	REST1	28	50
	40	37.14	36.84	35.31	81	7	FAN	REST1	28	50
	41	37.14	36.84	35.30	87	7	FAN	REST1	28	50
	42	37.14	36.85	35.30	78	7	FAN	REST1	28	50
	43	37.14	36.85	35.29	78	7	FAN	REST1	28	50
	44	37.14	36.84	35.29	80	7	FAN	REST1	28	50
	45	37.13	36.83	35.31	78	7	FAN	REST1	28	50
	46	37.13	36.85	35.31	79	7	FAN	REST1	28	50
	47	37.13	36.84	35.30	82	7	FAN	REST1	28	50
	48	37.13	36.83	35.31	79	7	FAN	REST1	28	50
	49	37.14	36.83	35.35	76	7	FAN	REST1	28	50
	50	37.13	36.83	35.37	83	7	FAN	REST1	28	50

min	number	Tre	Tes	Tsk-head	HR	ID	condition	period	Ta	RH
	51	37.13	36.84	35.37	82	7	FAN	REST1	28	50
	52	37.13	36.84	35.36	80	7	FAN	REST1	28	50
	53	37.13	36.84	35.38	81	7	FAN	REST1	28	50
	54	37.12	36.85	35.37	73	7	FAN	REST1	28	50
	55	37.12	36.85	35.36	77	7	FAN	REST1	28	50
	56	37.13	36.84	35.37	78	7	FAN	REST1	28	50
	57	37.12	36.83	35.36	75	7	FAN	REST1	28	50
	58	37.11	36.84	35.35	77	7	FAN	REST1	28	50
	59	37.11	36.85	35.35	76	7	FAN	REST1	28	50
	60	37.11	36.83	35.34	78	7	FAN	REST1	28	50
10	61	37.12	36.84	35.34	77	7	FAN	REST1	28	50
	62	37.12	36.85	35.32	74	7	FAN	REST1	28	50
	63	37.12	36.83	35.32	75	7	FAN	REST1	28	50
	64	37.12	36.83	35.33	75	7	FAN	REST1	28	50
	65	37.12	36.84	35.32	71	7	FAN	REST1	28	50
	66	37.11	36.82	35.29	72	7	FAN	REST1	28	50
	67	37.11	36.82	35.29	71	7	FAN	REST1	28	50
	68	37.12	36.84	35.31	78	7	FAN	REST1	28	50
	69	37.12	36.86	35.32	79	7	FAN	REST1	28	50
	70	37.12	36.85	35.33	78	7	FAN	REST1	28	50
	71	37.12	36.82	35.32	79	7	FAN	REST1	28	50
	72	37.12	36.82	35.32	80	7	FAN	REST1	28	50
	73	37.12	36.83	35.32	80	7	FAN	REST1	28	50
	74	37.11	36.85	35.33	76	7	FAN	REST1	28	50
	75	37.11	36.85	35.34	77	7	FAN	REST1	28	50
	76	37.11	36.82	35.33	80	7	FAN	REST1	28	50
	77	37.10	36.83	35.35	79	7	FAN	REST1	28	50
	78	37.11	36.86	35.35	81	7	FAN	REST1	28	50
	79	37.11	36.87	35.34	84	7	FAN	REST1	28	50
	80	37.11	36.86	35.31	85	7	FAN	REST1	28	50
	81	37.11	36.84	35.31	86	7	FAN	REST1	28	50
	82	37.11	36.75	35.31	97	7	FAN	REST1	28	50
	83	37.11	36.72	35.31	80	7	FAN	REST1	28	50
	84	37.11	36.76	35.30	89	7	FAN	REST1	28	50
	85	37.10	36.77	35.31	81	7	FAN	REST1	28	50
	86	37.10	36.84	35.33	79	7	FAN	REST1	28	50
	87	37.10	36.89	35.33	77	7	FAN	REST1	28	50
	88	37.10	36.84	35.33	83	7	FAN	REST1	28	50
	89	37.11	36.80	35.32	77	7	FAN	REST1	28	50
	90	37.10	36.80	35.31	83	7	FAN	REST1	28	50
15	91	37.11	36.81	35.32	82	7	FAN	REST1	28	50
	92	37.11	36.81	35.33	81	7	FAN	REST1	28	50
	93	37.08	36.82	35.33	89	7	FAN	REST1	28	50
	94	37.04	36.81	35.31	96	7	FAN	REST1	28	50
	95	37.01	36.74	35.29	90	7	FAN	REST1	28	50
	96	36.99	36.73	35.29	86	7	FAN	REST1	28	50

min	number	Tre	Tes	Tsk-head	HR	ID	condition	period	Ta	RH
	97	36.99	36.77	35.31	77	7	FAN	REST1	28	50
	98	37.00	36.75	35.30	79	7	FAN	REST1	28	50
	99	37.00	36.77	35.30	75	7	FAN	REST1	28	50
	100	37.00	36.78	35.33	77	7	FAN	REST1	28	50
	101	37.00	36.77	35.34	78	7	FAN	REST1	28	50
	102	37.01	36.78	35.33	80	7	FAN	REST1	28	50
	103	37.01	36.77	35.36	81	7	FAN	REST1	40	50
	104	36.99	36.76	35.59	100	7	FAN	REST1	40	50
	105	36.96	36.76	35.89	93	7	FAN	REST1	40	50
	106	36.96	36.73	36.03	80	7	FAN	REST1	40	50
	107	36.96	36.70	36.10	80	7	FAN	REST1	40	50
	108	36.96	36.71	36.15	83	7	FAN	REST1	40	50
	109	36.97	36.72	36.19	78	7	FAN	REST1	40	50
	110	36.96	36.71	36.26	93	7	FAN	REST1	40	50
	111	36.92	36.68	36.30	90	7	FAN	REST1	40	50
	112	36.89	36.67	36.33	88	7	FAN	REST1	40	50
	113	36.88	36.66	36.37	88	7	FAN	REST1	40	50
	114	36.87	36.65	36.40	93	7	FAN	REST1	40	50
	115	36.87	36.67	36.43	87	7	FAN	REST1	40	50
	116	36.88	36.68	36.46	91	7	FAN	REST1	40	50
	117	36.89	36.62	36.48	86	7	FAN	REST1	40	50
	118	36.89	36.57	36.49	84	7	FAN	REST1	40	50
	119	36.87	36.58	36.52	90	7	FAN	REST1	40	50
	120	36.86	36.60	36.54	83	7	FAN	REST1	40	50
20	121	36.86	36.64	36.57	82	7	FAN	REST1	40	50
	122	36.86	36.65	36.59	89	7	FAN	REST1	40	50
	123	36.85	36.64	36.60	82	7	FAN	REST1	40	50
	124	36.85	36.66	36.63	85	7	FAN	REST1	40	50
	125	36.86	36.68	36.64	91	7	FAN	REST1	40	50
	126	36.86	36.67	36.65	88	7	FAN	REST1	40	50
	127	36.86	36.66	36.68	91	7	FAN	REST1	40	50
	128	36.86	36.68	36.70	88	7	FAN	REST1	40	50
	129	36.85	36.68	36.72	87	7	FAN	REST1	40	50
	130	36.85	36.68	36.73	90	7	FAN	REST1	40	50
	131	36.86	36.71	36.74	94	7	FAN	REST1	40	50
	132	36.86	36.73	36.76	95	7	FAN	REST1	40	50
	133	36.86	36.73	36.77	89	7	FAN	REST1	40	50
	134	36.87	36.71	36.78	105	7	FAN	REST1	40	50
	135	36.91	36.70	36.82	99	7	FAN	REST1	40	50
	136	36.94	36.72	36.83	93	7	FAN	REST1	40	50
	137	36.96	36.71	36.83	88	7	FAN	REST1	40	50
	138	36.99	36.70	36.83	90	7	FAN	REST1	40	50
	139	37.00	36.71	36.84	78	7	FAN	EXERCISE1	40	50
	140	37.00	36.71	36.86	94	7	FAN	EXERCISE1	40	50
	141	37.00	36.71	36.87	95	7	FAN	EXERCISE1	40	50
	142	37.00	36.71	36.89	100	7	FAN	EXERCISE1	40	50

min	number	Tre	Tes	Tsk-head	HR	ID	condition	period	Ta	RH
	143	37.00	36.72	36.91	102	7	FAN	EXERCISE1	40	50
	144	37.00	36.72	36.92	104	7	FAN	EXERCISE1	40	50
	145	37.00	36.74	36.91	99	7	FAN	EXERCISE1	40	50
	146	37.00	36.75	36.91	105	7	FAN	EXERCISE1	40	50
	147	37.00	36.72	36.93	107	7	FAN	EXERCISE1	40	50
	148	37.00	36.68	36.94	107	7	FAN	EXERCISE1	40	50
	149	37.00	36.67	36.95	105	7	FAN	EXERCISE1	40	50
	150	37.00	36.68	36.96	106	7	FAN	EXERCISE1	40	50
25	151	37.00	36.68	36.97	106	7	FAN	EXERCISE1	40	50
	152	37.00	36.71	36.98	105	7	FAN	EXERCISE1	40	50
	153	37.00	36.71	36.98	108	7	FAN	EXERCISE1	40	50
	154	37.01	36.69	36.99	106	7	FAN	EXERCISE1	40	50
	155	37.01	36.69	37.00	106	7	FAN	EXERCISE1	40	50
	156	37.00	36.70	37.00	106	7	FAN	EXERCISE1	40	50
	157	37.00	36.70	37.02	108	7	FAN	EXERCISE1	40	50
	158	37.00	36.73	37.04	109	7	FAN	EXERCISE1	40	50
	159	37.01	36.74	37.04	108	7	FAN	EXERCISE1	40	50
	160	37.02	36.75	37.04	109	7	FAN	EXERCISE1	40	50
	161	37.02	36.78	37.06	106	7	FAN	EXERCISE1	40	50
	162	37.01	36.77	37.07	110	7	FAN	EXERCISE1	40	50
	163	37.01	36.77	37.07	109	7	FAN	EXERCISE1	40	50
	164	37.00	36.77	37.07	112	7	FAN	EXERCISE1	40	50
	165	37.01	36.79	37.09	110	7	FAN	EXERCISE1	40	50
	166	37.02	36.81	37.10	109	7	FAN	EXERCISE1	40	50
	167	37.02	36.80	37.11	110	7	FAN	EXERCISE1	40	50
	168	37.02	36.81	37.12	111	7	FAN	EXERCISE1	40	50
	169	37.01	36.82	37.13	110	7	FAN	EXERCISE1	40	50
	170	37.01	36.82	37.13	111	7	FAN	EXERCISE1	40	50
	171	37.02	36.84	37.14	110	7	FAN	EXERCISE1	40	50
	172	37.03	36.85	37.14	109	7	FAN	EXERCISE1	40	50
	173	37.03	36.88	37.15	110	7	FAN	EXERCISE1	40	50
	174	37.03	36.88	37.16	112	7	FAN	EXERCISE1	40	50
	175	37.03	36.89	37.17	111	7	FAN	EXERCISE1	40	50
	176	37.03	36.89	37.18	110	7	FAN	EXERCISE1	40	50
	177	37.03	36.90	37.18	111	7	FAN	EXERCISE1	40	50
	178	37.03	36.91	37.18	110	7	FAN	EXERCISE1	40	50
	179	37.03	36.91	37.19	112	7	FAN	EXERCISE1	40	50
	180	37.03	36.92	37.20	111	7	FAN	EXERCISE1	40	50
30	181	37.04	36.92	37.20	112	7	FAN	EXERCISE1	40	50
	182	37.04	36.94	37.22	112	7	FAN	EXERCISE1	40	50
	183	37.04	36.95	37.22	110	7	FAN	EXERCISE1	40	50
	184	37.04	36.94	37.22	109	7	FAN	EXERCISE1	40	50
	185	37.04	36.93	37.22	110	7	FAN	EXERCISE1	40	50
	186	37.03	36.95	37.22	113	7	FAN	EXERCISE1	40	50
	187	37.03	36.96	37.23	113	7	FAN	EXERCISE1	40	50
	188	37.04	36.96	37.22	114	7	FAN	EXERCISE1	40	50

min	number	Tre	Tes	Tsk-head	HR	ID	condition	period	Ta	RH
	189	37.04	36.98	37.22	112	7	FAN	EXERCISE1	40	50
	190	37.04	36.99	37.22	109	7	FAN	EXERCISE1	40	50
	191	37.04	36.99	37.22	112	7	FAN	EXERCISE1	40	50
	192	37.05	36.99	37.22	111	7	FAN	EXERCISE1	40	50
	193	37.05	36.99	37.21	114	7	FAN	EXERCISE1	40	50
	194	37.05	36.99	37.21	112	7	FAN	EXERCISE1	40	50
	195	37.05	36.98	37.22	111	7	FAN	EXERCISE1	40	50
	196	37.06	36.90	37.22	116	7	FAN	EXERCISE1	40	50
	197	37.06	36.86	37.21	116	7	FAN	EXERCISE1	40	50
	198	37.06	36.91	37.20	111	7	FAN	EXERCISE1	40	50
	199	37.07	36.93	37.20	112	7	FAN	EXERCISE1	40	50
	200	37.07	36.95	37.21	114	7	FAN	EXERCISE1	40	50
	201	37.08	36.95	37.21	112	7	FAN	EXERCISE1	40	50
	202	37.08	36.95	37.21	111	7	FAN	EXERCISE1	40	50
	203	37.08	36.97	37.22	116	7	FAN	EXERCISE1	40	50
	204	37.08	36.99	37.23	113	7	FAN	EXERCISE1	40	50
	205	37.08	36.99	37.23	115	7	FAN	EXERCISE1	40	50
	206	37.09	36.98	37.24	114	7	FAN	EXERCISE1	40	50
	207	37.09	36.97	37.25	114	7	FAN	EXERCISE1	40	50
	208	37.08	36.99	37.25	112	7	FAN	EXERCISE1	40	50
	209	37.09	37.00	37.24	112	7	FAN	EXERCISE1	40	50
	210	37.09	37.01	37.23	114	7	FAN	EXERCISE1	40	50
35	211	37.09	37.01	37.23	112	7	FAN	EXERCISE1	40	50
	212	37.09	37.01	37.23	113	7	FAN	EXERCISE1	40	50
	213	37.08	37.01	37.22	112	7	FAN	EXERCISE1	40	50
	214	37.09	37.00	37.21	114	7	FAN	EXERCISE1	40	50
	215	37.10	37.02	37.21	108	7	FAN	EXERCISE1	40	50
	216	37.11	37.02	37.21	113	7	FAN	EXERCISE1	40	50
	217	37.11	37.01	37.20	114	7	FAN	EXERCISE1	40	50
	218	37.11	37.04	37.21	112	7	FAN	EXERCISE1	40	50
	219	37.11	37.04	37.21	113	7	FAN	EXERCISE1	40	50
	220	37.10	37.03	37.20	115	7	FAN	EXERCISE1	40	50
	221	37.10	37.04	37.19	114	7	FAN	EXERCISE1	40	50
	222	37.10	37.04	37.20	113	7	FAN	EXERCISE1	40	50
	223	37.11	37.04	37.20	114	7	FAN	EXERCISE1	40	50
	224	37.11	37.06	37.20	113	7	FAN	EXERCISE1	40	50
	225	37.12	37.08	37.22	112	7	FAN	EXERCISE1	40	50
	226	37.12	37.06	37.22	113	7	FAN	EXERCISE1	40	50
	227	37.12	37.05	37.21	116	7	FAN	EXERCISE1	40	50
	228	37.12	37.05	37.20	117	7	FAN	EXERCISE1	40	50
	229	37.12	37.06	37.20	115	7	FAN	EXERCISE1	40	50
	230	37.13	37.07	37.21	115	7	FAN	EXERCISE1	40	50
	231	37.14	37.07	37.21	113	7	FAN	EXERCISE1	40	50
	232	37.14	37.07	37.20	113	7	FAN	EXERCISE1	40	50
	233	37.13	37.06	37.20	114	7	FAN	EXERCISE1	40	50
	234	37.13	37.05	37.20	114	7	FAN	EXERCISE1	40	50

min	number	Tre	Tes	Tsk-head	HR	ID	condition	period	Ta	RH
	235	37.13	37.05	37.20	114	7	FAN	EXERCISE1	40	50
	236	37.13	37.06	37.20	114	7	FAN	EXERCISE1	40	50
	237	37.13	37.08	37.20	114	7	FAN	EXERCISE1	40	50
	238	37.14	37.08	37.20	117	7	FAN	EXERCISE1	40	50
	239	37.15	37.07	37.21	118	7	FAN	EXERCISE1	40	50
	240	37.14	37.08	37.20	115	7	FAN	EXERCISE1	40	50
40	241	37.14	37.09	37.19	116	7	FAN	EXERCISE1	40	50
	242	37.15	37.07	37.17	118	7	FAN	EXERCISE1	40	50
	243	37.16	37.08	37.16	115	7	FAN	EXERCISE1	40	50
	244	37.16	37.00	37.16	118	7	FAN	EXERCISE1	40	50
	245	37.15	36.96	37.14	114	7	FAN	EXERCISE1	40	50
	246	37.15	37.04	37.16	115	7	FAN	EXERCISE1	40	50
	247	37.16	37.04	37.17	117	7	FAN	EXERCISE1	40	50
	248	37.17	37.06	37.16	116	7	FAN	EXERCISE1	40	50
	249	37.17	37.07	37.17	114	7	FAN	EXERCISE1	40	50
	250	37.17	37.07	37.16	113	7	FAN	EXERCISE1	40	50
	251	37.17	37.08	37.16	114	7	FAN	EXERCISE1	40	50
	252	37.17	37.07	37.16	116	7	FAN	EXERCISE1	40	50
	253	37.16	37.08	37.16	115	7	FAN	EXERCISE1	40	50
	254	37.16	37.10	37.18	114	7	FAN	EXERCISE1	40	50
	255	37.17	37.11	37.18	115	7	FAN	EXERCISE1	40	50
	256	37.16	37.11	37.15	116	7	FAN	EXERCISE1	40	50
	257	37.16	37.11	37.13	117	7	FAN	EXERCISE1	40	50
	258	37.16	37.12	37.11	116	7	FAN	EXERCISE1	40	50
	259	37.16	37.11	37.11	114	7	FAN	EXERCISE1	40	50
	260	37.17	37.10	37.11	113	7	FAN	EXERCISE1	40	50
	261	37.17	37.10	37.11	115	7	FAN	EXERCISE1	40	50
	262	37.18	37.09	37.11	120	7	FAN	EXERCISE1	40	50
	263	37.19	37.01	37.09	118	7	FAN	EXERCISE1	40	50
	264	37.19	36.98	37.09	113	7	FAN	EXERCISE1	40	50
	265	37.19	37.04	37.10	112	7	FAN	EXERCISE1	40	50
	266	37.19	37.05	37.10	114	7	FAN	EXERCISE1	40	50
	267	37.19	37.07	37.09	117	7	FAN	EXERCISE1	40	50
	268	37.21	37.10	37.09	114	7	FAN	EXERCISE1	40	50
	269	37.22	37.11	37.09	115	7	FAN	EXERCISE1	40	50
	270	37.21	37.11	37.09	115	7	FAN	EXERCISE1	40	50
45	271	37.21	37.12	37.08	116	7	FAN	EXERCISE1	40	50
	272	37.22	37.13	37.08	114	7	FAN	EXERCISE1	40	50
	273	37.21	37.11	37.07	112	7	FAN	EXERCISE1	40	50
	274	37.22	37.11	37.06	113	7	FAN	EXERCISE1	40	50
	275	37.22	37.14	37.05	114	7	FAN	EXERCISE1	40	50
	276	37.22	37.13	37.05	117	7	FAN	EXERCISE1	40	50
	277	37.23	37.13	37.06	115	7	FAN	EXERCISE1	40	50
	278	37.23	37.14	37.07	118	7	FAN	EXERCISE1	40	50
	279	37.23	37.14	37.06	116	7	FAN	EXERCISE1	40	50
	280	37.24	37.15	37.06	117	7	FAN	EXERCISE1	40	50

min	number	Tre	Tes	Tsk-head	HR	ID	condition	period	Ta	RH
	281	37.25	37.17	37.07	116	7	FAN	EXERCISE1	40	50
	282	37.25	37.15	37.06	116	7	FAN	EXERCISE1	40	50
	283	37.25	37.14	37.05	117	7	FAN	EXERCISE1	40	50
	284	37.25	37.16	37.06	113	7	FAN	EXERCISE1	40	50
	285	37.25	37.17	37.07	115	7	FAN	EXERCISE1	40	50
	286	37.25	37.16	37.06	118	7	FAN	EXERCISE1	40	50
	287	37.25	37.15	37.06	119	7	FAN	EXERCISE1	40	50
	288	37.26	37.17	37.07	121	7	FAN	EXERCISE1	40	50
	289	37.26	37.17	37.07	123	7	FAN	EXERCISE1	40	50
	290	37.25	37.09	37.06	119	7	FAN	EXERCISE1	40	50
	291	37.25	37.06	37.06	117	7	FAN	EXERCISE1	40	50
	292	37.26	37.12	37.06	116	7	FAN	EXERCISE1	40	50
	293	37.27	37.15	37.06	119	7	FAN	EXERCISE1	40	50
	294	37.27	37.15	37.05	117	7	FAN	EXERCISE1	40	50
	295	37.27	37.14	37.05	119	7	FAN	EXERCISE1	40	50
	296	37.27	37.16	37.06	118	7	FAN	EXERCISE1	40	50
	297	37.28	37.19	37.09	118	7	FAN	EXERCISE1	40	50
	298	37.27	37.19	37.09	118	7	FAN	EXERCISE1	40	50
	299	37.27	37.18	37.05	118	7	FAN	EXERCISE1	40	50
	300	37.27	37.19	37.05	118	7	FAN	EXERCISE1	40	50
50	301	37.28	37.20	37.02	120	7	FAN	EXERCISE1	40	50
	302	37.28	37.19	37.01	121	7	FAN	EXERCISE1	40	50
	303	37.29	37.20	37.03	118	7	FAN	EXERCISE1	40	50
	304	37.29	37.19	37.02	119	7	FAN	EXERCISE1	40	50
	305	37.29	37.18	36.98	122	7	FAN	EXERCISE1	40	50
	306	37.29	37.20	36.97	121	7	FAN	EXERCISE1	40	50
	307	37.29	37.21	36.94	120	7	FAN	EXERCISE1	40	50
	308	37.29	37.22	36.93	119	7	FAN	EXERCISE1	40	50
	309	37.30	37.23	36.94	119	7	FAN	EXERCISE1	40	50
	310	37.31	37.22	36.94	119	7	FAN	EXERCISE1	40	50
	311	37.31	37.13	36.94	119	7	FAN	EXERCISE1	40	50
	312	37.30	37.07	36.92	117	7	FAN	EXERCISE1	40	50
	313	37.30	37.13	36.91	116	7	FAN	EXERCISE1	40	50
	314	37.31	37.17	36.91	120	7	FAN	EXERCISE1	40	50
	315	37.32	37.18	36.90	116	7	FAN	EXERCISE1	40	50
	316	37.32	37.19	36.90	113	7	FAN	EXERCISE1	40	50
	317	37.32	37.21	36.89	116	7	FAN	EXERCISE1	40	50
	318	37.33	37.22	36.88	119	7	FAN	EXERCISE1	40	50
	319	37.33	37.20	36.86	116	7	FAN	EXERCISE1	40	50
	320	37.33	37.21	36.88	116	7	FAN	REST2	28	50
	321	37.33	37.19	36.88	120	7	FAN	REST2	28	50
	322	37.31	37.19	36.77	119	7	FAN	REST2	28	50
	323	37.31	37.22	36.55	92	7	FAN	REST2	28	50
	324	37.32	37.23	36.47	109	7	FAN	REST2	28	50
	325	37.33	37.24	36.54	101	7	FAN	REST2	28	50
	326	37.34	37.25	36.59	104	7	FAN	REST2	28	50

min	number	Tre	Tes	Tsk-head	HR	ID	condition	period	Ta	RH
	327	37.34	37.26	36.59	102	7	FAN	REST2	28	50
	328	37.35	37.26	36.57	98	7	FAN	REST2	28	50
	329	37.36	37.26	36.55	93	7	FAN	REST2	28	50
	330	37.37	37.27	36.54	93	7	FAN	REST2	28	50
55	331	37.38	37.26	36.52	103	7	FAN	REST2	28	50
	332	37.38	37.26	36.45	89	7	FAN	REST2	28	50
	333	37.38	37.26	36.40	93	7	FAN	REST2	28	50
	334	37.39	37.24	36.40	93	7	FAN	REST2	28	50
	335	37.39	37.23	36.39	94	7	FAN	REST2	28	50
	336	37.40	37.23	36.38	87	7	FAN	REST2	28	50
	337	37.40	37.19	36.38	96	7	FAN	REST2	28	50
	338	37.41	37.16	36.36	96	7	FAN	REST2	28	50
	339	37.41	32.57	36.33	102	7	FAN	REST2	28	50
	340	37.41	28.56	36.32	102	7	FAN	REST2	28	50
	341	37.41	30.39	36.29	100	7	FAN	REST2	28	50
	342	37.41	30.07	36.27	100	7	FAN	REST2	28	50
	343	37.41	28.33	36.26	101	7	FAN	REST2	28	50
	344	37.42	28.52	36.25	99	7	FAN	REST2	28	50
	345	37.42	28.56	36.25	85	7	FAN	REST2	28	50
	346	37.42	28.86	36.23	101	7	FAN	REST2	28	50
	347	37.42	28.65	36.20	93	7	FAN	REST2	28	50
	348	37.41	29.10	36.19	93	7	FAN	REST2	28	50
	349	37.42	30.84	36.17	93	7	FAN	REST2	28	50
	350	37.42	31.68	36.16	80	7	FAN	REST2	28	50
	351	37.41	32.27	36.17	74	7	FAN	REST2	28	50
	352	37.41	32.96	36.15	90	7	FAN	REST2	28	50
	353	37.41	33.72	36.13	75	7	FAN	REST2	28	50
	354	37.40	34.22	36.11	85	7	FAN	REST2	28	50
	355	37.40	34.72	36.08	80	7	FAN	REST2	28	50
	356	37.41	35.15	36.04	79	7	FAN	REST2	28	50
	357	37.41	35.39	36.02	73	7	FAN	REST2	28	50
	358	37.40	35.55	35.99	79	7	FAN	REST2	28	50
	359	37.40	35.70	35.97	80	7	FAN	REST2	28	50
	360	37.40	35.77	35.97	83	7	FAN	REST2	28	50
60	361	37.40	35.86	35.96	82	7	FAN	REST2	28	50
	362	37.40	35.99	35.94	81	7	FAN	REST2	28	50
	363	37.40	36.03	35.91	94	7	FAN	REST2	28	50
	364	37.41	31.86	35.89	102	7	FAN	REST2	28	50
	365	37.42	27.39	35.88	90	7	FAN	REST2	28	50
	366	37.42	27.65	35.88	80	7	FAN	REST2	28	50
	367	37.43	27.98	35.90	80	7	FAN	REST2	28	50
	368	37.43	29.39	35.91	87	7	FAN	REST2	28	50
	369	37.43	29.13	35.91	88	7	FAN	REST2	28	50
	370	37.43	29.13	35.90	83	7	FAN	REST2	28	50
	371	37.42	29.91	35.89	88	7	FAN	REST2	28	50
	372	37.42	28.55	35.90	98	7	FAN	REST2	28	50

min	number	Tre	Tes	Tsk-head	HR	ID	condition	period	Ta	RH
	373	37.42	29.82	35.88	91	7	FAN	REST2	28	50
	374	37.41	31.59	35.83	77	7	FAN	REST2	28	50
	375	37.40	32.26	35.82	72	7	FAN	REST2	28	50
	376	37.39	32.71	35.78	73	7	FAN	REST2	28	50
	377	37.39	33.10	35.75	75	7	FAN	REST2	28	50
	378	37.39	33.50	35.75	84	7	FAN	REST2	28	50
	379	37.39	33.79	35.74	71	7	FAN	REST2	28	50
	380	37.40	34.22	35.74	75	7	FAN	REST2	28	50
	381	37.40	34.51	35.72	94	7	FAN	REST2	28	50
	382	37.41	34.03	35.70	78	7	FAN	REST2	28	50
	383	37.41	33.70	35.69	77	7	FAN	REST2	28	50
	384	37.41	34.50	35.67	80	7	FAN	REST2	28	50
	385	37.41	35.09	35.66	77	7	FAN	REST2	28	50
	386	37.42	35.22	35.67	74	7	FAN	REST2	28	50
	387	37.42	35.40	35.65	76	7	FAN	REST2	28	50
	388	37.41	35.49	35.61	83	7	FAN	REST2	28	50
	389	37.41	35.64	35.60	81	7	FAN	REST2	28	50
	390	37.41	35.81	35.60	79	7	FAN	REST2	28	50
65	391	37.39	35.97	35.60	83	7	FAN	REST2	28	50
	392	37.39	36.11	35.61	76	7	FAN	REST2	28	50
	393	37.38	36.05	35.62	74	7	FAN	REST2	28	50
	394	37.38	35.97	35.62	73	7	FAN	REST2	28	50
	395	37.38	36.16	35.62	73	7	FAN	REST2	28	50
	396	37.37	36.36	35.61	71	7	FAN	REST2	28	50
	397	37.36	36.37	35.59	78	7	FAN	REST2	28	50
	398	37.36	36.45	35.57	81	7	FAN	REST2	28	50
	399	37.37	36.55	35.51	78	7	FAN	REST2	28	50
	400	37.37	36.61	35.43	82	7	FAN	REST2	28	50
	401	37.37	36.60	35.41	87	7	FAN	REST2	28	50
	402	37.37	36.57	35.46	80	7	FAN	REST2	28	50
	403	37.36	36.60	35.52	74	7	FAN	REST2	28	50
	404	37.35	36.64	35.56	75	7	FAN	REST2	28	50
	405	37.36	36.67	35.57	76	7	FAN	REST2	28	50
	406	37.37	36.63	35.52	81	7	FAN	REST2	28	50
	407	37.37	36.66	35.50	78	7	FAN	REST2	28	50
	408	37.36	36.78	35.52	75	7	FAN	REST2	28	50
	409	37.35	36.83	35.51	78	7	FAN	REST2	28	50
	410	37.35	36.80	35.50	77	7	FAN	REST2	28	50
	411	37.36	36.81	35.51	75	7	FAN	REST2	28	50
	412	37.36	36.85	35.51	72	7	FAN	REST2	28	50
	413	37.36	36.85	35.50	80	7	FAN	REST2	28	50
	414	37.36	36.88	35.53	79	7	FAN	REST2	28	50
	415	37.36	36.88	35.54	84	7	FAN	REST2	28	50
	416	37.36	36.87	35.52	79	7	FAN	REST2	28	50
	417	37.36	36.87	35.50	76	7	FAN	REST2	28	50
	418	37.35	36.82	35.50	75	7	FAN	REST2	28	50

min	number	Tre	Tes	Tsk-head	HR	ID	condition	period	Ta	RH
	419	37.35	36.79	35.50	76	7	FAN	REST2	28	50
	420	37.36	36.80	35.68	97	7	FAN	REST2	28	50
70	421	37.37	36.83	35.62	81	7	FAN	REST2	28	50
	422	37.37	36.87	35.32	84	7	FAN	REST2	28	50
	423	37.37	36.88	35.24	93	7	FAN	REST2	28	50
	424	37.36	36.89	35.20	96	7	FAN	REST2	28	50
	425	37.36	36.91	35.20	90	7	FAN	REST2	28	50
	426	37.36	36.91	35.22	80	7	FAN	REST2	28	50
	427	37.36	36.90	35.23	86	7	FAN	REST2	28	50
	428	37.37	36.92	35.24	90	7	FAN	REST2	28	50
	429	37.37	36.92	35.25	85	7	FAN	REST2	28	50
	430	37.36	36.92	35.27	89	7	FAN	REST2	28	50
	431	37.36	36.93	35.28	77	7	FAN	REST2	28	50
	432	37.36	36.92	35.31	76	7	FAN	REST2	28	50
	433	37.35	36.93	35.36	75	7	FAN	REST2	28	50
	434	37.35	36.92	35.39	72	7	FAN	REST2	28	50
	435	37.34	36.92	35.40	74	7	FAN	REST2	28	50
	436	37.34	36.93	35.42	79	7	FAN	REST2	28	50
	437	37.34	36.95	35.43	82	7	FAN	REST2	28	50
	438	37.35	36.96	35.44	81	7	FAN	REST2	28	50
	439	37.34	36.94	35.45	79	7	FAN	REST2	40	50
	440	37.34	36.93	35.47	100	7	FAN	REST2	40	50
	441	37.33	36.91	35.59	107	7	FAN	REST2	40	50
	442	37.33	36.91	35.73	81	7	FAN	REST2	40	50
	443	37.33	36.93	35.84	95	7	FAN	REST2	40	50
	444	37.33	36.95	35.93	86	7	FAN	REST2	40	50
	445	37.33	36.95	36.00	88	7	FAN	REST2	40	50
	446	37.33	36.95	36.07	75	7	FAN	REST2	40	50
	447	37.32	36.96	36.12	75	7	FAN	REST2	40	50
	448	37.32	36.94	36.17	82	7	FAN	REST2	40	50
	449	37.32	36.93	36.21	81	7	FAN	REST2	40	50
	450	37.32	36.74	36.24	77	7	FAN	REST2	40	50
75	451	37.32	36.63	36.27	78	7	FAN	REST2	40	50
	452	37.31	36.75	36.29	76	7	FAN	REST2	40	50
	453	37.32	36.81	36.33	88	7	FAN	REST2	40	50
	454	37.32	36.84	36.37	80	7	FAN	REST2	40	50
	455	37.32	36.83	36.40	85	7	FAN	REST2	40	50
	456	37.32	36.83	36.43	83	7	FAN	REST2	40	50
	457	37.32	36.86	36.46	81	7	FAN	REST2	40	50
	458	37.32	36.88	36.49	107	7	FAN	REST2	40	50
	459	37.31	36.86	36.50	95	7	FAN	REST2	40	50
	460	37.30	36.84	36.51	96	7	FAN	REST2	40	50
	461	37.30	36.85	36.54	97	7	FAN	REST2	40	50
	462	37.31	36.88	36.58	86	7	FAN	REST2	40	50
	463	37.31	36.87	36.60	83	7	FAN	EXERCISE2	40	50
	464	37.29	36.81	36.62	99	7	FAN	EXERCISE2	40	50

min	number	Tre	Tes	Tsk-head	HR	ID	condition	period	Ta	RH
	465	37.29	36.79	36.64	98	7	FAN	EXERCISE2	40	50
	466	37.30	36.83	36.66	103	7	FAN	EXERCISE2	40	50
	467	37.30	36.86	36.69	109	7	FAN	EXERCISE2	40	50
	468	37.30	36.66	36.70	106	7	FAN	EXERCISE2	40	50
	469	37.30	36.54	36.71	104	7	FAN	EXERCISE2	40	50
	470	37.30	36.70	36.74	107	7	FAN	EXERCISE2	40	50
	471	37.30	36.78	36.75	110	7	FAN	EXERCISE2	40	50
	472	37.30	36.83	36.76	113	7	FAN	EXERCISE2	40	50
	473	37.29	36.87	36.77	112	7	FAN	EXERCISE2	40	50
	474	37.29	36.87	36.78	110	7	FAN	EXERCISE2	40	50
	475	37.29	36.90	36.80	107	7	FAN	EXERCISE2	40	50
	476	37.30	36.94	36.82	106	7	FAN	EXERCISE2	40	50
	477	37.30	36.97	36.84	110	7	FAN	EXERCISE2	40	50
	478	37.30	36.97	36.85	111	7	FAN	EXERCISE2	40	50
	479	37.30	36.97	36.86	113	7	FAN	EXERCISE2	40	50
	480	37.30	36.99	36.88	114	7	FAN	EXERCISE2	40	50
80	481	37.30	37.01	36.89	113	7	FAN	EXERCISE2	40	50
	482	37.30	37.03	36.91	110	7	FAN	EXERCISE2	40	50
	483	37.30	37.04	36.92	108	7	FAN	EXERCISE2	40	50
	484	37.30	37.06	36.94	109	7	FAN	EXERCISE2	40	50
	485	37.29	37.08	36.96	113	7	FAN	EXERCISE2	40	50
	486	37.29	37.09	36.96	113	7	FAN	EXERCISE2	40	50
	487	37.30	37.10	36.98	114	7	FAN	EXERCISE2	40	50
	488	37.30	37.10	36.99	111	7	FAN	EXERCISE2	40	50
	489	37.31	37.11	37.00	112	7	FAN	EXERCISE2	40	50
	490	37.31	37.13	37.02	114	7	FAN	EXERCISE2	40	50
	491	37.30	37.13	37.03	114	7	FAN	EXERCISE2	40	50
	492	37.30	37.14	37.04	113	7	FAN	EXERCISE2	40	50
	493	37.30	37.14	37.04	113	7	FAN	EXERCISE2	40	50
	494	37.30	37.14	37.06	114	7	FAN	EXERCISE2	40	50
	495	37.31	37.15	37.07	115	7	FAN	EXERCISE2	40	50
	496	37.31	37.14	37.07	115	7	FAN	EXERCISE2	40	50
	497	37.32	37.15	37.08	114	7	FAN	EXERCISE2	40	50
	498	37.32	37.18	37.09	117	7	FAN	EXERCISE2	40	50
	499	37.32	37.19	37.10	115	7	FAN	EXERCISE2	40	50
	500	37.32	37.20	37.11	112	7	FAN	EXERCISE2	40	50
	501	37.32	37.21	37.12	115	7	FAN	EXERCISE2	40	50
	502	37.32	37.20	37.12	116	7	FAN	EXERCISE2	40	50
	503	37.33	37.20	37.13	117	7	FAN	EXERCISE2	40	50
	504	37.34	37.19	37.15	116	7	FAN	EXERCISE2	40	50
	505	37.34	37.18	37.15	119	7	FAN	EXERCISE2	40	50
	506	37.34	37.21	37.15	117	7	FAN	EXERCISE2	40	50
	507	37.33	37.21	37.16	114	7	FAN	EXERCISE2	40	50
	508	37.32	37.20	37.15	118	7	FAN	EXERCISE2	40	50
	509	37.33	37.21	37.16	118	7	FAN	EXERCISE2	40	50
	510	37.34	37.21	37.17	119	7	FAN	EXERCISE2	40	50

min	number	Tre	Tes	Tsk-head	HR	ID	condition	period	Ta	RH
85	511	37.34	37.22	37.18	115	7	FAN	EXERCISE2	40	50
	512	37.34	37.21	37.18	120	7	FAN	EXERCISE2	40	50
	513	37.35	37.20	37.17	118	7	FAN	EXERCISE2	40	50
	514	37.35	37.21	37.18	116	7	FAN	EXERCISE2	40	50
	515	37.35	37.23	37.19	115	7	FAN	EXERCISE2	40	50
	516	37.35	37.24	37.19	118	7	FAN	EXERCISE2	40	50
	517	37.35	37.22	37.18	119	7	FAN	EXERCISE2	40	50
	518	37.37	37.23	37.19	120	7	FAN	EXERCISE2	40	50
	519	37.37	37.23	37.19	116	7	FAN	EXERCISE2	40	50
	520	37.37	37.22	37.19	118	7	FAN	EXERCISE2	40	50
	521	37.37	37.23	37.20	114	7	FAN	EXERCISE2	40	50
	522	37.37	37.22	37.15	114	7	FAN	EXERCISE2	40	50
	523	37.37	37.20	37.14	119	7	FAN	EXERCISE2	40	50
	524	37.37	37.15	37.18	118	7	FAN	EXERCISE2	40	50
	525	37.38	37.13	37.18	117	7	FAN	EXERCISE2	40	50
	526	37.38	37.17	37.18	119	7	FAN	EXERCISE2	40	50
	527	37.37	37.19	37.19	116	7	FAN	EXERCISE2	40	50
	528	37.38	37.22	37.20	116	7	FAN	EXERCISE2	40	50
	529	37.39	37.24	37.21	117	7	FAN	EXERCISE2	40	50
	530	37.39	37.23	37.21	115	7	FAN	EXERCISE2	40	50
	531	37.39	37.23	37.20	116	7	FAN	EXERCISE2	40	50
	532	37.39	37.23	37.20	117	7	FAN	EXERCISE2	40	50
	533	37.39	37.24	37.21	118	7	FAN	EXERCISE2	40	50
	534	37.40	37.23	37.20	120	7	FAN	EXERCISE2	40	50
	535	37.40	37.23	37.21	117	7	FAN	EXERCISE2	40	50
	536	37.40	37.25	37.21	118	7	FAN	EXERCISE2	40	50
	537	37.41	37.27	37.21	118	7	FAN	EXERCISE2	40	50
	538	37.42	37.26	37.22	118	7	FAN	EXERCISE2	40	50
	539	37.41	37.24	37.21	118	7	FAN	EXERCISE2	40	50
	540	37.41	37.24	37.21	116	7	FAN	EXERCISE2	40	50
90	541	37.42	37.25	37.22	116	7	FAN	EXERCISE2	40	50
	542	37.42	37.25	37.23	118	7	FAN	EXERCISE2	40	50
	543	37.43	37.26	37.22	119	7	FAN	EXERCISE2	40	50
	544	37.43	37.27	37.23	119	7	FAN	EXERCISE2	40	50
	545	37.43	37.26	37.23	117	7	FAN	EXERCISE2	40	50
	546	37.43	37.26	37.24	120	7	FAN	EXERCISE2	40	50
	547	37.44	37.26	37.24	118	7	FAN	EXERCISE2	40	50
	548	37.44	37.25	37.23	118	7	FAN	EXERCISE2	40	50
	549	37.43	37.27	37.23	115	7	FAN	EXERCISE2	40	50
	550	37.44	37.29	37.24	116	7	FAN	EXERCISE2	40	50
	551	37.44	37.27	37.24	119	7	FAN	EXERCISE2	40	50
	552	37.45	37.26	37.24	120	7	FAN	EXERCISE2	40	50
	553	37.45	37.27	37.24	117	7	FAN	EXERCISE2	40	50
	554	37.45	37.29	37.24	122	7	FAN	EXERCISE2	40	50
	555	37.45	37.29	37.24	122	7	FAN	EXERCISE2	40	50
	556	37.46	37.29	37.23	122	7	FAN	EXERCISE2	40	50

min	number	Tre	Tes	Tsk-head	HR	ID	condition	period	Ta	RH
	557	37.46	37.23	37.24	119	7	FAN	EXERCISE2	40	50
	558	37.46	37.15	37.24	117	7	FAN	EXERCISE2	40	50
	559	37.46	37.15	37.24	117	7	FAN	EXERCISE2	40	50
	560	37.46	37.17	37.25	119	7	FAN	EXERCISE2	40	50
	561	37.47	37.20	37.25	118	7	FAN	EXERCISE2	40	50
	562	37.48	37.24	37.25	116	7	FAN	EXERCISE2	40	50
	563	37.48	37.22	37.25	120	7	FAN	EXERCISE2	40	50
	564	37.48	37.20	37.25	119	7	FAN	EXERCISE2	40	50
	565	37.48	37.23	37.26	119	7	FAN	EXERCISE2	40	50
	566	37.48	37.24	37.26	119	7	FAN	EXERCISE2	40	50
	567	37.48	37.24	37.26	120	7	FAN	EXERCISE2	40	50
	568	37.49	37.25	37.26	118	7	FAN	EXERCISE2	40	50
	569	37.49	37.26	37.26	119	7	FAN	EXERCISE2	40	50
	570	37.49	37.26	37.27	118	7	FAN	EXERCISE2	40	50
95	571	37.50	37.27	37.27	120	7	FAN	EXERCISE2	40	50
	572	37.50	37.28	37.27	119	7	FAN	EXERCISE2	40	50
	573	37.50	37.27	37.27	118	7	FAN	EXERCISE2	40	50
	574	37.51	37.28	37.28	119	7	FAN	EXERCISE2	40	50
	575	37.51	37.30	37.28	118	7	FAN	EXERCISE2	40	50
	576	37.51	37.29	37.29	118	7	FAN	EXERCISE2	40	50
	577	37.51	37.29	37.29	117	7	FAN	EXERCISE2	40	50
	578	37.50	37.29	37.28	118	7	FAN	EXERCISE2	40	50
	579	37.51	37.29	37.28	119	7	FAN	EXERCISE2	40	50
	580	37.52	37.29	37.29	117	7	FAN	EXERCISE2	40	50
	581	37.51	37.15	37.29	124	7	FAN	EXERCISE2	40	50
	582	37.51	37.13	37.30	123	7	FAN	EXERCISE2	40	50
	583	37.51	37.27	37.28	125	7	FAN	EXERCISE2	40	50
	584	37.52	37.27	37.25	122	7	FAN	EXERCISE2	40	50
	585	37.53	37.27	37.26	122	7	FAN	EXERCISE2	40	50
	586	37.53	37.26	37.26	123	7	FAN	EXERCISE2	40	50
	587	37.53	37.27	37.28	119	7	FAN	EXERCISE2	40	50
	588	37.53	37.29	37.29	117	7	FAN	EXERCISE2	40	50
	589	37.53	37.29	37.29	118	7	FAN	EXERCISE2	40	50
	590	37.53	37.31	37.29	120	7	FAN	EXERCISE2	40	50
	591	37.53	37.33	37.29	118	7	FAN	EXERCISE2	40	50
	592	37.54	37.32	37.29	122	7	FAN	EXERCISE2	40	50
	593	37.55	37.31	37.29	120	7	FAN	EXERCISE2	40	50
	594	37.54	37.32	37.28	118	7	FAN	EXERCISE2	40	50
	595	37.54	37.33	37.28	121	7	FAN	EXERCISE2	40	50
	596	37.55	37.33	37.29	120	7	FAN	EXERCISE2	40	50
	597	37.56	37.32	37.29	122	7	FAN	EXERCISE2	40	50
	598	37.56	37.32	37.29	122	7	FAN	EXERCISE2	40	50
	599	37.56	37.32	37.28	124	7	FAN	EXERCISE2	40	50
	600	37.57	37.33	37.28	123	7	FAN	EXERCISE2	40	50
100	601	37.57	37.34	37.29	122	7	FAN	EXERCISE2	40	50
	602	37.57	37.33	37.29	120	7	FAN	EXERCISE2	40	50

min	number	Tre	Tes	Tsk-head	HR	ID	condition	period	Ta	RH
	603	37.57	37.32	37.29	120	7	FAN	EXERCISE2	40	50
	604	37.57	37.34	37.29	121	7	FAN	EXERCISE2	40	50
	605	37.57	37.28	37.28	120	7	FAN	EXERCISE2	40	50
	606	37.57	37.17	37.27	119	7	FAN	EXERCISE2	40	50
	607	37.57	37.19	37.27	118	7	FAN	EXERCISE2	40	50
	608	37.57	37.27	37.27	121	7	FAN	EXERCISE2	40	50
	609	37.58	37.23	37.28	118	7	FAN	EXERCISE2	40	50
	610	37.58	37.21	37.29	120	7	FAN	EXERCISE2	40	50
	611	37.58	37.26	37.28	121	7	FAN	EXERCISE2	40	50
	612	37.59	37.30	37.28	120	7	FAN	EXERCISE2	40	50
	613	37.59	37.31	37.29	122	7	FAN	EXERCISE2	40	50
	614	37.59	37.30	37.28	121	7	FAN	EXERCISE2	40	50
	615	37.59	37.30	37.27	122	7	FAN	EXERCISE2	40	50
	616	37.59	37.31	37.26	122	7	FAN	EXERCISE2	40	50
	617	37.60	37.33	37.26	120	7	FAN	EXERCISE2	40	50
	618	37.60	37.34	37.26	123	7	FAN	EXERCISE2	40	50
	619	37.60	37.33	37.27	122	7	FAN	EXERCISE2	40	50
	620	37.59	37.33	37.28	121	7	FAN	EXERCISE2	40	50
	621	37.60	37.34	37.28	119	7	FAN	EXERCISE2	40	50
	622	37.60	37.33	37.27	124	7	FAN	EXERCISE2	40	50
	623	37.60	37.33	37.27	123	7	FAN	EXERCISE2	40	50
	624	37.61	37.34	37.27	124	7	FAN	EXERCISE2	40	50
	625	37.61	37.31	37.27	120	7	FAN	EXERCISE2	40	50
	626	37.62	37.33	37.28	121	7	FAN	EXERCISE2	40	50
	627	37.62	37.33	37.27	121	7	FAN	EXERCISE2	40	50
	628	37.62	37.33	37.28	121	7	FAN	EXERCISE2	40	50
	629	37.63	37.34	37.28	123	7	FAN	EXERCISE2	40	50
	630	37.62	37.33	37.26	124	7	FAN	EXERCISE2	40	50
105	631	37.62	37.35	37.26	125	7	FAN	EXERCISE2	40	50
	632	37.63	37.37	37.27	125	7	FAN	EXERCISE2	40	50
	633	37.64	37.28	37.28	123	7	FAN	EXERCISE2	40	50
	634	37.64	37.23	37.27	120	7	FAN	EXERCISE2	40	50
	635	37.64	37.29	37.26	121	7	FAN	EXERCISE2	40	50
	636	37.65	37.30	37.27	122	7	FAN	EXERCISE2	40	50
	637	37.65	37.32	37.28	123	7	FAN	EXERCISE2	40	50
	638	37.64	37.34	37.29	126	7	FAN	EXERCISE2	40	50
	639	37.64	37.33	37.28	124	7	FAN	EXERCISE2	40	50
	640	37.64	37.34	37.29	123	7	FAN	EXERCISE2	40	50
	641	37.65	37.36	37.29	119	7	FAN	EXERCISE2	40	50
	642	37.66	37.37	37.28	122	7	FAN	EXERCISE2	40	50
	643	37.65	37.37	37.27	123	7	FAN	EXERCISE2	40	50
	644	37.65	37.35	37.30	126	7	FAN	REST3	28	50
	645	37.65	37.20	37.27	126	7	FAN	REST3	28	50
	646	37.65	37.18	37.16	104	7	FAN	REST3	28	50
	647	37.65	37.34	37.06	101	7	FAN	REST3	28	50
	648	37.66	37.36	36.96	91	7	FAN	REST3	28	50

min	number	Tre	Tes	Tsk-head	HR	ID	condition	period	Ta	RH
	649	37.66	37.38	36.91	91	7	FAN	REST3	28	50
	650	37.66	37.40	36.86	88	7	FAN	REST3	28	50
	651	37.66	37.40	36.82	91	7	FAN	REST3	28	50
	652	37.67	37.39	36.78	93	7	FAN	REST3	28	50
	653	37.67	37.37	36.73	92	7	FAN	REST3	28	50
	654	37.67	37.38	36.68	90	7	FAN	REST3	28	50
	655	37.67	37.38	36.64	90	7	FAN	REST3	28	50
	656	37.67	37.35	36.60	91	7	FAN	REST3	28	50
	657	37.67	37.33	36.56	88	7	FAN	REST3	28	50
	658	37.68	37.32	36.52	90	7	FAN	REST3	28	50
	659	37.67	37.31	36.47	88	7	FAN	REST3	28	50
	660	37.66	37.29	36.44	88	7	FAN	REST3	28	50
110	661	37.66	37.27	36.42	85	7	FAN	REST3	28	50
	662	37.67	37.25	36.39	88	7	FAN	REST3	28	50
	663	37.67	37.24	36.36	90	7	FAN	REST3	28	50
	664	37.67	37.23	36.35	87	7	FAN	REST3	28	50
	665	37.67	37.21	36.34	88	7	FAN	REST3	28	50
	666	37.67	37.21	36.32	88	7	FAN	REST3	28	50
	667	37.66	37.21	36.29	85	7	FAN	REST3	28	50
	668	37.66	37.18	36.27	87	7	FAN	REST3	28	50
	669	37.66	37.16	36.25	82	7	FAN	REST3	28	50
	670	37.65	37.16	36.23	83	7	FAN	REST3	28	50
	671	37.65	37.16	36.21	90	7	FAN	REST3	28	50
	672	37.66	37.11	36.19	91	7	FAN	REST3	28	50
	673	37.67	37.09	36.18	82	7	FAN	REST3	28	50
	674	37.67	37.12	36.18	83	7	FAN	REST3	28	50
	675	37.66	37.13	36.16	83	7	FAN	REST3	28	50
	676	37.65	37.13	36.15	84	7	FAN	REST3	28	50
	677	37.65	37.13	36.13	81	7	FAN	REST3	28	50
	678	37.66	37.13	36.12	81	7	FAN	REST3	28	50
	679	37.66	37.13	36.11	85	7	FAN	REST3	28	50
	680	37.66	37.12	36.10	82	7	FAN	REST3	28	50
	681	37.66	37.04	36.06	102	7	FAN	REST3	28	50
	682	37.67	37.03	36.02	94	7	FAN	REST3	28	50
	683	37.68	37.09	35.98	97	7	FAN	REST3	28	50
	684	37.68	37.11	35.90	113	7	FAN	REST3	28	50
	685	37.68	37.14	35.84	90	7	FAN	REST3	28	50
	686	37.69	37.13	35.82	88	7	FAN	REST3	28	50
	687	37.69	37.12	35.81	91	7	FAN	REST3	28	50
	688	37.69	37.11	35.81	80	7	FAN	REST3	28	50
	689	37.69	37.11	35.82	85	7	FAN	REST3	28	50
	690	37.69	37.13	35.82	94	7	FAN	REST3	28	50
115	691	37.69	37.15	35.81	97	7	FAN	REST3	28	50
	692	37.69	37.18	35.81	89	7	FAN	REST3	28	50
	693	37.69	37.17	35.81	92	7	FAN	REST3	28	50
	694	37.69	36.97	35.79	93	7	FAN	REST3	28	50

min	number	Tre	Tes	Tsk-head	HR	ID	condition	period	Ta	RH
	695	37.69	36.93	35.78	71	7	FAN	REST3	28	50
	696	37.69	37.09	35.79	78	7	FAN	REST3	28	50
	697	37.69	37.12	35.79	83	7	FAN	REST3	28	50
	698	37.68	37.10	35.80	94	7	FAN	REST3	28	50
	699	37.69	37.04	35.80	87	7	FAN	REST3	28	50
	700	37.68	37.04	35.79	85	7	FAN	REST3	28	50
	701	37.68	37.08	35.79	80	7	FAN	REST3	28	50
	702	37.68	37.10	35.79	88	7	FAN	REST3	28	50
	703	37.68	37.13	35.80	84	7	FAN	REST3	28	50
	704	37.68	37.16	35.81	84	7	FAN	REST3	28	50
	705	37.68	37.17	35.83	86	7	FAN	REST3	28	50
	706	37.68	37.14	35.84	84	7	FAN	REST3	28	50
	707	37.68	37.13	35.85	84	7	FAN	REST3	28	50
	708	37.67	37.15	35.85	86	7	FAN	REST3	28	50
	709	37.68	37.17	35.86	82	7	FAN	REST3	28	50
0	1	36.76	36.79	35.12	71	8	FAN	REST1	28	50
	2	36.77	36.80	35.13	73	8	FAN	REST1	28	50
	3	36.77	36.80	35.14	71	8	FAN	REST1	28	50
	4	36.77	36.81	35.14	66	8	FAN	REST1	28	50
	5	36.77	36.81	35.14	67	8	FAN	REST1	28	50
	6	36.76	36.80	35.13	77	8	FAN	REST1	28	50
	7	36.76	36.79	35.13	69	8	FAN	REST1	28	50
	8	36.76	36.79	35.14	70	8	FAN	REST1	28	50
	9	36.76	36.80	35.15	71	8	FAN	REST1	28	50
	10	36.75	36.80	35.16	70	8	FAN	REST1	28	50
	11	36.76	36.81	35.18	77	8	FAN	REST1	28	50
	12	36.76	36.81	35.20	71	8	FAN	REST1	28	50
	13	36.77	36.80	35.21	72	8	FAN	REST1	28	50
	14	36.77	36.77	35.20	68	8	FAN	REST1	28	50
	15	36.76	36.77	35.20	70	8	FAN	REST1	28	50
	16	36.76	36.79	35.23	73	8	FAN	REST1	28	50
	17	36.76	36.77	35.24	66	8	FAN	REST1	28	50
	18	36.76	36.76	35.23	73	8	FAN	REST1	28	50
	19	36.76	36.78	35.24	72	8	FAN	REST1	28	50
	20	36.76	36.77	35.24	71	8	FAN	REST1	28	50
	21	36.76	36.77	35.24	73	8	FAN	REST1	28	50
	22	36.75	36.77	35.25	73	8	FAN	REST1	28	50
	23	36.76	36.77	35.26	70	8	FAN	REST1	28	50
	24	36.76	36.77	35.26	72	8	FAN	REST1	28	50
	25	36.76	36.76	35.25	74	8	FAN	REST1	28	50
	26	36.76	36.77	35.27	72	8	FAN	REST1	28	50
	27	36.76	36.76	35.27	74	8	FAN	REST1	28	50
	28	36.76	36.75	35.28	69	8	FAN	REST1	28	50
	29	36.76	36.77	35.29	71	8	FAN	REST1	28	50
	30	36.77	36.79	35.29	73	8	FAN	REST1	28	50
5	31	36.76	36.80	35.31	68	8	FAN	REST1	28	50

min	number	Tre	Tes	Tsk-head	HR	ID	condition	period	Ta	RH
	32	36.76	36.81	35.32	61	8	FAN	REST1	28	50
	33	36.77	36.78	35.32	67	8	FAN	REST1	28	50
	34	36.76	36.77	35.32	70	8	FAN	REST1	28	50
	35	36.76	36.78	35.31	74	8	FAN	REST1	28	50
	36	36.77	36.77	35.32	67	8	FAN	REST1	28	50
	37	36.77	36.77	35.35	75	8	FAN	REST1	28	50
	38	36.76	36.77	35.36	76	8	FAN	REST1	28	50
	39	36.76	36.75	35.37	74	8	FAN	REST1	28	50
	40	36.76	36.74	35.37	72	8	FAN	REST1	28	50
	41	36.76	36.75	35.39	69	8	FAN	REST1	28	50
	42	36.76	36.77	35.40	69	8	FAN	REST1	28	50
	43	36.77	36.76	35.40	70	8	FAN	REST1	28	50
	44	36.77	36.77	35.41	69	8	FAN	REST1	28	50
	45	36.77	36.77	35.40	63	8	FAN	REST1	28	50
	46	36.77	36.76	35.41	68	8	FAN	REST1	28	50
	47	36.77	36.76	35.42	69	8	FAN	REST1	28	50
	48	36.76	36.77	35.43	66	8	FAN	REST1	28	50
	49	36.76	36.77	35.44	69	8	FAN	REST1	28	50
	50	36.77	36.76	35.44	74	8	FAN	REST1	28	50
	51	36.77	36.74	35.44	71	8	FAN	REST1	28	50
	52	36.76	36.75	35.43	67	8	FAN	REST1	28	50
	53	36.76	36.78	35.43	70	8	FAN	REST1	28	50
	54	36.77	36.78	35.44	62	8	FAN	REST1	28	50
	55	36.78	36.76	35.44	67	8	FAN	REST1	28	50
	56	36.77	36.75	35.45	70	8	FAN	REST1	28	50
	57	36.76	36.75	35.45	67	8	FAN	REST1	28	50
	58	36.75	36.76	35.45	68	8	FAN	REST1	28	50
	59	36.76	36.78	35.46	70	8	FAN	REST1	28	50
	60	36.76	36.79	35.48	73	8	FAN	REST1	28	50
10	61	36.76	36.80	35.48	71	8	FAN	REST1	28	50
	62	36.77	36.79	35.48	69	8	FAN	REST1	28	50
	63	36.76	36.77	35.48	68	8	FAN	REST1	28	50
	64	36.76	36.78	35.48	67	8	FAN	REST1	28	50
	65	36.76	36.77	35.49	67	8	FAN	REST1	28	50
	66	36.76	36.77	35.49	74	8	FAN	REST1	28	50
	67	36.77	36.78	35.50	71	8	FAN	REST1	28	50
	68	36.77	36.78	35.50	69	8	FAN	REST1	28	50
	69	36.77	36.78	35.50	69	8	FAN	REST1	28	50
	70	36.78	36.79	35.51	67	8	FAN	REST1	28	50
	71	36.78	36.79	35.52	73	8	FAN	REST1	28	50
	72	36.77	36.78	35.52	77	8	FAN	REST1	28	50
	73	36.78	36.79	35.51	68	8	FAN	REST1	28	50
	74	36.78	36.78	35.51	70	8	FAN	REST1	28	50
	75	36.78	36.78	35.52	63	8	FAN	REST1	28	50
	76	36.78	36.78	35.53	74	8	FAN	REST1	28	50
	77	36.78	36.79	35.53	73	8	FAN	REST1	28	50

min	number	Tre	Tes	Tsk-head	HR	ID	condition	period	Ta	RH
	78	36.79	36.81	35.55	85	8	FAN	REST1	28	50
	79	36.79	36.81	35.56	76	8	FAN	REST1	28	50
	80	36.79	36.81	35.56	78	8	FAN	REST1	28	50
	81	36.79	36.79	35.56	78	8	FAN	REST1	28	50
	82	36.79	36.78	35.57	76	8	FAN	REST1	28	50
	83	36.79	36.78	35.57	79	8	FAN	REST1	28	50
	84	36.80	36.80	35.58	73	8	FAN	REST1	28	50
	85	36.80	36.81	35.57	70	8	FAN	REST1	28	50
	86	36.79	36.79	35.57	77	8	FAN	REST1	28	50
	87	36.79	36.79	35.57	75	8	FAN	REST1	28	50
	88	36.79	36.82	35.57	78	8	FAN	REST1	28	50
	89	36.79	36.81	35.57	72	8	FAN	REST1	28	50
	90	36.79	36.80	35.57	81	8	FAN	REST1	28	50
15	91	36.79	36.81	35.58	71	8	FAN	REST1	28	50
	92	36.79	36.82	35.58	68	8	FAN	REST1	28	50
	93	36.79	36.82	35.59	73	8	FAN	REST1	28	50
	94	36.79	36.81	35.59	72	8	FAN	REST1	28	50
	95	36.79	36.82	35.60	91	8	FAN	REST1	28	50
	96	36.79	36.85	35.59	78	8	FAN	REST1	28	50
	97	36.78	36.84	35.59	70	8	FAN	REST1	28	50
	98	36.78	36.83	35.60	77	8	FAN	REST1	28	50
	99	36.78	36.84	35.60	85	8	FAN	REST1	28	50
	100	36.78	36.83	35.60	83	8	FAN	REST1	28	50
	101	36.78	36.82	35.60	70	8	FAN	REST1	28	50
	102	36.78	36.84	35.61	76	8	FAN	REST1	28	50
	103	36.78	36.84	35.62	72	8	FAN	REST1	40	50
	104	36.77	36.82	35.70	86	8	FAN	REST1	40	50
	105	36.78	36.84	35.89	93	8	FAN	REST1	40	50
	106	36.78	36.84	36.09	92	8	FAN	REST1	40	50
	107	36.77	36.82	36.25	80	8	FAN	REST1	40	50
	108	36.78	36.84	36.39	77	8	FAN	REST1	40	50
	109	36.78	36.84	36.47	76	8	FAN	REST1	40	50
	110	36.77	36.82	36.52	79	8	FAN	REST1	40	50
	111	36.76	36.82	36.56	87	8	FAN	REST1	40	50
	112	36.76	36.84	36.57	81	8	FAN	REST1	40	50
	113	36.76	36.84	36.60	78	8	FAN	REST1	40	50
	114	36.76	36.83	36.61	78	8	FAN	REST1	40	50
	115	36.76	36.83	36.63	79	8	FAN	REST1	40	50
	116	36.76	36.85	36.66	77	8	FAN	REST1	40	50
	117	36.76	36.85	36.67	75	8	FAN	REST1	40	50
	118	36.76	36.85	36.69	75	8	FAN	REST1	40	50
	119	36.77	36.84	36.70	71	8	FAN	REST1	40	50
	120	36.76	36.83	36.70	75	8	FAN	REST1	40	50
20	121	36.76	36.83	36.71	75	8	FAN	REST1	40	50
	122	36.77	36.85	36.73	77	8	FAN	REST1	40	50
	123	36.77	36.86	36.75	67	8	FAN	REST1	40	50

min	number	Tre	Tes	Tsk-head	HR	ID	condition	period	Ta	RH
	124	36.77	36.86	36.77	71	8	FAN	REST1	40	50
	125	36.77	36.85	36.78	80	8	FAN	REST1	40	50
	126	36.76	36.86	36.78	70	8	FAN	REST1	40	50
	127	36.77	36.88	36.78	83	8	FAN	REST1	40	50
	128	36.77	36.88	36.77	78	8	FAN	REST1	40	50
	129	36.76	36.87	36.77	79	8	FAN	REST1	40	50
	130	36.76	36.86	36.78	82	8	FAN	REST1	40	50
	131	36.77	36.85	36.82	82	8	FAN	REST1	40	50
	132	36.77	36.85	36.83	79	8	FAN	REST1	40	50
	133	36.77	36.86	36.81	78	8	FAN	REST1	40	50
	134	36.77	36.87	36.80	81	8	FAN	REST1	40	50
	135	36.78	36.88	36.81	100	8	FAN	REST1	40	50
	136	36.78	36.86	36.80	82	8	FAN	REST1	40	50
	137	36.78	36.86	36.77	79	8	FAN	REST1	40	50
	138	36.77	36.86	36.78	75	8	FAN	REST1	40	50
	139	36.77	36.85	36.78	81	8	FAN	EXERCISE1	40	50
	140	36.77	36.86	36.79	79	8	FAN	EXERCISE1	40	50
	141	36.76	36.89	36.80	88	8	FAN	EXERCISE1	40	50
	142	36.76	36.89	36.80	94	8	FAN	EXERCISE1	40	50
	143	36.76	36.89	36.82	100	8	FAN	EXERCISE1	40	50
	144	36.76	36.88	36.83	99	8	FAN	EXERCISE1	40	50
	145	36.76	36.86	36.83	104	8	FAN	EXERCISE1	40	50
	146	36.76	36.86	36.83	108	8	FAN	EXERCISE1	40	50
	147	36.76	36.87	36.82	105	8	FAN	EXERCISE1	40	50
	148	36.76	36.87	36.82	104	8	FAN	EXERCISE1	40	50
	149	36.77	36.87	36.82	108	8	FAN	EXERCISE1	40	50
	150	36.77	36.87	36.82	106	8	FAN	EXERCISE1	40	50
25	151	36.77	36.89	36.81	104	8	FAN	EXERCISE1	40	50
	152	36.77	36.88	36.79	106	8	FAN	EXERCISE1	40	50
	153	36.76	36.88	36.79	104	8	FAN	EXERCISE1	40	50
	154	36.76	36.88	36.80	101	8	FAN	EXERCISE1	40	50
	155	36.76	36.88	36.81	103	8	FAN	EXERCISE1	40	50
	156	36.76	36.90	36.82	106	8	FAN	EXERCISE1	40	50
	157	36.76	36.90	36.81	108	8	FAN	EXERCISE1	40	50
	158	36.76	36.89	36.81	109	8	FAN	EXERCISE1	40	50
	159	36.77	36.90	36.83	105	8	FAN	EXERCISE1	40	50
	160	36.77	36.90	36.83	108	8	FAN	EXERCISE1	40	50
	161	36.77	36.89	36.84	108	8	FAN	EXERCISE1	40	50
	162	36.77	36.90	36.85	109	8	FAN	EXERCISE1	40	50
	163	36.77	36.91	36.84	107	8	FAN	EXERCISE1	40	50
	164	36.77	36.90	36.84	110	8	FAN	EXERCISE1	40	50
	165	36.77	36.90	36.85	107	8	FAN	EXERCISE1	40	50
	166	36.76	36.88	36.84	110	8	FAN	EXERCISE1	40	50
	167	36.76	36.88	36.84	107	8	FAN	EXERCISE1	40	50
	168	36.77	36.89	36.84	105	8	FAN	EXERCISE1	40	50
	169	36.77	36.90	36.85	103	8	FAN	EXERCISE1	40	50

min	number	Tre	Tes	Tsk-head	HR	ID	condition	period	Ta	RH
	170	36.78	36.92	36.86	103	8	FAN	EXERCISE1	40	50
	171	36.78	36.94	36.87	108	8	FAN	EXERCISE1	40	50
	172	36.79	36.96	36.88	109	8	FAN	EXERCISE1	40	50
	173	36.79	36.96	36.88	115	8	FAN	EXERCISE1	40	50
	174	36.78	36.96	36.89	113	8	FAN	EXERCISE1	40	50
	175	36.79	36.96	36.90	115	8	FAN	EXERCISE1	40	50
	176	36.79	36.96	36.89	116	8	FAN	EXERCISE1	40	50
	177	36.79	36.96	36.89	112	8	FAN	EXERCISE1	40	50
	178	36.78	36.95	36.88	112	8	FAN	EXERCISE1	40	50
	179	36.78	36.97	36.88	111	8	FAN	EXERCISE1	40	50
	180	36.79	36.98	36.89	112	8	FAN	EXERCISE1	40	50
30	181	36.79	36.97	36.88	111	8	FAN	EXERCISE1	40	50
	182	36.79	36.96	36.87	109	8	FAN	EXERCISE1	40	50
	183	36.80	36.97	36.88	108	8	FAN	EXERCISE1	40	50
	184	36.80	36.99	36.89	114	8	FAN	EXERCISE1	40	50
	185	36.79	36.99	36.89	111	8	FAN	EXERCISE1	40	50
	186	36.81	36.98	36.89	112	8	FAN	EXERCISE1	40	50
	187	36.81	36.99	36.89	112	8	FAN	EXERCISE1	40	50
	188	36.80	36.98	36.87	112	8	FAN	EXERCISE1	40	50
	189	36.81	36.97	36.88	116	8	FAN	EXERCISE1	40	50
	190	36.82	36.99	36.88	111	8	FAN	EXERCISE1	40	50
	191	36.82	37.02	36.88	114	8	FAN	EXERCISE1	40	50
	192	36.82	37.02	36.87	112	8	FAN	EXERCISE1	40	50
	193	36.82	37.00	36.86	109	8	FAN	EXERCISE1	40	50
	194	36.82	37.02	36.86	111	8	FAN	EXERCISE1	40	50
	195	36.83	37.03	36.88	110	8	FAN	EXERCISE1	40	50
	196	36.83	37.03	36.89	110	8	FAN	EXERCISE1	40	50
	197	36.83	37.03	36.89	110	8	FAN	EXERCISE1	40	50
	198	36.84	37.05	36.89	113	8	FAN	EXERCISE1	40	50
	199	36.84	37.04	36.90	114	8	FAN	EXERCISE1	40	50
	200	36.83	37.03	36.91	115	8	FAN	EXERCISE1	40	50
	201	36.84	37.05	36.92	115	8	FAN	EXERCISE1	40	50
	202	36.85	37.04	36.91	115	8	FAN	EXERCISE1	40	50
	203	36.85	37.04	36.89	117	8	FAN	EXERCISE1	40	50
	204	36.86	37.04	36.88	116	8	FAN	EXERCISE1	40	50
	205	36.87	37.05	36.89	115	8	FAN	EXERCISE1	40	50
	206	36.87	37.07	36.91	118	8	FAN	EXERCISE1	40	50
	207	36.87	37.06	36.91	115	8	FAN	EXERCISE1	40	50
	208	36.87	37.05	36.90	117	8	FAN	EXERCISE1	40	50
	209	36.87	37.05	36.90	118	8	FAN	EXERCISE1	40	50
	210	36.87	37.06	36.88	117	8	FAN	EXERCISE1	40	50
35	211	36.87	37.05	36.86	115	8	FAN	EXERCISE1	40	50
	212	36.88	37.06	36.85	120	8	FAN	EXERCISE1	40	50
	213	36.88	37.08	36.85	114	8	FAN	EXERCISE1	40	50
	214	36.88	37.06	36.84	118	8	FAN	EXERCISE1	40	50
	215	36.89	37.05	36.84	119	8	FAN	EXERCISE1	40	50

min	number	Tre	Tes	Tsk-head	HR	ID	condition	period	Ta	RH
	216	36.89	37.05	36.84	119	8	FAN	EXERCISE1	40	50
	217	36.89	37.05	36.84	119	8	FAN	EXERCISE1	40	50
	218	36.89	37.07	36.85	122	8	FAN	EXERCISE1	40	50
	219	36.90	37.06	36.85	123	8	FAN	EXERCISE1	40	50
	220	36.90	37.08	36.84	122	8	FAN	EXERCISE1	40	50
	221	36.90	37.09	36.84	124	8	FAN	EXERCISE1	40	50
	222	36.91	37.08	36.85	120	8	FAN	EXERCISE1	40	50
	223	36.91	37.08	36.84	119	8	FAN	EXERCISE1	40	50
	224	36.91	37.09	36.83	120	8	FAN	EXERCISE1	40	50
	225	36.91	37.08	36.82	118	8	FAN	EXERCISE1	40	50
	226	36.91	37.09	36.82	119	8	FAN	EXERCISE1	40	50
	227	36.91	37.09	36.84	115	8	FAN	EXERCISE1	40	50
	228	36.92	37.10	36.84	115	8	FAN	EXERCISE1	40	50
	229	36.91	37.11	36.82	118	8	FAN	EXERCISE1	40	50
	230	36.91	37.11	36.82	120	8	FAN	EXERCISE1	40	50
	231	36.92	37.12	36.83	121	8	FAN	EXERCISE1	40	50
	232	36.93	37.11	36.84	123	8	FAN	EXERCISE1	40	50
	233	36.93	37.11	36.85	126	8	FAN	EXERCISE1	40	50
	234	36.94	37.11	36.83	123	8	FAN	EXERCISE1	40	50
	235	36.94	37.12	36.82	119	8	FAN	EXERCISE1	40	50
	236	36.94	37.13	36.82	119	8	FAN	EXERCISE1	40	50
	237	36.94	37.12	36.82	121	8	FAN	EXERCISE1	40	50
	238	36.94	37.14	36.79	122	8	FAN	EXERCISE1	40	50
	239	36.95	37.17	36.77	123	8	FAN	EXERCISE1	40	50
	240	36.96	37.16	36.80	124	8	FAN	EXERCISE1	40	50
40	241	36.95	37.15	36.77	117	8	FAN	EXERCISE1	40	50
	242	36.96	37.14	36.75	119	8	FAN	EXERCISE1	40	50
	243	36.96	37.15	36.78	117	8	FAN	EXERCISE1	40	50
	244	36.96	37.18	36.79	123	8	FAN	EXERCISE1	40	50
	245	36.97	37.18	36.77	123	8	FAN	EXERCISE1	40	50
	246	36.96	37.16	36.76	121	8	FAN	EXERCISE1	40	50
	247	36.96	37.15	36.77	120	8	FAN	EXERCISE1	40	50
	248	36.97	37.17	36.75	121	8	FAN	EXERCISE1	40	50
	249	36.97	37.17	36.73	124	8	FAN	EXERCISE1	40	50
	250	36.98	37.18	36.71	126	8	FAN	EXERCISE1	40	50
	251	36.99	37.19	36.72	124	8	FAN	EXERCISE1	40	50
	252	36.99	37.17	36.77	124	8	FAN	EXERCISE1	40	50
	253	36.99	37.18	36.79	124	8	FAN	EXERCISE1	40	50
	254	36.99	37.20	36.76	123	8	FAN	EXERCISE1	40	50
	255	37.00	37.20	36.75	121	8	FAN	EXERCISE1	40	50
	256	37.00	37.20	36.74	121	8	FAN	EXERCISE1	40	50
	257	37.00	37.18	36.72	123	8	FAN	EXERCISE1	40	50
	258	37.01	37.17	36.75	119	8	FAN	EXERCISE1	40	50
	259	37.01	37.18	36.78	122	8	FAN	EXERCISE1	40	50
	260	37.01	37.21	36.77	126	8	FAN	EXERCISE1	40	50
	261	37.01	37.21	36.76	122	8	FAN	EXERCISE1	40	50

min	number	Tre	Tes	Tsk-head	HR	ID	condition	period	Ta	RH
	262	37.02	37.21	36.77	123	8	FAN	EXERCISE1	40	50
	263	37.03	37.22	36.77	124	8	FAN	EXERCISE1	40	50
	264	37.03	37.21	36.76	126	8	FAN	EXERCISE1	40	50
	265	37.03	37.24	36.76	127	8	FAN	EXERCISE1	40	50
	266	37.03	37.23	36.75	126	8	FAN	EXERCISE1	40	50
	267	37.03	37.20	36.70	125	8	FAN	EXERCISE1	40	50
	268	37.03	37.20	36.69	125	8	FAN	EXERCISE1	40	50
	269	37.03	37.21	36.69	123	8	FAN	EXERCISE1	40	50
	270	37.04	37.22	36.69	125	8	FAN	EXERCISE1	40	50
45	271	37.05	37.23	36.64	124	8	FAN	EXERCISE1	40	50
	272	37.05	37.22	36.58	124	8	FAN	EXERCISE1	40	50
	273	37.05	37.24	36.60	124	8	FAN	EXERCISE1	40	50
	274	37.05	37.25	36.61	126	8	FAN	EXERCISE1	40	50
	275	37.05	37.24	36.64	130	8	FAN	EXERCISE1	40	50
	276	37.06	37.24	36.65	128	8	FAN	EXERCISE1	40	50
	277	37.07	37.26	36.61	128	8	FAN	EXERCISE1	40	50
	278	37.07	37.28	36.60	126	8	FAN	EXERCISE1	40	50
	279	37.07	37.28	36.61	124	8	FAN	EXERCISE1	40	50
	280	37.07	37.27	36.61	125	8	FAN	EXERCISE1	40	50
	281	37.07	37.27	36.59	125	8	FAN	EXERCISE1	40	50
	282	37.07	37.27	36.59	123	8	FAN	EXERCISE1	40	50
	283	37.08	37.27	36.60	126	8	FAN	EXERCISE1	40	50
	284	37.09	37.29	36.60	125	8	FAN	EXERCISE1	40	50
	285	37.09	37.29	36.57	128	8	FAN	EXERCISE1	40	50
	286	37.09	37.29	36.58	124	8	FAN	EXERCISE1	40	50
	287	37.09	37.30	36.60	124	8	FAN	EXERCISE1	40	50
	288	37.08	37.26	36.58	127	8	FAN	EXERCISE1	40	50
	289	37.09	37.27	36.57	128	8	FAN	EXERCISE1	40	50
	290	37.10	37.28	36.58	128	8	FAN	EXERCISE1	40	50
	291	37.10	37.27	36.60	127	8	FAN	EXERCISE1	40	50
	292	37.10	37.30	36.59	126	8	FAN	EXERCISE1	40	50
	293	37.10	37.30	36.57	129	8	FAN	EXERCISE1	40	50
	294	37.10	37.30	36.56	130	8	FAN	EXERCISE1	40	50
	295	37.10	37.30	36.55	131	8	FAN	EXERCISE1	40	50
	296	37.10	37.31	36.54	129	8	FAN	EXERCISE1	40	50
	297	37.10	37.31	36.56	127	8	FAN	EXERCISE1	40	50
	298	37.11	37.31	36.58	129	8	FAN	EXERCISE1	40	50
	299	37.10	37.29	36.58	131	8	FAN	EXERCISE1	40	50
	300	37.10	37.29	36.58	130	8	FAN	EXERCISE1	40	50
50	301	37.11	37.30	36.57	130	8	FAN	EXERCISE1	40	50
	302	37.11	37.31	36.55	131	8	FAN	EXERCISE1	40	50
	303	37.12	37.33	36.55	128	8	FAN	EXERCISE1	40	50
	304	37.12	37.33	36.57	131	8	FAN	EXERCISE1	40	50
	305	37.12	37.33	36.56	130	8	FAN	EXERCISE1	40	50
	306	37.13	37.33	36.55	128	8	FAN	EXERCISE1	40	50
	307	37.13	37.34	36.54	124	8	FAN	EXERCISE1	40	50

min	number	Tre	Tes	Tsk-head	HR	ID	condition	period	Ta	RH
	308	37.13	37.33	36.54	130	8	FAN	EXERCISE1	40	50
	309	37.13	37.34	36.55	130	8	FAN	EXERCISE1	40	50
	310	37.12	37.33	36.55	128	8	FAN	EXERCISE1	40	50
	311	37.13	37.33	36.52	128	8	FAN	EXERCISE1	40	50
	312	37.14	37.34	36.49	130	8	FAN	EXERCISE1	40	50
	313	37.14	37.32	36.50	128	8	FAN	EXERCISE1	40	50
	314	37.14	37.31	36.51	128	8	FAN	EXERCISE1	40	50
	315	37.15	37.34	36.51	127	8	FAN	EXERCISE1	40	50
	316	37.15	37.35	36.52	126	8	FAN	EXERCISE1	40	50
	317	37.15	37.35	36.55	130	8	FAN	EXERCISE1	40	50
	318	37.15	37.35	36.56	127	8	FAN	EXERCISE1	40	50
	319	37.15	37.34	36.56	126	8	FAN	EXERCISE1	40	50
	320	37.16	37.36	36.58	120	8	FAN	REST2	28	50
	321	37.17	37.38	36.60	130	8	FAN	REST2	28	50
	322	37.16	37.37	36.59	131	8	FAN	REST2	28	50
	323	37.16	37.38	36.44	117	8	FAN	REST2	28	50
	324	37.17	37.41	36.17	100	8	FAN	REST2	28	50
	325	37.18	37.43	36.07	92	8	FAN	REST2	28	50
	326	37.18	37.42	36.08	94	8	FAN	REST2	28	50
	327	37.18	37.39	36.07	93	8	FAN	REST2	28	50
	328	37.19	37.39	36.09	95	8	FAN	REST2	28	50
	329	37.19	37.40	35.98	90	8	FAN	REST2	28	50
	330	37.19	37.41	35.85	92	8	FAN	REST2	28	50
55	331	37.19	37.43	35.88	86	8	FAN	REST2	28	50
	332	37.19	37.44	35.84	85	8	FAN	REST2	28	50
	333	37.19	37.42	35.75	86	8	FAN	REST2	28	50
	334	37.19	37.39	35.74	94	8	FAN	REST2	28	50
	335	37.19	37.37	35.69	79	8	FAN	REST2	28	50
	336	37.18	37.37	35.65	85	8	FAN	REST2	28	50
	337	37.17	37.37	35.67	80	8	FAN	REST2	28	50
	338	37.16	37.34	35.63	84	8	FAN	REST2	28	50
	339	37.15	35.25	35.61	90	8	FAN	REST2	28	50
	340	37.16	32.79	35.58	91	8	FAN	REST2	28	50
	341	37.16	32.63	35.52	88	8	FAN	REST2	28	50
	342	37.14	32.77	35.44	90	8	FAN	REST2	28	50
	343	37.13	32.50	35.23	84	8	FAN	REST2	28	50
	344	37.13	32.23	35.19	91	8	FAN	REST2	28	50
	345	37.14	32.26	35.29	82	8	FAN	REST2	28	50
	346	37.13	32.62	35.29	83	8	FAN	REST2	28	50
	347	37.13	33.10	35.25	84	8	FAN	REST2	28	50
	348	37.12	33.55	35.11	80	8	FAN	REST2	28	50
	349	37.11	33.98	34.99	75	8	FAN	REST2	28	50
	350	37.10	34.35	34.94	80	8	FAN	REST2	28	50
	351	37.09	34.67	34.90	78	8	FAN	REST2	28	50
	352	37.09	34.94	34.84	76	8	FAN	REST2	28	50
	353	37.09	34.04	34.84	86	8	FAN	REST2	28	50

min	number	Tre	Tes	Tsk-head	HR	ID	condition	period	Ta	RH
	354	37.08	32.05	34.84	91	8	FAN	REST2	28	50
	355	37.08	31.38	34.79	81	8	FAN	REST2	28	50
	356	37.08	31.86	34.73	76	8	FAN	REST2	28	50
	357	37.07	32.24	34.70	88	8	FAN	REST2	28	50
	358	37.06	32.58	34.78	87	8	FAN	REST2	28	50
	359	37.05	30.26	34.81	94	8	FAN	REST2	28	50
	360	37.05	29.72	34.73	88	8	FAN	REST2	28	50
60	361	37.05	31.74	34.64	79	8	FAN	REST2	28	50
	362	37.04	31.97	34.58	75	8	FAN	REST2	28	50
	363	37.05	31.90	34.55	77	8	FAN	REST2	28	50
	364	37.05	32.08	34.56	72	8	FAN	REST2	28	50
	365	37.04	32.62	34.59	71	8	FAN	REST2	28	50
	366	37.04	32.47	34.58	84	8	FAN	REST2	28	50
	367	37.04	30.54	34.64	91	8	FAN	REST2	28	50
	368	37.04	29.45	34.69	82	8	FAN	REST2	28	50
	369	37.04	30.41	34.62	79	8	FAN	REST2	28	50
	370	37.04	30.79	34.60	74	8	FAN	REST2	28	50
	371	37.03	30.79	34.66	68	8	FAN	REST2	28	50
	372	37.02	31.12	34.70	69	8	FAN	REST2	28	50
	373	37.03	31.47	34.72	73	8	FAN	REST2	28	50
	374	37.03	31.54	34.74	71	8	FAN	REST2	28	50
	375	37.01	31.74	34.74	81	8	FAN	REST2	28	50
	376	37.01	30.78	34.83	90	8	FAN	REST2	28	50
	377	37.00	29.71	34.81	82	8	FAN	REST2	28	50
	378	37.00	30.11	34.72	80	8	FAN	REST2	28	50
	379	37.00	30.87	34.77	87	8	FAN	REST2	28	50
	380	37.00	29.15	34.90	89	8	FAN	REST2	28	50
	381	37.00	27.76	34.90	87	8	FAN	REST2	28	50
	382	36.99	28.84	34.79	74	8	FAN	REST2	28	50
	383	36.99	29.15	34.77	75	8	FAN	REST2	28	50
	384	36.99	29.65	34.74	78	8	FAN	REST2	28	50
	385	36.98	30.02	34.71	68	8	FAN	REST2	28	50
	386	36.98	29.89	34.67	74	8	FAN	REST2	28	50
	387	36.99	30.06	34.62	78	8	FAN	REST2	28	50
	388	37.00	30.39	34.59	74	8	FAN	REST2	28	50
	389	37.01	30.72	34.65	78	8	FAN	REST2	28	50
	390	37.02	30.93	34.70	71	8	FAN	REST2	28	50
65	391	37.02	30.90	34.72	78	8	FAN	REST2	28	50
	392	37.02	30.88	34.76	75	8	FAN	REST2	28	50
	393	37.03	30.96	34.77	75	8	FAN	REST2	28	50
	394	37.03	31.13	34.77	73	8	FAN	REST2	28	50
	395	37.02	31.21	34.78	71	8	FAN	REST2	28	50
	396	37.02	31.20	34.81	77	8	FAN	REST2	28	50
	397	37.03	31.28	34.85	76	8	FAN	REST2	28	50
	398	37.03	31.36	34.84	79	8	FAN	REST2	28	50
	399	37.03	31.41	34.83	75	8	FAN	REST2	28	50

min	number	Tre	Tes	Tsk-head	HR	ID	condition	period	Ta	RH
	400	37.03	31.61	34.82	71	8	FAN	REST2	28	50
	401	37.03	31.79	34.81	71	8	FAN	REST2	28	50
	402	37.02	31.81	34.79	74	8	FAN	REST2	28	50
	403	37.03	31.93	34.83	71	8	FAN	REST2	28	50
	404	37.03	32.03	34.93	72	8	FAN	REST2	28	50
	405	37.03	32.12	34.98	69	8	FAN	REST2	28	50
	406	37.02	32.20	34.93	72	8	FAN	REST2	28	50
	407	37.01	32.29	34.84	72	8	FAN	REST2	28	50
	408	37.00	32.36	34.81	73	8	FAN	REST2	28	50
	409	36.99	32.43	34.87	71	8	FAN	REST2	28	50
	410	36.98	32.57	34.91	71	8	FAN	REST2	28	50
	411	36.98	32.63	34.95	77	8	FAN	REST2	28	50
	412	36.97	32.70	35.00	73	8	FAN	REST2	28	50
	413	36.97	32.84	35.04	74	8	FAN	REST2	28	50
	414	36.98	32.91	35.10	73	8	FAN	REST2	28	50
	415	36.97	32.97	35.14	72	8	FAN	REST2	28	50
	416	36.97	33.05	35.12	75	8	FAN	REST2	28	50
	417	36.98	33.29	35.05	80	8	FAN	REST2	28	50
	418	36.97	33.48	35.01	81	8	FAN	REST2	28	50
	419	36.97	33.54	35.24	67	8	FAN	REST2	28	50
	420	36.97	33.63	35.36	70	8	FAN	REST2	28	50
70	421	36.97	33.68		70	8	FAN	REST2	28	50
	422	36.97	33.77		65	8	FAN	REST2	28	50
	423	36.97	33.84		72	8	FAN	REST2	28	50
	424	36.97	33.78	35.27	68	8	FAN	REST2	28	50
	425	36.97	33.80	35.42	68	8	FAN	REST2	28	50
	426	36.97	33.92	35.46	66	8	FAN	REST2	28	50
	427	36.98	33.90	35.46	70	8	FAN	REST2	28	50
	428	36.98	33.92	35.46	69	8	FAN	REST2	28	50
	429	36.98	34.03	35.49	71	8	FAN	REST2	28	50
	430	36.98	34.12	35.52	70	8	FAN	REST2	28	50
	431	36.99	34.09	35.55	78	8	FAN	REST2	28	50
	432	36.99	34.09	35.58	83	8	FAN	REST2	28	50
	433	37.00	34.16	35.58	82	8	FAN	REST2	28	50
	434	37.00	34.27	35.61	72	8	FAN	REST2	28	50
	435	37.00	34.24	35.64	83	8	FAN	REST2	28	50
	436	37.00	34.14	35.62	82	8	FAN	REST2	28	50
	437	37.00	34.22	35.60	76	8	FAN	REST2	28	50
	438	37.00	34.30	35.64	78	8	FAN	REST2	28	50
	439	37.00	34.40	35.68	70	8	FAN	REST2	40	50
	440	36.99	34.64	35.86	91	8	FAN	REST2	40	50
	441	36.99	34.78	36.17	96	8	FAN	REST2	40	50
	442	37.01	34.87	36.37	90	8	FAN	REST2	40	50
	443	37.02	34.91	36.52	80	8	FAN	REST2	40	50
	444	37.02	34.86	36.65	74	8	FAN	REST2	40	50
	445	37.02	34.93	36.72	70	8	FAN	REST2	40	50

min	number	Tre	Tes	Tsk-head	HR	ID	condition	period	Ta	RH
	446	37.02	34.98	36.73	76	8	FAN	REST2	40	50
	447	37.02	35.84	36.74	72	8	FAN	REST2	40	50
	448	37.04	36.78	36.76	71	8	FAN	REST2	40	50
	449	37.03	36.83	36.71	67	8	FAN	REST2	40	50
	450	37.03	36.81	36.71	74	8	FAN	REST2	40	50
75	451	37.04	36.80	36.79	74	8	FAN	REST2	40	50
	452	37.03	36.80	36.81	79	8	FAN	REST2	40	50
	453	37.03	36.82	36.83	80	8	FAN	REST2	40	50
	454	37.04	36.80	36.85	73	8	FAN	REST2	40	50
	455	37.04	36.79	36.86	78	8	FAN	REST2	40	50
	456	37.04	36.78	36.86	71	8	FAN	REST2	40	50
	457	37.03	36.76	36.87	72	8	FAN	REST2	40	50
	458	37.05	36.80	36.85	96	8	FAN	REST2	40	50
	459	37.05	36.81	36.85	96	8	FAN	REST2	40	50
	460	37.04	36.80	36.88	74	8	FAN	REST2	40	50
	461	37.04	36.74	36.89	79	8	FAN	REST2	40	50
	462	37.03	36.69	36.89	89	8	FAN	REST2	40	50
	463	37.03	36.72	36.89	73	8	FAN	EXERCISE2	40	50
	464	37.04	36.76	36.90	88	8	FAN	EXERCISE2	40	50
	465	37.04	36.79	36.91	96	8	FAN	EXERCISE2	40	50
	466	37.03	36.80	36.92	104	8	FAN	EXERCISE2	40	50
	467	37.03	36.80	36.93	103	8	FAN	EXERCISE2	40	50
	468	37.04	36.80	36.93	105	8	FAN	EXERCISE2	40	50
	469	37.03	36.82	36.92	104	8	FAN	EXERCISE2	40	50
	470	37.02	36.83	36.91	108	8	FAN	EXERCISE2	40	50
	471	37.02	36.83	36.91	112	8	FAN	EXERCISE2	40	50
	472	37.02	36.83	36.92	108	8	FAN	EXERCISE2	40	50
	473	37.02	36.85	36.91	109	8	FAN	EXERCISE2	40	50
	474	37.02	36.41	36.91	108	8	FAN	EXERCISE2	40	50
	475	37.02	36.38	36.91	109	8	FAN	EXERCISE2	40	50
	476	37.03	36.84	36.92	111	8	FAN	EXERCISE2	40	50
	477	37.03	36.86	36.93	108	8	FAN	EXERCISE2	40	50
	478	37.03	36.87	36.94	111	8	FAN	EXERCISE2	40	50
	479	37.03	36.89	36.94	114	8	FAN	EXERCISE2	40	50
	480	37.02	36.88	36.94	112	8	FAN	EXERCISE2	40	50
80	481	37.03	36.89	36.97	112	8	FAN	EXERCISE2	40	50
	482	37.04	36.92	36.99	114	8	FAN	EXERCISE2	40	50
	483	37.04	36.94	36.99	117	8	FAN	EXERCISE2	40	50
	484	37.04	36.94	36.99	112	8	FAN	EXERCISE2	40	50
	485	37.04	36.93	37.00	118	8	FAN	EXERCISE2	40	50
	486	37.04	36.97	37.01	114	8	FAN	EXERCISE2	40	50
	487	37.04	36.97	37.01	113	8	FAN	EXERCISE2	40	50
	488	37.04	36.96	37.02	117	8	FAN	EXERCISE2	40	50
	489	37.05	37.01	37.03	117	8	FAN	EXERCISE2	40	50
	490	37.05	37.02	37.04	122	8	FAN	EXERCISE2	40	50
	491	37.05	37.01	37.04	120	8	FAN	EXERCISE2	40	50

min	number	Tre	Tes	Tsk-head	HR	ID	condition	period	Ta	RH
	492	37.05	37.03	37.04	119	8	FAN	EXERCISE2	40	50
	493	37.06	37.06	37.05	121	8	FAN	EXERCISE2	40	50
	494	37.05	37.08	37.07	125	8	FAN	EXERCISE2	40	50
	495	37.05	37.08	37.08	121	8	FAN	EXERCISE2	40	50
	496	37.06	37.06	37.09	112	8	FAN	EXERCISE2	40	50
	497	37.05	37.05	37.09	116	8	FAN	EXERCISE2	40	50
	498	37.05	37.07	37.09	121	8	FAN	EXERCISE2	40	50
	499	37.06	37.08	37.09	121	8	FAN	EXERCISE2	40	50
	500	37.06	37.09	37.09	119	8	FAN	EXERCISE2	40	50
	501	37.07	37.09	37.09	118	8	FAN	EXERCISE2	40	50
	502	37.08	37.11	37.10	120	8	FAN	EXERCISE2	40	50
	503	37.08	37.13	37.11	122	8	FAN	EXERCISE2	40	50
	504	37.09	37.13	37.12	125	8	FAN	EXERCISE2	40	50
	505	37.08	37.14	37.12	127	8	FAN	EXERCISE2	40	50
	506	37.08	37.13	37.11	125	8	FAN	EXERCISE2	40	50
	507	37.09	37.11	37.10	127	8	FAN	EXERCISE2	40	50
	508	37.09	37.12	37.11	122	8	FAN	EXERCISE2	40	50
	509	37.09	37.13	37.11	122	8	FAN	EXERCISE2	40	50
	510	37.08	37.13	37.10	122	8	FAN	EXERCISE2	40	50
85	511	37.09	37.09	37.10	122	8	FAN	EXERCISE2	40	50
	512	37.09	37.07	37.10	119	8	FAN	EXERCISE2	40	50
	513	37.09	37.10	37.09	121	8	FAN	EXERCISE2	40	50
	514	37.09	37.11	37.09	120	8	FAN	EXERCISE2	40	50
	515	37.10	37.14	37.09	122	8	FAN	EXERCISE2	40	50
	516	37.11	37.14	37.09	118	8	FAN	EXERCISE2	40	50
	517	37.11	37.11	37.10	122	8	FAN	EXERCISE2	40	50
	518	37.11	37.10	37.09	124	8	FAN	EXERCISE2	40	50
	519	37.11	37.13	37.09	125	8	FAN	EXERCISE2	40	50
	520	37.11	37.14	37.08	126	8	FAN	EXERCISE2	40	50
	521	37.11	37.11	37.08	124	8	FAN	EXERCISE2	40	50
	522	37.11	37.13	37.08	125	8	FAN	EXERCISE2	40	50
	523	37.12	37.14	37.09	125	8	FAN	EXERCISE2	40	50
	524	37.12	37.12	37.09	125	8	FAN	EXERCISE2	40	50
	525	37.12	37.13	37.10	127	8	FAN	EXERCISE2	40	50
	526	37.12	37.14	37.10	128	8	FAN	EXERCISE2	40	50
	527	37.13	37.16	37.10	131	8	FAN	EXERCISE2	40	50
	528	37.13	37.13	37.11	127	8	FAN	EXERCISE2	40	50
	529	37.13	37.12	37.11	125	8	FAN	EXERCISE2	40	50
	530	37.14	37.07	37.11	128	8	FAN	EXERCISE2	40	50
	531	37.14	37.06	37.11	129	8	FAN	EXERCISE2	40	50
	532	37.15	37.13	37.12	129	8	FAN	EXERCISE2	40	50
	533	37.15	37.14	37.11	126	8	FAN	EXERCISE2	40	50
	534	37.14	37.14	37.12	125	8	FAN	EXERCISE2	40	50
	535	37.13	37.15	37.12	124	8	FAN	EXERCISE2	40	50
	536	37.13	37.17	37.11	125	8	FAN	EXERCISE2	40	50
	537	37.13	37.17	37.12	126	8	FAN	EXERCISE2	40	50

min	number	Tre	Tes	Tsk-head	HR	ID	condition	period	Ta	RH
	538	37.14	37.18	37.12	125	8	FAN	EXERCISE2	40	50
	539	37.14	37.20	37.12	127	8	FAN	EXERCISE2	40	50
	540	37.14	37.19	37.12	126	8	FAN	EXERCISE2	40	50
90	541	37.14	37.16	37.11	129	8	FAN	EXERCISE2	40	50
	542	37.14	37.18	37.11	129	8	FAN	EXERCISE2	40	50
	543	37.15	37.20	37.12	126	8	FAN	EXERCISE2	40	50
	544	37.15	37.20	37.13	128	8	FAN	EXERCISE2	40	50
	545	37.15	37.21	37.14	127	8	FAN	EXERCISE2	40	50
	546	37.15	37.21	37.14	126	8	FAN	EXERCISE2	40	50
	547	37.15	37.20	37.13	126	8	FAN	EXERCISE2	40	50
	548	37.16	37.20	37.13	127	8	FAN	EXERCISE2	40	50
	549	37.16	37.22	37.13	127	8	FAN	EXERCISE2	40	50
	550	37.16	37.23	37.14	126	8	FAN	EXERCISE2	40	50
	551	37.16	37.23	37.14	127	8	FAN	EXERCISE2	40	50
	552	37.17	37.22	37.14	127	8	FAN	EXERCISE2	40	50
	553	37.18	37.19	37.14	131	8	FAN	EXERCISE2	40	50
	554	37.17	37.19	37.14	128	8	FAN	EXERCISE2	40	50
	555	37.17	37.23	37.14	130	8	FAN	EXERCISE2	40	50
	556	37.17	37.25	37.14	132	8	FAN	EXERCISE2	40	50
	557	37.17	37.25	37.14	127	8	FAN	EXERCISE2	40	50
	558	37.18	37.23	37.14	127	8	FAN	EXERCISE2	40	50
	559	37.18	37.23	37.14	127	8	FAN	EXERCISE2	40	50
	560	37.18	37.24	37.14	130	8	FAN	EXERCISE2	40	50
	561	37.18	37.22	37.14	131	8	FAN	EXERCISE2	40	50
	562	37.19	37.13	37.16	136	8	FAN	EXERCISE2	40	50
	563	37.19	37.15	37.16	132	8	FAN	EXERCISE2	40	50
	564	37.19	37.26	37.17	131	8	FAN	EXERCISE2	40	50
	565	37.19	37.27	37.18	131	8	FAN	EXERCISE2	40	50
	566	37.19	37.30	37.18	131	8	FAN	EXERCISE2	40	50
	567	37.19	37.32	37.18	131	8	FAN	EXERCISE2	40	50
	568	37.19	37.30	37.17	134	8	FAN	EXERCISE2	40	50
	569	37.20	37.31	37.17	130	8	FAN	EXERCISE2	40	50
	570	37.20	37.34	37.17	131	8	FAN	EXERCISE2	40	50
95	571	37.20	37.32	37.17	130	8	FAN	EXERCISE2	40	50
	572	37.21	37.28	37.17	131	8	FAN	EXERCISE2	40	50
	573	37.22	37.29	37.17	131	8	FAN	EXERCISE2	40	50
	574	37.21	37.28	37.16	132	8	FAN	EXERCISE2	40	50
	575	37.21	37.27	37.17	132	8	FAN	EXERCISE2	40	50
	576	37.21	37.29	37.18	130	8	FAN	EXERCISE2	40	50
	577	37.21	37.30	37.18	129	8	FAN	EXERCISE2	40	50
	578	37.22	37.26	37.18	133	8	FAN	EXERCISE2	40	50
	579	37.22	37.26	37.18	132	8	FAN	EXERCISE2	40	50
	580	37.22	37.32	37.18	133	8	FAN	EXERCISE2	40	50
	581	37.23	37.34	37.18	132	8	FAN	EXERCISE2	40	50
	582	37.23	37.31	37.18	132	8	FAN	EXERCISE2	40	50
	583	37.23	37.31	37.18	132	8	FAN	EXERCISE2	40	50

min	number	Tre	Tes	Tsk-head	HR	ID	condition	period	Ta	RH
	584	37.23	37.34	37.19	132	8	FAN	EXERCISE2	40	50
	585	37.23	37.34	37.20	134	8	FAN	EXERCISE2	40	50
	586	37.23	37.32	37.20	130	8	FAN	EXERCISE2	40	50
	587	37.23	37.32	37.20	130	8	FAN	EXERCISE2	40	50
	588	37.24	37.31	37.20	131	8	FAN	EXERCISE2	40	50
	589	37.25	37.31	37.20	131	8	FAN	EXERCISE2	40	50
	590	37.24	37.30	37.20	131	8	FAN	EXERCISE2	40	50
	591	37.24	37.30	37.20	134	8	FAN	EXERCISE2	40	50
	592	37.25	37.34	37.20	129	8	FAN	EXERCISE2	40	50
	593	37.25	37.36	37.20	130	8	FAN	EXERCISE2	40	50
	594	37.25	37.35	37.21	129	8	FAN	EXERCISE2	40	50
	595	37.25	37.25	37.21	132	8	FAN	EXERCISE2	40	50
	596	37.25	37.24	37.21	130	8	FAN	EXERCISE2	40	50
	597	37.26	37.34	37.21	131	8	FAN	EXERCISE2	40	50
	598	37.26	37.35	37.21	131	8	FAN	EXERCISE2	40	50
	599	37.26	37.34	37.20	133	8	FAN	EXERCISE2	40	50
	600	37.26	37.27	37.19	130	8	FAN	EXERCISE2	40	50
100	601	37.25	37.25	37.19	131	8	FAN	EXERCISE2	40	50
	602	37.26	37.31	37.20	130	8	FAN	EXERCISE2	40	50
	603	37.26	37.34	37.21	129	8	FAN	EXERCISE2	40	50
	604	37.27	37.35	37.21	134	8	FAN	EXERCISE2	40	50
	605	37.28	37.38	37.21	134	8	FAN	EXERCISE2	40	50
	606	37.27	37.37	37.20	136	8	FAN	EXERCISE2	40	50
	607	37.27	37.34	37.20	134	8	FAN	EXERCISE2	40	50
	608	37.28	37.33	37.20	132	8	FAN	EXERCISE2	40	50
	609	37.28	37.32	37.21	134	8	FAN	EXERCISE2	40	50
	610	37.28	37.31	37.22	133	8	FAN	EXERCISE2	40	50
	611	37.29	37.31	37.23	132	8	FAN	EXERCISE2	40	50
	612	37.29	37.33	37.24	134	8	FAN	EXERCISE2	40	50
	613	37.28	37.34	37.23	134	8	FAN	EXERCISE2	40	50
	614	37.28	37.34	37.24	134	8	FAN	EXERCISE2	40	50
	615	37.29	37.34	37.24	132	8	FAN	EXERCISE2	40	50
	616	37.29	37.31	37.24	133	8	FAN	EXERCISE2	40	50
	617	37.31	37.32	37.25	135	8	FAN	EXERCISE2	40	50
	618	37.31	37.32	37.25	134	8	FAN	EXERCISE2	40	50
	619	37.29	37.34	37.25	136	8	FAN	EXERCISE2	40	50
	620	37.29	37.38	37.25	137	8	FAN	EXERCISE2	40	50
	621	37.30	37.36	37.25	134	8	FAN	EXERCISE2	40	50
	622	37.30	37.38	37.25	134	8	FAN	EXERCISE2	40	50
	623	37.30	37.37	37.25	134	8	FAN	EXERCISE2	40	50
	624	37.31	37.34	37.25	131	8	FAN	EXERCISE2	40	50
	625	37.31	37.35	37.25	132	8	FAN	EXERCISE2	40	50
	626	37.30	37.37	37.25	132	8	FAN	EXERCISE2	40	50
	627	37.30	37.34	37.24	134	8	FAN	EXERCISE2	40	50
	628	37.29	37.32	37.23	133	8	FAN	EXERCISE2	40	50
	629	37.30	37.37	37.24	135	8	FAN	EXERCISE2	40	50

min	number	Tre	Tes	Tsk-head	HR	ID	condition	period	Ta	RH
	630	37.30	37.41	37.24	136	8	FAN	EXERCISE2	40	50
105	631	37.30	37.39	37.25	134	8	FAN	EXERCISE2	40	50
	632	37.31	37.36	37.25	137	8	FAN	EXERCISE2	40	50
	633	37.31	37.38	37.25	138	8	FAN	EXERCISE2	40	50
	634	37.31	37.42	37.26	137	8	FAN	EXERCISE2	40	50
	635	37.31	37.43	37.26	135	8	FAN	EXERCISE2	40	50
	636	37.31	37.38	37.26	136	8	FAN	EXERCISE2	40	50
	637	37.31	37.36	37.26	138	8	FAN	EXERCISE2	40	50
	638	37.32	37.37	37.25	137	8	FAN	EXERCISE2	40	50
	639	37.32	37.39	37.25	138	8	FAN	EXERCISE2	40	50
	640	37.33	37.41	37.26	135	8	FAN	EXERCISE2	40	50
	641	37.33	37.39	37.26	136	8	FAN	EXERCISE2	40	50
	642	37.33	37.37	37.26	135	8	FAN	EXERCISE2	40	50
	643	37.34	37.39	37.26	137	8	FAN	EXERCISE2	40	50
	644	37.35	37.39	37.27	134	8	FAN	REST3	28	50
	645	37.35	37.37	37.26	129	8	FAN	REST3	28	50
	646	37.36	37.37	37.27	135	8	FAN	REST3	28	50
	647	37.37	37.39	37.27	137	8	FAN	REST3	28	50
	648	37.37	37.41	37.12	131	8	FAN	REST3	28	50
	649	37.38	37.41	36.87	110	8	FAN	REST3	28	50
	650	37.38	37.42	36.74	112	8	FAN	REST3	28	50
	651	37.38	37.43	36.69	96	8	FAN	REST3	28	50
	652	37.39	37.44	36.65	96	8	FAN	REST3	28	50
	653	37.39	37.49	36.60	93	8	FAN	REST3	28	50
	654	37.39	37.52	36.56	93	8	FAN	REST3	28	50
	655	37.39	37.51	36.52	87	8	FAN	REST3	28	50
	656	37.39	37.50	36.47	84	8	FAN	REST3	28	50
	657	37.39	37.51	36.45	86	8	FAN	REST3	28	50
	658	37.38	37.50	36.37	93	8	FAN	REST3	28	50
	659	37.38	37.49	36.25	89	8	FAN	REST3	28	50
	660	37.38	37.50	36.20	86	8	FAN	REST3	28	50
110	661	37.39	37.50	36.16	93	8	FAN	REST3	28	50
	662	37.38	37.47	36.13	87	8	FAN	REST3	28	50
	663	37.38	37.45	36.10	90	8	FAN	REST3	28	50
	664	37.38	37.44	36.10	90	8	FAN	REST3	28	50
	665	37.37	37.44	36.10	92	8	FAN	REST3	28	50
	666	37.38	37.43	36.10	88	8	FAN	REST3	28	50
	667	37.37	37.38	36.09	83	8	FAN	REST3	28	50
	668	37.37	37.35	36.08	81	8	FAN	REST3	28	50
	669	37.37	37.35	36.06	82	8	FAN	REST3	28	50
	670	37.36	37.33	36.05	81	8	FAN	REST3	28	50
	671	37.36	37.33	36.05	80	8	FAN	REST3	28	50
	672	37.35	37.33	36.04	84	8	FAN	REST3	28	50
	673	37.35	37.30	36.03	79	8	FAN	REST3	28	50
	674	37.36	37.31	36.05	83	8	FAN	REST3	28	50
	675	37.35	37.31	36.04	80	8	FAN	REST3	28	50

min	number	Tre	Tes	Tsk-head	HR	ID	condition	period	Ta	RH
	676	37.35	37.28	35.98	79	8	FAN	REST3	28	50
	677	37.35	37.24	35.96	82	8	FAN	REST3	28	50
	678	37.35	37.23	35.96	81	8	FAN	REST3	28	50
	679	37.35	37.24	35.96	77	8	FAN	REST3	28	50
	680	37.35	37.22	35.92	86	8	FAN	REST3	28	50
	681	37.35	37.23	35.85	84	8	FAN	REST3	28	50
	682	37.35	37.18	35.81	86	8	FAN	REST3	28	50
	683	37.34	37.17	35.77	87	8	FAN	REST3	28	50
	684	37.34	37.22	35.70	86	8	FAN	REST3	28	50
	685	37.34	37.21	35.69	80	8	FAN	REST3	28	50
	686	37.33	37.21	35.73	76	8	FAN	REST3	28	50
	687	37.33	37.19	35.74	75	8	FAN	REST3	28	50
	688	37.33	37.18	35.75	75	8	FAN	REST3	28	50
	689	37.32	37.21	35.79	75	8	FAN	REST3	28	50
	690	37.32	37.23	35.80	77	8	FAN	REST3	28	50
115	691	37.32	37.22	35.79	79	8	FAN	REST3	28	50
	692	37.33	37.22	35.81	78	8	FAN	REST3	28	50
	693	37.32	37.22	35.84	78	8	FAN	REST3	28	50
	694	37.32	37.23	35.88	78	8	FAN	REST3	28	50
	695	37.32	37.23	35.89	79	8	FAN	REST3	28	50
	696	37.31	37.20	35.87	77	8	FAN	REST3	28	50
	697	37.32	37.21	35.87	76	8	FAN	REST3	28	50
	698	37.32	37.22	35.86	78	8	FAN	REST3	28	50
	699	37.31	37.22	35.81	82	8	FAN	REST3	28	50
	700	37.31	37.23	35.79	76	8	FAN	REST3	28	50
	701	37.32	37.22	35.78	76	8	FAN	REST3	28	50
	702	37.31	37.23	35.79	76	8	FAN	REST3	28	50
	703	37.31	37.23	35.84	79	8	FAN	REST3	28	50
	704	37.31	37.22	35.84	75	8	FAN	REST3	28	50
	705	37.31	37.23	35.84	75	8	FAN	REST3	28	50
	706	37.32	37.23	35.84	75	8	FAN	REST3	28	50
	707	37.31	37.23	35.80	74	8	FAN	REST3	28	50
	708	37.31	37.24	35.79	77	8	FAN	REST3	28	50
	709	37.31	37.24	35.77	72	8	FAN	REST3	28	50
0	1	36.92	36.76	34.92	81	9	FAN	REST1	28	50
	2	36.92	36.75	34.94	85	9	FAN	REST1	28	50
	3	36.91	36.72	34.93	78	9	FAN	REST1	28	50
	4	36.91	36.71	34.94	84	9	FAN	REST1	28	50
	5	36.92	36.75	34.97	86	9	FAN	REST1	28	50
	6	36.91	36.76	34.99	81	9	FAN	REST1	28	50
	7	36.91	36.75	35.01	84	9	FAN	REST1	28	50
	8	36.91	36.75	35.02	80	9	FAN	REST1	28	50
	9	36.91	36.73	35.03	83	9	FAN	REST1	28	50
	10	36.90	36.74	35.02	85	9	FAN	REST1	28	50
	11	36.89	36.77	34.99	89	9	FAN	REST1	28	50
	12	36.88	36.78	35.00	83	9	FAN	REST1	28	50

min	number	Tre	Tes	Tsk-head	HR	ID	condition	period	Ta	RH
	13	36.88	36.79	35.03	75	9	FAN	REST1	28	50
	14	36.88	36.79	35.04	77	9	FAN	REST1	28	50
	15	36.88	36.79	35.02	79	9	FAN	REST1	28	50
	16	36.88	36.79	34.99	79	9	FAN	REST1	28	50
	17	36.88	36.78	34.96	80	9	FAN	REST1	28	50
	18	36.87	36.77	34.95	75	9	FAN	REST1	28	50
	19	36.87	36.76	34.95	77	9	FAN	REST1	28	50
	20	36.88	36.76	34.95	79	9	FAN	REST1	28	50
	21	36.88	36.77	34.95	80	9	FAN	REST1	28	50
	22	36.88	36.77	34.97	77	9	FAN	REST1	28	50
	23	36.88	36.77	34.97	74	9	FAN	REST1	28	50
	24	36.89	36.79	34.97	81	9	FAN	REST1	28	50
	25	36.89	36.80	34.97	78	9	FAN	REST1	28	50
	26	36.89	36.79	34.97	78	9	FAN	REST1	28	50
	27	36.89	36.78	34.98	78	9	FAN	REST1	28	50
	28	36.90	36.79	34.98	78	9	FAN	REST1	28	50
	29	36.90	36.78	34.98	86	9	FAN	REST1	28	50
	30	36.90	36.71	34.98	85	9	FAN	REST1	28	50
5	31	36.91	36.69	34.96	81	9	FAN	REST1	28	50
	32	36.90	36.72	34.95	79	9	FAN	REST1	28	50
	33	36.90	36.72	34.94	75	9	FAN	REST1	28	50
	34	36.90	36.75	34.93	79	9	FAN	REST1	28	50
	35	36.90	36.76	34.93	76	9	FAN	REST1	28	50
	36	36.90	36.77	34.91	80	9	FAN	REST1	28	50
	37	36.90	36.77	34.90	78	9	FAN	REST1	28	50
	38	36.90	36.77	34.89	78	9	FAN	REST1	28	50
	39	36.90	36.75	34.88	79	9	FAN	REST1	28	50
	40	36.90	36.70	34.89	86	9	FAN	REST1	28	50
	41	36.89	36.71	34.88	79	9	FAN	REST1	28	50
	42	36.89	36.75	34.85	78	9	FAN	REST1	28	50
	43	36.88	36.74	34.84	77	9	FAN	REST1	28	50
	44	36.88	36.73	34.83	77	9	FAN	REST1	28	50
	45	36.88	36.73	34.81	80	9	FAN	REST1	28	50
	46	36.87	36.73	34.80	77	9	FAN	REST1	28	50
	47	36.87	36.74	34.79	76	9	FAN	REST1	28	50
	48	36.88	36.75	34.79	74	9	FAN	REST1	28	50
	49	36.88	36.75	34.78	76	9	FAN	REST1	28	50
	50	36.88	36.76	34.76	74	9	FAN	REST1	28	50
	51	36.88	36.77	34.74	75	9	FAN	REST1	28	50
	52	36.87	36.79	34.71	77	9	FAN	REST1	28	50
	53	36.87	36.78	34.70	76	9	FAN	REST1	28	50
	54	36.87	36.77	34.71	75	9	FAN	REST1	28	50
	55	36.88	36.76	34.71	72	9	FAN	REST1	28	50
	56	36.87	36.76	34.71	68	9	FAN	REST1	28	50
	57	36.85	36.76	34.70	72	9	FAN	REST1	28	50
	58	36.85	36.76	34.69	74	9	FAN	REST1	28	50

min	number	Tre	Tes	Tsk-head	HR	ID	condition	period	Ta	RH
	59	36.86	36.77	34.70	77	9	FAN	REST1	28	50
	60	36.86	36.79	34.69	72	9	FAN	REST1	28	50
10	61	36.86	36.79	34.67	72	9	FAN	REST1	28	50
	62	36.86	36.80	34.67	64	9	FAN	REST1	28	50
	63	36.86	36.79	34.67	71	9	FAN	REST1	28	50
	64	36.86	36.79	34.68	75	9	FAN	REST1	28	50
	65	36.86	36.79	34.69	71	9	FAN	REST1	28	50
	66	36.86	36.78	34.70	80	9	FAN	REST1	28	50
	67	36.86	36.74	34.73	87	9	FAN	REST1	28	50
	68	36.86	36.70	34.76	76	9	FAN	REST1	28	50
	69	36.85	36.71	34.78	74	9	FAN	REST1	28	50
	70	36.84	36.72	34.79	72	9	FAN	REST1	28	50
	71	36.83	36.74	34.79	72	9	FAN	REST1	28	50
	72	36.82	36.76	34.80	75	9	FAN	REST1	28	50
	73	36.81	36.75	34.79	66	9	FAN	REST1	28	50
	74	36.80	36.76	34.78	73	9	FAN	REST1	28	50
	75	36.80	36.76	34.77	76	9	FAN	REST1	28	50
	76	36.79	36.76	34.77	72	9	FAN	REST1	28	50
	77	36.79	36.65	34.78	89	9	FAN	REST1	28	50
	78	36.81	36.61	34.78	76	9	FAN	REST1	28	50
	79	36.81	36.71	34.77	70	9	FAN	REST1	28	50
	80	36.81	36.73	34.76	71	9	FAN	REST1	28	50
	81	36.82	36.74	34.77	70	9	FAN	REST1	28	50
	82	36.83	36.73	34.78	70	9	FAN	REST1	28	50
	83	36.83	36.74	34.78	71	9	FAN	REST1	28	50
	84	36.82	36.75	34.78	69	9	FAN	REST1	28	50
	85	36.82	36.76	34.77	68	9	FAN	REST1	28	50
	86	36.82	36.75	34.76	72	9	FAN	REST1	28	50
	87	36.83	36.73	34.76	68	9	FAN	REST1	28	50
	88	36.82	36.73	34.75	66	9	FAN	REST1	28	50
	89	36.82	36.73	34.74	70	9	FAN	REST1	28	50
	90	36.82	36.73	34.74	67	9	FAN	REST1	28	50
15	91	36.81	36.72	34.72	67	9	FAN	REST1	28	50
	92	36.81	36.72	34.70	71	9	FAN	REST1	28	50
	93	36.81	36.72	34.70	69	9	FAN	REST1	28	50
	94	36.81	36.73	34.70	68	9	FAN	REST1	28	50
	95	36.81	36.76	34.70	69	9	FAN	REST1	28	50
	96	36.81	36.76	34.70	79	9	FAN	REST1	28	50
	97	36.80	36.75	34.69	74	9	FAN	REST1	28	50
	98	36.80	36.75	34.69	76	9	FAN	REST1	28	50
	99	36.81	36.75	34.71	72	9	FAN	REST1	28	50
	100	36.81	36.75	34.72	76	9	FAN	REST1	28	50
	101	36.81	36.75	34.72	80	9	FAN	REST1	28	50
	102	36.81	36.73	34.73	80	9	FAN	REST1	28	50
	103	36.81	36.75	34.77	81	9	FAN	REST1	40	50
	104	36.81	36.75	34.85	96	9	FAN	REST1	40	50

min	number	Tre	Tes	Tsk-head	HR	ID	condition	period	Ta	RH
	105	36.81	36.71	34.95	99	9	FAN	REST1	40	50
	106	36.80	36.71	35.07	90	9	FAN	REST1	40	50
	107	36.80	36.71	35.19	80	9	FAN	REST1	40	50
	108	36.79	36.69	35.27	82	9	FAN	REST1	40	50
	109	36.78	36.62	35.33	87	9	FAN	REST1	40	50
	110	36.78	36.63	35.40	91	9	FAN	REST1	40	50
	111	36.78	36.67	35.46	83	9	FAN	REST1	40	50
	112	36.77	36.66	35.52	82	9	FAN	REST1	40	50
	113	36.76	36.67	35.53	78	9	FAN	REST1	40	50
	114	36.76	36.63	35.58	78	9	FAN	REST1	40	50
	115	36.75	36.61	35.68	77	9	FAN	REST1	40	50
	116	36.75	36.64	35.70	76	9	FAN	REST1	40	50
	117	36.75	36.66	35.74	101	9	FAN	REST1	40	50
	118	36.75	36.62	35.78	85	9	FAN	REST1	40	50
	119	36.75	36.61	35.80	87	9	FAN	REST1	40	50
	120	36.76	36.64	35.81	90	9	FAN	REST1	40	50
20	121	36.76	36.65	35.84	93	9	FAN	REST1	40	50
	122	36.75	36.64	35.88	85	9	FAN	REST1	40	50
	123	36.75	36.60	35.92	83	9	FAN	REST1	40	50
	124	36.74	36.66	35.94	85	9	FAN	REST1	40	50
	125	36.74	36.64	35.95	83	9	FAN	REST1	40	50
	126	36.73	36.56	35.99	77	9	FAN	REST1	40	50
	127	36.72	36.55	36.01	83	9	FAN	REST1	40	50
	128	36.71	36.56	36.01	79	9	FAN	REST1	40	50
	129	36.71	36.55	36.03	88	9	FAN	REST1	40	50
	130	36.71	36.53	36.05	88	9	FAN	REST1	40	50
	131	36.70	36.54	36.06	85	9	FAN	REST1	40	50
	132	36.70	36.57	36.07	81	9	FAN	REST1	40	50
	133	36.70	36.59	36.08	81	9	FAN	REST1	40	50
	134	36.70	36.56	36.09	97	9	FAN	REST1	40	50
	135	36.71	36.55	36.11	101	9	FAN	REST1	40	50
	136	36.71	36.53	36.12	86	9	FAN	REST1	40	50
	137	36.71	36.53	36.13	82	9	FAN	REST1	40	50
	138	36.70	36.55	36.13	82	9	FAN	REST1	40	50
	139	36.70	36.58	36.15	82	9	FAN	EXERCISE1	40	50
	140	36.70	36.59	36.16	84	9	FAN	EXERCISE1	40	50
	141	36.70	36.60	36.15	93	9	FAN	EXERCISE1	40	50
	142	36.70	36.61	36.15	96	9	FAN	EXERCISE1	40	50
	143	36.69	36.60	36.16	101	9	FAN	EXERCISE1	40	50
	144	36.69	36.59	36.15	104	9	FAN	EXERCISE1	40	50
	145	36.70	36.61	36.15	104	9	FAN	EXERCISE1	40	50
	146	36.70	36.62	36.16	103	9	FAN	EXERCISE1	40	50
	147	36.70	36.60	36.16	107	9	FAN	EXERCISE1	40	50
	148	36.71	36.58	36.15	100	9	FAN	EXERCISE1	40	50
	149	36.71	36.55	36.14	104	9	FAN	EXERCISE1	40	50
	150	36.71	36.57	36.13	103	9	FAN	EXERCISE1	40	50

min	number	Tre	Tes	Tsk-head	HR	ID	condition	period	Ta	RH
25	151	36.71	36.60	36.14	104	9	FAN	EXERCISE1	40	50
	152	36.71	36.61	36.15	103	9	FAN	EXERCISE1	40	50
	153	36.71	36.59	36.14	108	9	FAN	EXERCISE1	40	50
	154	36.71	36.59	36.14	107	9	FAN	EXERCISE1	40	50
	155	36.71	36.61	36.16	106	9	FAN	EXERCISE1	40	50
	156	36.71	36.61	36.16	105	9	FAN	EXERCISE1	40	50
	157	36.71	36.62	36.17	106	9	FAN	EXERCISE1	40	50
	158	36.72	36.64	36.18	109	9	FAN	EXERCISE1	40	50
	159	36.72	36.65	36.18	109	9	FAN	EXERCISE1	40	50
	160	36.72	36.67	36.20	110	9	FAN	EXERCISE1	40	50
	161	36.72	36.67	36.21	109	9	FAN	EXERCISE1	40	50
	162	36.72	36.65	36.22	110	9	FAN	EXERCISE1	40	50
	163	36.73	36.66	36.23	112	9	FAN	EXERCISE1	40	50
	164	36.73	36.67	36.24	115	9	FAN	EXERCISE1	40	50
	165	36.74	36.68	36.26	111	9	FAN	EXERCISE1	40	50
	166	36.74	36.69	36.28	109	9	FAN	EXERCISE1	40	50
	167	36.74	36.71	36.29	108	9	FAN	EXERCISE1	40	50
	168	36.75	36.72	36.30	110	9	FAN	EXERCISE1	40	50
	169	36.75	36.71	36.31	109	9	FAN	EXERCISE1	40	50
	170	36.76	36.71	36.32	109	9	FAN	EXERCISE1	40	50
	171	36.76	36.72	36.32	108	9	FAN	EXERCISE1	40	50
	172	36.76	36.73	36.32	108	9	FAN	EXERCISE1	40	50
	173	36.76	36.75	36.32	105	9	FAN	EXERCISE1	40	50
	174	36.76	36.78	36.31	112	9	FAN	EXERCISE1	40	50
	175	36.77	36.80	36.31	112	9	FAN	EXERCISE1	40	50
	176	36.78	36.80	36.33	114	9	FAN	EXERCISE1	40	50
	177	36.78	36.80	36.33	115	9	FAN	EXERCISE1	40	50
	178	36.78	36.81	36.33	111	9	FAN	EXERCISE1	40	50
	179	36.79	36.81	36.34	114	9	FAN	EXERCISE1	40	50
	180	36.79	36.83	36.35	116	9	FAN	EXERCISE1	40	50
30	181	36.79	36.83	36.35	115	9	FAN	EXERCISE1	40	50
	182	36.80	36.83	36.35	115	9	FAN	EXERCISE1	40	50
	183	36.80	36.83	36.35	114	9	FAN	EXERCISE1	40	50
	184	36.81	36.83	36.32	117	9	FAN	EXERCISE1	40	50
	185	36.81	36.83	36.29	117	9	FAN	EXERCISE1	40	50
	186	36.80	36.81	36.27	114	9	FAN	EXERCISE1	40	50
	187	36.81	36.80	36.26	115	9	FAN	EXERCISE1	40	50
	188	36.81	36.80	36.26	115	9	FAN	EXERCISE1	40	50
	189	36.82	36.79	36.28	117	9	FAN	EXERCISE1	40	50
	190	36.82	36.81	36.31	113	9	FAN	EXERCISE1	40	50
	191	36.82	36.82	36.32	114	9	FAN	EXERCISE1	40	50
	192	36.83	36.84	36.33	118	9	FAN	EXERCISE1	40	50
	193	36.83	36.87	36.33	123	9	FAN	EXERCISE1	40	50
	194	36.83	36.89	36.34	125	9	FAN	EXERCISE1	40	50
	195	36.84	36.89	36.34	120	9	FAN	EXERCISE1	40	50
	196	36.84	36.88	36.33	117	9	FAN	EXERCISE1	40	50

min	number	Tre	Tes	Tsk-head	HR	ID	condition	period	Ta	RH
	197	36.84	36.88	36.32	116	9	FAN	EXERCISE1	40	50
	198	36.84	36.84	36.32	118	9	FAN	EXERCISE1	40	50
	199	36.84	36.83	36.31	114	9	FAN	EXERCISE1	40	50
	200	36.84	36.84	36.31	113	9	FAN	EXERCISE1	40	50
	201	36.85	36.84	36.31	113	9	FAN	EXERCISE1	40	50
	202	36.85	36.85	36.31	117	9	FAN	EXERCISE1	40	50
	203	36.85	36.84	36.32	119	9	FAN	EXERCISE1	40	50
	204	36.86	36.87	36.33	124	9	FAN	EXERCISE1	40	50
	205	36.87	36.87	36.34	124	9	FAN	EXERCISE1	40	50
	206	36.86	36.85	36.35	123	9	FAN	EXERCISE1	40	50
	207	36.86	36.86	36.34	122	9	FAN	EXERCISE1	40	50
	208	36.87	36.87	36.34	117	9	FAN	EXERCISE1	40	50
	209	36.87	36.87	36.35	114	9	FAN	EXERCISE1	40	50
	210	36.87	36.90	36.34	115	9	FAN	EXERCISE1	40	50
35	211	36.87	36.91	36.33	117	9	FAN	EXERCISE1	40	50
	212	36.87	36.91	36.33	115	9	FAN	EXERCISE1	40	50
	213	36.87	36.89	36.34	113	9	FAN	EXERCISE1	40	50
	214	36.87	36.88	36.33	115	9	FAN	EXERCISE1	40	50
	215	36.88	36.74	36.32	123	9	FAN	EXERCISE1	40	50
	216	36.88	36.72	36.32	123	9	FAN	EXERCISE1	40	50
	217	36.89	36.86	36.32	119	9	FAN	EXERCISE1	40	50
	218	36.89	36.91	36.34	117	9	FAN	EXERCISE1	40	50
	219	36.89	36.92	36.35	118	9	FAN	EXERCISE1	40	50
	220	36.89	36.91	36.35	117	9	FAN	EXERCISE1	40	50
	221	36.88	36.93	36.34	118	9	FAN	EXERCISE1	40	50
	222	36.88	36.93	36.34	115	9	FAN	EXERCISE1	40	50
	223	36.89	36.94	36.37	114	9	FAN	EXERCISE1	40	50
	224	36.89	36.96	36.40	118	9	FAN	EXERCISE1	40	50
	225	36.89	36.96	36.42	121	9	FAN	EXERCISE1	40	50
	226	36.89	36.95	36.44	123	9	FAN	EXERCISE1	40	50
	227	36.89	36.92	36.44	124	9	FAN	EXERCISE1	40	50
	228	36.89	36.93	36.45	121	9	FAN	EXERCISE1	40	50
	229	36.90	36.94	36.45	119	9	FAN	EXERCISE1	40	50
	230	36.90	36.92	36.43	120	9	FAN	EXERCISE1	40	50
	231	36.91	36.95	36.43	121	9	FAN	EXERCISE1	40	50
	232	36.91	37.02	36.42	122	9	FAN	EXERCISE1	40	50
	233	36.91	37.05	36.41	121	9	FAN	EXERCISE1	40	50
	234	36.92	36.99	36.40	115	9	FAN	EXERCISE1	40	50
	235	36.92	36.94	36.40	115	9	FAN	EXERCISE1	40	50
	236	36.92	36.94	36.39	115	9	FAN	EXERCISE1	40	50
	237	36.92	36.94	36.38	119	9	FAN	EXERCISE1	40	50
	238	36.93	36.96	36.38	119	9	FAN	EXERCISE1	40	50
	239	36.94	36.94	36.36	119	9	FAN	EXERCISE1	40	50
	240	36.94	36.91	36.35	118	9	FAN	EXERCISE1	40	50
40	241	36.94	36.92	36.34	120	9	FAN	EXERCISE1	40	50
	242	36.95	36.93	36.32	118	9	FAN	EXERCISE1	40	50

min	number	Tre	Tes	Tsk-head	HR	ID	condition	period	Ta	RH
	243	36.95	36.94	36.33	119	9	FAN	EXERCISE1	40	50
	244	36.95	36.95	36.34	116	9	FAN	EXERCISE1	40	50
	245	36.95	36.95	36.35	118	9	FAN	EXERCISE1	40	50
	246	36.96	36.97	36.36	118	9	FAN	EXERCISE1	40	50
	247	36.96	36.97	36.37	125	9	FAN	EXERCISE1	40	50
	248	36.96	36.90	36.38	131	9	FAN	EXERCISE1	40	50
	249	36.96	36.91	36.40	131	9	FAN	EXERCISE1	40	50
	250	36.97	36.98	36.41	130	9	FAN	EXERCISE1	40	50
	251	36.98	36.99	36.42	128	9	FAN	EXERCISE1	40	50
	252	36.98	36.98	36.43	125	9	FAN	EXERCISE1	40	50
	253	36.98	37.00	36.43	124	9	FAN	EXERCISE1	40	50
	254	36.98	37.00	36.42	121	9	FAN	EXERCISE1	40	50
	255	36.98	37.00	36.41	119	9	FAN	EXERCISE1	40	50
	256	36.99	37.01	36.42	119	9	FAN	EXERCISE1	40	50
	257	36.99	37.00	36.44	119	9	FAN	EXERCISE1	40	50
	258	36.99	37.00	36.44	118	9	FAN	EXERCISE1	40	50
	259	37.00	37.04	36.45	115	9	FAN	EXERCISE1	40	50
	260	37.00	37.04	36.45	118	9	FAN	EXERCISE1	40	50
	261	37.00	37.01	36.45	118	9	FAN	EXERCISE1	40	50
	262	37.00	37.03	36.46	119	9	FAN	EXERCISE1	40	50
	263	37.00	37.05	36.46	118	9	FAN	EXERCISE1	40	50
	264	37.01	37.06	36.46	121	9	FAN	EXERCISE1	40	50
	265	37.01	37.06	36.47	121	9	FAN	EXERCISE1	40	50
	266	37.02	37.07	36.47	123	9	FAN	EXERCISE1	40	50
	267	37.02	37.09	36.47	123	9	FAN	EXERCISE1	40	50
	268	37.02	37.08	36.48	123	9	FAN	EXERCISE1	40	50
	269	37.03	37.08	36.49	122	9	FAN	EXERCISE1	40	50
	270	37.03	37.07	36.49	121	9	FAN	EXERCISE1	40	50
45	271	37.03	37.07	36.49	123	9	FAN	EXERCISE1	40	50
	272	37.03	37.09	36.49	124	9	FAN	EXERCISE1	40	50
	273	37.02	37.07	36.48	124	9	FAN	EXERCISE1	40	50
	274	37.03	37.06	36.49	123	9	FAN	EXERCISE1	40	50
	275	37.04	37.09	36.49	123	9	FAN	EXERCISE1	40	50
	276	37.04	37.08	36.49	121	9	FAN	EXERCISE1	40	50
	277	37.05	37.05	36.50	118	9	FAN	EXERCISE1	40	50
	278	37.05	37.06	36.50	121	9	FAN	EXERCISE1	40	50
	279	37.05	37.10	36.50	122	9	FAN	EXERCISE1	40	50
	280	37.05	37.11	36.50	123	9	FAN	EXERCISE1	40	50
	281	37.05	37.08	36.50	126	9	FAN	EXERCISE1	40	50
	282	37.06	37.07	36.51	125	9	FAN	EXERCISE1	40	50
	283	37.06	36.92	36.51	127	9	FAN	EXERCISE1	40	50
	284	37.06	36.89	36.51	126	9	FAN	EXERCISE1	40	50
	285	37.07	37.06	36.52	125	9	FAN	EXERCISE1	40	50
	286	37.07	37.06	36.52	122	9	FAN	EXERCISE1	40	50
	287	37.06	37.08	36.52	125	9	FAN	EXERCISE1	40	50
	288	37.07	37.09	36.52	128	9	FAN	EXERCISE1	40	50

min	number	Tre	Tes	Tsk-head	HR	ID	condition	period	Ta	RH
	289	37.07	37.09	36.53	130	9	FAN	EXERCISE1	40	50
	290	37.07	37.10	36.51	128	9	FAN	EXERCISE1	40	50
	291	37.07	37.09	36.51	128	9	FAN	EXERCISE1	40	50
	292	37.08	37.09	36.52	131	9	FAN	EXERCISE1	40	50
	293	37.08	37.09	36.52	128	9	FAN	EXERCISE1	40	50
	294	37.09	37.11	36.52	128	9	FAN	EXERCISE1	40	50
	295	37.10	37.11	36.53	123	9	FAN	EXERCISE1	40	50
	296	37.10	37.12	36.54	121	9	FAN	EXERCISE1	40	50
	297	37.10	37.14	36.54	124	9	FAN	EXERCISE1	40	50
	298	37.10	37.15	36.54	126	9	FAN	EXERCISE1	40	50
	299	37.11	37.15	36.55	129	9	FAN	EXERCISE1	40	50
	300	37.11	37.16	36.56	131	9	FAN	EXERCISE1	40	50
50	301	37.11	37.17	36.55	133	9	FAN	EXERCISE1	40	50
	302	37.11	37.14	36.55	132	9	FAN	EXERCISE1	40	50
	303	37.12	37.14	36.56	132	9	FAN	EXERCISE1	40	50
	304	37.12	37.18	36.56	128	9	FAN	EXERCISE1	40	50
	305	37.11	37.14	36.56	126	9	FAN	EXERCISE1	40	50
	306	37.12	37.13	36.58	125	9	FAN	EXERCISE1	40	50
	307	37.12	37.18	36.58	124	9	FAN	EXERCISE1	40	50
	308	37.12	37.17	36.58	124	9	FAN	EXERCISE1	40	50
	309	37.13	37.14	36.58	123	9	FAN	EXERCISE1	40	50
	310	37.13	37.08	36.58	125	9	FAN	EXERCISE1	40	50
	311	37.14	37.06	36.57	125	9	FAN	EXERCISE1	40	50
	312	37.14	37.10	36.57	125	9	FAN	EXERCISE1	40	50
	313	37.14	37.13	36.56	124	9	FAN	EXERCISE1	40	50
	314	37.14	37.13	36.55	123	9	FAN	EXERCISE1	40	50
	315	37.15	37.12	36.55	124	9	FAN	EXERCISE1	40	50
	316	37.14	37.11	36.56	125	9	FAN	EXERCISE1	40	50
	317	37.15	37.13	36.56	126	9	FAN	EXERCISE1	40	50
	318	37.16	37.15	36.57	125	9	FAN	EXERCISE1	40	50
	319	37.16	37.17	36.57	125	9	FAN	EXERCISE1	40	50
	320	37.15	37.17	36.56	122	9	FAN	REST2	28	50
	321	37.14	37.13	36.56	120	9	FAN	REST2	28	50
	322	37.15	37.15	36.45	115	9	FAN	REST2	28	50
	323	37.16	37.17	36.30	108	9	FAN	REST2	28	50
	324	37.17	37.19	36.05	105	9	FAN	REST2	28	50
	325	37.17	37.20	35.69	100	9	FAN	REST2	28	50
	326	37.17	37.17	35.48	89	9	FAN	REST2	28	50
	327	37.18	37.22	35.30	95	9	FAN	REST2	28	50
	328	37.17	37.25	35.01	91	9	FAN	REST2	28	50
	329	37.17	37.11	34.64	92	9	FAN	REST2	28	50
	330	37.17	37.05	34.40	94	9	FAN	REST2	28	50
55	331	37.17	37.18	34.38	90	9	FAN	REST2	28	50
	332	37.16	37.21	34.48	96	9	FAN	REST2	28	50
	333	37.17	37.20	34.56	89	9	FAN	REST2	28	50
	334	37.17	37.22	34.48	86	9	FAN	REST2	28	50

min	number	Tre	Tes	Tsk-head	HR	ID	condition	period	Ta	RH
	335	37.16	37.22	34.33	84	9	FAN	REST2	28	50
	336	37.16	37.18	34.27	83	9	FAN	REST2	28	50
	337	37.16	37.16	34.18	83	9	FAN	REST2	28	50
	338	37.17	37.15	34.07	85	9	FAN	REST2	28	50
	339	37.16	31.29	34.12	93	9	FAN	REST2	28	50
	340	37.16	27.02	34.19	97	9	FAN	REST2	28	50
	341	37.15	27.75	34.22	81	9	FAN	REST2	28	50
	342	37.15	27.81	34.24	86	9	FAN	REST2	28	50
	343	37.16	30.46	34.16	84	9	FAN	REST2	28	50
	344	37.15	32.57	34.15	86	9	FAN	REST2	28	50
	345	37.15	33.18	34.23	85	9	FAN	REST2	28	50
	346	37.16	30.77	34.29	86	9	FAN	REST2	28	50
	347	37.15	30.17	34.30	88	9	FAN	REST2	28	50
	348	37.14	32.08	34.39	84	9	FAN	REST2	28	50
	349	37.14	30.85	34.46	88	9	FAN	REST2	28	50
	350	37.14	30.64	34.53	88	9	FAN	REST2	28	50
	351	37.13	29.85	34.58	86	9	FAN	REST2	28	50
	352	37.13	30.20	34.65	88	9	FAN	REST2	28	50
	353	37.13	32.49	34.83	81	9	FAN	REST2	28	50
	354	37.12	32.95	34.98	82	9	FAN	REST2	28	50
	355	37.12	33.40	35.13	79	9	FAN	REST2	28	50
	356	37.11	33.77	35.25	73	9	FAN	REST2	28	50
	357	37.11	34.12	35.29	85	9	FAN	REST2	28	50
	358	37.12	31.86	35.28	88	9	FAN	REST2	28	50
	359	37.11	30.64	35.29	85	9	FAN	REST2	28	50
	360	37.11	32.50	35.32	86	9	FAN	REST2	28	50
60	361	37.11	33.48	35.35	83	9	FAN	REST2	28	50
	362	37.12	34.14	35.35	83	9	FAN	REST2	28	50
	363	37.11	34.51	35.36	81	9	FAN	REST2	28	50
	364	37.11	34.82	35.35	81	9	FAN	REST2	28	50
	365	37.11	31.47	35.34	83	9	FAN	REST2	28	50
	366	37.11	29.59	35.32	85	9	FAN	REST2	28	50
	367	37.11	32.03	35.27	83	9	FAN	REST2	28	50
	368	37.11	33.03	35.25	78	9	FAN	REST2	28	50
	369	37.11	33.84	35.23	84	9	FAN	REST2	28	50
	370	37.10	34.40	35.23	78	9	FAN	REST2	28	50
	371	37.10	34.54	35.29	79	9	FAN	REST2	28	50
	372	37.10	34.42	35.32	83	9	FAN	REST2	28	50
	373	37.10	34.38	35.31	83	9	FAN	REST2	28	50
	374	37.11	30.45	35.25	89	9	FAN	REST2	28	50
	375	37.11	28.26	35.20	97	9	FAN	REST2	28	50
	376	37.12	31.22	35.20	88	9	FAN	REST2	28	50
	377	37.12	32.86	35.17	89	9	FAN	REST2	28	50
	378	37.11	33.44	35.14	87	9	FAN	REST2	28	50
	379	37.11	33.62	35.12	77	9	FAN	REST2	28	50
	380	37.12	33.43	35.10	85	9	FAN	REST2	28	50

min	number	Tre	Tes	Tsk-head	HR	ID	condition	period	Ta	RH
	381	37.12	33.55	35.11	78	9	FAN	REST2	28	50
	382	37.12	34.27	35.14	82	9	FAN	REST2	28	50
	383	37.12	32.60	35.18	88	9	FAN	REST2	28	50
	384	37.12	31.77	35.22	79	9	FAN	REST2	28	50
	385	37.11	33.06	35.17	80	9	FAN	REST2	28	50
	386	37.11	33.69	35.14	82	9	FAN	REST2	28	50
	387	37.11	34.31	35.19	81	9	FAN	REST2	28	50
	388	37.11	34.75	35.18	76	9	FAN	REST2	28	50
	389	37.11	34.91	35.18	90	9	FAN	REST2	28	50
	390	37.11	34.78	35.17	96	9	FAN	REST2	28	50
65	391	37.11	35.02	35.12	81	9	FAN	REST2	28	50
	392	37.11	35.41	35.12	78	9	FAN	REST2	28	50
	393	37.10	35.64	35.14	76	9	FAN	REST2	28	50
	394	37.10	35.70	35.13	82	9	FAN	REST2	28	50
	395	37.10	32.74	35.14	86	9	FAN	REST2	28	50
	396	37.10	31.31	35.15	87	9	FAN	REST2	28	50
	397	37.10	33.07	35.16	77	9	FAN	REST2	28	50
	398	37.10	33.84	35.16	81	9	FAN	REST2	28	50
	399	37.10	34.53	35.14	80	9	FAN	REST2	28	50
	400	37.10	33.96	35.11	85	9	FAN	REST2	28	50
	401	37.10	33.98	35.13	84	9	FAN	REST2	28	50
	402	37.10	34.92	35.13	79	9	FAN	REST2	28	50
	403	37.10	35.03	35.05	84	9	FAN	REST2	28	50
	404	37.10	35.18	35.01	78	9	FAN	REST2	28	50
	405	37.09	35.38	35.03	84	9	FAN	REST2	28	50
	406	37.09	35.50	35.03	81	9	FAN	REST2	28	50
	407	37.10	35.59	35.06	93	9	FAN	REST2	28	50
	408	37.10	35.87	35.10	81	9	FAN	REST2	28	50
	409	37.10	36.02	35.09	78	9	FAN	REST2	28	50
	410	37.10	36.00	35.05	86	9	FAN	REST2	28	50
	411	37.10	36.09	34.83	79	9	FAN	REST2	28	50
	412	37.11	36.12	34.57	77	9	FAN	REST2	28	50
	413	37.12	36.14	34.45	83	9	FAN	REST2	28	50
	414	37.11	36.23	34.34	90	9	FAN	REST2	28	50
	415	37.11	36.32	34.25	88	9	FAN	REST2	28	50
	416	37.12	36.36	34.16	84	9	FAN	REST2	28	50
	417	37.13	36.39	34.07	83	9	FAN	REST2	28	50
	418	37.12	36.43	33.97	76	9	FAN	REST2	28	50
	419	37.12	36.48	33.91	76	9	FAN	REST2	28	50
	420	37.12	36.51	33.91	85	9	FAN	REST2	28	50
70	421	37.12	36.51	33.95	80	9	FAN	REST2	28	50
	422	37.12	36.52	33.96	75	9	FAN	REST2	28	50
	423	37.11	36.55	33.91	71	9	FAN	REST2	28	50
	424	37.11	36.58	33.90	82	9	FAN	REST2	28	50
	425	37.11	36.61	33.86	72	9	FAN	REST2	28	50
	426	37.11	36.64	33.85	73	9	FAN	REST2	28	50

min	number	Tre	Tes	Tsk-head	HR	ID	condition	period	Ta	RH
	427	37.11	36.68	33.87	88	9	FAN	REST2	28	50
	428	37.11	36.70	33.90	82	9	FAN	REST2	28	50
	429	37.11	36.69	33.87	79	9	FAN	REST2	28	50
	430	37.12	36.70	33.87	78	9	FAN	REST2	28	50
	431	37.12	36.73	33.86	80	9	FAN	REST2	28	50
	432	37.12	36.76	33.83	80	9	FAN	REST2	28	50
	433	37.13	36.78	33.83	77	9	FAN	REST2	28	50
	434	37.13	36.80	33.84	76	9	FAN	REST2	28	50
	435	37.13	36.81	33.87	73	9	FAN	REST2	28	50
	436	37.12	36.83	33.84	72	9	FAN	REST2	28	50
	437	37.11	36.36	33.81	81	9	FAN	REST2	28	50
	438	37.11	36.26	33.86	86	9	FAN	REST2	28	50
	439	37.12	36.67	33.91	89	9	FAN	REST2	40	50
	440	37.12	36.73	34.07	100	9	FAN	REST2	40	50
	441	37.14	36.73	34.30	95	9	FAN	REST2	40	50
	442	37.14	36.74	34.42	92	9	FAN	REST2	40	50
	443	37.13	36.79	34.53	87	9	FAN	REST2	40	50
	444	37.12	36.81	34.50	78	9	FAN	REST2	40	50
	445	37.12	36.82	34.61	77	9	FAN	REST2	40	50
	446	37.13	36.83	35.01	84	9	FAN	REST2	40	50
	447	37.13	36.78	35.25	84	9	FAN	REST2	40	50
	448	37.13	36.77	35.36	81	9	FAN	REST2	40	50
	449	37.13	36.83	35.44	81	9	FAN	REST2	40	50
	450	37.13	36.82	35.51	80	9	FAN	REST2	40	50
75	451	37.13	36.82	35.56	83	9	FAN	REST2	40	50
	452	37.12	36.84	35.60	88	9	FAN	REST2	40	50
	453	37.12	36.84	35.66	81	9	FAN	REST2	40	50
	454	37.12	36.83	35.70	80	9	FAN	REST2	40	50
	455	37.12	36.84	35.73	80	9	FAN	REST2	40	50
	456	37.11	36.84	35.76	82	9	FAN	REST2	40	50
	457	37.10	36.82	35.79	81	9	FAN	REST2	40	50
	458	37.11	36.83	35.80	100	9	FAN	REST2	40	50
	459	37.11	36.83	35.80	99	9	FAN	REST2	40	50
	460	37.11	36.81	35.82	93	9	FAN	REST2	40	50
	461	37.12	36.83	35.87	93	9	FAN	REST2	40	50
	462	37.12	36.83	35.89	90	9	FAN	REST2	40	50
	463	37.12	36.78	35.90	83	9	FAN	EXERCISE2	40	50
	464	37.12	36.76	35.94	84	9	FAN	EXERCISE2	40	50
	465	37.13	36.79	35.96	90	9	FAN	EXERCISE2	40	50
	466	37.12	36.80	35.97	97	9	FAN	EXERCISE2	40	50
	467	37.11	36.79	35.99	100	9	FAN	EXERCISE2	40	50
	468	37.11	36.79	36.00	105	9	FAN	EXERCISE2	40	50
	469	37.12	36.80	36.00	107	9	FAN	EXERCISE2	40	50
	470	37.11	36.79	35.99	107	9	FAN	EXERCISE2	40	50
	471	37.11	36.79	36.01	108	9	FAN	EXERCISE2	40	50
	472	37.11	36.80	36.03	105	9	FAN	EXERCISE2	40	50

min	number	Tre	Tes	Tsk-head	HR	ID	condition	period	Ta	RH
	473	37.11	36.79	36.06	106	9	FAN	EXERCISE2	40	50
	474	37.10	36.80	36.08	107	9	FAN	EXERCISE2	40	50
	475	37.10	36.80	36.10	106	9	FAN	EXERCISE2	40	50
	476	37.09	36.79	36.12	106	9	FAN	EXERCISE2	40	50
	477	37.09	36.81	36.13	105	9	FAN	EXERCISE2	40	50
	478	37.09	36.82	36.15	106	9	FAN	EXERCISE2	40	50
	479	37.08	36.83	36.17	108	9	FAN	EXERCISE2	40	50
	480	37.09	36.85	36.20	110	9	FAN	EXERCISE2	40	50
80	481	37.09	36.87	36.21	112	9	FAN	EXERCISE2	40	50
	482	37.08	36.88	36.21	108	9	FAN	EXERCISE2	40	50
	483	37.08	36.88	36.21	108	9	FAN	EXERCISE2	40	50
	484	37.08	36.89	36.21	109	9	FAN	EXERCISE2	40	50
	485	37.07	36.91	36.23	109	9	FAN	EXERCISE2	40	50
	486	37.07	36.92	36.25	111	9	FAN	EXERCISE2	40	50
	487	37.07	36.92	36.27	111	9	FAN	EXERCISE2	40	50
	488	37.07	36.64	36.31	114	9	FAN	EXERCISE2	40	50
	489	37.08	36.56	36.34	110	9	FAN	EXERCISE2	40	50
	490	37.08	36.79	36.34	109	9	FAN	EXERCISE2	40	50
	491	37.08	36.85	36.37	110	9	FAN	EXERCISE2	40	50
	492	37.09	36.86	36.40	114	9	FAN	EXERCISE2	40	50
	493	37.09	36.88	36.42	114	9	FAN	EXERCISE2	40	50
	494	37.10	36.90	36.45	113	9	FAN	EXERCISE2	40	50
	495	37.11	36.93	36.45	114	9	FAN	EXERCISE2	40	50
	496	37.11	36.95	36.45	114	9	FAN	EXERCISE2	40	50
	497	37.10	36.95	36.46	115	9	FAN	EXERCISE2	40	50
	498	37.10	36.96	36.47	116	9	FAN	EXERCISE2	40	50
	499	37.10	36.95	36.48	118	9	FAN	EXERCISE2	40	50
	500	37.11	36.95	36.49	115	9	FAN	EXERCISE2	40	50
	501	37.11	36.96	36.52	117	9	FAN	EXERCISE2	40	50
	502	37.12	36.96	36.53	120	9	FAN	EXERCISE2	40	50
	503	37.11	36.96	36.53	118	9	FAN	EXERCISE2	40	50
	504	37.11	36.97	36.56	117	9	FAN	EXERCISE2	40	50
	505	37.12	36.99	36.59	121	9	FAN	EXERCISE2	40	50
	506	37.13	36.91	36.60	118	9	FAN	EXERCISE2	40	50
	507	37.13	36.89	36.60	116	9	FAN	EXERCISE2	40	50
	508	37.13	36.98	36.60	117	9	FAN	EXERCISE2	40	50
	509	37.14	36.99	36.60	116	9	FAN	EXERCISE2	40	50
	510	37.13	36.97	36.60	116	9	FAN	EXERCISE2	40	50
85	511	37.13	36.98	36.60	116	9	FAN	EXERCISE2	40	50
	512	37.14	36.98	36.61	118	9	FAN	EXERCISE2	40	50
	513	37.14	36.99	36.62	119	9	FAN	EXERCISE2	40	50
	514	37.14	37.00	36.62	120	9	FAN	EXERCISE2	40	50
	515	37.14	37.00	36.61	117	9	FAN	EXERCISE2	40	50
	516	37.15	36.99	36.60	116	9	FAN	EXERCISE2	40	50
	517	37.14	36.98	36.59	114	9	FAN	EXERCISE2	40	50
	518	37.14	37.02	36.59	113	9	FAN	EXERCISE2	40	50

min	number	Tre	Tes	Tsk-head	HR	ID	condition	period	Ta	RH
	519	37.14	37.02	36.59	117	9	FAN	EXERCISE2	40	50
	520	37.14	36.86	36.59	117	9	FAN	EXERCISE2	40	50
	521	37.14	36.85	36.58	114	9	FAN	EXERCISE2	40	50
	522	37.14	37.00	36.57	116	9	FAN	EXERCISE2	40	50
	523	37.14	37.02	36.57	115	9	FAN	EXERCISE2	40	50
	524	37.15	36.98	36.57	116	9	FAN	EXERCISE2	40	50
	525	37.15	36.99	36.58	116	9	FAN	EXERCISE2	40	50
	526	37.15	36.97	36.59	118	9	FAN	EXERCISE2	40	50
	527	37.15	36.92	36.58	118	9	FAN	EXERCISE2	40	50
	528	37.14	36.91	36.58	118	9	FAN	EXERCISE2	40	50
	529	37.15	36.94	36.58	120	9	FAN	EXERCISE2	40	50
	530	37.16	36.97	36.59	120	9	FAN	EXERCISE2	40	50
	531	37.15	36.98	36.61	120	9	FAN	EXERCISE2	40	50
	532	37.15	36.97	36.61	124	9	FAN	EXERCISE2	40	50
	533	37.15	36.96	36.61	123	9	FAN	EXERCISE2	40	50
	534	37.16	36.97	36.63	121	9	FAN	EXERCISE2	40	50
	535	37.16	36.94	36.63	115	9	FAN	EXERCISE2	40	50
	536	37.15	36.98	36.61	116	9	FAN	EXERCISE2	40	50
	537	37.15	37.03	36.61	117	9	FAN	EXERCISE2	40	50
	538	37.15	37.02	36.63	122	9	FAN	EXERCISE2	40	50
	539	37.15	37.02	36.66	122	9	FAN	EXERCISE2	40	50
	540	37.16	37.02	36.68	121	9	FAN	EXERCISE2	40	50
90	541	37.16	37.01	36.67	119	9	FAN	EXERCISE2	40	50
	542	37.16	37.00	36.67	121	9	FAN	EXERCISE2	40	50
	543	37.16	37.04	36.66	120	9	FAN	EXERCISE2	40	50
	544	37.16	37.01	36.63	120	9	FAN	EXERCISE2	40	50
	545	37.16	37.00	36.62	118	9	FAN	EXERCISE2	40	50
	546	37.16	37.02	36.64	117	9	FAN	EXERCISE2	40	50
	547	37.16	37.04	36.64	118	9	FAN	EXERCISE2	40	50
	548	37.16	37.04	36.65	120	9	FAN	EXERCISE2	40	50
	549	37.16	37.04	36.66	120	9	FAN	EXERCISE2	40	50
	550	37.16	37.08	36.66	121	9	FAN	EXERCISE2	40	50
	551	37.16	37.06	36.66	122	9	FAN	EXERCISE2	40	50
	552	37.15	37.03	36.64	120	9	FAN	EXERCISE2	40	50
	553	37.15	37.04	36.64	119	9	FAN	EXERCISE2	40	50
	554	37.16	37.05	36.66	120	9	FAN	EXERCISE2	40	50
	555	37.16	37.06	36.67	118	9	FAN	EXERCISE2	40	50
	556	37.16	37.08	36.68	120	9	FAN	EXERCISE2	40	50
	557	37.17	37.06	36.69	121	9	FAN	EXERCISE2	40	50
	558	37.17	37.07	36.69	120	9	FAN	EXERCISE2	40	50
	559	37.17	37.07	36.69	122	9	FAN	EXERCISE2	40	50
	560	37.17	37.07	36.69	118	9	FAN	EXERCISE2	40	50
	561	37.17	37.07	36.65	113	9	FAN	EXERCISE2	40	50
	562	37.17	37.04	36.62	116	9	FAN	EXERCISE2	40	50
	563	37.17	37.07	36.62	116	9	FAN	EXERCISE2	40	50
	564	37.17	37.05	36.65	117	9	FAN	EXERCISE2	40	50

min	number	Tre	Tes	Tsk-head	HR	ID	condition	period	Ta	RH
	565	37.17	36.98	36.69	119	9	FAN	EXERCISE2	40	50
	566	37.18	37.01	36.71	121	9	FAN	EXERCISE2	40	50
	567	37.17	37.03	36.71	119	9	FAN	EXERCISE2	40	50
	568	37.18	37.04	36.71	121	9	FAN	EXERCISE2	40	50
	569	37.19	37.07	36.71	121	9	FAN	EXERCISE2	40	50
	570	37.19	37.08	36.70	120	9	FAN	EXERCISE2	40	50
95	571	37.19	37.08	36.69	119	9	FAN	EXERCISE2	40	50
	572	37.19	37.10	36.69	118	9	FAN	EXERCISE2	40	50
	573	37.19	37.13	36.68	120	9	FAN	EXERCISE2	40	50
	574	37.19	37.11	36.68	120	9	FAN	EXERCISE2	40	50
	575	37.19	37.09	36.66	122	9	FAN	EXERCISE2	40	50
	576	37.19	37.09	36.60	120	9	FAN	EXERCISE2	40	50
	577	37.19	37.10	36.56	118	9	FAN	EXERCISE2	40	50
	578	37.19	37.09	36.55	116	9	FAN	EXERCISE2	40	50
	579	37.20	37.09	36.54	117	9	FAN	EXERCISE2	40	50
	580	37.20	37.06	36.53	118	9	FAN	EXERCISE2	40	50
	581	37.20	37.07	36.51	117	9	FAN	EXERCISE2	40	50
	582	37.20	37.12	36.48	117	9	FAN	EXERCISE2	40	50
	583	37.21	37.11	36.48	117	9	FAN	EXERCISE2	40	50
	584	37.21	37.01	36.50	118	9	FAN	EXERCISE2	40	50
	585	37.21	36.95	36.51	121	9	FAN	EXERCISE2	40	50
	586	37.22	37.02	36.51	122	9	FAN	EXERCISE2	40	50
	587	37.21	37.06	36.49	119	9	FAN	EXERCISE2	40	50
	588	37.21	37.08	36.50	120	9	FAN	EXERCISE2	40	50
	589	37.22	37.08	36.53	119	9	FAN	EXERCISE2	40	50
	590	37.23	37.08	36.54	121	9	FAN	EXERCISE2	40	50
	591	37.24	37.10	36.54	121	9	FAN	EXERCISE2	40	50
	592	37.24	37.12	36.51	122	9	FAN	EXERCISE2	40	50
	593	37.23	37.10	36.49	122	9	FAN	EXERCISE2	40	50
	594	37.23	37.09	36.52	121	9	FAN	EXERCISE2	40	50
	595	37.23	37.11	36.53	122	9	FAN	EXERCISE2	40	50
	596	37.24	37.06	36.53	121	9	FAN	EXERCISE2	40	50
	597	37.24	37.01	36.51	122	9	FAN	EXERCISE2	40	50
	598	37.25	37.09	36.49	122	9	FAN	EXERCISE2	40	50
	599	37.25	37.13	36.49	123	9	FAN	EXERCISE2	40	50
	600	37.25	37.11	36.52	122	9	FAN	EXERCISE2	40	50
100	601	37.25	37.11	36.52	120	9	FAN	EXERCISE2	40	50
	602	37.24	37.12	36.51	119	9	FAN	EXERCISE2	40	50
	603	37.24	37.12	36.51	120	9	FAN	EXERCISE2	40	50
	604	37.25	37.11	36.49	122	9	FAN	EXERCISE2	40	50
	605	37.25	37.11	36.49	123	9	FAN	EXERCISE2	40	50
	606	37.26	37.13	36.52	123	9	FAN	EXERCISE2	40	50
	607	37.25	37.14	36.53	123	9	FAN	EXERCISE2	40	50
	608	37.25	37.14	36.53	123	9	FAN	EXERCISE2	40	50
	609	37.25	37.15	36.53	121	9	FAN	EXERCISE2	40	50
	610	37.26	37.14	36.51	122	9	FAN	EXERCISE2	40	50

min	number	Tre	Tes	Tsk-head	HR	ID	condition	period	Ta	RH
	611	37.25	37.15	36.50	123	9	FAN	EXERCISE2	40	50
	612	37.25	37.16	36.52	124	9	FAN	EXERCISE2	40	50
	613	37.26	37.15	36.52	120	9	FAN	EXERCISE2	40	50
	614	37.26	37.16	36.51	124	9	FAN	EXERCISE2	40	50
	615	37.26	37.16	36.49	125	9	FAN	EXERCISE2	40	50
	616	37.26	37.15	36.49	125	9	FAN	EXERCISE2	40	50
	617	37.26	37.17	36.50	125	9	FAN	EXERCISE2	40	50
	618	37.27	37.16	36.51	126	9	FAN	EXERCISE2	40	50
	619	37.28	37.15	36.52	126	9	FAN	EXERCISE2	40	50
	620	37.28	37.17	36.51	124	9	FAN	EXERCISE2	40	50
	621	37.28	37.16	36.51	122	9	FAN	EXERCISE2	40	50
	622	37.28	37.14	36.53	122	9	FAN	EXERCISE2	40	50
	623	37.28	37.13	36.53	123	9	FAN	EXERCISE2	40	50
	624	37.28	37.12	36.52	122	9	FAN	EXERCISE2	40	50
	625	37.28	37.16	36.51	123	9	FAN	EXERCISE2	40	50
	626	37.29	37.16	36.51	124	9	FAN	EXERCISE2	40	50
	627	37.29	37.15	36.53	124	9	FAN	EXERCISE2	40	50
	628	37.29	37.17	36.55	122	9	FAN	EXERCISE2	40	50
	629	37.29	37.17	36.54	122	9	FAN	EXERCISE2	40	50
	630	37.29	37.17	36.53	121	9	FAN	EXERCISE2	40	50
105	631	37.30	37.17	36.58	122	9	FAN	EXERCISE2	40	50
	632	37.31	37.16	36.62	120	9	FAN	EXERCISE2	40	50
	633	37.31	37.17	36.61	121	9	FAN	EXERCISE2	40	50
	634	37.31	37.15	36.59	120	9	FAN	EXERCISE2	40	50
	635	37.31	37.16	36.58	122	9	FAN	EXERCISE2	40	50
	636	37.31	37.21	36.58	120	9	FAN	EXERCISE2	40	50
	637	37.32	37.19	36.57	120	9	FAN	EXERCISE2	40	50
	638	37.31	37.17	36.57	121	9	FAN	EXERCISE2	40	50
	639	37.31	37.20	36.58	122	9	FAN	EXERCISE2	40	50
	640	37.32	37.22	36.58	122	9	FAN	EXERCISE2	40	50
	641	37.33	37.24	36.59	123	9	FAN	EXERCISE2	40	50
	642	37.33	37.23	36.59	123	9	FAN	EXERCISE2	40	50
	643	37.32	37.22	36.60	121	9	FAN	EXERCISE2	40	50
	644	37.32	37.20	36.60	121	9	FAN	REST3	28	50
	645	37.32	37.21	36.59	123	9	FAN	REST3	28	50
	646	37.31	37.28	36.38	116	9	FAN	REST3	28	50
	647	37.32	37.23	36.14	115	9	FAN	REST3	28	50
	648	37.34	37.20	36.02	103	9	FAN	REST3	28	50
	649	37.35	37.26	35.92	98	9	FAN	REST3	28	50
	650	37.35	37.28	35.82	103	9	FAN	REST3	28	50
	651	37.35	37.29	35.73	87	9	FAN	REST3	28	50
	652	37.35	37.29	35.64	84	9	FAN	REST3	28	50
	653	37.35	37.29	35.54	90	9	FAN	REST3	28	50
	654	37.35	37.29	35.44	90	9	FAN	REST3	28	50
	655	37.34	37.29	35.37	88	9	FAN	REST3	28	50
	656	37.34	37.30	35.31	94	9	FAN	REST3	28	50

min	number	Tre	Tes	Tsk-head	HR	ID	condition	period	Ta	RH
	657	37.35	37.30	35.26	86	9	FAN	REST3	28	50
	658	37.35	37.29	35.20	83	9	FAN	REST3	28	50
	659	37.35	37.29	35.13	87	9	FAN	REST3	28	50
	660	37.35	37.30	35.05	79	9	FAN	REST3	28	50
110	661	37.35	37.31	34.98	80	9	FAN	REST3	28	50
	662	37.35	37.31	34.91	81	9	FAN	REST3	28	50
	663	37.35	37.31	34.83	81	9	FAN	REST3	28	50
	664	37.35	37.30	34.76	83	9	FAN	REST3	28	50
	665	37.35	37.29	34.70	81	9	FAN	REST3	28	50
	666	37.34	37.30	34.64	79	9	FAN	REST3	28	50
	667	37.34	37.30	34.61	80	9	FAN	REST3	28	50
	668	37.35	37.29	34.60	81	9	FAN	REST3	28	50
	669	37.35	37.20	34.58	87	9	FAN	REST3	28	50
	670	37.35	37.17	34.54	83	9	FAN	REST3	28	50
	671	37.35	37.24	34.50	80	9	FAN	REST3	28	50
	672	37.34	37.25	34.46	79	9	FAN	REST3	28	50
	673	37.34	37.25	34.40	85	9	FAN	REST3	28	50
	674	37.34	37.25	34.34	86	9	FAN	REST3	28	50
	675	37.34	37.25	34.31	84	9	FAN	REST3	28	50
	676	37.34	37.25	34.27	83	9	FAN	REST3	28	50
	677	37.34	37.26	34.25	82	9	FAN	REST3	28	50
	678	37.34	37.28	34.23	87	9	FAN	REST3	28	50
	679	37.34	37.31	34.19	87	9	FAN	REST3	28	50
	680	37.34	37.30	34.20	86	9	FAN	REST3	28	50
	681	37.34	37.25	34.19	84	9	FAN	REST3	28	50
	682	37.34	37.23	34.14	86	9	FAN	REST3	28	50
	683	37.34	37.23	34.11	87	9	FAN	REST3	28	50
	684	37.34	37.15	34.18	91	9	FAN	REST3	28	50
	685	37.33	37.14	34.28	84	9	FAN	REST3	28	50
	686	37.34	37.23	34.33	85	9	FAN	REST3	28	50
	687	37.35	37.25	34.39	91	9	FAN	REST3	28	50
	688	37.35	37.23	34.46	89	9	FAN	REST3	28	50
	689	37.35	37.22	34.50	82	9	FAN	REST3	28	50
	690	37.36	37.23	34.53	81	9	FAN	REST3	28	50
115	691	37.35	37.24	34.58	79	9	FAN	REST3	28	50
	692	37.35	37.24	34.60	79	9	FAN	REST3	28	50
	693	37.35	37.24	34.63	86	9	FAN	REST3	28	50
	694	37.35	37.26	34.69	87	9	FAN	REST3	28	50
	695	37.36	37.26	34.73	84	9	FAN	REST3	28	50
	696	37.36	37.26	34.79	84	9	FAN	REST3	28	50
	697	37.36	37.25	34.82	85	9	FAN	REST3	28	50
	698	37.36	37.24	34.83	85	9	FAN	REST3	28	50
	699	37.35	37.24	34.85	85	9	FAN	REST3	28	50
	700	37.36	37.25	34.88	89	9	FAN	REST3	28	50
	701	37.36	37.24	34.93	92	9	FAN	REST3	28	50
	702	37.36	37.22	34.97	91	9	FAN	REST3	28	50

min	number	Tre	Tes	Tsk-head	HR	ID	condition	period	Ta	RH
	703	37.37	37.21	34.99	84	9	FAN	REST3	28	50
	704	37.37	37.15	35.04	87	9	FAN	REST3	28	50
	705	37.37	37.13	35.08	90	9	FAN	REST3	28	50
	706	37.36	37.14	35.09	89	9	FAN	REST3	28	50
	707	37.36	37.12	35.09	92	9	FAN	REST3	28	50
	708	37.37	37.11	35.08	90	9	FAN	REST3	28	50
	709	37.36	37.15	35.12	91	9	FAN	REST3	28	50

timing	RPE	thermal sensation	thermal comfort	ID	condition
t_1	7	1	1	1	CON
t_2	12	2	2	1	CON
t_3	13	2	2	1	CON
t_4	15	3	2	1	CON
t_5	11	-2	1	1	CON
t_6	11	1	1	1	CON
t_7	13	2	2	1	CON
t_8	15	2	2	1	CON
t_9	17	3	2	1	CON
t_{10}	11	-1	1	1	CON
t_1	10	3	2	2	CON
t_2	9	3	2	2	CON
t_3	12	3	2	2	CON
t_4	12	3	2	2	CON
t_5	8	0	0	2	CON
t_6	9	3	1	2	CON
t_7	11	3	1	2	CON
t_8	12	3	2	2	CON
t_9	14	3	2	2	CON
t_{10}	9	0	0	2	CON
t_1	8	2	1	3	CON
t_2	12	3	2	3	CON
t_3	13	3	2	3	CON
t_4	14	4	4	3	CON
t_5	9	-1	0	3	CON
t_6	9	2	1	3	CON
t_7	13	3	2	3	CON
t_8	15	4	3	3	CON
t_9	17	4	4	3	CON
t_{10}	11	-2	0	3	CON
t_1	6	3	1	4	CON
t_2	11	3	2	4	CON
t_3	13	3	2	4	CON
t_4	15	3	2	4	CON
t_5	9	-2	0	4	CON

timing	RPE	thermal sensation	thermal comfort	ID	condition
t_6	11	3	1	4	CON
t_7	13	3	2	4	CON
t_8	15	4	2	4	CON
t_9	16	4	3	4	CON
t_{10}	9	-2	0	4	CON
t_1	9	3	1	5	CON
t_2	11	3	2	5	CON
t_3	13	3	2	5	CON
t_4	12	3	2	5	CON
t_5	7	-2	0	5	CON
t_6	7	1	1	5	CON
t_7	11	2	1	5	CON
t_8	12	3	2	5	CON
t_9	13	4	2	5	CON
t_{10}	11	-2	0	5	CON
t_1	7	2	0	6	CON
t_2	11	3	1	6	CON
t_3	12	3	1	6	CON
t_4	13	4	2	6	CON
t_5	9	-2	0	6	CON
t_6	9	0	0	6	CON
t_7	11	1	0	6	CON
t_8	13	3	2	6	CON
t_9	15	4	3	6	CON
t_{10}	9	-2	0	6	CON
t_1	7	2	1	7	CON
t_2	8	2	0	7	CON
t_3	9	3	1	7	CON
t_4	9	3	1	7	CON
t_5	7	0	0	7	CON
t_6	7	1	0	7	CON
t_7	9	3	1	7	CON
t_8	10	3	1	7	CON
t_9	11	3	2	7	CON
t_{10}	7	0	0	7	CON

timing	RPE	thermal sensation	thermal comfort	ID	condition
t_1	9	3	1	8	CON
t_2	11	4	2	8	CON
t_3	13	4	3	8	CON
t_4	15	4	3	8	CON
t_5	7	-2	0	8	CON
t_6	7	1	1	8	CON
t_7	11	3	2	8	CON
t_8	16	4	4	8	CON
t_9	18	4	4	8	CON
t_{10}	11	-2	0	8	CON
t_1	10	2	1	9	CON
t_2	13	3	2	9	CON
t_3	15	3	2	9	CON
t_4	16	4	3	9	CON
t_5	10	-1	0	9	CON
t_6	11	2	1	9	CON
t_7	15	3	2	9	CON
t_8	17	4	3	9	CON
t_9	18	4	3	9	CON
t_{10}	14	0	1	9	CON
t_1	11	-1	0	1	VEST
t_2	12	0	1	1	VEST
t_3	12	1	1	1	VEST
t_4	13	2	1	1	VEST
t_5	11	-2	0	1	VEST
t_6	11	-1	0	1	VEST
t_7	12	1	2	1	VEST
t_8	13	1	1	1	VEST
t_9	13	2	1	1	VEST
t_{10}	12	-2	0	1	VEST
t_1	9	-3	1	2	VEST
t_2	11	0	1	2	VEST
t_3	12	1	1	2	VEST
t_4	11	0	0	2	VEST
t_5	7	-2	0	2	VEST

timing	RPE	thermal sensation	thermal comfort	ID	condition
t_6	11	-2	1	2	VEST
t_7	11	0	0	2	VEST
t_8	12	1	1	2	VEST
t_9	12	2	1	2	VEST
t_{10}	7	-2	0	2	VEST
t_1	7	0	1	3	VEST
t_2	12	2	1	3	VEST
t_3	12	2	1	3	VEST
t_4	13	2	2	3	VEST
t_5	10	-2	0	3	VEST
t_6	10	2	0	3	VEST
t_7	12	2	1	3	VEST
t_8	12	2	1	3	VEST
t_9	13	2	1	3	VEST
t_{10}	9	-3	0	3	VEST
t_1	6	-1	1	4	VEST
t_2	12	2	1	4	VEST
t_3	13	3	2	4	VEST
t_4	15	3	2	4	VEST
t_5	9	-2	0	4	VEST
t_6	9	-1	1	4	VEST
t_7	13	2	1	4	VEST
t_8	14	2	1	4	VEST
t_9	14	3	1	4	VEST
t_{10}	9	-2	0	4	VEST
t_1	11	-1	0	5	VEST
t_2	12	0	1	5	VEST
t_3	13	0	2	5	VEST
t_4	13	0	2	5	VEST
t_5	11	-2	0	5	VEST
t_6	11	-1	0	5	VEST
t_7	12	0	1	5	VEST
t_8	13	0	2	5	VEST
t_9	13	1	2	5	VEST
t_{10}	11	-2	0	5	VEST

timing	RPE	thermal sensation	thermal comfort	ID	condition
t_1	7	-2	0	6	VEST
t_2	11	-1	0	6	VEST
t_3	12	2	1	6	VEST
t_4	12	2	1	6	VEST
t_5	7	-2	0	6	VEST
t_6	7	-2	0	6	VEST
t_7	11	-1	0	6	VEST
t_8	12	1	0	6	VEST
t_9	13	2	1	6	VEST
t_{10}	7	-2	0	6	VEST
t_1	7	0	0	7	VEST
t_2	8	0	0	7	VEST
t_3	9	0	0	7	VEST
t_4	9	0	0	7	VEST
t_5	7	0	0	7	VEST
t_6	7	0	0	7	VEST
t_7	8	0	0	7	VEST
t_8	9	1	0	7	VEST
t_9	9	1	0	7	VEST
t_{10}	7	0	0	7	VEST
t_1	7	1	0	8	VEST
t_2	9	1	0	8	VEST
t_3	11	1	1	8	VEST
t_4	11	1	1	8	VEST
t_5	7	-2	0	8	VEST
t_6	7	0	0	8	VEST
t_7	10	1	1	8	VEST
t_8	11	1	1	8	VEST
t_9	11	1	1	8	VEST
t_{10}	7	-2	0	8	VEST
t_1	7	-2	0	9	VEST
t_2	13	1	1	9	VEST
t_3	15	2	1	9	VEST
t_4	16	3	2	9	VEST
t_5	7	-3	2	9	VEST

timing	RPE	thermal sensation	thermal comfort	ID	condition
t_6	7	0	0	9	VEST
t_7	13	1	1	9	VEST
t_8	17	3	2	9	VEST
t_9	18	3	2	9	VEST
t_{10}	12	-3	3	9	VEST
t_1	11	1	1	1	FAN
t_2	13	1	1	1	FAN
t_3	13	2	2	1	FAN
t_4	13	2	2	1	FAN
t_5	11	-1	1	1	FAN
t_6	13	1	1	1	FAN
t_7	13	1	1	1	FAN
t_8	13	2	2	1	FAN
t_9	14	2	3	1	FAN
t_{10}	12	-1	0	1	FAN
t_1	9	3	1	2	FAN
t_2	12	2	1	2	FAN
t_3	12	1	1	2	FAN
t_4	13	1	1	2	FAN
t_5	8	-1	0	2	FAN
t_6	9	2	1	2	FAN
t_7	11	1	1	2	FAN
t_8	12	1	1	2	FAN
t_9	12	1	1	2	FAN
t_{10}	9	-1	0	2	FAN
t_1	8	2	1	3	FAN
t_2	11	1	1	3	FAN
t_3	13	1	1	3	FAN
t_4	15	1	1	3	FAN
t_5	9	-2	0	3	FAN
t_6	10	1	1	3	FAN
t_7	12	1	1	3	FAN
t_8	13	1	1	3	FAN
t_9	15	1	1	3	FAN
t_{10}	11	-2	0	3	FAN

timing	RPE	thermal sensation	thermal comfort	ID	condition
t_1	7	1	1	4	FAN
t_2	9	2	1	4	FAN
t_3	13	2	1	4	FAN
t_4	13	2	1	4	FAN
t_5	9	-2	0	4	FAN
t_6	9	1	1	4	FAN
t_7	12	2	1	4	FAN
t_8	13	2	1	4	FAN
t_9	13	2	1	4	FAN
t_{10}	9	-3	0	4	FAN
t_1	11	2	1	5	FAN
t_2	11	0	1	5	FAN
t_3	11	0	1	5	FAN
t_4	11	0	1	5	FAN
t_5	11	-1	0	5	FAN
t_6	11	1	1	5	FAN
t_7	11	0	1	5	FAN
t_8	11	0	1	5	FAN
t_9	11	0	1	5	FAN
t_{10}	11	0	1	5	FAN
t_1	9	-1	0	6	FAN
t_2	11	2	1	6	FAN
t_3	11	2	1	6	FAN
t_4	12	2	1	6	FAN
t_5	9	-2	0	6	FAN
t_6	9	-1	0	6	FAN
t_7	11	1	1	6	FAN
t_8	12	2	1	6	FAN
t_9	13	3	2	6	FAN
t_{10}	9	-2	0	6	FAN
t_1	6	-1	0	7	FAN
t_2	7	-1	0	7	FAN
t_3	7	-1	0	7	FAN
t_4	7	-1	0	7	FAN
t_5	6	-1	0	7	FAN

timing	RPE	thermal sensation	thermal comfort	ID	condition
t_6	6	-1	0	7	FAN
t_7	7	-1	0	7	FAN
t_8	7	-1	0	7	FAN
t_9	7	-1	0	7	FAN
t_{10}	6	-1	0	7	FAN
t_1	7	2	1	8	FAN
t_2	11	2	1	8	FAN
t_3	13	2	1	8	FAN
t_4	13	2	1	8	FAN
t_5	7	-2	0	8	FAN
t_6	7	1	1	8	FAN
t_7	10	2	1	8	FAN
t_8	13	2	1	8	FAN
t_9	13	1	1	8	FAN
t_{10}	11	1	1	8	FAN
t_1	7	2	1	9	FAN
t_2	12	3	2	9	FAN
t_3	15	4	3	9	FAN
t_4	17	4	4	9	FAN
t_5	10	-3	0	9	FAN
t_6	10	2	1	9	FAN
t_7	17	3	3	9	FAN
t_8	18	4	4	9	FAN
t_9	19	4	4	9	FAN
t_{10}	10	-3	1	9	FAN

sweating	ID	condition
0.977	1	CON
0.824	2	CON
1.098	3	CON
0.84	4	CON
0.565	5	CON
1.303	6	CON
0.928	7	CON
1.767	8	CON
1.49	9	CON
0.683	1	VEST
0.795	2	VEST
0.695	3	VEST
0.647	4	VEST
0.464	5	VEST
1.069	6	VEST
0.581	7	VEST
0.879	8	VEST
1.389	9	VEST
-0.317	1	FAN
0.671	2	FAN
0.773	3	FAN
0.592	4	FAN
0.479	5	FAN
1.114	6	FAN
0.808	7	FAN
0.914	8	FAN
1.218	9	FAN
